# Transposon mediated functional genomic screening for BRAF inhibitor resistance reveals convergent Hippo and MAPK pathway activation events

**DOI:** 10.1038/s41598-025-86694-5

**Published:** 2025-01-24

**Authors:** Li Chen, Iulian Pruteanu-Malinici, Anahita Dastur, Xunqin Yin, Dennie Frederick, Ruslan I. Sadreyev, Cyril H. Benes

**Affiliations:** 1 Massachusetts General Hospital Cancer Center, Harvard Medical School, Charlestown, MA 02129 USA; 2 Department of Molecular Biology, Massachusetts General Hospital, Harvard Medical School, Boston, MA 02115 USA; 3 Present Address: Flagship Pioneering, Cambridge, MA USA; 4 Present Address: Sonata Therapeutics, Watertown, MA USA; 5 Present Address: Broad Institute, Cambridge, MA USA; 6 Present Address: Treeline Biosciences, San Diego, CA USA

**Keywords:** BRAF inhibitor resistance, Transposon mutagenesis screen, Hippo pathway, TAZ, NEDD4L, Cancer genomics, Cancer therapy, Skin cancer, Genome informatics, High-throughput screening, Transposition, Cancer genomics, Functional genomics, Gene regulation, Genomics, Genomic engineering, Regulatory networks, Systems analysis, Cancer genomics, Cancer therapy, Skin cancer, Molecular biology

## Abstract

Genotype-informed anticancer therapies such as BRAF inhibitors can show remarkable clinical efficacy in BRAF-mutant melanoma; however, drug resistance poses a major hurdle to successful cancer treatment. Many resistance events to targeted therapies have been identified, suggesting a complex path to improve therapeutics. Here, we showed the utility of a *piggyBac* transposon activation mutagenesis screen for the efficient identification of genes that are resistant to BRAF inhibition in melanoma. Although several forward genetic screens performed in the same context have identified a broad range of resistance genes that poorly overlap, an integrative analysis revealed a much smaller functional diversity of resistance mechanisms, including reactivation of the MAPK pathway, PI3K-AKT pathway, and Hippo pathway, suggesting that a relatively small number of therapeutic strategies might overcome resistance manifested by a large gene set. Moreover, we illustrated the pivotal role of the Hippo pathway effector TAZ (encoded by the *WWTR1* gene) in mediating BRAF inhibition resistance through transcriptional regulation of receptor tyrosine kinases and through interactions with the E3 ubiquitin ligase NEDD4L.

## Introduction

A vast array of genetic and nongenetic events, such as point mutation, overexpression, translocation, and alternative splicing, occurring across many genes have been shown to cause resistance to BRAF inhibitors in melanoma and other tumor types^1–9^. Although the analysis of clinical samples provides unique insight into therapeutically relevant resistance tumor heterogeneity, limitations in the availability of clinical samples and functional characterization of the human genome combined with a high number of passenger mutations limit the potential to identify low occurrence resistance events^10–13^. To prospectively identify genes that can promote resistance to BRAF and MEK inhibition several landmark studies using genetic screens in the context of BRAF^V600E^ mutant melanoma have been performed. These include gain-of-function (GoF) open reading frame (ORF) and aptamer-mediated dCAS9-activator screens, and loss-of-function (LoF) CRISPR knockout and shRNA screens^14–17^. These screens leverage collections of individually made elements, are rather labor-intensive and are associated with relatively high costs. Even the most advanced of these screening approaches have limitations leading to incomplete saturation; for example, an ORF library is typically limited to one isoform per gene, and noncoding GoF events are poorly covered by most screens. Interestingly, these screens and several transposon mutagenesis screens that have been previously performed in the BRAF^V600E^ melanoma cell lines offer insight into the complexity and diversity of the genes that can mediate resistance to a given therapeutic agent in a specific genomic context. Comparison of the results of these screens revealed different gene sets identified even by screens that were functionally analogous (gain- or loss-of-function) and well validated^18–20^. This raises several interesting questions such as whether screens that are genome-wide by design have indeed reached a substantial level of saturation, how many genes in total can mediate resistance and whether they fall into common resistance pathways.

We previously developed an in vitro* piggyBac* (PB) transposon activation mutagenesis screen with unbiased genome-wide insertions that activated, and in some cases disrupted, endogenous genes and demonstrated its applications in cancer cell tolerance to the cytotoxic reagent paclitaxel and in host cell resistance to Ebola or coronaviruses^21,22^. Here, we used this approach to further study resistance to BRAF inhibition in BRAF^V600E^ melanoma.

## Results

### A transposon screen to identify BRAF inhibitor resistance genes

**Fig. 1:**
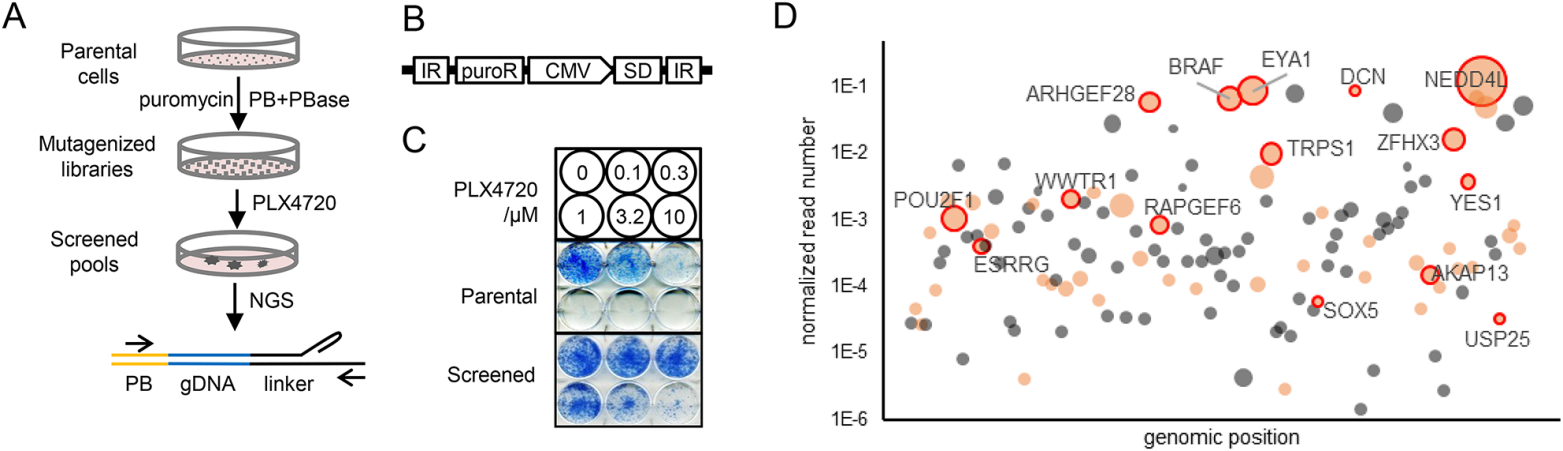
PB transposon mutagenesis screen. (**A**) Scheme of the PB mutagenesis screening process. Parental cancer cells were transfected with transposition (PB) and transposase (PBase) plasmids, passaged with puromycin to generate mutagenized libraries, and treated with PLX4720 to generate resistant colonies or pools. Insert sites were detected using linker-mediated PCR and NGS, with the shown amplicon structure of the transposon (PB), host genomic DNA (gDNA), linker, and PCR primers (arrows). (**B**) The PB transposition plasmid includes a cassette with an antibiotic selection marker (puroR) for transposon-mutagenized cell maintenance, a CMV promoter (CMV) and a splice donor (SD) for host gene transcription and alternative splicing, and two inverted repeats (IRs) for transposition. (**C**) Clonogenic assays showing the sensitive parental cells and PLX4720-resistant cells from transposon screen. (**D**) High confidence hits identified from resistant pools are displayed by genomic location (x-axis: chrs.1–22,x). The inserts for each gene were summarized, read numbers of each gene normalized to the total number of reads were plotted as y-coordinates, and the dot sizes represent the numbers of insertion events. Genes with a predicted activation effect are shown as orange bubbles, and candidates for follow-up validation assays are circled and labeled.

To identify genes conferring resistance to BRAF inhibition in melanoma, ten individual PB mutagenesis libraries were constructed by transfecting independently cultured PLX4720-sensitive BRAF^V600E^ A375 melanoma cells with the PB and transposase (PBase) plasmids (Fig. 1A, B). PLX4720 is an analog of vemurafenib (PLX4032), the first small molecule inhibitor drug clinically approved to treat BRAF-mutant advanced malignant melanoma^1^. Each library was treated with PLX4720 (Fig. 1C), and insertion sites were identified from each resistant pool by targeted deep sequencing.

To first gain insight into the breadth of target drug resistance events that can be captured with this approach, the overall distribution of insertions across the genome was analyzed. This analysis revealed that inserts were evenly distributed across the genome without major local preference, highlighting the genome-wide coverage ability of this approach (see also a previous report^21^). Supporting the mechanistic specificity of the insertion events, a strong enrichment of sequencing reads for a small number of loci was observed. The top 100 inserts represented most (> 99.5%) of the sequencing reads (**Fig. S1A**) of each pool and corresponded to 902 genomic loci associated with 767 genes (**Table S1**) based on most proximal genes (see below). We considered the top 132 genes of this aggregated list constituting 95% of sequencing reads, and including all 128 genes with multiple inserts, as high-confidence hits (Fig. 1D). We note however that some pathologically relevant genes (such as *PTEN*, ranked 134) might have been omitted by this prioritization strategy.

To confirm that the analytical approach identified driver rather than passenger events, 224 colonies, including 23 isolated directly from the primary screening plates and 201 derived by limiting dilution of the multipassaged individual resistant pools, were sequenced (**Table S2**). On average, 2.2 inserts (ranging from 1 to 12) were found per clone (**Fig. S1B**), indicating that co-enrichment of passenger inserts in resistant cells was infrequent and thus indicating that candidate resistance-driving genes should be readily identified by considering high read count genes and proximity to redundant insertion events.

To characterize the functional consequence of the insertions, we performed RNA-Seq on 10 clones and plotted the differential gene expression according to the insertion orientation and proximity (**Fig. S1C, D, Table S3**). This analysis indicated that, as predicted and consistent with our previous report^21^, expression variations mostly affected the nearest gene (13 of 16 inserts, including *LPP*, the second nearest gene after an undetectable antisense isoform *LPP-AS2*, *p* < 0.05, q < 0.05) and that upstream/sense-strand inserts result in activation of the host gene transcription. We also noted that an insertion as far as 48 kb robustly upregulated the expression of the nearest gene (*LPP)* (20.8-fold, *p* = 0). While genome structural and regulatory complexity are likely to impact how far a PB insertion can activate a gene, alternative splicing involving splice donor (SD) and acceptor (SA) site selection is affected by promoter activity and the transcription elongation rate^23,24^, and several human genes, such as dystrophin (*DMD*), indeed contain introns well over 100 kb. Thus, the capacity for distal activation demonstrated here is likely due to CMV-initiated strong transcriptional readthrough and the coupling of the ectopic SD to an endogenous SA and may therefore substantially reduce the number of clones needed for genome-wide coverage compared to the proximal effect alone. In addition to upstream sense-strand events, intragenic sense-strand inserts at the 5’ end of the gene or the protein coding sequence, effectively analogous to 5’ alternative splicing isoforms, were also observed to activate transcription in clones containing *NEDD4L, BRAF,* or *MCF2*.

Of the 132 high-confidence hits, 52 were associated with multiple incidences of nonidentical sense-strand, upstream or proximal, insertion events and thus most likely resulted in host gene overexpression (Fig. 1D**, Table S1**). Interestingly, the PB approach might also be capable of identifying resistance through LoF in addition to GOF events. Conceptually, LoF could occur either through insertion in the gene body or through insertion downstream of a gene in the opposite direction to transcription, leading to gene disruption or antisense RNA expression, among other mechanisms. In line with this possibility and with the previous identification of disruption of the *NPC1* gene in a similar transposon-mediated screen for viral invasion^22^, several potentially disruptive insertions were found in genes such as *GPC6 and PLS3*, and one was discovered in the well-characterized resistance gene *PTEN*, a lipid phosphatase that acts as a tumor suppressor to counteract the activity of phosphoinositide 3-kinases (PI3K) with PI3K activity promoting proliferation and survival (**Table S1-S3**). However, robust identification of such LoF driven resistance events is complicated by the broad range of insertion positions and disruption of gene structures that could underlie LoF. In addition, in the context of a diploid genome, LoF might only involve haploinsufficient genes, although these could actually constitute a large fraction of human genes^25^. Furthermore, cancer cell genomes are frequently not simply diploid due to whole genome duplication or other complex genomic rearrangements. For these different reasons, below, we focus on GoF events.

Confirming the ability of the PB approach to identify functionally relevant and drug target-related resistance events, *BRAF* (BRAF^V600E^, the direct target of inhibition, and in which A375 is homozygous for that mutation), was among the top hit genes with 8 insertion events (Fig. 1D**, Table S1**). Interestingly, although not the most prominent target in the primary screening, the majority (140/201) of subclones isolated by limiting dilution of the pools contained *BRAF* insertions (**Table S2**), implying that BRAF^V600E^ overexpression itself promotes a strong proliferative advantage, an observation in line with a previously published study^18^. Two insertion events were mapped to *RAF1* (*CRAF*), a paralog of *BRAF*, and a predicted activating insert was also found 2.7 kb upstream of the *KRAS* gene, the upstream activator of RAF kinases in the mitogen-activated protein kinase (MAPK) signaling cascade (**Table S1**). Interestingly, among the high-confidence hits, the only other kinase predicted to be upregulated was the SRC family tyrosine kinase gene *YES1*. SRC kinases, particularly SRC, FYN and YES (encoded by *YES1*), are indeed positive regulators of RAF kinases and are known to be upstream activators of the MAPK pathway in many contexts, including BRAF inhibitor resistance^26^. Other candidate resistance genes belong to a broad variety of functional classes. Multiple guanine exchange factors, including three RAP exchange factors (*RAPGEF2*, *RAPGEF4*, and *RAPGEF6*) and two Rho exchange factors (*VAV1* and *ARHGEF28*), were among the high-confidence hits, and another Rho exchange factor (*MCF2*) ranked 540 from the pools (**Table S1**), whose RNA expression was also induced 33-fold by an intragenic sense-strand insert in a clone (**Fig. S1D, Table S3**). Several transcriptional regulators were found among the most redundant hit genes such as *EYA1* (11 inserts), *POU2F1* (9 inserts), and the zinc finger proteins *ZFHX3* and *ZFHX4* (7 inserts each). Chromatin binding proteins (*ARID1A*, *SMC2*, *ACIN1*, and *PDS5A*) and other gene family members, such as sorting nexins (*SNX6* and *SNX14*), semaphorins (*SEMA3D* and *SMA6A*) and potassium (Kv) channel interacting proteins (*KCNIP1* and *KCNIP4*), were also identified. Notably, these genes do not colocalize in the host genome, suggesting functionally meaningful enrichment.

### Validation of resistance genes

**Fig. 2:**
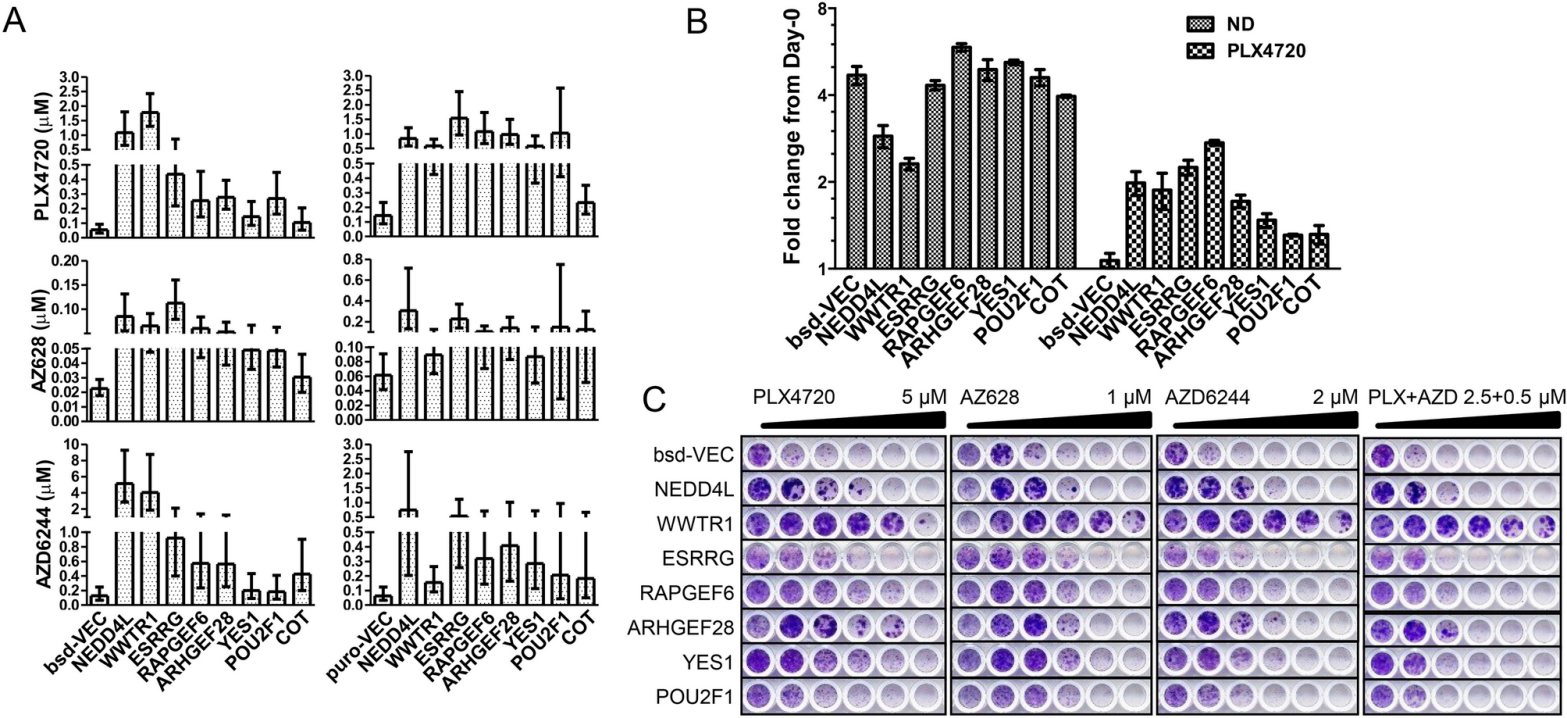
Effect of candidate resistance genes on cell proliferation evaluated by cDNA overexpression. (**A**) Half-maximal inhibition concentration (IC50) values of cells expressing the vector controls, candidate resistance genes, and a previously known resistance gene, *COT*. Experiments were performed on two sets of lentiviral vectors (left: blasticidin; right: puromycin). Values are means ± 95% confidence intervals (CIs). (**B**) Proliferation rate of cells transfected with candidate genes, either untreated (ND) or treated with 0.5 µM PLX4720. CellTiter-Glo measurements 4 days after drug treatment were normalized to the Day-0 values. Data are presented as mean ± standard error of the mean (SEM), n = 3. (**C**) Clonogenic assay with cells treated with the corresponding drugs at twofold serial dilutions.

To validate the results of the transposon screen we selected 15 gene candidates based on the predicted activation effect for single-gene ORF-mediated overexpression (circled and labeled in Fig. 1D). The expression of these genes was mediated through lentiviral infection in A375 cells, and cellular proliferation was measured in the presence of the BRAF inhibitor PLX4720, the pan-RAF inhibitor AZ628, and the MEK inhibitor AZD6244 (MEK is immediately downstream of RAF in the MAPK pathway) (**Fig. S2**). Seven candidates, *NEDD4L*, *WWTR1* (encoding TAZ, a Hippo pathway component), *ESRRG*, *RAPGEF6*, *ARHGEF28*, *YES1*, and *POU2F1* were further confirmed to enhance viability in the presence of inhibitors using dose titration assays (Fig. 2A), proliferation rate assays (Fig. 2B) and long-term clonogenic assays (Fig. 2C). As a frame of reference, the expression of these genes conferred a level of resistance to RAF and MEK inhibition comparable to that of the previously reported and well-validated resistance gene *COT*^27^. Supporting the specificity of the findings for BRAF inhibition, none of these genes induced resistance to the microtubule-disrupting agent paclitaxel while expression of the drug exporter *ABCB1* gene did, as expected (**Fig. S2B**)^21^.

We note that among the genes that were not validated, some were likely due to the limitations of the ORF clones. For example, *AKAP13* was only modestly overexpressed (1.5- to 2.2-fold greater than basal expression, **Fig. S2C**) but did induce partial resistance to PLX4720; *BRAF* wild-type cDNA also did not induce resistance, consistent with previously reported ORF screening results that did not identify wild-type *BRAF* as a resistance gene^15^, likely because only the V600 mutation allele overexpression can drive resistance^3^, which is what occurs upon transposon insertion in A375 cells. Furthermore, the available *NEDD4L* ORF construct used in the ORF screen and initially tested in our validation studies corresponded to a WW2 domain deletion isoform that failed to induce resistance (named herein NEDD4L-dWW2 to avoid confusion and further described below)^28^, highlighting the possibility that specific ORF inactivity or particular isoforms can contribute to false negatives in ORF based genetic studies. Overall, the results show a high rate of validation for the top transposon screen hits.

### Concordance of multiple genetic screens

The exact model of melanoma used here (the A375 cell line) was previously used to identify resistance genes via multiple screening modalities^14–17^. Surprisingly, the overlap between the lists of genes identified previously and the top 132 genes we identified was limited to 6 genes in the case of a previously reported ORF screen (*ESRRG*, *FOXP2*, *RAF1*, *RAPGEF4*, *VAV1*, and *WWTR1*) and 6 different genes in the case of a dCAS9-activator screen (*ATP10A*, *BCAS3*, *GLIS3*, *MECOM*, *PCDH7*, and *ZFHX4*). Although this could be explained at least in part by incomplete genome coverage of each library with some events functionally tested in only one of the screens, this very limited overlap across likely true positive hits (based on validation rates in the different studies) was still somewhat unexpected. For example, the genome-wide ORF screen should, in theory, capture the majority of predicted activated genes mapped to coding sequences in our screen. Interestingly, among the seven genes that we confirmed to induce resistance by overexpression experiments, *WWTR1* and *ESRRG* were successfully identified in the ORF resistance screen (z score ≥ the threshold level of 2.5 in that report); *RAPGEF6* narrowly missed the hit status (z score = 2.43), but *YES1* (z score = − 0.13) was not a hit. Finally, three (*NEDD4L*, *POU2F1*, and *ARHGEF28*) were among the genes that did not pass quality-control filters prior to screening. This illustrates the challenge of large genetic screens both in terms of functional coverage due to genomic bias or technical factors as well as inherently somewhat arbitrary choices of thresholds for hit calling.

**Fig. 3:**
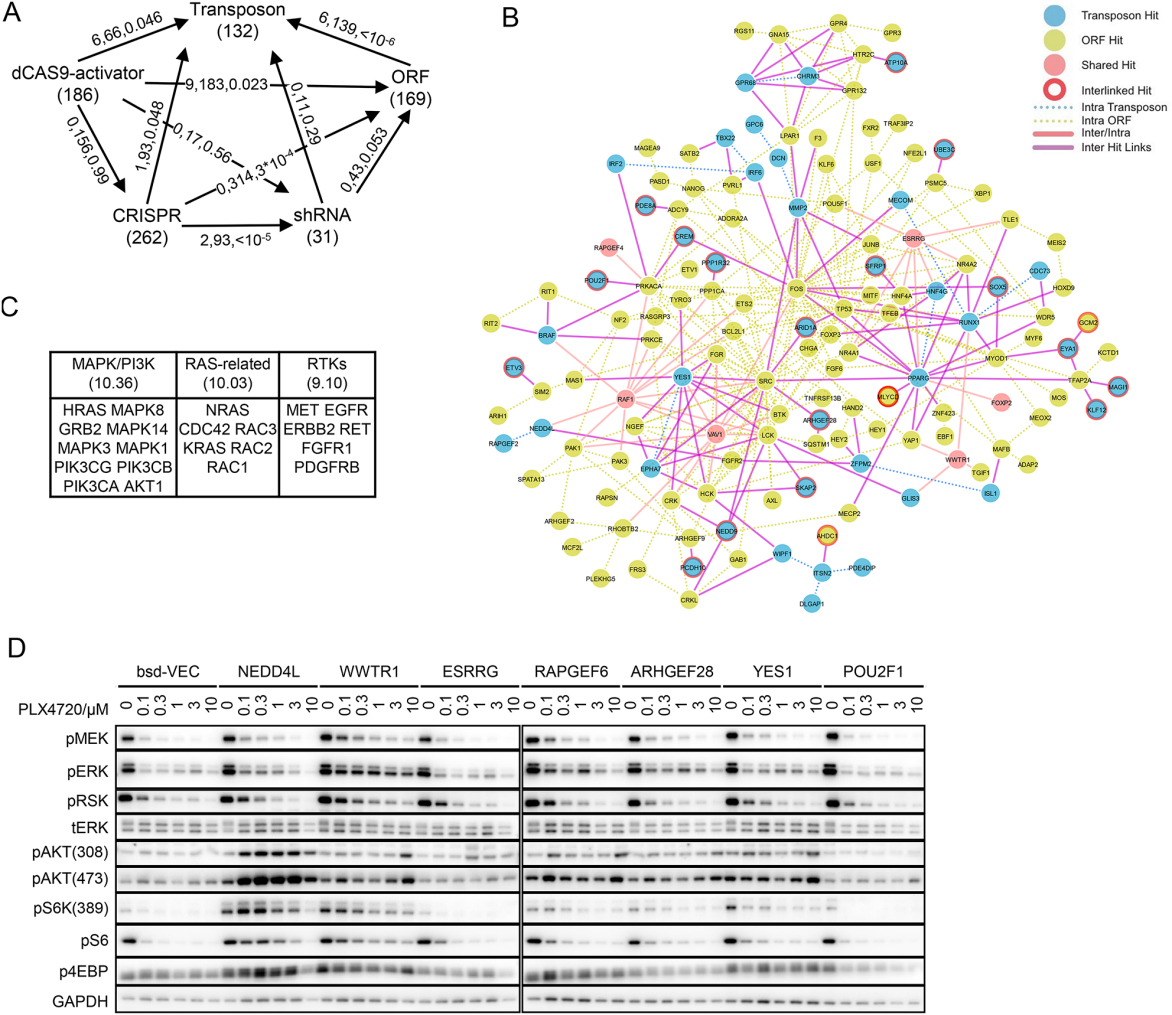
Interconnections between different screens. (**A**) Summary of STRING connections between different genetic screens. Each node represents a screen, with the gene set size indicated within parentheses. Edges represent STRING correlations with arrows pointing from the query gene set to the reference gene set. Numbers separated by commas denote numbers of overlapping genes, total STRING connections, and p values. (**B**) STRING network connections between the transposon and ORF gene sets. Nodes are color-coded according to their gene list of origin and edges. Nodes with red circles (interlinked hits) are those genes from a given list that only have connections to the other list. Edges are color-coded according to whether the connection is between genes from the same list or not. Edges connecting genes found in both screens to other genes (inter-and intra-list connections incident to solid pink nodes) are distinguished from those connecting nonoverlapping genes to the other list (only inter-list connections). (**C**) Top functional groups identified by DAVID Gene Functional Classification analysis of the core gene set highly connected to all screens. Enrichment scores are indicated within parentheses. (**D**) MAPK and PI3K signaling. Cells were treated with the indicated doses of PLX4720 for 24 h, and the levels of the indicated phosphorylated (pGENE) and total (tGENE) proteins were measured by western blotting.

To better understand whether the lack of overlap points to a higher-than-expected rate of false positive discovery or to low functional saturation and thus corresponding more to lack of screen sensitivity, we analyzed the functional relationship between the hit gene lists from the various GoF and LoF screens performed to uncover resistance events to BRAF inhibition in A375 cells. Genes identified by GoF ORF^15^ and dCAS9 activator^14^ screens and LoF CRISPR knockout^16^ and shRNA knockdown^17^ screens were mapped to a global functional cellular network using the Search Tool for the Retrieval of Interacting Genes/Proteins (STRING) (Fig. 3A**, Table S4**)^29^. Between the transposon and ORF sets, we found 139 STRING connections, which was significantly greater than random (*p* < 10^–6^) and more significant than other gene set pairs tested (**Fig. S3A**). The overall network constituted by genes from both screens was highly interconnected, and some of the genes identified in one screen were connected only to genes from the other screen but not to genes found within their own screen (circled in red in Fig. 3B). Similarly, 66 STRING database gene–gene connections were found between the transposon and dCAS9 activator hit gene lists (*p* = 0.046). The STRING connections between the LoF CRISPR and shRNA screens were also significant (*p* < 10^–5^), but neither matched the GoF screens except for the CRISPR-to-ORF pair, albeit presenting no actual gene overlap. We further sought to leverage the results of all the screens together to identify core programs contributing to resistance. We identified a core set of 133 genes by globally searching for all RefSeq genes with at least 10 connections to each of the screened gene sets (excluding the small gene set of the shRNA screen) (**Table S5**) with the top functional clusters of MAPK and PI3K pathways, RAS-related genes, and receptor tyrosine kinases (RTKs) (Fig. 3C). We further specifically analyzed the STRING connections between the screened gene sets and major MAPK pathway components and found significant functional connections from the GoF gene sets (p-values ranging from 0.01 to 10^–5^) (**Fig. S3B**).

Consistent with our STRING network analysis results and with other studies indicating that reactivation of MAPK activity is a major route for resistance in BRAF-driven models^2,27,30–33^, among the 7 genes validated through ORF overexpression, 5 (*NEDD4L*, *WWTR1*, *RAPGEF6*, *ARHGEF28* and *YES1*) led to maintenance of the MAPK signaling pathway upon BRAF inhibition (Fig. 3D). Overall, these results show that there is much better concordance at the functional network level across genes discovered by different screens than suggested by the lack of actual overlap of the gene lists. Moreover, many of the genes found to induce resistance appear to coalesce on a relatively narrow set of mechanisms, suggesting that a few core programs of resistance are repeatedly reached through different paths and could potentially be targeted to counter resistance.

### The Hippo pathway modulator TAZ as a global effector

**Fig. 4:**
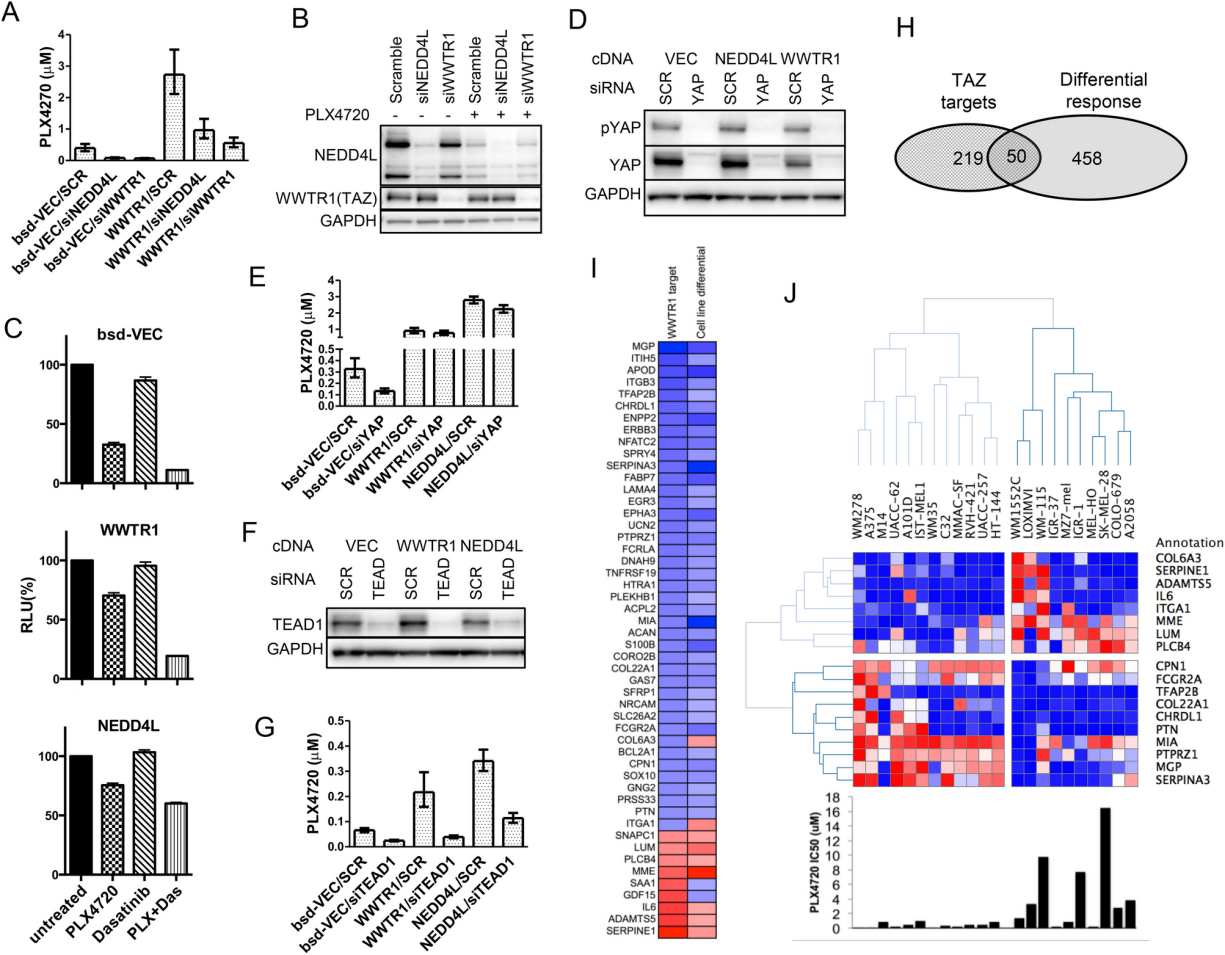
TAZ regulates gene expression. (**A**) PLX4720 IC50 values of control (bsd-VEC) or *WWTR1*-overexpressing cells following siRNA mediated knockdown of *NEDD4L* or *WWTR1*. SCR, scramble. Values are means ± 95% CIs, n = 3. (**B**) NEDD4L and TAZ protein expression in cells treated with siRNA for 48 h and then treated with 0.5 µM PLX4720 for 24 h. (**C**) Cell viability of the indicated cells treated with 300 nM PLX4720 (PLX) and 12.5 nM dasatinib (Das). Error bars denote the SEM, n = 3. (**D**) Effect of *YAP1* knockdown on protein expression and phosphorylation status in *NEDD4L* and *WWTR1* overexpressing cells. (**E**) PLX4720 IC50 values of control (bsd-VEC), *WWTR1*-, or *NEDD4L*-overexpressing cells with *YAP1* knockdown (siYAP) or scramble (SCR). Values are means ± 95% CIs, n = 5. (**F**) TEAD1 protein expression in *TEAD1* knockdown in control (VEC), *WWTR-*, and *NEDD4L-*overexpressing cells. (**G**) PLX4720 IC50 values of control (bsd-VEC), *WWTR1*-, or *NEDD4L*-overexpressing cells with *TEAD1* knockdown. Values are means ± 95% CIs, n = 3. (**H**) Intersect of TAZ-regulated targets and DEGs from PLX4720-sensitive and PLX4720-resistant melanoma cell lines. (**I**) Expression heatmap of the 50 overlapping genes. In the first column, the regulatory effects of TAZ are denoted either as repression (blue) or activation (red). The second column represents differential expression in two groups of melanoma cell lines, with blue indicating high expression in the sensitive group and red indicating high expression in the resistant group. (**J**) Unsupervised clustering of the expression values of 18 overlapping genes that most varied between the PLX4720-sensitive and PLX4720-resistant melanoma cell lines. Bars on the bottom panel indicate PLX4720 IC50 values.

The Hippo pathway effector TAZ as a transcriptional activator was by far the most prevalent hit for resistance gain among all genes tested in long-term drug treatment assays (Fig. 2C). Activation of YAP, the paralog of TAZ, has previously been shown to promote resistance to BRAF inhibition^34–36^. Hippo pathway inhibition (inhibition of MST1/2 kinases corresponding to Hippo in drosophila), leads to YAP/TAZ transcriptional program activation by nuclear translocation. Hippo pathway inactivation was previously shown to effectively resensitize cells to RTK inhibition^37^. Although the baseline level of TAZ protein did not predict the response to BRAF inhibitors in a panel of BRAF^V600E^ melanoma cell lines, ectopic overexpression increased resistance in several BRAF^V600E^ melanoma cell lines (**Fig. S4A, B**). Similarly, knocking down *WWTR1* induced further sensitization of A375 cells and antagonized their resistance to PLX4720 (Fig. 4A, B). This is also reminiscent of the sensitization of BRAF and other MAPK pathway activation tumor models to MAPK pathway inhibitors via the suppression of YAP1^34,35,38^. *WWTR1* was identified as a candidate resistance gene in the previous ORF screen along with *YAP1*^15^, and interestingly, as stated above, the SRC family tyrosine kinase YES1 validated in our screen was previously shown to regulate YAP1, which indeed was named after its association with YES1 (Yes Associated Protein 1)^39^. Consistent with these findings, we observed that the SRC family kinase inhibitor dasatinib strongly sensitized *WWTR1*-overexpressing cells to PLX4720 (Fig. 4C). We also observed that *YAP1* knockdown increased PLX4720 sensitivity in A375 parental cells but did not affect *WWTR1* overexpressing cells (Fig. 4D, E), consistent with the redundancy between the paralogs YAP1 and TAZ. Furthermore, knockdown of *TEADs* (*TEAD1-4*), the transcriptional partners that mediate the DNA binding of YAP1 and TAZ^40,41^, sensitized both parental and *WWTR1* overexpressing A375 cells to PLX4720 (Fig. 4F, G).

To elucidate the transcriptional changes induced by TAZ and their relevance across melanoma models, we divided 22 BRAF^V600^ mutant melanoma cell lines in our collection into two groups based on their sensitivity to PLX4720 and analyzed the differences in global gene expression between the two groups and identified 508 differentially expressed genes (DEGs) (**Table S6**). We also performed RNA-Seq on parental and *WWTR1* ORF-expressing A375 cells and found 315 DEGs with fourfold up- or downregulation (**Table S7**). Comparison of these two DEG lists revealed a significant overlap of 50 genes out of 269 TAZ target DEGs with measurements available in the cell line panel (chi-square = 284, *p* < 0.0001, Fig. 4H). This subset included several genes previously implicated in melanoma or tumor malignancy more broadly, such as *SOX10*, whose downregulation leads to elevated EGFR and PDGF receptor signals and increased resistance to BRAF inhibition^9^; *MIA*, which is involved in melanocyte lineage and melanoma development^42,43^; and *ERBB3*, which can reactivate MAPK upon MEK inhibition through a feedback mechanism^44,45^. Importantly, for almost all (46/50) genes, the DEG effect of the *WWTR1* ORF was predicted by the cell line PLX4720 sensitivity—39 genes downregulated by TAZ were highly expressed in the sensitive cell line group, while 7 upregulated genes were expressed in the resistant group (Fig. 4I). Furthermore, unsupervised clustering of the 18 most diversely expressed genes divided the melanoma lines according to their response to PLX4720 (Fig. 4J).

### TAZ and NEDD4L interconnect

The E3 ubiquitin-protein ligase *NEDD4L* was a prominent hit in our study with 32 nonidentical insertion events across 20 sites and 12.5% of all sequencing reads in the pools (Fig. 1D**, Table S1**). Interestingly, *NEDD4L* was also a hit in a previous BRAF inhibitor resistance study^20^. It was also implicated in EGFR-mTOR signaling in lung adenocarcinoma^46,47^ although in a somewhat functionally opposite way to our expectation. At least 8 NEDD4L protein isoforms have been reported (https://www.uniprot.org/uniprotkb/Q96PU5)^48,49^ and we noticed that two more isoforms at 230 kDa and 180 kDa might be present in A375 (**Fig. S4A**). To understand whether resistance to BRAF inhibition was limited to a specific isoform of NEDD4L, we initially investigated a prominent A375 cell isoform (PB clone B181 in **Fig. S1D, S5A**). However, cDNA-mediated (ORF) expression of a canonical NEDD4L isoform (110 kDa) was readily able to induce resistance in A375 and in other cell lines (**Fig. S4B**). Moreover, PLX4720 sensitivity across cell lines was not correlated with the expression of any specific isoform (**Fig. S4A**).

**Fig. 5:**
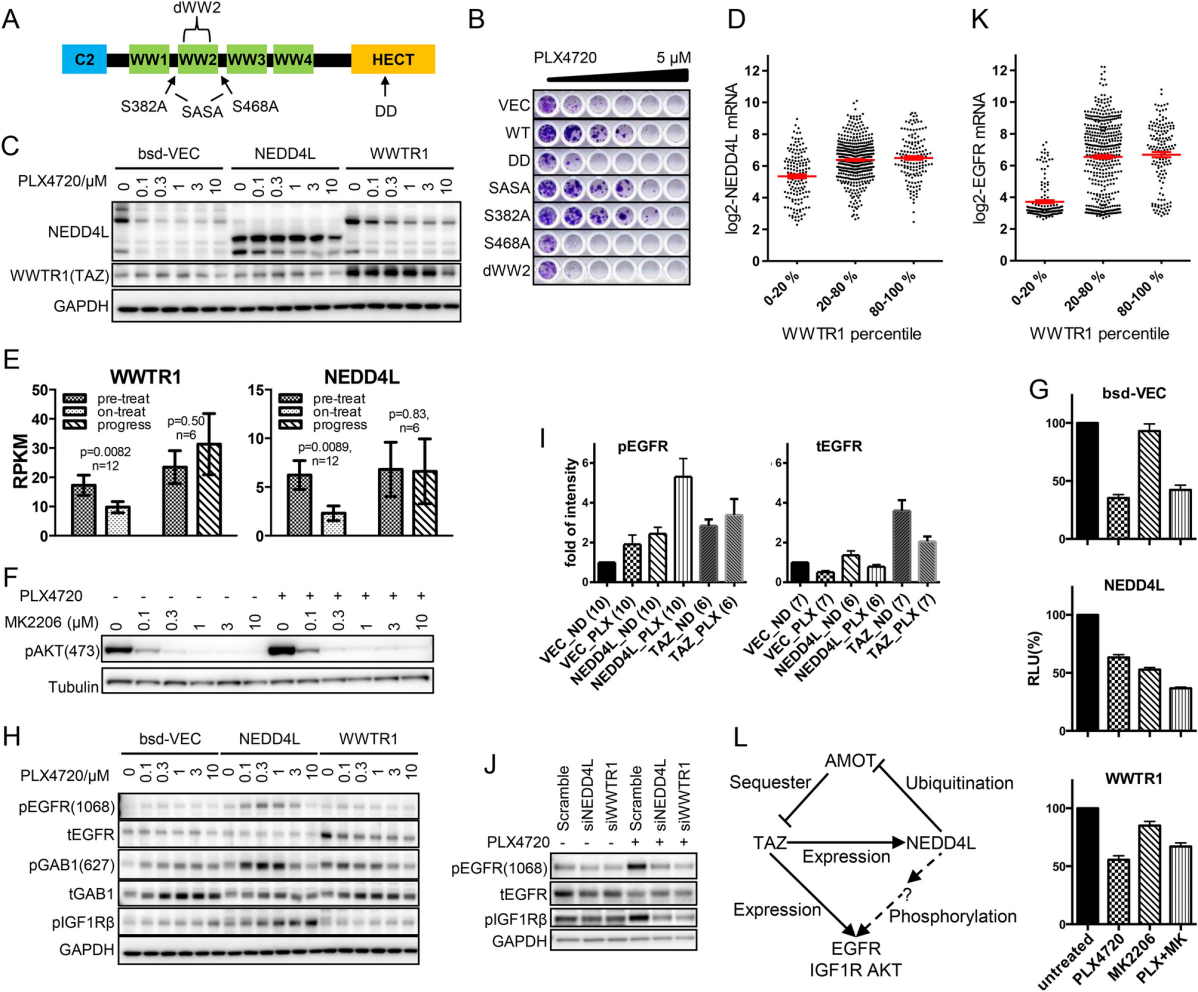
Functional connection between NEDD4L and TAZ. (**A**) Diagram of NEDD4L domains and mutations. (**B**) Clonogenic assay with cells expressing vector (VEC) or NEDD4L variants treated with PLX4720 at a twofold serial dilution. (**C**) Endogenous and exogenous NEDD4L and TAZ protein expression in control (bsd-VEC), *NEDD4L-,* and *WWTR1*-overexpressing cells after PLX4720 treatment for 24 h. (**D**) Correlation between *WWTR1* and *NEDD4L* mRNA expression across a panel of cancer cell lines of diverse origins. Each dot denotes a cell line. Red lines indicate mean ± SEM, n = 737. (**E**) *WWTR1* and *NEDD4L* mRNA expression in pre-treatment, on-treatment, and progression patient samples. Values represent means ± SEMs, and p-values were calculated using two-tailed paired t-test. (**F**) AKT phosphorylation in *NEDD4L-*overexpressing A375 cells treated with 0.5 µM PLX4720 and the indicated concentrations of MK2206 for 24 h. (**G**) Viabilities of the indicated cells treated with 300 nM PLX4720 and 1 µM MK2206 for 5 days. Values represent means ± SEMs, n = 10. Cellular activities were measured using CellTiter-Glo assays. (**H**) Kinase signals in vector control (bsd-VEC), *NEDD4L*-, or *WWTR1*-overexpressing cells. Cells were treated for 24 h and phosphorylated (pGENE) or total (tGENE) protein levels were measured by western blotting. (**I**) Quantification of phosphorylated EGFR (pEGFR) and total EGFR (tEGFR) protein expression from immunoblots of vector control (VEC), NEDD4L- (NEDD4L), and WWTR1 (TAZ)-overexpressing A375 cells either untreated (ND) or treated with PLX4720 (PLX). Parenthesized numbers indicate biological replicates. All values were normalized to the corresponding untreated vector control sample (VEC_ND) within each experiment. (**J**) Kinase signals in scramble control, *NEDD4L*, and *WWTR1* knockdown cells treated with PLX4720. Cells were transfected with siRNA for 48 h, followed by 0.5 µM PLX4720 treatment for 24 h. (**K**) Correlation between *WWTR1* and *EGFR* mRNA expression across a panel of cancer cell lines of diverse origins. Each dot denotes a cell line. Red lines indicate mean ± SEM, n = 737. (**L**) Hypothetical mechanism of TAZ and NEDD4L in mediating resistance to BRAF inhibition. Solid lines indicate known effects while the dashed line represents an indirect effect with a question mark denoting an unidentified component.

The NEDD4L protein contains an N-terminal C2 domain, four WW domains, and a HECT domain with E3 ubiquitin ligase catalytic activity (Fig. 5A)^28,50^. Previous studies have indicated that NEDD4L mediates the degradation of AMOT proteins, which are known regulators of the Hippo pathway^51–53^. According to these studies, AMOT proteins repress TAZ and YAP1 activity at least in part via cytoplasm retention of TAZ and YAP. We therefore studied the mechanistic underlying of NEDD4L mediated resistance by expressing a catalytically inactive C962A mutant of the HECT domain (DD) or a WW2-domain deletion mutant (dWW2), which was previously shown to be critical for the recognition of a number of NEDD4L substrates. In both cases, we found that resistance was indeed impaired compared to that obtained with wild-type NEDD4L (Fig. 5B**, S5A-C**).

NEDD4L has also been reported to regulate SMAD2/3, which are downstream effectors of the TGFβ pathway^50^. However, the projected effects of such possible role in BRAF-mutant melanoma are contradictory: NEDD4L-mediated SMAD2/3 degradation would lead to the termination of TGFβ signaling rather than its activation^28^, while previous reports have shown that TGFβ signaling upregulates the EGFR pathway to confer resistance to BRAF inhibition in melanoma^9^. Nonetheless, we tested this potential involvement by expressing missense mutations (S382A, S468A, or both (SASA)) targeting phosphorylation sites for SGK1, which is critical for SMAD2/3 regulation, and found that while protein expression of the S468A mutant failed despite decent mRNA expression (**Fig. S5A**), both S382A and SASA were comparable to those of the wild type in inducing resistance (Fig. 5B**, S5A–C**). Furthermore, knockdown of *SMAD2/3* modestly reduced rather than increasing the resistance mediated by both parental A375 and *NEDD4L* cells (**Fig. S5D, E**), and the SMAD2/3 protein level did not increase upon *NEDD4L* knockdown (**Fig. S5F**), suggesting the independent regulation of sensitivity to BRAF inhibition by NEDD4L and by TGFβ.

*WWTR1* expression led to moderate *NEDD4L* upregulation at the protein (Fig. 5C) and mRNA levels (1.5-fold, *p* = 3.19E−06, FDR = 3.03E−05; **Table S7**); conversely, *WWTR1* knockdown led to reduced NEDD4L protein expression (Fig. 4B). *NEDD4L* expression was also positively correlated with *WWTR1* expression in a panel of cancer cell lines (Fig. 5D**, Table S8**), and both were correlated with melanoma treatment outcome (Fig. 5E**,** with individual patient samples shown in **Fig. S6**). These findings suggested that NEDD4L might be involved in TAZ-mediated resistance.

Consistent with a role of NEDD4L in the regulation of other signaling pathways, *NEDD4L* overexpression led to a strong increase in AKT phosphorylation upon BRAF inhibition (Fig. 3D), and correspondingly, the AKT inhibitor MK2206 that strongly reduced AKT phosphorylation (Fig. 5F) effectively resensitized *NEDD4L*-overexpressing A375 cells to PLX4720 (Fig. 5G). NEDD4L also led to the phosphorylation of EGFR (at residue Y1068) and another RTK, IGF1R, while TAZ upregulated EGFR protein expression (Fig. 5H, I). Supporting these observations, knockdown of *NEDD4L* reduced EGFR and IGF1R phosphorylation and knockdown of *WWTR1* reduced EGFR expression (Fig. 5J). The regulation of *EGFR* expression by TAZ is also in line with the positive correlation between *EGFR* and *WWTR1* expression in a cell line panel (Fig. 5K**, Table S8**) and the elevated *EGFR* mRNA expression in *WWTR1* ORF-overexpressing cells (2.0-fold, *p* = 3.31E−12, FDR = 8.08E−11; **Table S7**). Therefore, TAZ plays an important role in the maintenance of MAPK pathway activation both through transcriptional regulation of some pathway component genes and likely through other signaling mechanisms (Fig. 5L). In line with this, both the EGFR inhibitor pelitinib (EKB569) and the IGF1R inhibitor BMS754807 countered resistance induced by *NEDD4L*, *WWTR1*, and all other resistance genes validated in this study (**Fig. S7**), suggesting that RTK inhibitors might resensitize cells to MAPK pathway inhibition more broadly than previously described^2,6,54^.

## Discussion

As an alternative to genetic screening platforms consisting of complex mutagen libraries (such as ORFs), transposon mutagenesis has the potential to tackle some of the complexity of host genome harboring both known protein coding genes and unidentified components, non-coding elements, or alternative isoforms. Moreover, activation of endogenous elements might provide more context specific results than ectopically expressed transcription activators. While transposon mutagenesis has been widely used in vivo^19,55^, its application as an in vitro cell line screening platform results in expandable off-the-shelf libraries that are ideal for applications involving complex treatment conditions, such as comparing resistance to multiple drugs. The low cost and simplicity of this approach, requiring only transient transfection of two plasmids, enables straightforward high-throughput platform deployment^20^. While not utilized in this study, additional features, such as tagging transposition plasmids with degenerate nucleotide barcodes, should further improve platform scalability. Such barcode diversity can then be sequenced to reveal nonidentical insertions even at the same genomic location, essentially eliminating the reliance on library replicates for identification of high confidence hits.

Our study provided further support for therapies targeting the Hippo pathway^37^ or protein ubiquitination^56,57^, and we also demonstrated that many different genes with a broad range of functions implicated in resistance to target inhibition therapies appear to coalesce to the pathway of primary target inhibition (here the MAPK pathway) and a restricted number of additional pathways, suggesting that the staggering complexity of the resistance landscape may not need to be addressed by an equivalently complex array of therapeutic strategies.

## Methods

### Cell culture reagents

All cell lines were obtained from the collection of the Genomics of Drug Sensitivity in Cancer (GDSC) project^13^, cultured in either DMEM/F12 or RPMI supplemented with 5% FBS and 1% penicillin/streptavidin, and maintained in a 37 °C/5% CO_2_ cell culture incubator. Therapeutic compounds were purchased from Selleckchem and ChemieTek and dissolved in dimethyl sulfoxide.

### Transposon mutagenesis library construction and screening

The *piggyBac* activation transposition plasmid pPB-SB-CMV-puro-SD was described previously^21^. A derivative plasmid containing the degenerate nucleotide barcodes mentioned in the Discussion section was verified by sequencing and the plasmid map and complete sequence are provided in the supplementary information (**Fig. S8, Supplementary Methods and Text**). Cell line libraries were constructed by transfecting A375 cells with the transposition plasmid and the transposase plasmid pCMV-hyPBase^58^. After puromycin selection, cells were treated with PLX4720, and surviving cells were pooled or isolated. Insertion site sequencing libraries were prepared using ligation-mediated PCR and identified with next-generation sequencing (NGS) as described previously^21^.

### Gene annotation for insert sites

Illumina sequencing data in FASTQ files were demultiplexed and trimmed to retain only the genomic DNA sequences. Reads of 7 bp or longer were aligned to the human reference genome (hg19) using Bowtie and unique reads were recorded. For the resistant pools, the top sequences by read count were reported based on our estimate of the survivor colony numbers from each library, with the top 100 inserts representing more than 99.5% of the total sequencing reads. For each insert, the three most proximal genes were annotated.

### Melanoma patient cohort

The patient cohort with metastatic melanoma containing BRAF^V600E^ mutation enrolled on clinical trials for treatment with a BRAF inhibitor vemurafenib or combined BRAF + MEK inhibitor (dabrafenib + trametinib or LGX818 + MEK162) was reported previously^59^. Patients provided written informed consent for the collection of tissue and blood samples for research and genomic profiling, as approved by the Dana-Farber/Harvard Cancer Center Institutional Review Board (DF/HCC Protocol 11–181).

### Lentiviral cDNA plasmid construction and cell assays

ORF entry clones were subcloned and inserted into two sets of lentiviral expression vectors (blasticidin and puromycin) for cell-based assays. Cells viability, apoptosis, and proliferation were assayed. All assays were performed at least three times.

### Immunoblotting

Immunoblotting was described in the Supplementary Methods. Signals were detected using SuperSignal West Femto substrate (Thermo Fisher) and imaged with a G-box imager (Syngene). Original image files were provided in the Supplementary Information. To measure relative intensities, immunoblot images were quantified using ImageJ (https://imagej.net/ij/) and signals were normalized to control gene bands from the same gel.

### RNA-Seq

Total RNA was prepared using RNeasy Mini Kit (Qiagen) and processed using a TruSeq RNA Sample Preparation Kit (Illumina) to generate libraries of sequencing molecules. For samples derived from transposon-inserted clones, equal amounts of 12 indexed subsamples were pooled and sequenced using an Illumina sequencer instrument. STAR aligner^60^ was used to map sequencing reads to transcripts. Read counts for individual transcripts were produced with HTSeq-count^61^, followed by the estimation of expression values as RPKM (reads per kilobase per million) and the detection of differentially expressed transcripts using EdgeR^62^. For *WWTR1*-overexpressing cDNA clones, samples were prepared from four biological replicates of viral infections, indexed, pooled, and sequenced. The Benjamini–Hochberg false discovery rate (FDR) was used to estimate the statistical significance of differences in gene expression.

### STRING analysis

Known and predicted protein–protein associations were tested using STRING analysis (http://string-db.org, Homo sapiens: 9606.protein.links.detailed.v9.1.txt.gz). STRING analyses were first performed between pairs of screened gene sets (**Table S4**). To identify a core set of genes responsible for resistance, all RefSeq genes were queried for their connections to components of every screened gene set (**Table S5**). The list of genes with at least 10 connections to each of four screens (excluding the small shRNA screen gene set) was used as input for the Database for Annotation, Visualization and Integrated Discovery (DAVID) analyses^63^.

### Clustering of gene expression values

The genes most differentially expressed between BRAF inhibitor-sensitive and -resistant cell lines were selected for unsupervised clustering using Pearson’s correlation across genes and cell lines. Clustering and heatmap generation were performed using Gene-E (http://www.broadinstitute.org/cancer/software/GENE-E/index.html).

Additional protocol details can be found in the **Supplementary Methods and Text**.

## Supplementary Information

Supplementary Information.

## Data Availability

Melanoma patient RNA-Seq data were deposited in the NCBI GEO under accession number GSE73470. This RNA-seq dataset can also be accessed from European Genome-phenome Archive (EGA Study ID EGAS00001000992). Cell line data are available through the Genomics of Drug Sensitivity in Cancer (https://www.cancerrxgene.org/) or DepMap (https://depmap.org/portal/) portals.

## References

[1] Bollag, G. et al. Clinical efficacy of a RAF inhibitor needs broad target blockade in BRAF-mutant melanoma. *Nature* **467**(7315), 596–599 (2010). 20823850 10.1038/nature09454 PMC2948082

[2] Corcoran, R. B. et al. EGFR-mediated re-activation of MAPK signaling contributes to insensitivity of BRAF mutant colorectal cancers to RAF inhibition with vemurafenib. *Cancer Discov* **2**(3), 227–235 (2012). 22448344 10.1158/2159-8290.CD-11-0341 PMC3308191

[3] Corcoran, R. B., et al. BRAF gene amplification can promote acquired resistance to MEK inhibitors in cancer cells harboring the BRAF V600E mutation*.**Sci. Signal*, 2010. **3**(149): p. ra84. 10.1126/scisignal.2001148 PMC3372405 21098728

[4] Flaherty, K. T. et al. Inhibition of mutated, activated BRAF in metastatic melanoma. *N Engl J Med* **363**(9), 809–819 (2010). 20818844 10.1056/NEJMoa1002011 PMC3724529

[5] Montagut, C. et al. Elevated CRAF as a potential mechanism of acquired resistance to BRAF inhibition in melanoma. *Cancer Res* **68**(12), 4853–4861 (2008). 18559533 10.1158/0008-5472.CAN-07-6787 PMC2692356

[6] Prahallad, A. et al. Unresponsiveness of colon cancer to BRAF (V600E) inhibition through feedback activation of EGFR. *Nature* **483**(7387), 100–103 (2012). 22281684 10.1038/nature10868

[7] Sanchez-Laorden, B., et al. BRAF inhibitors induce metastasis in RAS mutant or inhibitor-resistant melanoma cells by reactivating MEK and ERK signaling*.**Sci. Signal* 2014. **7**(318): ra30. 10.1126/scisignal.2004815 24667377

[8] Shi, H. et al. Acquired resistance and clonal evolution in melanoma during BRAF inhibitor therapy. *Cancer Discov* **4**(1), 80–93 (2014). 24265155 10.1158/2159-8290.CD-13-0642 PMC3936420

[9] Sun, C. et al. Reversible and adaptive resistance to BRAF(V600E) inhibition in melanoma. *Nature* **508**(7494), 118–122 (2014). 24670642 10.1038/nature13121

[10] Barretina, J. et al. The cancer cell line encyclopedia enables predictive modelling of anticancer drug sensitivity. *Nature* **483**(7391), 603–607 (2012). 22460905 10.1038/nature11003 PMC3320027

[11] Lawrence, M. S. et al. Mutational heterogeneity in cancer and the search for new cancer-associated genes. *Nature* **499**(7457), 214–218 (2013). 23770567 10.1038/nature12213 PMC3919509

[12] Solit, D. B. et al. BRAF mutation predicts sensitivity to MEK inhibition. *Nature* **439**(7074), 358–362 (2006). 16273091 10.1038/nature04304 PMC3306236

[13] Garnett, M. J. et al. Systematic identification of genomic markers of drug sensitivity in cancer cells. *Nature* **483**(7391), 570–575 (2012). 22460902 10.1038/nature11005 PMC3349233

[14] Konermann, S. et al. Genome-scale transcriptional activation by an engineered CRISPR-Cas9 complex. *Nature* **517**(7536), 583–588 (2015). 25494202 10.1038/nature14136 PMC4420636

[15] Johannessen, C. M. et al. A melanocyte lineage program confers resistance to MAP kinase pathway inhibition. *Nature* **504**(7478), 138–142 (2013). 24185007 10.1038/nature12688 PMC4098832

[16] Shalem, O. et al. Genome-scale CRISPR-Cas9 knockout screening in human cells. *Science* **343**(6166), 84–87 (2014). 24336571 10.1126/science.1247005 PMC4089965

[17] Whittaker, S. R. et al. A genome-scale RNA interference screen implicates NF1 loss in resistance to RAF inhibition. *Cancer Discov* **3**(3), 350–362 (2013). 23288408 10.1158/2159-8290.CD-12-0470 PMC3606893

[18] Choi, J. et al. Identification of PLX4032-resistance mechanisms and implications for novel RAF inhibitors. *Pigment Cell Melanoma Res* **27**(2), 253–262 (2014). 24283590 10.1111/pcmr.12197 PMC4065135

[19] Mann, M. B. et al. Transposon mutagenesis identifies genetic drivers of Braf(V600E) melanoma. *Nat Genet* **47**(5), 486–495 (2015). 25848750 10.1038/ng.3275 PMC4844184

[20] Zhu, E. Y. et al. Understanding cancer drug resistance with Sleeping Beauty functional genomic screens: Application to MAPK inhibition in cutaneous melanoma. *iScience* **26**(10), 107805 (2023). 37860756 10.1016/j.isci.2023.107805 PMC10582486

[21] Chen, L. et al. Transposon activation mutagenesis as a screening tool for identifying resistance to cancer therapeutics. *BMC Cancer* **13**(1), 93 (2013). 23442791 10.1186/1471-2407-13-93 PMC3598783

[22] Bruchez, A., et al. MHC class II transactivator CIITA induces cell resistance to Ebola virus and SARS-like coronaviruses. *Science*, 2020. 10.1126/science.abb3753 PMC7665841 32855215

[23] Anvar, S. Y. et al. Full-length mRNA sequencing uncovers a widespread coupling between transcription initiation and mRNA processing. *Genome Biol* **19**(1), 46 (2018). 29598823 10.1186/s13059-018-1418-0 PMC5877393

[24] Kornblihtt, A. R. et al. Multiple links between transcription and splicing. *RNA* **10**(10), 1489–1498 (2004). 15383674 10.1261/rna.7100104 PMC1370635

[25] Davoli, T. et al. Cumulative haploinsufficiency and triplosensitivity drive aneuploidy patterns and shape the cancer genome. *Cell* **155**(4), 948–962 (2013). 24183448 10.1016/j.cell.2013.10.011 PMC3891052

[26] Ruiz-Saenz, A. et al. A reversible SRC-relayed COX2 inflammatory program drives resistance to BRAF and EGFR inhibition in BRAF(V600E) colorectal tumors. *Nat Cancer* **4**(2), 240–256 (2023). 36759733 10.1038/s43018-022-00508-5 PMC9970872

[27] Johannessen, C. M. et al. COT drives resistance to RAF inhibition through MAP kinase pathway reactivation. *Nature* **468**(7326), 968–972 (2010). 21107320 10.1038/nature09627 PMC3058384

[28] Gao, S. et al. Ubiquitin ligase Nedd4L targets activated Smad2/3 to limit TGF-beta signaling. *Mol Cell* **36**(3), 457–468 (2009). 19917253 10.1016/j.molcel.2009.09.043 PMC2796330

[29] Jensen, L. J., et al. STRING 8: A global view on proteins and their functional interactions in 630 organisms*.**Nucleic Acids Res*., 2009. **37**(Database issue): D412–6. 10.1093/nar/gkn760 PMC2686466 18940858

[30] Goetz, E. M. et al. ERK mutations confer resistance to mitogen-activated protein kinase pathway inhibitors. *Cancer Res* **74**(23), 7079–7089 (2014). 25320010 10.1158/0008-5472.CAN-14-2073 PMC4300142

[31] Long, G. V. et al. Increased MAPK reactivation in early resistance to dabrafenib/trametinib combination therapy of BRAF-mutant metastatic melanoma. *Nat Commun* **5**, 5694 (2014). 25452114 10.1038/ncomms6694

[32] Van Allen, E. M. et al. The genetic landscape of clinical resistance to RAF inhibition in metastatic melanoma. *Cancer Discov* **4**(1), 94–109 (2014). 24265153 10.1158/2159-8290.CD-13-0617 PMC3947264

[33] Yadav, V. et al. Reactivation of mitogen-activated protein kinase (MAPK) pathway by FGF receptor 3 (FGFR3)/Ras mediates resistance to vemurafenib in human B-RAF V600E mutant melanoma. *J Biol Chem* **287**(33), 28087–28098 (2012). 22730329 10.1074/jbc.M112.377218 PMC3431627

[34] Lin, L. et al. The Hippo effector YAP promotes resistance to RAF- and MEK-targeted cancer therapies. *Nat Genet* **47**(3), 250–256 (2015). 25665005 10.1038/ng.3218 PMC4930244

[35] Kapoor, A. et al. Yap1 activation enables bypass of oncogenic Kras addiction in pancreatic cancer. *Cell* **158**(1), 185–197 (2014). 24954535 10.1016/j.cell.2014.06.003 PMC4109295

[36] Shao, D. D. et al. KRAS and YAP1 converge to regulate EMT and tumor survival. *Cell* **158**(1), 171–184 (2014). 24954536 10.1016/j.cell.2014.06.004 PMC4110062

[37] Chapeau, E. A., et al. Direct and selective pharmacological disruption of the YAP-TEAD interface by IAG933 inhibits Hippo-dependent and RAS-MAPK-altered cancers*.**Nat. Cancer*, 2024. 10.1038/s43018-024-00754-9 PMC11286534 38565920

[38] Flaherty, K. T., Wargo, J. A. & Bivona, T. G. YAP in MAPK pathway targeted therapy resistance. *Cell Cycle* **14**(12), 1765–1766 (2015). 26036142 10.1080/15384101.2015.1032644 PMC4612653

[39] Sudol, M. et al. Characterization of the mammalian YAP (Yes-associated protein) gene and its role in defining a novel protein module, the WW domain. *J Biol Chem* **270**(24), 14733–14741 (1995). 7782338 10.1074/jbc.270.24.14733

[40] Zanconato, F., et al. Genome-wide association between YAP/TAZ/TEAD and AP-1 at enhancers drives oncogenic growth. *Nat. Cell Biol.* (2015). 10.1038/ncb3216 PMC6186417 26258633

[41] Zhao, B. et al. TEAD mediates YAP-dependent gene induction and growth control. *Genes Dev* **22**(14), 1962–1971 (2008). 18579750 10.1101/gad.1664408 PMC2492741

[42] Blesch, A. et al. Cloning of a novel malignant melanoma-derived growth-regulatory protein, MIA. *Cancer Res* **54**(21), 5695–5701 (1994). 7923218

[43] Poser, I. et al. Functional role of MIA in melanocytes and early development of melanoma. *Oncogene* **23**(36), 6115–6124 (2004). 15208686 10.1038/sj.onc.1207797

[44] Carraway, K. L., 3rd and Cantley, L. C. A neu acquaintance for erbB3 and erbB4: A role for receptor heterodimerization in growth signaling. *Cell*. **78**(1): p. 5–8 (1994). 10.1016/0092-8674(94)90564-9 8033211

[45] Turke, A. B. et al. MEK inhibition leads to PI3K/AKT activation by relieving a negative feedback on ERBB receptors. *Cancer Res* **72**(13), 3228–3237 (2012). 22552284 10.1158/0008-5472.CAN-11-3747 PMC3515079

[46] Ding, K., et al. JAC4 inhibits EGFR-driven lung adenocarcinoma growth and metastasis through CTBP1-mediated JWA/AMPK/NEDD4L/EGFR axis. *Int. J. Mol. Sci.* **24**(10) (2023). 10.3390/ijms24108794 PMC10218353 37240137

[47] Li, G. et al. Downregulation of NEDD4L by EGFR signaling promotes the development of lung adenocarcinoma. *J Transl Med* **20**(1), 47 (2022). 35090513 10.1186/s12967-022-03247-4 PMC8800232

[48] Dunn, D. M. et al. Common variant of human NEDD4L activates a cryptic splice site to form a frameshifted transcript. *J Hum Genet* **47**(12), 665–676 (2002). 12522688 10.1007/s100380200102

[49] Chen, H. et al. NEDD4L on human chromosome 18q21 has multiple forms of transcripts and is a homologue of the mouse Nedd4-2 gene. *Eur J Hum Genet* **9**(12), 922–930 (2001). 11840194 10.1038/sj.ejhg.5200747

[50] Debonneville, C. et al. Phosphorylation of Nedd4-2 by Sgk1 regulates epithelial Na(+) channel cell surface expression. *EMBO J* **20**(24), 7052–7059 (2001). 11742982 10.1093/emboj/20.24.7052 PMC125341

[51] Rheinemann, L. et al. Interactions between AMOT PPxY motifs and NEDD4L WW domains function in HIV-1 release. *J Biol Chem* **297**(2), 100975 (2021). 34284061 10.1016/j.jbc.2021.100975 PMC8368996

[52] Wang, C. et al. The Nedd4-like ubiquitin E3 ligases target angiomotin/p130 to ubiquitin-dependent degradation. *Biochem J* **444**(2), 279–289 (2012). 22385262 10.1042/BJ20111983

[53] Zhao, B. et al. Angiomotin is a novel Hippo pathway component that inhibits YAP oncoprotein. *Genes Dev* **25**(1), 51–63 (2011). 21205866 10.1101/gad.2000111 PMC3012936

[54] Villanueva, J. et al. Acquired resistance to BRAF inhibitors mediated by a RAF kinase switch in melanoma can be overcome by cotargeting MEK and IGF-1R/PI3K. *Cancer Cell* **18**(6), 683–695 (2010). 21156289 10.1016/j.ccr.2010.11.023 PMC3026446

[55] Ni, T. K. et al. Low-copy piggyBac transposon mutagenesis in mice identifies genes driving melanoma. *Proc Natl Acad Sci USA* **110**(38), E3640–E3649 (2013). 24003131 10.1073/pnas.1314435110 PMC3780872

[56] Alrosan, A. Z. et al. Potential roles of NEDD4 and NEDD4L and their utility as therapeutic targets in high-incidence adult male cancers (Review). *Mol Clin Oncol* **19**(3), 68 (2023). 37614371 10.3892/mco.2023.2664 PMC10442760

[57] Li, B., Adam Eichhorn, P. J. & Chng, W. J. Targeting the ubiquitin pathway in lymphoid malignancies. *Cancer Lett.* **594**, 216978 (2024). 38795760 10.1016/j.canlet.2024.216978

[58] Yusa, K. et al. A hyperactive piggyBac transposase for mammalian applications. *Proc Natl Acad Sci USA* **108**(4), 1531–1536 (2011). 21205896 10.1073/pnas.1008322108 PMC3029773

[59] Kwong, L. N. et al. Co-clinical assessment identifies patterns of BRAF inhibitor resistance in melanoma. *J Clin Invest* **125**(4), 1459–1470 (2015). 25705882 10.1172/JCI78954 PMC4396463

[60] Dobin, A. et al. STAR: Ultrafast universal RNA-seq aligner. *Bioinformatics* **29**(1), 15–21 (2013). 23104886 10.1093/bioinformatics/bts635 PMC3530905

[61] Anders, S., Pyl, P. T. & Huber, W. HTSeq: A Python framework to work with high-throughput sequencing data. *Bioinformatics* **31**(2), 166–169 (2015). 25260700 10.1093/bioinformatics/btu638 PMC4287950

[62] McCarthy, D. J., Chen, Y. & Smyth, G. K. Differential expression analysis of multifactor RNA-Seq experiments with respect to biological variation. *Nucleic Acids Res* **40**(10), 4288–4297 (2012). 22287627 10.1093/nar/gks042 PMC3378882

[63] Huang da, W., Sherman, B. T., and Lempicki, R. A. Systematic and integrative analysis of large gene lists using DAVID bioinformatics resources. *Nat. Protoc.* **4**(1): 44–57 (2009). 10.1038/nprot.2008.211 19131956

