# Supplementary material for: Transposon mediated functional genomic screening for BRAF inhibitor resistance reveals convergent Hippo and MAPK pathway activation events
Source: Sci Rep. 2025 Jan 24;15:3048. doi: 10.1038/s41598-025-86694-5 (PMC11760944; doi:10.1038/s41598-025-86694-5)
Supplement: Supplementary file 1 — Supplementary Information. [file 41598_2025_86694_MOESM1_ESM.pdf]

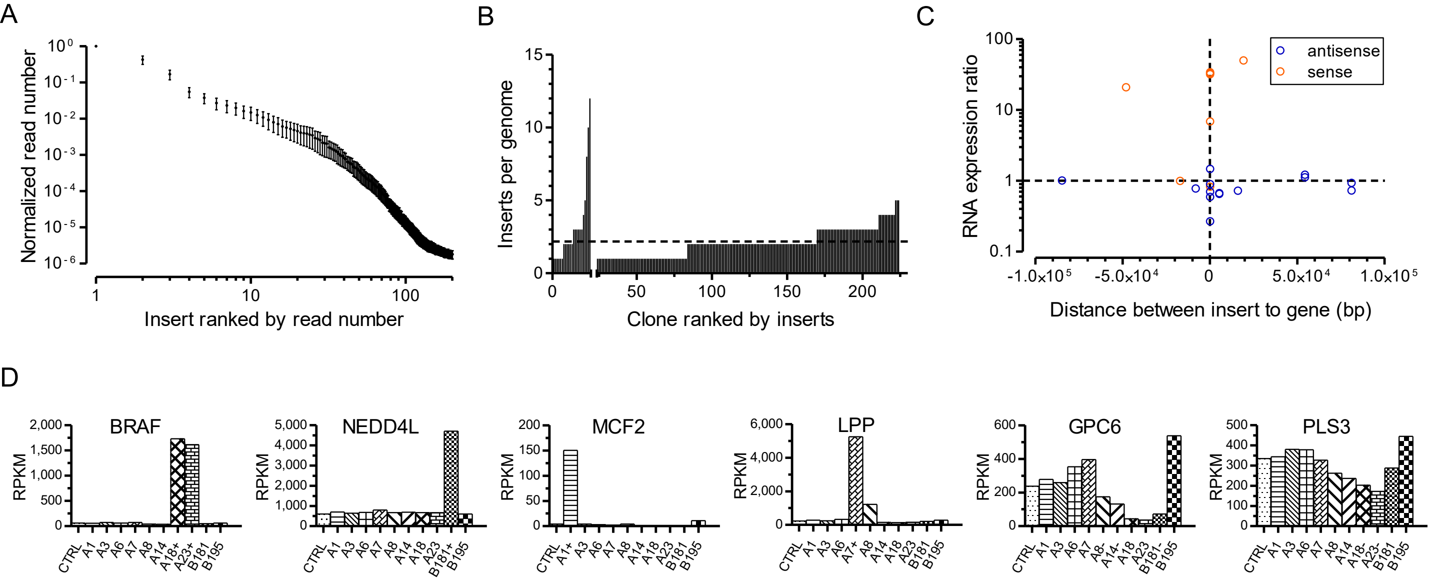


**Figure S1. Characterization of transposon inserts.**

(A) Distribution of sequencing read signal associated with top 100 insertion sites across ten resistant pools. x-axis, inserts ranked in each pool by read number; y-axis, read number normalized to the most abundant reads of each pool. Error bars denote standard error of mean.

(B) Number of inserts per clone. The dash line indicates the mean. Primary and limiting-dilution clones are separated by a gap in the x-axis.

(C) Gene expression relative to insertion position. Expression is affected by distance (x axis) and direction (sense or antisense) of PB inserts to the gene body. Negative distance values indicate inserts are upstream of the gene while positive values for downstream, and zero for intragenic inserts.

(D) Gene expression detected by RNA-seq in ten clones and a control sample (CTRL). Clones with the corresponding gene perturbed by transposon are labeled with a “+” for activation or “-” for disruption. RPKM, reads per kilobase million.

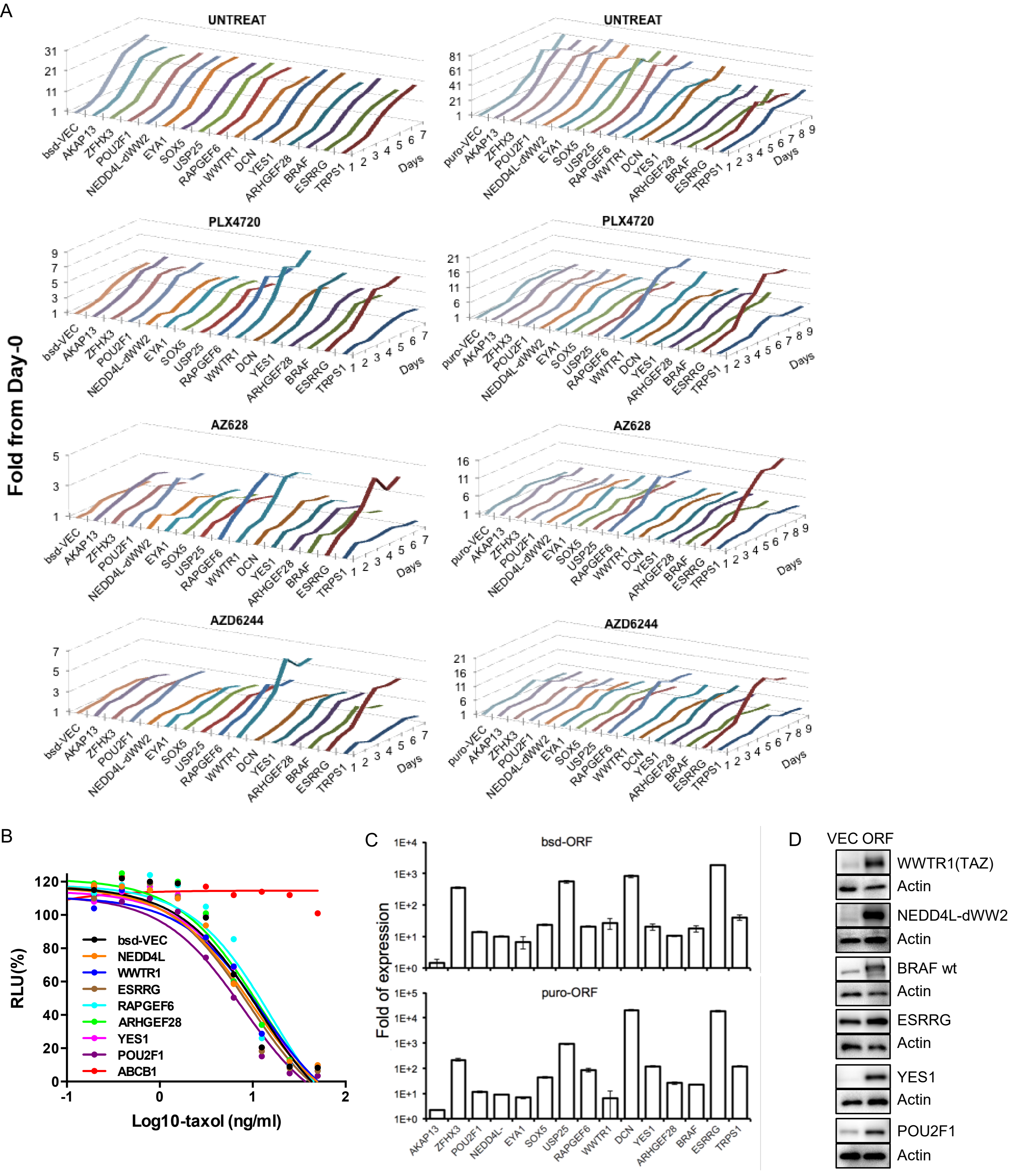


**Figure S2, Effect of candidate resistance genes on proliferation evaluated by cDNA overexpression.**
(A) Proliferation of A375 cells infected with lentiviral cDNA constructs across 7 or 9 days. Cells were either untreated, or treated with PLX4720, AZ628, or AZD6244 and normalized to Day-0. Each cDNA construct was subcloned into two lentivirus vectors (bsd-ORF and puro-ORF) for independent experiments.
(B) Dose responses of cells to paclitaxel (taxol). The taxol resistance gene ABCB1 was used as a control.
(C) Quantitative PCR for the indicated genes normalized to vector control cells. Error bars denote SEM, n=3. NEDD4L-dWW2 or NEDD4L- represents a WW2-domain-deletion isoform.
(D) Western blots of blasticidin vector (VEC) or ORF overexpression (ORF) proteins.

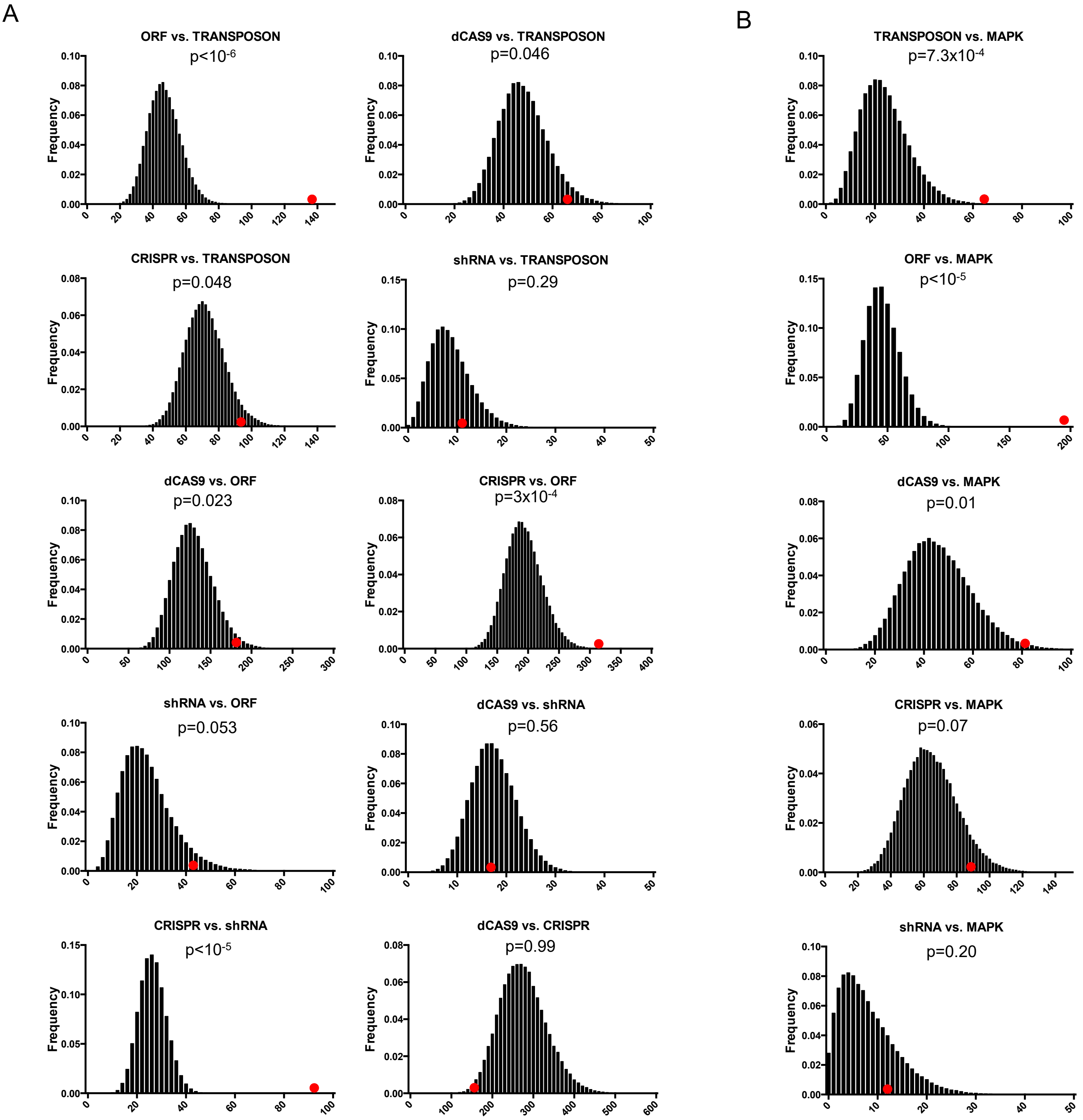


**Figure Statistical test for gene list connectivity based on STRING across multiple screens.**
(A) Distributions of STRING connections between genetic screens using genes across 10^6 (for ORF vs. TRANSPOSON) or 10^5 (for others) random picks from corresponding backgrounds. Red dots indicate the observed number of connections between two genesets.
(B) Distributions of STRING connections between transposon and MAPK components.

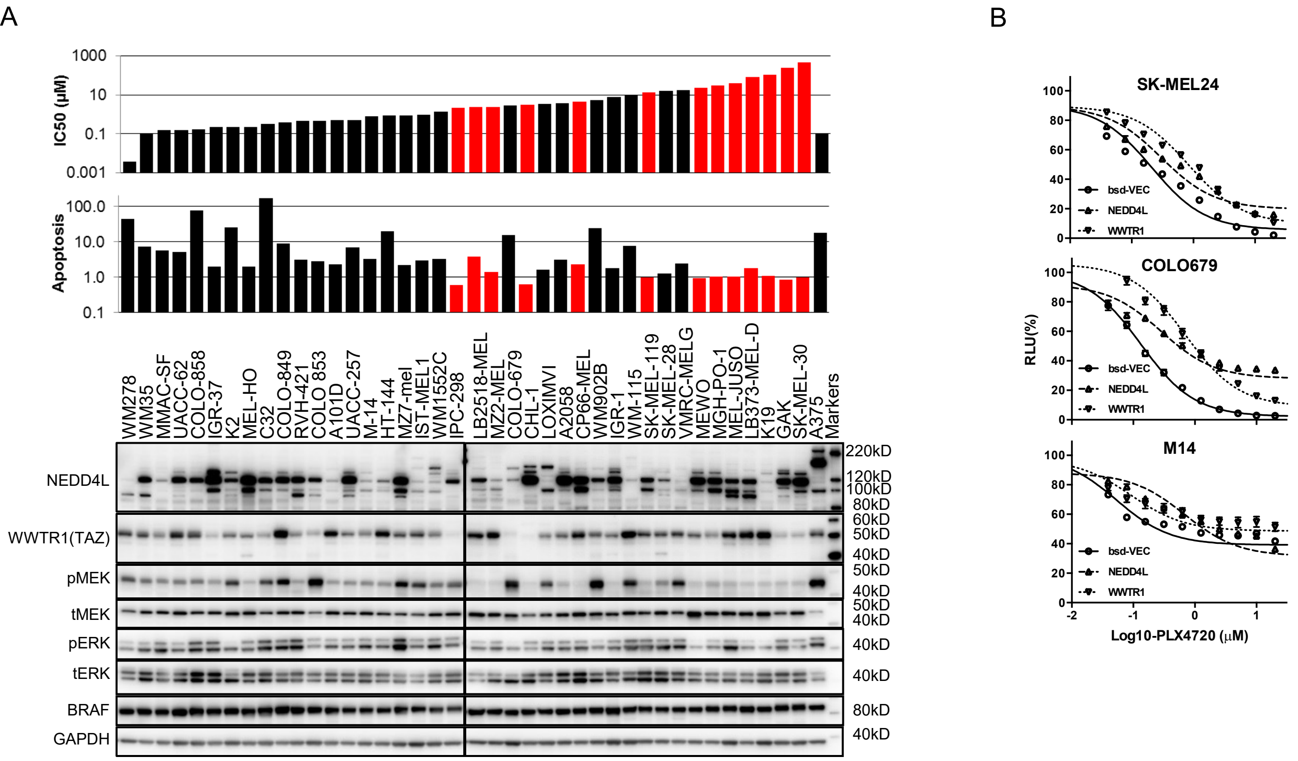


**Figure S4. TAZ and NEDD4L protein expression versus sensitivity in melanoma cell lines.**

(A) NEDD4L and TAZ (WWTR1) expression and MAPK signaling across melanoma cell lines. Expression of the indicated proteins by western blot performed across lysates of melanoma cells lines. Lysates were loaded from left to right according to sensitivity to PLX4720 in two gels processed in the same experiment. The corresponding IC50s measured by CellTiter-Glo and apoptosis induction determined using Caspase-Glo normalized to total cell number are shown in the top and middle histograms. BRAF V600E or V600D mutants are denoted as black columns while BRAF wildtypes are in red.

(B) PLX4720 dose response of different melanoma cell lines with NEDD4L or WWTR1 lentiviral overexpression. Error bars denote SEM, n=4.

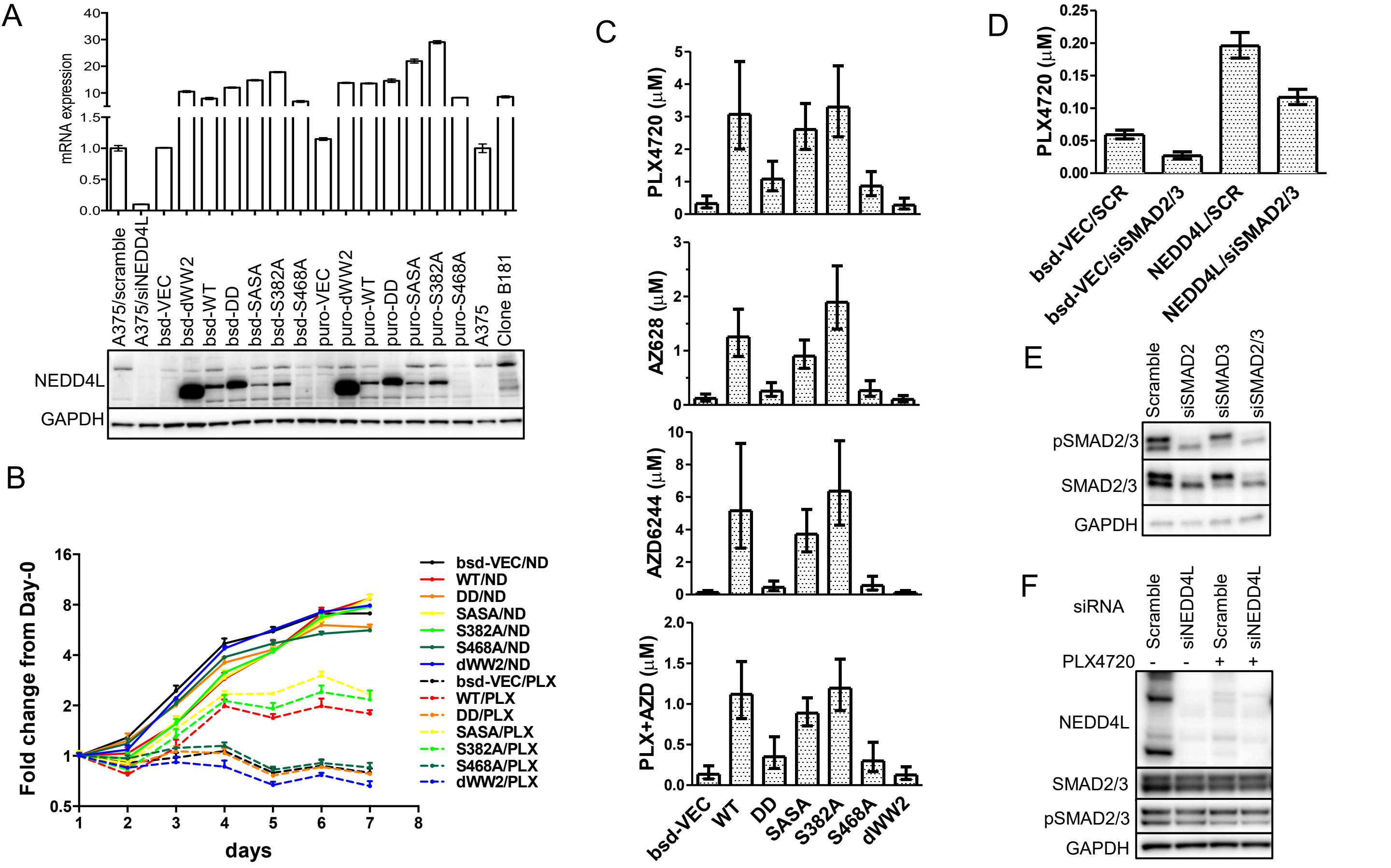


**Figure S5. NEDD4L mutation activities.**
(A) NEDD4L mutants expressed in two sets of vectors (bsd- and puro-). mRNA levels are shown as column graph on the top (mean ± SEM, n=2), and proteins are shown at the bottom. A transposon clone (B181) with insert in *NEDD4L* gene is also included in the assay.
(B) Proliferation of mutants either in the absence of drug (ND, solid lines) or presence of 0.5 μM PLX4720 (PLX, dash lines). Error bars denote SEM, n=3.
(C) IC50 of NEDD4L-mutant-expressing A375 cell lines treated with PLX4720, AZ628, AZD6244, and combination of PLX4720 and AZD6244. In the combinatorial treatment, PLX4720 and AZD6244 concentrations were fixed at 5:1 and the y-axis indicated PLX4720 concentrations. Error bars denote 95% CI.
(D) IC50 of PLX4720 of vector control and *NEDD4L*-overexpressing cells with *SMAD2/3* knockdown. Values are shown as mean ± 95% CI, n=3.
(E) *SMAD2/3* knockdown reduces both phospho- and total SMAD2/3.
(F) *NEDD4L* knockdown does not increase total or phospho-SMAD2/3.

A

| Patient | #7 | #12 | #25 | #2 | #6 | #13 | #24 | #19 | #34 | #9 | #22 | #16 | #15 | #10 |
|---|---|---|---|---|---|---|---|---|---|---|---|---|---|---|
| RECIST (shrink%) | 100 | 89 | 64 | 61 | 60 | 58 | 53 | 49 | 49 | 45 | 42 | 20 | 17 | 13 |
| PFS (month) | 17 | 12 | 3 | 9 | 21 | 9 | 2 | 17 | 15 | 7 | 3 | 11 | 6 | 3 |

B

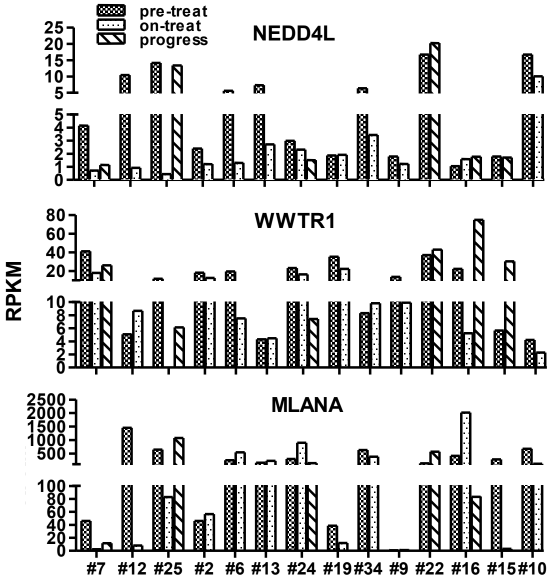


**Figure S6. WWTR1 and NEDD4L expression versus treatment outcome.**
(A) Response Evaluation Criteria In Solid Tumor (RECIST) and Progression Free Survival (PFS) are indicated for each patient.
(B) *NEDD4L*, *WWTR1*, and a melanoma biomaker *MLANA* mRNA expression in patient samples sorted by RECIST scores. Y-values indicated reads per kilobase million (RPKM) of RNA-Seq.

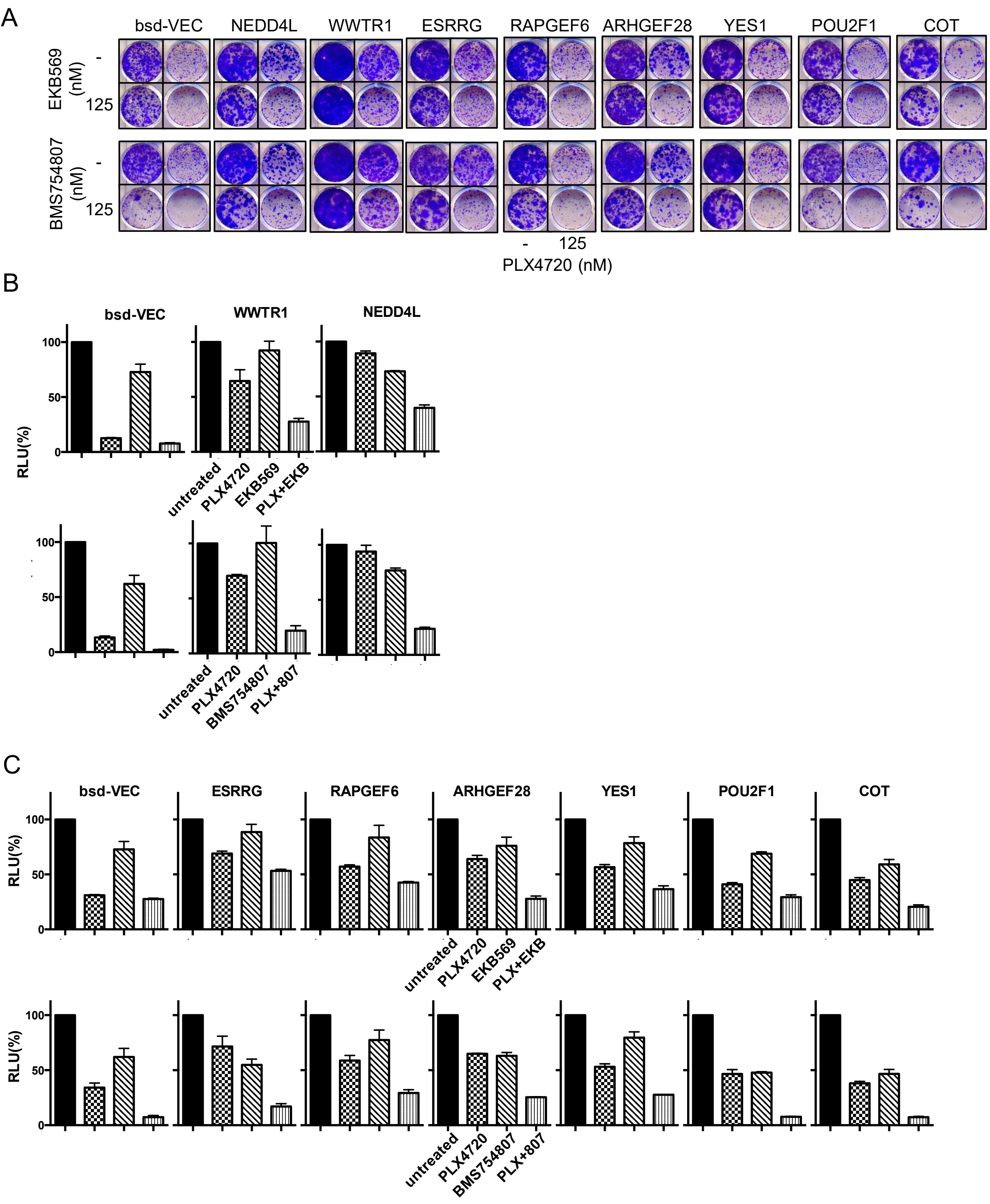


**Figure S7. EGFR and IGF1R inhibitions broadly resensitize resistant cells.**
(A) Resensitization measured by clonogenic assays after 12-day drug treatments.
(B) And (C) Responsiveness of resistance-gene-overexpressing A375 cells to 150nM PLX4720, 250nM EKB569, 250nM BMS754807, or combinations, in 5-day viability assays measured by CellTiter-Glo. Bars denote mean ± SEM, n=3. Note the two panels were prepared from different experiments and the baselines should only be compared within each panel.

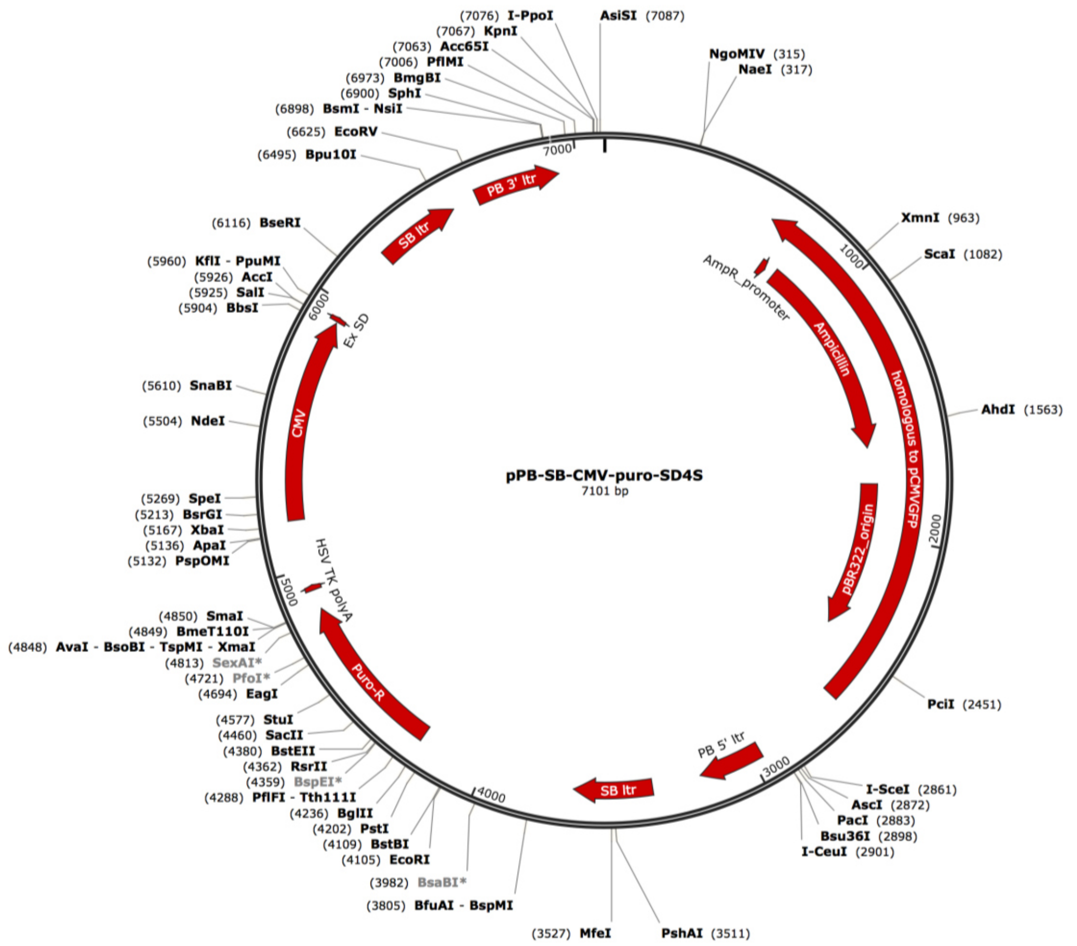


**Figure S8. Plasmid map of a PB-SB dual-LTR plasmid.**
Full sequence can be found in the Supplementary Text. Plasmid map is drawn using SnapGene.

**Table S1. Gene annotation of top 100 inserts in each resistant pools.**

Genes identified by the transposon screens were listed with gene name, location of the strongest insert, read number normalized to total reads, number of insert sites, number of insert events, and predicted effect on gene expression. Insert events refer to either hits in more than one resistant pool or multiple sites within a pool. High confidence hits are shaded. Activated genes predicted from insert orientation and proximity were marked with a "+".

| Gene | position | normalized read number | insert sites | insert events | effect prediction |
|---|---|---|---|---|---|
| NEDD4L | chr18:55950431 | 0.124757854 | 20 | 32 | + |
| EYA1 | chr8:72248874 | 0.089242154 | 5 | 11 | + |
| POU2F1 | chr1:167276097 | 0.001102743 | 9 | 9 | + |
| BRAF | chr7:140483217 | 0.068029828 | 3 | 8 | + |
| ZFHX3 | chr16:73044366 | 0.01682473 | 4 | 7 | + |
| TRPS1 | chr8:116654858 | 0.010065441 | 4 | 7 | + |
| ZFHX4 | chr8:77680504 | 0.004624953 | 6 | 7 | + |
| RAPGEF2 | chr4:160201207 | 0.001658968 | 5 | 7 | + |
| ARHGEF28 | chr5:73387835 | 0.061110225 | 4 | 6 | + |
| VAV1 | chr19:6781988 | 0.050608724 | 3 | 6 | + |
| PLS3 | chrX:115045927 | 0.053414439 | 2 | 5 | |
| GPC6 | chr13:94958370 | 0.041735035 | 4 | 5 | |
| WWTR1 | chr3:149386508 | 0.002125591 | 3 | 5 | + |
| RAPGEF6 | chr5:130954965 | 0.000865833 | 4 | 5 | + |
| AKAP13 | chr15:86140331 | 0.00015078 | 5 | 5 | + |
| CREM | chr10:35416711 | 0.083409724 | 1 | 4 | |
| LOC339622 | chr21:26523838 | 0.029561512 | 1 | 4 | |
| PABPC4L | chr4:136144718 | 0.028789137 | 1 | 4 | |
| ESRRG | chr1:217205344 | 0.000410911 | 4 | 4 | + |
| FOXP2 | chr7:114311067 | 0.000300021 | 4 | 4 | |
| HGSNAT | chr8:43068098 | 4.27768E-06 | 1 | 4 | |
| CHST9 | chr18:24786338 | 0.072273833 | 1 | 3 | + |
| YES1 | chr18:764184 | 0.003844651 | 1 | 3 | + |
| ITSN2 | chr2:24431266 | 0.002247845 | 1 | 3 | |
| UHMK1 | chr1:162458565 | 0.002008196 | 2 | 3 | + |
| KCNT2 | chr1:196390818 | 0.001911316 | 2 | 3 | + |
| TMTC2 | chr12:84130047 | 0.001428221 | 1 | 3 | |
| LINC00433 | chr13:89236670 | 0.001016377 | 2 | 3 | |
| CHRM3 | chr1:239740372 | 0.000693113 | 3 | 3 | + |
| RUNX1 | chr21:36556891 | 0.000603355 | 2 | 3 | + |
| PCDH7 | chr4:31968961 | 0.000294649 | 2 | 3 | |
| CDH6 | chr5:31075168 | 0.000270435 | 3 | 3 | + |
| ATP10A | chr15:26112320 | 0.000231617 | 2 | 3 | + |
| MECOM | chr3:169274502 | 0.000136276 | 3 | 3 | + |
| HNF4G | chr8:76554297 | 0.000110049 | 3 | 3 | + |
| PLSCR5 | chr3:146661822 | 9.40574E-05 | 3 | 3 | + |
| DCN | chr12:91640705 | 0.08953968 | 2 | 2 | + |
| GCKR | chr2:27754219 | 0.007159953 | 1 | 2 | |
| PRRX1 | chr1:170751740 | 0.006859031 | 1 | 2 | |
| HACE1 | chr6:105198339 | 0.006766768 | 1 | 2 | |
| IRF2 | chr4:185347059 | 0.004900672 | 1 | 2 | |
| PDE8A | chr15:85566976 | 0.003901193 | 1 | 2 | |
| GPR68 | chr14:91702157 | 0.003284196 | 1 | 2 | |
| ANTXR2 | chr4:81035247 | 0.002618165 | 2 | 2 | + |
| ZFPM2 | chr8:106720552 | 0.001970573 | 1 | 2 | |
| KCNIP4 | chr4:21761680 | 0.001840868 | 1 | 2 | |
| DOCK10 | chr2:225931999 | 0.001770084 | 1 | 2 | + |
| WIPF1 | chr2:175433818 | 0.001569414 | 1 | 2 | |
| ADAD1 | chr4:123354498 | 0.001347241 | 1 | 2 | |

| | | | | | |
|---|---|---|---|---|---|
| IFLTD1 | chr12:25892844 | 0.001336861 | 2 | 2 | + |
| SNX6 | chr14:35077412 | 0.001305134 | 1 | 2 | |
| MAGI1 | chr3:65546848 | 0.001198649 | 1 | 2 | |
| LGR5 | chr12:71972099 | 0.00117545 | 1 | 2 | |
| NINJ2 | chr12:741286 | 0.001099148 | 1 | 2 | |
| UGGT2 | chr13:96499087 | 0.000948455 | 1 | 2 | |
| TBX22 | chrX:79209729 | 0.000837512 | 1 | 2 | + |
| CCDC85A | chr2:57425826 | 0.000786263 | 1 | 2 | |
| MLIP | chr6:53988277 | 0.000763219 | 1 | 2 | |
| LINC00410 | chr13:91258712 | 0.000759805 | 1 | 2 | |
| CDH18 | chr5:19363294 | 0.000688564 | 1 | 2 | |
| LRIG2 | chr1:113570807 | 0.000663611 | 1 | 2 | + |
| SLC38A2 | chr12:46756165 | 0.000639206 | 1 | 2 | |
| KLF12 | chr13:74173242 | 0.000625679 | 1 | 2 | |
| IRF6 | chr1:209975965 | 0.000590138 | 1 | 2 | |
| CDC73 | chr1:193734204 | 0.000556566 | 2 | 2 | |
| ADHFE1 | chr8:67357341 | 0.000534965 | 1 | 2 | |
| CCDC146 | chr7:76848650 | 0.000507018 | 1 | 2 | |
| PRR20A | chr13:57324142 | 0.000493846 | 1 | 2 | + |
| ZNF507 | chr19:32855050 | 0.000477758 | 1 | 2 | |
| SLC33A1 | chr3:155564810 | 0.000449301 | 1 | 2 | |
| SLC35F3 | chr1:234111768 | 0.000429366 | 2 | 2 | |
| NELL2 | chr12:45065753 | 0.000409009 | 2 | 2 | |
| MMP2 | chr16:55481440 | 0.000372005 | 1 | 2 | + |
| CHM | chrX:85333141 | 0.000371544 | 1 | 2 | + |
| SEMA6A | chr5:115803054 | 0.000362896 | 1 | 2 | |
| ETV3 | chr1:157192476 | 0.000351274 | 1 | 2 | |
| SFRP1 | chr8:41071553 | 0.000345057 | 2 | 2 | |
| GRM8 | chr7:125487419 | 0.000322023 | 1 | 2 | |
| SUMO1P1 | chr20:52461649 | 0.000304771 | 2 | 2 | |
| EPHA7 | chr6:93926717 | 0.000246283 | 1 | 2 | |
| SKAP2 | chr7:26795532 | 0.000245428 | 1 | 2 | |
| TENM2 | chr5:167216727 | 0.000243735 | 2 | 2 | |
| GPR113 | chr2:26568090 | 0.000236747 | 1 | 2 | |
| SYT11 | chr1:155838789 | 0.000227882 | 1 | 2 | |
| PPP1R32 | chr11:61246147 | 0.000207267 | 1 | 2 | + |
| PDZRN4 | chr12:41919728 | 0.000207106 | 2 | 2 | |
| FAM160A1 | chr4:152311074 | 0.000200392 | 1 | 2 | |
| DLGAP1 | chr18:4229157 | 0.000196429 | 2 | 2 | + |
| DYNLRB2 | chr16:80297982 | 0.000190899 | 1 | 2 | + |
| PTPRQ | chr12:81083502 | 0.000169875 | 2 | 2 | |
| PTPRZ1 | chr7:121634925 | 0.000144196 | 2 | 2 | |
| LECT1 | chr13:53334923 | 0.000141454 | 2 | 2 | + |
| LOC440970 | chr3:84856401 | 0.00012964 | 2 | 2 | |
| RAF1 | chr3:12676395 | 0.000126965 | 2 | 2 | + |
| KCNIP1 | chr5:169960664 | 0.000125178 | 2 | 2 | + |
| PDZRN3 | chr3:73722713 | 0.00010972 | 2 | 2 | + |
| UBE3C | chr7:156946062 | 0.000103337 | 1 | 2 | |
| MCTP2 | chr15:95406428 | 0.000100099 | 2 | 2 | + |
| SCAF8 | chr6:155124040 | 9.42242E-05 | 2 | 2 | + |
| PDE4DIP | chr1:145079838 | 8.90799E-05 | 2 | 2 | + |
| BCAS3 | chr17:59299832 | 8.334E-05 | 2 | 2 | |
| LRRC4C | chr11:41574598 | 6.53463E-05 | 2 | 2 | |
| PDHA2 | chr4:96999245 | 6.47557E-05 | 2 | 2 | + |
| SOX5 | chr12:24034694 | 6.14684E-05 | 2 | 2 | + |
| C15orf54 | chr15:39449477 | 4.77028E-05 | 2 | 2 | + |

| | | | | | |
|---|---|---|---|---|---|
| ARID1A | chr1:27030884 | 4.65088E-05 | 2 | 2 | + |
| PIK3C2G | chr12:18640893 | 4.49689E-05 | 2 | 2 | |
| SEMA3D | chr7:84961632 | 4.01151E-05 | 2 | 2 | |
| PCDH10 | chr4:134339579 | 3.68791E-05 | 2 | 2 | |
| GALNTL6 | chr4:173827520 | 3.42353E-05 | 2 | 2 | |
| USP25 | chr21:17125119 | 3.39345E-05 | 2 | 2 | + |
| ISL1 | chr5:51204000 | 3.29618E-05 | 2 | 2 | |
| NRXN1 | chr2:52337792 | 3.06241E-05 | 2 | 2 | |
| DNAJC11 | chr1:6749931 | 2.81239E-05 | 1 | 2 | |
| TRIT1 | chr1:40353511 | 2.77346E-05 | 2 | 2 | + |
| MIER1 | chr1:67455740 | 2.76698E-05 | 2 | 2 | |
| DIRAS2 | chr9:93465100 | 2.45587E-05 | 2 | 2 | |
| MTIF2 | chr2:55481487 | 2.20394E-05 | 1 | 2 | |
| LSAMP | chr3:115708792 | 2.09054E-05 | 2 | 2 | |
| GLIS3 | chr9:4236392 | 2.0867E-05 | 2 | 2 | |
| KIAA1217 | chr10:24607748 | 1.81614E-05 | 2 | 2 | |
| AGBL1 | chr15:87833699 | 9.21117E-06 | 2 | 2 | |
| FAM5C | chr1:190399982 | 8.23231E-06 | 2 | 2 | |
| OR7E156P | chr13:65216283 | 5.42119E-06 | 2 | 2 | |
| RAPGEF4 | chr2:173736632 | 4.14936E-06 | 2 | 2 | + |
| MIR3134 | chr9:115061321 | 2.95895E-06 | 2 | 2 | + |
| LOC91948 | chr15:98411062 | 2.69692E-06 | 2 | 2 | |
| ELK3 | chr12:96527329 | 1.41589E-06 | 2 | 2 | |
| NEDD9 | chr6:11399655 | 0.024332366 | 1 | 1 | |
| KTN1 | chr14:56156632 | 0.006445131 | 1 | 1 | |
| SNX14 | chr6:86254232 | 0.003121453 | 1 | 1 | |
| PPARG | chr3:12393983 | 0.002801048 | 1 | 1 | |
| ADAM9 | chr8:38909781 | 0.002794283 | 1 | 1 | |
| PTEN | chr10:89665859 | 0.002759531 | 1 | 1 | |
| ALKBH3 | chr11:43900878 | 0.002112448 | 1 | 1 | |
| PTPLB | chr3:123224149 | 0.00202505 | 1 | 1 | |
| C11orf74 | chr11:36912050 | 0.002022545 | 1 | 1 | |
| POU2F2 | chr19:42623258 | 0.001859275 | 1 | 1 | |
| COL4A5 | chrX:107817324 | 0.001830524 | 1 | 1 | |
| LOC401164 | chr4:189360387 | 0.001553745 | 1 | 1 | |
| UTS2D | chr3:191032332 | 0.001398728 | 1 | 1 | |
| HHEX | chr10:94492359 | 0.001333613 | 1 | 1 | |
| PABPC5 | chrX:90362114 | 0.001107525 | 1 | 1 | |
| LOC284379 | chr19:54117726 | 0.0011036 | 1 | 1 | |
| LAP3 | chr4:17587613 | 0.001096961 | 1 | 1 | |
| LOC100507377 | chr12:74799042 | 0.00090126 | 1 | 1 | |
| RAPGEF1 | chr9:134558995 | 0.000668089 | 1 | 1 | |
| WAPAL | chr10:88167724 | 0.000632046 | 1 | 1 | |
| CEP85L | chr6:118976173 | 0.000612548 | 1 | 1 | |
| FST | chr5:52636515 | 0.000584702 | 1 | 1 | |
| PRICKLE2 | chr3:64278730 | 0.000568649 | 1 | 1 | |
| FAM178A | chr10:102700865 | 0.000562971 | 1 | 1 | |
| MAST4 | chr5:66026949 | 0.000464309 | 1 | 1 | |
| PSMD7 | chr16:73965121 | 0.000457342 | 1 | 1 | |
| LOC100506422 | chr9:26192125 | 0.000437125 | 1 | 1 | |
| TECRL | chr4:64478136 | 0.000436194 | 1 | 1 | |
| MPZL1 | chr1:167740772 | 0.000404302 | 1 | 1 | |
| LRP6 | chr12:12355944 | 0.000371183 | 1 | 1 | |
| VCPIP1 | chr8:67540411 | 0.000342114 | 1 | 1 | |
| RAB9BP1 | chr5:104221937 | 0.000328634 | 1 | 1 | |
| RNU6-19 | chr14:76775154 | 0.000319185 | 1 | 1 | |

| | | | | | |
|---|---|---|---|---|---|
| SH3RF3 | chr2:109801346 | 0.000300477 | 1 | 1 | |
| GDAP2 | chr1:118457277 | 0.00029428 | 1 | 1 | |
| DPRX | chr19:54139229 | 0.000289511 | 1 | 1 | |
| CCDC178 | chr18:30713405 | 0.000286508 | 1 | 1 | |
| LOC100505776 | chr18:69180152 | 0.000283114 | 1 | 1 | |
| GATA6 | chr18:19771280 | 0.000272498 | 1 | 1 | |
| C14orf37 | chr14:58642680 | 0.000262099 | 1 | 1 | |
| COG6 | chr13:40225285 | 0.000237566 | 1 | 1 | |
| SCAMP1 | chr5:77670237 | 0.000234822 | 1 | 1 | |
| SGMS1 | chr10:52360891 | 0.000234641 | 1 | 1 | |
| FRMD4B | chr3:69422307 | 0.000231077 | 1 | 1 | |
| IL1RAPL1 | chrX:28570271 | 0.000207124 | 1 | 1 | |
| RASGRF1 | chr15:79255277 | 0.000204199 | 1 | 1 | |
| DUSP10 | chr1:222146289 | 0.000201623 | 1 | 1 | |
| CDH8 | chr16:61304485 | 0.000201089 | 1 | 1 | |
| FASLG | chr1:172707625 | 0.000197864 | 1 | 1 | |
| C9orf84 | chr9:114583916 | 0.00019553 | 1 | 1 | |
| GTF2E1 | chr3:120474691 | 0.000194862 | 1 | 1 | |
| NBAS | chr2:15249735 | 0.000190784 | 1 | 1 | |
| SLC6A15 | chr12:84560099 | 0.000187427 | 1 | 1 | |
| BTG1 | chr12:92658834 | 0.000185961 | 1 | 1 | |
| SMIM13 | chr6:11136824 | 0.000184718 | 1 | 1 | |
| DRAM2 | chr1:111642324 | 0.000182365 | 1 | 1 | |
| NENF | chr1:212609814 | 0.000181167 | 1 | 1 | |
| ATXN1 | chr6:16879247 | 0.000181109 | 1 | 1 | |
| LINC00550 | chr13:69856519 | 0.00017871 | 1 | 1 | |
| TMPRSS15 | chr21:19717179 | 0.000174528 | 1 | 1 | |
| BARX2 | chr11:129484977 | 0.000174512 | 1 | 1 | |
| AGBL3 | chr7:134820438 | 0.000171148 | 1 | 1 | |
| MBD5 | chr2:149292679 | 0.000169467 | 1 | 1 | |
| SRSF4 | chr1:29491984 | 0.000164962 | 1 | 1 | |
| CLCN3 | chr4:170556703 | 0.000162908 | 1 | 1 | |
| MIR4436A | chr2:89155362 | 0.000162436 | 1 | 1 | |
| OTUD1 | chr10:23793288 | 0.000161517 | 1 | 1 | |
| PKIG | chr20:43246434 | 0.000161192 | 1 | 1 | |
| LCORL | chr4:17979366 | 0.000158304 | 1 | 1 | |
| P4HA3 | chr11:73974432 | 0.000157544 | 1 | 1 | |
| HAO1 | chr20:7458615 | 0.000157133 | 1 | 1 | |
| PIAS1 | chr15:68420179 | 0.000145008 | 1 | 1 | |
| HMBOX1 | chr8:28774123 | 0.000142904 | 1 | 1 | |
| HMHB1 | chr5:143268331 | 0.000137041 | 1 | 1 | |
| ZNF618 | chr9:116695185 | 0.000134435 | 1 | 1 | |
| BBS12 | chr4:123680569 | 0.000131275 | 1 | 1 | |
| NAV3 | chr12:78560659 | 0.000130615 | 1 | 1 | |
| DUSP3 | chr17:41841141 | 0.000127732 | 1 | 1 | |
| PROM1 | chr4:16016722 | 0.000126191 | 1 | 1 | |
| VPS13B | chr8:100702251 | 0.000125194 | 1 | 1 | |
| ZFAND5 | chr9:74951759 | 0.000120305 | 1 | 1 | |
| PDZD2 | chr5:32008137 | 0.000118319 | 1 | 1 | |
| LHX8 | chr1:75417683 | 0.000117168 | 1 | 1 | |
| TCF7L2 | chr10:114647939 | 0.000116324 | 1 | 1 | |
| HSF2 | chr6:122738804 | 0.000112035 | 1 | 1 | |
| MCTP1 | chr5:94170273 | 0.000110972 | 1 | 1 | |
| TCP11 | chr6:35124410 | 0.000110721 | 1 | 1 | |
| ASTN2 | chr9:119655075 | 0.000110574 | 1 | 1 | |
| SFTA1P | chr10:10833674 | 0.000102609 | 1 | 1 | |

| | | | | | |
|---|---|---|---|---|---|
| DYRK2 | chr12:68031055 | 0.000101977 | 1 | 1 | |
| CD38 | chr4:15883016 | 0.000101349 | 1 | 1 | |
| MIR4302 | chr12:25920639 | 0.000100749 | 1 | 1 | |
| ZCRB1 | chr12:42696421 | 9.90296E-05 | 1 | 1 | |
| MNAT1 | chr14:61198115 | 9.72652E-05 | 1 | 1 | |
| CCSER1 | chr4:91493890 | 9.72563E-05 | 1 | 1 | |
| P4HA1 | chr10:74826558 | 9.58511E-05 | 1 | 1 | |
| CBX5 | chr12:54628029 | 9.54831E-05 | 1 | 1 | |
| C7orf66 | chr7:108979702 | 9.39268E-05 | 1 | 1 | |
| UBE2D2 | chr5:138912792 | 9.04708E-05 | 1 | 1 | |
| TPH2 | chr12:72457080 | 8.99904E-05 | 1 | 1 | |
| PUS7L | chr12:44097394 | 8.96976E-05 | 1 | 1 | |
| SAMD4A | chr14:55055626 | 8.82273E-05 | 1 | 1 | |
| LOC100216546 | chr7:104640474 | 8.78984E-05 | 1 | 1 | |
| ROCK1 | chr18:18656553 | 8.75247E-05 | 1 | 1 | |
| ELMO1 | chr7:37331862 | 8.60423E-05 | 1 | 1 | |
| DYM | chr18:46661964 | 8.41502E-05 | 1 | 1 | |
| ZNF608 | chr5:124588940 | 8.28506E-05 | 1 | 1 | |
| MYSM1 | chr1:59127758 | 8.05237E-05 | 1 | 1 | |
| ARID1B | chr6:157315777 | 7.90638E-05 | 1 | 1 | |
| PGAP1 | chr2:197779319 | 7.64605E-05 | 1 | 1 | |
| LOC100129034 | chr9:127120324 | 7.5283E-05 | 1 | 1 | |
| TRERF1 | chr6:42357215 | 7.30489E-05 | 1 | 1 | |
| CAMK2G | chr10:75632940 | 7.20827E-05 | 1 | 1 | |
| ACIN1 | chr14:23546344 | 7.10684E-05 | 1 | 1 | |
| FAT2 | chr5:150990641 | 6.92838E-05 | 1 | 1 | |
| MTRNR2L6 | chr7:142395525 | 6.90509E-05 | 1 | 1 | |
| KIAA1731 | chr11:93408275 | 6.85355E-05 | 1 | 1 | |
| TMEM229A | chr7:123768606 | 6.808E-05 | 1 | 1 | |
| AKIRIN2 | chr6:88441345 | 6.7729E-05 | 1 | 1 | |
| GPR110 | chr6:47018714 | 6.76516E-05 | 1 | 1 | |
| RP1-177G6.2 | chrX:139737858 | 6.4339E-05 | 1 | 1 | |
| ABTB2 | chr11:34343365 | 6.42998E-05 | 1 | 1 | |
| RNF8 | chr6:37319198 | 6.39392E-05 | 1 | 1 | |
| ZNF804A | chr2:185198546 | 6.3305E-05 | 1 | 1 | |
| PAPLN | chr14:73722879 | 6.22693E-05 | 1 | 1 | |
| GOT2 | chr16:58824041 | 6.08224E-05 | 1 | 1 | |
| PAIP2B | chr2:71438858 | 6.02185E-05 | 1 | 1 | |
| LOC401497 | chr9:30899633 | 5.74567E-05 | 1 | 1 | |
| ANXA2P3 | chr10:66953016 | 5.67769E-05 | 1 | 1 | |
| MBOAT1 | chr6:20174163 | 5.66822E-05 | 1 | 1 | |
| CSGALNACT1 | chr8:19416805 | 5.62751E-05 | 1 | 1 | |
| GPR156 | chr3:119865469 | 5.61131E-05 | 1 | 1 | |
| LMO7 | chr13:76425598 | 5.61024E-05 | 1 | 1 | |
| DTNA | chr18:32437976 | 5.59681E-05 | 1 | 1 | |
| RAI1 | chr17:17646823 | 5.59563E-05 | 1 | 1 | |
| ACTL7B | chr9:111523123 | 5.55069E-05 | 1 | 1 | |
| PURG | chr8:30840266 | 5.44583E-05 | 1 | 1 | |
| TFAP2D | chr6:50554229 | 5.40382E-05 | 1 | 1 | |
| ANKRD28 | chr3:15833763 | 5.39339E-05 | 1 | 1 | |
| PCDH17 | chr13:59109348 | 5.39287E-05 | 1 | 1 | |
| ZNF438 | chr10:31248923 | 5.35428E-05 | 1 | 1 | |
| RBMXL2 | chr11:7160212 | 5.33194E-05 | 1 | 1 | |
| ERCC6L2 | chr9:98679479 | 5.28701E-05 | 1 | 1 | |
| ADPGK-AS1 | chr15:73097963 | 5.05462E-05 | 1 | 1 | |
| MIR4437 | chr2:182103309 | 4.96166E-05 | 1 | 1 | |

| | | | | | |
|---|---|---|---|---|---|
| KLHL12 | chr1:202883375 | 4.92609E-05 | 1 | 1 | |
| AMOTL1 | chr11:94497780 | 4.92558E-05 | 1 | 1 | |
| COL24A1 | chr1:86208901 | 4.88246E-05 | 1 | 1 | |
| DOK6 | chr18:67322462 | 4.81471E-05 | 1 | 1 | |
| KRT18 | chr12:53362164 | 4.80859E-05 | 1 | 1 | |
| KRIT1 | chr7:91848849 | 4.8085E-05 | 1 | 1 | |
| LCP1 | chr13:46705895 | 4.79561E-05 | 1 | 1 | |
| CSK | chr15:75075243 | 4.77474E-05 | 1 | 1 | |
| HSPB1 | chr7:75933515 | 4.73874E-05 | 1 | 1 | |
| RBM19 | chr12:114250490 | 4.70785E-05 | 1 | 1 | |
| MAP4K5 | chr14:50888767 | 4.67941E-05 | 1 | 1 | |
| UBE2E2 | chr3:23508763 | 4.63397E-05 | 1 | 1 | |
| GUSBP1 | chr5:21664122 | 4.52283E-05 | 1 | 1 | |
| QPCT | chr2:37620005 | 4.51275E-05 | 1 | 1 | |
| RREB1 | chr6:7112902 | 4.49773E-05 | 1 | 1 | |
| LRP1B | chr2:141219428 | 4.44781E-05 | 1 | 1 | |
| TAF2 | chr8:120720152 | 4.34559E-05 | 1 | 1 | |
| ARL6 | chr3:97506979 | 4.33274E-05 | 1 | 1 | |
| PRRC2C | chr1:171537561 | 4.2849E-05 | 1 | 1 | |
| TMC1 | chr9:75168048 | 4.25035E-05 | 1 | 1 | |
| TMTC3 | chr12:88734320 | 4.23497E-05 | 1 | 1 | |
| FGFR1 | chr8:38331127 | 4.22151E-05 | 1 | 1 | |
| OXR1 | chr8:107082285 | 4.20639E-05 | 1 | 1 | |
| ZBTB20 | chr3:114766652 | 4.20507E-05 | 1 | 1 | |
| SMG9 | chr19:44251969 | 4.08946E-05 | 1 | 1 | |
| SP4 | chr7:21447248 | 4.0294E-05 | 1 | 1 | |
| FOXB2 | chr9:79709245 | 3.95902E-05 | 1 | 1 | |
| OLFML2A | chr9:127587348 | 3.93015E-05 | 1 | 1 | |
| BCKDHB | chr6:81225741 | 3.91515E-05 | 1 | 1 | |
| CDKAL1 | chr6:21185855 | 3.86048E-05 | 1 | 1 | |
| HSD52 | chr1:59582933 | 3.85298E-05 | 1 | 1 | |
| GPRIN3 | chr4:90172371 | 3.84548E-05 | 1 | 1 | |
| BHLHE40 | chr3:5095217 | 3.81163E-05 | 1 | 1 | |
| PPAP2B | chr1:56747830 | 3.74747E-05 | 1 | 1 | |
| ACTBL2 | chr5:56965484 | 3.71515E-05 | 1 | 1 | |
| MED1 | chr17:37572098 | 3.70397E-05 | 1 | 1 | |
| CDK8 | chr13:26934427 | 3.67716E-05 | 1 | 1 | |
| GAREM | chr18:29969017 | 3.67529E-05 | 1 | 1 | |
| NR2C1 | chr12:95441336 | 3.65705E-05 | 1 | 1 | |
| FAM27A | chr9:45685367 | 3.6455E-05 | 1 | 1 | |
| FRAT1 | chr10:99068319 | 3.61826E-05 | 1 | 1 | |
| IQCH | chr15:67789776 | 3.57535E-05 | 1 | 1 | |
| SLC25A33 | chr1:9560677 | 3.53823E-05 | 1 | 1 | |
| EPHX1 | chr1:226004628 | 3.38355E-05 | 1 | 1 | |
| LPAR1 | chr9:113721504 | 3.36818E-05 | 1 | 1 | |
| NLN | chr5:65140753 | 3.36318E-05 | 1 | 1 | |
| DPY19L2P4 | chr7:89599212 | 3.34043E-05 | 1 | 1 | |
| GLG1 | chr16:74519476 | 3.22136E-05 | 1 | 1 | |
| RGS5 | chr1:163187061 | 3.18098E-05 | 1 | 1 | |
| PHLDB2 | chr3:111568188 | 3.16765E-05 | 1 | 1 | |
| CYLC2 | chr9:105193929 | 3.13556E-05 | 1 | 1 | |
| LINC00351 | chr13:85586013 | 2.98888E-05 | 1 | 1 | |
| TXNDC16 | chr14:52899792 | 2.90843E-05 | 1 | 1 | |
| MIRLET7BHG | chr22:46526092 | 2.90711E-05 | 1 | 1 | |
| CDH9 | chr5:27106151 | 2.85165E-05 | 1 | 1 | |
| KLF5 | chr13:73863701 | 2.77442E-05 | 1 | 1 | |

| | | | | | |
|---|---|---|---|---|---|
| CCDC102B | chr18:66475694 | 2.75966E-05 | 1 | 1 | |
| TMEM196 | chr7:19932078 | 2.72821E-05 | 1 | 1 | |
| ALX1 | chr12:85661232 | 2.7272E-05 | 1 | 1 | |
| AHCYL1 | chr1:110553387 | 2.72334E-05 | 1 | 1 | |
| GPM6B | chrX:13882333 | 2.68053E-05 | 1 | 1 | |
| ABLIM1 | chr10:116427520 | 2.553E-05 | 1 | 1 | |
| YPEL5 | chr2:30322575 | 2.51971E-05 | 1 | 1 | |
| KRT34 | chr17:39534696 | 2.50731E-05 | 1 | 1 | |
| LINC00536 | chr8:116825989 | 2.41457E-05 | 1 | 1 | |
| LSP1P3 | chr5:28475743 | 2.40603E-05 | 1 | 1 | |
| BAI3 | chr6:69382550 | 2.40007E-05 | 1 | 1 | |
| ZFAND3 | chr6:37730334 | 2.36736E-05 | 1 | 1 | |
| IGJ | chr4:71542320 | 2.35573E-05 | 1 | 1 | |
| TMEM38B | chr9:108962020 | 2.35535E-05 | 1 | 1 | |
| ZCCHC24 | chr10:81188241 | 2.33524E-05 | 1 | 1 | |
| SYBU | chr8:110625565 | 2.2636E-05 | 1 | 1 | |
| C2orf71 | chr2:29314365 | 2.25601E-05 | 1 | 1 | |
| RBM46 | chr4:155771549 | 2.2559E-05 | 1 | 1 | |
| RAB11FIP5 | chr2:73356872 | 2.24173E-05 | 1 | 1 | |
| MTHFS | chr15:80135763 | 2.24103E-05 | 1 | 1 | |
| TPST1 | chr7:65721676 | 2.18264E-05 | 1 | 1 | |
| RORB | chr9:77131382 | 2.16462E-05 | 1 | 1 | |
| PITX2 | chr4:111544487 | 2.15468E-05 | 1 | 1 | |
| MIR221 | chrX:45504047 | 2.1006E-05 | 1 | 1 | |
| DSC3 | chr18:28537934 | 2.05992E-05 | 1 | 1 | |
| KCNK2 | chr1:215165498 | 2.04163E-05 | 1 | 1 | |
| KALRN | chr3:123942617 | 1.96282E-05 | 1 | 1 | |
| LINC00837 | chr10:29314876 | 1.96205E-05 | 1 | 1 | |
| GPNMB | chr7:23288228 | 1.95489E-05 | 1 | 1 | |
| PYDC2 | chr3:191295304 | 1.91287E-05 | 1 | 1 | |
| NAA50 | chr3:113453595 | 1.89053E-05 | 1 | 1 | |
| FBXO15 | chr18:71437358 | 1.89048E-05 | 1 | 1 | |
| C1orf105 | chr1:172437100 | 1.86372E-05 | 1 | 1 | |
| AHCY | chr20:32925180 | 1.85906E-05 | 1 | 1 | |
| EPHA8 | chr1:22924427 | 1.85744E-05 | 1 | 1 | |
| TSN | chr2:123480299 | 1.84814E-05 | 1 | 1 | |
| ELF3 | chr1:202011572 | 1.83907E-05 | 1 | 1 | |
| VDAC3 | chr8:42250115 | 1.82722E-05 | 1 | 1 | |
| HDX | chrX:83743207 | 1.80766E-05 | 1 | 1 | |
| TOX3 | chr16:52506371 | 1.7627E-05 | 1 | 1 | |
| CGGBP1 | chr3:88134242 | 1.75769E-05 | 1 | 1 | |
| PKHD1 | chr6:51733532 | 1.72541E-05 | 1 | 1 | |
| MIPEPP3 | chr13:21943685 | 1.68925E-05 | 1 | 1 | |
| FAM3C | chr7:121220335 | 1.68088E-05 | 1 | 1 | |
| RYR2 | chr1:237907819 | 1.6718E-05 | 1 | 1 | |
| DLG1 | chr3:196836288 | 1.60673E-05 | 1 | 1 | |
| SEMA3C | chr7:80719031 | 1.60649E-05 | 1 | 1 | |
| SLC24A2 | chr9:19818017 | 1.6045E-05 | 1 | 1 | |
| SERPINB3 | chr18:61326903 | 1.60115E-05 | 1 | 1 | |
| ZNF536 | chr19:31069617 | 1.57525E-05 | 1 | 1 | |
| ERP27 | chr12:15062621 | 1.57488E-05 | 1 | 1 | |
| PCDH20 | chr13:63139191 | 1.5712E-05 | 1 | 1 | |
| LOC642366 | chr5:50593760 | 1.5637E-05 | 1 | 1 | |
| ZNF695 | chr1:247165170 | 1.5531E-05 | 1 | 1 | |
| SPATA6 | chr1:48963284 | 1.54333E-05 | 1 | 1 | |
| EOMES | chr3:27645524 | 1.53969E-05 | 1 | 1 | |

| | | | | | |
|---|---|---|---|---|---|
| SND1 | chr7:127603737 | 1.52989E-05 | 1 | 1 | |
| FAM208A | chr3:56676019 | 1.49966E-05 | 1 | 1 | |
| ADAMTSL3 | chr15:84337574 | 1.46647E-05 | 1 | 1 | |
| MIR100HG | chr11:121957408 | 1.46204E-05 | 1 | 1 | |
| ARPP21 | chr3:35949810 | 1.43927E-05 | 1 | 1 | |
| IGDCC4 | chr15:65704486 | 1.43505E-05 | 1 | 1 | |
| MARCH6 | chr5:10331938 | 1.42572E-05 | 1 | 1 | |
| MURC | chr9:103408070 | 1.41928E-05 | 1 | 1 | |
| ROBO1 | chr3:80578500 | 1.40215E-05 | 1 | 1 | |
| ADAMTSL1 | chr9:18692850 | 1.3813E-05 | 1 | 1 | |
| LINC00704 | chr10:4272123 | 1.35474E-05 | 1 | 1 | |
| AR | chrX:66873356 | 1.33857E-05 | 1 | 1 | |
| CACNA1E | chr1:181361535 | 1.30433E-05 | 1 | 1 | |
| DOCK6 | chr19:11318495 | 1.29611E-05 | 1 | 1 | |
| ERCC3 | chr2:128044274 | 1.28936E-05 | 1 | 1 | |
| RPS7P5 | chr1:240122402 | 1.27968E-05 | 1 | 1 | |
| VENTXP1 | chrX:26777915 | 1.27647E-05 | 1 | 1 | |
| NAT2 | chr8:18291899 | 1.25717E-05 | 1 | 1 | |
| MIR3977 | chr5:82050552 | 1.22125E-05 | 1 | 1 | |
| GALNT10 | chr5:153641583 | 1.21645E-05 | 1 | 1 | |
| FAM96A | chr15:64379065 | 1.2143E-05 | 1 | 1 | |
| AHR | chr7:17312579 | 1.20511E-05 | 1 | 1 | |
| EIF4G2 | chr11:10815129 | 1.20188E-05 | 1 | 1 | |
| NHSL1 | chr6:138915744 | 1.1963E-05 | 1 | 1 | |
| MIR4493 | chr11:123177064 | 1.1598E-05 | 1 | 1 | |
| DNAJC3 | chr13:96401602 | 1.15404E-05 | 1 | 1 | |
| ARL8B | chr3:5211672 | 1.13968E-05 | 1 | 1 | |
| KCNH8 | chr3:19088235 | 1.13943E-05 | 1 | 1 | |
| ENC1 | chr5:73919237 | 1.11677E-05 | 1 | 1 | |
| LINC00333 | chr13:85212244 | 1.08231E-05 | 1 | 1 | |
| NEGR1 | chr1:73047190 | 1.05722E-05 | 1 | 1 | |
| TCF7L1 | chr2:85333922 | 1.05412E-05 | 1 | 1 | |
| UGP2 | chr2:64093710 | 1.04818E-05 | 1 | 1 | |
| SPIRE1 | chr18:12605124 | 1.01675E-05 | 1 | 1 | |
| PMS1 | chr2:190650215 | 9.67181E-06 | 1 | 1 | |
| SBF2 | chr11:9908279 | 9.62697E-06 | 1 | 1 | |
| ANXA10 | chr4:168631705 | 9.57082E-06 | 1 | 1 | |
| RLF | chr1:40599751 | 9.48093E-06 | 1 | 1 | |
| ALG9 | chr11:111658727 | 9.47436E-06 | 1 | 1 | |
| SEL1L | chr14:82524065 | 9.46385E-06 | 1 | 1 | |
| MTX2 | chr2:177272555 | 9.46364E-06 | 1 | 1 | |
| ABI3BP | chr3:100731128 | 9.24807E-06 | 1 | 1 | |
| MCPH1 | chr8:6494210 | 9.19051E-06 | 1 | 1 | |
| YTHDC2 | chr5:112905776 | 8.72641E-06 | 1 | 1 | |
| GABRA5 | chr15:27135387 | 8.6035E-06 | 1 | 1 | |
| PCGEM1 | chr2:193408806 | 8.29414E-06 | 1 | 1 | |
| RAP1GDS1 | chr4:99342850 | 8.19189E-06 | 1 | 1 | |
| TPRKB | chr2:73946755 | 8.11187E-06 | 1 | 1 | |
| ZNF708 | chr19:21442024 | 7.95246E-06 | 1 | 1 | |
| LINC00589 | chr8:29416568 | 7.88816E-06 | 1 | 1 | |
| RSBN1L | chr7:77414403 | 7.69393E-06 | 1 | 1 | |
| ALG10 | chr12:33971946 | 7.58899E-06 | 1 | 1 | |
| SOX6 | chr11:16541400 | 7.54519E-06 | 1 | 1 | |
| ABI1 | chr10:27103979 | 7.54203E-06 | 1 | 1 | |
| KLHL14 | chr18:30285373 | 7.53086E-06 | 1 | 1 | |
| SEMA3A | chr7:83666172 | 7.46382E-06 | 1 | 1 | |

| | | | | | |
|---|---|---|---|---|---|
| TAF1B | chr2:10067917 | 7.45338E-06 | 1 | 1 | |
| VAPB | chr20:56963565 | 7.36405E-06 | 1 | 1 | |
| NAV2 | chr11:19709216 | 7.29101E-06 | 1 | 1 | |
| ZNF217 | chr20:52299695 | 7.16043E-06 | 1 | 1 | |
| CTDSPL | chr3:37973819 | 7.10822E-06 | 1 | 1 | |
| ZNF559 | chr19:9453215 | 7.00571E-06 | 1 | 1 | |
| CCDC14 | chr3:123671688 | 6.6342E-06 | 1 | 1 | |
| SCLT1 | chr4:129941085 | 6.60646E-06 | 1 | 1 | |
| DEFB121 | chr20:29990873 | 6.56519E-06 | 1 | 1 | |
| PLK1S1 | chr20:21138992 | 6.50559E-06 | 1 | 1 | |
| LSM14A | chr19:34493625 | 6.3831E-06 | 1 | 1 | |
| NPAS2 | chr2:101517561 | 6.19736E-06 | 1 | 1 | |
| MEFV | chr16:3296165 | 6.1585E-06 | 1 | 1 | |
| IPO5 | chr13:98648879 | 6.10066E-06 | 1 | 1 | |
| C16orf52 | chr16:22058083 | 6.04386E-06 | 1 | 1 | |
| ETS1 | chr11:127840164 | 6.01073E-06 | 1 | 1 | |
| LOC100188947 | chr10:93348658 | 5.92874E-06 | 1 | 1 | |
| PTGER3 | chr1:71429731 | 5.85181E-06 | 1 | 1 | |
| HABP2 | chr10:115300211 | 5.78751E-06 | 1 | 1 | |
| AMY1A | chr1:104294376 | 5.76607E-06 | 1 | 1 | |
| LINC00210 | chr1:218159405 | 5.73996E-06 | 1 | 1 | |
| PKD2L1 | chr10:102048658 | 5.68285E-06 | 1 | 1 | |
| SLC6A7 | chr5:149583133 | 5.66556E-06 | 1 | 1 | |
| LOC100129620 | chr1:99503747 | 5.6649E-06 | 1 | 1 | |
| ANK3 | chr10:62067747 | 5.60295E-06 | 1 | 1 | |
| GRIN2A | chr16:9808539 | 5.48295E-06 | 1 | 1 | |
| PDLIM1 | chr10:97000006 | 5.4357E-06 | 1 | 1 | |
| LPHN2 | chr1:82373115 | 5.42215E-06 | 1 | 1 | |
| SOBP | chr6:107963851 | 5.38869E-06 | 1 | 1 | |
| GSG1 | chr12:13302527 | 5.34016E-06 | 1 | 1 | |
| RGMB | chr5:97418590 | 5.15093E-06 | 1 | 1 | |
| CT64 | chr3:163079044 | 5.08367E-06 | 1 | 1 | |
| PIP4K2A | chr10:22880689 | 5.08315E-06 | 1 | 1 | |
| DENND4C | chr9:19355541 | 5.07271E-06 | 1 | 1 | |
| EN1 | chr2:119493025 | 4.98199E-06 | 1 | 1 | |
| ARHGAP32 | chr11:129093644 | 4.9756E-06 | 1 | 1 | |
| PSMD12 | chr17:65331548 | 4.94981E-06 | 1 | 1 | |
| PLA2G4A | chr1:188035074 | 4.94037E-06 | 1 | 1 | |
| PRPF38A | chr1:52880637 | 4.85031E-06 | 1 | 1 | |
| RAPGEF5 | chr7:22296797 | 4.84925E-06 | 1 | 1 | |
| GLI3 | chr7:42189731 | 4.62596E-06 | 1 | 1 | |
| RNF180 | chr5:63626449 | 4.6058E-06 | 1 | 1 | |
| INTS9 | chr8:28714621 | 4.5172E-06 | 1 | 1 | |
| IRF2BP2 | chr1:234742591 | 4.43218E-06 | 1 | 1 | |
| FAM129A | chr1:184901547 | 4.29028E-06 | 1 | 1 | |
| S100A2 | chr1:153541101 | 4.28355E-06 | 1 | 1 | |
| PAQR3 | chr4:79845878 | 4.2833E-06 | 1 | 1 | |
| TMED7-TICAM2 | chr5:115001591 | 4.24123E-06 | 1 | 1 | |
| PIK3R5 | chr17:8818652 | 4.21553E-06 | 1 | 1 | |
| LINC00221 | chr14:106843957 | 4.21275E-06 | 1 | 1 | |
| EIF3IP1 | chr7:109097341 | 4.14079E-06 | 1 | 1 | |
| ATXN8OS | chr13:70945183 | 4.09594E-06 | 1 | 1 | |
| LYZL4 | chr3:42414473 | 4.06605E-06 | 1 | 1 | |
| TRIM62 | chr1:33629213 | 4.03788E-06 | 1 | 1 | |
| AKAP6 | chr14:33216678 | 4.03788E-06 | 1 | 1 | |
| TTC14 | chr3:180228211 | 4.02654E-06 | 1 | 1 | |

| | | | | | |
|---|---|---|---|---|---|
| AADAC | chr3:151557990 | 4.0011E-06 | 1 | 1 | |
| GALNT8 | chr12:4847122 | 3.94087E-06 | 1 | 1 | |
| LOC100506474 | chr2:13119738 | 3.91068E-06 | 1 | 1 | |
| GABRA2 | chr4:46222111 | 3.88376E-06 | 1 | 1 | |
| LOC255130 | chr4:59780875 | 3.88376E-06 | 1 | 1 | |
| DOCK7 | chr1:62982053 | 3.84182E-06 | 1 | 1 | |
| SLC36A4 | chr11:92905307 | 3.79954E-06 | 1 | 1 | |
| ZNF107 | chr7:64164544 | 3.72733E-06 | 1 | 1 | |
| LRRIQ1 | chr12:85651067 | 3.69757E-06 | 1 | 1 | |
| BCLAF1 | chr6:136625087 | 3.59895E-06 | 1 | 1 | |
| TMEM248 | chr7:66350776 | 3.45107E-06 | 1 | 1 | |
| PIWIL2 | chr8:22149931 | 3.39336E-06 | 1 | 1 | |
| MAP7D2 | chrX:20132880 | 3.36296E-06 | 1 | 1 | |
| TEX26 | chr13:31561229 | 3.35201E-06 | 1 | 1 | |
| OSBPL8 | chr12:76791392 | 3.34389E-06 | 1 | 1 | |
| IL1RAPL2 | chrX:104966813 | 3.34084E-06 | 1 | 1 | |
| SSBP2 | chr5:80897421 | 3.28429E-06 | 1 | 1 | |
| NUPR1L | chr7:56329635 | 3.28429E-06 | 1 | 1 | |
| SLC37A2 | chr11:124927958 | 3.2826E-06 | 1 | 1 | |
| CASK | chrX:41853829 | 3.25721E-06 | 1 | 1 | |
| TENM3 | chr4:183388116 | 3.22892E-06 | 1 | 1 | |
| AOX1 | chr2:201459144 | 3.19559E-06 | 1 | 1 | |
| PDS5A | chr4:40009189 | 3.19005E-06 | 1 | 1 | |
| PCDH15 | chr10:55372252 | 3.09438E-06 | 1 | 1 | |
| KIAA1109 | chr4:123192860 | 3.0556E-06 | 1 | 1 | |
| PRB2 | chr12:11586586 | 3.02115E-06 | 1 | 1 | |
| BCAT1 | chr12:24859438 | 3.00663E-06 | 1 | 1 | |
| PAPSS2 | chr10:89488360 | 2.94489E-06 | 1 | 1 | |
| MIR31HG | chr9:21509765 | 2.90447E-06 | 1 | 1 | |
| KSR2 | chr12:118325199 | 2.89096E-06 | 1 | 1 | |
| MIR548AS | chr13:104673300 | 2.87156E-06 | 1 | 1 | |
| DCTN4 | chr5:150144552 | 2.83804E-06 | 1 | 1 | |
| MIR4465 | chr6:141622859 | 2.83201E-06 | 1 | 1 | |
| KRAS | chr12:25406576 | 2.81569E-06 | 1 | 1 | |
| EBF2 | chr8:25916384 | 2.8123E-06 | 1 | 1 | |
| ALPL | chr1:21841118 | 2.80178E-06 | 1 | 1 | |
| ZMYM4 | chr1:35753055 | 2.71513E-06 | 1 | 1 | |
| ANK1 | chr8:41769846 | 2.63097E-06 | 1 | 1 | |
| MIR4500HG | chr13:87296318 | 2.59366E-06 | 1 | 1 | |
| GPR98 | chr5:90554750 | 2.58105E-06 | 1 | 1 | |
| DISP1 | chr1:223147051 | 2.53697E-06 | 1 | 1 | |
| PTPRU | chr1:29835532 | 2.49429E-06 | 1 | 1 | |
| MCF2 | chrX:138677077 | 2.44016E-06 | 1 | 1 | |
| TLE4 | chr9:82006428 | 2.40228E-06 | 1 | 1 | |
| CPSF2 | chr14:92628564 | 2.3403E-06 | 1 | 1 | |
| NOX4 | chr11:89076178 | 2.31312E-06 | 1 | 1 | |
| ZFP37 | chr9:115794661 | 2.29786E-06 | 1 | 1 | |
| RAB28 | chr4:13377256 | 2.2722E-06 | 1 | 1 | |
| LOC340094 | chr5:4546809 | 2.26141E-06 | 1 | 1 | |
| KCND2 | chr7:119880465 | 2.25601E-06 | 1 | 1 | |
| ZIC1 | chr3:147518581 | 2.22745E-06 | 1 | 1 | |
| STAT4 | chr2:191889978 | 2.19889E-06 | 1 | 1 | |
| FRK | chr6:116313575 | 2.19889E-06 | 1 | 1 | |
| EPB41L2 | chr6:131242649 | 2.19711E-06 | 1 | 1 | |
| SPCS3 | chr4:177306013 | 2.17567E-06 | 1 | 1 | |
| PP12613 | chr4:122686566 | 2.16684E-06 | 1 | 1 | |

| | | | | | |
|---|---|---|---|---|---|
| RHOT1 | chr17:30492942 | 2.13766E-06 | 1 | 1 | |
| CDH10 | chr5:24709731 | 2.13661E-06 | 1 | 1 | |
| COQ7 | chr16:19081549 | 2.11322E-06 | 1 | 1 | |
| FSTL5 | chr4:163161694 | 2.10431E-06 | 1 | 1 | |
| SUCNR1 | chr3:151719573 | 2.10065E-06 | 1 | 1 | |
| MYO16 | chr13:109977212 | 2.08193E-06 | 1 | 1 | |
| C11orf44 | chr11:130469123 | 2.0559E-06 | 1 | 1 | |
| CXCR4 | chr2:137203502 | 2.04531E-06 | 1 | 1 | |
| LOC147093 | chr17:39715456 | 2.04473E-06 | 1 | 1 | |
| SKP1P2 | chr12:17263656 | 2.03583E-06 | 1 | 1 | |
| DGKB | chr7:14378648 | 2.00703E-06 | 1 | 1 | |
| GRM5 | chr11:88265428 | 1.95828E-06 | 1 | 1 | |
| OR5H2 | chr3:98021510 | 1.90764E-06 | 1 | 1 | |
| NCAM2 | chr21:22452081 | 1.8883E-06 | 1 | 1 | |
| ZCCHC12 | chrX:118006903 | 1.88822E-06 | 1 | 1 | |
| LOC100129027 | chr21:47180697 | 1.87369E-06 | 1 | 1 | |
| COPZ2 | chr17:46100055 | 1.84687E-06 | 1 | 1 | |
| LINC00645 | chr14:27861781 | 1.84434E-06 | 1 | 1 | |
| SERGEF | chr11:17870754 | 1.80879E-06 | 1 | 1 | |
| ZRANB2-AS2 | chr1:71779924 | 1.77202E-06 | 1 | 1 | |
| FIGN | chr2:164781973 | 1.75615E-06 | 1 | 1 | |
| SMC2 | chr9:106603930 | 1.75031E-06 | 1 | 1 | |
| ANGPT1 | chr8:108317194 | 1.72553E-06 | 1 | 1 | |
| PHF21A | chr11:46014946 | 1.67301E-06 | 1 | 1 | |
| MBNL1-AS1 | chr3:151815287 | 1.67164E-06 | 1 | 1 | |
| LOC100302640 | chr3:106929962 | 1.64436E-06 | 1 | 1 | |
| OR4L1 | chr14:20521150 | 1.62908E-06 | 1 | 1 | |
| FRMD4A | chr10:14004157 | 1.56214E-06 | 1 | 1 | |
| SSFA2 | chr2:182798148 | 1.53962E-06 | 1 | 1 | |
| TFPI | chr2:188607567 | 1.51958E-06 | 1 | 1 | |
| TFAP2C | chr20:55470608 | 1.51958E-06 | 1 | 1 | |
| CNTN3 | chr3:74163157 | 1.51576E-06 | 1 | 1 | |
| CLLU1OS | chr12:92733157 | 1.51576E-06 | 1 | 1 | |
| C3orf38 | chr3:88676426 | 1.51118E-06 | 1 | 1 | |
| GTF2F2 | chr13:45807700 | 1.51108E-06 | 1 | 1 | |
| ALG13 | chrX:110987084 | 1.49159E-06 | 1 | 1 | |
| SRL | chr16:4267917 | 1.47144E-06 | 1 | 1 | |
| GRIA4 | chr11:105516929 | 1.45002E-06 | 1 | 1 | |
| TNFRSF11A | chr18:60065108 | 1.42164E-06 | 1 | 1 | |
| NXT1 | chr20:23332388 | 1.42013E-06 | 1 | 1 | |
| PAM | chr5:102270293 | 1.4089E-06 | 1 | 1 | |
| BMP8A | chr1:39962912 | 1.39438E-06 | 1 | 1 | |
| MIR890 | chrX:145023693 | 1.39081E-06 | 1 | 1 | |
| SMO | chr7:128851007 | 1.36058E-06 | 1 | 1 | |
| TSPAN11 | chr12:31136286 | 1.36033E-06 | 1 | 1 | |
| FBXW7 | chr4:153140767 | 1.33732E-06 | 1 | 1 | |
| RLTPR | chr16:67677128 | 1.33043E-06 | 1 | 1 | |
| LRRC8D | chr1:90378914 | 1.31765E-06 | 1 | 1 | |
| SATB2 | chr2:199710014 | 1.30471E-06 | 1 | 1 | |
| CNIH3 | chr1:224744698 | 1.28552E-06 | 1 | 1 | |
| LOC100130264 | chr20:19249747 | 1.26468E-06 | 1 | 1 | |
| TES | chr7:115871721 | 1.25439E-06 | 1 | 1 | |
| CRIM1 | chr2:36674895 | 1.22181E-06 | 1 | 1 | |
| MAPRE2 | chr18:32562730 | 1.1959E-06 | 1 | 1 | |
| DCBLD2 | chr3:98606532 | 1.16909E-06 | 1 | 1 | |
| CYTH1 | chr17:76708215 | 1.1187E-06 | 1 | 1 | |

| | | | | | |
|---|---|---|---|---|---|
| PKIA | chr8:78880836 | 1.10391E-06 | 1 | 1 | |
| ZNF680 | chr7:63991801 | 1.08936E-06 | 1 | 1 | |
| ARAP2 | chr4:34645314 | 1.08714E-06 | 1 | 1 | |
| SETD4 | chr21:37279613 | 1.08714E-06 | 1 | 1 | |
| DUT | chr15:48615763 | 1.08165E-06 | 1 | 1 | |
| ALG14 | chr1:95477920 | 1.07631E-06 | 1 | 1 | |
| APPBP2 | chr17:58611625 | 1.0683E-06 | 1 | 1 | |
| CECR2 | chr22:17888531 | 1.06199E-06 | 1 | 1 | |
| STAC3 | chr12:57638553 | 1.06136E-06 | 1 | 1 | |
| WWP2 | chr16:69900466 | 1.06136E-06 | 1 | 1 | |
| YTHDF3 | chr8:64137266 | 1.06104E-06 | 1 | 1 | |
| BBS9 | chr7:33341249 | 1.04815E-06 | 1 | 1 | |
| SCAF11 | chr12:46307948 | 1.04641E-06 | 1 | 1 | |
| DNAJC13 | chr3:132126926 | 1.03807E-06 | 1 | 1 | |
| HIST1H2BD | chr6:26174906 | 1.01817E-06 | 1 | 1 | |
| STEAP1 | chr7:89792015 | 9.87678E-07 | 1 | 1 | |
| PRKCQ-AS1 | chr10:6706518 | 9.77599E-07 | 1 | 1 | |
| SPATA17 | chr1:218046444 | 9.64584E-07 | 1 | 1 | |
| MIR147A | chr9:123042716 | 9.58634E-07 | 1 | 1 | |
| APOB | chr2:21239485 | 9.32432E-07 | 1 | 1 | |
| ASB17 | chr1:76460048 | 9.27208E-07 | 1 | 1 | |
| PRKAA1 | chr5:40784085 | 9.20969E-07 | 1 | 1 | |
| CDH13 | chr16:83466830 | 9.19887E-07 | 1 | 1 | |
| DCAF12 | chr9:34099112 | 9.00279E-07 | 1 | 1 | |
| ELP2 | chr18:33740884 | 8.84988E-07 | 1 | 1 | |
| EDIL3 | chr5:83710671 | 8.76816E-07 | 1 | 1 | |
| ROR2 | chr9:94486709 | 8.76816E-07 | 1 | 1 | |
| TXNDC8 | chr9:113113451 | 8.76816E-07 | 1 | 1 | |
| WIPF3 | chr7:29873355 | 8.46691E-07 | 1 | 1 | |
| GALNT2 | chr1:230002108 | 8.45656E-07 | 1 | 1 | |
| MLLT10 | chr10:21937422 | 7.86661E-07 | 1 | 1 | |
| LOC646329 | chr7:130584382 | 7.35719E-07 | 1 | 1 | |
| MLLT3 | chr9:20387541 | 7.26238E-07 | 1 | 1 | |
| TNFSF18 | chr1:172957318 | 7.15562E-07 | 1 | 1 | |
| PIEZO2 | chr18:11372112 | 7.07991E-07 | 1 | 1 | |
| PLXNA4 | chr7:132272386 | 7.0517E-07 | 1 | 1 | |
| MYO5A | chr15:52753989 | 6.96644E-07 | 1 | 1 | |
| TRIO | chr5:14314892 | 6.85927E-07 | 1 | 1 | |
| EMP1 | chr12:13356334 | 6.64491E-07 | 1 | 1 | |
| CD59 | chr11:33736302 | 6.43056E-07 | 1 | 1 | |
| TRIM29 | chr11:120032251 | 6.21621E-07 | 1 | 1 | |
| CNTNAP2 | chr7:146399353 | 6.1398E-07 | 1 | 1 | |
| BRIP1 | chr17:59916516 | 6.09659E-07 | 1 | 1 | |
| ZBTB8B | chr1:32895413 | 5.89992E-07 | 1 | 1 | |
| C3orf79 | chr3:153246449 | 5.89992E-07 | 1 | 1 | |
| SERTAD2 | chr2:64966153 | 5.89468E-07 | 1 | 1 | |
| NOVA1 | chr14:26421751 | 5.84544E-07 | 1 | 1 | |
| OR2K2 | chr9:113952519 | 5.64387E-07 | 1 | 1 | |
| FLJ26245 | chr16:35002116 | 5.54309E-07 | 1 | 1 | |
| ULK4 | chr3:41481089 | 5.44231E-07 | 1 | 1 | |
| MRPS30 | chr5:44892518 | 5.44231E-07 | 1 | 1 | |
| RBL2 | chr16:53507564 | 5.44231E-07 | 1 | 1 | |
| ATF6 | chr1:161887231 | 5.34152E-07 | 1 | 1 | |
| SMARCA2 | chr9:2028681 | 5.34152E-07 | 1 | 1 | |
| GCFC2 | chr2:75927564 | 5.22891E-07 | 1 | 1 | |
| BCHE | chr3:165510119 | 5.14445E-07 | 1 | 1 | |

| | | | | | |
|---|---|---|---|---|---|
| NFXL1 | chr4:47893050 | 5.14445E-07 | 1 | 1 | |
| SLC35F4 | chr14:58083207 | 5.14445E-07 | 1 | 1 | |
| ZNF692 | chr1:249166593 | 5.11327E-07 | 1 | 1 | |
| MAML3 | chr4:140680831 | 5.11327E-07 | 1 | 1 | |
| MGST2 | chr4:140621611 | 5.03693E-07 | 1 | 1 | |
| GLUD2 | chrX:121043501 | 4.93842E-07 | 1 | 1 | |
| BRAT1 | chr7:2578045 | 4.93839E-07 | 1 | 1 | |
| BNC2 | chr9:16707692 | 4.70113E-07 | 1 | 1 | |
| GPC5 | chr13:92790761 | 4.63604E-07 | 1 | 1 | |
| ALDH3A2 | chr17:19561329 | 4.53525E-07 | 1 | 1 | |
| NGFRAP1 | chrX:102631363 | 4.53525E-07 | 1 | 1 | |
| GPR176 | chr15:40197033 | 4.43447E-07 | 1 | 1 | |
| ADAMTS9-AS2 | chr3:64966071 | 4.36534E-07 | 1 | 1 | |
| CACNG7 | chr19:54442107 | 4.36534E-07 | 1 | 1 | |
| TCHH | chr1:152091980 | 4.21218E-07 | 1 | 1 | |
| ZNF660 | chr3:44649045 | 4.1813E-07 | 1 | 1 | |
| RNF217 | chr6:125400510 | 3.93055E-07 | 1 | 1 | |
| BANK1 | chr4:102720093 | 3.92169E-07 | 1 | 1 | |
| DAPK1 | chr9:90295717 | 3.82977E-07 | 1 | 1 | |
| ANP32E | chr1:150181372 | 3.69375E-07 | 1 | 1 | |
| LOC644838 | chr2:67526759 | 3.53995E-07 | 1 | 1 | |
| HEY2 | chr6:126072688 | 3.53995E-07 | 1 | 1 | |
| DIS3L | chr15:66594669 | 3.53995E-07 | 1 | 1 | |
| RBFOX1 | chr16:6601479 | 3.53995E-07 | 1 | 1 | |
| CHST6 | chr16:75515368 | 3.53995E-07 | 1 | 1 | |
| GNG7 | chr19:2551003 | 3.53995E-07 | 1 | 1 | |
| PACSIN2 | chr22:43353530 | 3.48594E-07 | 1 | 1 | |
| NBEA | chr13:35993950 | 3.42664E-07 | 1 | 1 | |
| ATP11B | chr3:182620326 | 3.34504E-07 | 1 | 1 | |
| DCUN1D1 | chr3:182674600 | 3.34504E-07 | 1 | 1 | |
| KCNN2 | chr5:113490124 | 3.34504E-07 | 1 | 1 | |
| CSNK1G3 | chr5:123149816 | 3.34504E-07 | 1 | 1 | |
| REV1 | chr2:100066619 | 3.34329E-07 | 1 | 1 | |
| APTX | chr9:33012126 | 3.34329E-07 | 1 | 1 | |
| ABI3 | chr17:47294176 | 3.32585E-07 | 1 | 1 | |
| PRPF18 | chr10:13654435 | 3.12429E-07 | 1 | 1 | |
| FBXL7 | chr5:15951751 | 3.02216E-07 | 1 | 1 | |
| DPP6 | chr7:154600699 | 2.94996E-07 | 1 | 1 | |
| IRAK1BP1 | chr6:79337562 | 2.82194E-07 | 1 | 1 | |
| ZBTB38 | chr3:141106744 | 2.7533E-07 | 1 | 1 | |
| EYA4 | chr6:133542316 | 2.61446E-07 | 1 | 1 | |
| PGA5 | chr11:61015430 | 2.55663E-07 | 1 | 1 | |
| NR2C2 | chr3:15079339 | 2.50878E-07 | 1 | 1 | |
| TRAM1L1 | chr4:118112967 | 2.50878E-07 | 1 | 1 | |
| KCNA7 | chr19:49577959 | 2.50878E-07 | 1 | 1 | |
| GPR85 | chr7:112700922 | 2.46921E-07 | 1 | 1 | |
| ZEB1 | chr10:31893698 | 2.35997E-07 | 1 | 1 | |
| NLRC4 | chr2:32493523 | 2.32396E-07 | 1 | 1 | |
| LOC728342 | chr5:117265406 | 2.32396E-07 | 1 | 1 | |
| UNC5D | chr8:34923228 | 2.32396E-07 | 1 | 1 | |
| GCNT2 | chr6:10600131 | 2.31802E-07 | 1 | 1 | |
| LACE1 | chr6:108854877 | 2.17871E-07 | 1 | 1 | |
| PAK6 | chr15:40527399 | 2.17871E-07 | 1 | 1 | |
| HPVC1 | chr7:54386578 | 2.16331E-07 | 1 | 1 | |
| TMPRSS6 | chr22:37480133 | 2.16331E-07 | 1 | 1 | |
| C22orf34 | chr22:49759550 | 2.16331E-07 | 1 | 1 | |

| | | | | | |
|---|---|---|---|---|---|
| UFM1 | chr13:38991048 | 2.03347E-07 | 1 | 1 | |
| NEGR1-IT1 | chr1:72300981 | 2.01567E-07 | 1 | 1 | |
| FMO2 | chr1:171163824 | 1.96664E-07 | 1 | 1 | |
| C1orf100 | chr1:244555854 | 1.96664E-07 | 1 | 1 | |
| MYADML | chr2:34294094 | 1.91489E-07 | 1 | 1 | |
| ROBO2 | chr3:76731822 | 1.88822E-07 | 1 | 1 | |
| VGLL3 | chr3:86955670 | 1.8141E-07 | 1 | 1 | |
| LDLRAD3 | chr11:36142205 | 1.74297E-07 | 1 | 1 | |
| ADAMTS18 | chr16:77431230 | 1.74297E-07 | 1 | 1 | |
| TNIK | chr3:170829874 | 1.71332E-07 | 1 | 1 | |
| MPHOSPH8 | chr13:20173814 | 1.71332E-07 | 1 | 1 | |
| ACTL8 | chr1:18244791 | 1.67252E-07 | 1 | 1 | |
| CD1C | chr1:158279409 | 1.67252E-07 | 1 | 1 | |
| IGSF8 | chr1:160076992 | 1.67252E-07 | 1 | 1 | |
| ARIH2 | chr3:48965176 | 1.67252E-07 | 1 | 1 | |
| MIR548I1 | chr3:125492167 | 1.67252E-07 | 1 | 1 | |
| PRR23A | chr3:138726573 | 1.67252E-07 | 1 | 1 | |
| GSX2 | chr4:54994283 | 1.67252E-07 | 1 | 1 | |
| LOC100144602 | chr4:66577791 | 1.67252E-07 | 1 | 1 | |
| LOC100505875 | chr4:80160771 | 1.67252E-07 | 1 | 1 | |
| IPO11 | chr5:61859972 | 1.67252E-07 | 1 | 1 | |
| MYOT | chr5:137195006 | 1.67252E-07 | 1 | 1 | |
| PPP1R2P3 | chr5:156240329 | 1.67252E-07 | 1 | 1 | |
| QKI | chr6:164844173 | 1.67252E-07 | 1 | 1 | |
| HGF | chr7:81318751 | 1.67252E-07 | 1 | 1 | |
| SEMA3E | chr7:83155731 | 1.67252E-07 | 1 | 1 | |
| GRM3 | chr7:85877827 | 1.67252E-07 | 1 | 1 | |
| DPYSL2 | chr8:26458936 | 1.67252E-07 | 1 | 1 | |
| FAM84B | chr8:127632268 | 1.67252E-07 | 1 | 1 | |
| ACER3 | chr11:76547704 | 1.67252E-07 | 1 | 1 | |
| PLBD1 | chr12:14739932 | 1.67252E-07 | 1 | 1 | |
| CRY1 | chr12:107552424 | 1.67252E-07 | 1 | 1 | |
| SLITRK6 | chr13:86529470 | 1.67252E-07 | 1 | 1 | |
| PRKCH | chr14:61774886 | 1.67252E-07 | 1 | 1 | |
| LOC283585 | chr14:87306396 | 1.67252E-07 | 1 | 1 | |
| MNS1 | chr15:56808385 | 1.67252E-07 | 1 | 1 | |
| MEF2A | chr15:100237202 | 1.67252E-07 | 1 | 1 | |
| AIPL1 | chr17:6189126 | 1.67252E-07 | 1 | 1 | |
| KCNG1 | chr20:49664120 | 1.67252E-07 | 1 | 1 | |
| ADARB1 | chr21:46664466 | 1.67252E-07 | 1 | 1 | |
| CXorf57 | chrX:105815497 | 1.67252E-07 | 1 | 1 | |
| ASCC3 | chr6:101360583 | 1.61254E-07 | 1 | 1 | |
| UCP2 | chr11:73692233 | 1.61254E-07 | 1 | 1 | |
| MIR548A3 | chr3:103225119 | 1.45248E-07 | 1 | 1 | |
| SNX2 | chr5:122120746 | 1.45248E-07 | 1 | 1 | |
| ITFG3 | chr16:283517 | 1.45248E-07 | 1 | 1 | |

**Table S2. List of resistant clones.**

Clones A1-A23 were isolated from screened plate and B1-B201 were from limiting dilution of resistant pools. Each insert was ann
orientation. A negative distance value indicates insert upstream of gene, positive value for downstream, and 0 for intragenic. CM
antisense of the host gene body.

| clone | insert | insert | insert |
|---|---|---|---|
| A1 | chrX:138684373 MCF2 0/+ | chr17:39095514 KRT23 -1679/+ | chr18:25449894 CDH2 81035/- |
| A2 | chr4:186742830 SORBS2 0/- | chr14:55490722 WDHD1 0/+ | chr12:69246594 CPM 0/+ |
| A3 | chr2:203664266 ICA1L 0/- | chr11:16146421 SOX6 0/- | chr11:113652668 CLDN25 1462/+ |
| A4 | chr5:109326726 LOC100289673 10552 | chr2:190016957 COL5A2 0/+ | chr10:63732986 ARID5B 0/+ |
| A5 | chr18:24786338 CHST9 -21037/+ | chr13:82153549 SPRY2 -1238464/+ | |
| A6 | chr2:220825933 MIR4268 -54648/- | chr5:122026084 SNX2 -84665/- | chr9:99951467 LOC100499484 -49240/ |
| A7 | chr2:83256616 LOC1720 171724/- | chr1:51138260 FAF1 0/- | chr3:187823607 LPP-AS2 45386/- |
| A8 | chr13:94939649 GPC6 0/- | chr2:39231446 SOS1 0/+ | |
| A9 | chr13:94939649 GPC6 0/- | | |
| A10 | chr13:94939649 GPC6 0/- | | |
| A11 | chr13:94939649 GPC6 0/- | | |
| A12 | chr13:94939649 GPC6 0/- | | |
| A13 | chr13:94939649 GPC6 0/- | | |
| A14 | chr13:94939649 GPC6 0/- | | |
| A15 | chr13:94939649 GPC6 0/- | chr9:108030042 SLC44A1 0/- | |
| A16 | chrX:115045927 PLS3 160749/- | chr7:140460815 BRAF 0/+ | |
| A17 | chrX:115045927 PLS3 160749/- | chr7:140460815 BRAF 0/+ | |
| A18 | chrX:115045927 PLS3 160749/- | chr7:140460815 BRAF 0/+ | chr14:56156632 KTN1 5331/- |
| A19 | chrX:115045927 PLS3 160749/- | chr7:140460815 BRAF 0/+ | |
| A20 | chrX:115045927 PLS3 160749/- | chr7:140460815 BRAF 0/+ | chr8:68050488 CSPP1 0/- |
| A21 | chrX:115045927 PLS3 160749/- | chr7:140460815 BRAF 0/+ | chr7:114567455 MDFIC 0/- |
| A22 | chrX:115045927 PLS3 160749/- | chr7:140460815 BRAF 0/+ | chr14:56156632 KTN1 5331/- |
| A23 | chrX:115045927 PLS3 160749/- | chr7:140460815 BRAF 0/+ | chr14:56156632 KTN1 5331/- |
| B1 | chr10:35416711 CREM 0/- | | |
| B2 | chr13:94939649 GPC6 0/- | | |
| B3 | chr7:140460815 BRAF 0/+ | chrX:115045927 PLS3 160749/- | chr14:56156632 KTN1 5331/- |
| B4 | chr7:140483217 BRAF 0/+ | | |
| B5 | chr7:140460815 BRAF 0/+ | chrX:115045927 PLS3 160749/- | chr14:56156632 KTN1 5331/- |
| B6 | chr13:94939649 GPC6 0/- | | |
| B7 | chr7:140460815 BRAF 0/+ | chrX:115045927 PLS3 160749/- | |
| B8 | chr13:94939649 GPC6 0/- | | |
| B9 | chr7:140460815 BRAF 0/+ | chrX:115045927 PLS3 160749/- | chr14:56156632 KTN1 5331/- |
| B10 | chr7:140460815 BRAF 0/+ | chrX:115045927 PLS3 160749/- | |
| B11 | chr13:94939649 GPC6 0/- | chr1:170751740 PRRX1 43200/+ | |
| B12 | chr7:140460815 BRAF 0/+ | chrX:115045927 PLS3 160749/- | chr14:56156632 KTN1 5331/- |
| B13 | chr13:94939649 GPC6 0/- | chr1:170751740 PRRX1 43200/+ | |
| B14 | chr7:140460815 BRAF 0/+ | chrX:115045927 PLS3 160749/- | |
| B15 | chr7:140460815 BRAF 0/+ | chrX:115045927 PLS3 160749/- | |
| B16 | chr7:140460815 BRAF 0/+ | chrX:115045927 PLS3 160749/- | |
| B17 | chr7:140460815 BRAF 0/+ | chrX:115045927 PLS3 160749/- | |
| B18 | chr13:94939649 GPC6 0/- | chr1:170751740 PRRX1 43200/+ | |
| B19 | chr13:94939649 GPC6 0/- | | |
| B20 | chr13:94939649 GPC6 0/- | | |
| B21 | chr7:140460815 BRAF 0/+ | chrX:115045927 PLS3 160749/- | |
| B22 | chr7:140460815 BRAF 0/+ | chrX:115045927 PLS3 160749/- | |
| B23 | chr7:140483217 BRAF 0/+ | | |
| B24 | chr13:94939649 GPC6 0/- | | |
| B25 | chr13:94939649 GPC6 0/- | | |

| | | | |
|---|---|---|---|
| B26 | chr7:140483217 BRAF 0/+ | | |
| B27 | chr13:94939649 GPC6 0/- | chr1:170751740 PRRX1 43200/+ | |
| B28 | chr7:140460815 BRAF 0/+ | chrX:115045927 PLS3 160749/- | chr14:56156632 KTN1 5331/- |
| B29 | chr7:140460815 BRAF 0/+ | chrX:115045927 PLS3 160749/- | |
| B30 | chr7:140460815 BRAF 0/+ | chrX:115045927 PLS3 160749/- | |
| B31 | chr7:140483217 BRAF 0/+ | | |
| B32 | chr7:140460815 BRAF 0/+ | chrX:115045927 PLS3 160749/- | chr14:56156632 KTN1 5331/- |
| B33 | chr7:140460815 BRAF 0/+ | chrX:115045927 PLS3 160749/- | |
| B34 | chr13:94939649 GPC6 0/- | | |
| B35 | chr7:140460815 BRAF 0/+ | chrX:115045927 PLS3 160749/- | |
| B36 | chr13:94939649 GPC6 0/- | | |
| B37 | chr13:94939649 GPC6 0/- | | |
| B38 | chr7:140460815 BRAF 0/+ | chrX:115045927 PLS3 160749/- | chr14:56156632 KTN1 5331/- |
| B39 | chr7:140460815 BRAF 0/+ | chrX:115045927 PLS3 160749/- | |
| B40 | chr13:94939649 GPC6 0/- | | |
| B41 | chr13:94939649 GPC6 0/- | chr1:170751740 PRRX1 43200/+ | |
| B42 | chr7:140460815 BRAF 0/+ | chrX:115045927 PLS3 160749/- | chr14:56156632 KTN1 5331/- |
| B43 | chrX:115044473 PLS3 159295/- | | |
| B44 | chr13:94939649 GPC6 0/- | | |
| B45 | chr13:94939649 GPC6 0/- | | |
| B46 | chr7:140460815 BRAF 0/+ | chrX:115045927 PLS3 160749/- | chr14:56156632 KTN1 5331/- |
| B47 | chr7:140460815 BRAF 0/+ | chrX:115045927 PLS3 160749/- | |
| B48 | chr7:140460815 BRAF 0/+ | chrX:115045927 PLS3 160749/- | chr14:56156632 KTN1 5331/- |
| B49 | chr13:94939649 GPC6 0/- | chr1:170751740 PRRX1 43200/+ | |
| B50 | chr13:94939649 GPC6 0/- | chr1:170751740 PRRX1 43200/+ | |
| B51 | chr7:140460815 BRAF 0/+ | chrX:115045927 PLS3 160749/- | |
| B52 | chr13:94939649 GPC6 0/- | chr1:170751740 PRRX1 43200/+ | |
| B53 | chr13:94939649 GPC6 0/- | | |
| B54 | chr7:140483217 BRAF 0/+ | | |
| B55 | chr7:140460815 BRAF 0/+ | chrX:115045927 PLS3 160749/- | chr14:56156632 KTN1 5331/- |
| B56 | chr13:94939649 GPC6 0/- | | |
| B57 | chr7:140460815 BRAF 0/+ | chrX:115045927 PLS3 160749/- | chr14:56156632 KTN1 5331/- |
| B58 | chr7:140460815 BRAF 0/+ | chrX:115045927 PLS3 160749/- | |
| B59 | chr7:140460815 BRAF 0/+ | chrX:115045927 PLS3 160749/- | chr14:56156632 KTN1 5331/- |
| B60 | chr13:94939649 GPC6 0/- | | |
| B61 | chr7:140460815 BRAF 0/+ | chrX:115045927 PLS3 160749/- | |
| B62 | chr7:140483217 BRAF 0/+ | | |
| B63 | chr7:140460815 BRAF 0/+ | chrX:115045927 PLS3 160749/- | |
| B64 | chr7:140483217 BRAF 0/+ | | |
| B65 | chr13:94939649 GPC6 0/- | | |
| B66 | chr7:140483217 BRAF 0/+ | chr13:94939649 GPC6 0/- | |
| B67 | chr7:140460815 BRAF 0/+ | chrX:115045927 PLS3 160749/- | |
| B68 | chr7:140460815 BRAF 0/+ | chrX:115045927 PLS3 160749/- | chr14:56156632 KTN1 5331/- |
| B69 | chr7:140460815 BRAF 0/+ | chrX:115045927 PLS3 160749/- | chr14:56156632 KTN1 5331/- |
| B70 | chr7:140460815 BRAF 0/+ | chrX:115045927 PLS3 160749/- | |
| B71 | chr7:140460815 BRAF 0/+ | chrX:115045927 PLS3 160749/- | chr14:56156632 KTN1 5331/- |
| B72 | chr13:94939649 GPC6 0/- | | |
| B73 | chr7:140483217 BRAF 0/+ | chr13:94939649 GPC6 0/- | |
| B74 | chr13:94939649 GPC6 0/- | | |
| B75 | chr7:140460815 BRAF 0/+ | chrX:115044473 PLS3 159295/- | |
| B76 | chr7:140483217 BRAF 0/+ | | |
| B77 | chr13:94939649 GPC6 0/- | chr1:170751740 PRRX1 43200/+ | |
| B78 | chr7:140460815 BRAF 0/+ | chrX:115045927 PLS3 160749/- | |

| | | | |
|---|---|---|---|
| B79 | chr7:140460815 BRAF 0/+ | chrX:115045927 PLS3 160749/- | |
| B80 | chr7:140460815 BRAF 0/+ | chrX:115045927 PLS3 160749/- | |
| B81 | chr7:140460815 BRAF 0/+ | chrX:115045927 PLS3 160749/- | chr14:56156632 KTN1 5331/- |
| B82 | chr7:140460815 BRAF 0/+ | chrX:115045927 PLS3 160749/- | |
| B83 | chr7:140460815 BRAF 0/+ | chrX:115045927 PLS3 160749/- | |
| B84 | chr7:140460815 BRAF 0/+ | chrX:115045927 PLS3 160749/- | chr14:56156632 KTN1 5331/- |
| B85 | chr7:140460815 BRAF 0/+ | chrX:115045927 PLS3 160749/- | |
| B86 | chr7:140460815 BRAF 0/+ | chrX:115045927 PLS3 160749/- | chr14:56156632 KTN1 5331/- |
| B87 | chr7:140460815 BRAF 0/+ | chrX:115045927 PLS3 160749/- | |
| B88 | chr2:68244025 C1D 25306/+ | | |
| B89 | chr7:140460815 BRAF 0/+ | chrX:115045927 PLS3 160749/- | chr14:56156632 KTN1 5331/- |
| B90 | chr7:140483217 BRAF 0/+ | | |
| B91 | chr13:94939649 GPC6 0/- | | |
| B92 | chr7:140460815 BRAF 0/+ | chrX:115045927 PLS3 160749/- | |
| B93 | chr7:140483217 BRAF 0/+ | | |
| B94 | chr13:94939649 GPC6 0/- | | |
| B95 | chr7:140460815 BRAF 0/+ | chrX:115045927 PLS3 160749/- | |
| B96 | chr7:140483217 BRAF 0/+ | | |
| B97 | chr7:140460815 BRAF 0/+ | chrX:115045927 PLS3 160749/- | |
| B98 | chr7:140460815 BRAF 0/+ | chrX:115045927 PLS3 160749/- | |
| B99 | chr7:140460815 BRAF 0/+ | chrX:115045927 PLS3 160749/- | |
| B100 | chr13:94939649 GPC6 0/- | | |
| B101 | chr7:140460815 BRAF 0/+ | chrX:115045927 PLS3 160749/- | chr13:94939649 GPC6 0/- |
| B102 | chr7:140460815 BRAF 0/+ | chrX:115045927 PLS3 160749/- | |
| B103 | chr13:94939649 GPC6 0/- | | |
| B104 | chr7:140460815 BRAF 0/+ | chrX:115045927 PLS3 160749/- | |
| B105 | chr13:94939649 GPC6 0/- | | |
| B106 | chr7:140460815 BRAF 0/+ | chrX:115045927 PLS3 160749/- | |
| B107 | chr7:140460815 BRAF 0/+ | chrX:115045927 PLS3 160749/- | |
| B108 | chr7:140460815 BRAF 0/+ | chrX:115045927 PLS3 160749/- | chr14:56156632 KTN1 5331/- |
| B109 | chr7:140460815 BRAF 0/+ | chrX:115045927 PLS3 160749/- | |
| B110 | chr7:140460815 BRAF 0/+ | chrX:115045927 PLS3 160749/- | chr7:140483217 BRAF 0/+ |
| B111 | chr7:140460815 BRAF 0/+ | chrX:115045927 PLS3 160749/- | chr7:140483217 BRAF 0/+ |
| B112 | chr7:140460815 BRAF 0/+ | chrX:115045927 PLS3 160749/- | |
| B113 | chr7:140460815 BRAF 0/+ | chrX:115045927 PLS3 160749/- | |
| B114 | chr7:140483217 BRAF 0/+ | | |
| B115 | chr13:94939649 GPC6 0/- | | |
| B116 | chr7:140460815 BRAF 0/+ | chrX:115045927 PLS3 160749/- | chr14:56156632 KTN1 5331/- |
| B117 | chr7:140460815 BRAF 0/+ | chrX:115045927 PLS3 160749/- | |
| B118 | chr7:140460815 BRAF 0/+ | chrX:115045927 PLS3 160749/- | |
| B119 | chr7:140460815 BRAF 0/+ | chrX:115045927 PLS3 160749/- | |
| B120 | chr21:38722375 DYRK1A -17483/- | chr19:16137669 LINC00661 0/+ | chr8:114360638 CSMD3 0/- |
| B121 | chr7:140460815 BRAF 0/+ | chrX:115045927 PLS3 160749/- | |
| B122 | chr7:140483217 BRAF 0/+ | chr13:94939649 GPC6 0/- | |
| B123 | chr7:140460815 BRAF 0/+ | chrX:115045927 PLS3 160749/- | |
| B124 | chr7:140460815 BRAF 0/+ | chrX:115045927 PLS3 160749/- | chr7:140483217 BRAF 0/+ |
| B125 | chr7:140460815 BRAF 0/+ | | |
| B126 | chr7:140460815 BRAF 0/+ | chrX:115045927 PLS3 160749/- | chr12:56345588 DGKA 0/- |
| B127 | chr7:140460815 BRAF 0/+ | | |
| B128 | chr3:178840783 PIK3CA -25527/+ | chr1:214688471 PTPN14 0/+ | chr3:178500378 KCNMB2 0/+ |
| B129 | chr13:94939649 GPC6 0/- | | |
| B130 | chr7:140460815 BRAF 0/+ | chrX:115045927 PLS3 160749/- | chr7:140483217 BRAF 0/+ |
| B131 | chr7:140460815 BRAF 0/+ | chrX:115045927 PLS3 160749/- | chr14:56156632 KTN1 5331/- |

| | | | |
|---|---|---|---|
| B132 | chr21:38722375 DYRK1A -17483/- | chr19:16137669 LINC00661 0/+ | chr8:114360638 CSMD3 0/- |
| B133 | chr7:140460815 BRAF 0/+ | chrX:115045927 PLS3 160749/- | chr2:15098373 NBAS 208658/- |
| B134 | chr7:140483217 BRAF 0/+ | | |
| B135 | chr7:140460815 BRAF 0/+ | chrX:115045927 PLS3 160749/- | chr13:94939649 GPC6 0/- |
| B136 | chr7:140460815 BRAF 0/+ | chrX:115045927 PLS3 160749/- | |
| B137 | chr10:35416711 CREM 0/- | | |
| B138 | chr13:94939649 GPC6 0/- | chr1:170751740 PRRX1 43200/+ | |
| B139 | chr7:140460815 BRAF 0/+ | chrX:115045927 PLS3 160749/- | chr14:56156632 KTN1 5331/- |
| B140 | chr13:94939649 GPC6 0/- | | |
| B141 | chr13:94939649 GPC6 0/- | chr1:170751740 PRRX1 43200/+ | chr3:12621903 MKRN2 0/- |
| B142 | chr12:91548330 DCN 0/- | chr12:78560659 NAV3 0/+ | |
| B143 | chr7:140460815 BRAF 0/+ | chrX:115045927 PLS3 160749/- | chr14:56156632 KTN1 5331/- |
| B144 | chrX:115045927 PLS3 160749/- | chr14:56156632 KTN1 5331/- | |
| B145 | chr7:140460815 BRAF 0/+ | chrX:115045927 PLS3 160749/- | chr14:56156632 KTN1 5331/- |
| B146 | chr13:94939649 GPC6 0/- | | |
| B147 | chr7:140460815 BRAF 0/+ | chrX:115045927 PLS3 160749/- | chr14:56156632 KTN1 5331/- |
| B148 | chr12:33996081 ALG10 -179134/- | chr4:99319442 RAP1GDS1 0/- | |
| B149 | chr7:140460815 BRAF 0/+ | chrX:115045927 PLS3 160749/- | |
| B150 | chr7:140460815 BRAF 0/+ | chrX:115045927 PLS3 160749/- | chr2:68244025 C1D 25306/+ |
| B151 | chr7:140460815 BRAF 0/+ | chrX:115045927 PLS3 160749/- | chr13:94939649 GPC6 0/- |
| B152 | chr7:140483217 BRAF 0/+ | chr13:94939649 GPC6 0/- | chr12:91548330 DCN 0/- |
| B153 | chr7:140460815 BRAF 0/+ | chrX:115045927 PLS3 160749/- | |
| B154 | chr7:140460815 BRAF 0/+ | chrX:115045927 PLS3 160749/- | |
| B155 | chrX:115045927 PLS3 160749/- | chr14:56156632 KTN1 5331/- | chr7:140483217 BRAF 0/+ |
| B156 | chr13:94939649 GPC6 0/- | | |
| B157 | chr12:91548330 DCN 0/- | | |
| B158 | chr7:140460815 BRAF 0/+ | chrX:115045927 PLS3 160749/- | chr13:94939649 GPC6 0/- |
| B159 | chr7:140460815 BRAF 0/+ | chrX:115045927 PLS3 160749/- | |
| B160 | chr7:140460815 BRAF 0/+ | chrX:115045927 PLS3 160749/- | |
| B161 | chr7:140460815 BRAF 0/+ | chrX:115045927 PLS3 160749/- | |
| B162 | chr7:140483217 BRAF 0/+ | | |
| B163 | chr7:140460815 BRAF 0/+ | chrX:115045927 PLS3 160749/- | chr14:56156632 KTN1 5331/- |
| B164 | chr7:140460815 BRAF 0/+ | chrX:115045927 PLS3 160749/- | |
| B165 | chrX:115045927 PLS3 160749/- | chr7:140483217 BRAF 0/+ | |
| B166 | chrX:115045927 PLS3 160749/- | chr14:56156632 KTN1 5331/- | |
| B167 | chr7:140483217 BRAF 0/+ | chr13:94939649 GPC6 0/- | |
| B168 | chr7:140460815 BRAF 0/+ | chrX:115045927 PLS3 160749/- | |
| B169 | chr7:140460815 BRAF 0/+ | chrX:115045927 PLS3 160749/- | |
| B170 | chr7:140460815 BRAF 0/+ | chrX:115045927 PLS3 160749/- | chr14:56156632 KTN1 5331/- |
| B171 | chr13:94939649 GPC6 0/- | | |
| B172 | chr7:140460815 BRAF 0/+ | chrX:115045927 PLS3 160749/- | chr14:56156632 KTN1 5331/- |
| B173 | chr7:140460815 BRAF 0/+ | chrX:115045927 PLS3 160749/- | chr14:56156632 KTN1 5331/- |
| B174 | chr7:140460815 BRAF 0/+ | chrX:115045927 PLS3 160749/- | |
| B175 | chr7:140460815 BRAF 0/+ | chrX:115045927 PLS3 160749/- | |
| B176 | chr7:140460815 BRAF 0/+ | chrX:115045927 PLS3 160749/- | chr14:56156632 KTN1 5331/- |
| B177 | chr13:94939649 GPC6 0/- | | |
| B178 | chr7:140460815 BRAF 0/+ | chrX:115045927 PLS3 160749/- | chr14:56156632 KTN1 5331/- |
| B179 | chr7:140483217 BRAF 0/+ | | |
| B180 | chr7:140460815 BRAF 0/+ | chrX:115045927 PLS3 160749/- | chr13:94939649 GPC6 0/- |
| B181 | chr13:94939649 GPC6 0/- | chr18:55917356 NEDD4L 0/+ | |
| B182 | chr7:140460815 BRAF 0/+ | chrX:115045927 PLS3 160749/- | chr13:94939649 GPC6 0/- |
| B183 | chrX:115045927 PLS3 160749/- | chr7:140483217 BRAF 0/+ | |
| B184 | chr7:140460815 BRAF 0/+ | chrX:115045927 PLS3 160749/- | chr14:56156632 KTN1 5331/- |

| | | | |
|---|---|---|---|
| B185 | chr7:140460815 BRAF 0/+ | chrX:115045927 PLS3 160749/- | |
| B186 | chr7:140460815 BRAF 0/+ | chrX:115045927 PLS3 160749/- | |
| B187 | chr7:140460815 BRAF 0/+ | chrX:115045927 PLS3 160749/- | |
| B188 | chr7:140460815 BRAF 0/+ | chrX:115045927 PLS3 160749/- | |
| B189 | chr7:140460815 BRAF 0/+ | chrX:115045927 PLS3 160749/- | |
| B190 | chr7:140483217 BRAF 0/+ | | |
| B191 | chrX:115044473 PLS3 159295/- | | |
| B192 | chr12:91548330 DCN 0/- | | |
| B193 | chr7:140460815 BRAF 0/+ | chrX:115045927 PLS3 160749/- | |
| B194 | chr7:140460815 BRAF 0/+ | chrX:115045927 PLS3 160749/- | |
| B195 | chr6:11399655 NEDD9 -17075/+ | | |
| B196 | chrX:115045927 PLS3 160749/- | | |
| B197 | chr7:140460815 BRAF 0/+ | | |
| B198 | chr13:94939649 GPC6 0/- | chr1:170751740 PRRX1 43200/+ | chr3:12621903 MKRN2 0/- |
| B199 | chr7:140460815 BRAF 0/+ | chrX:115045927 PLS3 160749/- | chrX:115044473 PLS3 159295/- |
| B200 | chr7:140460815 BRAF 0/+ | chrX:115045927 PLS3 160749/- | |
| B201 | chrX:115045927 PLS3 160749/- | | |

ɔtated for genomic position, proximal gene, and relative distance and
/ promoter orientations are denoted as "+" for sense strand and "-" for

| insert | insert | insert |
|---|---|---|
| | | |
| chr13:93619495 GPC5 100009/- | chr5:166641588 TENM2 -70254/- | chr1:93278561 RPL5 -19032/- |
| | | |
| chr1:7335071 CAMTA1 0/- | chr9:5932584 KIAA2026 0/- | |
| | | |
| chr5:139358867 NRG2 0/- | | |
| | | |
| | | |
| | | |
| | | |
| | | |
| | | |
| | | |
| | | |
| | | |
| | | |
| | | |
| | | |
| | | |
| chr14:56156632 KTN1 5331/- | chr5:59456342 PDE4D 0/- | chr6:155662756 TFB1M -27140/- |
| | | |
| chr7:19932078 TMEM196 -119675/- | chr17:67546369 MAP2K6 7900/- | chrX:101909170 ARMCX5-GPRASP2 0/- |
| | | |
| | | |
| | | |
| | | |
| | | |
| | | |
| | | |
| | | |
| | | |
| | | |
| | | |
| | | |
| | | |
| | | |
| | | |
| | | |
| | | |
| | | |
| | | |
| | | |
| | | |
| | | |
| | | |
| | | |
| | | |
| | | |

| | | |
|---|---|---|
| | | |
| | | |
| | | |
| | | |
| | | |
| | | |
| | | |
| | | |
| | | |
| | | |
| | | |
| | | |
| | | |
| | | |
| | | |
| | | |
| | | |
| | | |
| | | |
| | | |
| | | |
| | | |
| | | |
| | | |
| | | |
| | | |
| | | |
| | | |
| | | |
| | | |
| | | |
| | | |
| | | |
| | | |
| | | |
| | | |
| | | |
| | | |
| | | |
| | | |
| | | |
| | | |
| | | |
| | | |
| chr13:94939649 GPC6 0/- | | |
| | | |
| | | |
| | | |
| | | |
| | | |
| | | |
| | | |

| | | |
|---|---|---|
| | | |
| | | |
| | | |
| | | |
| | | |
| chr1:44585386 KLF17 0/+ | | |
| | | |
| | | |
| | | |
| | | |
| | | |
| | | |
| | | |
| | | |
| | | |
| | | |
| | | |
| | | |
| | | |
| | | |
| | | |
| | | |
| | | |
| | | |
| | | |
| | | |
| | | |
| | | |
| | | |
| | | |
| | | |
| | | |
| chr13:94939649 GPC6 0/- | | |
| | | |
| | | |
| | | |
| | | |
| | | |
| | | |
| | | |
| | | |
| chr5:116549795 LOC728342 -201412/+ | chr5:37169570 C5orf42 0/- | |
| | | |
| | | |
| | | |
| | | |
| | | |
| | | |
| | | |
| chr12:90497671 LOC338758 391943/+ | | |
| | | |
| | | |
| chr2:68244025 C1D 25306/+ | | |

| | | |
|---|---|---|
| chr5:116549795 LOC728342 -201412/+ | chr5:37169570 C5orf42 0/- | |
| | | |
| | | |
| chr1:170751740 PRRX1 43200/+ | | |
| | | |
| | | |
| | | |
| | | |
| | | |
| | | |
| | | |
| | | |
| | | |
| | | |
| | | |
| | | |
| | | |
| | | |
| | | |
| | | |
| | | |
| | | |
| | | |
| chr13:94939649 GPC6 0/- | | |
| | | |
| | | |
| | | |
| | | |
| | | |
| | | |
| | | |
| chr3:12676395 RAF1 0/+ | | |
| | | |
| | | |
| | | |
| | | |
| | | |
| | | |
| | | |
| | | |
| | | |
| chr13:94939649 GPC6 0/- | | |
| | | |
| | | |
| chr8:78880836 PKIA -547499/+ | | |
| | | |
| | | |
| | | |
| | | |
| | | |
| chr11:34343365 ABTB2 0/- | | |
| | | |
| chr7:140483217 BRAF 0/+ | chr3:12676395 RAF1 0/+ | |

| | | |
|---|---|---|
| | | |
| | | |
| | | |
| | | |
| | | |
| | | |
| | | |
| | | |
| | | |
| | | |
| | | |
| | | |
| | | |
| | | |
| | | |
| | | |

| insert | insert | insert |
|---|---|---|
| | | |
| chr1:156395700 C1orf61 0/+ | chrX:9631982 TBL1X 0/- | chr7:80985100 HGF 346343/- |
| | | |
| | | |
| | | |
| | | |
| | | |
| | | |
| | | |
| | | |
| | | |
| | | |
| | | |
| | | |
| | | |
| | | |
| | | |
| | | |
| chr6:53324604 GCLC 37535/- | chr3:17258017 TBC1D5 0/- | chr3:190037300 CLDN1 0/+ |
| | | |
| chr7:131287901 PODXL -46526/+ | chr10:122600003 WDR11-AS1 0/- | |
| | | |
| | | |
| | | |
| | | |
| | | |
| | | |
| | | |
| | | |
| | | |
| | | |
| | | |
| | | |
| | | |
| | | |
| | | |
| | | |
| | | |
| | | |
| | | |
| | | |
| | | |
| | | |
| | | |
| | | |
| | | |
| | | |

| | | |
|---|---|---|
| | | |
| | | |
| | | |
| | | |
| | | |
| | | |
| | | |
| | | |
| | | |
| | | |
| | | |
| | | |
| | | |
| | | |
| | | |
| | | |
| | | |
| | | |
| | | |
| | | |
| | | |
| | | |
| | | |
| | | |
| | | |
| | | |
| | | |
| | | |
| | | |
| | | |
| | | |
| | | |
| | | |
| | | |
| | | |
| | | |
| | | |
| | | |
| | | |
| | | |
| | | |
| | | |
| | | |
| | | |
| | | |
| | | |
| | | |
| | | |
| | | |
| | | |
| | | |
| | | |

| | | |
|---|---|---|
| | | |
| | | |
| | | |
| | | |
| | | |
| | | |
| | | |
| | | |
| | | |
| | | |
| | | |
| | | |
| | | |
| | | |
| | | |
| | | |
| | | |
| | | |
| | | |
| | | |
| | | |
| | | |
| | | |
| | | |
| | | |
| | | |
| | | |
| | | |
| | | |
| | | |
| | | |
| | | |
| | | |
| | | |
| | | |
| | | |
| | | |
| | | |
| | | |
| | | |
| | | |
| | | |
| | | |
| | | |
| | | |
| | | |
| | | |
| | | |
| | | |
| | | |
| | | |
| | | |

| | | |
|---|---|---|
| | | |
| | | |
| | | |
| | | |
| | | |
| | | |
| | | |
| | | |
| | | |
| | | |
| | | |
| | | |
| | | |
| | | |
| | | |
| | | |

| insert | insert | insert |
|---|---|---|
| | | |
| chr10:6812753 LINC00707 -8806/- | | |
| | | |
| | | |
| | | |
| | | |
| | | |
| | | |
| | | |
| | | |
| | | |
| | | |
| | | |
| | | |
| | | |
| | | |
| | | |
| | | |
| | | |
| chr18:55909557 NEDD4L 0/+ | chr6:101718136 GRIK2 -128724/+ | chr11:134412361 LOC283177 36807/- |
| | | |
| | | |
| | | |
| | | |
| | | |
| | | |
| | | |
| | | |
| | | |
| | | |
| | | |
| | | |
| | | |
| | | |
| | | |
| | | |
| | | |
| | | |
| | | |
| | | |
| | | |
| | | |
| | | |
| | | |
| | | |
| | | |
| | | |
| | | |

| | | |
|---|---|---|
| | | |
| | | |
| | | |
| | | |
| | | |
| | | |
| | | |
| | | |
| | | |
| | | |
| | | |
| | | |
| | | |
| | | |
| | | |
| | | |
| | | |
| | | |
| | | |
| | | |
| | | |
| | | |
| | | |
| | | |
| | | |
| | | |
| | | |
| | | |
| | | |
| | | |
| | | |
| | | |
| | | |
| | | |
| | | |
| | | |
| | | |
| | | |
| | | |
| | | |
| | | |
| | | |
| | | |
| | | |
| | | |
| | | |
| | | |
| | | |
| | | |
| | | |
| | | |
| | | |

| | | |
|---|---|---|
| | | |
| | | |
| | | |
| | | |
| | | |
| | | |
| | | |
| | | |
| | | |
| | | |
| | | |
| | | |
| | | |
| | | |
| | | |
| | | |
| | | |
| | | |
| | | |
| | | |
| | | |
| | | |
| | | |
| | | |
| | | |
| | | |
| | | |
| | | |
| | | |
| | | |
| | | |
| | | |
| | | |
| | | |
| | | |
| | | |
| | | |
| | | |
| | | |
| | | |
| | | |
| | | |
| | | |
| | | |
| | | |
| | | |
| | | |
| | | |
| | | |
| | | |
| | | |
| | | |

| | | |
|---|---|---|
| | | |
| | | |
| | | |
| | | |
| | | |
| | | |
| | | |
| | | |
| | | |
| | | |
| | | |
| | | |
| | | |
| | | |
| | | |
| | | |
| | | |
| | | |
| | | |
| | | |
| | | |
| | | |
| | | |
| | | |
| | | |
| | | |
| | | |
| | | |
| | | |
| | | |
| | | |
| | | |
| | | |
| | | |
| | | |
| | | |
| | | |
| | | |
| | | |
| | | |
| | | |
| | | |
| | | |
| | | |
| | | |
| | | |
| | | |
| | | |
| | | |
| | | |
| | | |
| | | |

| | | |
|---|---|---|
| | | |
| | | |
| | | |
| | | |
| | | |
| | | |
| | | |
| | | |
| | | |
| | | |
| | | |
| | | |
| | | |
| | | |
| | | |
| | | |

**Table S3. Gene expression proximal to transposon inserts in 10 clones.**

Listed are clone ID, insert position, gene name, distance between insert and gene body (negative value for upstream, 0 for intragenic, and
the parental A375 control (RNA ratio), p- and q-values (Pval, Qval) for the three nearest genes. Ratio is capped at 50 and marked with an a

| | | first gene | | | | | | | | |
|---|---|---|---|---|---|---|---|---|---|---|
| clone | position | name | distance | strand | RPKM | RNA ratio | Pval | Qval | name | distance |
| B181 | chr18:55917356 | NEDD4L | 0 | + | 4702 | 6.89 | 0.0E+00 | 0.0E+00 | MIR122 | -200949 |
| | chr13:94939649 | GPC6 | 0 | - | 72 | 0.27 | 9.9E-27 | 4.5E-25 | DCT | 152191 |
| B195 | chr6:11399655 | NEDD9 | -17075 | + | 111 | 1.00 | 1.0E+00 | 1.0E+00 | TMEM170B | -138804 |
| A1 | chr17:39095514 | KRT23 | -1679 | + | n.d. | | | | KRT39 | 19154 |
| | chr18:25449894 | CDH2 | 81035 | - | 621 | 0.73 | 5.7E-09 | 3.0E-07 | CHST9 | -684593 |
| | chrX:138684373 | MCF2 | 0 | + | 150 | 32.70 | 4.0E-35 | 1.5E-32 | F9 | 38757 |
| A3 | chr11:16146421 | SOX6 | 0 | - | 41 | 1.09 | 7.3E-01 | 1.0E+00 | C11orf58 | -613726 |
| | chr11:113652668 | CLDN25 | 1462 | + | n.d. | | | | ZW10 | -8184 |
| | chr2:203664266 | ICA1L | 0 | - | 31 | 1.38 | 3.3E-01 | 9.7E-01 | FAM117B | 29787 |
| A6 | chr2:220825933 | MIR4268 | -54648 | - | n.d. | | | | SLC4A3 | 319232 |
| | chr5:122026084 | SNX2 | -84665 | - | 336 | 1.01 | 9.7E-01 | 1.0E+00 | SNX24 | -155075 |
| | chr5:139358867 | NRG2 | 0 | - | 52 | 1.47 | 7.8E-02 | 3.3E-01 | PSD2 | 134820 |
| | chr9:99951467 | LOC100499484 | -49240 | + | n.d. | | | | LOC100499484-C9O | -49240 |
| A7 | chr1:51138260 | FAF1 | 0 | - | 315 | 0.90 | 1.9E-01 | 5.5E-01 | DMRTA2 | -249142 |
| | chr2:83256616 | LOC1720 | 171724 | - | n.d. | | | | FUNDC2P2 | -1E+06 |
| | chr3:187823607 | LPP-AS2 (LPP) | 45386 | - | n.d. | | | | LPP | -48055 |
| A8 | chr2:39231446 | SOS1 | 0 | + | 271 | 0.82 | 2.1E-02 | 8.9E-02 | ARHGEF33 | 28857 |
| | chr13:94939649 | GPC6 | 0 | - | 174 | 0.70 | 4.5E-04 | 3.3E-03 | DCT | 152191 |
| A14 | chr13:94939649 | GPC6 | 0 | - | 132 | 0.59 | 1.1E-06 | 9.8E-06 | DCT | 152191 |
| A18 | chr7:140460815 | BRAF | 0 | + | 1731 | 31.97 | 0.0E+00 | 0.0E+00 | NDUFB2 | 54370 |
| | chr14:56156632 | KTN1 | 5331 | - | 584 | 0.67 | 7.0E-15 | 2.2E-13 | RPL13AP3 | -76330 |
| | chrX:115045927 | PLS3 | 160749 | - | 203 | 0.68 | 1.5E-05 | 1.2E-04 | AGTR2 | -256030 |
| A23 | chr7:140460815 | BRAF | 0 | + | 1612 | 34.14 | 0.0E+00 | 0.0E+00 | NDUFB2 | 54370 |
| | chr14:56156632 | KTN1 | 5331 | - | 498 | 0.66 | 6.0E-15 | 1.1E-13 | RPL13AP3 | -76330 |
| | chrX:115045927 | PLS3 | 160749 | - | 172 | 0.66 | 1.0E-05 | 6.9E-05 | AGTR2 | -256030 |

positive value for downstream), strand orientation ("+" and "-"), RNA-Seq read signal (RPKM, read per kilobase million), ratio to
sterisk if control expression is undetectable.

| second gene | | | | | third gene | | | | | | |
|---|---|---|---|---|---|---|---|---|---|---|---|
| strand | RPKM | RNA ratio | Pval | Qval | name | distance | strand | RPKM | RNA ratio | Pval | Qval |
| + | n.d. | | | | MIR3591 | 201028 | - | n.d. | | | |
| + | n.d. | | | | TGDS | 286658 | + | 136 | 1.04 | 8.0E-01 | 1.0E+00 |
| - | 40 | 1.34 | 2.3E-01 | 5.7E-01 | SMIM13 | 260687 | - | n.d. | | | |
| + | 386 | 50* | 3.5E-105 | 6.4E-102 | KRT40 | 38453 | + | n.d. | | | |
| - | n.d. | | | | AQP4-AS1 | 933985 | + | n.d. | | | |
| - | n.d. | | | | ATP11C | 124131 | + | 137 | 0.92 | 5.4E-01 | 1.0E+00 |
| + | 438 | 0.84 | 9.4E-03 | 1.0E-01 | PLEKHA7 | 662785 | - | 32 | 1.36 | 3.4E-01 | 9.8E-01 |
| - | 203 | 0.78 | 1.1E-02 | 1.1E-01 | USP28 | 15928 | - | 188 | 0.72 | 8.8E-04 | 1.6E-02 |
| + | 47 | 0.83 | 3.2E-01 | 9.5E-01 | WDR12 | 81056 | - | 220 | 0.93 | 4.5E-01 | 1.0E+00 |
| + | 118 | 1.28 | 1.0E-01 | 4.0E-01 | STK11IP | 344761 | + | 127 | 0.76 | 2.2E-02 | 1.3E-01 |
| - | 130 | 0.79 | 5.3E-02 | 2.5E-01 | MGC32805 | -211303 | + | n.d. | | | |
| + | n.d. | | | | PURA | -134840 | + | 331 | 1.11 | 2.2E-01 | 6.6E-01 |
| + | n.d. | | | | LOC340508 | -107241 | - | n.d. | | | |
| - | n.d. | | | | CDKN2C | -296106 | + | 391 | 1.49 | 8.2E-07 | 1.5E-05 |
| - | n.d. | | | | SUCLG1 | 1394030 | + | 301 | 0.95 | 5.4E-01 | 1.0E+00 |
| + | 5249 | 20.83 | 0.0E+00 | 0.0E+00 | FLJ42393 | -72723 | + | n.d. | | | |
| - | n.d. | | | | LOC375196 | -43962 | + | n.d. | | | |
| + | n.d. | | | | TGDS | 286658 | + | 121 | 1.01 | 1.0E+00 | 1.0E+00 |
| + | n.d. | | | | TGDS | 286658 | + | 98 | 0.91 | 4.9E-01 | 9.6E-01 |
| - | 257 | 1.12 | 2.2E-01 | 5.3E-01 | NDUFB2-AS1 | -63939 | + | n.d. | | | |
| - | n.d. | | | | LINC00520 | 91220 | + | n.d. | | | |
| - | n.d. | | | | LUZP4 | 503807 | - | n.d. | | | |
| - | 243 | 1.22 | 3.2E-02 | 9.8E-02 | NDUFB2-AS1 | -63939 | + | n.d. | | | |
| - | n.d. | | | | LINC00520 | 91220 | + | n.d. | | | |
| - | n.d. | | | | LUZP4 | 503807 | - | n.d. | | | |

**Table S4. STRING connections between screens.**

Lists of genes from each screen and their STRING (Search Tool for the Retrieval of Interacting Genes/Proteins) connections to other ge

| PB | ORF | dCAS9 | CRISPR | shRNA |
|---|---|---|---|---|
| NEDD4L | 8 | 3 | 11 | 1 |
| EYA1 | 10 | 4 | 7 | 0 |
| POU2F1 | 7 | 5 | 9 | 1 |
| BRAF | 11 | 4 | 3 | 0 |
| ZFHX3 | 6 | 0 | 6 | 1 |
| TRPS1 | 4 | 1 | 2 | 0 |
| ZFHX4 | 1 | 0 | 0 | 0 |
| RAPGEF2 | 8 | 5 | 3 | 0 |
| ARHGEF28 | 16 | 6 | 6 | 0 |
| VAV1 | 26 | 11 | 14 | 3 |
| PLS3 | 0 | 1 | 7 | 0 |
| GPC6 | 0 | 1 | 0 | 0 |
| WWTR1 | 16 | 2 | 4 | 0 |
| RAPGEF6 | 5 | 2 | 2 | 0 |
| AKAP13 | 11 | 5 | 3 | 0 |
| CREM | 16 | 2 | 8 | 1 |
| LOC339622 | 1 | 0 | 0 | 1 |
| PABPC4L | 0 | 0 | 0 | 0 |
| ESRRG | 24 | 1 | 3 | 0 |
| FOXP2 | 34 | 4 | 10 | 1 |
| HGSNAT | 1 | 1 | 2 | 0 |
| CHST9 | 0 | 0 | 0 | 0 |
| YES1 | 19 | 6 | 8 | 1 |
| ITSN2 | 2 | 1 | 3 | 0 |
| UHMK1 | 2 | 0 | 1 | 1 |
| KCNT2 | 0 | 1 | 2 | 0 |
| TMTC2 | 0 | 1 | 0 | 0 |
| LINC00433 | 1 | 0 | 0 | 1 |
| CHRM3 | 8 | 2 | 2 | 0 |
| RUNX1 | 31 | 5 | 18 | 4 |
| PCDH7 | 0 | 3 | 0 | 0 |
| CDH6 | 1 | 1 | 0 | 0 |
| ATP10A | 4 | 4 | 6 | 0 |
| MECOM | 22 | 5 | 8 | 2 |
| HNF4G | 5 | 0 | 0 | 0 |
| PLSCR5 | 0 | 0 | 0 | 0 |
| DCN | 10 | 3 | 9 | 2 |
| GCKR | 4 | 1 | 1 | 0 |
| PRRX1 | 7 | 1 | 7 | 0 |
| HACE1 | 3 | 0 | 4 | 0 |
| IRF2 | 9 | 2 | 6 | 0 |
| PDE8A | 1 | 0 | 0 | 2 |
| GPR68 | 7 | 1 | 0 | 0 |
| ANTXR2 | 8 | 4 | 13 | 1 |
| ZFPM2 | 10 | 1 | 4 | 2 |
| KCNIP4 | 0 | 0 | 0 | 0 |
| DOCK10 | 0 | 0 | 0 | 1 |
| WIPF1 | 5 | 0 | 1 | 2 |
| ADAD1 | 0 | 1 | 5 | 1 |
| IFLTD1 | 0 | 0 | 0 | 0 |

| CRISPR | PB | ORF | dCAS9 | shRNA |
|---|---|---|---|---|
| RPS19 | 0 | 2 | 1 | 2 |
| TCOF1 | 2 | 3 | 0 | 0 |
| NIP7 | 1 | 5 | 4 | 3 |
| ARL17A | 0 | 0 | 0 | 0 |
| RPS18 | 0 | 1 | 3 | 1 |
| RPS11 | 1 | 0 | 1 | 1 |
| RPL32 | 0 | 1 | 1 | 1 |
| DENR | 2 | 3 | 2 | 3 |
| RPL6 | 1 | 8 | 4 | 9 |
| ACTL6A | 4 | 7 | 2 | 5 |
| PPAN | 8 | 3 | 2 | 2 |
| ENO1 | 11 | 15 | 11 | 4 |
| ASH2L | 3 | 9 | 1 | 1 |
| SNRPF | 1 | 5 | 2 | 4 |
| GABPA | 6 | 5 | 0 | 0 |
| VOPP1 | 0 | 0 | 1 | 0 |
| RSL1D1 | 0 | 3 | 2 | 0 |
| URB1 | 0 | 2 | 1 | 0 |
| MRP63 | 3 | 2 | 1 | 0 |
| NAA30 | 1 | 2 | 0 | 0 |
| KPNB1 | 5 | 6 | 1 | 2 |
| LSM2 | 0 | 0 | 0 | 1 |
| MASP2 | 1 | 2 | 0 | 1 |
| DEFB131 | 0 | 0 | 0 | 0 |
| NHP2L1 | 1 | 2 | 3 | 2 |
| RPS15A | 3 | 4 | 2 | 1 |
| EEF1A1 | 2 | 8 | 5 | 1 |
| RBX1 | 4 | 11 | 5 | 2 |
| RPL8 | 1 | 5 | 1 | 1 |
| POLR2I | 2 | 3 | 2 | 3 |
| NUTF2 | 1 | 3 | 1 | 1 |
| TPT1 | 5 | 5 | 5 | 3 |
| ATP6V0C | 0 | 2 | 0 | 0 |
| RPL7A | 1 | 2 | 1 | 1 |
| EEF2 | 1 | 9 | 5 | 2 |
| COPB1 | 5 | 10 | 4 | 1 |
| RBMX2 | 1 | 6 | 0 | 2 |
| POLR3H | 5 | 2 | 1 | 1 |
| RPL10 | 1 | 8 | 1 | 2 |
| TUBG1 | 2 | 6 | 3 | 2 |
| KCNJ16 | 4 | 0 | 1 | 0 |
| ZNF207 | 1 | 2 | 1 | 1 |
| CAPZB | 0 | 0 | 0 | 0 |
| HNRNPU | 0 | 10 | 2 | 3 |
| RPL37 | 1 | 2 | 1 | 1 |
| CAPZA1 | 1 | 4 | 0 | 0 |
| SS18L2 | 0 | 2 | 0 | 0 |
| PRPF38B | 3 | 1 | 0 | 2 |
| MAPK1 | 21 | 51 | 19 | 7 |
| MOCS3 | 1 | 2 | 2 | 0 |

| dCAS9 | PB | ORF | CRISPR |
|---|---|---|---|
| MARCH13 | 0 | 0 | 0 |
| AARSD1 | 0 | 0 | 0 |
| ABLIM2 | 7 | 13 | 2 |
| ACP6 | 0 | 0 | 0 |
| ACVR2A | 0 | 3 | 0 |
| ADORA1 | 1 | 11 | 1 |
| AKR1B1 | 0 | 1 | 0 |
| ANKRD29 | 0 | 1 | 0 |
| AP4B1 | 0 | 1 | 1 |
| APBB1 | 3 | 17 | 60 |
| ARHGAP6 | 0 | 0 | 0 |
| ARHGEF1 | 8 | 17 | 9 |
| ARHGEF2 | 10 | 25 | 35 |
| ARHGEF5 | 0 | 0 | 0 |
| ATL1 | 0 | 0 | 0 |
| ATP10A | 5 | 4 | 6 |
| B4GALNT2 | 0 | 0 | 0 |
| BCAP29 | 0 | 0 | 3 |
| BCAR1 | 4 | 12 | 3 |
| BCAR3 | 3 | 2 | 3 |
| BCAS3 | 14 | 28 | 24 |
| BCL7C | 1 | 2 | 18 |
| BRI3 | 0 | 4 | 8 |
| BRINP1 | 0 | 0 | 0 |
| C11orf21 | 0 | 0 | 0 |
| C14orf39 | 0 | 0 | 0 |
| C19orf18 | 0 | 0 | 0 |
| C19orf68 | 0 | 0 | 0 |
| C3orf27 | 0 | 0 | 0 |
| C9orf50 | 0 | 0 | 0 |
| CA12 | 0 | 4 | 5 |
| CA3 | 0 | 0 | 0 |
| CCER1 | 0 | 0 | 0 |
| CCND2 | 13 | 23 | 17 |
| CDR2 | 1 | 0 | 0 |
| CEP63 | 1 | 1 | 3 |
| CGB8 | 0 | 0 | 0 |
| CGNL1 | 3 | 2 | 3 |
| CHID1 | 2 | 4 | 6 |
| CHN2 | 8 | 17 | 4 |
| CHST15 | 0 | 0 | 0 |
| CNR1 | 1 | 1 | 3 |
| COA3 | 0 | 1 | 8 |
| COL25A1 | 1 | 6 | 1 |
| CPEB1 | 0 | 0 | 0 |
| CPLX2 | 2 | 1 | 3 |
| CRB2 | 0 | 3 | 4 |
| CST5 | 0 | 1 | 0 |
| DAG1 | 7 | 8 | 2 |
| DCAF7 | 0 | 0 | 0 |

| | | | | |
|---|---|---|---|---|
| SNX6 | 1 | 2 | 2 | 1 |
| MAGI1 | 1 | 1 | 1 | 0 |
| LGR5 | 9 | 2 | 3 | 0 |
| NINJ2 | 0 | 0 | 0 | 0 |
| UGGT2 | 0 | 0 | 1 | 1 |
| TBX22 | 9 | 1 | 3 | 0 |
| CCDC85A | 1 | 0 | 3 | 0 |
| MLIP | 0 | 1 | 0 | 0 |
| LINC00410 | 1 | 0 | 0 | 1 |
| CDH18 | 1 | 0 | 0 | 0 |
| LRIG2 | 3 | 1 | 0 | 0 |
| SLC38A2 | 3 | 2 | 8 | 2 |
| KLF12 | 2 | 1 | 0 | 0 |
| IRF6 | 13 | 3 | 10 | 1 |
| CDC73 | 2 | 1 | 21 | 0 |
| ADHFE1 | 9 | 4 | 9 | 1 |
| CCDC146 | 1 | 0 | 1 | 0 |
| PRR20A | 0 | 0 | 0 | 0 |
| ZNF507 | 1 | 0 | 0 | 0 |
| SLC33A1 | 1 | 0 | 7 | 1 |
| SLC35F3 | 0 | 0 | 1 | 0 |
| NELL2 | 2 | 1 | 0 | 0 |
| MMP2 | 19 | 9 | 7 | 1 |
| CHM | 2 | 1 | 12 | 2 |
| SEMA6A | 7 | 2 | 9 | 0 |
| ETV3 | 2 | 1 | 2 | 0 |
| SFRP1 | 5 | 6 | 5 | 0 |
| GRM8 | 5 | 4 | 2 | 1 |
| SUMO1P1 | 1 | 0 | 0 | 1 |
| EPHA7 | 7 | 4 | 1 | 0 |
| SKAP2 | 3 | 0 | 1 | 1 |
| TENM2 | 0 | 0 | 0 | 0 |
| GPR113 | 0 | 0 | 0 | 0 |
| SYT11 | 5 | 2 | 5 | 0 |
| PPP1R32 | 3 | 0 | 0 | 0 |
| PDZRN4 | 2 | 0 | 3 | 0 |
| FAM160A1 | 0 | 0 | 0 | 0 |
| DLGAP1 | 2 | 0 | 1 | 0 |
| DYNLRB2 | 2 | 0 | 6 | 2 |
| PTPRQ | 0 | 0 | 0 | 0 |
| PTPRZ1 | 4 | 4 | 0 | 0 |
| LECT1 | 1 | 1 | 1 | 0 |
| LOC440970 | 1 | 0 | 0 | 1 |
| RAF1 | 23 | 5 | 11 | 2 |
| KCNIP1 | 3 | 3 | 0 | 0 |
| PDZRN3 | 4 | 0 | 2 | 0 |
| UBE3C | 1 | 0 | 5 | 0 |
| MCTP2 | 0 | 0 | 1 | 0 |
| SCAF8 | 2 | 2 | 15 | 2 |
| PDE4DIP | 2 | 0 | 0 | 0 |
| BCAS3 | 1 | 1 | 4 | 1 |
| LRRC4C | 2 | 0 | 1 | 1 |
| PDHA2 | 0 | 1 | 3 | 1 |

| | | | | |
|---|---|---|---|---|
| POP7 | 0 | 2 | 0 | 2 |
| UTP18 | 1 | 7 | 2 | 1 |
| PSMC2 | 3 | 7 | 7 | 2 |
| PIGW | 0 | 0 | 0 | 0 |
| RPL30 | 2 | 5 | 1 | 1 |
| DCPS | 0 | 1 | 1 | 3 |
| RPS21 | 0 | 0 | 0 | 0 |
| BEX1 | 1 | 2 | 1 | 1 |
| SNRNP70 | 1 | 6 | 5 | 2 |
| H3F3B | 18 | 40 | 13 | 9 |
| RPL38 | 1 | 1 | 1 | 1 |
| TSR1 | 1 | 6 | 2 | 2 |
| SNRNP200 | 0 | 3 | 1 | 1 |
| ZNRD1 | 0 | 2 | 0 | 0 |
| TFAM | 3 | 8 | 2 | 1 |
| PDAP1 | 0 | 2 | 0 | 0 |
| OCM | 2 | 3 | 1 | 0 |
| PCF11 | 2 | 1 | 0 | 2 |
| C6orf226 | 0 | 0 | 0 | 0 |
| PPP2CA | 3 | 14 | 2 | 2 |
| RAE1 | 4 | 6 | 0 | 1 |
| CIRH1A | 0 | 4 | 0 | 1 |
| DDX42 | 0 | 2 | 0 | 1 |
| RPS5 | 2 | 6 | 2 | 2 |
| SART3 | 0 | 2 | 2 | 0 |
| CTU2 | 0 | 3 | 0 | 0 |
| SBDS | 0 | 5 | 1 | 0 |
| HSD17B10 | 0 | 7 | 3 | 2 |
| OR5K3 | 0 | 0 | 0 | 0 |
| PMF1 | 0 | 1 | 0 | 0 |
| ZNF259 | 1 | 0 | 2 | 0 |
| MTA3 | 1 | 1 | 0 | 1 |
| ANKRD49 | 0 | 0 | 0 | 0 |
| PCBP1 | 1 | 4 | 3 | 3 |
| FCF1 | 1 | 1 | 1 | 0 |
| CTNNBL1 | 0 | 1 | 0 | 1 |
| TUBGCP3 | 3 | 0 | 3 | 1 |
| KDSR | 0 | 1 | 1 | 0 |
| HSPA8 | 4 | 19 | 5 | 3 |
| ZFHX3 | 4 | 6 | 0 | 1 |
| DYNLL1 | 7 | 7 | 3 | 2 |
| C17orf81 | 0 | 0 | 0 | 0 |
| POLR3K | 1 | 4 | 1 | 1 |
| SAE1 | 1 | 9 | 1 | 4 |
| TBCA | 1 | 4 | 1 | 2 |
| RPS27A | 31 | 75 | 24 | 8 |
| ARMC7 | 0 | 0 | 0 | 0 |
| POLR1B | 3 | 3 | 1 | 1 |
| FAM96B | 1 | 3 | 3 | 2 |
| RPL13 | 1 | 2 | 3 | 1 |
| CUL1 | 4 | 21 | 7 | 3 |
| SUPT6H | 2 | 2 | 2 | 0 |
| YY1 | 15 | 23 | 7 | 5 |

| | | | |
|---|---|---|---|
| DDX11 | 7 | 16 | 9 |
| DNASE1L2 | 10 | 16 | 11 |
| DTX3 | 0 | 2 | 1 |
| DYRK3 | 0 | 0 | 0 |
| EFNA1 | 6 | 12 | 1 |
| EGFR | 29 | 54 | 35 |
| EIF4EBP2 | 0 | 0 | 0 |
| EPAS1 | 0 | 0 | 0 |
| FGD1 | 7 | 17 | 8 |
| FGF17 | 2 | 8 | 5 |
| FGF8 | 10 | 16 | 11 |
| FICD | 1 | 3 | 4 |
| FOXJ1 | 0 | 0 | 0 |
| FOXO4 | 0 | 0 | 0 |
| FSD1 | 0 | 0 | 0 |
| GAB2 | 0 | 0 | 0 |
| GABRQ | 0 | 0 | 0 |
| GBE1 | 0 | 4 | 8 |
| GCK | 3 | 9 | 5 |
| GCNT1 | 4 | 4 | 6 |
| GLIS3 | 2 | 5 | 1 |
| GPR35 | 0 | 6 | 0 |
| GSR | 2 | 6 | 7 |
| HDX | 0 | 0 | 0 |
| HMGXB3 | 0 | 0 | 0 |
| HOXB4 | 0 | 0 | 0 |
| IER3IP1 | 0 | 2 | 1 |
| IFNGR1 | 2 | 9 | 1 |
| IGF1R | 9 | 20 | 14 |
| INHBA | 0 | 1 | 15 |
| IQGAP3 | 4 | 2 | 7 |
| ISLR2 | 0 | 0 | 0 |
| ITGA9 | 2 | 5 | 1 |
| ITGB3 | 0 | 0 | 0 |
| ITGB5 | 1 | 6 | 1 |
| JUN | 0 | 0 | 0 |
| KCND1 | 2 | 0 | 0 |
| KCTD20 | 1 | 2 | 1 |
| KIAA0040 | 0 | 0 | 0 |
| KIAA1804 | 1 | 3 | 5 |
| KRAS | 0 | 6 | 0 |
| LAMP5 | 0 | 2 | 0 |
| LOC1027248 | 0 | 0 | 0 |
| LOC730183 | 0 | 1 | 0 |
| LPAR1 | 5 | 11 | 0 |
| LPAR5 | 0 | 1 | 0 |
| LRRC10B | 0 | 0 | 0 |
| LYPD2 | 1 | 1 | 5 |
| MAGEB6 | 0 | 0 | 0 |
| MAP3K11 | 1 | 11 | 3 |
| MDK | 3 | 1 | 1 |
| MECOM | 6 | 18 | 2 |
| MEIS2 | 2 | 21 | 0 |

| | | | | |
|---|---|---|---|---|
| SOX5 | 11 | 2 | 5 | 1 |
| C15orf54 | 0 | 0 | 0 | 0 |
| ARID1A | 4 | 1 | 8 | 2 |
| PIK3C2G | 4 | 0 | 3 | 1 |
| SEMA3D | 1 | 0 | 0 | 0 |
| PCDH10 | 2 | 0 | 1 | 0 |
| GALNTL6 | 0 | 0 | 0 | 0 |
| USP25 | 1 | 0 | 5 | 1 |
| ISL1 | 22 | 6 | 9 | 1 |
| NRXN1 | 1 | 3 | 4 | 0 |
| DNAJC11 | 0 | 0 | 1 | 0 |
| TRIT1 | 1 | 2 | 16 | 3 |
| MIER1 | 5 | 1 | 9 | 1 |
| DIRAS2 | 1 | 2 | 1 | 0 |
| MTIF2 | 3 | 3 | 49 | 2 |
| LSAMP | 1 | 0 | 0 | 1 |
| GLIS3 | 4 | 1 | 0 | 0 |
| KIAA1217 | 4 | 3 | 3 | 0 |
| AGBL1 | 0 | 0 | 1 | 0 |
| FAM5C | 1 | 0 | 0 | 1 |
| OR7E156P | 1 | 0 | 0 | 1 |
| RAPGEF4 | 16 | 2 | 4 | 1 |
| MIR3134 | 1 | 0 | 0 | 1 |
| LOC91948 | 1 | 0 | 0 | 1 |
| ELK3 | 2 | 0 | 4 | 0 |
| NEDD9 | 7 | 4 | 3 | 1 |
| KTN1 | 0 | 0 | 0 | 0 |
| SNX14 | 4 | 1 | 2 | 0 |
| PPARG | 35 | 9 | 28 | 3 |

| | | | | |
|---|---|---|---|---|
| RANBP1 | 0 | 9 | 2 | 3 |
| RPL13A | 1 | 3 | 1 | 1 |
| BUD31 | 1 | 3 | 0 | 1 |
| CDC45 | 0 | 11 | 3 | 3 |
| NOC4L | 0 | 4 | 0 | 0 |
| EXOSC8 | 3 | 3 | 1 | 2 |
| DGCR8 | 2 | 10 | 1 | 2 |
| EXOSC5 | 1 | 5 | 2 | 3 |
| USP1 | 4 | 4 | 3 | 5 |
| ANAPC7 | 1 | 4 | 1 | 1 |
| CCDC73 | 0 | 0 | 0 | 0 |
| RBBP5 | 2 | 5 | 1 | 0 |
| ARL2 | 1 | 3 | 0 | 0 |
| RFC5 | 3 | 6 | 2 | 3 |
| HDAC3 | 6 | 23 | 3 | 7 |
| PSMB3 | 1 | 5 | 3 | 2 |
| RAB1B | 8 | 18 | 8 | 1 |
| EIF3H | 2 | 4 | 2 | 2 |
| C15orf63 | 0 | 0 | 0 | 0 |
| GTPBP4 | 0 | 5 | 1 | 3 |
| PSMA6 | 2 | 4 | 3 | 1 |
| COX6B1 | 1 | 6 | 3 | 2 |
| ZBTB8OS | 1 | 1 | 0 | 0 |
| EIF6 | 0 | 10 | 3 | 4 |
| CDAN1 | 5 | 3 | 2 | 1 |
| RSL24D1 | 2 | 4 | 2 | 2 |
| PES1 | 1 | 5 | 1 | 1 |
| SKP1 | 8 | 21 | 8 | 6 |
| EIF1 | 1 | 3 | 1 | 2 |
| HSPD1 | 7 | 18 | 5 | 3 |
| PFDN6 | 0 | 0 | 1 | 0 |
| MED21 | 2 | 9 | 3 | 2 |
| WDR55 | 0 | 1 | 0 | 0 |
| CWC25 | 1 | 1 | 0 | 0 |
| RPA3 | 0 | 4 | 3 | 0 |
| UTP14A | 0 | 7 | 1 | 1 |
| CHORDC1 | 3 | 3 | 0 | 1 |
| RBM48 | 2 | 0 | 0 | 0 |
| DDX41 | 0 | 7 | 1 | 2 |
| PSME3 | 0 | 6 | 3 | 0 |
| AURKA | 2 | 13 | 5 | 3 |
| NCL | 3 | 16 | 2 | 3 |
| RPS9 | 1 | 2 | 2 | 1 |
| POLR3B | 5 | 2 | 2 | 1 |
| MPLKIP | 1 | 3 | 4 | 0 |
| UFD1L | 1 | 5 | 0 | 1 |
| ZMYND8 | 1 | 1 | 0 | 0 |
| CCNA2 | 1 | 14 | 7 | 4 |
| ALG2 | 2 | 3 | 1 | 1 |
| RPAIN | 0 | 1 | 0 | 0 |
| EIF2S2 | 2 | 6 | 5 | 1 |
| SNAPC4 | 2 | 0 | 0 | 1 |
| FCGR1A | 3 | 11 | 4 | 1 |

| | | | |
|---|---|---|---|
| MGAT3 | 0 | 0 | 0 |
| MLLT6 | 0 | 0 | 0 |
| MMRN2 | 1 | 4 | 10 |
| MRFAP1 | 1 | 3 | 9 |
| MRPS35 | 2 | 3 | 42 |
| MSRB3 | 2 | 0 | 1 |
| NBL1 | 0 | 3 | 4 |
| NEFM | 0 | 0 | 0 |
| NEIL3 | 1 | 2 | 18 |
| NEK5 | 1 | 2 | 1 |
| NFS1 | 3 | 14 | 8 |
| OSBPL1A | 3 | 0 | 2 |
| P2RY1 | 0 | 0 | 0 |
| P2RY8 | 1 | 3 | 2 |
| PABPC5 | 2 | 1 | 3 |
| PAK7 | 0 | 0 | 0 |
| PBX1 | 2 | 9 | 1 |
| PBX2 | 0 | 1 | 0 |
| PCDH7 | 3 | 0 | 0 |
| PDCD4 | 0 | 9 | 11 |
| PHB | 2 | 9 | 25 |
| PHC2 | 0 | 3 | 5 |
| PLEKHG5 | 1 | 13 | 2 |
| PLXDC2 | 3 | 3 | 0 |
| PPDPF | 0 | 0 | 0 |
| PRKCE | 0 | 0 | 0 |
| PROM1 | 7 | 16 | 7 |
| PSMF1 | 0 | 1 | 15 |
| PYGO1 | 0 | 3 | 0 |
| RAB42 | 0 | 0 | 0 |
| RAPGEF1 | 7 | 13 | 2 |
| RASGRF1 | 3 | 15 | 8 |
| RASSF5 | 3 | 17 | 5 |
| RHOG | 0 | 0 | 0 |
| RNF223 | 0 | 0 | 0 |
| RNF41 | 0 | 1 | 4 |
| RNF6 | 1 | 4 | 4 |
| RPS16 | 1 | 4 | 42 |
| RRAS2 | 9 | 17 | 11 |
| SEBOX | 0 | 0 | 0 |
| SHB | 1 | 7 | 1 |
| SIGIRR | 1 | 3 | 1 |
| SLC19A2 | 1 | 3 | 1 |
| SLC25A20 | 2 | 3 | 5 |
| SLC2A3 | 0 | 0 | 0 |
| SLC32A1 | 5 | 3 | 2 |
| SNED1 | 0 | 1 | 0 |
| SNX13 | 3 | 1 | 1 |
| SOCS6 | 0 | 0 | 0 |
| SPHK1 | 1 | 4 | 10 |
| SSC5D | 0 | 0 | 0 |
| STAT4 | 3 | 15 | 8 |
| STAU2 | 4 | 1 | 5 |

| | | | | |
|---|---|---|---|---|
| PFDN4 | 2 | 4 | 0 | 1 |
| C5orf62 | 0 | 0 | 0 | 0 |
| C7orf23 | 0 | 0 | 0 | 0 |
| PGP | 0 | 2 | 0 | 0 |
| ACTR6 | 5 | 6 | 4 | 2 |
| CNPY2 | 0 | 1 | 4 | 1 |
| CCDC58 | 0 | 0 | 1 | 0 |
| MCM3AP | 1 | 0 | 0 | 0 |
| SERAC1 | 0 | 0 | 0 | 0 |
| RFC3 | 3 | 3 | 1 | 5 |
| POLR1A | 3 | 2 | 1 | 1 |
| TRMT6 | 0 | 3 | 0 | 0 |
| YPEL1 | 0 | 0 | 0 | 0 |
| TRIAP1 | 0 | 0 | 0 | 0 |
| TOR1B | 0 | 0 | 0 | 0 |
| OR5AR1 | 0 | 0 | 0 | 0 |
| MAX | 6 | 16 | 4 | 3 |
| STX18 | 1 | 3 | 0 | 0 |
| SNRPG | 2 | 2 | 1 | 2 |
| RBMX | 3 | 6 | 1 | 1 |
| POLRMT | 0 | 0 | 0 | 0 |
| GCSH | 3 | 2 | 3 | 2 |
| PDRG1 | 0 | 1 | 0 | 0 |
| NRF1 | 2 | 6 | 0 | 0 |
| EIF5 | 2 | 3 | 1 | 2 |
| ELAC2 | 2 | 7 | 1 | 0 |
| RAN | 12 | 13 | 9 | 5 |
| BCAS2 | 2 | 3 | 3 | 3 |
| CTU1 | 0 | 0 | 0 | 0 |
| CNIH4 | 0 | 0 | 0 | 1 |
| EXOSC1 | 1 | 2 | 1 | 0 |
| GINS2 | 0 | 2 | 3 | 0 |
| TAF1C | 1 | 4 | 0 | 1 |
| TARS | 4 | 3 | 4 | 2 |
| RPL36 | 2 | 2 | 1 | 3 |
| MRPS18C | 0 | 0 | 1 | 0 |
| MRPL36 | 0 | 0 | 1 | 0 |
| MFAP1 | 0 | 1 | 2 | 1 |
| FAM185A | 0 | 0 | 0 | 0 |
| VN1R2 | 0 | 0 | 0 | 0 |
| VPS13D | 1 | 0 | 0 | 0 |
| ANAPC1 | 2 | 3 | 2 | 0 |
| CEBPZ | 7 | 7 | 1 | 2 |
| FARSA | 2 | 5 | 1 | 1 |
| NBR1 | 2 | 3 | 1 | 4 |
| XPO1 | 0 | 0 | 0 | 0 |
| KRTCAP2 | 1 | 0 | 1 | 1 |
| C19orf69 | 0 | 0 | 0 | 0 |
| RRP1B | 0 | 4 | 0 | 0 |
| SPTLC1 | 1 | 2 | 1 | 0 |
| CFHR4 | 1 | 0 | 0 | 0 |
| SRCAP | 3 | 3 | 2 | 1 |
| HAUS1 | 2 | 1 | 2 | 1 |

| | | | |
|---|---|---|---|
| TAPBP | 1 | 1 | 5 |
| TAS2R19 | 0 | 1 | 0 |
| TCEA2 | 1 | 2 | 11 |
| TCF7L1 | 0 | 0 | 3 |
| TEAD4 | 1 | 4 | 4 |
| TFAP2C | 1 | 5 | 4 |
| TFEB | 3 | 28 | 5 |
| TMEM129 | 0 | 0 | 0 |
| TMEM133 | 0 | 1 | 0 |
| TMEM199 | 0 | 0 | 0 |
| TMEM26 | 0 | 0 | 1 |
| TNFRSF1B | 7 | 17 | 8 |
| TNNC1 | 1 | 3 | 2 |
| TNRC18 | 3 | 0 | 0 |
| TOR3A | 0 | 0 | 0 |
| TRIB1 | 1 | 9 | 5 |
| TRIM65 | 0 | 3 | 5 |
| TRIM7 | 0 | 0 | 0 |
| TYW1 | 1 | 1 | 3 |
| UBE2E3 | 0 | 0 | 0 |
| VKORC1 | 1 | 5 | 7 |
| VSX1 | 4 | 2 | 2 |
| WNT7A | 6 | 6 | 0 |
| ZASP | 0 | 0 | 0 |
| ZCCHC11 | 12 | 17 | 11 |
| ZFHX4 | 7 | 9 | 9 |
| ZNF582 | 0 | 1 | 1 |
| ZNF704 | 4 | 12 | 3 |
| ZNF747 | 0 | 0 | 0 |
| ZNF83 | 0 | 3 | 4 |

| | | | | |
|---|---|---|---|---|
| ATR | 4 | 6 | 5 | 1 |
| AHSA2 | 3 | 1 | 1 | 0 |
| SPHAR | 2 | 0 | 1 | 0 |
| NUPL1 | 1 | 2 | 0 | 0 |
| SMG1 | 0 | 4 | 0 | 1 |
| KIAA0586 | 0 | 1 | 1 | 0 |
| RPP21 | 0 | 0 | 0 | 4 |
| LSM11 | 0 | 0 | 0 | 0 |
| NDNL2 | 2 | 0 | 1 | 0 |
| ACTL6B | 2 | 4 | 1 | 3 |
| MMS22L | 1 | 2 | 1 | 0 |
| UBA52 | 29 | 73 | 24 | 8 |
| PPP2R3C | 1 | 1 | 1 | 1 |
| PRPF6 | 2 | 2 | 0 | 1 |
| ATP5I | 0 | 0 | 0 | 0 |
| HCFC1 | 5 | 15 | 1 | 2 |
| PABPN1 | 0 | 11 | 0 | 1 |
| SFSWAP | 1 | 1 | 0 | 0 |
| GINS1 | 1 | 2 | 2 | 1 |
| ATP5B | 4 | 10 | 5 | 4 |
| PPP4C | 1 | 8 | 3 | 3 |
| ZNF513 | 0 | 2 | 1 | 0 |
| C15orf42 | 0 | 0 | 0 | 0 |
| CHKA | 1 | 11 | 4 | 1 |
| RBM39 | 0 | 1 | 2 | 1 |
| HAUS5 | 0 | 0 | 0 | 0 |
| FANCM | 1 | 2 | 0 | 0 |
| DNAJC17 | 0 | 1 | 0 | 0 |
| C21orf59 | 0 | 0 | 0 | 0 |
| TDO2 | 0 | 0 | 0 | 0 |
| BRAT1 | 0 | 1 | 0 | 0 |
| TOMM22 | 0 | 1 | 1 | 1 |
| OVGP1 | 1 | 1 | 1 | 0 |
| SMC5 | 0 | 0 | 1 | 1 |
| C19orf52 | 0 | 0 | 0 | 0 |
| U2AF1 | 1 | 3 | 0 | 2 |
| HORMAD2 | 1 | 1 | 0 | 0 |
| GLRA1 | 6 | 4 | 3 | 0 |
| FAM98B | 0 | 0 | 0 | 0 |
| MYC | 29 | 53 | 16 | 6 |
| PDCD7 | 0 | 1 | 0 | 0 |
| PSIP1 | 4 | 7 | 0 | 2 |
| HELZ | 1 | 2 | 0 | 1 |
| ANKLE2 | 0 | 0 | 0 | 1 |
| RPS6 | 5 | 13 | 8 | 3 |
| PADI4 | 1 | 4 | 1 | 1 |
| PRPF38A | 0 | 0 | 0 | 0 |
| GTF3C1 | 1 | 4 | 0 | 1 |
| ASNS | 1 | 4 | 0 | 0 |
| PPYR1 | 0 | 0 | 0 | 0 |
| SUZ12 | 3 | 15 | 2 | 3 |
| SAFB | 2 | 6 | 0 | 0 |
| SPC24 | 0 | 2 | 2 | 0 |

| | | | | |
|---|---|---|---|---|
| FAM63B | 0 | 0 | 0 | 0 |

enesets. PB, transposon.

| shRNA |
|---|
| 0 |
| 0 |
| 0 |
| 0 |
| 0 |
| 0 |
| 0 |
| 1 |
| 0 |
| 3 |
| 0 |
| 0 |
| 4 |
| 0 |
| 0 |
| 0 |
| 0 |
| 0 |
| 1 |
| 0 |
| 2 |
| 0 |
| 0 |
| 0 |
| 0 |
| 0 |
| 0 |
| 0 |
| 0 |
| 0 |
| 2 |
| 0 |
| 0 |
| 2 |
| 0 |
| 0 |
| 0 |
| 0 |
| 2 |
| 0 |
| 0 |
| 0 |
| 1 |
| 0 |
| 0 |
| 0 |
| 0 |
| 0 |
| 0 |
| 0 |

| ORF | PB | dCAS9 | CRISPR | shRNA |
|---|---|---|---|---|
| NR4A1 | 5 | 3 | 5 | 2 |
| TFEB | 3 | 17 | 5 | 1 |
| POU5F1 | 4 | 2 | 10 | 2 |
| YAP1 | 6 | 5 | 9 | 1 |
| MITF | 7 | 4 | 15 | 3 |
| GCM2 | 5 | 1 | 1 | 0 |
| ETV1 | 5 | 2 | 6 | 1 |
| HNF4A | 14 | 9 | 17 | 3 |
| MYOD1 | 16 | 12 | 22 | 4 |
| TFAP2A | 12 | 1 | 8 | 1 |
| FOXP3 | 10 | 8 | 9 | 4 |
| ASCL2 | 2 | 3 | 7 | 1 |
| MEOX2 | 1 | 0 | 0 | 0 |
| HOXD9 | 6 | 8 | 15 | 2 |
| FOXA3 | 3 | 0 | 1 | 2 |
| TLE1 | 3 | 2 | 11 | 1 |
| ASCL4 | 0 | 0 | 2 | 0 |
| ETS2 | 5 | 3 | 5 | 2 |
| TGIF1 | 1 | 0 | 1 | 0 |
| SOX14 | 4 | 1 | 1 | 0 |
| PASD1 | 0 | 0 | 3 | 0 |
| HOXC11 | 5 | 0 | 4 | 0 |
| EBF1 | 9 | 4 | 8 | 2 |
| JUNB | 8 | 5 | 11 | 2 |
| KLF6 | 4 | 2 | 8 | 3 |
| SATB1 | 1 | 1 | 6 | 1 |
| SHOX2 | 5 | 1 | 1 | 0 |
| GPR101 | 0 | 2 | 5 | 1 |
| GPR35 | 0 | 5 | 0 | 0 |
| LPAR1 | 5 | 9 | 0 | 0 |
| GPR132 | 2 | 1 | 7 | 0 |
| GPR52 | 0 | 1 | 0 | 0 |
| GPR161 | 1 | 0 | 0 | 0 |
| GPR119 | 2 | 1 | 1 | 0 |
| GNA15 | 3 | 7 | 15 | 3 |
| P2RY8 | 1 | 2 | 2 | 0 |
| NGEF | 5 | 3 | 3 | 0 |
| PLEKHG5 | 1 | 11 | 2 | 0 |
| ARHGEF9 | 3 | 2 | 9 | 2 |
| PLEKHG3 | 0 | 0 | 0 | 0 |
| PLEKHG6 | 1 | 1 | 1 | 0 |
| RASGRP4 | 0 | 1 | 2 | 1 |
| ARHGEF19 | 1 | 0 | 0 | 0 |
| ARHGEF3 | 3 | 3 | 0 | 0 |
| RAF1 | 10 | 5 | 11 | 2 |
| PAK3 | 3 | 2 | 8 | 0 |
| PAK1 | 6 | 11 | 15 | 3 |
| MOS | 1 | 1 | 3 | 0 |
| FBXO5 | 1 | 1 | 40 | 3 |
| TNFAIP1 | 0 | 0 | 2 | 0 |

| shRNA | PB | ORF | dCAS9 | CRISPR |
|---|---|---|---|---|
| NF1 | 15 | 25 | 5 | 16 |
| HOXD8 | 2 | 3 | 1 | 4 |
| COL8A1 | 1 | 0 | 1 | 0 |
| VCX3A | 0 | 0 | 0 | 1 |
| POFUT1 | 1 | 4 | 1 | 7 |
| NCF1 | 2 | 12 | 1 | 1 |
| MAP1S | 0 | 2 | 1 | 9 |
| MRS2 | 10 | 4 | 3 | 20 |
| MORF4 | 1 | 1 | 0 | 0 |
| EMP2 | 1 | 0 | 2 | 1 |
| TNKS1BP1 | 1 | 0 | 1 | 1 |
| POTEB | 0 | 0 | 0 | 0 |
| TFDP1 | 0 | 6 | 2 | 22 |
| ZNF606 | 2 | 0 | 0 | 0 |
| HSPC105 | 1 | 1 | 0 | 0 |
| ATP1A1 | 2 | 6 | 1 | 9 |
| LOC402434 | 1 | 1 | 0 | 0 |
| WDR57 | 0 | 4 | 0 | 36 |
| RPL6 | 1 | 8 | 4 | 54 |
| RPP21 | 0 | 0 | 0 | 15 |
| MED12 | 1 | 15 | 1 | 18 |
| SFPQ | 2 | 5 | 0 | 27 |
| CSRNP1 | 0 | 8 | 1 | 0 |
| SPOCK3 | 3 | 0 | 1 | 0 |
| FYB | 4 | 15 | 1 | 5 |
| HDAC2 | 13 | 29 | 7 | 58 |
| GSK3A | 4 | 11 | 4 | 10 |
| PRKACB | 1 | 4 | 4 | 5 |
| THNSL2 | 0 | 0 | 0 | 0 |
| C20orf85 | 0 | 0 | 0 | 0 |
| CHMP2A | 1 | 6 | 1 | 16 |

| | | | | | |
|---|---|---|---|---|---|
| 1 | KLHL10 | 0 | 0 | 4 | 2 |
| 1 | ARIH1 | 0 | 0 | 10 | 0 |
| 0 | FRS3 | 0 | 2 | 1 | 0 |
| 0 | SQSTM1 | 7 | 1 | 16 | 4 |
| 0 | GAB1 | 3 | 2 | 1 | 0 |
| 7 | RAPSN | 2 | 1 | 0 | 0 |
| 0 | CARD9 | 1 | 4 | 6 | 2 |
| 0 | WDR5 | 3 | 2 | 32 | 2 |
| 2 | LCK | 15 | 9 | 13 | 3 |
| 1 | HCK | 6 | 6 | 8 | 2 |
| 1 | AHDC1 | 2 | 0 | 1 | 0 |
| 0 | BEND5 | 1 | 0 | 0 | 0 |
| 0 | PVRL1 | 4 | 2 | 2 | 0 |
| 0 | UNC45B | 1 | 1 | 2 | 0 |
| 0 | FGR | 9 | 9 | 11 | 2 |
| 0 | AXL | 0 | 1 | 2 | 0 |
| 0 | FGF6 | 0 | 5 | 3 | 1 |
| 0 | PI16 | 0 | 0 | 1 | 0 |
| 1 | RIT1 | 1 | 0 | 2 | 1 |
| 0 | RIT2 | 1 | 0 | 0 | 0 |
| 0 | SAMD4B | 0 | 1 | 7 | 0 |
| 0 | PSMC5 | 4 | 3 | 42 | 2 |
| 0 | ICAM3 | 2 | 3 | 1 | 2 |
| 0 | ADAP2 | 1 | 1 | 1 | 3 |
| 0 | KCTD17 | 0 | 0 | 1 | 0 |
| 0 | SLC35A4 | 0 | 0 | 3 | 0 |
| 0 | VPS28 | 1 | 2 | 16 | 1 |
| 0 | MPPED1 | 1 | 0 | 1 | 0 |
| 3 | MECP2 | 8 | 5 | 15 | 4 |
| 0 | BRMS1L | 2 | 1 | 8 | 1 |
| 1 | FBP1 | 3 | 3 | 10 | 0 |
| 0 | MLYCD | 2 | 1 | 4 | 0 |
| 0 | DNAJC5B | 0 | 0 | 5 | 1 |
| 0 | BCL2L1 | 5 | 3 | 16 | 2 |
| 0 | IGHA2 | 1 | 0 | 3 | 0 |
| 0 | FOS | 26 | 22 | 42 | 4 |
| 0 | XBP1 | 2 | 1 | 9 | 0 |
| 0 | MAFB | 3 | 3 | 4 | 0 |
| 0 | WWTR1 | 16 | 2 | 4 | 0 |
| 0 | SATB2 | 6 | 2 | 4 | 1 |
| 0 | ESRRG | 22 | 1 | 3 | 0 |
| 0 | NR4A2 | 5 | 1 | 2 | 2 |
| 0 | SP6 | 1 | 0 | 5 | 0 |
| 0 | MEIS2 | 2 | 17 | 0 | 1 |
| 0 | HAND2 | 5 | 1 | 10 | 0 |
| 0 | HEY1 | 5 | 3 | 9 | 2 |
| 0 | NFE2L1 | 1 | 1 | 4 | 0 |
| 0 | FOXP2 | 24 | 4 | 10 | 1 |
| 1 | HEY2 | 4 | 0 | 5 | 1 |
| 3 | ISX | 0 | 0 | 0 | 0 |
| 0 | OLIG3 | 3 | 1 | 2 | 0 |
| 0 | TP53 | 28 | 21 | 74 | 9 |
| 1 | ZNF423 | 3 | 0 | 1 | 0 |

| | | | | | |
|---|---|---|---|---|---|
| 0 | FOXJ1 | 4 | 3 | 3 | 0 |
| 0 | MYF6 | 3 | 5 | 8 | 0 |
| 1 | PURG | 2 | 1 | 3 | 0 |
| 0 | ZNF503 | 1 | 0 | 0 | 0 |
| 2 | SIM2 | 9 | 2 | 9 | 2 |
| 0 | CRX | 2 | 3 | 6 | 0 |
| 0 | SP8 | 7 | 2 | 2 | 0 |
| 0 | USF1 | 7 | 1 | 17 | 2 |
| 0 | NANOG | 11 | 7 | 20 | 3 |
| 0 | LPAR4 | 0 | 0 | 0 | 0 |
| 4 | MAS1 | 8 | 4 | 5 | 3 |
| 0 | GPR4 | 3 | 1 | 0 | 0 |
| 0 | ADCY9 | 3 | 4 | 8 | 2 |
| 0 | HTR2C | 4 | 3 | 3 | 0 |
| 0 | ADORA2A | 0 | 2 | 4 | 0 |
| 0 | GPBAR1 | 0 | 0 | 0 | 0 |
| 0 | GPR3 | 1 | 0 | 0 | 0 |
| 0 | VAV1 | 14 | 11 | 14 | 3 |
| 0 | MCF2L | 3 | 2 | 2 | 0 |
| 0 | TBC1D3G | 0 | 1 | 1 | 0 |
| 1 | ARHGEF2 | 1 | 3 | 3 | 1 |
| 1 | RASGRP3 | 2 | 2 | 1 | 0 |
| 0 | SPATA13 | 1 | 3 | 5 | 1 |
| 0 | IQSEC1 | 0 | 3 | 0 | 0 |
| 0 | RAPGEF4 | 11 | 2 | 4 | 1 |
| 0 | RASGRP2 | 4 | 4 | 0 | 1 |
| 1 | PRKACA | 8 | 6 | 10 | 3 |
| 0 | NF2 | 3 | 4 | 9 | 2 |
| 0 | PRKCE | 8 | 3 | 5 | 1 |
| 0 | MAP3K14 | 1 | 0 | 0 | 1 |
| 0 | KLHL3 | 0 | 0 | 3 | 0 |
| 1 | TRIM62 | 0 | 0 | 1 | 0 |
| 1 | KLHL2 | 2 | 2 | 7 | 0 |
| 0 | TRIM50 | 0 | 0 | 2 | 1 |
| 0 | CRKL | 6 | 15 | 11 | 1 |
| 0 | CRK | 12 | 13 | 12 | 2 |
| 1 | TRAF3IP2 | 1 | 0 | 1 | 0 |
| 1 | TEX11 | 3 | 0 | 1 | 0 |
| 1 | CIQA | 1 | 0 | 0 | 1 |
| 0 | SRC | 33 | 25 | 34 | 6 |
| 0 | BTK | 4 | 4 | 10 | 2 |
| 0 | LYN | 0 | 0 | 0 | 0 |
| 0 | KLHL34 | 0 | 1 | 1 | 1 |
| 1 | WDR18 | 1 | 2 | 31 | 1 |
| 0 | PCDHGB1 | 0 | 0 | 0 | 0 |
| 0 | TEKT5 | 0 | 1 | 0 | 0 |
| 0 | TYRO3 | 6 | 5 | 8 | 0 |
| 0 | FGFR2 | 16 | 12 | 12 | 3 |
| 0 | CHGA | 5 | 5 | 5 | 2 |
| 1 | IFNA10 | 0 | 1 | 0 | 0 |
| 0 | RHOBTB2 | 3 | 2 | 1 | 0 |
| 1 | SAMD4A | 1 | 0 | 9 | 0 |
| 0 | FXR2 | 2 | 1 | 7 | 1 |

| |
|---|
| 0 |
| 0 |
| 0 |
| 0 |
| 1 |
| 0 |
| 1 |
| 0 |
| 0 |
| 0 |
| 0 |
| 2 |
| 1 |
| 0 |
| 0 |
| 1 |
| 1 |
| 0 |
| 0 |
| 0 |
| 0 |
| 1 |
| 2 |
| 0 |
| 2 |
| 2 |
| 0 |
| 1 |
| 0 |
| 0 |

| | | | | |
|---|---|---|---|---|
| ATAD1 | 5 | 2 | 13 | 0 |
| F3 | 10 | 4 | 11 | 1 |
| RGS11 | 2 | 1 | 1 | 0 |
| KCTD1 | 0 | 2 | 1 | 0 |
| SLC4A2 | 1 | 1 | 0 | 0 |
| MAGEA9 | 0 | 0 | 0 | 0 |
| PPP1CA | 6 | 12 | 85 | 9 |
| EIF4H | 0 | 3 | 35 | 3 |
| TPI1 | 1 | 6 | 32 | 0 |
| NASP | 0 | 0 | 0 | 0 |
| TNFRSF13B | 1 | 0 | 2 | 0 |
| CYP2E1 | 1 | 1 | 2 | 0 |
| CCDC150 | 0 | 1 | 1 | 0 |

**Table S5. Core geneset highly connected to all screens.**

List of genes from RefSeq database with at least 10 STRING connections to each screen geneset, excluding the small geneset of shRNA screen.

| Gene | ENSP_ID | PB | ORF | dCAS9 | CRISPR | shRNA |
|---|---|---|---|---|---|---|
| CD4 | ENSP00000011653 | 18 | 46 | 19 | 45 | 8 |
| ESR1 | ENSP00000206249 | 25 | 54 | 23 | 70 | 4 |
| VCL | ENSP00000211998 | 11 | 24 | 10 | 10 | 1 |
| MAPK1 | ENSP00000215832 | 21 | 51 | 19 | 28 | 7 |
| RNASEH2A | ENSP00000221486 | 10 | 20 | 10 | 52 | 5 |
| TGFB1 | ENSP00000221930 | 23 | 43 | 18 | 33 | 5 |
| CCND1 | ENSP00000227507 | 24 | 60 | 22 | 46 | 5 |
| PXN | ENSP00000228307 | 11 | 30 | 13 | 11 | 3 |
| IFNG | ENSP00000229135 | 20 | 38 | 17 | 31 | 4 |
| GAPDH | ENSP00000229239 | 26 | 68 | 20 | 65 | 4 |
| ENO2 | ENSP00000229277 | 11 | 20 | 11 | 27 | 2 |
| MAPK14 | ENSP00000229794 | 20 | 41 | 11 | 25 | 6 |
| ENO1 | ENSP00000234590 | 11 | 15 | 11 | 45 | 4 |
| CDKN1A | ENSP00000244741 | 15 | 42 | 15 | 43 | 7 |
| RAC2 | ENSP00000249071 | 11 | 31 | 12 | 13 | 3 |
| MYOD1 | ENSP00000250003 | 16 | 36 | 12 | 22 | 4 |
| APOE | ENSP00000252486 | 13 | 26 | 11 | 18 | 2 |
| EPO | ENSP00000252723 | 12 | 28 | 13 | 21 | 3 |
| MNX1 | ENSP00000252971 | 21 | 37 | 13 | 13 | 0 |
| H3F3B | ENSP00000254810 | 18 | 40 | 11 | 55 | 9 |
| KRAS | ENSP00000256078 | 13 | 46 | 15 | 30 | 9 |
| IL6 | ENSP00000258743 | 19 | 47 | 15 | 29 | 5 |
| CDH1 | ENSP00000261769 | 23 | 46 | 16 | 31 | 2 |
| PDGFRB | ENSP00000261799 | 16 | 24 | 10 | 16 | 2 |
| N/A | ENSP00000262266 | 24 | 49 | 16 | 79 | 5 |
| MAPK3 | ENSP00000263025 | 15 | 38 | 12 | 27 | 7 |
| TYR | ENSP00000263321 | 23 | 41 | 27 | 44 | 8 |
| IL1B | ENSP00000263341 | 10 | 32 | 10 | 21 | 3 |
| PIK3CA | ENSP00000263967 | 15 | 47 | 18 | 24 | 3 |
| STAT3 | ENSP00000264657 | 18 | 44 | 13 | 25 | 4 |
| POMC | ENSP00000264708 | 15 | 36 | 12 | 17 | 4 |
| N/A | ENSP00000264998 | 13 | 32 | 10 | 23 | 1 |
| KNG1 | ENSP00000265023 | 12 | 25 | 10 | 12 | 2 |
| EGF | ENSP00000265171 | 30 | 46 | 19 | 28 | 7 |
| SRF | ENSP00000265354 | 14 | 30 | 13 | 18 | 2 |
| N/A | ENSP00000266427 | 17 | 19 | 10 | 20 | 3 |
| RB1 | ENSP00000267163 | 12 | 27 | 11 | 30 | 4 |
| CDH2 | ENSP00000269141 | 15 | 18 | 11 | 11 | 1 |
| TP53 | ENSP00000269305 | 28 | 58 | 21 | 74 | 9 |
| ERBB2 | ENSP00000269571 | 30 | 41 | 20 | 37 | 6 |
| SOD1 | ENSP00000270142 | 11 | 21 | 10 | 59 | 3 |
| AKT1 | ENSP00000270202 | 36 | 70 | 32 | 52 | 8 |
| CALM2 | ENSP00000272298 | 24 | 49 | 20 | 47 | 5 |
| FTH1 | ENSP00000273550 | 15 | 32 | 12 | 38 | 4 |
| EGFR | ENSP00000275493 | 29 | 54 | 22 | 35 | 7 |
| NOTCH1 | ENSP00000277541 | 25 | 52 | 16 | 24 | 8 |
| CD44 | ENSP00000278385 | 17 | 45 | 14 | 16 | 1 |
| N/A | ENSP00000284384 | 10 | 21 | 11 | 18 | 4 |
| SST | ENSP00000287641 | 16 | 32 | 11 | 17 | 3 |

| | | | | | | |
|---|---|---|---|---|---|---|
| PIK3CB | ENSP00000289153 | 11 | 40 | 14 | 21 | 3 |
| CALM3 | ENSP00000291295 | 26 | 49 | 20 | 38 | 4 |
| REL | ENSP00000295025 | 19 | 34 | 12 | 30 | 5 |
| ALPPL2 | ENSP00000295453 | 24 | 43 | 13 | 32 | 6 |
| ALB | ENSP00000295897 | 33 | 63 | 27 | 71 | 8 |
| SHH | ENSP00000297261 | 21 | 36 | 12 | 16 | 5 |
| NOS3 | ENSP00000297494 | 11 | 31 | 12 | 18 | 0 |
| N/A | ENSP00000299293 | 10 | 18 | 12 | 12 | 2 |
| CRK | ENSP00000300574 | 12 | 21 | 13 | 12 | 2 |
| PRL | ENSP00000302150 | 18 | 36 | 11 | 20 | 1 |
| VAV1 | ENSP00000302269 | 10 | 22 | 11 | 14 | 3 |
| IGF1 | ENSP00000302665 | 20 | 42 | 14 | 23 | 6 |
| BCR | ENSP00000303507 | 11 | 37 | 12 | 18 | 1 |
| RAC3 | ENSP00000304283 | 17 | 45 | 20 | 20 | 4 |
| FOS | ENSP00000306245 | 26 | 62 | 22 | 42 | 4 |
| CYCS | ENSP00000307786 | 10 | 41 | 11 | 46 | 4 |
| HRAS | ENSP00000309845 | 34 | 65 | 25 | 50 | 11 |
| CASP3 | ENSP00000311032 | 18 | 46 | 18 | 42 | 5 |
| LEP | ENSP00000312652 | 21 | 35 | 11 | 20 | 2 |
| CDC42 | ENSP00000314458 | 23 | 45 | 18 | 23 | 3 |
| ZHX2 | ENSP00000314709 | 21 | 37 | 16 | 21 | 1 |
| MET | ENSP00000317272 | 11 | 28 | 12 | 11 | 1 |
| HSD17B6 | ENSP00000318631 | 14 | 15 | 10 | 11 | 2 |
| POLE | ENSP00000322570 | 23 | 45 | 16 | 69 | 5 |
| DNMT3B | ENSP00000328547 | 10 | 23 | 10 | 32 | 4 |
| TSPO | ENSP00000328973 | 36 | 78 | 32 | 73 | 9 |
| ACTG1 | ENSP00000331514 | 12 | 30 | 11 | 51 | 5 |
| SMAD3 | ENSP00000332973 | 11 | 22 | 10 | 16 | 5 |
| FGF1 | ENSP00000338548 | 19 | 33 | 12 | 16 | 2 |
| GRB2 | ENSP00000339007 | 16 | 44 | 15 | 32 | 2 |
| SMAD4 | ENSP00000341551 | 16 | 34 | 11 | 30 | 3 |
| ADCY2 | ENSP00000342952 | 13 | 27 | 10 | 15 | 2 |
| PKN1 | ENSP00000343325 | 13 | 25 | 12 | 18 | 4 |
| CTNNB1 | ENSP00000344456 | 18 | 35 | 13 | 27 | 5 |
| UBC | ENSP00000344818 | 42 | 60 | 37 | 188 | 16 |
| FN1 | ENSP00000346839 | 23 | 41 | 20 | 28 | 6 |
| RET | ENSP00000347942 | 17 | 22 | 15 | 16 | 2 |
| MBP | ENSP00000348273 | 10 | 27 | 10 | 16 | 2 |
| RAC1 | ENSP00000348461 | 19 | 47 | 20 | 23 | 6 |
| INS-IGF2 | ENSP00000348986 | 38 | 69 | 30 | 72 | 10 |
| CALM1 | ENSP00000349467 | 27 | 53 | 21 | 41 | 4 |
| ACTB | ENSP00000349960 | 13 | 29 | 11 | 52 | 5 |
| BRCA1 | ENSP00000350283 | 13 | 31 | 11 | 59 | 3 |
| SRC | ENSP00000350941 | 33 | 58 | 25 | 34 | 6 |
| REV3L | ENSP00000351697 | 26 | 47 | 17 | 70 | 5 |
| PIK3CG | ENSP00000352121 | 14 | 39 | 13 | 21 | 4 |
| DNAH8 | ENSP00000352312 | 15 | 42 | 13 | 45 | 6 |
| MAPK8 | ENSP00000353483 | 15 | 31 | 11 | 21 | 2 |
| MTOR | ENSP00000354558 | 17 | 45 | 12 | 35 | 4 |
| CDKN2A | ENSP00000355153 | 14 | 40 | 11 | 35 | 5 |
| H3F3A | ENSP00000355778 | 17 | 40 | 13 | 59 | 9 |
| PARK2 | ENSP00000355865 | 15 | 37 | 14 | 25 | 5 |
| PTGS2 | ENSP00000356438 | 15 | 37 | 14 | 19 | 3 |

| | | | | | | |
|---|---|---|---|---|---|---|
| NES | ENSP00000357206 | 16 | 24 | 10 | 20 | 1 |
| FYN | ENSP00000357656 | 14 | 32 | 12 | 13 | 1 |
| N/A | ENSP00000358175 | 14 | 41 | 11 | 48 | 8 |
| NGF | ENSP00000358525 | 26 | 44 | 17 | 33 | 5 |
| NRAS | ENSP00000358548 | 14 | 42 | 15 | 36 | 9 |
| JUN | ENSP00000360266 | 27 | 62 | 25 | 51 | 5 |
| PTEN | ENSP00000361021 | 27 | 48 | 15 | 44 | 6 |
| VEGFA | ENSP00000361125 | 25 | 54 | 21 | 36 | 7 |
| CD40 | ENSP00000361359 | 10 | 29 | 10 | 18 | 3 |
| MMP9 | ENSP00000361405 | 12 | 34 | 13 | 16 | 3 |
| ALPL | ENSP00000363965 | 24 | 43 | 13 | 32 | 5 |
| MYC | ENSP00000367207 | 29 | 53 | 16 | 58 | 6 |
| CKAP2 | ENSP00000367276 | 13 | 22 | 16 | 58 | 5 |
| POLA1 | ENSP00000368349 | 23 | 52 | 15 | 73 | 8 |
| PCNA | ENSP00000368438 | 14 | 32 | 12 | 95 | 7 |
| JAK2 | ENSP00000371067 | 13 | 33 | 11 | 19 | 2 |
| ALPP | ENSP00000375881 | 25 | 45 | 14 | 33 | 6 |
| PAX3 | ENSP00000375921 | 12 | 24 | 11 | 12 | 1 |
| N/A | ENSP00000376688 | 12 | 19 | 11 | 11 | 5 |
| FGFR1 | ENSP00000380280 | 14 | 21 | 12 | 13 | 1 |
| RELA | ENSP00000384273 | 12 | 32 | 11 | 29 | 4 |
| ELAVL1 | ENSP00000385269 | 17 | 33 | 14 | 60 | 6 |
| CXCR4 | ENSP00000386884 | 10 | 33 | 10 | 18 | 3 |
| GCG | ENSP00000387662 | 11 | 36 | 10 | 12 | 2 |
| UBA52 | ENSP00000388107 | 29 | 73 | 24 | 104 | 8 |
| N/A | ENSP00000389265 | 15 | 41 | 17 | 39 | 3 |
| N/A | ENSP00000392858 | 17 | 43 | 17 | 39 | 3 |
| TNF | ENSP00000398698 | 19 | 50 | 21 | 50 | 6 |
| FGFR2 | ENSP00000410294 | 16 | 24 | 12 | 12 | 3 |
| BDNF | ENSP00000414303 | 16 | 35 | 13 | 23 | 3 |
| TGFBI | ENSP00000416330 | 15 | 36 | 15 | 15 | 1 |

**Table S6. Differentially expressed genes between PLX4720 sensitive and resistant BRAF V600E melanoma cell lines**

Shown are gene expression measured by U219 microarray. Cell lines are sorted by PLX4720 IC50 and splitted to a sensitive group an

| Gene | WM278 | A375 | WM35 | MMAC-SF | UACC-62 | IGR-37 | MEL-HO |
|---|---|---|---|---|---|---|---|
| ABTB2 | 8.2585 | 6.95618 | 7.83118 | 7.2887 | 6.14106 | 4.88887 | 5.75692 |
| ACAM | 3.18788 | 4.45323 | 2.74345 | 2.7257 | 2.83797 | 2.88925 | 2.88759 |
| ACAN | 8.71187 | 8.39678 | 5.10148 | 3.20172 | 5.95878 | 3.20097 | 3.37094 |
| ACP6 | 9.41095 | 8.56747 | 6.49242 | 7.43934 | 8.83154 | 7.59868 | 6.81361 |
| ACPL2 | 10.0686 | 11.3344 | 8.81519 | 5.98298 | 9.59488 | 7.44799 | 6.00857 |
| ACSS1 | 8.77134 | 7.11986 | 9.87193 | 7.94931 | 7.61095 | 10.3355 | 9.19028 |
| ACTA2 | 8.83244 | 8.88175 | 7.9439 | 8.31004 | 8.53396 | 2.99651 | 6.88397 |
| ACTG2 | 3.15115 | 3.03746 | 3.72235 | 3.46038 | 3.20627 | 4.17376 | 3.89697 |
| ACTN2 | 5.37916 | 5.28188 | 6.39316 | 3.18369 | 5.36094 | 3.51866 | 3.186 |
| ADAMTS5 | 3.10337 | 2.84638 | 2.75143 | 3.03019 | 2.79788 | 2.96584 | 2.82631 |
| ADORA2B | 8.52292 | 8.7806 | 7.69217 | 4.10734 | 9.41386 | 4.52739 | 7.08145 |
| ADRBK2 | 3.95695 | 3.81131 | 4.76954 | 7.7916 | 4.69764 | 8.16277 | 5.58257 |
| AGPAT9 | 8.89994 | 8.85594 | 8.72935 | 6.81138 | 7.91284 | 7.96381 | 8.51016 |
| AHCYL2 | 7.63261 | 7.51852 | 6.84801 | 7.89043 | 6.96812 | 8.63862 | 8.60324 |
| AK5 | 3.13991 | 3.46269 | 3.09688 | 4.77637 | 3.12049 | 3.24942 | 3.23995 |
| AKR1C2 | 5.92739 | 10.5279 | 3.42945 | 6.7616 | 5.13844 | 3.33419 | 3.4159 |
| ALCAM | 9.54927 | 8.45375 | 8.1764 | 3.06259 | 9.73839 | 5.4516 | 3.69971 |
| ALDH1A1 | 6.63325 | 3.70083 | 4.96361 | 9.40701 | 5.91952 | 5.87293 | 4.52941 |
| ALDH2 | 4.00529 | 6.70421 | 3.3957 | 3.90979 | 3.37327 | 4.07939 | 3.69771 |
| ALDH5A1 | 4.16672 | 6.04993 | 5.11052 | 4.04907 | 5.0507 | 6.52797 | 4.59138 |
| ALDOC | 7.99837 | 8.21539 | 3.31339 | 3.67426 | 9.86451 | 3.70414 | 8.03534 |
| AMIGO2 | 8.32555 | 7.79071 | 3.33263 | 3.69777 | 3.64606 | 3.40481 | 5.12985 |
| ANGPT1 | 9.95819 | 6.19931 | 6.45165 | 3.27577 | 9.15921 | 3.62 | 3.28766 |
| ANGPTL2 | 4.22583 | 6.68216 | 6.45826 | 8.14525 | 8.23181 | 8.29733 | 7.07279 |
| ANGPTL7 | 5.04071 | 4.26361 | 3.56483 | 3.63993 | 5.48637 | 3.52402 | 3.35466 |
| ANKRD12 | 6.63894 | 6.38733 | 6.18818 | 6.10688 | 6.84928 | 3.83643 | 7.36904 |
| ANLN | 7.49928 | 6.20526 | 7.80264 | 7.17468 | 8.49829 | 6.26324 | 8.21604 |
| ANO4 | 3.14077 | 4.65371 | 2.80431 | 6.69521 | 3.7704 | 2.94063 | 6.97283 |
| ANP32E | 3.89364 | 5.70769 | 5.34688 | 3.70768 | 6.99183 | 4.60904 | 6.67464 |
| ANPEP | 3.53035 | 5.32474 | 3.43121 | 3.59004 | 3.56258 | 3.31925 | 3.35573 |
| APOC2 | 3.07412 | 3.26914 | 10.7122 | 4.12368 | 4.06168 | 8.96369 | 3.57575 |
| APOD | 6.65569 | 3.59603 | 11.8712 | 6.4216 | 8.98616 | 7.25809 | 10.8206 |
| ARHGAP6 | 5.41915 | 5.39492 | 3.33287 | 4.18929 | 8.47056 | 3.0214 | 3.01554 |
| ARNT2 | 9.7775 | 10.7444 | 9.35187 | 8.89063 | 9.12655 | 9.30936 | 8.26186 |
| ASPM | 6.98449 | 8.28634 | 6.90651 | 6.15908 | 8.34053 | 5.86312 | 7.67716 |
| ATRX | 7.14362 | 7.73714 | 4.7361 | 6.27761 | 8.14968 | 4.53632 | 5.59361 |
| ATRX \|\| LOC65 | 8.57129 | 8.41176 | 6.44951 | 7.7714 | 8.18056 | 4.56441 | 6.03126 |
| AZGP1 | 6.69023 | 7.23953 | 7.93646 | 3.30557 | 5.59347 | 3.88101 | 3.58969 |
| AZK* | 4.69982 | 4.75018 | 5.25072 | 4.62339 | 4.79145 | 4.62558 | 4.45251 |
| B3GALNT1 | 4.04334 | 3.20585 | 3.85329 | 3.47361 | 3.34501 | 3.55427 | 6.86144 |
| BAALC | 4.50078 | 4.67595 | 3.43143 | 3.38709 | 8.75524 | 3.19337 | 5.75288 |
| BASP1 | 4.49866 | 3.24208 | 2.97687 | 3.30284 | 3.28333 | 3.09031 | 2.95524 |
| BAT2L2 | 7.19991 | 7.4202 | 6.56073 | 6.9273 | 8.04149 | 4.44973 | 7.20418 |
| BCAN | 2.76711 | 2.94836 | 3.99615 | 7.34395 | 2.80579 | 8.34735 | 4.19325 |
| BCL2A1 | 7.58768 | 6.43054 | 10.2834 | 9.19059 | 9.70207 | 9.50122 | 9.80269 |
| BDKRB1 | 2.93496 | 2.93949 | 2.79308 | 2.82247 | 2.69225 | 2.83255 | 2.82816 |
| BDNF | 3.7972 | 2.97136 | 3.06216 | 2.78025 | 3.00673 | 3.09819 | 3.10408 |
| BEST1 | 3.71223 | 4.43004 | 6.20468 | 8.20479 | 4.0061 | 8.03498 | 6.11141 |
| BEX1 | 5.61204 | 3.22091 | 3.1932 | 8.06528 | 4.85987 | 4.28001 | 3.31979 |

| | | | | | | | |
|---|---|---|---|---|---|---|---|
| BFSP1 | 9.70302 | 8.15319 | 4.4421 | 4.5402 | 6.63555 | 4.83462 | 3.25423 |
| BHLHE41 | 7.07243 | 4.44272 | 9.35822 | 9.93152 | 6.36853 | 9.59543 | 9.79668 |
| BST2 | 11.1036 | 6.80162 | 10.7984 | 12.0148 | 10.8278 | 6.66044 | 10.7598 |
| BTBD10 | 6.64637 | 5.83203 | 6.50463 | 7.48965 | 6.73706 | 6.53149 | 6.78458 |
| BTBD16 | 8.75242 | 6.28669 | 4.05196 | 4.14529 | 5.13771 | 3.19654 | 3.16838 |
| BTG2 | 4.50032 | 6.81643 | 5.52466 | 6.1254 | 6.77263 | 4.91901 | 6.09264 |
| BUB1 | 6.21632 | 6.09707 | 7.36186 | 4.97389 | 7.83099 | 7.76724 | 8.20643 |
| C10orf90 | 9.2051 | 8.06452 | 6.87362 | 7.4564 | 7.21806 | 5.80602 | 6.48847 |
| C14orf106 | 3.22874 | 3.80551 | 4.18406 | 3.06798 | 5.61587 | 2.85805 | 5.06307 |
| C14orf145 | 4.17577 | 4.00108 | 5.53935 | 4.43349 | 5.40144 | 4.25241 | 5.61117 |
| C1orf59 | 3.62236 | 4.75536 | 3.8645 | 3.99349 | 3.42074 | 4.42842 | 3.34784 |
| C1S | 10.9289 | 3.7252 | 3.16441 | 9.27198 | 5.55986 | 4.66976 | 3.24498 |
| C22orf9 | 6.42482 | 5.64464 | 9.79125 | 7.38687 | 7.369 | 8.41734 | 9.32044 |
| C3orf70 | 9.25916 | 5.56992 | 7.18038 | 4.83969 | 7.94141 | 7.62647 | 6.99592 |
| C4orf7 | 10.0105 | 10.4315 | 2.79988 | 2.98279 | 2.96576 | 3.06316 | 3.1166 |
| C5orf62 | 3.47187 | 3.30335 | 3.53146 | 3.7237 | 7.70414 | 3.08762 | 3.57819 |
| C6orf167 | 4.72609 | 4.78525 | 5.39117 | 3.92472 | 5.80863 | 5.58321 | 5.59 |
| C6orf192 | 7.90293 | 8.80532 | 10.0247 | 9.94034 | 6.9973 | 9.47323 | 8.01852 |
| CACNA1A | 3.29201 | 5.29593 | 3.20028 | 3.34105 | 3.07724 | 5.96721 | 3.54277 |
| CAPG | 3.7265 | 8.6782 | 10.8589 | 11.2194 | 5.89172 | 11.9379 | 12.1787 |
| CAPN3 | 5.29114 | 4.87662 | 8.45018 | 11.0911 | 8.09062 | 11.4542 | 11.5799 |
| CASP8AP2 | 6.70213 | 7.20518 | 7.68547 | 6.65981 | 6.92198 | 5.23884 | 7.24925 |
| CCDC88A | 5.96478 | 3.39676 | 3.62218 | 3.87 | 5.27772 | 3.22674 | 6.12621 |
| CD200 | 9.931 | 7.96687 | 9.95917 | 7.58754 | 9.19153 | 9.63867 | 7.28623 |
| CD59 | 7.79907 | 4.40181 | 6.03293 | 5.2168 | 4.61485 | 4.14039 | 5.34081 |
| CD74 | 9.97148 | 10.3956 | 7.92009 | 10.4758 | 8.51839 | 2.932 | 3.91288 |
| CD9 | 10.2613 | 9.86521 | 9.3397 | 10.027 | 8.09842 | 10.3043 | 7.8994 |
| CDH1 | 3.01485 | 2.79166 | 3.14161 | 9.01171 | 2.82625 | 4.69893 | 9.33274 |
| CDH13 | 8.78142 | 11.2208 | 3.04523 | 3.14919 | 5.95074 | 2.99043 | 2.84878 |
| CEACAM1 | 3.27589 | 3.38443 | 7.06754 | 10.0263 | 6.65833 | 8.95768 | 8.86807 |
| CENPE | 6.06774 | 5.7465 | 5.83922 | 4.27124 | 6.63911 | 3.37695 | 7.90655 |
| CENPF | 5.36456 | 5.93614 | 5.54656 | 4.3 | 7.86143 | 3.52229 | 7.33593 |
| CENPV | 3.57598 | 5.73647 | 3.75467 | 3.95819 | 3.44212 | 3.97649 | 3.86378 |
| CEP152 | 6.71089 | 6.75452 | 6.52932 | 7.11797 | 6.92122 | 5.93468 | 7.51277 |
| CEP55 | 6.60808 | 7.27837 | 7.92757 | 7.06607 | 7.35206 | 6.80759 | 8.41586 |
| CFH | 5.75758 | 3.28432 | 2.68159 | 3.007 | 5.00843 | 2.78859 | 2.63945 |
| CFH \|\| CFHR1 | 6.15574 | 3.74464 | 3.66674 | 3.35437 | 5.47362 | 3.28736 | 3.36716 |
| CHRDL1 | 7.94198 | 10.7094 | 3.22856 | 3.55317 | 9.62219 | 3.37363 | 3.35819 |
| CKAP2L | 6.52535 | 6.22612 | 7.36646 | 4.94596 | 7.22979 | 5.10999 | 6.58226 |
| CKB | 3.49231 | 6.74947 | 4.81939 | 3.98592 | 8.16507 | 5.87093 | 6.72897 |
| CKS1B | 5.87407 | 8.18789 | 8.14767 | 6.70395 | 7.67537 | 8.20869 | 8.65142 |
| CLCN5 | 6.29997 | 5.83349 | 6.36084 | 7.05075 | 6.54958 | 6.80208 | 4.73639 |
| CMBL | 8.66665 | 9.11423 | 5.99201 | 8.48175 | 8.89135 | 3.42697 | 5.82216 |
| CNIH3 | 4.00276 | 4.9542 | 8.8409 | 4.59223 | 7.49959 | 8.47374 | 8.20309 |
| CNTN3 | 2.90583 | 8.26627 | 2.92964 | 7.45796 | 3.30658 | 2.74056 | 2.84308 |
| COL14A1 | 3.05406 | 2.84752 | 3.02959 | 2.96395 | 4.41844 | 3.19046 | 7.38014 |
| COL16A1 | 8.45944 | 10.5734 | 7.58258 | 8.6678 | 9.0507 | 7.26527 | 3.8768 |
| COL22A1 | 8.66164 | 4.34814 | 3.11662 | 7.69247 | 5.35972 | 3.28457 | 3.19104 |
| COL3A1 | 2.86008 | 2.72116 | 2.80993 | 2.78973 | 3.30968 | 2.78612 | 2.83722 |
| COL4A5 | 8.55277 | 6.69834 | 4.40207 | 6.37792 | 6.87841 | 4.28698 | 3.17089 |
| COL5A1 | 4.59344 | 5.73455 | 3.5195 | 3.51963 | 6.57251 | 3.54382 | 3.93018 |
| COL6A3 | 3.15489 | 2.99285 | 2.96598 | 2.91236 | 5.83356 | 2.89954 | 2.97774 |
| COL9A1 | 5.76536 | 4.20485 | 3.27201 | 3.3179 | 7.40931 | 2.83235 | 3.14028 |

| | | | | | | | |
|---|---|---|---|---|---|---|---|
| COLEC10 | 2.91222 | 2.76455 | 2.95429 | 3.07438 | 2.92252 | 2.95743 | 2.84227 |
| COLEC12 | 6.28786 | 7.55537 | 3.67317 | 3.72215 | 4.38605 | 3.79397 | 3.87096 |
| COPZ2 | 8.51268 | 7.74166 | 8.56162 | 8.87813 | 8.45581 | 7.60348 | 4.69078 |
| CORO2B | 3.71542 | 4.2035 | 7.18068 | 6.99951 | 4.69919 | 5.25999 | 3.94661 |
| CPM | 7.33507 | 5.1515 | 6.81795 | 7.97405 | 7.26098 | 4.23421 | 6.80248 |
| CPN1 | 9.59088 | 8.81121 | 9.30309 | 9.60976 | 6.83171 | 7.34313 | 8.8071 |
| CPPED1 | 5.39229 | 6.17408 | 7.87582 | 7.6741 | 7.6949 | 7.21121 | 8.25937 |
| CPT1C | 3.91753 | 3.74113 | 6.49772 | 3.15444 | 7.42656 | 7.38568 | 3.33224 |
| CPXM2 | 3.22319 | 3.43267 | 3.32067 | 3.40334 | 3.09419 | 3.32112 | 3.33276 |
| CRYAB | 4.30404 | 5.45491 | 8.59867 | 12.592 | 10.1962 | 8.37825 | 4.43509 |
| CRYL1 | 9.79107 | 9.1526 | 9.22477 | 8.85883 | 7.10864 | 9.81359 | 7.06585 |
| CSAG3 || CSAG | 7.82768 | 10.0585 | 4.90049 | 3.11681 | 8.47324 | 3.82554 | 4.40234 |
| CSPG4 | 8.03189 | 8.62419 | 8.01748 | 3.25801 | 6.19523 | 8.40574 | 6.93866 |
| CT45A3 || CT45 | 6.36594 | 11.525 | 3.18581 | 2.92324 | 11.966 | 3.06696 | 3.18603 |
| CTAG1B || CTA | 3.40597 | 10.1414 | 3.43312 | 3.34419 | 3.87797 | 3.42076 | 3.56846 |
| CTGF | 10.0394 | 4.68981 | 6.77341 | 4.82101 | 11.1495 | 4.07671 | 4.71415 |
| CTSZ | 10.3575 | 9.14078 | 10.2906 | 10.9196 | 9.7428 | 10.8162 | 7.53611 |
| CUBN | 4.79598 | 3.28474 | 3.66733 | 4.0425 | 8.2404 | 3.60009 | 3.39272 |
| CXCL1 | 11.8752 | 9.23278 | 7.44778 | 8.543 | 7.17706 | 7.17594 | 7.73468 |
| CYB5R2 | 9.36771 | 3.63469 | 2.90671 | 3.80716 | 5.38665 | 3.72244 | 7.13055 |
| CYP27A1 | 9.27298 | 6.37773 | 8.47124 | 8.42666 | 8.51493 | 8.67588 | 8.52683 |
| CYR61 | 7.73891 | 7.68968 | 3.05013 | 4.77523 | 8.99128 | 3.45685 | 3.39514 |
| DAAM2 | 5.94765 | 7.86654 | 9.21862 | 8.11483 | 8.24382 | 7.29427 | 7.91841 |
| DCT | 3.24345 | 4.23577 | 12.3174 | 12.2112 | 5.66867 | 12.742 | 12.3519 |
| DDX43 | 3.28986 | 3.41495 | 3.51773 | 3.36768 | 6.05977 | 5.35709 | 3.13397 |
| DEK | 6.799 | 6.87081 | 8.18305 | 7.24214 | 6.61224 | 5.51318 | 8.11526 |
| DEPDC1 | 7.09128 | 7.21381 | 7.54891 | 7.03151 | 8.98856 | 6.81584 | 9.00233 |
| DEPDC6 | 4.11972 | 6.87314 | 6.08783 | 5.54354 | 7.80412 | 5.42843 | 2.97774 |
| DHRS3 | 11.0265 | 11.0344 | 4.52268 | 3.79381 | 5.35258 | 3.54983 | 4.64069 |
| DIAPH3 | 5.86756 | 4.73833 | 5.9081 | 5.25106 | 6.42906 | 7.09756 | 6.62823 |
| DIP2B | 3.99579 | 5.67508 | 7.14492 | 4.29341 | 7.01543 | 7.05636 | 7.73963 |
| DLGAP5 | 6.72183 | 7.11712 | 7.92136 | 5.45176 | 8.58858 | 6.74591 | 9.00498 |
| DLL3 | 5.81574 | 6.31289 | 7.18968 | 7.7114 | 7.04397 | 7.99427 | 10.0974 |
| DNAH9 | 3.50369 | 6.25806 | 3.28645 | 3.42268 | 7.50178 | 3.20774 | 3.09795 |
| DNER | 7.22334 | 3.08092 | 3.66982 | 3.30979 | 6.90783 | 6.79859 | 3.69164 |
| DSE | 5.60272 | 4.52637 | 4.70059 | 3.87312 | 4.17841 | 5.38428 | 3.58803 |
| DST | 7.27013 | 4.499 | 4.96231 | 4.61669 | 7.88936 | 2.9649 | 6.46381 |
| DTL | 4.07681 | 5.75452 | 6.21837 | 5.65629 | 6.19009 | 6.12586 | 5.72952 |
| DUSP23 | 7.21272 | 8.85671 | 7.77647 | 8.20247 | 8.93927 | 8.98477 | 8.67244 |
| E2F7 | 5.5398 | 6.48083 | 7.48408 | 5.91257 | 7.68943 | 7.54111 | 7.11094 |
| EBF3 | 7.84984 | 9.4417 | 3.34576 | 3.41541 | 6.40923 | 3.39336 | 5.8258 |
| ECT2 | 5.09461 | 5.8244 | 6.89295 | 5.46543 | 7.01847 | 6.56713 | 7.38559 |
| EDA2R | 6.71997 | 4.4885 | 5.74676 | 6.71248 | 6.79049 | 3.14904 | 5.20852 |
| EDIL3 | 9.15211 | 9.3249 | 3.79658 | 4.31683 | 7.90051 | 3.35354 | 3.12372 |
| EDNRB | 10.5342 | 9.68345 | 9.67577 | 10.2096 | 8.37785 | 10.4271 | 5.98769 |
| EFHD1 | 3.33683 | 3.41065 | 3.50058 | 6.94332 | 3.62544 | 4.55856 | 3.60595 |
| EFR3B | 7.9205 | 4.68665 | 7.11725 | 8.31927 | 7.67186 | 8.48554 | 6.24992 |
| EGF | 5.68051 | 5.03226 | 3.03231 | 3.20521 | 4.77035 | 3.0692 | 7.25092 |
| EGFR | 4.52782 | 4.06115 | 3.06332 | 3.79716 | 4.13417 | 3.81922 | 3.19431 |
| EGR3 | 8.13441 | 8.71783 | 9.25807 | 5.35433 | 8.41617 | 7.74522 | 6.69548 |
| EIF5B | 9.14979 | 8.98676 | 9.17027 | 8.688 | 10.0265 | 5.48233 | 10.2244 |
| ELOVL2 | 7.08022 | 4.31393 | 6.85441 | 6.64995 | 7.60933 | 4.06327 | 8.20773 |
| ENPP2 | 10.9252 | 10.5357 | 7.1856 | 8.77297 | 8.8502 | 5.35802 | 4.72248 |

| | | | | | | | |
|---|---|---|---|---|---|---|---|
| ENSG00000188( | 6.7443 | 6.97273 | 7.05022 | 7.38575 | 8.09254 | 8.05461 | 7.05203 |
| EPDR1 | 7.89193 | 6.61998 | 8.75497 | 10.1977 | 9.59719 | 9.65948 | 8.27635 |
| EPHA3 | 8.89085 | 8.89312 | 8.37638 | 8.7888 | 9.77779 | 6.40354 | 3.10709 |
| EPHX2 | 4.15671 | 4.52593 | 7.36117 | 4.33785 | 7.38222 | 7.46357 | 6.10415 |
| ERBB3 | 10.4587 | 10.9588 | 9.62403 | 10.0861 | 8.70034 | 9.32117 | 9.37594 |
| EZR | 5.62071 | 6.54057 | 7.76934 | 4.30993 | 6.54473 | 4.2741 | 9.06189 |
| FABP7 | 10.2424 | 7.78532 | 7.48172 | 3.05605 | 10.384 | 9.12176 | 10.971 |
| FAM101B | 6.74816 | 5.86912 | 5.36101 | 9.46461 | 7.81191 | 9.69306 | 7.47738 |
| FAM129A | 9.42357 | 7.42033 | 3.24098 | 2.92717 | 7.68298 | 3.58135 | 7.89526 |
| FAM134B | 5.76527 | 8.80648 | 6.55754 | 8.2606 | 8.10079 | 5.34123 | 5.82389 |
| FAM167A | 4.02593 | 3.23119 | 4.15102 | 3.6272 | 3.23568 | 6.65581 | 3.46577 |
| FAM198B | 8.78087 | 3.74976 | 5.64849 | 7.90739 | 7.47411 | 4.02917 | 7.15791 |
| FAP | 8.25166 | 5.53977 | 9.10307 | 3.17341 | 7.70119 | 5.78098 | 2.91244 |
| FARP1 | 8.5987 | 8.55017 | 7.05735 | 6.52361 | 7.75353 | 8.59463 | 8.09797 |
| FARP2 | 7.31427 | 4.83249 | 6.62465 | 5.85206 | 7.31632 | 6.14267 | 4.73774 |
| FBXL16 | 5.01107 | 3.6577 | 6.76036 | 6.41456 | 3.9962 | 8.03478 | 3.39679 |
| FCGR2A | 9.43208 | 8.09845 | 5.0792 | 5.28395 | 5.65032 | 5.00254 | 6.23128 |
| FCGR2A \|\| FCG | 10.374 | 9.25131 | 6.79357 | 4.86487 | 7.9147 | 5.84435 | 8.48444 |
| FCGR2B | 4.33327 | 3.96414 | 5.19588 | 5.51775 | 5.5307 | 7.50104 | 5.18808 |
| FCRLA | 10.9185 | 6.61194 | 8.72918 | 6.76415 | 7.10649 | 5.69211 | 6.84616 |
| FEZ1 | 9.11101 | 9.06637 | 7.81907 | 4.34845 | 5.15374 | 5.34793 | 3.1419 |
| FLRT2 | 4.31301 | 4.80811 | 4.37102 | 5.29665 | 5.80144 | 7.28454 | 3.24982 |
| FOSL2 | 4.98407 | 4.6102 | 4.29695 | 4.94368 | 5.48365 | 7.61478 | 5.32722 |
| FREM2 | 8.85115 | 4.98277 | 6.29658 | 3.47723 | 6.98666 | 3.20013 | 2.96479 |
| FST | 11.0348 | 11.3215 | 8.69173 | 9.12016 | 8.06207 | 5.57834 | 4.36361 |
| FSTL5 | 4.1846 | 2.79585 | 3.10348 | 3.13987 | 3.22793 | 3.08836 | 3.41364 |
| FXYD3 | 8.63325 | 11.0125 | 11.0367 | 12.0918 | 9.9954 | 3.82579 | 10.9336 |
| GAB2 | 6.72113 | 5.45718 | 7.25181 | 7.54197 | 5.80175 | 6.32579 | 7.32354 |
| GALC | 4.36959 | 6.89079 | 4.2893 | 4.23594 | 7.37033 | 4.26799 | 3.80889 |
| GALNT5 | 3.05097 | 4.46112 | 3.54826 | 3.2365 | 8.24001 | 2.68633 | 3.22285 |
| GAPDHS | 2.83354 | 3.07499 | 9.34556 | 9.17373 | 7.6913 | 8.49186 | 8.4079 |
| GAS1 | 8.46387 | 8.16134 | 7.75869 | 9.12545 | 7.21768 | 8.72119 | 6.49062 |
| GAS7 | 11.0433 | 10.4859 | 10.2613 | 10.0472 | 11.1713 | 9.14848 | 7.68787 |
| GBP2 | 8.25999 | 9.03383 | 3.67525 | 6.90502 | 5.76995 | 3.72269 | 4.63558 |
| GDF11 | 6.18872 | 7.4789 | 7.24934 | 8.0857 | 6.98717 | 5.70325 | 7.19887 |
| GDF15 | 9.13398 | 6.66756 | 12.3635 | 12.2095 | 10.9599 | 10.2916 | 12.4294 |
| GHR | 8.30979 | 3.70618 | 4.21987 | 7.7356 | 8.35882 | 4.31125 | 7.79015 |
| GIMAP2 | 7.8123 | 6.70806 | 7.24916 | 5.39763 | 3.60328 | 7.31822 | 6.96705 |
| GLIPR1 | 5.6884 | 4.21662 | 3.41968 | 3.38207 | 6.64966 | 3.61606 | 4.95529 |
| GNG2 | 11.0585 | 10.2898 | 9.27022 | 9.2262 | 10.9281 | 7.10288 | 7.7318 |
| GNG7 | 9.32021 | 8.19211 | 8.81876 | 8.25803 | 6.63315 | 8.50392 | 7.10836 |
| GOLGA4 | 9.17658 | 9.63823 | 9.29486 | 9.76025 | 9.07332 | 4.0244 | 10.3763 |
| GPC4 | 6.13656 | 4.08221 | 2.58117 | 7.1191 | 7.20619 | 5.80731 | 2.88758 |
| GPC6 | 10.4763 | 9.51948 | 7.93629 | 4.26213 | 9.17102 | 3.06329 | 5.21386 |
| GPM6B | 4.63388 | 3.67402 | 10.505 | 10.1151 | 9.72632 | 10.3093 | 10.0031 |
| GPNMB | 11.091 | 10.5653 | 12.1456 | 11.7434 | 10.8869 | 12.0351 | 11.7226 |
| GPR155 | 9.12864 | 4.80143 | 7.91204 | 4.8487 | 5.93714 | 3.76126 | 4.94471 |
| GPR56 | 6.6263 | 9.24391 | 10.2761 | 11.2101 | 10.6092 | 11.7598 | 11.4584 |
| GRASP | 5.38996 | 4.63733 | 5.52585 | 5.54774 | 6.92857 | 5.77449 | 6.14258 |
| GSTT1 | 7.14256 | 7.27433 | 3.84132 | 6.35991 | 3.59854 | 3.72139 | 7.865 |
| HAPLN1 | 11.8269 | 11.7201 | 3.18331 | 3.26337 | 3.49971 | 2.983 | 3.10383 |
| HIF1A | 6.76426 | 6.98475 | 6.07266 | 5.9177 | 8.76793 | 5.59621 | 8.23601 |
| HLA-B | 11.577 | 11.9669 | 9.78573 | 11.3618 | 10.1232 | 10.8617 | 9.91742 |

| | | | | | | | |
|---|---|---|---|---|---|---|---|
| HLA-DMA | 8.95121 | 8.85947 | 8.40917 | 9.60448 | 8.88225 | 6.40647 | 4.55012 |
| HLA-DMB | 7.33849 | 7.16351 | 6.15802 | 8.07775 | 7.15028 | 3.98783 | 4.20666 |
| HLA-DPA1 | 12.4631 | 12.2986 | 11.9773 | 12.6202 | 11.5597 | 6.72107 | 7.22228 |
| HLA-DPB1 | 9.41197 | 10.356 | 8.54691 | 9.21075 | 8.22904 | 3.40147 | 7.78899 |
| HLA-DQA1 | 7.49001 | 3.50269 | 3.97706 | 8.83535 | 8.8891 | 3.49134 | 4.39229 |
| HLA-DQB1 | 7.14931 | 3.74978 | 3.84551 | 7.35668 | 7.52491 | 3.28378 | 5.67565 |
| HLA-DRA | 11.8541 | 12.0907 | 10.748 | 12.0841 | 11.4266 | 3.19817 | 5.90689 |
| HLA-DRB1 | 10.6966 | 11.0218 | 9.15134 | 11.0163 | 10.3366 | 3.51354 | 3.68956 |
| HLA-DRB4 | 10.4548 | 10.1883 | 8.03572 | 10.6093 | 3.65188 | 2.92796 | 3.01658 |
| HLA-DRB5 | 3.39446 | 3.42921 | 7.79533 | 9.34126 | 3.58529 | 3.38875 | 3.21497 |
| HMMR | 6.33502 | 6.30147 | 8.09383 | 6.0027 | 7.21377 | 5.65856 | 8.17419 |
| HOXA11 | 2.83026 | 3.19422 | 3.41029 | 3.30379 | 2.90892 | 4.07775 | 3.378 |
| HOXB13 | 6.03047 | 5.67702 | 3.61399 | 5.48264 | 4.81909 | 7.07324 | 3.3764 |
| HOXC13 | 6.5969 | 7.35954 | 5.34774 | 4.92421 | 5.6298 | 5.43004 | 5.84327 |
| HSPA12A | 6.58806 | 6.27427 | 7.57561 | 8.32983 | 7.33291 | 8.22207 | 6.64875 |
| HSPA1A | 10.5719 | 10.4154 | 5.02797 | 11.2421 | 9.62463 | 9.63324 | 3.14634 |
| HSPA2 | 4.91636 | 4.44612 | 3.97983 | 7.99254 | 8.94519 | 4.8843 | 3.80955 |
| HTRA1 | 10.4346 | 10.0757 | 9.96543 | 8.89764 | 9.99116 | 9.89805 | 8.96658 |
| ICAM1 | 8.97172 | 8.61982 | 5.9736 | 7.48445 | 8.43582 | 4.45102 | 6.88071 |
| ID3 | 8.11235 | 10.0251 | 7.1532 | 3.84342 | 8.25212 | 6.43801 | 7.94775 |
| IFITM1 | 11.3638 | 9.44116 | 10.6245 | 9.86362 | 8.83455 | 6.257 | 5.27318 |
| IFITM2 | 11.4223 | 10.9653 | 10.6969 | 10.5839 | 8.60045 | 5.77104 | 4.79648 |
| IFT74 | 7.04393 | 7.95367 | 4.50187 | 5.21523 | 6.86868 | 4.10484 | 6.47166 |
| IGFBP3 | 11.4146 | 8.02571 | 5.87256 | 7.02417 | 11.9363 | 3.66224 | 4.32415 |
| IL13RA2 | 10.5691 | 12.0612 | 3.21809 | 5.12349 | 12.2498 | 3.14391 | 2.84758 |
| IL24 | 3.08056 | 9.66779 | 3.3967 | 3.00533 | 3.04748 | 3.4094 | 3.07783 |
| IL31RA | 3.52821 | 3.89867 | 3.40543 | 3.13573 | 3.44389 | 3.47999 | 3.4982 |
| IL6 | 5.1416 | 2.99867 | 3.0643 | 2.98601 | 3.16025 | 3.61342 | 3.47614 |
| INSR | 4.32033 | 6.30375 | 5.57455 | 5.35671 | 5.55351 | 6.51458 | 5.10073 |
| IRS1 | 6.15054 | 3.94957 | 3.74401 | 5.91552 | 6.53845 | 4.88442 | 3.79272 |
| IRS2 | 8.72457 | 10.0775 | 8.99573 | 9.37897 | 8.16947 | 9.46419 | 7.0309 |
| ITGA1 | 3.7037 | 4.17417 | 3.76478 | 3.1384 | 3.24215 | 2.96908 | 3.00828 |
| ITGA10 | 7.88013 | 8.34802 | 3.45716 | 3.58745 | 7.81581 | 2.75924 | 3.14076 |
| ITGB1 | 6.72458 | 6.67689 | 5.48379 | 6.16284 | 8.7479 | 7.0183 | 6.32717 |
| ITGB3 | 8.55881 | 9.17266 | 8.41838 | 8.39808 | 8.04767 | 4.12305 | 7.50829 |
| ITGB8 | 9.69041 | 8.54185 | 8.94081 | 8.46326 | 8.93468 | 8.02822 | 5.73062 |
| ITIH5 | 8.2946 | 8.89647 | 8.76466 | 3.97291 | 10.3103 | 7.05768 | 3.44529 |
| ITIH5L | 7.28066 | 5.63953 | 4.65666 | 3.26742 | 5.99431 | 3.05487 | 3.07327 |
| ITPRIP | 5.66787 | 6.00051 | 4.73971 | 3.6776 | 3.24742 | 7.62524 | 3.9167 |
| JPH1 | 6.32535 | 7.41999 | 7.51281 | 7.77178 | 8.04187 | 6.68668 | 3.38582 |
| KAT2B | 9.80911 | 8.91353 | 9.91716 | 9.33291 | 8.91915 | 7.84707 | 8.12583 |
| KCNE4 | 5.08177 | 3.21391 | 5.79649 | 6.10336 | 7.37134 | 7.30359 | 3.67781 |
| KIF14 | 4.5899 | 5.35045 | 3.63428 | 3.54917 | 6.64965 | 4.07615 | 5.83779 |
| KIF18A | 4.04473 | 4.52292 | 5.56685 | 5.15207 | 5.77526 | 3.84461 | 6.87459 |
| KRT27 | 2.80979 | 2.94292 | 4.59038 | 6.89435 | 3.01313 | 3.66413 | 5.01075 |
| KTN1 | 7.53834 | 7.81197 | 6.66464 | 6.61345 | 8.52195 | 3.84438 | 8.35326 |
| LAMA4 | 8.17852 | 6.06337 | 6.94651 | 5.70602 | 11.0241 | 4.72914 | 5.77547 |
| LAPTM5 | 3.51174 | 3.06184 | 3.29591 | 3.23649 | 3.3581 | 3.38719 | 3.51223 |
| LARGE | 8.14894 | 5.76041 | 9.15106 | 8.07551 | 7.16709 | 6.94056 | 7.54711 |
| LASS1 | 7.3262 | 7.78585 | 6.05844 | 6.43995 | 7.12849 | 7.09465 | 5.49381 |
| LCN2 | 9.61713 | 3.26368 | 3.11138 | 3.60339 | 2.80589 | 2.63831 | 2.99407 |
| LOC100131392 | 10.4376 | 3.69573 | 10.3885 | 3.73793 | 5.61913 | 3.51266 | 5.28663 |
| LOC100133678 | 7.09166 | 7.74947 | 4.0924 | 8.57221 | 6.13722 | 3.61694 | 3.73795 |

| | | | | | | | |
|---|---|---|---|---|---|---|---|
| LOC100290966 | 10.7879 | 10.6532 | 8.74912 | 10.9119 | 10.8787 | 3.68707 | 3.87222 |
| LOC100292463 | 9.78709 | 7.50606 | 12.8309 | 12.7433 | 11.8545 | 11.066 | 13.1367 |
| LOC100294036 | 10.7684 | 10.6723 | 8.69547 | 10.7333 | 7.09521 | 3.29649 | 3.99296 |
| LOC100294224 | 9.75045 | 10.8107 | 4.17742 | 10.7549 | 8.74026 | 3.54668 | 3.82531 |
| LOC387763 | 5.19059 | 4.99848 | 8.05677 | 8.68571 | 4.05842 | 9.73951 | 8.99904 |
| LOC399940 \|\| L | 8.38804 | 6.93041 | 7.83 | 6.9742 | 7.8345 | 4.33838 | 6.17677 |
| LOC440459 \|\| S | 4.12653 | 3.61908 | 11.2809 | 8.97603 | 2.88171 | 10.2678 | 9.99998 |
| LOXL4 | 4.92057 | 12.5319 | 3.22784 | 3.0967 | 3.04986 | 3.08328 | 3.3756 |
| LPCAT2 | 4.90372 | 7.10045 | 8.06784 | 5.61712 | 8.43621 | 6.82994 | 2.87666 |
| LPXN | 7.70315 | 7.15236 | 8.13357 | 8.51731 | 8.03013 | 6.7628 | 9.92905 |
| LUM | 2.57936 | 2.66148 | 2.82469 | 7.48375 | 7.68492 | 5.99651 | 9.84536 |
| LY96 | 6.40057 | 3.38819 | 3.64913 | 8.64233 | 7.87615 | 7.8856 | 7.29646 |
| MAF | 4.43373 | 8.15023 | 6.53257 | 7.92911 | 7.59012 | 5.86276 | 6.04018 |
| MAGEA2B \|\| M | 7.60542 | 9.93671 | 5.46247 | 3.27391 | 8.95801 | 3.40594 | 5.78601 |
| MAGEA3 \|\| MA | 11.6263 | 11.8163 | 10.8929 | 4.38905 | 11.7652 | 10.6778 | 10.4305 |
| MAGEA4 | 3.54131 | 11.1031 | 3.6012 | 3.59773 | 10.9282 | 3.32648 | 4.08073 |
| MAGEB2 | 3.02665 | 2.99696 | 3.04199 | 3.01193 | 2.98639 | 2.91282 | 4.26483 |
| MANSC1 | 6.41499 | 6.91894 | 4.32772 | 4.4275 | 4.45492 | 3.27867 | 4.86765 |
| MCF2L | 9.54354 | 7.06026 | 6.58526 | 5.32643 | 3.74111 | 8.24925 | 7.56839 |
| MCOLN2 | 6.81801 | 6.57822 | 7.24833 | 6.75313 | 6.04393 | 7.10417 | 7.40506 |
| MCTP2 | 3.65464 | 2.65991 | 3.05937 | 3.69185 | 2.99308 | 3.10973 | 4.04477 |
| MDK | 4.71195 | 8.37397 | 6.61766 | 3.60194 | 3.56102 | 3.8547 | 7.42733 |
| MDM2 | 6.07907 | 5.36103 | 5.3266 | 9.25007 | 6.05676 | 3.39879 | 7.30899 |
| METTL7B | 2.81115 | 4.88561 | 6.9479 | 3.62036 | 6.51662 | 3.00704 | 3.53268 |
| MFAP2 | 3.43654 | 7.16242 | 3.16045 | 3.39871 | 3.8983 | 4.00466 | 3.75652 |
| MGC39393* | 4.88395 | 8.75002 | 7.57392 | 9.77534 | 5.99431 | 10.1391 | 7.33806 |
| MGP | 8.67806 | 8.57764 | 4.43321 | 9.21279 | 12.548 | 3.2934 | 3.74561 |
| MGST1 | 3.60595 | 8.72705 | 5.13395 | 3.77594 | 9.90875 | 10.3524 | 9.0273 |
| MIA | 11.4013 | 10.6137 | 11.1506 | 10.6153 | 10.3601 | 9.77909 | 10.3045 |
| MICAL2 | 5.5694 | 4.21005 | 6.41222 | 4.70534 | 5.24766 | 6.00212 | 6.63261 |
| MID2 | 6.45433 | 3.26673 | 4.07283 | 5.84694 | 4.15398 | 2.80038 | 2.7622 |
| MKI67 | 6.9312 | 6.27362 | 7.82229 | 5.08038 | 7.33727 | 3.90417 | 7.64098 |
| MLANA | 3.70688 | 3.47238 | 12.6716 | 12.8955 | 10.0074 | 12.6046 | 12.8904 |
| MME | 4.01392 | 3.97259 | 5.24768 | 3.98779 | 3.35627 | 5.53469 | 5.16197 |
| MMP1 | 5.45021 | 2.98383 | 4.39414 | 3.34339 | 3.78173 | 2.86635 | 3.56097 |
| MMP8 | 4.45622 | 3.67519 | 6.82989 | 6.25777 | 4.70007 | 6.13211 | 7.28081 |
| MPPED2 | 3.48627 | 4.35125 | 3.85379 | 8.50787 | 3.26162 | 5.41691 | 7.18115 |
| MRAS | 7.40352 | 5.71373 | 8.64601 | 7.17161 | 7.31122 | 7.63705 | 6.80651 |
| MRC2 | 10.7188 | 9.5538 | 5.19572 | 3.07822 | 7.7378 | 3.3151 | 3.06991 |
| MRPS6 | 8.94266 | 8.15075 | 9.48767 | 9.36484 | 9.13956 | 7.22565 | 7.12738 |
| MTUS1 | 8.80331 | 6.36622 | 9.28599 | 7.78775 | 10.5911 | 7.23584 | 8.57044 |
| MUC15 | 10.5476 | 9.09984 | 3.62133 | 3.28409 | 3.42572 | 3.44088 | 3.58213 |
| MX2 | 9.00455 | 8.0197 | 9.0461 | 7.58421 | 3.09611 | 5.78191 | 8.12698 |
| MYOF | 8.62684 | 8.56075 | 8.29208 | 9.52153 | 9.23722 | 4.99349 | 3.97 |
| MYOZ2 | 3.39774 | 2.97269 | 4.09331 | 2.79599 | 3.75744 | 3.34779 | 2.7494 |
| NANOS1 | 8.036 | 7.52484 | 7.11823 | 9.24901 | 5.85512 | 4.30099 | 7.0294 |
| NCAM1 | 8.09554 | 5.26595 | 8.38152 | 8.89976 | 6.43831 | 7.78123 | 4.25626 |
| NDC80 | 6.04452 | 6.2289 | 6.29229 | 5.81014 | 7.35191 | 5.54209 | 7.63252 |
| NEK2 | 3.28686 | 4.38196 | 4.10779 | 3.55532 | 4.62098 | 4.51801 | 4.50359 |
| NEK9 | 8.10931 | 7.44104 | 7.7487 | 6.71659 | 6.80891 | 8.42129 | 7.20425 |
| NFATC2 | 11.7441 | 11.4228 | 10.6774 | 11.5375 | 10.2302 | 5.57877 | 8.05797 |
| NFIA | 5.71678 | 5.80279 | 3.28415 | 6.159 | 6.70544 | 5.60331 | 5.24421 |
| NHLRC3 | 7.24726 | 6.1053 | 7.63201 | 7.57597 | 6.2115 | 7.44447 | 7.31405 |

| | | | | | | | |
|---|---|---|---|---|---|---|---|
| NINL | 4.81912 | 3.56824 | 4.90448 | 3.6316 | 3.64272 | 3.65841 | 3.7912 |
| NLGN4X | 7.69822 | 8.75199 | 4.13966 | 2.81789 | 7.83711 | 3.07399 | 2.9427 |
| NoGeneName | 5.99404 | 4.56056 | 7.28701 | 7.38112 | 3.46615 | 9.08758 | 8.67646 |
| NRCAM | 6.63405 | 7.46366 | 8.00624 | 7.38313 | 8.5783 | 5.49926 | 3.41294 |
| NRIP3 | 3.55767 | 3.91252 | 5.97213 | 3.68637 | 3.9405 | 4.83494 | 9.34537 |
| NRXN3 | 9.42255 | 3.64352 | 5.07439 | 4.9252 | 6.98912 | 3.45685 | 5.47364 |
| NSUN5 | 8.57748 | 8.17707 | 7.76728 | 8.39702 | 8.97162 | 8.69747 | 9.50311 |
| NUF2 | 5.50753 | 6.44201 | 5.77274 | 4.66519 | 6.80092 | 5.68743 | 6.77856 |
| NUPR1 | 3.89648 | 3.77781 | 11.2132 | 10.8418 | 8.69204 | 11.5051 | 4.31328 |
| OAS1 | 5.98261 | 4.87297 | 6.6852 | 8.43147 | 5.09453 | 4.22442 | 5.34069 |
| OLFML2B | 3.6423 | 2.88866 | 3.32521 | 3.77116 | 3.97062 | 3.77145 | 3.18331 |
| OLIG2 | 8.84692 | 6.62871 | 6.69056 | 4.77197 | 3.37541 | 6.91406 | 7.05628 |
| OVOS* \|\| LOC7 | 13.0889 | 12.7386 | 12.7739 | 12.2428 | 11.5465 | 12.8309 | 12.2562 |
| PABPC4L | 3.10833 | 5.43333 | 3.10633 | 3.01838 | 3.02928 | 3.39302 | 2.83996 |
| PAPPA | 8.81798 | 6.4656 | 3.74601 | 3.80013 | 4.0977 | 3.54123 | 4.0407 |
| PCDH1 | 5.37883 | 4.96431 | 6.59046 | 7.56472 | 4.56412 | 4.09171 | 3.32085 |
| PCDHB16 | 8.55098 | 7.73684 | 6.66768 | 7.74066 | 8.05719 | 3.57147 | 3.25869 |
| PCDHB5 | 7.73552 | 6.76681 | 5.56708 | 7.94909 | 8.00974 | 4.32413 | 2.92648 |
| PCDHB6 | 5.96131 | 6.6447 | 4.30083 | 6.16625 | 8.16693 | 2.89705 | 2.78722 |
| PCSK5 | 8.51001 | 4.08633 | 6.51736 | 6.96153 | 7.97573 | 3.38342 | 2.9403 |
| PDE4DIP | 5.13548 | 6.54925 | 4.82241 | 5.7535 | 7.05673 | 5.79635 | 5.32733 |
| PHGDH | 8.72278 | 9.76078 | 10.2414 | 10.8015 | 9.22722 | 11.1701 | 10.2458 |
| PHIP | 9.00278 | 8.662 | 8.66624 | 7.86399 | 8.4601 | 5.34578 | 9.09151 |
| PHKA1 | 3.60027 | 5.3422 | 2.72047 | 6.29851 | 5.42538 | 4.45865 | 4.04209 |
| PHLDB2 | 6.19545 | 8.49878 | 5.65393 | 7.05116 | 8.19373 | 9.42289 | 4.01865 |
| PI15 | 7.51654 | 7.80768 | 9.80594 | 9.99503 | 9.86658 | 8.3006 | 7.56023 |
| PIR | 9.67709 | 7.73897 | 10.7761 | 10.897 | 8.89025 | 11.5156 | 9.97813 |
| PLA1A | 3.73529 | 3.36389 | 9.17261 | 8.40274 | 7.20879 | 10.2655 | 8.51766 |
| PLAGL1 | 3.00407 | 3.07201 | 3.11592 | 2.68893 | 5.99933 | 3.35127 | 3.56231 |
| PLAT | 7.82801 | 7.44011 | 9.97978 | 10.9381 | 9.92143 | 6.3482 | 8.01922 |
| PLCB4 | 7.57357 | 4.24707 | 3.36822 | 5.63384 | 5.73075 | 3.02317 | 7.56901 |
| PLEKHA5 | 5.73973 | 7.40245 | 7.41781 | 7.32932 | 7.81824 | 9.33468 | 8.79838 |
| PLEKHB1 | 3.7798 | 3.6957 | 4.42808 | 5.64053 | 5.14175 | 3.81713 | 3.39126 |
| PLK4 | 3.79729 | 4.48117 | 5.6452 | 3.99519 | 4.90934 | 5.24628 | 6.20594 |
| PLN | 2.99821 | 2.59822 | 2.62949 | 2.76497 | 2.81656 | 2.79798 | 2.76478 |
| PLOD2 | 6.99156 | 7.02864 | 5.63009 | 5.83649 | 7.84871 | 4.91437 | 6.70979 |
| PLP1 | 11.9477 | 11.3283 | 11.5509 | 12.1011 | 11.2255 | 11.315 | 9.96741 |
| PLXNB2 | 8.62418 | 7.15831 | 8.59478 | 7.60704 | 9.0162 | 6.23754 | 8.64152 |
| PNMAL1 | 2.99465 | 9.87246 | 3.04415 | 2.98959 | 3.16255 | 4.82943 | 6.47171 |
| PNPLA4 | 7.91648 | 2.59009 | 8.43421 | 9.2237 | 9.08831 | 8.60417 | 8.7929 |
| POSTN | 3.17992 | 2.97325 | 2.8815 | 2.87432 | 10.2184 | 3.10069 | 2.73978 |
| PPIG | 7.30027 | 5.69069 | 7.08216 | 5.58404 | 8.29645 | 2.76698 | 7.66225 |
| PPP1R14C | 5.26237 | 4.5861 | 7.58325 | 6.19137 | 5.44266 | 3.4489 | 7.83372 |
| PPP1R3C | 3.5713 | 3.33869 | 3.45972 | 8.81185 | 3.77092 | 3.54407 | 7.64989 |
| PRDM7 | 2.78694 | 3.5026 | 4.84412 | 6.77181 | 3.03498 | 9.59355 | 8.98795 |
| PRDM8 | 3.34109 | 4.32258 | 3.7684 | 4.01285 | 4.03631 | 3.76047 | 3.65251 |
| PREX1 | 7.28097 | 5.89576 | 6.23303 | 7.72536 | 5.91143 | 5.39993 | 5.30699 |
| PRKCD | 9.28197 | 8.92673 | 7.47084 | 7.33186 | 8.56168 | 7.58337 | 7.52603 |
| PRR4 | 6.66776 | 4.2916 | 4.82448 | 4.05753 | 6.2961 | 3.47078 | 4.1286 |
| PRSS23 | 10.5405 | 9.11769 | 9.46471 | 10.7615 | 10.4561 | 4.85736 | 4.93702 |
| PRSS33 | 3.42481 | 8.14379 | 4.09505 | 8.31902 | 3.36373 | 4.8377 | 4.49235 |
| PTEN | 8.37716 | 8.26202 | 7.80703 | 8.38926 | 7.29954 | 6.76116 | 7.66194 |
| PTGES | 4.94048 | 7.06696 | 3.67946 | 4.22666 | 4.10544 | 5.182 | 3.33739 |

| | | | | | | | |
|---|---|---|---|---|---|---|---|
| PTGS2 | 8.07346 | 3.16856 | 3.0259 | 3.38239 | 6.68828 | 2.84759 | 2.80994 |
| PTN | 10.1545 | 7.96615 | 3.33507 | 3.30115 | 8.37814 | 3.08837 | 3.18877 |
| PTPRN2 | 8.02637 | 2.92676 | 3.34027 | 3.19389 | 3.6129 | 3.44518 | 3.40161 |
| PTPRZ1 | 10.7566 | 7.43443 | 8.44611 | 7.91076 | 9.1842 | 4.8643 | 5.30916 |
| PYCARD | 3.78477 | 3.09934 | 10.2832 | 10.8948 | 3.01229 | 11.8052 | 9.23083 |
| PYGB | 9.70351 | 10.1856 | 9.44576 | 7.82575 | 8.49704 | 7.17704 | 8.76331 |
| PYROXD2 | 7.36027 | 7.77736 | 4.81706 | 5.78122 | 4.43479 | 3.73289 | 3.80036 |
| RAB17 | 7.86717 | 8.29327 | 7.66118 | 7.91406 | 6.53989 | 9.47749 | 9.2903 |
| RAB31 | 8.45811 | 6.18513 | 7.06867 | 3.99952 | 5.6871 | 4.65482 | 7.08095 |
| RAB7B | 3.85829 | 6.53615 | 5.13955 | 6.92896 | 6.49387 | 4.92979 | 3.08859 |
| RAD51AP1 | 5.03575 | 6.1296 | 6.24648 | 5.46181 | 6.52943 | 6.13922 | 6.97952 |
| RARB | 4.99008 | 3.15487 | 3.05348 | 3.02895 | 4.39734 | 3.45082 | 4.20948 |
| RASSF2 | 3.39388 | 3.21879 | 4.7638 | 3.35948 | 3.00222 | 3.61797 | 7.92987 |
| RASSF8 | 6.92198 | 7.78812 | 6.68092 | 6.64123 | 6.94802 | 5.38866 | 6.54571 |
| RBM25 | 7.17169 | 6.78695 | 7.18927 | 7.29453 | 7.69882 | 3.30151 | 8.09165 |
| RCS* | 8.14705 | 7.7976 | 8.23179 | 3.06233 | 8.10802 | 3.1634 | 3.18517 |
| RFTN1 | 8.72962 | 7.57841 | 5.68485 | 7.28727 | 4.95718 | 4.44455 | 3.99772 |
| RGS1 | 2.73728 | 2.721 | 5.19808 | 2.91126 | 2.84061 | 7.38276 | 3.16952 |
| RGS2 | 7.00986 | 7.09701 | 6.35815 | 6.53835 | 8.38188 | 7.46266 | 4.35942 |
| RHBDF1 | 8.73402 | 8.37556 | 8.51043 | 7.33188 | 7.51027 | 6.98055 | 7.97613 |
| RIPK4 | 8.2681 | 9.61056 | 8.67332 | 9.95026 | 7.81207 | 10.2104 | 10.0012 |
| RNF128 | 8.44807 | 7.13146 | 6.79831 | 4.39405 | 9.60096 | 2.96236 | 5.45349 |
| RNF175 | 6.50736 | 5.41195 | 2.86685 | 3.32924 | 7.19415 | 2.86025 | 4.69433 |
| RNLS | 3.47013 | 3.29634 | 3.66535 | 3.52904 | 3.3878 | 9.4236 | 3.86172 |
| ROPN1B \|\| ROP | 8.6041 | 5.61142 | 9.34882 | 9.37546 | 8.11725 | 9.05432 | 9.58426 |
| RPS23 | 9.40785 | 8.83902 | 8.55672 | 9.40026 | 5.88453 | 7.39691 | 4.6826 |
| RPS4Y1 | 3.51822 | 3.50057 | 3.30393 | 3.43651 | 3.58238 | 12.0159 | 4.01156 |
| RRAGD | 8.79132 | 8.68073 | 10.3749 | 9.36583 | 8.92978 | 10.7003 | 10.8901 |
| RUNX3 | 3.11176 | 5.73973 | 7.8855 | 7.22414 | 2.95102 | 8.29749 | 8.5835 |
| RYR2 | 6.72143 | 6.28848 | 2.92844 | 2.79043 | 6.0457 | 3.08216 | 3.05512 |
| S100B | 11.6766 | 8.91269 | 11.3912 | 11.9794 | 11.6532 | 9.10189 | 10.4076 |
| SAA1 | 6.74204 | 2.96782 | 3.3493 | 3.57448 | 3.15726 | 3.24633 | 3.25316 |
| SAA1 \|\| SAA2 | 8.83776 | 3.30423 | 3.07638 | 3.4141 | 3.00055 | 2.82436 | 2.80767 |
| SAT1 | 9.34184 | 8.54695 | 9.32443 | 10.3699 | 9.60369 | 10.4236 | 10.1353 |
| SATB1 | 8.70044 | 7.24734 | 7.57998 | 6.17973 | 6.62224 | 7.87902 | 6.13255 |
| SCG5 | 5.20993 | 3.00863 | 3.18498 | 2.98913 | 3.16954 | 3.25099 | 2.99346 |
| SCIN | 2.90819 | 3.23648 | 4.3138 | 5.76711 | 2.8158 | 2.95991 | 2.89758 |
| SCRG1 | 10.477 | 3.47162 | 3.93297 | 3.78234 | 9.31207 | 3.10896 | 3.20443 |
| SDC1 | 4.49453 | 5.21882 | 5.96572 | 4.14375 | 6.95442 | 3.96861 | 7.5266 |
| SDC2 | 8.69748 | 7.94011 | 6.30065 | 7.45543 | 6.81196 | 3.08613 | 4.94871 |
| SEMA3B | 9.82434 | 8.83848 | 9.69112 | 4.75722 | 5.37998 | 4.54038 | 4.40638 |
| SERPINA3 | 12.8469 | 12.4301 | 5.93206 | 7.19517 | 13.0129 | 3.71325 | 3.75275 |
| SERPINB2 | 7.86106 | 2.77324 | 2.92954 | 2.81955 | 3.08269 | 3.0133 | 3.38048 |
| SERPINE1 | 6.07035 | 3.08293 | 3.21333 | 2.84497 | 8.89472 | 3.28404 | 3.23934 |
| SERPINE2 | 10.533 | 9.25231 | 10.0445 | 9.69067 | 11.296 | 7.0182 | 7.58675 |
| SFRP1 | 12.3795 | 11.5406 | 10.5436 | 10.3856 | 9.53509 | 5.95302 | 8.50049 |
| SGCD | 6.66036 | 3.45914 | 6.74022 | 8.25613 | 6.10616 | 7.79199 | 4.70818 |
| SGOL2 | 4.52065 | 4.76421 | 5.26914 | 4.38385 | 5.92374 | 3.93745 | 6.56261 |
| SHANK3 | 7.84856 | 5.4494 | 7.37068 | 7.81537 | 6.9361 | 7.66594 | 6.78519 |
| SHROOM4 | 6.61845 | 6.13696 | 5.57558 | 6.41874 | 7.2533 | 4.89588 | 3.80853 |
| SLAIN1 | 4.39919 | 4.64855 | 3.43028 | 3.39323 | 5.26954 | 7.67946 | 6.28109 |
| SLAMF7 | 3.3195 | 3.41835 | 3.53104 | 4.32139 | 3.69517 | 3.90939 | 8.20478 |
| SLC14A1 | 3.33228 | 3.82546 | 3.00102 | 3.29915 | 2.93529 | 2.90518 | 2.79936 |

| | | | | | | | |
|---|---|---|---|---|---|---|---|
| SLC16A3 | 6.3441 | 3.03501 | 4.87697 | 2.9003 | 3.34989 | 3.55762 | 2.73361 |
| SLC16A4 | 9.10598 | 8.00772 | 6.87995 | 8.68794 | 8.03338 | 7.42399 | 6.26465 |
| SLC16A6 | 3.24852 | 3.28381 | 10.2645 | 7.19276 | 2.93489 | 9.21474 | 8.78566 |
| SLC1A1 | 9.76581 | 9.42596 | 5.4114 | 7.06323 | 7.07114 | 6.80127 | 3.80169 |
| SLC1A4 | 9.45898 | 8.42385 | 10.0018 | 10.2797 | 9.31221 | 11.1459 | 7.28809 |
| SLC26A2 | 11.8735 | 11.1046 | 7.18588 | 8.66264 | 10.3552 | 8.07588 | 8.20317 |
| SLC45A2 | 3.30176 | 2.9155 | 8.91743 | 10.7356 | 9.10817 | 10.0639 | 10.6386 |
| SLC5A3 | 10.7581 | 10.4542 | 10.6185 | 10.3707 | 10.9285 | 7.79555 | 7.53415 |
| SLCO3A1 | 5.57964 | 4.26666 | 6.62673 | 7.98666 | 3.56122 | 6.05469 | 3.95096 |
| SMC2 | 5.18313 | 7.10525 | 5.90719 | 6.23295 | 7.85898 | 5.31335 | 6.99179 |
| SMC3 | 8.04335 | 8.76694 | 7.4263 | 6.89771 | 7.65008 | 4.17293 | 7.93123 |
| SMC4 | 8.05218 | 8.98633 | 9.1432 | 7.22836 | 9.28731 | 6.29662 | 9.44806 |
| SNAPC1 | 7.2684 | 7.40571 | 5.28495 | 5.22817 | 8.29003 | 4.91139 | 7.8475 |
| SNX10 | 10.9207 | 10.2307 | 10.7473 | 10.6431 | 10.2703 | 9.27946 | 10.837 |
| SOAT1 | 4.92471 | 6.00848 | 5.93475 | 6.25983 | 5.59867 | 4.74742 | 6.94015 |
| SORBS2 | 9.86583 | 7.59063 | 9.117 | 6.71628 | 8.38775 | 3.93381 | 3.46459 |
| SORCS1 | 7.86404 | 7.22177 | 7.27966 | 7.45043 | 7.14509 | 5.56087 | 4.90962 |
| SOX10 | 8.24883 | 8.17349 | 8.02801 | 7.63945 | 7.49305 | 7.15875 | 7.96047 |
| SOX6 | 9.0815 | 3.99718 | 7.5822 | 9.20654 | 8.51943 | 8.39296 | 8.71966 |
| SPANXB2 \|\| SPA | 3.20109 | 3.0522 | 3.41231 | 3.25959 | 3.39645 | 3.01516 | 3.12861 |
| SPANXE \|\| SPAN | 3.03829 | 2.91219 | 2.99796 | 3.03465 | 2.97408 | 2.99501 | 3.00979 |
| SPATS1 | 2.73723 | 2.86783 | 2.94833 | 7.78595 | 9.09687 | 6.42091 | 6.61008 |
| SPESP1 | 2.54278 | 7.76702 | 2.66733 | 2.50469 | 2.57052 | 8.10025 | 7.63415 |
| SPP1 | 10.948 | 4.80115 | 8.88176 | 7.95422 | 3.92259 | 5.65751 | 10.0724 |
| SPRY4 | 10.384 | 10.3953 | 7.80282 | 7.81836 | 9.63552 | 6.67879 | 6.19945 |
| SPRYD5 | 9.43561 | 8.71118 | 8.98086 | 8.75656 | 9.28499 | 6.16188 | 7.64911 |
| SRGN | 6.96045 | 6.36367 | 6.52026 | 5.4535 | 6.8937 | 5.98105 | 7.57991 |
| STAT5A | 6.39061 | 6.80712 | 5.69754 | 6.45076 | 6.21261 | 7.58856 | 6.54192 |
| STC1 | 5.38697 | 3.89054 | 3.05086 | 3.27682 | 9.49114 | 3.63656 | 3.09626 |
| STC2 | 4.01563 | 3.56293 | 2.79457 | 3.54224 | 3.10958 | 3.14986 | 2.9779 |
| STK32B | 9.64615 | 9.53981 | 6.63018 | 7.61868 | 9.00239 | 6.54706 | 8.64449 |
| SYCP2 | 3.52958 | 3.28805 | 3.64124 | 4.46805 | 4.50697 | 2.92511 | 3.11499 |
| TBXAS1 | 6.87864 | 5.42788 | 3.09426 | 3.09502 | 6.24258 | 2.92589 | 3.96822 |
| TCN1 | 3.30198 | 3.50441 | 6.05916 | 8.60765 | 8.77841 | 9.56738 | 7.45653 |
| TESK2 | 4.22233 | 5.29247 | 7.85569 | 7.30756 | 5.13285 | 8.07496 | 8.11106 |
| TF | 9.61957 | 6.17651 | 7.0074 | 9.93207 | 7.31954 | 7.36865 | 3.30196 |
| TFAP2B | 9.00576 | 12.3265 | 3.03391 | 3.22599 | 3.65116 | 2.82948 | 2.9901 |
| TFAP2C | 10.7354 | 10.7058 | 10.099 | 9.3613 | 9.64171 | 8.33676 | 6.83543 |
| TFPI | 9.04304 | 5.18851 | 4.87151 | 3.91958 | 8.87173 | 3.93864 | 3.43542 |
| TFPI2 | 7.57775 | 5.53383 | 2.90237 | 3.01723 | 2.71765 | 3.12774 | 3.76353 |
| TGFBI | 11.9385 | 9.65568 | 11.5491 | 3.20578 | 6.98249 | 3.646 | 3.47897 |
| THBS2 | 10.4488 | 5.49353 | 3.10167 | 2.72722 | 3.46119 | 2.90697 | 9.64788 |
| TIMP3 | 12.1795 | 11.4575 | 11.6836 | 11.0263 | 12.0709 | 10.2655 | 8.66161 |
| TM4SF1 | 10.3631 | 9.32608 | 9.02575 | 8.34737 | 10.86 | 4.38462 | 9.09523 |
| TMEM140 | 8.29308 | 3.96168 | 7.83035 | 9.14969 | 5.44736 | 8.22247 | 6.28752 |
| TMEM195 | 11.8345 | 10.2426 | 3.45352 | 3.53771 | 11.2076 | 3.9674 | 2.82937 |
| TMEM200A | 3.18485 | 2.86952 | 2.87604 | 3.3311 | 2.87371 | 2.95428 | 3.07058 |
| TMEM200B | 3.28987 | 4.27993 | 3.77029 | 3.57894 | 3.00423 | 4.347 | 4.91187 |
| TMEM229B | 5.80042 | 6.62885 | 6.2039 | 4.82176 | 6.08251 | 5.62486 | 7.01355 |
| TMEM47 | 8.47504 | 7.79139 | 5.03499 | 7.50634 | 10.0544 | 3.19196 | 3.01495 |
| TMEM98 | 9.67562 | 8.76282 | 7.43996 | 9.38567 | 5.83929 | 9.03218 | 9.73286 |
| TMSB4X | 10.8866 | 7.31889 | 3.26421 | 6.74464 | 5.75966 | 4.78693 | 11.9491 |
| TNFRSF19 | 8.06865 | 8.38054 | 8.66344 | 6.63226 | 8.92657 | 9.86906 | 5.83373 |

| | | | | | | | |
|---|---|---|---|---|---|---|---|
| TNFRSF6B | 3.59765 | 3.53005 | 3.17698 | 3.68181 | 3.29614 | 3.57953 | 3.09481 |
| TOP2A | 9.90778 | 10.4353 | 10.0653 | 9.70927 | 11.1619 | 10.1685 | 10.6287 |
| TPD52 | 7.21857 | 8.52478 | 8.71685 | 9.24658 | 8.28971 | 8.65396 | 9.72833 |
| TPD52L1 | 6.32897 | 10.9676 | 6.70806 | 8.73409 | 9.43058 | 4.78336 | 11.0815 |
| TPM2 | 5.99511 | 10.6234 | 7.00318 | 5.75367 | 6.47348 | 6.47553 | 4.34888 |
| TPR | 8.5546 | 9.07686 | 7.17823 | 8.07256 | 8.93429 | 4.09074 | 8.13194 |
| TRIM2 | 10.165 | 8.87019 | 10.1932 | 9.44206 | 8.28538 | 7.96247 | 9.18192 |
| TRIM48 | 9.55803 | 3.19167 | 9.43286 | 2.91639 | 4.23944 | 2.94573 | 3.48758 |
| TRIM9 | 7.51513 | 7.23266 | 6.88424 | 3.4158 | 8.87422 | 4.13789 | 3.95278 |
| TRIP11 | 4.9828 | 5.66095 | 5.23254 | 3.91138 | 6.66382 | 3.43044 | 6.01556 |
| TRPM8 | 9.24846 | 5.58519 | 6.94213 | 5.91249 | 7.88878 | 5.5659 | 4.21028 |
| TSPAN13 | 11.5654 | 10.9343 | 7.11719 | 6.88463 | 9.94955 | 7.21399 | 6.94433 |
| TSPAN33 | 7.08073 | 6.34212 | 6.07358 | 7.23186 | 7.05188 | 7.34984 | 6.00941 |
| TSPAN5 | 5.15738 | 4.4394 | 3.45842 | 4.36516 | 5.52995 | 3.32674 | 3.66108 |
| TUSC1 | 5.66969 | 6.70072 | 5.39328 | 2.65963 | 6.26489 | 6.43348 | 6.00627 |
| TYR | 6.51961 | 2.83159 | 12.6433 | 11.4131 | 11.425 | 12.5496 | 11.644 |
| UACA | 6.65259 | 7.38396 | 7.42453 | 6.7488 | 8.7261 | 5.75347 | 7.65989 |
| UBD | 11.2544 | 6.69599 | 3.15271 | 3.28622 | 3.18956 | 3.06102 | 3.30088 |
| UCN2 | 4.26018 | 5.97327 | 9.46258 | 5.25155 | 7.45957 | 3.38445 | 8.94635 |
| UGT8 | 9.65586 | 8.02384 | 6.74626 | 4.09234 | 6.98597 | 4.07847 | 4.14335 |
| UHRF1 | 4.63882 | 5.2616 | 6.47426 | 4.88763 | 5.72861 | 4.97915 | 5.38824 |
| VAMP8 | 4.91642 | 7.30494 | 7.23519 | 3.83114 | 6.57959 | 7.56733 | 3.20429 |
| VAV3 | 3.62181 | 3.53172 | 4.40998 | 7.54556 | 6.76214 | 8.02478 | 9.60056 |
| VEGFC | 6.19478 | 4.99374 | 3.77203 | 4.53193 | 7.26846 | 3.6771 | 3.57813 |
| VGF | 8.73209 | 10.2627 | 7.78202 | 8.35795 | 10.283 | 8.69637 | 11.0355 |
| VWA5A | 3.32487 | 7.23699 | 6.02757 | 5.96955 | 5.09581 | 3.87254 | 3.33173 |
| WDR66 | 3.60282 | 3.11011 | 2.87243 | 3.22882 | 3.4559 | 3.90939 | 3.08143 |
| WIPI1 | 9.52241 | 8.94689 | 10.6708 | 9.78243 | 9.1959 | 11.1801 | 10.485 |
| WNK4 | 6.44305 | 7.07078 | 3.79175 | 3.17424 | 5.28795 | 3.3191 | 3.07521 |
| XAGE1C \|\| XAG | 2.94943 | 11.4359 | 3.13768 | 3.02508 | 7.82349 | 3.00083 | 3.16352 |
| XIST | 3.34043 | 11.3416 | 10.0641 | 11.3333 | 10.4392 | 3.19771 | 9.56088 |
| XYLT1 | 6.99626 | 6.84974 | 6.40267 | 7.24612 | 8.69019 | 5.03954 | 3.3732 |
| ZC3HAV1L | 3.21883 | 4.62193 | 6.67243 | 3.50161 | 5.22409 | 5.36137 | 3.18719 |
| ZNF83 | 9.17347 | 8.92621 | 8.18214 | 7.17876 | 8.80447 | 8.00956 | 7.54847 |
| ZNF91 | 5.72607 | 8.42637 | 3.56649 | 6.96933 | 6.72509 | 5.14346 | 6.81433 |

ıd a resistant group (separated by a double line). Genes with expression value difference over 1 between two groups are reported.

| C32 | RVH-421 | A101D | UACC-257 | M14 | HT-144 | MZ7-mel | IST-MEL1 |
|---|---|---|---|---|---|---|---|
| 7.83281 | 6.38739 | 6.32771 | 6.42397 | 5.78613 | 6.94834 | 4.04148 | 6.77163 |
| 2.87807 | 2.8661 | 2.8839 | 2.86088 | 2.70124 | 2.84658 | 2.88803 | 6.46068 |
| 6.09589 | 3.36277 | 5.48856 | 3.20367 | 3.42781 | 3.29622 | 3.27338 | 4.66227 |
| 7.90053 | 6.94308 | 9.41644 | 7.7796 | 6.53038 | 6.71002 | 6.23176 | 8.19104 |
| 6.98608 | 6.88871 | 8.2453 | 6.52179 | 6.60869 | 7.00681 | 6.00466 | 9.18593 |
| 9.05906 | 9.03931 | 8.38849 | 9.32362 | 9.20336 | 9.22085 | 7.43435 | 8.33812 |
| 8.95778 | 8.53903 | 9.16344 | 8.83477 | 3.53347 | 8.78577 | 6.42896 | 4.48872 |
| 3.34361 | 3.30741 | 3.5917 | 3.52499 | 3.26338 | 3.07211 | 3.94067 | 4.22965 |
| 2.6916 | 6.61183 | 3.27603 | 3.05503 | 3.26686 | 3.64613 | 2.76664 | 3.10351 |
| 2.88397 | 2.86938 | 2.97925 | 2.7272 | 2.73487 | 3.07596 | 2.88884 | 3.03092 |
| 5.98622 | 6.68556 | 7.38739 | 5.22532 | 4.61812 | 2.8189 | 3.92864 | 7.93148 |
| 7.27382 | 8.07494 | 3.67385 | 7.84019 | 4.41951 | 3.89921 | 5.45379 | 3.88967 |
| 9.78526 | 6.98564 | 9.25132 | 5.32718 | 7.58948 | 5.59075 | 4.70164 | 9.66879 |
| 11.5272 | 8.08493 | 8.92152 | 7.84726 | 6.94044 | 7.38717 | 6.29508 | 6.50382 |
| 3.61585 | 3.38379 | 3.24001 | 2.80779 | 5.8656 | 3.40591 | 2.89573 | 4.28746 |
| 3.71334 | 4.92088 | 5.0739 | 3.38401 | 3.32799 | 5.16127 | 3.19366 | 6.60628 |
| 7.42316 | 9.83216 | 8.05666 | 4.6337 | 8.58584 | 9.33519 | 9.25111 | 8.20166 |
| 10.1008 | 9.69897 | 5.7246 | 7.7796 | 5.29339 | 7.97907 | 3.85041 | 3.38359 |
| 4.07677 | 8.70062 | 8.44744 | 10.9005 | 3.16568 | 3.54427 | 4.70418 | 7.05626 |
| 4.1757 | 6.27484 | 3.29788 | 5.03112 | 3.19929 | 4.62314 | 4.15826 | 2.98037 |
| 10.0578 | 4.50961 | 8.55553 | 7.73002 | 4.14885 | 6.51239 | 4.93746 | 7.13662 |
| 3.86096 | 3.8096 | 5.4401 | 4.31619 | 5.10907 | 3.88542 | 3.6921 | 9.36067 |
| 4.78901 | 4.08419 | 7.40753 | 3.1222 | 5.7094 | 7.05579 | 3.74302 | 8.7252 |
| 3.18223 | 6.47831 | 6.33317 | 7.31177 | 3.30697 | 3.22194 | 3.29529 | 6.56417 |
| 3.59009 | 3.51466 | 9.81295 | 3.99783 | 3.38663 | 3.51329 | 3.37181 | 3.30206 |
| 6.54162 | 6.9359 | 6.47635 | 3.751 | 7.5161 | 6.77184 | 7.72427 | 6.30559 |
| 7.26322 | 3.44183 | 8.64288 | 5.1471 | 9.17757 | 5.73681 | 8.33359 | 9.92276 |
| 3.18127 | 4.01445 | 5.08721 | 4.10919 | 6.92639 | 7.4376 | 6.45222 | 4.30498 |
| 5.50364 | 3.72304 | 6.24251 | 5.35911 | 7.29064 | 5.4108 | 7.80163 | 7.89448 |
| 3.623 | 3.45429 | 3.20149 | 3.3103 | 3.02458 | 3.97978 | 3.42667 | 5.2433 |
| 8.14553 | 3.62096 | 3.30813 | 12.2085 | 3.35966 | 3.35364 | 8.65677 | 3.23262 |
| 6.63405 | 9.76678 | 8.11518 | 9.1469 | 5.73337 | 5.38094 | 4.3187 | 5.95697 |
| 8.0574 | 3.68148 | 6.08163 | 3.04707 | 3.16361 | 6.11012 | 3.07148 | 2.7975 |
| 9.69465 | 8.12825 | 9.01804 | 9.92915 | 8.96819 | 9.05203 | 7.30758 | 7.39624 |
| 7.54692 | 3.60954 | 7.97321 | 4.45483 | 8.75946 | 6.29467 | 9.08204 | 9.30972 |
| 6.15695 | 7.65432 | 7.30603 | 3.61783 | 7.71124 | 7.06413 | 6.78294 | 7.98778 |
| 6.24995 | 8.27536 | 8.10555 | 3.3978 | 7.92063 | 7.86413 | 7.94745 | 7.84561 |
| 5.64798 | 4.25064 | 7.69131 | 4.83353 | 3.18526 | 4.81666 | 3.0548 | 3.71746 |
| 4.31903 | 4.01066 | 4.55475 | 5.10666 | 5.88146 | 5.32932 | 5.81132 | 5.19344 |
| 3.45823 | 5.38509 | 4.6994 | 6.43052 | 7.40093 | 3.30495 | 7.99485 | 5.2881 |
| 9.43372 | 6.87444 | 8.02022 | 3.23196 | 3.2058 | 6.53326 | 3.90972 | 3.41811 |
| 3.72347 | 3.04307 | 3.16376 | 3.7392 | 3.53658 | 3.32741 | 3.16467 | 3.43565 |
| 6.13792 | 7.49796 | 7.51678 | 3.7938 | 7.15709 | 7.68895 | 7.778 | 8.31715 |
| 3.53341 | 4.61341 | 2.83673 | 8.08255 | 3.20185 | 3.03744 | 5.75988 | 2.95938 |
| 9.94612 | 10.1339 | 9.51766 | 9.90648 | 10.355 | 9.54518 | 9.8677 | 7.24744 |
| 3.02742 | 3.39248 | 2.95218 | 2.8222 | 2.8868 | 3.06423 | 2.74265 | 2.86513 |
| 2.99505 | 3.06357 | 6.29224 | 3.24113 | 2.6306 | 5.47481 | 3.13314 | 4.14101 |
| 7.33447 | 8.6518 | 4.77671 | 8.22579 | 5.06272 | 6.67013 | 6.5953 | 3.24311 |
| 3.39363 | 3.36779 | 6.99365 | 4.02553 | 3.33802 | 3.54427 | 3.49501 | 6.57049 |

| | | | | | | | |
|---|---|---|---|---|---|---|---|
| 7.37207 | 4.67665 | 5.96198 | 4.69267 | 5.39191 | 5.80707 | 4.02607 | 5.35943 |
| 6.39993 | 9.40377 | 6.93365 | 10.8091 | 4.4257 | 7.15015 | 8.42847 | 6.74615 |
| 7.11952 | 6.72531 | 10.7725 | 10.198 | 10.6288 | 7.82237 | 3.94982 | 7.15916 |
| 6.49094 | 7.30195 | 6.26843 | 6.15671 | 7.19642 | 7.13595 | 8.97306 | 6.6278 |
| 4.93269 | 4.1863 | 6.87556 | 3.08974 | 3.11173 | 3.26103 | 2.84525 | 3.01677 |
| 8.74063 | 5.60461 | 6.91133 | 6.51798 | 3.81015 | 6.4987 | 6.01855 | 4.19145 |
| 6.88972 | 3.16598 | 7.92952 | 7.63725 | 8.58297 | 5.88959 | 8.34454 | 9.26099 |
| 7.65574 | 6.96752 | 6.89622 | 6.1174 | 6.83777 | 6.77541 | 6.58079 | 3.42659 |
| 4.5675 | 3.04223 | 4.04838 | 3.01621 | 6.49862 | 4.50598 | 4.15289 | 6.63916 |
| 4.4718 | 4.0624 | 4.64175 | 3.55679 | 5.74997 | 4.76131 | 5.01086 | 5.70728 |
| 3.44171 | 3.25955 | 5.87547 | 3.87589 | 3.52232 | 5.92862 | 4.05436 | 6.49886 |
| 3.39026 | 5.49526 | 10.6538 | 9.8473 | 2.86222 | 8.21274 | 3.25024 | 4.21692 |
| 7.61087 | 9.42527 | 7.317 | 8.94085 | 6.28485 | 7.55662 | 7.04188 | 4.57338 |
| 6.82201 | 6.81861 | 8.15189 | 7.85437 | 3.03947 | 8.53443 | 6.61299 | 7.02244 |
| 3.00589 | 3.01102 | 7.98615 | 2.81988 | 2.92144 | 2.84686 | 2.88076 | 2.99634 |
| 7.21529 | 3.27502 | 4.53761 | 3.44283 | 7.35251 | 4.54341 | 6.31828 | 3.43008 |
| 4.85419 | 3.82796 | 4.94592 | 5.56499 | 6.40389 | 5.12917 | 6.00192 | 6.45281 |
| 9.97077 | 8.32489 | 9.49095 | 10.5015 | 8.63064 | 9.10122 | 10.0596 | 5.80738 |
| 4.10308 | 3.25768 | 3.4007 | 6.30099 | 7.2657 | 5.80879 | 3.81841 | 3.86523 |
| 5.72373 | 4.50626 | 6.91542 | 11.5621 | 9.45385 | 8.9477 | 5.88337 | 3.29188 |
| 9.79554 | 8.35643 | 8.81675 | 11.9657 | 8.16338 | 9.11895 | 10.3371 | 3.02564 |
| 6.43398 | 6.38181 | 6.57897 | 3.14537 | 7.52225 | 6.86132 | 7.81563 | 7.55616 |
| 4.07699 | 4.2925 | 5.98052 | 3.5086 | 4.83489 | 4.64902 | 6.67351 | 5.5826 |
| 9.44759 | 8.57471 | 8.78618 | 9.61887 | 8.81205 | 8.90124 | 9.13915 | 8.11855 |
| 6.73695 | 4.59614 | 6.41707 | 5.44033 | 4.51907 | 6.12928 | 4.47549 | 4.75379 |
| 10.8781 | 6.34247 | 8.52831 | 3.97066 | 5.75471 | 10.7611 | 3.15821 | 2.73446 |
| 10.6079 | 7.50688 | 9.64254 | 9.5744 | 10.0177 | 7.49948 | 7.72215 | 9.78973 |
| 2.56511 | 3.62044 | 3.01947 | 11.2301 | 3.16079 | 2.80478 | 4.21122 | 2.88886 |
| 3.12636 | 2.82421 | 8.57286 | 3.02865 | 3.96112 | 6.37602 | 3.10123 | 10.5258 |
| 6.80055 | 4.98642 | 6.78984 | 10.6734 | 4.62509 | 3.96878 | 8.09224 | 3.46236 |
| 5.91945 | 2.87111 | 7.32336 | 2.85311 | 8.04651 | 5.05343 | 7.68584 | 7.13173 |
| 6.09933 | 3.60592 | 7.28803 | 2.71894 | 9.5009 | 6.37376 | 7.54408 | 9.21398 |
| 3.50009 | 4.08256 | 3.97717 | 4.52853 | 5.93983 | 3.30112 | 7.401 | 6.86891 |
| 6.76863 | 4.84198 | 6.72573 | 3.54097 | 8.13931 | 6.79858 | 7.78194 | 8.11629 |
| 7.63184 | 3.93541 | 8.35124 | 5.88915 | 10.1779 | 6.61731 | 9.02244 | 9.99038 |
| 2.86638 | 3.04305 | 2.75583 | 2.73937 | 2.8067 | 3.17178 | 4.84277 | 2.80304 |
| 3.52874 | 3.44482 | 3.41445 | 3.54938 | 3.49746 | 4.20137 | 6.40623 | 3.51822 |
| 3.75493 | 4.09369 | 7.05821 | 3.52159 | 3.3716 | 4.51011 | 3.36811 | 6.65341 |
| 5.63725 | 4.16666 | 7.23399 | 4.13904 | 7.76066 | 5.05749 | 7.05197 | 8.76131 |
| 8.34804 | 4.74306 | 4.58502 | 6.47083 | 5.94402 | 6.74311 | 7.75174 | 6.81039 |
| 8.51259 | 6.10248 | 7.44371 | 8.20524 | 9.06017 | 7.75765 | 9.13919 | 9.40072 |
| 6.60495 | 6.66792 | 7.14654 | 7.36493 | 6.60031 | 5.76203 | 5.21344 | 4.87265 |
| 6.60725 | 8.0933 | 8.94762 | 8.59522 | 7.3509 | 7.97088 | 7.95277 | 5.58519 |
| 5.96941 | 8.42009 | 6.92299 | 8.79647 | 5.53032 | 7.88346 | 3.94647 | 4.73858 |
| 2.57393 | 2.99967 | 3.54975 | 4.40893 | 2.65002 | 2.71915 | 2.71014 | 3.02711 |
| 4.65133 | 6.15856 | 3.92032 | 7.84744 | 3.17476 | 3.73508 | 3.06019 | 2.97786 |
| 10.1037 | 9.10885 | 9.09333 | 7.35228 | 4.47826 | 8.08998 | 4.7848 | 6.40854 |
| 2.97248 | 4.41685 | 5.83348 | 4.52668 | 2.89285 | 3.94884 | 3.33213 | 5.66217 |
| 2.77581 | 3.4603 | 3.05334 | 2.83032 | 2.8251 | 2.94834 | 2.68749 | 3.74105 |
| 8.08043 | 5.88517 | 6.35716 | 3.67936 | 3.02436 | 5.59702 | 5.4277 | 3.07708 |
| 3.62235 | 3.49024 | 3.36585 | 3.45826 | 3.83188 | 4.32337 | 3.62742 | 9.87828 |
| 5.29834 | 3.08666 | 2.93514 | 3.09694 | 3.29855 | 2.7714 | 3.21663 | 4.06101 |
| 7.85684 | 3.66144 | 3.31027 | 3.37747 | 3.89108 | 3.05065 | 3.15134 | 3.26281 |

| | | | | | | | |
|---|---|---|---|---|---|---|---|
| 3.07305 | 2.97399 | 3.58163 | 2.86004 | 2.94852 | 2.78104 | 2.92427 | 4.25232 |
| 3.62772 | 5.31406 | 3.62775 | 3.74515 | 3.51175 | 4.50399 | 3.73091 | 9.68214 |
| 5.83813 | 8.21476 | 7.67548 | 6.85616 | 3.78038 | 7.84805 | 6.89752 | 4.67834 |
| 2.87765 | 3.37367 | 3.7238 | 7.21397 | 3.35938 | 3.62591 | 3.09384 | 4.18496 |
| 5.68938 | 6.30988 | 8.26879 | 7.42486 | 4.33741 | 5.86868 | 3.6839 | 4.24303 |
| 9.5796 | 9.81151 | 7.05919 | 9.5317 | 9.54875 | 9.45846 | 11.4473 | 5.56597 |
| 6.57426 | 6.66721 | 6.91565 | 7.52499 | 8.14383 | 6.85951 | 7.03462 | 6.66821 |
| 2.92124 | 6.7117 | 6.11108 | 3.86624 | 3.83792 | 2.77288 | 2.88918 | 5.73891 |
| 3.51293 | 3.46948 | 3.29394 | 3.30524 | 7.63676 | 9.16315 | 9.61896 | 3.52862 |
| 11.112 | 10.8731 | 9.84094 | 10.1547 | 3.3987 | 10.5211 | 7.47548 | 5.37039 |
| 8.7134 | 9.25365 | 8.13874 | 9.48811 | 7.34785 | 8.84908 | 7.36751 | 7.51211 |
| 2.97438 | 3.1804 | 9.53337 | 5.39083 | 8.24183 | 8.72045 | 7.3221 | 8.51783 |
| 8.6232 | 6.2215 | 6.08481 | 8.31391 | 6.54487 | 7.49706 | 6.58365 | 6.52485 |
| 3.09902 | 2.97645 | 6.88687 | 3.17605 | 3.17555 | 8.01852 | 3.25122 | 3.00849 |
| 3.4189 | 3.3254 | 3.12359 | 10.7715 | 3.34189 | 3.43943 | 3.35281 | 3.53848 |
| 6.16439 | 5.69669 | 10.6048 | 5.68736 | 3.91174 | 7.22603 | 7.59431 | 10.4147 |
| 9.31329 | 10.462 | 10.2237 | 9.46065 | 9.75871 | 11.0132 | 3.61623 | 4.41125 |
| 5.90835 | 6.78257 | 7.62564 | 4.30812 | 3.45936 | 3.78579 | 3.13589 | 2.97999 |
| 3.54834 | 9.34328 | 12.5493 | 8.23241 | 3.30156 | 8.97306 | 6.38474 | 7.35168 |
| 3.30464 | 7.77585 | 9.0381 | 4.48339 | 3.6699 | 3.78844 | 3.34648 | 5.04349 |
| 9.8912 | 8.10505 | 7.91804 | 9.31403 | 7.53998 | 7.28929 | 7.38362 | 6.70653 |
| 5.01671 | 4.13676 | 9.67074 | 6.63012 | 5.46365 | 5.71648 | 5.85555 | 9.88054 |
| 9.1665 | 7.85089 | 7.54876 | 8.53643 | 8.93257 | 7.5468 | 8.01373 | 4.23121 |
| 12.1015 | 12.0737 | 10.1775 | 12.9138 | 12.0488 | 12.287 | 8.20287 | 2.65471 |
| 3.09201 | 3.54783 | 3.19858 | 4.85716 | 5.26305 | 6.33416 | 6.78747 | 7.14276 |
| 6.74632 | 6.97575 | 7.42912 | 5.47183 | 8.56157 | 7.51943 | 7.5738 | 8.42324 |
| 7.90414 | 3.9872 | 8.7456 | 7.8865 | 9.63499 | 6.58046 | 8.88343 | 9.45897 |
| 5.06658 | 5.97328 | 5.1449 | 4.24755 | 6.22407 | 5.57094 | 3.91948 | 3.86056 |
| 9.56604 | 8.78606 | 9.22046 | 5.70797 | 10.2339 | 10.3492 | 7.87324 | 11.3253 |
| 6.16576 | 3.58426 | 7.18411 | 5.84331 | 8.14812 | 5.51529 | 6.32278 | 8.36538 |
| 5.74214 | 6.91475 | 7.49714 | 4.60712 | 7.02976 | 7.45018 | 6.61487 | 3.36295 |
| 7.25225 | 4.07616 | 8.61194 | 6.15618 | 9.06313 | 7.16432 | 8.45147 | 9.59544 |
| 8.63214 | 10.7643 | 6.69756 | 11.2542 | 7.83981 | 6.39217 | 7.01107 | 6.16215 |
| 5.68525 | 3.28692 | 5.47462 | 3.42322 | 2.9159 | 3.42227 | 3.18801 | 2.85003 |
| 2.66312 | 3.50817 | 6.09072 | 3.26267 | 3.09567 | 3.30755 | 6.72759 | 7.18245 |
| 3.84644 | 4.47636 | 6.18105 | 3.45708 | 3.78803 | 5.27914 | 4.48209 | 5.35973 |
| 5.51601 | 5.45232 | 6.62061 | 3.34117 | 6.08591 | 6.49927 | 5.52477 | 6.61767 |
| 5.81398 | 3.67815 | 5.46088 | 6.32558 | 7.69436 | 5.19791 | 8.2282 | 7.79012 |
| 11.0665 | 9.38127 | 8.57805 | 9.71809 | 8.44045 | 8.01791 | 8.93615 | 8.20045 |
| 7.39383 | 3.88486 | 6.68997 | 6.81299 | 8.11738 | 5.05846 | 6.82172 | 7.53158 |
| 3.50664 | 3.24406 | 4.00431 | 3.24596 | 3.18854 | 8.44946 | 3.26525 | 4.46506 |
| 5.97006 | 3.75803 | 6.95151 | 6.28279 | 8.38921 | 5.74536 | 7.39126 | 7.68109 |
| 6.45944 | 5.61815 | 6.60697 | 6.82097 | 3.046 | 4.41106 | 5.57192 | 2.79411 |
| 3.59421 | 3.24984 | 3.15136 | 3.20533 | 3.19834 | 8.43521 | 6.65977 | 8.00077 |
| 9.94487 | 9.95574 | 8.17481 | 10.7798 | 9.33367 | 10.4968 | 9.91668 | 6.79642 |
| 3.34415 | 4.03976 | 3.05716 | 8.68585 | 3.01035 | 3.51286 | 10.8216 | 7.41166 |
| 8.90483 | 7.2374 | 7.5589 | 8.29219 | 7.31584 | 7.71207 | 6.9515 | 7.96373 |
| 6.78727 | 4.76338 | 5.78612 | 5.13189 | 3.09653 | 3.54652 | 2.83283 | 3.28455 |
| 3.73741 | 3.03882 | 5.60544 | 2.93135 | 4.09129 | 3.35962 | 3.3151 | 5.73785 |
| 6.09101 | 3.97627 | 5.27187 | 8.18411 | 5.32186 | 6.99044 | 4.72747 | 7.73153 |
| 9.0455 | 9.49886 | 9.94763 | 3.3676 | 9.85134 | 9.41868 | 9.92944 | 10.1414 |
| 7.41273 | 4.88947 | 7.37393 | 7.37024 | 5.31483 | 6.28059 | 3.38548 | 5.40677 |
| 10.295 | 8.08571 | 10.076 | 4.98843 | 6.77279 | 7.42063 | 3.01254 | 6.77638 |

| | | | | | | | |
|---|---|---|---|---|---|---|---|
| 7.28333 | 7.172 | 3.97525 | 5.81591 | 6.2866 | 6.18804 | 5.03438 | 3.71683 |
| 7.33195 | 8.02052 | 8.93657 | 6.54178 | 8.64388 | 9.0389 | 7.04965 | 3.26233 |
| 6.96997 | 5.51349 | 9.12133 | 7.07282 | 5.73853 | 7.50973 | 5.95722 | 3.82737 |
| 5.81545 | 5.64827 | 4.29901 | 5.97544 | 3.28969 | 4.31087 | 4.39643 | 3.60223 |
| 9.70003 | 9.6514 | 10.2463 | 9.77744 | 8.95573 | 10.6303 | 9.21101 | 9.55609 |
| 7.27299 | 7.4917 | 5.79945 | 7.97329 | 7.28601 | 6.14342 | 6.19511 | 8.52488 |
| 10.3467 | 11.3947 | 9.35154 | 8.64839 | 3.72799 | 10.5291 | 3.81867 | 9.41558 |
| 7.88324 | 8.27467 | 5.51849 | 10.1272 | 8.17908 | 6.82534 | 9.90687 | 8.37333 |
| 8.732 | 10.7931 | 10.7888 | 8.01788 | 9.28548 | 9.6736 | 10.2841 | 9.24852 |
| 7.81235 | 7.84571 | 7.92773 | 5.97492 | 5.58983 | 7.99672 | 8.20574 | 6.3559 |
| 3.206 | 2.90496 | 3.5093 | 3.07905 | 2.95653 | 3.4165 | 5.04183 | 3.64169 |
| 7.56908 | 7.96636 | 8.67169 | 7.09431 | 7.21462 | 7.88243 | 3.72408 | 3.05412 |
| 8.62954 | 6.40165 | 7.44048 | 4.49989 | 9.08436 | 6.11815 | 8.61074 | 8.27705 |
| 6.84826 | 7.96127 | 7.21568 | 8.38299 | 6.87389 | 7.01956 | 6.85239 | 8.29093 |
| 5.79141 | 7.21302 | 5.13237 | 7.85707 | 4.46896 | 5.72823 | 6.04112 | 4.31355 |
| 5.34094 | 4.46822 | 3.07087 | 5.12942 | 3.33418 | 3.78932 | 2.95334 | 2.86888 |
| 8.58443 | 6.00383 | 5.8601 | 7.67812 | 4.29187 | 6.92665 | 5.99905 | 4.84186 |
| 9.79473 | 7.47693 | 6.29082 | 8.80999 | 5.32463 | 7.98967 | 7.84952 | 6.61888 |
| 3.50735 | 4.04273 | 5.13892 | 7.06849 | 3.53501 | 6.32848 | 3.56487 | 3.32475 |
| 10.5614 | 7.42575 | 8.09222 | 9.17434 | 5.30964 | 7.20989 | 8.86557 | 6.66278 |
| 5.52013 | 6.34773 | 8.65282 | 4.80855 | 3.9886 | 7.4302 | 6.63244 | 7.73841 |
| 4.00973 | 3.19932 | 2.90692 | 4.80938 | 5.85444 | 4.45414 | 8.49837 | 8.20305 |
| 4.74577 | 5.09691 | 5.4968 | 4.49116 | 4.36738 | 4.74129 | 6.09481 | 6.3639 |
| 3.39827 | 4.12131 | 9.14859 | 4.46175 | 3.52788 | 4.22457 | 2.90515 | 8.13329 |
| 8.29714 | 10.1153 | 8.21366 | 4.47739 | 7.74314 | 10.2751 | 5.79381 | 8.42163 |
| 5.43532 | 3.23854 | 3.07638 | 3.31502 | 4.21166 | 7.6701 | 7.18116 | 3.2586 |
| 12.3022 | 7.65348 | 8.95628 | 11.4603 | 10.1928 | 11.6559 | 9.06943 | 6.69913 |
| 6.85866 | 7.56798 | 5.3012 | 7.46221 | 6.75989 | 6.56581 | 6.0458 | 4.58808 |
| 3.73013 | 4.1719 | 7.92163 | 3.45467 | 4.36855 | 5.27147 | 6.17494 | 6.4103 |
| 3.26675 | 5.25346 | 5.6836 | 2.93031 | 3.10982 | 3.22396 | 2.94016 | 2.80548 |
| 2.90669 | 3.46956 | 3.03623 | 9.27853 | 2.78052 | 3.74843 | 7.44088 | 2.69692 |
| 8.06831 | 9.0274 | 10.3713 | 7.80694 | 7.80236 | 8.64635 | 8.44682 | 7.1958 |
| 10.4844 | 9.63309 | 10.9882 | 10.6463 | 8.64653 | 10.5496 | 5.37952 | 10.7216 |
| 8.22685 | 3.49446 | 6.97804 | 3.35324 | 5.70565 | 4.91634 | 2.35764 | 5.32238 |
| 8.43707 | 6.10342 | 6.44873 | 6.26491 | 4.56567 | 7.91956 | 4.38319 | 5.76668 |
| 12.5231 | 11.8423 | 11.0299 | 12.7346 | 8.7756 | 10.9967 | 11.9863 | 6.40609 |
| 8.88054 | 7.73089 | 4.95344 | 7.88967 | 3.53087 | 7.63419 | 6.06233 | 2.88656 |
| 7.84547 | 4.23646 | 7.0973 | 5.65659 | 6.4231 | 5.1049 | 4.45095 | 3.89965 |
| 4.22449 | 4.07112 | 7.81832 | 3.45076 | 4.93337 | 5.15908 | 3.94242 | 5.67455 |
| 10.0324 | 8.84435 | 10.3756 | 8.69929 | 9.30844 | 9.49274 | 5.54523 | 10.3744 |
| 9.0042 | 8.99769 | 6.81777 | 8.41237 | 6.93166 | 9.10085 | 7.60524 | 7.44361 |
| 8.36231 | 9.72072 | 9.17118 | 3.00996 | 9.65512 | 9.81178 | 9.5946 | 8.72082 |
| 6.49819 | 4.14119 | 6.59172 | 6.73688 | 5.27125 | 5.76179 | 3.57801 | 4.31009 |
| 9.33886 | 8.25461 | 8.40959 | 5.10964 | 3.59686 | 9.32594 | 2.69167 | 6.93013 |
| 9.6336 | 10.4722 | 10.4806 | 10.7263 | 9.42519 | 10.1033 | 10.8375 | 3.25804 |
| 11.9011 | 11.9484 | 11.5193 | 12.0453 | 11.3685 | 12.3078 | 11.3825 | 7.59284 |
| 6.78375 | 3.86681 | 9.65521 | 4.87014 | 5.44637 | 6.41142 | 3.09825 | 4.52714 |
| 12.0295 | 11.2888 | 9.20643 | 11.4904 | 10.7326 | 11.4098 | 9.87391 | 8.58056 |
| 7.38464 | 6.1418 | 7.48524 | 5.86359 | 4.74877 | 5.76691 | 4.58893 | 4.70194 |
| 5.32823 | 4.06432 | 8.01426 | 7.71135 | 4.1616 | 3.56165 | 5.08308 | 6.14119 |
| 3.40337 | 3.27502 | 3.93409 | 3.00051 | 2.89173 | 6.47416 | 3.10844 | 6.6682 |
| 8.60124 | 6.9987 | 9.55176 | 6.63839 | 6.65399 | 8.77784 | 9.42282 | 9.31394 |
| 11.7474 | 10.8323 | 10.6796 | 7.75378 | 8.25296 | 10.9105 | 8.42814 | 7.32287 |

| | | | | | | | |
|---|---|---|---|---|---|---|---|
| 9.74034 | 7.67552 | 8.83194 | 6.94956 | 5.25837 | 9.6368 | 4.65426 | 3.507 |
| 8.16533 | 5.49435 | 7.4335 | 6.48517 | 5.03386 | 8.34307 | 3.78329 | 4.0157 |
| 12.995 | 11.8089 | 11.752 | 9.18542 | 7.81056 | 12.848 | 5.18517 | 3.66417 |
| 9.85722 | 7.73568 | 8.7627 | 8.33719 | 3.08078 | 10.1415 | 3.17928 | 4.12534 |
| 4.04821 | 5.32075 | 7.85716 | 3.62859 | 3.26093 | 3.76271 | 3.46735 | 3.24361 |
| 8.48728 | 4.07677 | 7.69214 | 3.46987 | 3.53154 | 3.51747 | 3.29556 | 3.25012 |
| 12.4061 | 8.75134 | 11.6409 | 3.13683 | 7.28695 | 12.2706 | 2.95487 | 3.53693 |
| 11.9308 | 8.43571 | 10.7023 | 3.08695 | 4.67887 | 11.7903 | 3.51691 | 2.86837 |
| 10.7411 | 6.98817 | 3.22008 | 3.20276 | 3.23267 | 11.4559 | 3.06234 | 2.90503 |
| 10.646 | 4.26518 | 9.4122 | 3.07925 | 3.1826 | 3.75279 | 3.12691 | 2.99876 |
| 6.03488 | 3.03784 | 7.08893 | 4.51941 | 8.77606 | 6.07864 | 8.21069 | 8.02083 |
| 2.88455 | 3.53851 | 3.74068 | 3.88347 | 2.69091 | 3.42239 | 4.28241 | 3.06707 |
| 6.08632 | 6.69928 | 5.32532 | 5.25512 | 2.82318 | 5.35435 | 4.35386 | 3.80799 |
| 5.90254 | 6.3411 | 7.20719 | 6.44537 | 5.53915 | 5.7029 | 4.09274 | 5.64605 |
| 7.59389 | 7.70583 | 7.10304 | 7.92536 | 6.43868 | 7.59172 | 7.54648 | 5.54407 |
| 11.1663 | 12.2663 | 10.5688 | 10.4148 | 10.565 | 9.78732 | 8.49736 | 10.8741 |
| 10.5462 | 3.59122 | 3.32022 | 9.38004 | 7.4359 | 8.04879 | 8.54514 | 6.65593 |
| 11.8246 | 9.34511 | 9.63915 | 8.85479 | 8.5858 | 9.34315 | 10.3421 | 9.02746 |
| 9.79949 | 6.54441 | 10.28 | 8.39073 | 7.40795 | 8.12474 | 6.95415 | 5.43477 |
| 9.18009 | 3.68538 | 9.43471 | 5.43952 | 7.85896 | 5.91843 | 6.74009 | 10.4411 |
| 11.2669 | 4.35618 | 7.95849 | 7.85035 | 7.87144 | 9.92447 | 3.10589 | 9.8385 |
| 12.7658 | 6.93858 | 8.46052 | 10.6925 | 5.42408 | 11.9152 | 7.62511 | 5.69664 |
| 5.07634 | 5.76527 | 7.0397 | 3.13398 | 7.04882 | 6.6742 | 7.12756 | 7.11752 |
| 5.93347 | 8.85359 | 10.4356 | 6.37853 | 4.01567 | 8.72572 | 3.5965 | 10.141 |
| 2.96864 | 3.42505 | 3.15855 | 8.77849 | 3.20371 | 9.43172 | 4.51915 | 2.81899 |
| 3.22668 | 3.14626 | 4.03192 | 3.11642 | 3.85089 | 4.9754 | 2.84511 | 4.73863 |
| 3.20835 | 3.77831 | 3.25119 | 3.34424 | 3.30376 | 3.36576 | 3.56299 | 3.41639 |
| 3.27117 | 3.10436 | 9.25464 | 3.03889 | 3.30071 | 3.36195 | 3.27967 | 3.69131 |
| 6.00274 | 4.70092 | 3.78554 | 6.04072 | 5.04742 | 5.33166 | 4.11509 | 4.52657 |
| 4.66236 | 4.62658 | 4.86914 | 5.11445 | 6.47172 | 6.29176 | 5.90732 | 6.33828 |
| 8.83326 | 9.13791 | 8.41238 | 8.96864 | 7.21387 | 8.10057 | 6.40008 | 7.02538 |
| 3.15874 | 3.3316 | 3.45344 | 3.07296 | 3.6585 | 3.06937 | 7.21104 | 5.18519 |
| 7.33096 | 3.21938 | 2.89984 | 6.86758 | 3.24773 | 3.62902 | 3.28004 | 4.99745 |
| 7.79231 | 7.30944 | 8.11265 | 7.09435 | 8.91753 | 7.66934 | 6.26307 | 9.57868 |
| 8.07526 | 7.44178 | 7.9457 | 7.51413 | 5.90745 | 6.14356 | 4.39321 | 7.66483 |
| 5.96092 | 7.61776 | 9.82577 | 6.5204 | 7.22941 | 7.00192 | 6.12924 | 7.96416 |
| 3.95819 | 3.7566 | 7.15193 | 3.45819 | 8.13691 | 7.8215 | 3.49063 | 3.95964 |
| 6.5735 | 3.10767 | 4.74137 | 3.60881 | 2.84509 | 3.71477 | 2.96258 | 2.90423 |
| 4.33483 | 4.04988 | 4.1356 | 4.43958 | 5.14596 | 4.14686 | 6.39135 | 6.36799 |
| 9.01985 | 6.7095 | 6.33869 | 5.73773 | 6.45621 | 6.66364 | 5.39331 | 5.83228 |
| 9.53938 | 8.85016 | 10.169 | 8.51074 | 9.67093 | 10.0306 | 8.13353 | 7.53159 |
| 5.07023 | 7.05278 | 5.71628 | 3.01153 | 3.16145 | 3.20193 | 6.28387 | 3.16219 |
| 6.09386 | 3.37217 | 6.18821 | 3.51404 | 7.11307 | 4.13467 | 6.77145 | 8.15095 |
| 4.6974 | 3.69569 | 5.81664 | 3.67628 | 7.44365 | 4.83384 | 5.54052 | 6.74511 |
| 6.1017 | 3.27665 | 3.22388 | 4.81335 | 3.10481 | 3.0996 | 2.92369 | 2.70392 |
| 7.49885 | 7.58433 | 8.11581 | 3.2801 | 7.65529 | 8.06864 | 7.99275 | 8.09915 |
| 8.59447 | 6.75651 | 5.49996 | 6.18702 | 6.64655 | 6.30494 | 5.67785 | 6.24808 |
| 3.53021 | 3.53695 | 2.91604 | 3.24843 | 3.40546 | 3.23538 | 3.18979 | 5.4404 |
| 8.12556 | 8.576 | 8.14959 | 7.93903 | 6.52339 | 7.44669 | 6.79767 | 6.91 |
| 7.87424 | 8.04639 | 4.46556 | 7.07101 | 5.69636 | 7.80118 | 7.41089 | 5.85157 |
| 2.71936 | 3.07742 | 10.4315 | 2.85946 | 2.6457 | 3.06591 | 2.99292 | 3.17817 |
| 3.78569 | 9.44831 | 3.49395 | 3.27075 | 7.21744 | 6.71493 | 3.47345 | 3.46757 |
| 4.80351 | 5.50856 | 5.47589 | 3.61502 | 3.17555 | 8.61278 | 3.74051 | 3.60327 |

| | | | | | | | |
|---|---|---|---|---|---|---|---|
| 11.5919 | 8.2893 | 10.5121 | 3.63475 | 4.94288 | 11.5439 | 3.81691 | 3.23021 |
| 13.2232 | 12.7472 | 11.6467 | 13.1294 | 9.7468 | 11.6144 | 12.3556 | 7.11917 |
| 11.4509 | 7.55755 | 8.42589 | 3.33407 | 3.73683 | 11.1023 | 3.08997 | 3.12419 |
| 7.22782 | 7.75316 | 7.72443 | 3.58743 | 3.18848 | 11.7329 | 3.74932 | 3.23447 |
| 5.90155 | 8.48758 | 4.1367 | 9.59528 | 5.89238 | 6.9565 | 9.84526 | 3.06001 |
| 8.51234 | 9.1047 | 4.26717 | 8.08825 | 8.07102 | 7.73206 | 4.21137 | 3.4333 |
| 3.5606 | 8.81792 | 5.07332 | 5.24709 | 9.09797 | 7.91496 | 11.0632 | 6.54949 |
| 9.90276 | 6.086 | 3.45462 | 8.07596 | 3.13618 | 3.23997 | 3.34679 | 2.89566 |
| 5.65803 | 6.05134 | 6.24566 | 2.97209 | 6.85347 | 3.75787 | 3.71322 | 7.74312 |
| 9.65143 | 9.99326 | 8.13257 | 8.33222 | 9.59405 | 5.11972 | 4.18217 | 3.93403 |
| 2.77549 | 4.30035 | 2.97872 | 6.99759 | 3.08946 | 5.89023 | 7.41845 | 2.79249 |
| 9.99479 | 9.68002 | 7.15144 | 8.75226 | 2.87647 | 7.47132 | 7.80795 | 2.61545 |
| 6.23015 | 6.46985 | 6.90167 | 8.23923 | 5.59324 | 3.981 | 3.79584 | 4.03031 |
| 3.50843 | 3.06139 | 9.56808 | 6.34858 | 9.19993 | 8.65679 | 7.74969 | 8.59063 |
| 6.97164 | 12.2158 | 12.199 | 11.1028 | 12.1673 | 11.6361 | 10.7821 | 11.5599 |
| 3.20807 | 3.65889 | 3.38046 | 3.38587 | 3.36702 | 3.27943 | 3.31443 | 3.07666 |
| 2.95609 | 2.84411 | 9.49709 | 3.05088 | 2.84892 | 10.2336 | 2.876 | 3.30071 |
| 4.47398 | 4.03023 | 6.70946 | 5.74783 | 4.73833 | 3.90629 | 4.25821 | 5.20799 |
| 6.28349 | 3.38489 | 3.40025 | 8.83669 | 3.32997 | 3.27812 | 3.52996 | 7.32036 |
| 6.13388 | 6.86167 | 7.59536 | 7.29741 | 6.57555 | 6.10019 | 4.79024 | 3.71065 |
| 2.86783 | 3.16598 | 4.10638 | 3.61811 | 4.14634 | 2.75234 | 4.7848 | 3.03422 |
| 8.36929 | 7.51784 | 5.08081 | 5.07668 | 4.46399 | 8.25636 | 6.13569 | 8.38441 |
| 5.46376 | 6.09261 | 8.24971 | 7.499 | 4.36943 | 6.27384 | 5.37755 | 4.22094 |
| 3.32859 | 5.13499 | 3.17059 | 5.68627 | 3.10633 | 3.44768 | 3.42157 | 2.83252 |
| 3.63689 | 3.51477 | 3.26834 | 4.01379 | 5.18662 | 4.37604 | 6.54549 | 4.79739 |
| 9.09162 | 4.12089 | 7.17254 | 7.05857 | 9.2427 | 8.98184 | 6.13716 | 5.22985 |
| 8.76135 | 10.0255 | 10.4952 | 9.78537 | 3.65362 | 10.2128 | 3.19794 | 12.4172 |
| 4.94448 | 8.79872 | 7.43802 | 11.3696 | 7.90052 | 7.36035 | 9.92024 | 10.5112 |
| 9.90679 | 10.8739 | 10.7745 | 10.7902 | 7.81724 | 9.95886 | 4.39351 | 11.0621 |
| 5.21722 | 5.05527 | 4.7351 | 5.33024 | 7.72921 | 3.49171 | 6.32426 | 7.89262 |
| 2.86698 | 2.92911 | 2.94443 | 4.49089 | 3.01042 | 2.99967 | 2.78446 | 2.98381 |
| 8.05669 | 3.64513 | 7.58857 | 3.1332 | 9.17884 | 5.91508 | 7.68565 | 9.03048 |
| 11.3903 | 13.0984 | 5.41767 | 12.7706 | 11.4256 | 11.1211 | 13.1821 | 3.63889 |
| 4.16927 | 4.07185 | 3.94599 | 9.08784 | 3.21074 | 5.66133 | 10.2512 | 3.46624 |
| 3.1566 | 11.6469 | 6.89677 | 3.39985 | 6.84684 | 3.32131 | 3.24781 | 11.0473 |
| 8.20662 | 10.0378 | 10.2408 | 6.73825 | 6.7502 | 5.46715 | 5.84083 | 3.69317 |
| 6.73783 | 3.78631 | 5.89771 | 7.08232 | 6.26774 | 6.37239 | 3.16291 | 3.63307 |
| 7.26454 | 7.5407 | 7.2231 | 6.87121 | 8.12493 | 7.14748 | 7.50923 | 5.00367 |
| 6.86779 | 5.35171 | 2.91459 | 2.9572 | 5.17333 | 8.58473 | 3.99027 | 9.7146 |
| 8.50731 | 8.67344 | 8.0328 | 8.63742 | 8.06287 | 7.47446 | 6.96357 | 7.90613 |
| 5.77513 | 9.20354 | 9.95184 | 7.70815 | 8.43801 | 9.04435 | 8.24687 | 4.66296 |
| 3.54824 | 3.61 | 8.15985 | 3.44026 | 3.27283 | 3.45662 | 3.47197 | 8.58836 |
| 3.29755 | 3.31302 | 7.66358 | 8.53141 | 7.42069 | 3.16454 | 4.17297 | 3.16516 |
| 8.54139 | 8.86024 | 9.39979 | 3.50387 | 10.0165 | 7.53286 | 8.86491 | 10.6435 |
| 3.85274 | 2.8987 | 2.98348 | 2.82438 | 2.90728 | 2.99993 | 8.42843 | 2.9259 |
| 5.05891 | 9.79004 | 8.30891 | 10.5187 | 5.60424 | 8.41229 | 10.1556 | 5.11021 |
| 7.61207 | 3.2214 | 8.24539 | 7.3783 | 6.54451 | 6.57187 | 3.24781 | 5.45475 |
| 6.37809 | 4.53184 | 7.43947 | 4.99225 | 9.12759 | 5.61298 | 7.99888 | 8.73704 |
| 4.06257 | 3.08059 | 4.55467 | 3.71713 | 5.58021 | 4.13784 | 5.68193 | 6.398 |
| 7.82871 | 7.73778 | 7.12705 | 7.99551 | 3.396 | 8.88901 | 7.19283 | 3.43759 |
| 9.99265 | 9.94559 | 9.1754 | 6.91924 | 10.0218 | 10.8878 | 5.63803 | 9.83735 |
| 6.37323 | 4.51614 | 5.44096 | 6.92574 | 3.33092 | 5.98631 | 4.51717 | 3.02489 |
| 8.40649 | 6.38686 | 5.94446 | 7.87515 | 6.28778 | 7.01011 | 7.29446 | 5.00273 |

| | | | | | | | |
|---|---|---|---|---|---|---|---|
| 3.63348 | 5.16821 | 4.34436 | 4.07187 | 5.0268 | 3.69775 | 5.03871 | 6.5741 |
| 5.00562 | 3.04941 | 9.31263 | 2.9093 | 2.93371 | 5.42052 | 2.94216 | 2.88684 |
| 9.28882 | 8.79453 | 7.5775 | 6.88405 | 8.78795 | 3.75018 | 8.37724 | 3.43362 |
| 9.01181 | 3.26656 | 7.68926 | 3.16289 | 7.69807 | 5.24562 | 3.38433 | 6.89033 |
| 8.26694 | 6.65235 | 6.76731 | 7.40243 | 7.25959 | 4.84172 | 5.66828 | 5.45664 |
| 6.58991 | 4.1473 | 8.58966 | 7.17306 | 6.41216 | 5.87056 | 3.19858 | 5.63127 |
| 8.74445 | 7.84098 | 8.01036 | 8.50165 | 8.34235 | 7.9941 | 8.11912 | 5.99498 |
| 5.79999 | 3.27318 | 6.8369 | 5.2251 | 7.51229 | 5.60761 | 7.76636 | 8.96375 |
| 8.47746 | 11.4591 | 6.49056 | 9.65516 | 9.18647 | 11.0817 | 9.12943 | 3.49332 |
| 5.80281 | 5.10601 | 5.47117 | 6.48143 | 6.39216 | 4.03719 | 4.23945 | 2.87112 |
| 3.20597 | 2.91361 | 3.63736 | 3.3673 | 3.10729 | 3.51376 | 4.70107 | 4.00346 |
| 7.86325 | 4.61625 | 4.57608 | 8.01389 | 3.69527 | 6.41477 | 3.31423 | 5.56187 |
| 12.6099 | 13.0063 | 12.288 | 12.7023 | 12.7905 | 12.6299 | 11.9609 | 12.2313 |
| 3.42242 | 3.36782 | 3.18573 | 3.07503 | 3.09687 | 5.29425 | 4.64732 | 3.68086 |
| 4.08647 | 3.5934 | 5.25209 | 3.79894 | 3.86037 | 4.08788 | 3.94963 | 5.04486 |
| 7.12752 | 7.39134 | 5.63334 | 5.39995 | 4.2328 | 6.13559 | 3.98849 | 5.94585 |
| 7.88286 | 6.9044 | 3.20833 | 4.05624 | 3.23075 | 3.51462 | 4.43445 | 5.19435 |
| 7.92297 | 6.58254 | 2.90241 | 4.18271 | 2.75 | 2.83877 | 4.5771 | 5.92838 |
| 6.78392 | 7.94086 | 2.78889 | 3.49335 | 3.02407 | 2.82143 | 3.09002 | 4.38798 |
| 3.81051 | 3.32088 | 4.78192 | 2.87836 | 2.99574 | 5.6272 | 3.18419 | 4.84332 |
| 5.66673 | 6.50054 | 6.55427 | 5.8667 | 6.1523 | 4.62983 | 4.84068 | 7.30217 |
| 10.5815 | 11.2844 | 8.89915 | 10.7727 | 10.5097 | 10.5418 | 9.24802 | 10.3037 |
| 6.96224 | 8.04549 | 8.05814 | 5.11554 | 8.28214 | 8.66141 | 9.16915 | 8.44055 |
| 5.69645 | 5.96918 | 4.48471 | 4.66384 | 2.66064 | 3.9828 | 2.43413 | 2.65117 |
| 7.81045 | 5.54875 | 7.95229 | 7.34316 | 5.52643 | 7.4443 | 9.59342 | 9.58773 |
| 10.5017 | 10.6859 | 6.86157 | 10.7276 | 8.22101 | 11.6073 | 9.0706 | 5.48972 |
| 10.4922 | 9.99773 | 9.3122 | 10.9659 | 10.0575 | 10.3803 | 10.3258 | 7.94069 |
| 10.4519 | 9.19181 | 8.26529 | 11.7133 | 3.99756 | 8.50286 | 8.27218 | 3.07187 |
| 3.10613 | 3.06496 | 3.21292 | 3.34573 | 3.19546 | 3.05687 | 2.93083 | 3.88259 |
| 8.74962 | 9.71111 | 8.36296 | 7.09482 | 5.75539 | 8.29339 | 3.95902 | 7.90271 |
| 3.52917 | 4.33046 | 5.57283 | 5.03139 | 5.978 | 5.06019 | 6.61437 | 3.78733 |
| 8.4506 | 7.72741 | 4.89316 | 7.4945 | 8.81795 | 6.5638 | 9.77921 | 7.36662 |
| 5.06132 | 4.48346 | 6.68998 | 8.65405 | 3.91499 | 4.30683 | 3.84449 | 3.42081 |
| 4.8892 | 3.67961 | 4.99035 | 4.06808 | 6.67217 | 4.52495 | 6.5053 | 6.87751 |
| 2.75392 | 3.12644 | 2.61907 | 2.64231 | 2.65077 | 2.74647 | 7.45557 | 2.77938 |
| 7.49221 | 5.86721 | 7.87967 | 6.21758 | 7.49612 | 6.75878 | 7.28466 | 8.4383 |
| 11.3257 | 11.6484 | 11.1645 | 11.1087 | 10.9882 | 11.7755 | 11.0717 | 9.29476 |
| 9.20424 | 8.45106 | 8.6469 | 8.2392 | 6.57077 | 7.99411 | 7.06334 | 6.73451 |
| 2.92661 | 5.14481 | 8.48485 | 9.14458 | 3.06211 | 3.00444 | 5.36679 | 3.10837 |
| 7.83699 | 8.19612 | 7.94173 | 7.9873 | 6.31051 | 7.53954 | 7.06298 | 6.40737 |
| 2.87765 | 8.25486 | 2.88044 | 2.89208 | 3.12817 | 4.3481 | 3.03529 | 7.93597 |
| 7.0914 | 7.07196 | 7.76034 | 3.09694 | 8.06562 | 7.41034 | 7.49743 | 8.30972 |
| 8.59713 | 4.42615 | 6.99472 | 6.54734 | 3.86161 | 6.68134 | 3.70419 | 6.06861 |
| 9.61326 | 10.559 | 8.71387 | 7.7111 | 5.97641 | 4.09771 | 8.67032 | 3.64839 |
| 9.06007 | 10.0808 | 3.04741 | 9.28176 | 3.60306 | 6.009 | 2.90515 | 3.23962 |
| 4.53566 | 3.74657 | 3.59372 | 3.37781 | 3.67299 | 3.71093 | 3.89412 | 7.19894 |
| 3.53895 | 3.99617 | 6.80122 | 5.31555 | 3.4853 | 5.50895 | 3.4877 | 6.34319 |
| 8.79462 | 6.61419 | 6.41064 | 6.84513 | 7.18351 | 7.2944 | 6.58317 | 5.8575 |
| 8.48332 | 5.15533 | 4.66107 | 4.69595 | 3.38698 | 4.32057 | 3.33227 | 5.6273 |
| 9.86152 | 10.1921 | 10.4693 | 7.93789 | 7.39663 | 9.2575 | 5.07717 | 9.83522 |
| 8.49669 | 4.98416 | 3.50502 | 10.0028 | 3.62428 | 7.10724 | 3.25708 | 5.34729 |
| 4.26038 | 5.31091 | 2.66119 | 7.80152 | 7.87815 | 9.16233 | 7.93597 | 7.68688 |
| 3.94219 | 4.5697 | 5.16593 | 4.73452 | 3.58249 | 4.99396 | 3.41507 | 7.24915 |

| | | | | | | | |
|---|---|---|---|---|---|---|---|
| 5.62496 | 2.82453 | 6.43188 | 2.47871 | 4.33824 | 3.032 | 4.91936 | 7.42838 |
| 3.4599 | 4.15776 | 10.199 | 3.60097 | 3.58236 | 3.0946 | 3.06504 | 11.7335 |
| 3.61485 | 3.24264 | 3.61748 | 3.38715 | 3.09821 | 3.01701 | 3.05318 | 8.27913 |
| 8.12211 | 8.31481 | 8.31858 | 7.04143 | 6.83901 | 8.5405 | 7.33224 | 7.96256 |
| 3.43807 | 9.30916 | 10.0447 | 10.3416 | 6.75099 | 6.33495 | 8.77973 | 4.24029 |
| 9.7262 | 10.1828 | 8.29302 | 9.35687 | 8.30359 | 7.14735 | 8.19746 | 9.44052 |
| 10.5222 | 3.83173 | 7.98485 | 5.20032 | 3.35367 | 8.10464 | 5.01344 | 4.89044 |
| 8.5004 | 9.43819 | 7.01551 | 10.7129 | 7.13405 | 8.462 | 10.5924 | 6.47738 |
| 3.11589 | 6.85662 | 8.2864 | 4.54498 | 8.90605 | 6.53722 | 6.70187 | 8.64162 |
| 6.94954 | 3.89023 | 5.22526 | 5.70574 | 3.25424 | 3.63127 | 3.08752 | 4.23151 |
| 5.33986 | 4.07908 | 6.71054 | 6.07745 | 7.98517 | 5.73548 | 7.39732 | 8.76113 |
| 4.70077 | 3.45904 | 5.17943 | 3.13181 | 5.56609 | 6.46795 | 4.97748 | 6.60825 |
| 4.80828 | 5.10596 | 3.24744 | 7.2637 | 3.2634 | 3.56213 | 4.11223 | 3.23594 |
| 6.79784 | 4.59922 | 6.08896 | 4.83405 | 7.06148 | 7.73866 | 7.27183 | 9.118 |
| 7.48473 | 7.59777 | 7.33425 | 3.11149 | 8.18717 | 7.5789 | 7.78779 | 8.08796 |
| 3.1906 | 8.37885 | 3.30827 | 3.05787 | 3.8379 | 7.11512 | 3.21539 | 3.15461 |
| 6.23302 | 6.1087 | 5.48304 | 8.01464 | 6.27094 | 5.73933 | 8.76005 | 8.27644 |
| 2.99505 | 2.93115 | 2.77172 | 7.39957 | 2.94263 | 2.88996 | 11.2972 | 3.17637 |
| 9.2903 | 9.44498 | 5.51849 | 4.69806 | 5.05185 | 7.13698 | 9.68569 | 9.18579 |
| 7.90597 | 7.23355 | 7.54272 | 7.00346 | 5.91299 | 7.09126 | 5.68471 | 7.49244 |
| 9.97043 | 9.97401 | 7.99879 | 9.69702 | 8.42016 | 8.65216 | 10.0898 | 6.90166 |
| 5.58999 | 5.72992 | 6.47811 | 5.63643 | 8.18046 | 4.15823 | 2.92726 | 2.74188 |
| 8.58408 | 6.55465 | 6.25914 | 2.94159 | 2.86112 | 7.27691 | 2.84448 | 5.43453 |
| 4.16327 | 4.1597 | 3.59128 | 5.49379 | 3.76755 | 7.976 | 8.96321 | 4.92305 |
| 10.0145 | 9.11716 | 9.56339 | 9.84273 | 9.69067 | 8.59774 | 8.75448 | 8.28926 |
| 8.07172 | 8.62204 | 5.95901 | 8.25021 | 6.17079 | 8.99481 | 6.0633 | 7.89332 |
| 10.9691 | 10.955 | 3.44121 | 3.65518 | 6.24764 | 12.1404 | 3.41411 | 8.35765 |
| 10.4145 | 10.0607 | 9.18435 | 9.85989 | 9.99726 | 10.2344 | 11.0612 | 7.0195 |
| 6.89002 | 5.14677 | 3.49456 | 8.30444 | 8.22785 | 5.89677 | 8.97323 | 7.25581 |
| 3.16542 | 2.96947 | 5.08561 | 3.13169 | 2.79032 | 2.88499 | 2.80069 | 3.52086 |
| 10.1706 | 10.6083 | 11.9688 | 10.552 | 9.55624 | 10.8457 | 10.4895 | 8.59337 |
| 3.07958 | 3.33563 | 12.1976 | 3.16926 | 3.42425 | 3.53815 | 3.20205 | 3.08096 |
| 2.70854 | 3.10112 | 12.7167 | 2.97954 | 2.56748 | 2.89136 | 3.31248 | 2.8498 |
| 10.2276 | 7.81156 | 9.0251 | 9.06443 | 8.48985 | 7.38646 | 8.29863 | 7.78371 |
| 5.36135 | 6.61183 | 6.53592 | 7.05564 | 2.99224 | 8.07302 | 6.62415 | 3.27059 |
| 3.41191 | 3.26088 | 4.80072 | 2.83847 | 3.04177 | 2.92724 | 3.21639 | 4.2311 |
| 2.69961 | 2.9985 | 3.03806 | 6.46874 | 3.20703 | 3.2189 | 9.2303 | 2.61764 |
| 8.90791 | 5.47707 | 7.94405 | 3.11981 | 3.11118 | 5.29104 | 3.2659 | 8.14058 |
| 8.63461 | 6.55441 | 5.49224 | 5.97278 | 9.2712 | 7.14114 | 7.31316 | 9.29108 |
| 8.22041 | 6.39783 | 7.27138 | 3.20018 | 5.13599 | 7.31011 | 2.70916 | 7.52649 |
| 8.91708 | 5.52783 | 8.24498 | 7.20409 | 4.24398 | 6.72856 | 4.73025 | 8.31677 |
| 12.687 | 7.09007 | 12.199 | 10.9104 | 3.43504 | 11.2002 | 3.56145 | 10.4213 |
| 2.99809 | 2.66567 | 4.15946 | 2.93061 | 3.0066 | 2.89114 | 2.68094 | 5.15236 |
| 4.48208 | 3.22406 | 4.15211 | 3.19715 | 2.84022 | 2.88037 | 3.02294 | 5.21131 |
| 10.2323 | 9.69776 | 10.7105 | 8.80537 | 8.8244 | 10.3134 | 8.63772 | 8.92798 |
| 9.51996 | 9.55304 | 11.2226 | 9.93666 | 10.4797 | 11.1303 | 9.49092 | 11.9534 |
| 6.02155 | 7.19099 | 7.00914 | 6.0041 | 6.26947 | 7.11287 | 4.15436 | 3.22614 |
| 4.6454 | 3.9863 | 6.56585 | 3.17459 | 7.18136 | 4.64898 | 6.27576 | 6.97251 |
| 7.76593 | 7.15812 | 5.09492 | 7.25405 | 5.85003 | 7.9945 | 6.39278 | 4.97187 |
| 5.87742 | 5.86815 | 5.38539 | 6.64821 | 5.27904 | 4.85217 | 3.15554 | 4.97471 |
| 4.83959 | 3.00559 | 8.16582 | 6.82032 | 3.01574 | 3.19668 | 3.32679 | 3.27153 |
| 3.66766 | 5.07232 | 3.57531 | 6.42432 | 6.2028 | 3.66883 | 9.82708 | 3.35241 |
| 2.88753 | 2.82469 | 6.77172 | 2.89494 | 3.00124 | 3.27071 | 3.10033 | 7.42713 |

| | | | | | | | |
|---|---|---|---|---|---|---|---|
| 3.65784 | 4.14131 | 3.48155 | 3.35066 | 6.67209 | 3.61915 | 6.8609 | 7.42521 |
| 7.98793 | 7.16293 | 8.28677 | 8.42767 | 4.83964 | 5.64499 | 6.17943 | 4.14658 |
| 3.09719 | 6.75187 | 3.61 | 3.60274 | 7.72933 | 6.23577 | 9.9924 | 4.80411 |
| 6.72504 | 5.30324 | 5.99308 | 5.5401 | 3.02302 | 6.00111 | 3.93483 | 8.73686 |
| 9.41968 | 9.89529 | 7.8635 | 7.20996 | 8.77775 | 9.65851 | 9.31017 | 6.76951 |
| 8.67115 | 8.1863 | 10.2851 | 9.40309 | 7.73566 | 9.52232 | 8.22956 | 11.0917 |
| 5.24499 | 9.26529 | 4.77736 | 10.1575 | 9.19039 | 4.12462 | 10.7382 | 2.91813 |
| 9.57246 | 9.03488 | 10.5367 | 9.50563 | 9.07969 | 9.44639 | 7.18938 | 10.171 |
| 4.00801 | 5.22217 | 4.81159 | 6.17356 | 5.92423 | 4.14361 | 3.12902 | 5.85019 |
| 5.96311 | 5.18265 | 7.18858 | 4.0547 | 9.06867 | 6.81836 | 7.46415 | 8.99866 |
| 7.27828 | 6.84466 | 7.92249 | 3.72527 | 9.12594 | 8.19164 | 8.57642 | 8.96926 |
| 8.1637 | 5.04959 | 9.55798 | 4.25128 | 10.6684 | 8.39299 | 10.2076 | 10.4947 |
| 6.41341 | 5.5221 | 8.51952 | 4.85532 | 6.80841 | 8.11227 | 7.55348 | 8.76813 |
| 10.5059 | 10.2911 | 11.2435 | 10.0329 | 11.2594 | 11.4288 | 9.74168 | 8.34726 |
| 5.36762 | 5.38508 | 6.13533 | 7.33896 | 7.3879 | 5.49069 | 8.47194 | 6.95576 |
| 8.93999 | 8.02869 | 10.6267 | 3.62481 | 6.13966 | 8.24014 | 4.55229 | 6.24507 |
| 7.14214 | 7.95368 | 8.6493 | 3.46392 | 6.66597 | 8.13458 | 3.23607 | 4.35709 |
| 8.72927 | 8.15171 | 7.33079 | 8.10924 | 7.51155 | 7.28243 | 7.34954 | 7.03815 |
| 7.81346 | 7.46466 | 8.63351 | 9.38329 | 5.97736 | 7.44139 | 7.1882 | 5.91133 |
| 3.10617 | 3.39177 | 4.63322 | 3.61268 | 3.07001 | 3.35453 | 3.02771 | 3.3456 |
| 3.05487 | 3.10592 | 3.82316 | 3.20353 | 3.30847 | 2.8839 | 2.62086 | 2.86608 |
| 7.18565 | 4.69953 | 5.66976 | 9.31555 | 4.80417 | 6.86823 | 3.0085 | 2.79587 |
| 7.71194 | 2.46781 | 2.45172 | 2.73047 | 5.89478 | 2.72914 | 10.7063 | 6.94921 |
| 8.52369 | 10.6997 | 9.86347 | 9.36458 | 10.7834 | 11.0678 | 10.3022 | 11.4152 |
| 9.26868 | 8.67264 | 7.41626 | 8.48862 | 6.73463 | 9.11923 | 6.9509 | 8.97481 |
| 9.60118 | 10.9611 | 6.15145 | 9.29699 | 9.54636 | 9.38986 | 6.39353 | 4.76939 |
| 6.2291 | 6.71804 | 10.0208 | 6.39895 | 6.57842 | 6.11404 | 4.73175 | 6.94533 |
| 6.92142 | 5.40608 | 5.36905 | 7.75172 | 6.1443 | 7.38858 | 6.1014 | 3.64169 |
| 3.47795 | 3.36364 | 5.87853 | 3.37265 | 3.04799 | 3.52614 | 3.18693 | 8.06754 |
| 3.51112 | 4.12381 | 4.40998 | 3.3835 | 3.31238 | 3.2431 | 3.16277 | 6.29578 |
| 6.84251 | 6.04016 | 9.02297 | 4.2233 | 8.7638 | 6.64188 | 5.32568 | 9.07254 |
| 4.43465 | 3.67823 | 4.35306 | 2.60046 | 5.49729 | 4.84835 | 6.06142 | 6.25821 |
| 3.90412 | 3.05031 | 9.39357 | 7.05706 | 3.02046 | 2.92724 | 2.76926 | 7.17585 |
| 3.26632 | 8.50073 | 7.17979 | 6.24897 | 3.51515 | 10.8378 | 3.43139 | 3.10169 |
| 7.15669 | 6.22543 | 4.29403 | 8.70476 | 5.99162 | 5.22958 | 6.09767 | 4.66789 |
| 8.71992 | 7.75808 | 11.219 | 7.73912 | 5.16611 | 11.74 | 9.8076 | 8.6495 |
| 3.1813 | 3.56685 | 3.60745 | 2.8664 | 9.95644 | 3.1671 | 3.07918 | 2.543 |
| 10.1795 | 9.84323 | 9.6513 | 7.09893 | 10.1649 | 10.8708 | 10.0437 | 11.1875 |
| 5.38613 | 5.1599 | 4.45212 | 3.28118 | 3.6377 | 4.10939 | 6.23087 | 7.49079 |
| 3.2463 | 3.07245 | 3.53695 | 7.14882 | 3.10092 | 3.4465 | 7.27131 | 6.18735 |
| 7.58113 | 10.1366 | 9.75963 | 3.54713 | 10.9613 | 10.0014 | 9.06423 | 12.078 |
| 9.60144 | 10.1184 | 9.58653 | 9.67233 | 5.09288 | 3.13701 | 3.28252 | 9.08183 |
| 10.651 | 10.7312 | 12.238 | 10.4285 | 9.30691 | 11.1934 | 9.13794 | 11.543 |
| 10.294 | 8.76063 | 9.31135 | 7.62844 | 8.93148 | 7.47615 | 2.81802 | 9.38144 |
| 8.95587 | 8.60514 | 7.25781 | 8.25584 | 6.59615 | 5.67476 | 5.96711 | 3.34073 |
| 6.25491 | 2.89987 | 2.97891 | 3.58712 | 3.13973 | 7.0756 | 3.14666 | 4.57028 |
| 2.99542 | 3.1854 | 2.98556 | 2.98093 | 2.94315 | 3.16103 | 3.03431 | 2.79714 |
| 3.49638 | 3.4563 | 3.47408 | 4.1885 | 4.80891 | 6.32062 | 3.33992 | 4.54546 |
| 7.8818 | 7.18202 | 7.20962 | 7.85822 | 5.63484 | 7.11939 | 6.93838 | 4.63365 |
| 6.67476 | 6.22518 | 9.66234 | 3.07782 | 7.32536 | 8.17326 | 3.05735 | 7.69091 |
| 8.40453 | 8.40177 | 7.27845 | 10.0696 | 6.86176 | 8.30629 | 9.45359 | 6.76693 |
| 11.2003 | 9.7103 | 11.1394 | 11.5495 | 7.4246 | 8.61696 | 11.0743 | 12.4845 |
| 7.95364 | 8.33582 | 8.7786 | 8.16346 | 7.5011 | 9.32159 | 7.16811 | 7.18899 |

| | | | | | | | |
|---|---|---|---|---|---|---|---|
| 3.07402 | 3.50092 | 3.34666 | 3.17604 | 3.42069 | 3.96879 | 3.49809 | 3.72009 |
| 10.0573 | 5.85126 | 10.4444 | 8.28079 | 11.8095 | 10.0528 | 10.8609 | 12.1376 |
| 9.5179 | 8.75408 | 8.1352 | 9.54288 | 8.59821 | 7.8092 | 7.94889 | 8.83817 |
| 8.74677 | 7.77861 | 8.79176 | 9.24018 | 10.1488 | 8.40746 | 4.27973 | 4.49906 |
| 6.32999 | 5.6619 | 6.2239 | 5.30748 | 5.42502 | 9.49799 | 5.91153 | 10.5412 |
| 8.96974 | 7.91804 | 8.59533 | 3.86936 | 8.49377 | 8.75875 | 9.11472 | 9.31568 |
| 9.40803 | 9.27651 | 10.4167 | 8.53595 | 9.32011 | 9.31598 | 7.78995 | 9.31557 |
| 2.87299 | 8.2585 | 2.94417 | 2.7523 | 5.94383 | 5.30595 | 2.90515 | 2.7895 |
| 7.72232 | 6.49866 | 6.85316 | 4.86583 | 4.67186 | 7.78009 | 3.31843 | 5.96758 |
| 4.01063 | 4.96554 | 5.58146 | 3.02818 | 5.29875 | 5.97047 | 6.20753 | 4.84322 |
| 7.94539 | 6.2949 | 8.05201 | 6.52687 | 4.21693 | 3.85129 | 4.93152 | 3.92905 |
| 9.57114 | 7.01657 | 10.1293 | 6.94069 | 6.68218 | 4.27499 | 5.18082 | 10.4216 |
| 7.92881 | 6.54196 | 7.34673 | 6.99633 | 5.15897 | 6.10996 | 4.97728 | 4.33388 |
| 5.82293 | 6.13262 | 4.42904 | 3.16927 | 6.01411 | 5.00097 | 7.83646 | 7.69767 |
| 2.91959 | 6.2777 | 4.31645 | 6.60764 | 6.1125 | 6.36017 | 6.54136 | 6.67881 |
| 11.9614 | 12.6556 | 2.96805 | 12.7099 | 12.1597 | 11.6479 | 12.4842 | 3.03406 |
| 7.0364 | 5.68666 | 6.90935 | 3.25885 | 8.74446 | 7.11963 | 8.51507 | 8.4688 |
| 3.26236 | 3.11425 | 10.4585 | 3.14685 | 3.31762 | 2.98209 | 3.14295 | 3.24639 |
| 8.96974 | 6.26747 | 3.78576 | 6.35174 | 4.07986 | 3.69534 | 5.38053 | 3.94867 |
| 8.88445 | 7.46428 | 8.58926 | 5.13702 | 6.35648 | 7.61109 | 6.13516 | 7.44167 |
| 5.72351 | 3.31911 | 5.38359 | 5.97071 | 7.53563 | 5.36137 | 6.30563 | 7.58537 |
| 3.05088 | 4.48085 | 8.94578 | 9.04397 | 3.30595 | 5.4973 | 3.07661 | 2.66397 |
| 9.23218 | 9.37473 | 6.34816 | 8.89478 | 4.62878 | 8.30713 | 8.91659 | 3.59936 |
| 3.54853 | 3.7306 | 6.05693 | 3.4294 | 3.4466 | 4.39979 | 5.87139 | 9.18576 |
| 11.6797 | 9.94356 | 4.60172 | 9.99465 | 7.62871 | 9.23131 | 4.88541 | 7.17844 |
| 7.32828 | 3.44437 | 4.34319 | 3.41775 | 3.1101 | 3.47989 | 2.89561 | 3.10291 |
| 3.37212 | 3.49523 | 3.67551 | 3.33352 | 3.16388 | 3.32797 | 3.12852 | 3.2716 |
| 9.75991 | 10.4714 | 9.29328 | 9.86477 | 7.34996 | 9.52635 | 10.6288 | 8.4209 |
| 6.94763 | 4.22041 | 5.26654 | 3.05741 | 3.26716 | 5.11356 | 3.37446 | 5.8703 |
| 3.08794 | 3.01696 | 11.0226 | 2.96159 | 10.0455 | 10.5097 | 3.03143 | 8.83121 |
| 3.05717 | 3.21224 | 9.68495 | 2.97954 | 3.22899 | 3.417 | 3.22235 | 3.08123 |
| 5.93813 | 8.01834 | 5.12459 | 5.35767 | 6.23356 | 4.30198 | 4.46239 | 3.12726 |
| 3.33609 | 6.69926 | 6.23801 | 7.84528 | 6.63316 | 7.0598 | 7.38523 | 7.55405 |
| 8.77729 | 8.67345 | 4.99082 | 5.53301 | 6.1844 | 7.36213 | 3.92457 | 8.08712 |
| 3.57293 | 5.59933 | 3.25915 | 4.68925 | 4.14153 | 3.59959 | 3.24674 | 4.56523 |

| WM1552C | COLO-679 | LOXIMVI | A2058 | IGR-1 | WM-115 | SK-MEL-28 |
|---|---|---|---|---|---|---|
| 5.32692 | 6.66404 | 4.25444 | 6.47061 | 4.32008 | 5.95715 | 5.98648 |
| 3.91413 | 3.1748 | 5.97308 | 3.89749 | 2.89525 | 7.05778 | 2.8613 |
| 3.14963 | 3.24247 | 3.02236 | 3.33113 | 3.27994 | 6.42696 | 3.61993 |
| 5.81082 | 8.20114 | 7.00553 | 7.40789 | 6.3534 | 6.22494 | 7.06642 |
| 5.16107 | 5.71062 | 6.88452 | 6.91749 | 4.49654 | 8.05172 | 7.46952 |
| 5.26855 | 9.07006 | 3.96214 | 8.87543 | 7.0045 | 6.54175 | 7.72603 |
| 8.95914 | 7.80509 | 9.4685 | 6.46557 | 4.2859 | 8.70614 | 3.57867 |
| 4.07261 | 3.08352 | 3.14089 | 5.24361 | 11.9739 | 4.94924 | 3.37419 |
| 2.93662 | 3.25 | 2.90268 | 2.6908 | 3.3746 | 3.27733 | 3.98139 |
| 7.87377 | 2.8259 | 3.50721 | 2.56505 | 4.09483 | 7.45996 | 3.04805 |
| 8.44236 | 5.1485 | 5.23028 | 4.1562 | 8.54405 | 7.3156 | 4.22606 |
| 3.51742 | 4.49086 | 6.68081 | 4.58974 | 3.67282 | 6.15992 | 5.79503 |
| 5.16952 | 7.42745 | 5.30066 | 6.00894 | 7.88232 | 8.29699 | 8.13776 |
| 6.52973 | 7.7022 | 6.41715 | 6.66902 | 7.86734 | 6.88025 | 7.41456 |
| 4.8475 | 3.43117 | 8.79012 | 2.86895 | 3.09979 | 5.28345 | 4.19218 |
| 3.45114 | 3.23352 | 3.25816 | 3.3254 | 4.11477 | 3.67311 | 5.22104 |
| 10.4157 | 10.0856 | 11.6342 | 8.29962 | 4.62595 | 11.327 | 8.94786 |
| 3.31384 | 9.17233 | 3.20196 | 3.67402 | 5.42826 | 3.31797 | 9.25589 |
| 4.50659 | 3.28395 | 3.6628 | 3.68987 | 3.73389 | 3.67795 | 3.96067 |
| 3.16323 | 3.31264 | 3.1147 | 4.07559 | 4.70003 | 4.10686 | 2.99657 |
| 3.71087 | 5.29662 | 8.02005 | 8.0412 | 3.44588 | 8.29965 | 3.36622 |
| 11.2734 | 4.02157 | 9.81932 | 6.70288 | 7.51971 | 7.61152 | 3.29387 |
| 8.73446 | 4.0173 | 7.65507 | 7.20662 | 6.80509 | 8.93152 | 6.83398 |
| 7.1309 | 3.42917 | 3.0984 | 3.22761 | 7.64403 | 6.35823 | 3.50023 |
| 3.30098 | 3.84655 | 3.10126 | 3.55813 | 3.41742 | 3.23012 | 3.87151 |
| 7.75239 | 7.19616 | 6.53065 | 7.65247 | 7.21077 | 8.02497 | 6.78789 |
| 10.3874 | 8.8586 | 4.69453 | 8.44842 | 8.32433 | 8.43862 | 7.85611 |
| 2.78309 | 7.28094 | 2.82313 | 5.11976 | 3.04751 | 4.8038 | 8.02848 |
| 8.04311 | 7.21988 | 5.00213 | 7.12483 | 6.2852 | 5.94521 | 5.96585 |
| 8.7822 | 3.11159 | 5.71964 | 6.36554 | 3.23784 | 10.0703 | 3.42159 |
| 3.05337 | 10.4337 | 3.1436 | 3.37184 | 7.38845 | 3.26692 | 3.27426 |
| 3.72459 | 3.39597 | 3.13457 | 6.98899 | 4.8038 | 8.90139 | 9.96914 |
| 3.04933 | 3.06033 | 2.97443 | 2.90793 | 3.04432 | 3.10704 | 2.79626 |
| 7.39204 | 8.77681 | 3.90769 | 9.04162 | 8.88388 | 6.69265 | 9.14217 |
| 9.11313 | 9.17801 | 4.02985 | 8.59383 | 7.53827 | 8.08203 | 8.39414 |
| 6.59171 | 6.94062 | 7.23596 | 8.1023 | 8.56175 | 8.68684 | 6.09761 |
| 7.87344 | 7.39775 | 7.33229 | 8.30158 | 8.58072 | 8.88085 | 7.23161 |
| 3.10568 | 3.25703 | 3.05357 | 3.6051 | 3.21295 | 4.16588 | 3.81457 |
| 6.9635 | 5.27297 | 8.06046 | 5.15251 | 3.83963 | 5.24356 | 5.44125 |
| 9.17168 | 8.97025 | 3.2529 | 8.54369 | 2.99614 | 3.10493 | 7.41327 |
| 3.32873 | 8.88442 | 3.06449 | 3.27378 | 2.97807 | 6.01173 | 3.1906 |
| 9.28657 | 3.37971 | 9.68675 | 2.95732 | 4.96506 | 9.42928 | 3.5486 |
| 7.49749 | 7.86079 | 6.96401 | 8.43104 | 8.0096 | 7.95525 | 6.66357 |
| 2.91286 | 5.0535 | 3.74347 | 4.30355 | 2.95933 | 2.65866 | 2.90479 |
| 2.78558 | 7.74729 | 9.15874 | 9.41637 | 6.73018 | 6.17001 | 10.6 |
| 7.61529 | 2.74058 | 5.95277 | 2.74416 | 2.71236 | 7.36717 | 2.89704 |
| 8.58442 | 3.22801 | 10.28 | 4.68483 | 3.45464 | 7.91766 | 2.80581 |
| 3.01114 | 4.88454 | 3.67313 | 6.87683 | 7.3102 | 3.13882 | 7.22614 |
| 11.4686 | 3.57503 | 11.5124 | 3.16072 | 3.69049 | 10.8293 | 5.15956 |

| | | | | | | |
|---|---|---|---|---|---|---|
| 4.13916 | 3.55985 | 3.15697 | 4.47035 | 3.59202 | 5.52631 | 5.14155 |
| 3.98945 | 6.96293 | 4.89946 | 5.41683 | 10.1461 | 6.75825 | 10.5529 |
| 11.4341 | 9.55771 | 8.20203 | 6.22392 | 5.93273 | 6.51721 | 11.7035 |
| 7.74343 | 8.50387 | 8.2229 | 6.78913 | 9.17878 | 7.16706 | 7.78415 |
| 2.89584 | 3.22178 | 3.23809 | 2.85172 | 3.52825 | 3.49965 | 3.19696 |
| 4.84209 | 5.75384 | 6.73429 | 3.94777 | 4.53492 | 5.86374 | 5.08753 |
| 8.51241 | 8.30291 | 4.96369 | 7.76034 | 8.7785 | 6.70572 | 8.03812 |
| 2.75823 | 7.30975 | 2.83364 | 4.56032 | 6.76836 | 6.24914 | 8.37638 |
| 4.91211 | 5.22998 | 4.69673 | 6.21615 | 5.4486 | 5.10285 | 4.79137 |
| 6.50533 | 6.48543 | 4.64178 | 5.23144 | 6.77391 | 4.79883 | 6.56135 |
| 5.41291 | 6.02386 | 5.04955 | 3.88371 | 3.72464 | 3.43877 | 7.7153 |
| 6.54463 | 5.74711 | 3.66338 | 3.05273 | 3.06119 | 9.82947 | 4.63027 |
| 7.41288 | 5.76795 | 5.60403 | 6.69313 | 8.36166 | 7.12901 | 8.60893 |
| 2.9626 | 7.05756 | 3.10578 | 6.72956 | 3.24571 | 7.60391 | 3.09664 |
| 3.23169 | 2.84215 | 2.70514 | 2.77543 | 2.6892 | 2.97506 | 2.89075 |
| 6.65624 | 3.86042 | 8.07816 | 3.5303 | 6.71745 | 8.40893 | 3.35625 |
| 6.56136 | 6.37553 | 5.93437 | 6.72051 | 5.58222 | 5.8364 | 6.22201 |
| 6.69524 | 7.66711 | 6.53716 | 7.57291 | 9.77466 | 6.34097 | 8.6136 |
| 7.23844 | 5.13269 | 6.80226 | 4.27726 | 3.45038 | 6.84761 | 3.47939 |
| 10.1659 | 4.87899 | 2.97581 | 4.05057 | 12.1744 | 3.64513 | 12.6146 |
| 3.33665 | 10.8856 | 3.26456 | 9.86177 | 12.2981 | 4.78812 | 10.8465 |
| 7.44116 | 7.25378 | 6.62541 | 8.29943 | 7.16842 | 8.1254 | 7.32167 |
| 5.54523 | 5.10235 | 4.11024 | 6.93656 | 5.77225 | 5.21233 | 6.18377 |
| 2.7656 | 7.68215 | 3.34468 | 9.24069 | 3.15159 | 7.98064 | 9.78536 |
| 3.77917 | 4.4352 | 3.61253 | 3.90843 | 4.53243 | 4.44205 | 3.81803 |
| 3.21296 | 3.42735 | 3.3397 | 3.30081 | 3.0805 | 9.88642 | 5.59236 |
| 8.9576 | 9.59116 | 4.57299 | 7.78598 | 4.97167 | 9.78523 | 8.96035 |
| 3.13946 | 3.57086 | 2.80985 | 3.74176 | 5.06106 | 2.90634 | 3.45236 |
| 9.2013 | 2.64884 | 10.5522 | 3.93031 | 3.14888 | 10.8362 | 3.13285 |
| 2.90497 | 6.21976 | 3.45059 | 6.83688 | 9.2261 | 4.30945 | 9.72508 |
| 7.58789 | 8.10612 | 3.55654 | 7.44995 | 6.67396 | 5.99732 | 6.50418 |
| 6.81899 | 7.17076 | 3.80763 | 8.89989 | 8.60509 | 6.49702 | 6.74484 |
| 8.20706 | 3.74568 | 7.86169 | 9.24879 | 4.15121 | 6.4422 | 5.93444 |
| 8.97558 | 6.30001 | 5.92709 | 7.50716 | 7.834 | 7.72782 | 7.00583 |
| 9.45705 | 9.12309 | 5.57851 | 8.59016 | 8.69213 | 6.51977 | 8.30594 |
| 9.94793 | 3.33223 | 3.03566 | 3.86627 | 2.71278 | 10.1319 | 2.64039 |
| 10.5541 | 3.98437 | 3.8153 | 4.76753 | 3.16582 | 10.5489 | 3.46199 |
| 3.20029 | 3.59164 | 3.49112 | 3.764 | 3.79207 | 4.05809 | 3.63405 |
| 7.66625 | 7.33863 | 4.9056 | 6.78534 | 7.48907 | 6.71586 | 6.78984 |
| 9.73209 | 7.80037 | 4.90117 | 8.82073 | 7.70063 | 5.70127 | 5.22938 |
| 9.98055 | 8.99023 | 8.21417 | 8.6501 | 8.34645 | 7.79906 | 8.42612 |
| 3.12051 | 5.82058 | 3.64801 | 5.94518 | 5.69766 | 4.31704 | 7.18889 |
| 7.33697 | 7.7218 | 3.60206 | 8.21158 | 3.45589 | 4.01629 | 8.23122 |
| 4.68697 | 5.82853 | 3.7461 | 3.89796 | 6.40071 | 8.24694 | 8.76577 |
| 2.8228 | 3.98544 | 2.80055 | 2.55405 | 2.58963 | 2.7894 | 2.91109 |
| 2.95597 | 4.65462 | 3.02344 | 3.80315 | 3.43338 | 4.01521 | 2.84328 |
| 4.82571 | 3.53217 | 5.09452 | 6.00111 | 3.38955 | 6.63909 | 4.65843 |
| 3.15276 | 3.52553 | 2.92396 | 3.17776 | 2.83588 | 2.93838 | 3.52913 |
| 12.1513 | 2.90473 | 2.97506 | 2.83417 | 2.95162 | 12.6229 | 2.91948 |
| 7.67414 | 2.97879 | 3.20962 | 3.20852 | 3.02406 | 6.92005 | 3.36581 |
| 11.4763 | 3.47257 | 4.81876 | 3.44565 | 4.41226 | 9.07486 | 3.83659 |
| 10.5741 | 3.0925 | 7.89011 | 2.89887 | 6.15051 | 3.9603 | 2.82045 |
| 2.96723 | 3.28023 | 2.96636 | 2.93146 | 3.05015 | 3.15545 | 3.0595 |

| | | | | | | |
|---|---|---|---|---|---|---|
| 5.30688 | 3.03611 | 10.8519 | 2.81335 | 2.73543 | 3.62351 | 2.98882 |
| 11.6816 | 3.6739 | 3.61918 | 4.61051 | 3.66243 | 11.904 | 3.82835 |
| 7.81622 | 7.74547 | 3.40532 | 7.3947 | 4.51999 | 8.96773 | 7.45579 |
| 3.19072 | 4.20209 | 3.36986 | 3.57317 | 3.01691 | 4.61806 | 3.42986 |
| 2.44871 | 5.17704 | 2.82195 | 3.92182 | 3.13261 | 6.53595 | 6.77767 |
| 3.13244 | 8.79408 | 2.82099 | 7.22437 | 7.12952 | 4.39565 | 9.99915 |
| 4.64894 | 6.85098 | 3.25867 | 3.53598 | 7.20784 | 5.09293 | 6.95446 |
| 3.17606 | 3.03503 | 5.85168 | 2.72704 | 2.6892 | 3.53931 | 2.88262 |
| 3.5355 | 3.1723 | 3.50808 | 3.28058 | 3.43338 | 3.28629 | 3.33518 |
| 3.78621 | 6.64615 | 3.80101 | 4.47136 | 10.1504 | 9.52744 | 4.12971 |
| 5.62462 | 7.95652 | 7.36824 | 8.28395 | 7.53519 | 7.54606 | 9.23167 |
| 8.35377 | 7.83609 | 9.41075 | 8.42753 | 8.18322 | 7.58811 | 7.84298 |
| 4.18122 | 5.12448 | 4.01272 | 8.67362 | 4.48688 | 5.21654 | 7.13092 |
| 2.99662 | 2.99672 | 8.28901 | 5.60032 | 3.10638 | 3.04106 | 3.08216 |
| 3.47565 | 3.25249 | 3.48338 | 3.49115 | 6.47713 | 3.06772 | 3.61455 |
| 10.2021 | 8.4115 | 10.2753 | 5.82151 | 5.65059 | 10.3263 | 8.62703 |
| 10.2005 | 10.6908 | 8.61069 | 10.2649 | 10.613 | 3.53098 | 11.9203 |
| 3.37045 | 3.1968 | 3.2237 | 3.30674 | 3.35668 | 6.24828 | 4.4676 |
| 6.53055 | 4.07885 | 11.5778 | 7.53702 | 7.32336 | 11.0239 | 7.05734 |
| 3.32753 | 3.30535 | 2.9116 | 6.09717 | 5.19477 | 7.89228 | 3.05442 |
| 4.64852 | 8.21467 | 3.93494 | 8.27684 | 7.44047 | 7.49588 | 9.24999 |
| 11.443 | 8.77956 | 11.4644 | 5.25122 | 7.96855 | 11.7348 | 5.5643 |
| 7.11106 | 8.32577 | 2.9956 | 8.07865 | 6.24178 | 6.15667 | 8.08934 |
| 2.9748 | 12.3541 | 3.12659 | 12.8852 | 13.153 | 3.08561 | 11.8311 |
| 3.86817 | 3.21015 | 9.08873 | 2.90918 | 3.11683 | 3.07856 | 3.32836 |
| 9.23852 | 7.5182 | 7.9329 | 8.92766 | 7.67749 | 7.70263 | 7.59176 |
| 9.35147 | 9.50369 | 5.72204 | 8.79846 | 9.0807 | 7.72132 | 8.61625 |
| 3.79045 | 3.28441 | 4.33677 | 3.3899 | 3.66461 | 4.60416 | 3.66044 |
| 8.53147 | 5.89987 | 8.83797 | 7.13446 | 6.6541 | 9.59377 | 4.59906 |
| 7.04038 | 6.96285 | 6.02313 | 7.79032 | 7.78826 | 6.08888 | 6.64704 |
| 3.79551 | 4.3248 | 6.28746 | 4.28442 | 4.91048 | 4.40939 | 4.08341 |
| 9.43854 | 8.91682 | 4.89616 | 8.55273 | 8.36353 | 7.51083 | 8.30303 |
| 3.70189 | 10.0802 | 7.38602 | 7.726 | 6.78447 | 4.93822 | 7.39856 |
| 3.13522 | 3.07084 | 3.0108 | 2.93953 | 3.0937 | 3.14727 | 3.16987 |
| 2.71928 | 6.33827 | 8.86938 | 3.53739 | 9.80882 | 7.98394 | 4.00897 |
| 9.30751 | 3.84341 | 10.0695 | 6.53667 | 3.73257 | 9.38943 | 3.91356 |
| 6.18984 | 5.59852 | 6.26889 | 6.41286 | 7.51473 | 8.10437 | 6.26147 |
| 7.28648 | 6.41217 | 5.42969 | 6.99539 | 6.32805 | 4.99419 | 5.96796 |
| 7.82019 | 9.49343 | 3.46162 | 8.95716 | 8.45418 | 5.63996 | 8.07171 |
| 9.59504 | 7.81963 | 9.15784 | 6.9 | 7.07411 | 9.42093 | 7.03022 |
| 3.15005 | 3.07598 | 3.45956 | 3.20973 | 3.28868 | 3.01368 | 3.34674 |
| 7.5055 | 7.52932 | 5.65748 | 7.14813 | 7.3811 | 6.69417 | 7.56546 |
| 3.34157 | 6.03192 | 6.43102 | 3.13425 | 4.26389 | 3.08034 | 3.54291 |
| 11.2533 | 3.50856 | 3.22806 | 3.29163 | 5.88705 | 9.96282 | 3.27093 |
| 2.88179 | 10.1017 | 5.84183 | 9.71026 | 8.7897 | 8.88586 | 8.58961 |
| 7.45452 | 10.3614 | 3.23922 | 7.57548 | 3.83645 | 7.93261 | 4.14406 |
| 3.51144 | 5.63185 | 3.20722 | 7.16097 | 7.18342 | 4.87061 | 7.87804 |
| 2.91258 | 5.35049 | 2.7646 | 5.70144 | 4.20407 | 3.70213 | 5.99576 |
| 7.66911 | 3.26084 | 8.43549 | 3.01558 | 3.19384 | 8.47084 | 3.27767 |
| 7.17772 | 7.8179 | 3.00792 | 5.71706 | 3.99365 | 7.52585 | 4.6304 |
| 9.27499 | 9.75871 | 9.63927 | 9.92296 | 10.0004 | 9.70298 | 9.56565 |
| 8.21756 | 5.6704 | 3.27998 | 7.669 | 3.1282 | 3.324 | 6.17221 |
| 7.25061 | 3.53172 | 3.02416 | 4.12514 | 8.03877 | 9.88272 | 5.06733 |

| | | | | | | |
|---|---|---|---|---|---|---|
| 5.98557 | 6.36886 | 4.56137 | 7.3558 | 5.27066 | 5.39085 | 5.72635 |
| 9.73185 | 9.35143 | 3.01781 | 3.38258 | 7.77177 | 9.92513 | 8.83223 |
| 3.26204 | 7.35695 | 2.83506 | 8.24106 | 6.68063 | 4.75323 | 4.66941 |
| 3.9353 | 6.91293 | 3.17204 | 3.56216 | 3.41946 | 4.08823 | 4.5871 |
| 4.08031 | 8.85601 | 3.15322 | 9.62274 | 9.02085 | 7.96364 | 8.84291 |
| 8.10404 | 8.22788 | 8.63805 | 8.02029 | 9.06604 | 7.30138 | 7.16719 |
| 2.69074 | 4.89492 | 2.81699 | 9.40296 | 9.1403 | 8.45947 | 10.4924 |
| 9.98578 | 9.0799 | 8.41389 | 9.92443 | 8.11112 | 7.7364 | 9.54323 |
| 6.34536 | 10.7299 | 9.26564 | 10.1325 | 9.28526 | 8.45715 | 6.23567 |
| 3.29256 | 4.84361 | 5.64047 | 5.97485 | 6.33806 | 7.029 | 3.69775 |
| 7.19331 | 5.60831 | 9.22821 | 3.24413 | 6.70755 | 6.50254 | 3.37419 |
| 3.85618 | 7.61349 | 3.22989 | 6.6692 | 6.16616 | 8.4038 | 4.7402 |
| 9.6599 | 9.75843 | 8.33497 | 7.34365 | 5.8632 | 10.986 | 3.1615 |
| 4.94283 | 5.64283 | 3.10408 | 8.21923 | 7.47763 | 5.94641 | 7.97952 |
| 3.3753 | 4.11393 | 3.82253 | 5.75249 | 4.56273 | 5.71121 | 8.23901 |
| 3.02285 | 3.3394 | 5.22587 | 3.55389 | 3.14628 | 3.29995 | 4.25675 |
| 3.22909 | 4.95724 | 2.73228 | 5.47303 | 4.24305 | 4.26561 | 6.17217 |
| 3.72839 | 7.11873 | 3.02763 | 7.05887 | 6.27088 | 6.49645 | 8.35065 |
| 3.17872 | 5.10086 | 3.39157 | 3.57177 | 4.79505 | 3.25346 | 4.64593 |
| 4.00946 | 7.95902 | 3.21959 | 4.05877 | 9.50058 | 7.46622 | 8.24666 |
| 2.86617 | 5.45991 | 2.54086 | 6.62628 | 3.10574 | 8.17964 | 3.56882 |
| 7.0676 | 6.82496 | 8.28279 | 9.42252 | 3.40694 | 7.99595 | 5.10182 |
| 7.97959 | 4.13861 | 7.41905 | 5.9525 | 8.93655 | 7.99725 | 4.90599 |
| 3.12041 | 3.22601 | 2.87203 | 4.69849 | 3.01786 | 7.45617 | 3.13878 |
| 8.28692 | 5.12183 | 6.85657 | 3.92541 | 4.34751 | 10.3387 | 5.07382 |
| 9.30164 | 4.34783 | 3.02787 | 3.07259 | 5.68467 | 7.48502 | 3.42722 |
| 2.90651 | 9.5751 | 2.70573 | 7.48578 | 6.33592 | 10.9369 | 10.2013 |
| 2.83487 | 4.40717 | 5.09085 | 6.19582 | 5.77996 | 4.82498 | 6.98784 |
| 8.27024 | 5.8046 | 8.99426 | 6.36517 | 5.34271 | 7.95063 | 4.69795 |
| 3.30418 | 3.04453 | 2.65874 | 2.72334 | 3.1462 | 3.23618 | 3.11043 |
| 2.89987 | 5.79483 | 2.75944 | 2.74627 | 7.55326 | 2.5021 | 9.61887 |
| 3.9611 | 7.23732 | 3.63356 | 9.3008 | 6.72243 | 8.82787 | 7.47083 |
| 8.84057 | 9.76392 | 3.15813 | 9.30431 | 9.35138 | 11.1961 | 8.77926 |
| 7.40281 | 2.64281 | 4.60451 | 4.66649 | 4.21521 | 5.72259 | 4.57288 |
| 5.29285 | 5.4601 | 6.05853 | 6.91085 | 3.79008 | 5.22201 | 4.79553 |
| 9.6565 | 12.7031 | 12.0237 | 11.4871 | 5.34342 | 8.47724 | 9.94622 |
| 5.10986 | 7.77285 | 4.612 | 6.89225 | 2.79645 | 6.06525 | 6.52783 |
| 5.65222 | 6.63271 | 3.40076 | 5.625 | 5.22642 | 5.66662 | 6.44506 |
| 8.99233 | 4.73751 | 8.89754 | 5.4441 | 6.75506 | 7.40054 | 3.83629 |
| 9.42952 | 8.04786 | 4.86451 | 8.92476 | 7.39846 | 10.6499 | 7.34053 |
| 3.70389 | 7.05541 | 2.59213 | 6.25289 | 4.05846 | 7.87471 | 7.52637 |
| 8.47688 | 9.7675 | 8.72607 | 9.5516 | 9.6543 | 10.0434 | 9.20929 |
| 3.02179 | 3.61595 | 2.43354 | 2.63437 | 2.29032 | 4.96868 | 4.37279 |
| 4.93363 | 3.3548 | 9.48705 | 3.02056 | 2.92026 | 8.67614 | 8.96111 |
| 3.18905 | 11.1029 | 3.03088 | 9.04259 | 8.36831 | 6.66097 | 10.429 |
| 9.52932 | 12.1557 | 4.92128 | 11.9455 | 10.7035 | 11.3173 | 11.8263 |
| 3.46287 | 4.31915 | 3.37153 | 3.62829 | 6.91992 | 8.09392 | 5.2062 |
| 3.4095 | 8.24263 | 3.51448 | 9.83096 | 11.8561 | 8.2636 | 10.9897 |
| 3.91832 | 5.09012 | 3.73897 | 4.9653 | 6.09629 | 5.36812 | 4.57386 |
| 6.41933 | 3.25836 | 3.72722 | 7.07554 | 6.28589 | 3.27251 | 3.62542 |
| 3.08295 | 3.08061 | 3.14744 | 2.98705 | 2.80899 | 2.90744 | 2.84964 |
| 8.36582 | 7.50133 | 7.31264 | 9.41314 | 8.57377 | 9.47855 | 9.12796 |
| 11.564 | 9.23776 | 11.5949 | 7.57694 | 7.45807 | 13.0336 | 9.76955 |

| | | | | | | |
|---|---|---|---|---|---|---|
| 6.3365 | 4.6723 | 2.96987 | 5.36277 | 3.36738 | 7.30841 | 8.17062 |
| 4.43355 | 5.99931 | 3.75287 | 4.97617 | 3.71969 | 7.88072 | 6.35306 |
| 3.61407 | 8.57094 | 5.21705 | 8.73583 | 3.88789 | 12.2384 | 11.4746 |
| 3.31407 | 8.50822 | 3.12585 | 8.32199 | 3.12441 | 9.56362 | 3.51714 |
| 3.27058 | 3.68549 | 3.20865 | 3.22222 | 3.45941 | 3.12901 | 3.3566 |
| 3.4164 | 6.15797 | 3.31628 | 3.22993 | 2.95248 | 3.39781 | 3.88631 |
| 3.12667 | 4.65525 | 2.99564 | 3.01932 | 3.05154 | 12.079 | 7.08903 |
| 4.11677 | 4.21322 | 2.98399 | 3.33593 | 3.03476 | 10.725 | 4.58488 |
| 3.10137 | 3.05513 | 2.81264 | 3.00996 | 3.13349 | 10.3855 | 7.66663 |
| 3.1128 | 3.08274 | 3.11841 | 3.22986 | 3.2384 | 3.46127 | 3.30795 |
| 7.77457 | 8.02069 | 4.63779 | 7.55452 | 7.93195 | 5.04801 | 7.85689 |
| 5.8847 | 7.20406 | 5.30919 | 5.6872 | 4.12358 | 3.66027 | 2.8495 |
| 3.21779 | 4.73245 | 2.79496 | 6.1822 | 3.92267 | 4.9579 | 4.40616 |
| 4.90056 | 5.70115 | 4.06425 | 5.14863 | 4.56186 | 5.02864 | 4.83333 |
| 3.66693 | 6.2568 | 3.60176 | 6.83815 | 3.91713 | 6.23576 | 7.09829 |
| 11.4613 | 9.83918 | 11.2895 | 11.6726 | 10.6148 | 11.1779 | 11.3812 |
| 3.98887 | 9.99281 | 4.13972 | 6.77745 | 8.89909 | 8.84114 | 9.81141 |
| 5.68215 | 7.7187 | 3.57182 | 10.4794 | 5.07166 | 11.2409 | 11.2874 |
| 7.34268 | 7.88808 | 4.21182 | 5.16297 | 3.77291 | 9.08238 | 8.15402 |
| 9.85007 | 10.6836 | 8.80472 | 6.61956 | 8.23508 | 8.6975 | 8.79967 |
| 11.1002 | 3.59217 | 11.0143 | 6.87079 | 3.51661 | 7.78202 | 5.26848 |
| 12.2375 | 7.19817 | 10.0193 | 9.81855 | 5.87037 | 9.77401 | 4.84018 |
| 6.35779 | 6.91398 | 8.14719 | 6.92922 | 10.0208 | 6.45722 | 6.03658 |
| 9.37337 | 3.57101 | 4.95967 | 5.62375 | 3.57185 | 8.83258 | 5.54126 |
| 2.88362 | 3.3354 | 11.8591 | 2.99657 | 2.85016 | 7.87634 | 3.31215 |
| 9.33676 | 11.2795 | 9.81271 | 3.13552 | 2.90958 | 4.68358 | 3.22897 |
| 4.16809 | 3.21136 | 9.94209 | 3.33065 | 3.50052 | 8.75196 | 3.49294 |
| 10.4871 | 3.39323 | 8.86829 | 3.78441 | 3.38006 | 7.58304 | 3.19183 |
| 2.81386 | 3.63299 | 3.91032 | 3.11299 | 5.65316 | 3.27165 | 5.356 |
| 7.86628 | 4.7869 | 6.77479 | 3.51344 | 5.85851 | 7.03591 | 5.80369 |
| 5.57242 | 9.17854 | 7.93061 | 9.61869 | 8.64396 | 6.63228 | 8.07464 |
| 5.45734 | 3.97596 | 4.3342 | 4.27146 | 3.26196 | 8.33402 | 3.19273 |
| 7.16771 | 3.03515 | 2.95946 | 3.27494 | 3.03639 | 5.02875 | 3.45536 |
| 6.73021 | 6.84779 | 9.83319 | 8.68209 | 8.5751 | 10.4107 | 5.75246 |
| 4.67233 | 7.88314 | 4.17245 | 6.92347 | 6.48195 | 7.16012 | 7.73996 |
| 5.65655 | 6.23476 | 2.59009 | 7.12299 | 7.00004 | 9.05522 | 8.26017 |
| 3.02796 | 3.09598 | 3.26685 | 3.322 | 3.99134 | 7.03167 | 7.76706 |
| 3.0744 | 2.84636 | 3.06368 | 3.01159 | 2.96051 | 5.17298 | 3.96808 |
| 4.88995 | 4.21704 | 9.25867 | 6.09357 | 6.65213 | 6.36536 | 4.01679 |
| 6.26802 | 7.10515 | 3.88085 | 7.36869 | 3.7832 | 4.97803 | 5.85907 |
| 4.49511 | 9.23599 | 5.13298 | 8.90484 | 7.84375 | 8.61648 | 8.29396 |
| 3.22736 | 3.02712 | 2.87323 | 7.23011 | 2.85742 | 7.75336 | 3.10905 |
| 6.47295 | 6.732 | 3.67389 | 6.52177 | 6.86381 | 5.80683 | 4.8732 |
| 7.60025 | 6.60654 | 5.76338 | 6.18908 | 6.54118 | 5.487 | 5.84057 |
| 2.93435 | 3.12255 | 2.81703 | 2.96272 | 3.62106 | 2.97184 | 3.72832 |
| 8.3692 | 8.13631 | 7.79434 | 8.39547 | 8.54877 | 8.10014 | 7.33172 |
| 3.36247 | 7.36929 | 3.28293 | 5.73714 | 4.31201 | 5.99394 | 7.24473 |
| 9.7947 | 3.0153 | 3.04916 | 3.57212 | 10.0875 | 3.3312 | 3.23732 |
| 3.8055 | 6.14808 | 3.76869 | 7.22406 | 6.44651 | 3.1684 | 7.77857 |
| 4.46058 | 4.29667 | 3.62514 | 7.00541 | 6.02723 | 5.04167 | 6.07628 |
| 3.08898 | 2.84886 | 2.69959 | 2.69807 | 2.6892 | 2.5769 | 3.02373 |
| 3.33539 | 3.92714 | 3.61718 | 3.60884 | 5.36208 | 6.2 | 3.66491 |
| 3.59635 | 3.58851 | 3.24364 | 3.62563 | 3.38871 | 6.7702 | 3.76864 |

| | | | | | | |
|---|---|---|---|---|---|---|
| 4.03556 | 4.36681 | 3.56421 | 3.45085 | 3.25672 | 10.5378 | 5.32403 |
| 10.1571 | 13.2978 | 12.6908 | 12.5039 | 5.92068 | 9.73897 | 10.5472 |
| 3.30466 | 4.04589 | 3.3266 | 3.15077 | 3.39246 | 11.465 | 4.1992 |
| 3.52684 | 3.3865 | 3.22873 | 3.57713 | 3.39687 | 10.6328 | 4.16857 |
| 3.62962 | 4.58682 | 3.10462 | 4.6767 | 8.92741 | 3.70752 | 6.88439 |
| 2.77829 | 7.41278 | 3.1737 | 6.12675 | 6.2423 | 4.67741 | 6.40711 |
| 10.6365 | 7.98631 | 2.95963 | 8.64188 | 10.2064 | 10.0965 | 10.2776 |
| 4.95322 | 8.13261 | 3.07162 | 3.12696 | 3.2823 | 7.25058 | 3.39761 |
| 4.80732 | 6.38289 | 3.86795 | 3.15498 | 3.30934 | 3.94035 | 3.23133 |
| 9.38778 | 6.76596 | 8.04177 | 5.90951 | 6.4108 | 9.90339 | 9.61166 |
| 10.4178 | 7.84885 | 2.65533 | 7.36833 | 9.22846 | 10.141 | 7.70151 |
| 6.64577 | 4.45314 | 9.29865 | 7.04108 | 6.32835 | 7.21126 | 7.20121 |
| 3.13428 | 9.20716 | 3.02263 | 6.23238 | 7.14498 | 4.50146 | 6.38875 |
| 8.17218 | 9.06184 | 9.36424 | 8.93882 | 9.52718 | 6.78279 | 7.67445 |
| 11.5986 | 11.3289 | 11.1222 | 11.4888 | 11.2562 | 11.0095 | 11.7075 |
| 3.45591 | 3.4354 | 3.24038 | 4.22215 | 4.47827 | 3.22864 | 7.37761 |
| 3.3223 | 2.89123 | 10.3369 | 8.1826 | 3.07754 | 2.89803 | 3.03691 |
| 3.20886 | 3.79681 | 3.01737 | 4.16633 | 3.35228 | 3.02117 | 3.77325 |
| 3.49223 | 5.99567 | 3.38375 | 3.49469 | 9.43717 | 5.48557 | 3.41028 |
| 3.71246 | 6.20479 | 6.37253 | 6.18075 | 4.37904 | 5.69663 | 7.76571 |
| 6.87615 | 2.9826 | 2.66121 | 7.04896 | 6.72186 | 5.96733 | 6.06449 |
| 9.30069 | 4.901 | 8.73472 | 9.58392 | 4.81799 | 8.05463 | 4.7281 |
| 4.66034 | 5.86876 | 6.24851 | 4.89025 | 5.62839 | 6.71499 | 4.2196 |
| 3.07756 | 4.46244 | 2.72915 | 3.39737 | 2.75926 | 2.92539 | 3.08091 |
| 5.4133 | 3.94634 | 6.84273 | 8.01686 | 3.97051 | 3.20544 | 3.66544 |
| 3.32514 | 3.72477 | 3.53504 | 8.28939 | 9.15497 | 4.07505 | 7.40815 |
| 3.78951 | 5.05712 | 3.1697 | 6.43687 | 3.92387 | 8.59458 | 4.71812 |
| 12.1614 | 10.1296 | 3.28312 | 11.5512 | 12.2568 | 10.6894 | 11.9902 |
| 3.34552 | 8.4137 | 3.41681 | 7.93885 | 6.03014 | 8.41349 | 10.8184 |
| 9.95353 | 5.68355 | 10.7232 | 7.09875 | 4.91392 | 8.58101 | 4.00955 |
| 2.83946 | 2.69888 | 2.76115 | 2.8601 | 2.61081 | 2.95936 | 2.83609 |
| 7.69295 | 8.69139 | 4.342 | 8.14822 | 7.42349 | 4.56796 | 8.33863 |
| 3.42511 | 12.2382 | 3.69987 | 10.8469 | 13.2189 | 3.51796 | 13.2454 |
| 9.06737 | 5.95645 | 11.0756 | 7.78827 | 10.0986 | 8.29561 | 9.43258 |
| 7.7653 | 12.7244 | 7.51414 | 11.6179 | 2.96099 | 11.0165 | 5.31692 |
| 3.11722 | 4.99547 | 3.20582 | 5.97978 | 7.38332 | 5.97998 | 8.02614 |
| 2.65253 | 6.72544 | 2.80296 | 6.35652 | 3.32606 | 2.72793 | 4.39918 |
| 4.81704 | 7.04016 | 3.97855 | 5.15535 | 4.86243 | 7.27635 | 7.21106 |
| 9.91122 | 3.40467 | 8.55056 | 7.5173 | 3.53783 | 10.8249 | 4.35067 |
| 8.02742 | 8.17951 | 7.57668 | 7.27027 | 7.01222 | 7.45988 | 6.18346 |
| 3.82915 | 9.1355 | 5.13093 | 9.3675 | 9.39473 | 5.70503 | 7.11853 |
| 3.50637 | 3.3125 | 3.4511 | 3.32552 | 3.6266 | 3.09295 | 3.28012 |
| 5.14636 | 3.72512 | 8.86568 | 8.08845 | 3.73939 | 8.23395 | 3.20643 |
| 11.4656 | 7.99731 | 9.25862 | 4.33796 | 7.08406 | 10.5576 | 9.02458 |
| 2.70758 | 9.1441 | 3.45489 | 2.88863 | 6.72422 | 5.08838 | 2.82748 |
| 4.93972 | 7.64633 | 3.82253 | 7.02097 | 4.54746 | 3.30018 | 9.06893 |
| 5.23515 | 4.93847 | 3.40965 | 8.6964 | 3.46476 | 7.5282 | 3.68637 |
| 7.99566 | 7.63756 | 6.26425 | 7.4432 | 7.17449 | 5.5791 | 6.92166 |
| 6.87124 | 4.63817 | 3.58247 | 5.3873 | 5.29433 | 3.66561 | 5.02718 |
| 8.48215 | 6.31085 | 7.12331 | 3.34048 | 5.8379 | 8.28347 | 7.6079 |
| 3.44886 | 8.89192 | 9.23048 | 5.08898 | 4.58683 | 9.93055 | 8.91993 |
| 3.09042 | 4.07987 | 5.96967 | 7.29557 | 3.09979 | 2.65059 | 3.66772 |
| 4.00817 | 5.71758 | 5.52462 | 6.97218 | 5.31556 | 6.71316 | 7.21843 |

| | | | | | | |
|---|---|---|---|---|---|---|
| 6.46585 | 6.40713 | 3.51304 | 5.99914 | 6.14054 | 5.73413 | 4.69795 |
| 2.75528 | 2.99986 | 2.94907 | 3.02299 | 4.40558 | 7.3481 | 3.16984 |
| 3.6204 | 3.18159 | 3.5327 | 3.94405 | 8.25479 | 4.73086 | 6.28236 |
| 3.0014 | 4.11764 | 2.71884 | 6.47418 | 3.94335 | 7.93974 | 7.1981 |
| 7.75807 | 7.39075 | 10.0573 | 5.45899 | 9.06714 | 8.54077 | 8.55293 |
| 3.29007 | 3.79471 | 3.12882 | 3.50937 | 3.452 | 6.78675 | 4.4693 |
| 8.18955 | 7.64906 | 4.27114 | 8.3263 | 8.56621 | 4.64419 | 6.8216 |
| 8.34348 | 7.41151 | 4.41014 | 7.16612 | 7.09101 | 5.66801 | 6.8728 |
| 4.92311 | 4.02011 | 5.54983 | 10.5845 | 3.8971 | 8.08914 | 8.98147 |
| 3.77254 | 3.38666 | 8.40554 | 3.47378 | 4.5946 | 4.4357 | 6.09851 |
| 10.5774 | 3.62179 | 3.10101 | 3.13188 | 4.17263 | 7.36846 | 3.09834 |
| 3.25542 | 6.71139 | 3.4078 | 5.61059 | 4.92325 | 7.04217 | 4.15019 |
| 7.43077 | 12.4108 | 7.53289 | 10.5499 | 13.0004 | 11.4178 | 12.6658 |
| 6.25204 | 6.07529 | 3.98104 | 6.69728 | 3.15194 | 5.75356 | 2.95401 |
| 9.42114 | 4.13205 | 10.6267 | 4.24789 | 3.9039 | 11.04 | 3.78522 |
| 2.80259 | 5.71794 | 2.97366 | 2.95413 | 3.588 | 3.50206 | 3.26485 |
| 4.09417 | 4.05121 | 4.69885 | 3.70918 | 3.25802 | 7.30866 | 4.69942 |
| 3.64159 | 2.94241 | 7.22612 | 4.57352 | 2.83229 | 8.66441 | 5.37753 |
| 3.15392 | 3.00724 | 7.83851 | 3.37555 | 2.77677 | 7.93131 | 3.62847 |
| 2.7392 | 2.97921 | 2.88321 | 2.96359 | 3.08798 | 7.77697 | 2.86427 |
| 7.75528 | 6.97844 | 9.54318 | 6.53373 | 7.63941 | 9.14108 | 6.80526 |
| 6.25151 | 8.81792 | 3.20821 | 11.1356 | 9.51505 | 9.41257 | 11.5882 |
| 9.43434 | 8.66153 | 8.34494 | 8.78485 | 8.58568 | 9.46664 | 8.76267 |
| 3.87866 | 3.55422 | 2.83598 | 5.22666 | 5.00159 | 2.84328 | 4.7414 |
| 9.35824 | 8.94711 | 9.70991 | 8.5808 | 8.16383 | 9.34298 | 4.91704 |
| 2.86378 | 7.8553 | 3.37849 | 7.23169 | 6.25146 | 3.81642 | 8.4653 |
| 5.23574 | 9.90002 | 4.77465 | 10.7074 | 10.5671 | 7.63033 | 11.3507 |
| 2.93751 | 7.53214 | 3.02366 | 6.57094 | 9.12421 | 3.09095 | 11.9939 |
| 8.27624 | 3.41137 | 7.8179 | 3.96773 | 3.24656 | 7.75088 | 3.66072 |
| 6.3954 | 8.82679 | 6.17004 | 6.71925 | 6.82181 | 7.5713 | 8.07706 |
| 6.7377 | 8.19865 | 3.37736 | 6.43829 | 6.28359 | 5.44869 | 8.77068 |
| 9.37893 | 8.76252 | 9.54599 | 9.91856 | 9.78498 | 8.57336 | 8.69107 |
| 3.24388 | 6.45651 | 3.01313 | 4.27386 | 3.60023 | 3.43752 | 4.00028 |
| 6.94819 | 6.03363 | 4.10145 | 5.74496 | 5.86879 | 4.88319 | 5.34442 |
| 2.79339 | 10.6495 | 2.77021 | 3.97017 | 2.9483 | 2.69614 | 2.82432 |
| 7.61486 | 7.23745 | 7.96331 | 7.85142 | 7.50911 | 7.93557 | 7.34629 |
| 3.372 | 11.4434 | 3.56393 | 10.842 | 10.4215 | 10.9542 | 10.961 |
| 8.00069 | 8.31592 | 4.23167 | 6.80455 | 5.70266 | 8.45402 | 6.56863 |
| 3.19299 | 6.02375 | 2.57293 | 4.71682 | 7.67928 | 5.78043 | 3.18921 |
| 7.38915 | 8.59758 | 2.34853 | 7.04219 | 7.67722 | 4.75163 | 8.50982 |
| 8.44975 | 4.34799 | 9.97317 | 3.57896 | 3.22436 | 9.5206 | 2.88699 |
| 7.4207 | 7.49585 | 7.0271 | 7.88305 | 7.62528 | 8.15768 | 7.68553 |
| 3.66794 | 3.92063 | 3.57905 | 3.8568 | 3.56331 | 3.43027 | 8.21885 |
| 3.31501 | 8.56462 | 3.8282 | 3.45875 | 3.65307 | 3.8529 | 3.96574 |
| 2.86498 | 3.09567 | 2.79153 | 8.60029 | 9.92713 | 2.84724 | 9.85082 |
| 8.12465 | 3.97608 | 9.12454 | 3.75546 | 3.57503 | 7.23114 | 4.32944 |
| 4.33502 | 3.63162 | 4.35427 | 4.07879 | 4.1559 | 5.43092 | 4.76553 |
| 4.90217 | 7.59969 | 5.7771 | 6.49118 | 7.61494 | 5.65144 | 6.63689 |
| 3.89563 | 4.08469 | 4.08766 | 4.10306 | 3.99576 | 4.82347 | 3.57252 |
| 8.98914 | 7.27486 | 8.13523 | 7.39447 | 4.84192 | 10.3494 | 8.92564 |
| 2.94592 | 4.93877 | 2.98083 | 6.16165 | 3.01377 | 3.27015 | 7.14955 |
| 7.4264 | 8.17953 | 7.75705 | 7.51105 | 7.98764 | 7.84416 | 7.92708 |
| 9.13324 | 3.32606 | 8.94948 | 4.00321 | 8.22691 | 7.06696 | 3.87292 |

| | | | | | | |
|---|---|---|---|---|---|---|
| 11.3917 | 3.17093 | 6.70203 | 4.87394 | 3.07331 | 10.0866 | 3.81581 |
| 3.23973 | 3.14899 | 3.70904 | 3.48966 | 3.14991 | 6.2618 | 3.12985 |
| 5.87984 | 3.27654 | 9.62621 | 2.80831 | 3.34329 | 9.19648 | 2.99674 |
| 3.05178 | 6.94575 | 2.72547 | 5.586 | 3.25889 | 10.0677 | 5.41891 |
| 5.31341 | 4.89963 | 2.82099 | 10.204 | 3.32232 | 2.9018 | 10.7651 |
| 5.48559 | 9.27936 | 6.20233 | 8.64283 | 7.76677 | 6.83479 | 9.03002 |
| 4.53633 | 3.68578 | 3.82887 | 6.01718 | 3.68162 | 3.43713 | 4.877 |
| 3.69158 | 7.58837 | 3.3736 | 8.81739 | 8.77814 | 5.06963 | 9.66089 |
| 8.64553 | 6.23487 | 8.86979 | 7.68038 | 9.0319 | 10.2489 | 4.54904 |
| 3.05957 | 4.37258 | 2.89313 | 3.63497 | 3.79759 | 3.48442 | 3.25187 |
| 7.82676 | 7.33646 | 5.16687 | 7.14909 | 7.60743 | 5.63621 | 6.71833 |
| 3.01114 | 6.1121 | 6.00375 | 3.17377 | 3.40911 | 7.36698 | 3.21356 |
| 3.43608 | 3.24536 | 2.95309 | 3.79374 | 3.94079 | 3.29922 | 3.35043 |
| 7.50699 | 6.39426 | 8.04677 | 6.94312 | 6.82727 | 7.33122 | 7.52328 |
| 7.72029 | 7.71646 | 7.28968 | 8.72169 | 7.5635 | 7.7888 | 7.18355 |
| 3.16307 | 6.13947 | 2.89661 | 3.02711 | 2.91933 | 3.04619 | 3.05627 |
| 9.46177 | 7.40121 | 9.43668 | 7.67759 | 8.58862 | 10.3108 | 4.54492 |
| 9.11528 | 7.28976 | 2.75371 | 2.91476 | 9.01782 | 2.95948 | 8.36797 |
| 10.3153 | 9.08583 | 7.90084 | 7.22708 | 11.0967 | 8.938 | 5.87453 |
| 7.24622 | 6.59749 | 6.75489 | 6.03482 | 6.57915 | 7.3926 | 6.72221 |
| 6.78406 | 7.63026 | 3.00497 | 10.4531 | 8.73449 | 8.09109 | 9.56089 |
| 3.17535 | 7.71465 | 3.35354 | 5.21683 | 2.73494 | 7.02502 | 7.25268 |
| 3.05983 | 3.09476 | 2.89499 | 7.4047 | 3.22382 | 2.77148 | 3.15843 |
| 5.56219 | 8.31851 | 5.67219 | 3.8007 | 10.0621 | 3.56703 | 5.86723 |
| 3.38546 | 9.31622 | 3.40076 | 9.11845 | 9.34818 | 6.41242 | 10.4186 |
| 7.30893 | 6.99901 | 5.84897 | 6.23921 | 4.75531 | 7.42013 | 4.93754 |
| 3.74669 | 3.54524 | 3.53504 | 11.269 | 11.701 | 3.33019 | 10.9478 |
| 3.57291 | 10.1112 | 3.40076 | 10.55 | 10.0695 | 7.43952 | 10.3997 |
| 5.57933 | 7.39494 | 6.45008 | 7.72922 | 8.77235 | 3.73305 | 8.76863 |
| 3.19228 | 2.87999 | 2.85454 | 3.27102 | 2.78639 | 3.01773 | 3.04335 |
| 2.95076 | 11.7102 | 2.92008 | 9.02693 | 8.75745 | 10.7324 | 10.9177 |
| 3.16323 | 3.21361 | 3.81559 | 3.14974 | 3.21272 | 3.12257 | 3.06445 |
| 3.16773 | 3.05048 | 4.40715 | 2.97183 | 2.81977 | 2.81212 | 2.80435 |
| 6.66085 | 7.5258 | 7.4218 | 8.89421 | 8.53284 | 9.84675 | 10.3381 |
| 4.57831 | 6.63125 | 6.94961 | 3.31398 | 3.14566 | 7.40572 | 5.4974 |
| 10.7565 | 3.15678 | 9.84902 | 3.31967 | 3.28676 | 9.08515 | 2.77331 |
| 2.77353 | 9.854 | 2.70513 | 2.75031 | 6.8107 | 3.42845 | 8.00218 |
| 3.15448 | 5.71867 | 3.02123 | 3.66533 | 3.22158 | 6.95018 | 3.8505 |
| 9.34862 | 4.86667 | 9.30132 | 8.25595 | 4.79164 | 9.78278 | 6.62376 |
| 5.45205 | 3.46131 | 2.38894 | 5.91543 | 3.48213 | 5.15035 | 5.59498 |
| 5.48079 | 4.83754 | 3.77233 | 6.03883 | 5.88129 | 6.57376 | 4.17559 |
| 3.16502 | 8.22409 | 3.44973 | 9.95464 | 3.52187 | 5.23141 | 4.43431 |
| 7.39955 | 3.42325 | 9.7125 | 3.03882 | 2.72756 | 9.62555 | 3.42438 |
| 10.9361 | 2.80163 | 10.493 | 3.02306 | 3.43403 | 11.5894 | 2.72343 |
| 4.55989 | 9.61656 | 3.20827 | 6.73016 | 6.04804 | 8.27726 | 9.04568 |
| 10.3577 | 6.7923 | 2.87075 | 8.11919 | 4.00254 | 11.0851 | 11.0763 |
| 2.8798 | 5.55266 | 2.94616 | 5.99989 | 6.1075 | 5.80178 | 6.07406 |
| 6.07648 | 6.77478 | 3.62514 | 6.02323 | 6.5331 | 5.26974 | 6.47468 |
| 3.65664 | 4.52039 | 7.18359 | 6.48677 | 5.64223 | 5.62178 | 6.21891 |
| 3.00236 | 3.98512 | 2.89548 | 3.8457 | 3.36171 | 5.10867 | 5.522 |
| 2.79464 | 4.11605 | 4.2172 | 6.78987 | 7.53961 | 2.89508 | 3.32752 |
| 3.60208 | 10.6319 | 3.31529 | 4.26752 | 5.40814 | 3.02753 | 8.23253 |
| 8.95296 | 3.00772 | 3.116 | 2.78811 | 2.79432 | 10.7523 | 2.79498 |

| | | | | | | |
|---|---|---|---|---|---|---|
| 8.74792 | 6.1574 | 5.37204 | 6.36558 | 7.13205 | 3.59418 | 3.80724 |
| 9.07427 | 7.41596 | 3.13432 | 3.39325 | 4.45205 | 7.01545 | 6.91339 |
| 9.03274 | 6.78531 | 3.02926 | 7.12665 | 9.11925 | 8.83773 | 8.97285 |
| 3.8856 | 3.28177 | 3.25101 | 5.4512 | 4.5944 | 5.1968 | 6.39624 |
| 4.96741 | 7.19581 | 3.27989 | 9.03499 | 9.60858 | 6.98295 | 9.66376 |
| 6.58685 | 7.91165 | 6.90517 | 9.39058 | 7.2993 | 7.66167 | 8.05685 |
| 3.20595 | 10.1006 | 3.33433 | 7.43413 | 10.2719 | 3.08226 | 9.55338 |
| 8.09617 | 8.45381 | 8.32864 | 8.93954 | 7.82212 | 9.56069 | 7.52812 |
| 3.26704 | 4.86683 | 2.98662 | 4.88305 | 3.35317 | 4.50466 | 3.91286 |
| 6.86979 | 8.1567 | 7.00263 | 8.25949 | 7.75468 | 7.70033 | 6.82315 |
| 8.10648 | 8.70658 | 7.99739 | 8.94986 | 9.08338 | 7.00759 | 7.28401 |
| 9.69666 | 9.72396 | 8.03679 | 9.16763 | 8.99924 | 9.50249 | 9.68007 |
| 7.78869 | 6.53003 | 7.35151 | 8.06473 | 8.49135 | 7.49252 | 6.88148 |
| 5.81382 | 9.43255 | 3.16562 | 10.3179 | 9.20283 | 9.31992 | 10.1568 |
| 6.76113 | 7.53046 | 6.53064 | 6.53745 | 6.7113 | 6.06981 | 7.53449 |
| 3.77908 | 3.58672 | 3.35199 | 3.95389 | 6.15887 | 9.77704 | 4.42803 |
| 3.03577 | 5.60336 | 2.94302 | 8.31978 | 5.40262 | 5.1422 | 5.75467 |
| 3.1057 | 7.56056 | 3.39532 | 7.47266 | 7.79628 | 5.54156 | 7.90781 |
| 3.04638 | 8.34361 | 3.01067 | 8.27414 | 8.11399 | 6.13192 | 9.44425 |
| 9.28647 | 3.27953 | 3.96928 | 3.02402 | 7.30422 | 8.05737 | 3.30624 |
| 11.7223 | 2.88393 | 11.9204 | 3.07533 | 3.15584 | 3.26332 | 2.90644 |
| 2.86514 | 3.88605 | 2.71094 | 4.08479 | 6.51985 | 2.72837 | 4.08957 |
| 7.087 | 2.59744 | 6.60826 | 2.79339 | 2.70252 | 8.21582 | 7.47438 |
| 2.76567 | 11.2991 | 9.9712 | 10.9888 | 9.25214 | 9.71625 | 9.28178 |
| 5.60776 | 7.49577 | 6.74777 | 5.95038 | 5.03597 | 8.19861 | 7.59686 |
| 2.91254 | 8.70201 | 2.94775 | 8.04134 | 7.11774 | 6.48991 | 8.0676 |
| 4.08938 | 3.32746 | 3.33864 | 4.83977 | 6.85932 | 8.25136 | 7.99651 |
| 3.78794 | 3.57632 | 3.27174 | 6.57702 | 3.98925 | 5.92537 | 5.92664 |
| 9.45153 | 3.24833 | 8.66036 | 3.81771 | 5.77589 | 7.81396 | 3.30328 |
| 8.7975 | 2.9505 | 7.48744 | 3.70944 | 4.92035 | 3.9346 | 2.92599 |
| 8.22585 | 3.82195 | 3.4536 | 5.82126 | 6.80882 | 8.54952 | 4.5619 |
| 3.93175 | 7.22697 | 3.11244 | 4.23157 | 2.942 | 5.41124 | 6.69094 |
| 3.25652 | 2.8659 | 3.13573 | 6.66121 | 3.28741 | 3.1218 | 3.16075 |
| 3.41153 | 6.99757 | 3.37675 | 3.89655 | 8.48571 | 3.27061 | 4.25972 |
| 3.118 | 6.27726 | 3.4454 | 5.12394 | 6.65032 | 4.77236 | 8.32366 |
| 3.11775 | 3.89123 | 3.14914 | 6.94179 | 5.81245 | 3.41368 | 3.12421 |
| 3.02792 | 2.88177 | 2.93126 | 2.612 | 2.66154 | 2.66389 | 3.04496 |
| 8.65059 | 5.61522 | 4.18483 | 5.34107 | 5.07214 | 10.5087 | 9.83682 |
| 10.2342 | 5.43683 | 10.0212 | 7.45318 | 6.849 | 10.3493 | 6.80201 |
| 6.61921 | 2.80678 | 11.7116 | 9.53473 | 4.25821 | 7.76538 | 4.60299 |
| 10.8995 | 5.51614 | 9.62401 | 3.39237 | 10.8375 | 12.6656 | 3.58892 |
| 4.52322 | 10.1031 | 4.81889 | 11.0932 | 2.86102 | 3.38356 | 2.79775 |
| 12.1892 | 11.1717 | 2.90126 | 9.56283 | 9.33175 | 12.112 | 10.3557 |
| 4.68127 | 3.62785 | 11.024 | 8.47608 | 9.86153 | 8.78911 | 9.54342 |
| 4.28649 | 7.64989 | 4.87587 | 4.85591 | 6.84301 | 6.81211 | 8.70839 |
| 2.67459 | 4.29948 | 3.5363 | 3.55522 | 3.02938 | 7.84727 | 6.65965 |
| 8.47171 | 2.94825 | 9.77329 | 2.92161 | 3.06439 | 7.12343 | 2.87901 |
| 6.2027 | 3.67561 | 4.30904 | 3.72491 | 5.27469 | 7.7143 | 4.31534 |
| 4.39958 | 5.44958 | 3.33944 | 6.2234 | 5.46013 | 4.07406 | 5.85514 |
| 3.09533 | 6.98163 | 2.89953 | 2.93694 | 3.05698 | 9.86252 | 3.0529 |
| 3.59445 | 9.14158 | 3.13213 | 7.84479 | 8.87416 | 4.04216 | 9.3942 |
| 11.8069 | 10.7575 | 10.9353 | 11.0629 | 11.5642 | 11.7837 | 11.6178 |
| 4.8647 | 5.01044 | 3.29277 | 9.19431 | 7.75541 | 7.05944 | 6.9165 |

| | | | | | | |
|---|---|---|---|---|---|---|
| 9.76613 | 3.26192 | 7.80652 | 3.40106 | 3.58958 | 3.60935 | 3.23124 |
| 11.4585 | 10.9015 | 8.5132 | 11.3621 | 10.7318 | 10.7205 | 10.1544 |
| 4.2586 | 8.24694 | 2.7359 | 9.96225 | 9.50047 | 3.9549 | 10.3068 |
| 5.28962 | 10.2105 | 6.59621 | 8.10133 | 5.2406 | 5.30825 | 8.50224 |
| 10.8366 | 5.03993 | 10.1934 | 8.38576 | 5.10161 | 8.68019 | 6.32294 |
| 9.12588 | 8.82318 | 7.44226 | 9.61638 | 8.429 | 8.93573 | 7.93529 |
| 3.53278 | 9.42027 | 3.45626 | 9.43533 | 9.69842 | 8.50285 | 8.83577 |
| 3.05243 | 2.99434 | 2.71264 | 2.96805 | 3.88977 | 4.50281 | 2.96236 |
| 3.27511 | 5.02173 | 4.52161 | 3.91096 | 4.67709 | 6.70437 | 3.85467 |
| 7.39151 | 6.25784 | 4.91205 | 6.35321 | 5.81318 | 6.44805 | 6.14075 |
| 3.33627 | 8.84687 | 2.90219 | 4.33318 | 5.63591 | 8.74068 | 3.52983 |
| 7.10338 | 7.75717 | 3.7296 | 7.21269 | 7.23841 | 8.40582 | 7.48311 |
| 4.06341 | 6.02763 | 4.37728 | 5.71991 | 5.43722 | 4.96294 | 4.83157 |
| 5.6128 | 4.39963 | 7.55278 | 4.69789 | 3.39378 | 9.07225 | 3.75572 |
| 5.6808 | 7.33375 | 7.36757 | 7.42458 | 9.12379 | 6.43478 | 6.52454 |
| 2.75847 | 12.7609 | 2.93471 | 12.5994 | 12.0786 | 2.84989 | 12.3804 |
| 8.52208 | 7.25219 | 5.72076 | 8.29962 | 9.06984 | 7.65737 | 7.70835 |
| 2.91197 | 3.08366 | 3.5141 | 3.16072 | 3.95654 | 3.32558 | 2.9241 |
| 3.68613 | 7.82997 | 3.12202 | 4.06231 | 6.84213 | 3.84091 | 8.56542 |
| 3.55732 | 6.14301 | 4.22404 | 7.22336 | 3.72896 | 4.69737 | 3.87717 |
| 6.94075 | 6.45848 | 6.2971 | 6.7741 | 5.58154 | 5.69541 | 4.62564 |
| 3.19713 | 8.22481 | 3.29527 | 3.28899 | 7.69818 | 3.23972 | 9.54418 |
| 3.30566 | 4.57064 | 3.53769 | 5.83755 | 5.38766 | 4.83933 | 11.1234 |
| 10.3683 | 4.22827 | 9.29478 | 6.76728 | 3.95114 | 9.90551 | 4.39258 |
| 5.02349 | 10.4357 | 10.5704 | 6.88103 | 7.17018 | 10.3382 | 7.86947 |
| 4.674 | 3.49946 | 3.518 | 3.13754 | 3.3119 | 3.60585 | 3.4296 |
| 6.3902 | 3.6077 | 10.8203 | 3.74586 | 7.34056 | 3.32828 | 3.06245 |
| 8.32176 | 9.79637 | 8.80836 | 9.66439 | 3.84586 | 9.40936 | 10.539 |
| 3.09767 | 3.50056 | 3.53223 | 3.07715 | 3.22997 | 3.32566 | 3.10444 |
| 11.2448 | 3.29924 | 2.91882 | 5.61478 | 10.9054 | 5.60894 | 3.228 |
| 3.03331 | 10.0204 | 2.83506 | 2.94906 | 2.87355 | 2.7864 | 3.20999 |
| 4.32501 | 3.67962 | 4.37689 | 3.12733 | 3.00242 | 7.8581 | 4.70358 |
| 7.56786 | 8.14649 | 7.41738 | 7.97247 | 8.05054 | 5.79971 | 6.63589 |
| 9.38173 | 5.6803 | 2.99541 | 7.06497 | 6.44422 | 9.37369 | 7.0726 |
| 3.79183 | 6.14385 | 3.46162 | 3.44916 | 5.69203 | 4.07836 | 5.87472 |

**Table S7. Gene expression regulated by TAZ.**

List of genes with altered expression value in A375 cells overexpressing WWTR1 (TAZ). Genes with 1.5-fold expression value changes are listed.

| Gene | log2FoldChange | PValue | FDR |
|---|---|---|---|
| OLR1 | 8.295749723 | 5.61E-99 | 4.62E-95 |
| KRT17 | 7.999507512 | 3.12E-50 | 1.84E-47 |
| NPPB | 7.883741309 | 5.15E-66 | 9.43E-63 |
| TNNT2 | 7.202164658 | 3.94E-89 | 2.16E-85 |
| MFAP5 | 6.446544772 | 9.19E-57 | 7.97E-54 |
| KPRP | 6.274498442 | 3.16E-31 | 5.10E-29 |
| CDH5 | 6.243188881 | 1.36E-23 | 1.14E-21 |
| SDPR | 6.16365841 | 6.66E-12 | 1.55E-10 |
| ALPP | 5.987943101 | 1.69E-53 | 1.16E-50 |
| APCS | 5.549864485 | 6.88E-35 | 1.45E-32 |
| PSG4 | 5.356464469 | 5.16E-48 | 2.83E-45 |
| ANO2 | 5.253792612 | 4.48E-34 | 8.89E-32 |
| NPR3 | 5.173511465 | 4.99E-11 | 1.05E-09 |
| ANXA3 | 5.105238112 | 6.23E-27 | 7.22E-25 |
| CCL3 | 5.059252073 | 1.59E-38 | 4.29E-36 |
| KCND3 | 4.928089584 | 1.55E-65 | 2.56E-62 |
| XIRP1 | 4.905662414 | 4.52E-09 | 7.22E-08 |
| TNNC1 | 4.640529737 | 8.09E-18 | 3.79E-16 |
| KIF17 | 4.564688149 | 8.60E-45 | 3.94E-42 |
| ANKRD1 | 4.55405431 | 1.01E-33 | 1.93E-31 |
| SERPINE1 | 4.547500596 | 3.02E-39 | 8.44E-37 |
| DYSF | 4.529680031 | 9.46E-44 | 3.80E-41 |
| TLL1 | 4.488050946 | 1.43E-34 | 2.92E-32 |
| HCLS1 | 4.449774922 | 1.07E-25 | 1.07E-23 |
| KRTAP2-3 | 4.436129834 | 4.76E-21 | 3.16E-19 |
| KISS1 | 4.368497247 | 1.53E-31 | 2.52E-29 |
| ANKRD2 | 4.27348164 | 1.03E-26 | 1.17E-24 |
| RP11-54A9.1 | 4.264510616 | 2.48E-19 | 1.37E-17 |
| RP11-380J14 | 4.239688085 | 2.30E-26 | 2.53E-24 |
| IL18 | 4.221584142 | 1.28E-12 | 3.26E-11 |
| VSTM1 | 4.219731838 | 7.75E-33 | 1.33E-30 |
| SFTA1P | 4.209688786 | 4.71E-24 | 4.08E-22 |
| CCDC148 | 4.143676735 | 9.37E-59 | 9.08E-56 |
| MOB3B | 4.127966293 | 7.60E-38 | 1.96E-35 |
| MUC2 | 4.055689153 | 1.11E-06 | 1.16E-05 |
| KRT34 | 4.03331082 | 4.50E-25 | 4.26E-23 |
| LINC00520 | 3.994091812 | 3.66E-20 | 2.20E-18 |
| CTD-2354A18 | 3.933871346 | 1.27E-25 | 1.27E-23 |
| FOLR1 | 3.82720568 | 4.28E-15 | 1.49E-13 |
| EDN1 | 3.793610814 | 6.43E-23 | 5.00E-21 |
| UNC13D | 3.722885886 | 1.13E-40 | 3.71E-38 |
| ITGB2 | 3.719145896 | 5.04E-31 | 7.90E-29 |
| ECSCR | 3.710431237 | 4.71E-23 | 3.70E-21 |
| TOX | 3.635796179 | 2.10E-39 | 6.08E-37 |
| PVRL4 | 3.600519091 | 9.30E-34 | 1.80E-31 |
| TGFB2 | 3.591517856 | 1.30E-25 | 1.29E-23 |
| LRRC4C | 3.575160298 | 2.51E-29 | 3.51E-27 |
| STXBP6 | 3.531389065 | 2.28E-14 | 7.35E-13 |
| WNT7A | 3.52063926 | 5.92E-33 | 1.04E-30 |
| LYPD6B | 3.506193617 | 4.01E-10 | 7.43E-09 |

| | | | |
|---|---|---|---|
| CXCL14 | 3.502745849 | 0.00011164 | 0.000717 |
| ADAMTS5 | 3.473753507 | 1.21E-07 | 1.51E-06 |
| TMEM40 | 3.459036671 | 8.75E-23 | 6.73E-21 |
| IL6 | 3.446263483 | 3.44E-07 | 3.99E-06 |
| GNGT2 | 3.420920932 | 8.17E-22 | 5.93E-20 |
| CLSTN2 | 3.413351328 | 3.01E-19 | 1.63E-17 |
| MPP7 | 3.398785379 | 2.09E-12 | 5.23E-11 |
| GBX2 | 3.33837745 | 1.73E-37 | 4.26E-35 |
| PEAR1 | 3.285467675 | 1.01E-29 | 1.45E-27 |
| GDF15 | 3.277974661 | 8.06E-11 | 1.64E-09 |
| IGDCC4 | 3.262405125 | 1.00E-27 | 1.25E-25 |
| RP3-399L15.3 | 3.260168163 | 4.14E-41 | 1.42E-38 |
| NLRP10 | 3.240493136 | 4.16E-13 | 1.13E-11 |
| PGM5 | 3.210399918 | 3.13E-09 | 5.10E-08 |
| ALS2CL | 3.194350049 | 2.91E-56 | 2.39E-53 |
| CDH4 | 3.165918645 | 1.80E-13 | 5.20E-12 |
| SYTL3 | 3.158298312 | 5.60E-21 | 3.62E-19 |
| CDA | 3.155533234 | 3.33E-27 | 3.98E-25 |
| PPME1 | 3.088891995 | 5.02E-83 | 2.07E-79 |
| TAGLN | 3.07171079 | 2.34E-17 | 1.04E-15 |
| MIR503HG | 3.035816426 | 3.51E-26 | 3.76E-24 |
| GFRA1 | 3.0315276 | 7.15E-15 | 2.44E-13 |
| SFN | 3.016695296 | 9.38E-20 | 5.36E-18 |
| SAA1 | 2.962517175 | 9.82E-05 | 0.00064159 |
| ADAMTS6 | 2.961519996 | 3.31E-24 | 2.93E-22 |
| FAM155B | 2.938829865 | 3.30E-23 | 2.64E-21 |
| CTC-308K20. | 2.936473775 | 4.82E-41 | 1.62E-38 |
| LCP1 | 2.906207571 | 3.87E-21 | 2.58E-19 |
| MME | 2.868266589 | 6.64E-14 | 2.05E-12 |
| ZNF542 | 2.867926638 | 9.03E-51 | 5.51E-48 |
| SBSN | 2.859032821 | 1.45E-09 | 2.49E-08 |
| RAB3B | 2.855026363 | 4.23E-37 | 1.03E-34 |
| 4-Mar | 2.833736473 | 4.92E-18 | 2.38E-16 |
| GOLT1A | 2.818027998 | 1.26E-21 | 8.99E-20 |
| MIR137HG | 2.814876107 | 1.45E-32 | 2.44E-30 |
| CALB1 | 2.809417134 | 4.76E-26 | 4.99E-24 |
| ADM | 2.801038008 | 2.25E-42 | 8.23E-40 |
| CD274 | 2.76702629 | 2.44E-24 | 2.19E-22 |
| UCA1 | 2.729457454 | 1.18E-11 | 2.69E-10 |
| TMEFF2 | 2.706522495 | 0.00011321 | 0.00072482 |
| APLN | 2.675495467 | 1.66E-14 | 5.41E-13 |
| ABI3 | 2.670343366 | 1.03E-16 | 4.37E-15 |
| FGF5 | 2.664332447 | 1.39E-30 | 2.10E-28 |
| F3 | 2.658192289 | 2.16E-67 | 5.09E-64 |
| TMEM178B | 2.643431486 | 2.42E-13 | 6.86E-12 |
| GAL | 2.624368385 | 1.20E-24 | 1.10E-22 |
| KITLG | 2.610832893 | 4.26E-17 | 1.85E-15 |
| TSPAN2 | 2.598004099 | 1.25E-26 | 1.41E-24 |
| GABRA5 | 2.595991903 | 1.20E-20 | 7.50E-19 |
| PLEKHA7 | 2.581110604 | 1.42E-23 | 1.19E-21 |
| RNF144B | 2.560764295 | 2.02E-33 | 3.71E-31 |
| MISP | 2.550432418 | 4.81E-18 | 2.34E-16 |
| DUSP8 | 2.546822774 | 8.92E-09 | 1.36E-07 |
| TRIM58 | 2.51651337 | 3.13E-39 | 8.60E-37 |
| PIK3AP1 | 2.499963187 | 1.67E-43 | 6.56E-41 |
| CGN | 2.49925825 | 1.28E-15 | 4.77E-14 |

| | | | |
|---|---|---|---|
| OSCAR | 2.480662883 | 2.20E-07 | 2.66E-06 |
| SEMA3E | 2.454563604 | 8.25E-08 | 1.07E-06 |
| ZNF185 | 2.445142482 | 1.06E-37 | 2.64E-35 |
| PCLO | 2.445087483 | 2.44E-63 | 3.35E-60 |
| PTPRR | 2.431227647 | 7.36E-19 | 3.79E-17 |
| AC073130.1 | 2.427627739 | 2.45E-14 | 7.87E-13 |
| DMKN | 2.426208326 | 7.16E-12 | 1.66E-10 |
| DCLK1 | 2.419658314 | 7.97E-20 | 4.62E-18 |
| TMEM45A | 2.418440717 | 2.13E-35 | 4.67E-33 |
| AOX1 | 2.417598845 | 4.49E-22 | 3.29E-20 |
| HSPB8 | 2.409790926 | 8.56E-30 | 1.26E-27 |
| TNFSF4 | 2.404877345 | 4.45E-07 | 5.04E-06 |
| INPP5D | 2.396608462 | 5.24E-51 | 3.32E-48 |
| OR2W3 | 2.388281999 | 5.20E-33 | 9.21E-31 |
| TIE1 | 2.377111098 | 2.12E-24 | 1.91E-22 |
| RP11-244F12 | 2.376675383 | 3.29E-15 | 1.16E-13 |
| IL2RG | 2.373958336 | 3.71E-05 | 0.00027196 |
| WWC1 | 2.352972947 | 1.44E-17 | 6.52E-16 |
| RASGRF1 | 2.345864223 | 1.20E-12 | 3.09E-11 |
| UPK3B | 2.334883363 | 9.25E-07 | 9.83E-06 |
| NRG1 | 2.330952538 | 2.93E-16 | 1.18E-14 |
| OR51B5 | 2.322759467 | 1.14E-15 | 4.29E-14 |
| THSD1 | 2.316896931 | 3.16E-19 | 1.70E-17 |
| CPED1 | 2.304026957 | 1.60E-23 | 1.32E-21 |
| NUAK2 | 2.279924628 | 2.43E-39 | 6.91E-37 |
| AP1M2 | 2.269804017 | 2.57E-19 | 1.41E-17 |
| RGS7 | 2.258057608 | 2.19E-28 | 2.82E-26 |
| SH2D5 | 2.255799535 | 7.13E-27 | 8.21E-25 |
| C12orf39 | 2.253373251 | 0.00015285 | 0.00093884 |
| CITED2 | 2.245659758 | 1.32E-07 | 1.64E-06 |
| PRR5L | 2.245211213 | 5.42E-20 | 3.19E-18 |
| PLEKHA6 | 2.229394198 | 7.73E-18 | 3.64E-16 |
| ARHGDIB | 2.185853975 | 9.43E-21 | 6.02E-19 |
| NID2 | 2.175647013 | 3.25E-06 | 3.08E-05 |
| RPL13AP20 | 2.164062725 | 7.20E-18 | 3.41E-16 |
| PTPRB | 2.161328111 | 7.76E-16 | 2.96E-14 |
| PAQR5 | 2.160661775 | 1.95E-13 | 5.62E-12 |
| PLCB4 | 2.145277672 | 2.24E-19 | 1.24E-17 |
| FSTL1 | 2.144394917 | 2.71E-26 | 2.96E-24 |
| MAGEC1 | 2.136390024 | 7.32E-07 | 7.90E-06 |
| RP11-540A21 | 2.133951306 | 1.54E-08 | 2.26E-07 |
| SPNS2 | 2.13356092 | 6.60E-19 | 3.41E-17 |
| FAM49A | 2.124423567 | 2.93E-05 | 0.00021987 |
| WWTR1 | 2.110566515 | 2.11E-29 | 2.98E-27 |
| FRAS1 | 2.094691768 | 3.92E-19 | 2.08E-17 |
| CD33 | 2.093915785 | 7.32E-11 | 1.50E-09 |
| PROSER2 | 2.090202393 | 6.88E-56 | 5.40E-53 |
| S1PR1 | 2.087188029 | 9.52E-09 | 1.44E-07 |
| LUM | 2.086699231 | 6.37E-07 | 6.96E-06 |
| RGS4 | 2.080933758 | 1.62E-05 | 0.00013007 |
| IL7R | 2.076081501 | 1.63E-35 | 3.67E-33 |
| ROS1 | 2.073108207 | 2.81E-17 | 1.23E-15 |
| MATN3 | 2.072939798 | 3.06E-13 | 8.51E-12 |
| NEXN | 2.064875841 | 2.09E-14 | 6.81E-13 |
| SNAPC1 | 2.064087081 | 3.13E-22 | 2.32E-20 |
| CDHR1 | 2.059371727 | 2.83E-06 | 2.72E-05 |

| | | | |
|---|---|---|---|
| CA9 | 2.048984166 | 0.00181086 | 0.00794627 |
| SPN | 2.029303899 | 0.00155257 | 0.00697606 |
| VGLL3 | 2.019542772 | 9.19E-20 | 5.28E-18 |
| TBC1D2 | 2.018432162 | 5.93E-29 | 8.07E-27 |
| HRK | 2.012178073 | 2.07E-12 | 5.17E-11 |
| SERPINB7 | 2.010717473 | 6.91E-16 | 2.67E-14 |
| EPB41L3 | 2.007400383 | 5.49E-12 | 1.30E-10 |
| CPA4 | 2.005531357 | 1.59E-06 | 1.60E-05 |
| GADD45B | 2.00459661 | 9.10E-43 | 3.41E-40 |
| CCBE1 | 1.997904082 | 1.86E-11 | 4.14E-10 |
| SLAMF7 | 1.997468447 | 6.25E-09 | 9.81E-08 |
| EXPH5 | 1.987808461 | 4.94E-14 | 1.56E-12 |
| CCDC80 | 1.976836703 | 1.79E-17 | 8.02E-16 |
| CHST15 | 1.962169426 | 0.00040208 | 0.00217789 |
| PLK2 | 1.953376518 | 9.47E-23 | 7.25E-21 |
| SCG5 | 1.948593597 | 1.07E-14 | 3.58E-13 |
| CYR61 | 1.941306958 | 4.04E-19 | 2.14E-17 |
| NPAS1 | 1.936654165 | 5.99E-37 | 1.41E-34 |
| IGFBP4 | 1.914205974 | 1.44E-28 | 1.88E-26 |
| TRIML2 | 1.913039903 | 1.78E-05 | 0.00014129 |
| ITGB2-AS1 | 1.907560271 | 8.73E-06 | 7.52E-05 |
| POSTN | 1.903894033 | 0.00046428 | 0.00246473 |
| NUAK1 | 1.897488099 | 5.70E-55 | 4.09E-52 |
| TGM1 | 1.893809747 | 4.82E-11 | 1.02E-09 |
| SAMD5 | 1.888562941 | 1.73E-09 | 2.94E-08 |
| CTGF | 1.888195211 | 2.76E-29 | 3.82E-27 |
| CCNA1 | 1.881778146 | 3.50E-13 | 9.67E-12 |
| GDA | 1.880739782 | 1.62E-17 | 7.29E-16 |
| CYP11A1 | 1.859901467 | 5.63E-13 | 1.49E-11 |
| TUFT1 | 1.859582741 | 7.33E-27 | 8.39E-25 |
| CTD-2130O1: | 1.849230956 | 6.53E-06 | 5.85E-05 |
| ADRB2 | 1.848621958 | 1.32E-14 | 4.38E-13 |
| DGKI | 1.847397121 | 1.94E-05 | 0.00015244 |
| CD163L1 | 1.827463003 | 4.54E-06 | 4.19E-05 |
| TGM2 | 1.823198291 | 7.68E-16 | 2.95E-14 |
| PNMA2 | 1.821277136 | 4.37E-09 | 7.00E-08 |
| SCN5A | 1.817597351 | 1.00E-07 | 1.28E-06 |
| MYO5B | 1.814594642 | 3.87E-12 | 9.38E-11 |
| MUC13 | 1.810793209 | 3.73E-07 | 4.29E-06 |
| UCP2 | 1.79222863 | 1.38E-10 | 2.71E-09 |
| RP1-102E24.( | 1.790920576 | 4.62E-14 | 1.47E-12 |
| GREB1 | 1.784308659 | 7.31E-12 | 1.69E-10 |
| EPB41L4B | 1.782743842 | 3.30E-11 | 7.13E-10 |
| C20orf197 | 1.778773754 | 3.13E-06 | 2.99E-05 |
| PKP2 | 1.778588667 | 1.59E-27 | 1.96E-25 |
| KCNK6 | 1.771770668 | 7.05E-08 | 9.23E-07 |
| UGCG | 1.760340867 | 3.88E-13 | 1.06E-11 |
| ZNF699 | 1.756763221 | 5.20E-21 | 3.38E-19 |
| OR51B2 | 1.752791638 | 0.0005424 | 0.00282306 |
| SAMD4A | 1.749179785 | 3.51E-32 | 5.85E-30 |
| GDPD5 | 1.748053637 | 1.85E-28 | 2.40E-26 |
| KIF1A | 1.742336909 | 9.19E-30 | 1.34E-27 |
| SUGCT | 1.741670956 | 7.78E-13 | 2.04E-11 |
| RP11-245M2 | 1.738793881 | 3.56E-13 | 9.82E-12 |
| NFASC | 1.732962643 | 7.51E-09 | 1.16E-07 |
| ADAMTS1 | 1.730764477 | 1.30E-15 | 4.85E-14 |

| | | | |
|---|---|---|---|
| DUSP1 | 1.726441902 | 6.92E-06 | 6.14E-05 |
| DDAH1 | 1.723432074 | 2.77E-44 | 1.17E-41 |
| PDGFB | 1.721348433 | 1.12E-13 | 3.34E-12 |
| PRR16 | 1.720433416 | 1.89E-12 | 4.75E-11 |
| KIRREL3 | 1.719021381 | 3.08E-15 | 1.09E-13 |
| PLCXD2 | 1.716633424 | 3.42E-06 | 3.23E-05 |
| PAGE1 | 1.712841083 | 6.06E-09 | 9.55E-08 |
| SLFNL1 | 1.709268634 | 7.25E-09 | 1.13E-07 |
| ARL14EPL | 1.706567236 | 0.00011967 | 0.00075818 |
| CD1D | 1.704213579 | 8.70E-08 | 1.12E-06 |
| KRT18P18 | 1.692444219 | 1.16E-12 | 2.98E-11 |
| SSR3 | 1.683796413 | 4.43E-38 | 1.16E-35 |
| USP2 | 1.680091701 | 2.34E-14 | 7.55E-13 |
| NLRP3 | 1.679116263 | 5.52E-06 | 5.00E-05 |
| FAP | 1.670014347 | 0.0001469 | 0.00090607 |
| RP11-386I14 | 1.667174317 | 1.10E-19 | 6.27E-18 |
| ZNF582 | 1.657102296 | 2.12E-12 | 5.29E-11 |
| RAB30 | 1.656101347 | 3.28E-29 | 4.50E-27 |
| NABP1 | 1.653174786 | 1.13E-26 | 1.27E-24 |
| DNAH11 | 1.652633343 | 4.04E-24 | 3.54E-22 |
| TRBC2 | 1.652101833 | 0.00164904 | 0.00734972 |
| LIMA1 | 1.648737006 | 2.34E-25 | 2.29E-23 |
| HHAT | 1.646834401 | 6.23E-20 | 3.63E-18 |
| IGFN1 | 1.645789327 | 1.03E-05 | 8.74E-05 |
| MAPK13 | 1.644902496 | 3.41E-09 | 5.54E-08 |
| HES2 | 1.642704003 | 5.03E-11 | 1.06E-09 |
| HES7 | 1.641055313 | 2.21E-23 | 1.81E-21 |
| ANKRD33B | 1.632036408 | 4.89E-12 | 1.17E-10 |
| PDE2A | 1.627083061 | 2.75E-06 | 2.65E-05 |
| ZNF431 | 1.626337994 | 7.94E-14 | 2.43E-12 |
| DOCK2 | 1.625287489 | 1.15E-07 | 1.45E-06 |
| LRP3 | 1.620897912 | 4.59E-07 | 5.19E-06 |
| ANKRD30A | 1.619178777 | 4.62E-10 | 8.48E-09 |
| MSX1 | 1.618425591 | 5.41E-31 | 8.41E-29 |
| CYTH3 | 1.615668359 | 1.44E-46 | 7.42E-44 |
| PLAC8 | 1.615437739 | 0.00024925 | 0.00143663 |
| MCTP2 | 1.59844963 | 1.30E-06 | 1.34E-05 |
| PRICKLE1 | 1.595737212 | 2.60E-05 | 0.00019826 |
| CREB5 | 1.589709896 | 6.66E-09 | 1.04E-07 |
| STC2 | 1.586924905 | 6.57E-10 | 1.18E-08 |
| PRR5 | 1.586393658 | 1.22E-20 | 7.62E-19 |
| KRT18 | 1.58608112 | 1.27E-28 | 1.69E-26 |
| ADTRP | 1.585117983 | 8.33E-05 | 0.00055807 |
| RP11-38P22. | 1.580751751 | 4.38E-12 | 1.05E-10 |
| P2RY1 | 1.577587693 | 2.23E-10 | 4.26E-09 |
| CLDN1 | 1.56623532 | 0.00083565 | 0.00410791 |
| CES3 | 1.561761355 | 8.39E-10 | 1.49E-08 |
| AC124789.1 | 1.561739159 | 3.29E-12 | 8.03E-11 |
| CTIF | 1.559551601 | 1.64E-66 | 3.37E-63 |
| FAM196B | 1.555881452 | 0.00123139 | 0.00573989 |
| KRT18P33 | 1.554915853 | 1.58E-12 | 4.00E-11 |
| OGFRL1 | 1.553700881 | 1.15E-22 | 8.80E-21 |
| PLA2G4C | 1.540836704 | 2.59E-05 | 0.0001978 |
| RPS6KL1 | 1.537548516 | 7.77E-12 | 1.79E-10 |
| FOXO6 | 1.526321163 | 3.17E-19 | 1.70E-17 |
| C16orf74 | 1.520499126 | 3.74E-13 | 1.02E-11 |

| | | | |
|---|---|---|---|
| TNS1 | 1.511671603 | 6.13E-05 | 0.0004238 |
| RP11-54F2.1 | 1.511627074 | 5.55E-07 | 6.13E-06 |
| MYCL | 1.507185087 | 4.96E-07 | 5.56E-06 |
| CTB-175P5.4 | 1.502865778 | 7.69E-16 | 2.95E-14 |
| HMOX1 | 1.502769221 | 8.21E-09 | 1.26E-07 |
| SCARA3 | 1.5004406 | 3.43E-08 | 4.76E-07 |
| RHOBTB1 | 1.493862985 | 1.38E-08 | 2.05E-07 |
| GPRC5B | 1.492641289 | 6.65E-07 | 7.24E-06 |
| CAV1 | 1.492576979 | 2.90E-14 | 9.31E-13 |
| C19orf33 | 1.486428504 | 0.00029743 | 0.00167737 |
| PDCD1LG2 | 1.48299546 | 1.18E-10 | 2.36E-09 |
| PALLD | 1.482098609 | 2.21E-16 | 9.05E-15 |
| HBEGF | 1.482030813 | 5.66E-27 | 6.66E-25 |
| FGD6 | 1.481598702 | 4.97E-09 | 7.91E-08 |
| CCDC85C | 1.481178703 | 1.26E-26 | 1.41E-24 |
| MBOAT2 | 1.479662594 | 8.00E-25 | 7.37E-23 |
| KRT18P31 | 1.476175794 | 1.78E-15 | 6.50E-14 |
| NOG | 1.472319269 | 2.12E-06 | 2.08E-05 |
| PRKCE | 1.471467798 | 4.99E-25 | 4.67E-23 |
| FAM101B | 1.471292419 | 1.21E-21 | 8.69E-20 |
| DENND2A | 1.470453922 | 2.75E-12 | 6.76E-11 |
| F2RL2 | 1.466410437 | 1.12E-05 | 9.41E-05 |
| ALDOC | 1.463428913 | 8.05E-13 | 2.11E-11 |
| MAOA | 1.462445623 | 1.85E-07 | 2.26E-06 |
| SLCO1A2 | 1.454356009 | 7.55E-07 | 8.13E-06 |
| DAPK1 | 1.449667329 | 5.42E-08 | 7.23E-07 |
| RP5-1028K7. | 1.446460041 | 0.00020595 | 0.00121774 |
| AC104389.28 | 1.445940978 | 7.43E-06 | 6.55E-05 |
| LINC00707 | 1.44427452 | 1.84E-11 | 4.11E-10 |
| PDLIM1P1 | 1.439914977 | 4.81E-07 | 5.42E-06 |
| NETO1 | 1.43955131 | 2.77E-11 | 6.03E-10 |
| GJB3 | 1.435469968 | 1.34E-08 | 2.00E-07 |
| AP001065.15 | 1.431668211 | 1.35E-08 | 2.00E-07 |
| SUSD1 | 1.42882577 | 3.47E-06 | 3.27E-05 |
| RAP1GAP2 | 1.427746561 | 5.43E-17 | 2.33E-15 |
| BTG2 | 1.42698657 | 1.27E-13 | 3.78E-12 |
| REEP1 | 1.424511284 | 3.62E-07 | 4.17E-06 |
| ARC | 1.423645248 | 1.16E-06 | 1.21E-05 |
| IL32 | 1.422000678 | 0.00065591 | 0.00333072 |
| PTX3 | 1.414984294 | 2.41E-06 | 2.35E-05 |
| BDNF | 1.414677637 | 2.29E-16 | 9.38E-15 |
| LMO7 | 1.412302677 | 6.74E-28 | 8.54E-26 |
| DKK1 | 1.408782113 | 7.37E-08 | 9.63E-07 |
| EPS8L2 | 1.405203603 | 2.03E-06 | 2.00E-05 |
| EFEMP1 | 1.404539281 | 2.36E-07 | 2.83E-06 |
| PTPRF | 1.402537389 | 1.45E-23 | 1.21E-21 |
| FUT1 | 1.396992556 | 0.00010082 | 0.00065568 |
| LSP1 | 1.396593757 | 5.01E-08 | 6.73E-07 |
| RP4-555D20. | 1.395697544 | 6.99E-05 | 0.00047672 |
| RCN3 | 1.39431762 | 5.70E-20 | 3.34E-18 |
| BCAM | 1.394301645 | 2.70E-05 | 0.00020402 |
| PPP1R1C | 1.387856695 | 1.64E-09 | 2.80E-08 |
| PODXL | 1.384991391 | 5.74E-20 | 3.35E-18 |
| TPM1 | 1.382958206 | 5.52E-07 | 6.11E-06 |
| SRGN | 1.380241984 | 7.01E-12 | 1.63E-10 |
| COL17A1 | 1.379760686 | 0.00012387 | 0.00078074 |

| | | | |
|---|---|---|---|
| JDP2 | 1.378805165 | 0.0008982 | 0.00437981 |
| ETS1 | 1.37850426 | 4.28E-20 | 2.54E-18 |
| FDXR | 1.376987815 | 9.55E-19 | 4.87E-17 |
| GADD45A | 1.373309377 | 6.97E-36 | 1.60E-33 |
| FAM83H | 1.37050707 | 9.78E-06 | 8.32E-05 |
| GRB14 | 1.370370699 | 0.0003235 | 0.00180703 |
| RGMB-AS1 | 1.364005826 | 3.21E-10 | 6.04E-09 |
| BNC1 | 1.36197599 | 1.32E-09 | 2.28E-08 |
| HMGCS1 | 1.358220221 | 5.17E-08 | 6.92E-07 |
| C10orf54 | 1.355642469 | 5.54E-07 | 6.13E-06 |
| SLC35F3 | 1.355130904 | 3.21E-10 | 6.04E-09 |
| DSC3 | 1.35282083 | 1.17E-17 | 5.37E-16 |
| ADD2 | 1.342818688 | 3.23E-07 | 3.78E-06 |
| RP11-879F14 | 1.336966903 | 8.95E-06 | 7.69E-05 |
| ARHGAP23 | 1.334307659 | 4.00E-23 | 3.16E-21 |
| GPR126 | 1.331107593 | 9.64E-38 | 2.44E-35 |
| SYT17 | 1.330799798 | 1.38E-06 | 1.40E-05 |
| MYLK | 1.327252574 | 3.64E-11 | 7.83E-10 |
| BCL11A | 1.322171827 | 7.55E-08 | 9.84E-07 |
| GALNT18 | 1.321683348 | 5.26E-06 | 4.78E-05 |
| ADAMTSL4 | 1.320409623 | 0.00011199 | 0.00071841 |
| CAV2 | 1.320025602 | 2.28E-17 | 1.01E-15 |
| KLF2 | 1.319106944 | 7.58E-07 | 8.15E-06 |
| ALPK2 | 1.317500216 | 0.00069968 | 0.00353446 |
| IL24 | 1.307840384 | 0.00064276 | 0.00327201 |
| SYNC | 1.299810884 | 0.00015564 | 0.00095344 |
| UAP1 | 1.296070861 | 3.78E-19 | 2.01E-17 |
| SBF2-AS1 | 1.294578152 | 2.77E-05 | 0.00020907 |
| MYPN | 1.292482758 | 3.22E-05 | 0.00023966 |
| FLRT2 | 1.292306987 | 4.01E-09 | 6.45E-08 |
| CDC42EP1 | 1.290591933 | 2.51E-08 | 3.56E-07 |
| KIAA1244 | 1.287403963 | 1.63E-15 | 5.99E-14 |
| IL12A | 1.280923146 | 1.34E-08 | 2.00E-07 |
| C4orf36 | 1.277702167 | 3.36E-09 | 5.46E-08 |
| AJUBA | 1.275579727 | 4.96E-23 | 3.87E-21 |
| CRIM1 | 1.271072793 | 3.32E-26 | 3.57E-24 |
| PALD1 | 1.267712425 | 1.95E-07 | 2.37E-06 |
| EEPD1 | 1.266796481 | 3.70E-06 | 3.47E-05 |
| VGLL2 | 1.265678411 | 0.00040303 | 0.00218178 |
| TSC22D2 | 1.254397974 | 3.07E-35 | 6.57E-33 |
| NRXN3 | 1.254071908 | 1.92E-06 | 1.90E-05 |
| LPAR1 | 1.253339525 | 0.00047438 | 0.00250704 |
| PKP4P1 | 1.252670288 | 1.10E-09 | 1.94E-08 |
| NMNAT2 | 1.244306499 | 2.75E-17 | 1.21E-15 |
| NLRP2 | 1.242665223 | 0.00014484 | 0.00089462 |
| PDLIM1 | 1.242638506 | 9.83E-07 | 1.03E-05 |
| PPARGC1B | 1.23939236 | 1.02E-07 | 1.30E-06 |
| RBMS2 | 1.239247857 | 1.01E-16 | 4.30E-15 |
| EXT1 | 1.23796178 | 2.34E-27 | 2.84E-25 |
| TUBB6 | 1.236794595 | 2.96E-20 | 1.78E-18 |
| GLCCI1 | 1.234047427 | 5.30E-13 | 1.42E-11 |
| LYPD6 | 1.232417086 | 2.34E-12 | 5.83E-11 |
| JUN | 1.23163188 | 1.53E-05 | 0.00012325 |
| RP11-1002K1 | 1.230976004 | 1.29E-05 | 0.00010609 |
| OSBP2 | 1.228918164 | 8.55E-07 | 9.13E-06 |
| SYDE2 | 1.228587689 | 4.32E-13 | 1.17E-11 |

| | | | |
|---|---|---|---|
| FADS3 | 1.224398385 | 1.08E-08 | 1.62E-07 |
| ADAMTS12 | 1.223966062 | 8.94E-13 | 2.33E-11 |
| SHROOM3 | 1.222006505 | 1.01E-14 | 3.40E-13 |
| SEMA3F | 1.219588894 | 4.82E-07 | 5.43E-06 |
| SGK223 | 1.210304598 | 4.62E-07 | 5.22E-06 |
| CISH | 1.208169471 | 7.01E-09 | 1.09E-07 |
| SCN8A | 1.206207689 | 1.85E-12 | 4.66E-11 |
| PAWR | 1.205513102 | 5.08E-14 | 1.60E-12 |
| PRKAG2 | 1.200697454 | 2.23E-13 | 6.39E-12 |
| CHRNB1 | 1.199987003 | 1.26E-10 | 2.49E-09 |
| RBMS2P1 | 1.198233588 | 1.83E-09 | 3.08E-08 |
| LACTB | 1.187923528 | 1.31E-15 | 4.86E-14 |
| XG | 1.187786366 | 1.08E-05 | 9.10E-05 |
| TXNRD2 | 1.187586673 | 1.28E-27 | 1.59E-25 |
| ARHGEF28 | 1.186962225 | 1.77E-16 | 7.33E-15 |
| BCL2L1 | 1.186524887 | 1.29E-26 | 1.43E-24 |
| ZNF788 | 1.186269215 | 1.45E-15 | 5.37E-14 |
| RP11-1100L3 | 1.186217147 | 1.18E-08 | 1.77E-07 |
| PKP4 | 1.184417158 | 8.48E-24 | 7.20E-22 |
| TENM2 | 1.183149902 | 0.00230423 | 0.00973271 |
| WNT10B | 1.182859607 | 2.98E-10 | 5.63E-09 |
| GTF2H2 | 1.182225711 | 0.00041358 | 0.00223007 |
| NEK3 | 1.18045109 | 0.00046071 | 0.00244894 |
| RP11-215G15 | 1.179765863 | 2.57E-14 | 8.24E-13 |
| LEPREL1 | 1.179029457 | 0.00036027 | 0.00197827 |
| CHCHD10 | 1.177788877 | 5.02E-16 | 1.98E-14 |
| FOSL2 | 1.177690161 | 2.68E-21 | 1.82E-19 |
| WTIP | 1.17629881 | 2.27E-13 | 6.46E-12 |
| RP11-14N7.2 | 1.17518189 | 1.49E-09 | 2.56E-08 |
| SAMD12 | 1.173569856 | 1.56E-13 | 4.54E-12 |
| AFF1 | 1.171763454 | 8.19E-35 | 1.71E-32 |
| TRPM2 | 1.170361965 | 1.15E-07 | 1.44E-06 |
| CMIP | 1.168872852 | 2.80E-35 | 6.07E-33 |
| RP11-289I10 | 1.16561675 | 8.12E-09 | 1.25E-07 |
| PRELP | 1.161207995 | 0.00022077 | 0.0012942 |
| TUBB4A | 1.153876776 | 0.00010353 | 0.00067145 |
| P2RX5 | 1.152601541 | 1.63E-08 | 2.38E-07 |
| PAQR3 | 1.149225901 | 4.38E-31 | 6.94E-29 |
| HSPA4L | 1.148487993 | 1.71E-06 | 1.71E-05 |
| LINC01021 | 1.148227259 | 2.79E-05 | 0.00021037 |
| GPR160 | 1.143301938 | 0.00014976 | 0.00092156 |
| MTL5 | 1.1413036 | 0.00012763 | 0.00080187 |
| ERVMER34-1 | 1.137094196 | 0.00036581 | 0.00200666 |
| COL4A6 | 1.134377518 | 6.06E-05 | 0.00041994 |
| ZNF582-AS1 | 1.132033194 | 2.12E-05 | 0.00016529 |
| CSMD3 | 1.130206065 | 8.02E-07 | 8.60E-06 |
| RFTN1 | 1.129459922 | 7.53E-15 | 2.56E-13 |
| KAZN | 1.128964394 | 5.83E-13 | 1.54E-11 |
| LINC00664 | 1.12646277 | 4.35E-09 | 6.98E-08 |
| CTC-241F20.3 | 1.12046409 | 1.77E-09 | 3.00E-08 |
| FAM212B | 1.116033786 | 3.93E-08 | 5.38E-07 |
| CARD11 | 1.115476125 | 2.63E-05 | 0.00020006 |
| MPP4 | 1.112202876 | 3.38E-07 | 3.93E-06 |
| RP11-673E1.3 | 1.110938053 | 2.61E-07 | 3.09E-06 |
| CORO1C | 1.108862346 | 5.88E-18 | 2.81E-16 |
| RP11-30P6.6 | 1.102623769 | 1.18E-06 | 1.23E-05 |

| | | | |
|---|---|---|---|
| NTN4 | 1.102310463 | 5.74E-17 | 2.46E-15 |
| CDKN1A | 1.100986288 | 1.33E-14 | 4.40E-13 |
| CDC20P1 | 1.100729108 | 4.35E-07 | 4.95E-06 |
| LINC00883 | 1.09890777 | 7.09E-15 | 2.43E-13 |
| TRIM22 | 1.096664225 | 1.21E-05 | 0.00010019 |
| ACER2 | 1.091941816 | 0.00089351 | 0.00436094 |
| LATS2 | 1.089753651 | 6.36E-19 | 3.30E-17 |
| ARHGAP30 | 1.089399999 | 0.00022946 | 0.00133896 |
| FEM1C | 1.086807304 | 1.43E-17 | 6.50E-16 |
| COL12A1 | 1.086164783 | 1.34E-09 | 2.32E-08 |
| NNMT | 1.084877439 | 0.00156039 | 0.0070039 |
| IRS1 | 1.084303667 | 5.96E-09 | 9.40E-08 |
| TERT | 1.08388457 | 4.86E-11 | 1.03E-09 |
| SAV1 | 1.078271403 | 5.15E-14 | 1.62E-12 |
| SHANK1 | 1.077981835 | 0.00010562 | 0.00068361 |
| DSP | 1.07778998 | 1.18E-07 | 1.48E-06 |
| RP11-554I8.2 | 1.074447044 | 1.82E-07 | 2.22E-06 |
| TNFAIP2 | 1.074344121 | 0.0001922 | 0.00114839 |
| SLC16A10 | 1.070345736 | 1.05E-09 | 1.84E-08 |
| NDRG1 | 1.064632599 | 1.33E-09 | 2.30E-08 |
| CLU | 1.064131858 | 1.06E-05 | 8.94E-05 |
| RP11-255H23 | 1.063622555 | 2.12E-06 | 2.08E-05 |
| GFOD1 | 1.063463997 | 3.03E-07 | 3.55E-06 |
| PINK1 | 1.059899292 | 4.90E-14 | 1.55E-12 |
| MAP3K1 | 1.059498739 | 1.12E-17 | 5.15E-16 |
| AC027612.6 | 1.056778933 | 5.00E-05 | 0.00035512 |
| DDX60L | 1.05636942 | 2.32E-15 | 8.39E-14 |
| DSE | 1.056202948 | 5.83E-09 | 9.20E-08 |
| ARVCF | 1.054588287 | 1.01E-08 | 1.53E-07 |
| ABLIM1 | 1.049433029 | 1.42E-05 | 0.00011527 |
| FJX1 | 1.04531594 | 5.47E-14 | 1.72E-12 |
| EVA1A | 1.045111404 | 1.08E-10 | 2.17E-09 |
| DUSP9 | 1.04281516 | 1.63E-12 | 4.11E-11 |
| ARHGEF26 | 1.040377588 | 8.67E-12 | 1.99E-10 |
| YOD1 | 1.039373082 | 3.30E-13 | 9.15E-12 |
| CYFIP2 | 1.039281482 | 2.95E-09 | 4.84E-08 |
| ALCAM | 1.038707461 | 9.45E-14 | 2.87E-12 |
| LLGL2 | 1.034190386 | 2.83E-09 | 4.66E-08 |
| RAB15 | 1.03136351 | 1.53E-12 | 3.90E-11 |
| GPR85 | 1.028667468 | 9.91E-07 | 1.04E-05 |
| CDR2L | 1.028162118 | 5.60E-13 | 1.49E-11 |
| TRIM59 | 1.026669833 | 1.16E-06 | 1.21E-05 |
| EGFR | 1.023683332 | 3.31E-12 | 8.08E-11 |
| ARID3A | 1.021526416 | 5.92E-05 | 0.00041188 |
| NEK6 | 1.019467364 | 1.38E-21 | 9.68E-20 |
| TEC | 1.017700545 | 1.74E-06 | 1.74E-05 |
| RASSF2 | 1.01635402 | 2.22E-05 | 0.00017235 |
| CDC20 | 1.014335309 | 7.87E-07 | 8.45E-06 |
| HS3ST3B1 | 1.014141218 | 5.81E-07 | 6.40E-06 |
| KLHL29 | 1.011151082 | 3.66E-11 | 7.86E-10 |
| TMOD3 | 1.010757117 | 1.59E-20 | 9.80E-19 |
| RP11-655M1 | 1.010730109 | 0.00235187 | 0.00988577 |
| CYCSP55 | 1.006980292 | 1.80E-09 | 3.03E-08 |
| ELL2P1 | 1.006054955 | 1.36E-05 | 0.00011078 |
| LINC00152 | 1.00542148 | 1.28E-08 | 1.91E-07 |
| EFNB1 | 1.003580264 | 1.53E-08 | 2.25E-07 |

| | | | |
|---|---|---|---|
| SIK1 | 0.998862685 | 4.96E-14 | 1.57E-12 |
| SEMA3G | 0.993532383 | 1.95E-05 | 0.00015287 |
| PVR | 0.991934375 | 9.85E-16 | 3.73E-14 |
| ZNF804A | 0.991809449 | 3.69E-13 | 1.01E-11 |
| GPX3 | 0.991367042 | 0.00128128 | 0.00593212 |
| AMOTL2 | 0.989668841 | 2.48E-17 | 1.10E-15 |
| RAB11FIP1 | 0.989616533 | 1.28E-21 | 9.11E-20 |
| IGFBP3 | 0.986988504 | 0.00087276 | 0.00426997 |
| GNAI1 | 0.98502946 | 4.77E-21 | 3.16E-19 |
| MAP1B | 0.983758295 | 3.20E-11 | 6.93E-10 |
| MED12L | 0.982904073 | 9.92E-06 | 8.43E-05 |
| ERBB2IP | 0.982584578 | 1.16E-21 | 8.35E-20 |
| ADAMTSL1 | 0.981751275 | 0.00010657 | 0.00068872 |
| FAM72C | 0.978806201 | 0.00092254 | 0.00448288 |
| TBC1D30 | 0.978068055 | 2.46E-06 | 2.39E-05 |
| MAST1 | 0.976529988 | 1.31E-05 | 0.00010795 |
| MB21D2 | 0.975531296 | 1.23E-08 | 1.84E-07 |
| PLD6 | 0.97483191 | 6.44E-08 | 8.50E-07 |
| MICAL2 | 0.974305999 | 5.17E-07 | 5.76E-06 |
| GREB1L | 0.971111048 | 2.01E-06 | 1.98E-05 |
| TMEM154 | 0.970324845 | 8.59E-08 | 1.10E-06 |
| SLC25A19 | 0.969292579 | 9.53E-08 | 1.22E-06 |
| DUSP3 | 0.969265053 | 6.76E-26 | 7.00E-24 |
| ANKRD13A | 0.967083831 | 1.89E-19 | 1.05E-17 |
| LZTS3 | 0.966626464 | 2.46E-11 | 5.41E-10 |
| MYL12A | 0.966570447 | 5.54E-08 | 7.38E-07 |
| ARG2 | 0.961813279 | 3.58E-08 | 4.96E-07 |
| FAM198B | 0.959209433 | 4.74E-07 | 5.35E-06 |
| BBC3 | 0.957457135 | 5.56E-07 | 6.14E-06 |
| HIF1A | 0.956566547 | 3.18E-19 | 1.70E-17 |
| C3orf52 | 0.955216966 | 4.48E-08 | 6.08E-07 |
| RP11-490M8 | 0.953925224 | 8.98E-06 | 7.70E-05 |
| FABP5 | 0.953507873 | 0.00039516 | 0.00214552 |
| PVRL2 | 0.952410121 | 1.90E-21 | 1.31E-19 |
| GCLM | 0.948005784 | 3.97E-07 | 4.55E-06 |
| DNAJB6 | 0.947915792 | 9.04E-14 | 2.75E-12 |
| ZNF442 | 0.947776885 | 0.00059627 | 0.00305896 |
| RRAS2 | 0.947628945 | 3.54E-07 | 4.09E-06 |
| RAB3IL1 | 0.946877855 | 9.48E-05 | 0.0006224 |
| ST3GAL1 | 0.946562675 | 8.15E-20 | 4.71E-18 |
| MSMO1 | 0.945611485 | 4.92E-05 | 0.00034993 |
| FAM167A | 0.943029106 | 3.42E-07 | 3.97E-06 |
| ZNF296 | 0.942080979 | 0.00030014 | 0.00169033 |
| MIR4435-1H( | 0.940405141 | 5.71E-11 | 1.19E-09 |
| RP11-611D2( | 0.938483474 | 1.79E-06 | 1.78E-05 |
| DUSP5 | 0.938465334 | 3.37E-05 | 0.0002496 |
| BARX1 | 0.937728649 | 1.96E-05 | 0.00015408 |
| MLK4 | 0.937504039 | 9.03E-11 | 1.82E-09 |
| RBM38 | 0.935360989 | 6.45E-11 | 1.34E-09 |
| FOXF2 | 0.9333714 | 9.05E-11 | 1.82E-09 |
| CHMP4C | 0.932290075 | 1.90E-05 | 0.0001493 |
| RP11-744K17 | 0.930621857 | 0.00087997 | 0.0043015 |
| RP11-671C19 | 0.930363476 | 8.03E-16 | 3.06E-14 |
| FEZ2 | 0.929729815 | 6.46E-15 | 2.22E-13 |
| ABL2 | 0.929590143 | 4.66E-09 | 7.44E-08 |
| RASL11A | 0.928905563 | 5.25E-06 | 4.78E-05 |

| | | | |
|---|---|---|---|
| BOK | 0.925808409 | 3.18E-16 | 1.28E-14 |
| CPNE7 | 0.921902041 | 9.09E-05 | 0.00060102 |
| FMNL1 | 0.919732431 | 9.45E-07 | 1.00E-05 |
| GCNT1 | 0.918754282 | 5.70E-05 | 0.00039769 |
| HK2P1 | 0.918733182 | 3.17E-06 | 3.02E-05 |
| WWC2 | 0.917568971 | 1.47E-16 | 6.11E-15 |
| LIN28B | 0.915416253 | 7.95E-07 | 8.53E-06 |
| MITF | 0.915254018 | 5.97E-10 | 1.08E-08 |
| KRT80 | 0.912924776 | 8.81E-05 | 0.00058676 |
| GPR176 | 0.912211048 | 5.13E-13 | 1.38E-11 |
| KCNH1 | 0.910399511 | 0.00011635 | 0.00074087 |
| DIAPH3 | 0.910363903 | 1.82E-12 | 4.59E-11 |
| AC005336.5 | 0.909199569 | 0.00014243 | 0.00088038 |
| ADAMTS7 | 0.908500685 | 5.81E-07 | 6.40E-06 |
| ZNF704 | 0.908455987 | 2.26E-06 | 2.22E-05 |
| PGM2 | 0.906581679 | 5.37E-17 | 2.32E-15 |
| TEAD4 | 0.90564336 | 2.48E-09 | 4.10E-08 |
| BAX | 0.905514786 | 1.85E-07 | 2.26E-06 |
| BNIP3P1 | 0.90384355 | 7.33E-09 | 1.14E-07 |
| FRMD6 | 0.903068763 | 1.01E-07 | 1.29E-06 |
| GLIPR1 | 0.901586789 | 3.11E-05 | 0.00023238 |
| PRKX | 0.900894374 | 4.06E-13 | 1.11E-11 |
| GLIPR2 | 0.900335865 | 0.00019523 | 0.00116394 |
| RIN2 | 0.899915429 | 1.61E-14 | 5.27E-13 |
| GATA2 | 0.898944886 | 1.20E-07 | 1.50E-06 |
| WNT3 | 0.89813419 | 6.31E-10 | 1.14E-08 |
| RP11-253E3.: | 0.897609347 | 2.59E-13 | 7.27E-12 |
| CNN3 | 0.896337279 | 1.44E-11 | 3.26E-10 |
| CYP2S1 | 0.896200332 | 0.00131281 | 0.00605428 |
| PDIA5 | 0.893732519 | 7.10E-07 | 7.69E-06 |
| SLC7A5 | 0.891716142 | 1.45E-08 | 2.14E-07 |
| FSTL3 | 0.891603455 | 4.46E-14 | 1.42E-12 |
| C2CD3 | 0.891182784 | 1.63E-14 | 5.33E-13 |
| RP11-216L13 | 0.890575161 | 6.42E-05 | 0.00044162 |
| RHBDF2 | 0.886501931 | 1.55E-09 | 2.65E-08 |
| RASSF5 | 0.884763546 | 2.49E-08 | 3.53E-07 |
| ETS2 | 0.884246163 | 1.97E-10 | 3.76E-09 |
| RAB20 | 0.883227117 | 3.36E-05 | 0.00024855 |
| SRXN1 | 0.882013156 | 0.00021695 | 0.001275 |
| CD3EAP | 0.881061576 | 0.00045485 | 0.00242094 |
| CHKA | 0.879692192 | 7.47E-15 | 2.55E-13 |
| RP3-510D11. | 0.878858799 | 1.38E-05 | 0.00011284 |
| BOP1 | 0.874542241 | 5.47E-05 | 0.00038342 |
| NRD1 | 0.874404099 | 1.80E-18 | 9.08E-17 |
| ASRGL1 | 0.874073038 | 6.73E-08 | 8.84E-07 |
| VOPP1 | 0.873469903 | 7.19E-12 | 1.67E-10 |
| FLNB | 0.87302305 | 3.48E-13 | 9.61E-12 |
| ZNF714 | 0.871907211 | 1.96E-10 | 3.75E-09 |
| TM4SF19 | 0.871883491 | 1.44E-06 | 1.46E-05 |
| PROCR | 0.870500248 | 2.49E-19 | 1.37E-17 |
| MROH1 | 0.868491664 | 1.05E-07 | 1.34E-06 |
| METRNL | 0.86546938 | 2.66E-12 | 6.58E-11 |
| NAV2 | 0.864048393 | 0.0007048 | 0.00355487 |
| DHCR7 | 0.863171617 | 4.83E-07 | 5.43E-06 |
| PCID2 | 0.862320994 | 8.70E-14 | 2.65E-12 |
| ZNF488 | 0.862241141 | 0.00018272 | 0.00110013 |

| | | | |
|---|---|---|---|
| RAB32 | 0.862173925 | 2.25E-13 | 6.43E-12 |
| FIGN | 0.861077265 | 5.09E-08 | 6.83E-07 |
| PDGFA | 0.860411858 | 1.53E-05 | 0.00012317 |
| ERCC2 | 0.859949968 | 3.54E-14 | 1.13E-12 |
| GLUL | 0.855417996 | 5.52E-07 | 6.12E-06 |
| TFRC | 0.854624132 | 7.66E-09 | 1.18E-07 |
| KLF5 | 0.853552418 | 1.28E-15 | 4.77E-14 |
| NLRP1 | 0.85280058 | 7.08E-10 | 1.27E-08 |
| SEC14L1 | 0.851866361 | 1.24E-19 | 7.04E-18 |
| SLC9A3R1 | 0.845693205 | 3.35E-08 | 4.66E-07 |
| BNIP3 | 0.845671634 | 6.55E-11 | 1.35E-09 |
| STARD8 | 0.843893184 | 0.0023428 | 0.00986528 |
| RP11-548L20 | 0.843616346 | 0.00158873 | 0.00711558 |
| SPHK1 | 0.842776897 | 0.00037022 | 0.0020268 |
| EZR | 0.841870525 | 7.03E-14 | 2.16E-12 |
| IL6R | 0.841055593 | 0.00022788 | 0.0013307 |
| USP18 | 0.840366464 | 1.77E-05 | 0.00014023 |
| TMEM160 | 0.838865539 | 1.79E-09 | 3.03E-08 |
| CCDC86 | 0.837602283 | 1.89E-08 | 2.72E-07 |
| CHORDC1 | 0.837340267 | 0.00199717 | 0.00865091 |
| SOGA2 | 0.83724795 | 3.09E-12 | 7.57E-11 |
| DSEL | 0.837109835 | 4.82E-07 | 5.42E-06 |
| MRS2 | 0.836530096 | 3.74E-15 | 1.32E-13 |
| TNFSF9 | 0.836361588 | 0.00047535 | 0.00251138 |
| HERC5 | 0.835229278 | 0.00013585 | 0.00084605 |
| RPS27L | 0.834244251 | 1.55E-07 | 1.91E-06 |
| RUSC2 | 0.833897379 | 5.35E-12 | 1.27E-10 |
| MEX3B | 0.830330159 | 5.04E-07 | 5.63E-06 |
| RP11-412P11 | 0.830306144 | 0.00027538 | 0.00156585 |
| RGS2 | 0.829296976 | 1.52E-05 | 0.00012284 |
| PINX1 | 0.827935643 | 0.00036907 | 0.00202321 |
| C12orf75 | 0.827835087 | 7.01E-15 | 2.41E-13 |
| NRIP3 | 0.827527263 | 1.19E-05 | 9.91E-05 |
| DLC1 | 0.825059352 | 1.20E-07 | 1.50E-06 |
| BTBD10 | 0.823711833 | 1.39E-12 | 3.53E-11 |
| KIAA1522 | 0.822902746 | 9.72E-06 | 8.28E-05 |
| ATAD3B | 0.822595631 | 1.72E-07 | 2.11E-06 |
| DENND3 | 0.822440617 | 1.73E-08 | 2.50E-07 |
| MARS2 | 0.820895534 | 1.34E-05 | 0.00010959 |
| TRIO | 0.820652194 | 1.15E-07 | 1.44E-06 |
| RDM1 | 0.820626754 | 0.00110011 | 0.00521652 |
| PM20D2 | 0.81956566 | 6.68E-16 | 2.59E-14 |
| CHML | 0.819344435 | 1.14E-07 | 1.44E-06 |
| EBNA1BP2 | 0.819188872 | 2.50E-09 | 4.12E-08 |
| IER5 | 0.817966905 | 5.65E-13 | 1.50E-11 |
| FLII | 0.817172393 | 6.12E-13 | 1.61E-11 |
| CDC42EP2 | 0.816606831 | 2.50E-07 | 2.98E-06 |
| RPS27P27 | 0.815169367 | 2.41E-05 | 0.00018582 |
| TXNRD1 | 0.813391981 | 1.28E-13 | 3.78E-12 |
| MAGEA1 | 0.812345223 | 0.00103198 | 0.00493606 |
| PDGFRB | 0.810489517 | 0.00121167 | 0.00566879 |
| MPP5 | 0.810446876 | 2.95E-09 | 4.84E-08 |
| LRRC20 | 0.809949087 | 6.14E-07 | 6.73E-06 |
| FHL1 | 0.809551349 | 0.00164959 | 0.00735019 |
| EMR2 | 0.809063621 | 0.00033302 | 0.00185331 |
| ZNF331 | 0.809061484 | 1.22E-10 | 2.42E-09 |

| | | | |
|---|---|---|---|
| LCAT | 0.806862812 | 0.00042346 | 0.00227548 |
| SMURF2 | 0.804572473 | 1.04E-05 | 8.76E-05 |
| NT5C2 | 0.804213264 | 2.60E-12 | 6.45E-11 |
| GCAT | 0.803962859 | 2.52E-08 | 3.56E-07 |
| CYP2J2 | 0.802881483 | 0.00022313 | 0.00130572 |
| CHIC2 | 0.802103107 | 2.61E-10 | 4.94E-09 |
| ALDH1B1 | 0.801928412 | 5.19E-12 | 1.23E-10 |
| CALD1 | 0.801168238 | 9.38E-11 | 1.88E-09 |
| TMSB4XP8 | 0.800994228 | 0.00023744 | 0.00138061 |
| NR2F2 | 0.800231884 | 7.47E-09 | 1.16E-07 |
| YDJC | 0.800143284 | 5.03E-17 | 2.17E-15 |
| RP11-676M6 | 0.798297073 | 8.55E-12 | 1.97E-10 |
| PHLDA3 | 0.796928383 | 2.24E-09 | 3.74E-08 |
| SERINC2 | 0.796120235 | 0.00014234 | 0.00088017 |
| SIPA1L3 | 0.794814367 | 9.92E-14 | 2.99E-12 |
| KCNQ5 | 0.794262273 | 9.72E-06 | 8.28E-05 |
| PDLIM2 | 0.793629894 | 8.29E-11 | 1.68E-09 |
| TP53INP1 | 0.792088813 | 1.14E-08 | 1.71E-07 |
| MPP3 | 0.791451655 | 0.00011014 | 0.00070846 |
| PNP | 0.788499925 | 1.04E-08 | 1.56E-07 |
| SLC22A15 | 0.787752801 | 0.00122495 | 0.00571472 |
| CTPS1 | 0.786930036 | 4.74E-06 | 4.36E-05 |
| CLUH | 0.784635948 | 3.19E-08 | 4.46E-07 |
| UNC13A | 0.783229927 | 0.00023448 | 0.00136536 |
| KBTBD8 | 0.780464159 | 0.002358 | 0.00990649 |
| NBEAL2 | 0.779448926 | 5.42E-06 | 4.91E-05 |
| EEF1A2 | 0.778712033 | 0.00010064 | 0.00065495 |
| PPIF | 0.777213475 | 1.23E-14 | 4.07E-13 |
| BCL9L | 0.776315772 | 1.09E-11 | 2.48E-10 |
| NLN | 0.77364106 | 2.07E-06 | 2.05E-05 |
| EGFLAM | 0.773347117 | 0.00140134 | 0.00639985 |
| DRAM1 | 0.772906331 | 9.43E-09 | 1.43E-07 |
| EDA2R | 0.772491407 | 2.15E-05 | 0.00016725 |
| ACTA2 | 0.771262292 | 0.00223028 | 0.00946649 |
| UCHL3 | 0.77000616 | 0.00018857 | 0.0011312 |
| ELL2 | 0.76920739 | 2.31E-06 | 2.26E-05 |
| DUSP2 | 0.76874712 | 8.48E-07 | 9.08E-06 |
| IL15RA | 0.76641865 | 0.00039876 | 0.00216218 |
| PHLPP1 | 0.765701434 | 3.73E-08 | 5.14E-07 |
| STX3 | 0.764568368 | 1.92E-10 | 3.68E-09 |
| BMP2K | 0.761840385 | 1.52E-05 | 0.00012256 |
| ZP3 | 0.761838518 | 0.00076045 | 0.00380064 |
| RP11-660L16 | 0.761833856 | 0.00049756 | 0.0026096 |
| RP11-309G3. | 0.759221551 | 0.00227735 | 0.00963646 |
| NFIA | 0.758962562 | 1.35E-06 | 1.38E-05 |
| BAG2 | 0.75673572 | 6.65E-07 | 7.24E-06 |
| PEA15 | 0.75573536 | 1.46E-10 | 2.85E-09 |
| SLC17A9 | 0.754775799 | 1.35E-14 | 4.45E-13 |
| ZFPM2 | 0.754509662 | 4.88E-08 | 6.57E-07 |
| ARID3B | 0.751797196 | 0.00059009 | 0.00303766 |
| PLK3 | 0.750663443 | 5.23E-08 | 7.00E-07 |
| RINL | 0.74738935 | 5.08E-05 | 0.00035962 |
| IMPDH1 | 0.74665687 | 4.48E-06 | 4.13E-05 |
| KIAA1211 | 0.746230054 | 7.04E-06 | 6.24E-05 |
| DLG1 | 0.746033568 | 6.51E-14 | 2.02E-12 |
| LPAR3 | 0.745922656 | 6.82E-11 | 1.41E-09 |

| | | | |
|---|---|---|---|
| AC073150.6 | 0.745558682 | 0.00182947 | 0.00801938 |
| OPN3 | 0.744954799 | 0.00090798 | 0.00441735 |
| RP1-152L7.5 | 0.743912218 | 5.04E-07 | 5.63E-06 |
| CTU1 | 0.743879866 | 3.39E-06 | 3.20E-05 |
| CTDSPL | 0.743877502 | 1.28E-09 | 2.23E-08 |
| IRAK1 | 0.742654706 | 1.47E-09 | 2.53E-08 |
| LRRC8E | 0.741728246 | 0.00135709 | 0.00622365 |
| ACSL1 | 0.741552547 | 1.40E-13 | 4.11E-12 |
| CRY1 | 0.740344054 | 5.09E-11 | 1.07E-09 |
| RP11-713C19 | 0.74005105 | 1.98E-13 | 5.70E-12 |
| RELL2 | 0.73981677 | 0.00067838 | 0.00343636 |
| SLC8B1 | 0.739399608 | 8.74E-09 | 1.34E-07 |
| TMEM144 | 0.737564081 | 2.32E-05 | 0.00017994 |
| PDGFC | 0.734523783 | 8.49E-10 | 1.51E-08 |
| ANKRD34A | 0.733736819 | 3.03E-09 | 4.95E-08 |
| EFCAB4B | 0.731600788 | 1.46E-08 | 2.16E-07 |
| RP11-564D11 | 0.731485788 | 0.00013338 | 0.00083255 |
| ARHGAP29 | 0.731305837 | 4.82E-06 | 4.42E-05 |
| CLTB | 0.730844209 | 1.28E-09 | 2.22E-08 |
| RPL26L1 | 0.730185815 | 4.86E-08 | 6.54E-07 |
| SQLE | 0.730088839 | 8.50E-06 | 7.36E-05 |
| TAF1A | 0.729856798 | 6.87E-06 | 6.11E-05 |
| SLC29A2 | 0.728451519 | 2.70E-05 | 0.00020402 |
| SCARF2 | 0.727273939 | 2.15E-07 | 2.59E-06 |
| C17orf51 | 0.727203342 | 8.24E-06 | 7.17E-05 |
| GET4 | 0.72706079 | 9.08E-05 | 0.00060054 |
| RP11-686D22 | 0.726648948 | 0.00042141 | 0.00226713 |
| POLRMTP1 | 0.725640998 | 6.38E-06 | 5.73E-05 |
| ITPK1 | 0.724288737 | 8.85E-09 | 1.35E-07 |
| FAM162A | 0.723990941 | 9.46E-09 | 1.44E-07 |
| CDV3 | 0.723592534 | 4.38E-11 | 9.32E-10 |
| PPP1R13L | 0.72341833 | 0.00025356 | 0.00145791 |
| TBC1D23 | 0.722377409 | 5.62E-14 | 1.76E-12 |
| TRIP6 | 0.721881351 | 2.80E-15 | 1.00E-13 |
| GRK6P1 | 0.72124831 | 0.00019523 | 0.00116394 |
| FZD4 | 0.720028554 | 9.04E-05 | 0.00059871 |
| GPR63 | 0.718627875 | 0.00098954 | 0.00475938 |
| CCDC69 | 0.718556852 | 9.76E-05 | 0.00063811 |
| NCEH1 | 0.716535072 | 3.36E-09 | 5.47E-08 |
| RASSF7 | 0.71630029 | 1.65E-06 | 1.65E-05 |
| SMPD3 | 0.716120385 | 0.00087999 | 0.0043015 |
| BATF2 | 0.715844255 | 0.00098055 | 0.00472711 |
| NTMT1 | 0.715348724 | 9.21E-09 | 1.40E-07 |
| RP11-350E12 | 0.713611434 | 9.07E-05 | 0.00060013 |
| HK2 | 0.712710317 | 2.43E-06 | 2.37E-05 |
| PDCD2L | 0.709205652 | 2.46E-07 | 2.94E-06 |
| COMTD1 | 0.707324884 | 0.00109295 | 0.00518854 |
| JADE3 | 0.706509888 | 9.99E-06 | 8.48E-05 |
| CDC25A | 0.705805498 | 3.32E-05 | 0.00024618 |
| DAB2 | 0.704951231 | 7.56E-06 | 6.64E-05 |
| MESDC1 | 0.70480392 | 1.42E-07 | 1.76E-06 |
| PKN1 | 0.70299016 | 2.50E-13 | 7.07E-12 |
| ADA | 0.701940674 | 2.25E-06 | 2.21E-05 |
| RPL23AP82 | 0.700850262 | 0.00154899 | 0.00696219 |
| SRGAP1 | 0.700501667 | 0.001026 | 0.00491319 |
| GNB1L | 0.700210694 | 7.49E-05 | 0.00050778 |

| | | | |
|---|---|---|---|
| FTH1 | 0.699629221 | 0.00135453 | 0.00621885 |
| ARHGEF17 | 0.699096763 | 6.44E-06 | 5.78E-05 |
| CHST2 | 0.69894792 | 0.00192706 | 0.00839135 |
| SPIRE1 | 0.69867398 | 9.25E-09 | 1.41E-07 |
| EPG5 | 0.69860118 | 1.09E-05 | 9.19E-05 |
| ATP5G1P4 | 0.698433963 | 0.00114786 | 0.00540557 |
| DAPK3 | 0.698150477 | 8.09E-12 | 1.86E-10 |
| SPAG1 | 0.697240264 | 1.44E-06 | 1.46E-05 |
| CCDC84 | 0.696728508 | 3.76E-06 | 3.52E-05 |
| QSOX1 | 0.696601246 | 6.16E-14 | 1.92E-12 |
| PDXK | 0.695629077 | 6.26E-10 | 1.13E-08 |
| PRPS1 | 0.694596825 | 1.58E-10 | 3.06E-09 |
| FAM117A | 0.694524507 | 8.86E-05 | 0.0005887 |
| C20orf27 | 0.692812522 | 2.74E-11 | 5.96E-10 |
| NMT2 | 0.692705532 | 0.00205941 | 0.00887847 |
| RPL26P30 | 0.692323319 | 8.36E-05 | 0.00055988 |
| SDC1 | 0.692042719 | 2.55E-06 | 2.47E-05 |
| SESN1 | 0.69026306 | 0.00021566 | 0.0012683 |
| GBGT1 | 0.687451305 | 3.41E-07 | 3.96E-06 |
| CTA-29F11.1 | 0.686545304 | 2.65E-05 | 0.00020094 |
| ZNF25 | 0.685163441 | 0.00035802 | 0.00196718 |
| SKP2 | 0.683305688 | 1.68E-08 | 2.44E-07 |
| HPRT1 | 0.682798827 | 9.52E-14 | 2.88E-12 |
| TSPAN33 | 0.682476147 | 0.00108641 | 0.00516194 |
| TNPO1P1 | 0.682265018 | 0.00026313 | 0.00150402 |
| C20orf24 | 0.679951318 | 4.41E-05 | 0.0003177 |
| CTD-2561J22 | 0.679523609 | 0.00010477 | 0.00067842 |
| FMN2 | 0.67908109 | 2.58E-12 | 6.40E-11 |
| FLI1 | 0.678995605 | 0.0012169 | 0.005689 |
| UBE2J1 | 0.67861447 | 6.91E-10 | 1.24E-08 |
| TXNP4 | 0.677712884 | 0.00209926 | 0.00901617 |
| KIF1C | 0.6773231 | 2.03E-08 | 2.90E-07 |
| NUDCD1 | 0.677319966 | 5.81E-09 | 9.19E-08 |
| ANXA2 | 0.677233005 | 8.06E-05 | 0.00054264 |
| NANP | 0.676692508 | 3.82E-11 | 8.16E-10 |
| MAPK12 | 0.676181413 | 1.31E-05 | 0.00010731 |
| FHL3 | 0.67532712 | 4.87E-05 | 0.00034695 |
| CASP4 | 0.674947078 | 0.00017202 | 0.00104338 |
| TOMM40 | 0.673833952 | 3.63E-08 | 5.02E-07 |
| SLC27A5 | 0.671053357 | 2.65E-07 | 3.14E-06 |
| C12orf5 | 0.670421494 | 9.58E-06 | 8.17E-05 |
| TSEN15P1 | 0.66809401 | 0.0017295 | 0.00764015 |
| MIEF2 | 0.667758629 | 1.41E-05 | 0.00011476 |
| ZNF330 | 0.667103295 | 3.90E-12 | 9.45E-11 |
| NANS | 0.667091342 | 4.29E-12 | 1.03E-10 |
| MTSS1L | 0.667049321 | 3.04E-11 | 6.61E-10 |
| PHLDB2 | 0.66651354 | 3.07E-07 | 3.60E-06 |
| RRAGD | 0.665813484 | 3.25E-06 | 3.08E-05 |
| RP11-177C12 | 0.665798856 | 0.00119596 | 0.00560166 |
| PDE1C | 0.664864713 | 4.10E-06 | 3.81E-05 |
| RP11-395L14 | 0.664344633 | 0.0003981 | 0.00215932 |
| EPHA2 | 0.664028094 | 2.42E-05 | 0.0001863 |
| HSPD1P5 | 0.663568385 | 0.00100459 | 0.00482043 |
| PAICSP3 | 0.663532868 | 0.00232243 | 0.00979953 |
| AC009299.5 | 0.663320652 | 3.90E-05 | 0.00028416 |
| PGK1P2 | 0.663252127 | 1.28E-06 | 1.32E-05 |

| | | | |
|---|---|---|---|
| SLC43A2 | 0.662540598 | 0.00109972 | 0.00521613 |
| PLCD3 | 0.661487237 | 7.84E-06 | 6.86E-05 |
| MVD | 0.659413211 | 3.16E-05 | 0.00023564 |
| SH3BP4 | 0.659158176 | 4.45E-08 | 6.04E-07 |
| FCRLB | 0.658281309 | 0.00040889 | 0.00220912 |
| TRAP1 | 0.656737829 | 7.22E-10 | 1.29E-08 |
| CCDC124 | 0.655615002 | 3.06E-07 | 3.59E-06 |
| RP11-528A10 | 0.6543951 | 0.00229019 | 0.00968334 |
| RP11-438N16 | 0.654129545 | 3.37E-10 | 6.32E-09 |
| HDAC7 | 0.653346136 | 7.63E-09 | 1.18E-07 |
| INO80C | 0.653345637 | 2.34E-05 | 0.00018104 |
| RP11-423H2. | 0.652769364 | 0.0001058 | 0.00068457 |
| HOMER2 | 0.652514169 | 4.35E-05 | 0.00031424 |
| ANKZF1 | 0.652435307 | 3.57E-10 | 6.68E-09 |
| PNO1 | 0.652211272 | 1.11E-05 | 9.32E-05 |
| LETM2 | 0.651419284 | 0.00034228 | 0.00189638 |
| GXYLT1 | 0.651338445 | 4.29E-07 | 4.89E-06 |
| AC104297.1 | 0.651334871 | 1.88E-05 | 0.00014801 |
| RNASET2 | 0.651160955 | 8.77E-05 | 0.00058495 |
| STRIP2 | 0.650810826 | 3.06E-05 | 0.00022851 |
| SGMS2 | 0.650793125 | 9.67E-05 | 0.00063313 |
| VRK2 | 0.648515877 | 9.95E-07 | 1.05E-05 |
| SAPCD2 | 0.648200166 | 0.00027227 | 0.00155144 |
| WT1 | 0.647456007 | 4.13E-05 | 0.00029925 |
| RP11-162O12 | 0.646493869 | 8.25E-06 | 7.17E-05 |
| WDR77 | 0.646171641 | 0.00018996 | 0.00113834 |
| UBE2SP1 | 0.645279644 | 5.59E-10 | 1.02E-08 |
| FAR1P1 | 0.644562997 | 0.00170712 | 0.00756244 |
| RP11-516A11 | 0.644423869 | 1.20E-05 | 9.97E-05 |
| RP11-807E13 | 0.644081612 | 0.0006572 | 0.00333624 |
| SMCO4 | 0.642367454 | 0.0014598 | 0.00661912 |
| MYL12B | 0.64149065 | 9.84E-11 | 1.97E-09 |
| RP11-2C24.5 | 0.641091483 | 0.00161034 | 0.00720259 |
| RP11-641A6. | 0.641080889 | 0.00154718 | 0.00695687 |
| UBE2S | 0.640828398 | 1.20E-09 | 2.09E-08 |
| RHOF | 0.639965719 | 0.00058436 | 0.00301268 |
| TAX1BP3 | 0.639216555 | 5.44E-06 | 4.93E-05 |
| ACO1 | 0.639010188 | 1.41E-06 | 1.43E-05 |
| ARRB1 | 0.638877016 | 0.00206867 | 0.00891139 |
| ZFP42 | 0.638421923 | 9.89E-05 | 0.00064491 |
| ASCC3 | 0.638019532 | 0.00042204 | 0.00226977 |
| SLC7A6 | 0.637860078 | 2.23E-07 | 2.69E-06 |
| FAM207A | 0.637401844 | 0.000166 | 0.00101053 |
| ATP5G1P5 | 0.637047608 | 0.00041229 | 0.00222385 |
| GABARAPL1 | 0.636299154 | 4.73E-10 | 8.66E-09 |
| TTC27 | 0.636285535 | 4.17E-05 | 0.00030164 |
| LRFN4 | 0.635372808 | 5.54E-11 | 1.16E-09 |
| RASAL2 | 0.635250106 | 0.00217886 | 0.0092865 |
| MCC | 0.635232336 | 5.02E-05 | 0.00035614 |
| RP11-467J12 | 0.634876945 | 0.00178963 | 0.00787408 |
| PROSER1 | 0.634623727 | 8.83E-10 | 1.57E-08 |
| RP11-142L4.2 | 0.634125605 | 0.00081585 | 0.00403277 |
| PUS1 | 0.63323612 | 1.22E-05 | 0.00010087 |
| DOCK9 | 0.633218066 | 0.00033858 | 0.00188043 |
| SLFN12 | 0.63320755 | 8.04E-06 | 7.02E-05 |
| SPTLC2 | 0.633046122 | 4.22E-09 | 6.78E-08 |

| | | | |
|---|---|---|---|
| C3orf58 | 0.632673491 | 8.42E-08 | 1.09E-06 |
| RRP1B | 0.631875655 | 6.37E-06 | 5.72E-05 |
| LINC01029 | 0.631790646 | 3.14E-05 | 0.00023418 |
| DSG2 | 0.630290146 | 1.56E-10 | 3.04E-09 |
| AC093673.5 | 0.629684064 | 0.00075201 | 0.00376532 |
| PWWP2B | 0.629181564 | 2.64E-05 | 0.00020061 |
| CCDC58 | 0.628705158 | 0.00030524 | 0.00171439 |
| TRIM7 | 0.628697652 | 0.00029327 | 0.00165669 |
| STRBP | 0.628145714 | 7.41E-06 | 6.53E-05 |
| ZNF598 | 0.627202466 | 2.61E-10 | 4.94E-09 |
| FTLP3 | 0.627074807 | 1.62E-06 | 1.63E-05 |
| RP11-365D23 | 0.626401661 | 0.00227435 | 0.00962624 |
| TTC7A | 0.626320464 | 4.02E-07 | 4.61E-06 |
| FTL | 0.625476733 | 1.58E-06 | 1.59E-05 |
| POLRMT | 0.623130079 | 3.01E-06 | 2.87E-05 |
| ATAD3A | 0.621823824 | 2.65E-08 | 3.74E-07 |
| TRAM2 | 0.621084267 | 2.49E-11 | 5.47E-10 |
| TNPO1 | 0.620825735 | 1.76E-10 | 3.39E-09 |
| PFAS | 0.620599059 | 7.64E-05 | 0.00051704 |
| RP11-265N6. | 0.620444383 | 4.96E-07 | 5.56E-06 |
| ANXA2P2 | 0.619697371 | 0.00032527 | 0.0018163 |
| TMSB4X | 0.619510015 | 0.00091128 | 0.00443208 |
| AP000439.1 | 0.619400661 | 0.00024871 | 0.00143405 |
| RAB11FIP5 | 0.617311625 | 7.87E-11 | 1.60E-09 |
| FLNA | 0.616591062 | 1.80E-10 | 3.46E-09 |
| CECR2 | 0.615726013 | 0.00233252 | 0.00983469 |
| DUSP16 | 0.615250769 | 1.42E-05 | 0.00011525 |
| HAUS2 | 0.615219851 | 1.44E-08 | 2.13E-07 |
| PNPT1 | 0.614885471 | 0.00019904 | 0.00118199 |
| SLC7A6OS | 0.613806697 | 0.00013073 | 0.00081841 |
| PARD3B | 0.613656988 | 0.0006712 | 0.003401 |
| RP1-273G13. | 0.613426773 | 9.72E-05 | 0.00063618 |
| LINC00665 | 0.613189432 | 2.29E-07 | 2.75E-06 |
| PLOD2 | 0.612922368 | 1.77E-08 | 2.57E-07 |
| DUSP7 | 0.612536832 | 1.31E-06 | 1.34E-05 |
| STAP2 | 0.612188138 | 0.00034054 | 0.0018894 |
| FTLP2 | 0.611969751 | 5.12E-06 | 4.68E-05 |
| PPM1H | 0.609595305 | 0.00048189 | 0.00254185 |
| FASN | 0.609406491 | 1.06E-06 | 1.10E-05 |
| RHPN2 | 0.609097435 | 1.59E-08 | 2.32E-07 |
| GRK6 | 0.606668325 | 1.57E-09 | 2.68E-08 |
| GPRC5A | 0.606645515 | 0.0019997 | 0.00865731 |
| HOXA1 | 0.606629834 | 0.00220148 | 0.00937324 |
| JPH1 | 0.605652288 | 7.78E-06 | 6.81E-05 |
| CMSS1 | 0.605626133 | 5.13E-09 | 8.15E-08 |
| LRRC8B | 0.605330233 | 0.00032003 | 0.00179133 |
| PNPT1P1 | 0.604901775 | 0.00097079 | 0.00468556 |
| ZNF430 | 0.604012842 | 9.43E-05 | 0.00061972 |
| KIAA1671 | 0.603971628 | 0.00066875 | 0.00338962 |
| CMBL | 0.60334197 | 9.87E-10 | 1.74E-08 |
| SLC8A1 | 0.603283656 | 0.00133454 | 0.0061476 |
| DYRK2 | 0.603136464 | 4.43E-09 | 7.08E-08 |
| HPDL | 0.602375943 | 8.54E-06 | 7.38E-05 |
| RP11-601I15. | 0.602148627 | 0.00027722 | 0.00157578 |
| ANKRD27 | 0.601285891 | 2.22E-06 | 2.18E-05 |
| GCH1 | 0.601199025 | 0.00019761 | 0.00117602 |

| | | | |
|---|---|---|---|
| PGAM1 | 0.60098549 | 0.00017009 | 0.00103238 |
| HOXD13 | 0.600916805 | 0.00012612 | 0.00079357 |
| MRPS5 | 0.60085806 | 2.21E-08 | 3.15E-07 |
| SLC7A2 | 0.600619086 | 1.09E-06 | 1.14E-05 |
| RP11-134G8. | 0.600207212 | 0.00231434 | 0.00976789 |
| PRDX6 | 0.600032814 | 8.63E-11 | 1.75E-09 |
| PIP5K1P1 | 0.599408823 | 0.00048594 | 0.00256158 |
| TRIM14 | 0.599174393 | 9.85E-08 | 1.26E-06 |
| NEDD4L | 0.599065921 | 3.19E-06 | 3.03E-05 |
| ASL | 0.598752604 | 0.00046722 | 0.00247475 |
| MID1 | 0.597597244 | 1.69E-05 | 0.00013468 |
| FAM160B1 | 0.59756021 | 4.31E-06 | 3.99E-05 |
| TJP1 | 0.597537385 | 9.95E-08 | 1.27E-06 |
| KLHL18 | 0.597404133 | 6.11E-05 | 0.00042315 |
| CSRNP1 | 0.597351678 | 0.0022148 | 0.00941775 |
| MCTP1 | 0.596431162 | 2.54E-05 | 0.00019409 |
| RGS10 | 0.596216176 | 0.00026971 | 0.00153895 |
| EHD4 | 0.594286593 | 1.79E-06 | 1.78E-05 |
| RP11-567G24 | 0.594119006 | 0.00136232 | 0.00624591 |
| ZNF880 | 0.593592146 | 0.00018657 | 0.00112092 |
| FZD5 | 0.593488305 | 0.00013081 | 0.00081841 |
| TRABD | 0.592840893 | 5.41E-09 | 8.58E-08 |
| LYN | 0.592736901 | 4.31E-08 | 5.87E-07 |
| RHOB | 0.591958299 | 1.06E-05 | 8.98E-05 |
| HEG1 | 0.591890824 | 4.95E-08 | 6.66E-07 |
| NAMPTL | 0.590753695 | 9.95E-06 | 8.46E-05 |
| SOHLH2 | 0.589984273 | 2.86E-07 | 3.37E-06 |
| RUVBL1 | 0.589341861 | 4.31E-07 | 4.91E-06 |
| CUTC | 0.588680302 | 1.16E-06 | 1.20E-05 |
| ZNF675 | 0.588625956 | 0.00209824 | 0.0090152 |
| PGK1 | 0.588200549 | 1.49E-05 | 0.00012055 |
| SLC39A4 | 0.587873724 | 0.00031975 | 0.00179035 |
| ANXA2P3 | 0.587749403 | 0.00100104 | 0.00480621 |
| DPP9 | 0.587507003 | 1.75E-05 | 0.00013924 |
| RP11-23P13. | 0.587230557 | 0.00212384 | 0.00909199 |
| SYDE1 | 0.585803004 | 3.62E-10 | 6.76E-09 |
| LARP4P | 0.585545807 | 0.00177649 | 0.00782253 |
| FAM83G | 0.5850699 | 1.28E-05 | 0.00010552 |
| CTDSP1 | -0.58503239 | 5.32E-07 | 5.91E-06 |
| MFSD11 | -0.585559059 | 2.01E-05 | 0.0001573 |
| RP11-349A22 | -0.586327639 | 1.51E-05 | 0.00012194 |
| ARPC1B | -0.586592753 | 8.09E-08 | 1.05E-06 |
| RP11-697E2. | -0.586860894 | 0.00012634 | 0.00079467 |
| ACAP3 | -0.58844186 | 2.03E-05 | 0.00015909 |
| TMEM179B | -0.589119146 | 0.00010713 | 0.00069124 |
| TNFRSF1A | -0.58964288 | 1.45E-05 | 0.00011756 |
| BDH2P1 | -0.589888558 | 0.00234789 | 0.00987914 |
| LRP11 | -0.589995034 | 0.00013905 | 0.00086209 |
| IFT74 | -0.590423282 | 6.15E-07 | 6.74E-06 |
| CUX1 | -0.590546978 | 2.30E-06 | 2.25E-05 |
| NME3 | -0.591447778 | 1.86E-07 | 2.26E-06 |
| RPS6KA3 | -0.591604222 | 5.23E-06 | 4.77E-05 |
| RNF146 | -0.592280343 | 2.43E-06 | 2.37E-05 |
| STIM2 | -0.59270958 | 1.13E-05 | 9.50E-05 |
| ITPKB | -0.592779228 | 1.25E-06 | 1.29E-05 |
| AC005789.11 | -0.592831238 | 0.00042298 | 0.00227408 |

| | | | |
|---|---|---|---|
| LBX2 | -0.593280785 | 0.00016724 | 0.00101673 |
| PREX1 | -0.593390273 | 7.18E-07 | 7.75E-06 |
| HIP1 | -0.594979863 | 2.26E-09 | 3.77E-08 |
| WAC-AS1 | -0.59538391 | 5.37E-05 | 0.00037788 |
| DDX26B | -0.595736594 | 0.00053285 | 0.0027786 |
| NBPF10 | -0.596437428 | 0.00032586 | 0.001819 |
| HEXIM1 | -0.596530711 | 1.19E-09 | 2.08E-08 |
| TBC1D4 | -0.600256019 | 8.50E-06 | 7.36E-05 |
| SFR1 | -0.60128843 | 0.00020819 | 0.00122963 |
| TPBG | -0.601450394 | 9.20E-06 | 7.86E-05 |
| ADD3 | -0.60149805 | 2.48E-09 | 4.10E-08 |
| RASA1 | -0.602535668 | 3.41E-05 | 0.00025204 |
| NMNAT1 | -0.602681429 | 0.00013861 | 0.00085967 |
| RP11-661A12 | -0.602840659 | 0.00220412 | 0.00937961 |
| DHTKD1 | -0.603407337 | 1.34E-06 | 1.37E-05 |
| BNC2 | -0.603485462 | 5.13E-07 | 5.72E-06 |
| NAPEPLD | -0.604480346 | 2.76E-06 | 2.66E-05 |
| ZMYND8 | -0.605263438 | 4.20E-10 | 7.75E-09 |
| ZNF436 | -0.605560478 | 8.51E-06 | 7.36E-05 |
| FRMD5 | -0.607362641 | 3.94E-06 | 3.67E-05 |
| CDKN1B | -0.608086462 | 0.00011332 | 0.00072496 |
| LINC00648 | -0.609293537 | 0.0002465 | 0.00142329 |
| ZKSCAN1 | -0.609483243 | 8.57E-06 | 7.39E-05 |
| HOXB7 | -0.609589315 | 9.13E-06 | 7.83E-05 |
| UGGT2 | -0.61002028 | 3.66E-05 | 0.00026837 |
| WDR91 | -0.61104224 | 5.26E-05 | 0.00037047 |
| NFATC1 | -0.611421787 | 0.00076564 | 0.00382078 |
| SSH1 | -0.611857936 | 7.12E-07 | 7.70E-06 |
| SCARB2 | -0.611986128 | 3.49E-07 | 4.03E-06 |
| MOK | -0.612826568 | 0.00195496 | 0.00849486 |
| TIAM1 | -0.613479395 | 1.40E-05 | 0.00011427 |
| SAGE1 | -0.614593188 | 8.54E-07 | 9.13E-06 |
| USP6NL | -0.614839692 | 7.86E-10 | 1.40E-08 |
| ARSB | -0.616119649 | 4.97E-07 | 5.56E-06 |
| DALRD3 | -0.61629349 | 1.16E-09 | 2.03E-08 |
| RP11-221N13 | -0.616776785 | 0.00075347 | 0.00377033 |
| OPHN1 | -0.616924473 | 0.00019739 | 0.00117515 |
| KDELC2 | -0.617919777 | 6.30E-05 | 0.00043416 |
| DECR1 | -0.617964641 | 5.17E-08 | 6.92E-07 |
| POGLUT1 | -0.619057239 | 1.43E-08 | 2.12E-07 |
| SH3BGRL | -0.619120677 | 2.67E-05 | 0.00020232 |
| MRPS6 | -0.619570258 | 3.23E-08 | 4.49E-07 |
| CEP85L | -0.619997491 | 1.77E-06 | 1.77E-05 |
| BCAP29 | -0.620617946 | 5.14E-06 | 4.69E-05 |
| CRELD1 | -0.620709596 | 1.29E-05 | 0.00010642 |
| NRBP2 | -0.620808166 | 0.0001459 | 0.00090047 |
| LRP6 | -0.62126871 | 1.04E-08 | 1.56E-07 |
| RP1-102E24.1 | -0.621551625 | 0.00016726 | 0.00101673 |
| TMEM121 | -0.621705873 | 0.00081498 | 0.00403103 |
| BAZ2B | -0.621740187 | 4.81E-06 | 4.42E-05 |
| FAM168A | -0.623067576 | 2.38E-10 | 4.53E-09 |
| SAP30L | -0.624592407 | 1.23E-08 | 1.84E-07 |
| RABL5 | -0.624823022 | 1.34E-06 | 1.37E-05 |
| CALHM2 | -0.626019777 | 0.00021394 | 0.00125999 |
| AHCYL2 | -0.626293371 | 1.47E-08 | 2.17E-07 |
| TXNDC15 | -0.626670232 | 8.48E-08 | 1.09E-06 |

| | | | |
|---|---|---|---|
| ID3 | -0.626875679 | 0.00167273 | 0.00744109 |
| ZNF513 | -0.627158543 | 1.44E-06 | 1.46E-05 |
| KCTD13 | -0.628464571 | 2.13E-05 | 0.00016575 |
| SRGAP2B | -0.628759838 | 1.62E-05 | 0.00012969 |
| WDR19 | -0.628833351 | 2.61E-05 | 0.00019869 |
| CCDC106 | -0.629484533 | 7.18E-06 | 6.35E-05 |
| OSER1-AS1 | -0.629842786 | 0.00071233 | 0.00358952 |
| STPG1 | -0.630078939 | 3.42E-05 | 0.0002525 |
| DPYSL2 | -0.63198272 | 2.13E-09 | 3.58E-08 |
| ARSA | -0.632307089 | 0.00122445 | 0.00571472 |
| SNX21 | -0.632500484 | 1.62E-06 | 1.63E-05 |
| CREM | -0.632857587 | 0.00160072 | 0.00716541 |
| ABHD2 | -0.634074524 | 3.82E-09 | 6.17E-08 |
| PELI2 | -0.634124581 | 8.18E-05 | 0.00054938 |
| DHRS1 | -0.634250629 | 4.18E-05 | 0.0003021 |
| LINC00341 | -0.634303399 | 0.00131201 | 0.00605227 |
| RPS4XP8 | -0.635347161 | 0.00021817 | 0.00128081 |
| IFT27 | -0.635568184 | 0.00018868 | 0.00113148 |
| TRPM4 | -0.636389691 | 0.0002402 | 0.00139472 |
| PGAP1 | -0.636849282 | 1.33E-05 | 0.00010938 |
| ACP2 | -0.637139476 | 2.43E-05 | 0.00018667 |
| RPL37P23 | -0.637425617 | 1.30E-06 | 1.34E-05 |
| CD276 | -0.638075987 | 8.26E-08 | 1.07E-06 |
| MCOLN3 | -0.638313323 | 0.0011705 | 0.0055012 |
| SMPD1 | -0.639085216 | 5.69E-07 | 6.28E-06 |
| SLC27A3 | -0.639321982 | 0.00042538 | 0.00228476 |
| CHSY1 | -0.639536289 | 1.79E-11 | 4.01E-10 |
| PRICKLE4 | -0.639826958 | 0.00120579 | 0.00564609 |
| DOPEY1 | -0.639971834 | 0.00026559 | 0.0015165 |
| CBX5 | -0.642060742 | 1.45E-10 | 2.83E-09 |
| LINC00205 | -0.642125805 | 0.00129396 | 0.00597908 |
| CREB3L4 | -0.642208191 | 6.48E-06 | 5.81E-05 |
| RPS4XP2 | -0.643044273 | 0.0014606 | 0.00662092 |
| ZNF558 | -0.644041163 | 0.00017642 | 0.0010661 |
| C9orf16 | -0.644264803 | 6.21E-05 | 0.00042822 |
| ZEB1 | -0.644341745 | 3.61E-08 | 4.99E-07 |
| MEGF9 | -0.644472954 | 5.84E-07 | 6.43E-06 |
| COL4A2 | -0.645286141 | 9.72E-05 | 0.00063585 |
| FBXL17 | -0.645661752 | 2.10E-08 | 3.00E-07 |
| NAGLU | -0.645758497 | 1.90E-07 | 2.31E-06 |
| ETV6 | -0.646901974 | 3.66E-09 | 5.92E-08 |
| DYRK1B | -0.647110753 | 0.0002818 | 0.00159851 |
| ARSK | -0.647362413 | 1.59E-05 | 0.00012784 |
| TSPAN31 | -0.648473669 | 7.58E-08 | 9.88E-07 |
| FAM46A | -0.649108527 | 2.56E-05 | 0.00019563 |
| KRT222 | -0.649163598 | 0.00066432 | 0.00336927 |
| ITGA5 | -0.649287141 | 8.88E-05 | 0.00059011 |
| AC003665.1 | -0.649395923 | 0.00157748 | 0.00707198 |
| GDF11 | -0.649504781 | 2.53E-11 | 5.55E-10 |
| CD44 | -0.650370882 | 2.15E-09 | 3.61E-08 |
| DLK2 | -0.651923393 | 0.00073105 | 0.00367265 |
| SUFU | -0.6530755 | 5.90E-07 | 6.49E-06 |
| FAM214B | -0.653712611 | 2.28E-07 | 2.74E-06 |
| METTL7B | -0.653793834 | 0.00024217 | 0.00140321 |
| TMPO-AS1 | -0.654149966 | 0.00065406 | 0.00332234 |
| HAPLN3 | -0.654247742 | 1.33E-05 | 0.00010938 |

| | | | |
|---|---|---|---|
| FAM102A | -0.654695428 | 1.31E-06 | 1.34E-05 |
| FNTB | -0.654698188 | 0.00029283 | 0.0016554 |
| AC009245.3 | -0.654726458 | 8.70E-05 | 0.00057991 |
| ZFP14 | -0.655165912 | 0.00055755 | 0.00289094 |
| PHLDB1 | -0.655489942 | 8.83E-09 | 1.35E-07 |
| ZNF546 | -0.655789493 | 0.00115349 | 0.00542744 |
| ARID5A | -0.657347395 | 0.00011522 | 0.00073456 |
| TNFAIP8 | -0.65769231 | 0.00035301 | 0.00194352 |
| BPHL | -0.657863107 | 0.00189948 | 0.00829316 |
| GAB1 | -0.658301028 | 9.67E-05 | 0.00063313 |
| ZNF34 | -0.658409814 | 0.00012337 | 0.00077838 |
| ZDHHC20 | -0.658757323 | 3.73E-05 | 0.00027315 |
| VEZF1P1 | -0.659425098 | 3.03E-05 | 0.00022641 |
| PTCH1 | -0.659624399 | 8.66E-07 | 9.24E-06 |
| PXK | -0.660068811 | 1.94E-09 | 3.27E-08 |
| BACE1 | -0.661673448 | 1.90E-10 | 3.65E-09 |
| TBX2 | -0.661734247 | 5.08E-06 | 4.64E-05 |
| SARNP | -0.663264753 | 0.00015086 | 0.00092765 |
| CCSAP | -0.665731372 | 7.32E-13 | 1.92E-11 |
| TANC1 | -0.665882578 | 5.61E-09 | 8.88E-08 |
| VPS36 | -0.666042839 | 3.60E-10 | 6.73E-09 |
| PHF2P2 | -0.666595299 | 0.00082529 | 0.00406792 |
| RP11-53O19. | -0.668593501 | 0.00034313 | 0.00189806 |
| RAB36 | -0.669999572 | 0.00013317 | 0.00083187 |
| RP11-212P7. | -0.671352891 | 0.00145065 | 0.0065885 |
| SPATA20 | -0.672887366 | 3.59E-08 | 4.98E-07 |
| BTN2A2 | -0.674331058 | 3.39E-05 | 0.00025067 |
| AP1S2 | -0.675028695 | 3.31E-09 | 5.38E-08 |
| DZIP3 | -0.675238595 | 3.64E-08 | 5.02E-07 |
| DAG1 | -0.675456597 | 4.24E-11 | 9.05E-10 |
| ERCC1 | -0.675598281 | 4.84E-10 | 8.86E-09 |
| SIRPAP1 | -0.675762174 | 0.00125754 | 0.00584356 |
| GLCE | -0.676419869 | 5.28E-10 | 9.62E-09 |
| TMEM67 | -0.6768389 | 0.00124264 | 0.00578414 |
| GTPBP2 | -0.676953241 | 0.00236816 | 0.00994665 |
| MPV17L | -0.676995362 | 0.00145259 | 0.00659553 |
| S100A10 | -0.677759493 | 6.52E-06 | 5.84E-05 |
| HOXA3 | -0.677800987 | 3.47E-06 | 3.26E-05 |
| SRPX2 | -0.677814026 | 2.33E-05 | 0.00018003 |
| CMTM6 | -0.677827495 | 2.71E-12 | 6.69E-11 |
| ZMYM5 | -0.67880057 | 5.03E-05 | 0.00035632 |
| PSD4 | -0.679314725 | 0.00045933 | 0.00244318 |
| TP53 | -0.679398945 | 5.12E-05 | 0.00036183 |
| ZNF521 | -0.680061274 | 1.97E-05 | 0.00015408 |
| RP11-458F8.4 | -0.680090789 | 0.00011389 | 0.00072829 |
| PMP22 | -0.680507989 | 1.30E-05 | 0.00010693 |
| NFKB1 | -0.681935061 | 0.00077474 | 0.00385686 |
| PDE4D | -0.682877114 | 7.38E-07 | 7.96E-06 |
| GSK3B | -0.683076267 | 1.67E-10 | 3.22E-09 |
| EHD3 | -0.68342463 | 1.59E-11 | 3.57E-10 |
| GPR135 | -0.683976847 | 0.00022107 | 0.00129552 |
| MMD | -0.684144257 | 7.87E-06 | 6.88E-05 |
| DCBLD2 | -0.684437015 | 2.78E-06 | 2.68E-05 |
| KIF13A | -0.685660749 | 1.92E-08 | 2.75E-07 |
| C20orf112 | -0.686760452 | 8.09E-09 | 1.24E-07 |
| VAMP1 | -0.686807375 | 0.00118407 | 0.00555229 |

| | | | |
|---|---|---|---|
| CTSZ | -0.687089928 | 4.86E-07 | 5.46E-06 |
| B4GALT6 | -0.687667292 | 3.80E-08 | 5.23E-07 |
| CELSR1 | -0.687682343 | 3.43E-07 | 3.98E-06 |
| TTC13 | -0.687747803 | 6.51E-10 | 1.17E-08 |
| ATG2B | -0.687909429 | 1.36E-09 | 2.35E-08 |
| MAP1A | -0.688030682 | 0.00173621 | 0.00766566 |
| CLHC1 | -0.688223567 | 0.00025187 | 0.00144969 |
| IRF1 | -0.689035409 | 6.34E-06 | 5.70E-05 |
| MINPP1 | -0.689728979 | 3.07E-11 | 6.65E-10 |
| ZDHHC14 | -0.690507282 | 2.43E-05 | 0.00018685 |
| SLC35B2 | -0.691918769 | 6.65E-08 | 8.75E-07 |
| ABHD8 | -0.692022616 | 1.43E-07 | 1.77E-06 |
| RBM5 | -0.692341139 | 4.25E-09 | 6.83E-08 |
| RP11-57H14. | -0.692497159 | 0.00104682 | 0.00499833 |
| C6orf1 | -0.692729223 | 4.63E-06 | 4.27E-05 |
| ABAT | -0.693048054 | 0.00076215 | 0.00380682 |
| ZNF821 | -0.693329958 | 0.00176822 | 0.00779236 |
| RP11-259N1 | -0.694325016 | 1.64E-05 | 0.0001313 |
| LEPROT | -0.694643827 | 3.24E-06 | 3.07E-05 |
| PLEKHG1 | -0.69494255 | 0.00107414 | 0.00511297 |
| FAM173B | -0.69553571 | 5.00E-05 | 0.00035472 |
| CROCCP3 | -0.695591427 | 0.00045457 | 0.00242019 |
| CCDC163P | -0.696769387 | 8.12E-05 | 0.00054581 |
| TRNP1 | -0.697909347 | 2.08E-11 | 4.61E-10 |
| FBXO27 | -0.69830168 | 0.00014018 | 0.00086788 |
| NFE2L3 | -0.698467017 | 5.09E-05 | 0.00036035 |
| CSAD | -0.699765708 | 0.00057114 | 0.00295582 |
| ST3GAL3 | -0.70058456 | 4.38E-05 | 0.00031603 |
| ABHD15 | -0.700778476 | 7.75E-07 | 8.33E-06 |
| RP11-820L6. | -0.70278233 | 0.00024794 | 0.0014306 |
| GGT7 | -0.702961285 | 2.06E-10 | 3.94E-09 |
| CNKSR2 | -0.703542237 | 0.00013555 | 0.00084486 |
| UBBP4 | -0.703649847 | 0.00046481 | 0.00246595 |
| CKAP2L | -0.704240245 | 1.53E-07 | 1.88E-06 |
| FAM114A1 | -0.705571763 | 0.00013839 | 0.000859 |
| PLA2G6 | -0.705846458 | 5.71E-05 | 0.00039826 |
| RP11-365H2 | -0.706225902 | 1.77E-08 | 2.56E-07 |
| CIRBP | -0.706534493 | 0.00017902 | 0.00107901 |
| RBPJ | -0.708595208 | 1.14E-13 | 3.41E-12 |
| FLG-AS1 | -0.708818953 | 1.85E-06 | 1.84E-05 |
| NOMO2 | -0.709128227 | 7.35E-05 | 0.00050035 |
| GPAA1 | -0.709738904 | 5.50E-13 | 1.46E-11 |
| DCPS | -0.71071436 | 4.37E-12 | 1.05E-10 |
| HDAC4 | -0.710737974 | 5.39E-05 | 0.00037846 |
| NACC2 | -0.710873084 | 1.65E-06 | 1.65E-05 |
| TCTN2 | -0.712996823 | 0.00047156 | 0.00249537 |
| FAM3C2 | -0.713753789 | 5.93E-11 | 1.24E-09 |
| AGAP2 | -0.714553358 | 0.00102679 | 0.00491552 |
| ANO8 | -0.715171917 | 4.77E-06 | 4.38E-05 |
| GABARAP | -0.715578587 | 2.75E-08 | 3.87E-07 |
| AIF1L | -0.717963855 | 0.00014012 | 0.00086788 |
| AC004980.7 | -0.718081501 | 0.00202621 | 0.00876056 |
| CLASP1 | -0.718557241 | 2.31E-06 | 2.26E-05 |
| AC091729.9 | -0.721019563 | 7.24E-05 | 0.00049302 |
| TMEM14A | -0.721307836 | 0.00061241 | 0.00313397 |
| CREBL2 | -0.722016424 | 7.12E-14 | 2.19E-12 |

| | | | |
|---|---|---|---|
| SLC12A7 | -0.722675684 | 7.91E-08 | 1.03E-06 |
| UHRF2 | -0.723530342 | 0.00215385 | 0.00919417 |
| CEP70 | -0.723663962 | 2.33E-05 | 0.00018014 |
| ETV4 | -0.723764731 | 4.48E-11 | 9.52E-10 |
| ACP6 | -0.724929718 | 1.09E-07 | 1.38E-06 |
| TOM1L2 | -0.725311227 | 1.38E-12 | 3.52E-11 |
| PCDHB7 | -0.725709917 | 6.57E-06 | 5.87E-05 |
| KCNAB2 | -0.727072601 | 3.87E-05 | 0.00028228 |
| ZFYVE28 | -0.727714369 | 0.00020049 | 0.00118931 |
| ABHD6 | -0.728557499 | 1.57E-06 | 1.59E-05 |
| POPDC3 | -0.728817317 | 1.34E-10 | 2.64E-09 |
| TMEM17 | -0.730059716 | 1.38E-06 | 1.41E-05 |
| TAPT1 | -0.730759023 | 1.67E-13 | 4.84E-12 |
| TIAM2 | -0.730820707 | 9.10E-06 | 7.80E-05 |
| KCTD15 | -0.73223443 | 8.83E-11 | 1.78E-09 |
| MYO5A | -0.732611768 | 1.57E-10 | 3.05E-09 |
| ZDHHC20P4 | -0.732794335 | 0.00023195 | 0.00135254 |
| TEF | -0.732964954 | 0.0003278 | 0.00182612 |
| AC012512.1 | -0.733558794 | 1.27E-07 | 1.58E-06 |
| RP11-343N15 | -0.734496883 | 7.59E-06 | 6.66E-05 |
| ARHGEF11 | -0.735427513 | 2.99E-09 | 4.89E-08 |
| SLCO4A1 | -0.736080497 | 1.08E-11 | 2.47E-10 |
| TBC1D19 | -0.737801819 | 0.00028137 | 0.00159664 |
| RP11-166D19 | -0.737840544 | 3.28E-07 | 3.83E-06 |
| BSCL2 | -0.738206817 | 1.45E-06 | 1.47E-05 |
| RP11-226L15 | -0.738381577 | 1.47E-05 | 0.00011885 |
| GPR137 | -0.738544531 | 1.58E-08 | 2.32E-07 |
| BHLHB9 | -0.74027552 | 0.00041187 | 0.00222232 |
| CD83 | -0.742881038 | 0.00034542 | 0.00190755 |
| PITPNM2 | -0.743138143 | 6.07E-09 | 9.56E-08 |
| SEC31B | -0.744609082 | 7.48E-05 | 0.00050746 |
| LHFPL2 | -0.745017205 | 1.79E-09 | 3.03E-08 |
| VPS37D | -0.745939723 | 0.00053015 | 0.00276539 |
| PRKACB | -0.74610221 | 7.55E-15 | 2.56E-13 |
| ZDHHC13 | -0.7461318 | 3.61E-07 | 4.16E-06 |
| HPS5 | -0.746594005 | 0.00014319 | 0.00088478 |
| TIMP1 | -0.746845512 | 1.73E-08 | 2.51E-07 |
| RMDN2 | -0.7481103 | 1.34E-05 | 0.00010951 |
| DNM1 | -0.748593278 | 9.22E-10 | 1.63E-08 |
| SLC44A1 | -0.749726391 | 9.90E-14 | 2.99E-12 |
| CAPN5 | -0.750277373 | 3.13E-10 | 5.89E-09 |
| FAM3C | -0.75154722 | 2.29E-07 | 2.75E-06 |
| SESTD1 | -0.751784507 | 6.75E-08 | 8.86E-07 |
| CCDC28B | -0.751807377 | 1.04E-06 | 1.09E-05 |
| CLDN4 | -0.752103662 | 0.00074106 | 0.00371839 |
| NET1 | -0.752538395 | 8.47E-05 | 0.00056651 |
| SLC22A4 | -0.754086118 | 0.00090254 | 0.00439606 |
| AF013593.1 | -0.755325378 | 0.0007275 | 0.00365593 |
| RP11-46C24. | -0.756287023 | 2.71E-06 | 2.62E-05 |
| FOXD2 | -0.757172786 | 9.19E-06 | 7.86E-05 |
| TMEM198 | -0.758289278 | 0.00024505 | 0.00141587 |
| SUSD5 | -0.759475253 | 1.03E-09 | 1.81E-08 |
| WNK4 | -0.759633737 | 4.81E-11 | 1.02E-09 |
| FOXF1 | -0.760630003 | 0.00016512 | 0.00100631 |
| GABRA3 | -0.760721934 | 4.99E-05 | 0.00035467 |
| ANK3 | -0.76074101 | 0.00011462 | 0.00073184 |

| | | | |
|---|---|---|---|
| NRG2 | -0.76083759 | 9.89E-05 | 0.00064491 |
| RARG | -0.76093094 | 1.34E-10 | 2.64E-09 |
| UPP1 | -0.761026586 | 3.20E-07 | 3.75E-06 |
| SEMA4C | -0.763073733 | 4.87E-11 | 1.03E-09 |
| IFI35 | -0.763155619 | 5.72E-05 | 0.00039863 |
| MTHFD2L | -0.763737973 | 4.64E-06 | 4.27E-05 |
| UGT2B7 | -0.764521238 | 0.00103406 | 0.00494169 |
| DNAJC4 | -0.764595792 | 2.96E-05 | 0.00022161 |
| KIAA0513 | -0.764758208 | 4.29E-07 | 4.89E-06 |
| SLC16A9 | -0.766181333 | 0.00041599 | 0.00224236 |
| CTC-228N24. | -0.766626685 | 9.97E-09 | 1.51E-07 |
| SLC9A7P1 | -0.768174514 | 0.00011564 | 0.00073692 |
| ING4 | -0.770358851 | 5.78E-10 | 1.05E-08 |
| ADAM23 | -0.771332166 | 1.59E-05 | 0.00012756 |
| TGFBR2 | -0.771839547 | 6.93E-07 | 7.52E-06 |
| CCDC24 | -0.773021315 | 0.00023388 | 0.00136232 |
| PCDH10 | -0.773366012 | 0.00195474 | 0.00849486 |
| FRMD4A | -0.773854438 | 3.25E-11 | 7.02E-10 |
| ARL6IP5 | -0.774370271 | 1.30E-06 | 1.34E-05 |
| SGCB | -0.774739435 | 2.39E-11 | 5.27E-10 |
| IGFBP7 | -0.776574955 | 6.59E-09 | 1.03E-07 |
| RP11-216N14 | -0.777207591 | 9.92E-06 | 8.43E-05 |
| IFT122 | -0.777981727 | 2.00E-08 | 2.87E-07 |
| ATP1A1 | -0.778653559 | 1.63E-10 | 3.15E-09 |
| PI15 | -0.779164233 | 0.00072246 | 0.0036317 |
| GXYLT2 | -0.779171147 | 3.45E-06 | 3.25E-05 |
| ARHGAP33 | -0.779462021 | 0.00082078 | 0.00405053 |
| EHBP1 | -0.779723868 | 4.03E-15 | 1.41E-13 |
| FAM89B | -0.779850375 | 7.10E-08 | 9.29E-07 |
| ENDOD1 | -0.780648189 | 2.64E-11 | 5.79E-10 |
| NID1 | -0.780695452 | 0.00049809 | 0.00261139 |
| EMILIN1 | -0.781411445 | 1.97E-07 | 2.39E-06 |
| CUEDC1 | -0.781601411 | 5.90E-15 | 2.05E-13 |
| MCL1 | -0.78224849 | 1.25E-05 | 0.00010349 |
| DPY19L1 | -0.782383095 | 3.85E-14 | 1.23E-12 |
| LMLN | -0.782763682 | 1.40E-05 | 0.00011366 |
| IFT88 | -0.785567494 | 1.49E-07 | 1.84E-06 |
| SUMF1 | -0.786883628 | 6.40E-09 | 1.00E-07 |
| SMAD6 | -0.787602998 | 4.63E-05 | 0.00033202 |
| HFE | -0.787833013 | 2.86E-05 | 0.00021521 |
| CALB2 | -0.788856008 | 0.00228393 | 0.00965934 |
| AL109763.2 | -0.789001715 | 0.0009595 | 0.00463687 |
| SORBS1 | -0.789917586 | 0.00163976 | 0.0073143 |
| TAPT1-AS1 | -0.79013302 | 7.70E-05 | 0.00052097 |
| SPATS2L | -0.791320443 | 2.78E-07 | 3.29E-06 |
| FAM43A | -0.791819308 | 2.75E-06 | 2.65E-05 |
| MARCKSL1 | -0.792062097 | 6.04E-10 | 1.09E-08 |
| TESK2 | -0.792412254 | 6.90E-06 | 6.13E-05 |
| RP11-196G18 | -0.792439848 | 3.00E-06 | 2.87E-05 |
| KIAA1324L | -0.792835617 | 2.48E-11 | 5.46E-10 |
| IDUA | -0.79290986 | 1.79E-06 | 1.78E-05 |
| SLC4A3 | -0.794158271 | 1.57E-07 | 1.93E-06 |
| CCT6P3 | -0.794759807 | 0.00075028 | 0.0037578 |
| PLIN2 | -0.794903071 | 4.09E-08 | 5.58E-07 |
| RABL2A | -0.795147014 | 4.68E-05 | 0.00033505 |
| CCNJL | -0.795605275 | 4.76E-13 | 1.28E-11 |

| | | | |
|---|---|---|---|
| LPXN | -0.796357408 | 0.00070109 | 0.00353829 |
| TGFBR3 | -0.797359455 | 0.00114713 | 0.0054037 |
| PDK2 | -0.798262993 | 4.44E-08 | 6.03E-07 |
| SLC16A4 | -0.798941494 | 4.50E-06 | 4.16E-05 |
| PDLIM4 | -0.799112769 | 9.07E-09 | 1.38E-07 |
| WWP2 | -0.799373754 | 2.01E-07 | 2.44E-06 |
| ALAD | -0.800189845 | 3.09E-15 | 1.10E-13 |
| SSFA2 | -0.803724555 | 0.00015792 | 0.00096637 |
| BICD1 | -0.803820483 | 3.99E-18 | 1.96E-16 |
| CYB5D2 | -0.804476494 | 0.00093221 | 0.00452053 |
| TTLL3 | -0.804500387 | 0.0002096 | 0.00123728 |
| COL6A2 | -0.804670824 | 0.0004306 | 0.00231049 |
| NT5E | -0.805092989 | 8.19E-06 | 7.13E-05 |
| LRRC73 | -0.80556497 | 0.0001169 | 0.00074321 |
| CCNG2 | -0.80623197 | 0.00016519 | 0.00100635 |
| RP11-18H7.1 | -0.806274639 | 0.00090078 | 0.00438879 |
| SIK2 | -0.806675348 | 7.04E-17 | 3.01E-15 |
| TCP11L2 | -0.806753837 | 0.00233255 | 0.00983469 |
| PLCB1 | -0.807837706 | 6.04E-08 | 8.00E-07 |
| CACNA2D1 | -0.808022462 | 3.92E-08 | 5.38E-07 |
| THBS3 | -0.808622508 | 0.00018811 | 0.00112925 |
| ANTXR2 | -0.810513513 | 2.41E-10 | 4.58E-09 |
| HPCAL1 | -0.811560729 | 3.02E-11 | 6.57E-10 |
| ADAM12 | -0.812189568 | 0.00044467 | 0.00237439 |
| OSBPL5 | -0.812696006 | 2.04E-05 | 0.0001592 |
| APOBEC3G | -0.813302685 | 7.64E-05 | 0.00051704 |
| ANGPTL2 | -0.814387811 | 2.03E-13 | 5.83E-12 |
| HOTAIR | -0.814579017 | 0.00077245 | 0.00384659 |
| RP5-1024G6. | -0.814677219 | 2.52E-07 | 3.00E-06 |
| PKD2 | -0.819786603 | 4.08E-13 | 1.11E-11 |
| LMNA | -0.819806036 | 2.43E-11 | 5.35E-10 |
| CPS1 | -0.82020816 | 1.24E-06 | 1.29E-05 |
| FZD9 | -0.820425068 | 0.00046604 | 0.00247021 |
| GNE | -0.820578082 | 2.43E-07 | 2.90E-06 |
| RP11-445F12 | -0.82081599 | 0.00227794 | 0.00963649 |
| YPEL2 | -0.823857829 | 0.00017438 | 0.0010561 |
| HMGA2 | -0.823975137 | 0.00094248 | 0.00456361 |
| SAT1 | -0.823975506 | 8.64E-06 | 7.45E-05 |
| LAMC1 | -0.82452264 | 1.86E-12 | 4.67E-11 |
| ELOVL4 | -0.826139142 | 2.38E-08 | 3.38E-07 |
| RP11-603J24 | -0.82729077 | 8.55E-15 | 2.88E-13 |
| SLC27A1 | -0.830520237 | 0.00048721 | 0.00256742 |
| AC074212.6 | -0.832534676 | 0.00072236 | 0.0036317 |
| ZNF517 | -0.832660581 | 9.20E-05 | 0.0006069 |
| PBXIP1 | -0.833374592 | 2.64E-13 | 7.40E-12 |
| TTC30B | -0.833552509 | 9.18E-06 | 7.85E-05 |
| SERPINB8 | -0.83643028 | 7.54E-12 | 1.74E-10 |
| TCTN1 | -0.836476613 | 9.92E-14 | 2.99E-12 |
| LGALS1 | -0.837319434 | 2.77E-08 | 3.91E-07 |
| GALNT5 | -0.838072618 | 0.00010103 | 0.0006568 |
| KIAA1755 | -0.8381537 | 1.17E-05 | 9.75E-05 |
| PRR4 | -0.839243606 | 1.62E-06 | 1.63E-05 |
| ARHGEF37 | -0.839377911 | 0.00070075 | 0.0035382 |
| RP11-541N1( | -0.840206861 | 0.0002407 | 0.00139664 |
| SOCS6 | -0.840719684 | 5.16E-12 | 1.23E-10 |
| ADAM8 | -0.841357043 | 0.00020233 | 0.00119892 |

| | | | |
|---|---|---|---|
| PLCD1 | -0.841717396 | 0.00018917 | 0.00113401 |
| CRISPLD1 | -0.842165155 | 0.0004818 | 0.00254185 |
| PAQR4 | -0.844197869 | 7.33E-14 | 2.25E-12 |
| ZBTB38 | -0.84431511 | 1.49E-07 | 1.84E-06 |
| CMPK2 | -0.845690966 | 0.00078664 | 0.00390663 |
| TAPBPL | -0.845839628 | 1.86E-05 | 0.00014657 |
| LUCAT1 | -0.847011873 | 0.00128717 | 0.00595269 |
| ALDH3A2 | -0.847255285 | 1.57E-11 | 3.53E-10 |
| PLA2R1 | -0.847324997 | 0.00065351 | 0.00332085 |
| MOCOS | -0.847415547 | 2.92E-05 | 0.00021918 |
| ABTB1 | -0.847571281 | 7.69E-12 | 1.78E-10 |
| PSKH1 | -0.848405881 | 8.89E-09 | 1.36E-07 |
| RAB13 | -0.848475772 | 2.58E-15 | 9.26E-14 |
| PCDHGA1 | -0.84878581 | 0.00015399 | 0.00094491 |
| GLB1L | -0.850969122 | 7.90E-05 | 0.00053338 |
| SASH1 | -0.851163773 | 9.94E-14 | 2.99E-12 |
| CCDC159 | -0.854299391 | 0.00011675 | 0.00074253 |
| SPATA17 | -0.855288335 | 0.00074472 | 0.00373219 |
| ACVR1 | -0.855316814 | 1.06E-12 | 2.76E-11 |
| ERMAP | -0.855438267 | 1.37E-06 | 1.39E-05 |
| RAB38 | -0.856708707 | 1.07E-05 | 8.99E-05 |
| PIR | -0.8578433 | 1.04E-09 | 1.84E-08 |
| TNS3 | -0.861369012 | 1.01E-06 | 1.06E-05 |
| AAMDC | -0.862112699 | 0.00025789 | 0.00147613 |
| LYST | -0.862762023 | 8.69E-05 | 0.00057965 |
| APLP2 | -0.863521111 | 2.47E-13 | 7.00E-12 |
| BCAS3 | -0.8646533 | 6.39E-11 | 1.33E-09 |
| RP11-479G22 | -0.866769284 | 0.00086187 | 0.00422172 |
| SEZ6L2 | -0.867430222 | 1.99E-06 | 1.97E-05 |
| HGSNAT | -0.868422609 | 1.61E-06 | 1.62E-05 |
| GNG4 | -0.869787099 | 3.46E-07 | 4.01E-06 |
| KLHL22 | -0.87154065 | 1.00E-05 | 8.49E-05 |
| CA5B | -0.871702635 | 6.20E-05 | 0.00042774 |
| FGFR3 | -0.872362744 | 0.00012009 | 0.00076029 |
| ERVK13-1 | -0.872667198 | 8.51E-07 | 9.11E-06 |
| LMBRD1 | -0.873214825 | 8.83E-07 | 9.41E-06 |
| CABLES2 | -0.874200223 | 3.03E-09 | 4.95E-08 |
| CEP19 | -0.875175469 | 0.00063312 | 0.00323091 |
| CNR1 | -0.87570591 | 3.85E-10 | 7.18E-09 |
| RP3-368A4.5 | -0.876131957 | 0.00012792 | 0.00080309 |
| PRKAR1A | -0.87620737 | 5.65E-12 | 1.33E-10 |
| CCDC121 | -0.87818022 | 0.00027884 | 0.0015828 |
| EPS8 | -0.87830352 | 2.78E-09 | 4.58E-08 |
| PCYOX1L | -0.878604447 | 8.03E-08 | 1.04E-06 |
| MMP14 | -0.879608465 | 4.05E-08 | 5.54E-07 |
| PAN3-AS1 | -0.879871314 | 0.00140544 | 0.006415 |
| BAIAP2-AS1 | -0.880300966 | 1.87E-07 | 2.28E-06 |
| ID1 | -0.880813538 | 1.85E-08 | 2.65E-07 |
| DLGAP1-AS1 | -0.880975544 | 2.83E-05 | 0.00021298 |
| NDE1 | -0.881383491 | 2.10E-15 | 7.59E-14 |
| FAM107B | -0.88191492 | 1.48E-06 | 1.50E-05 |
| SRGAP2 | -0.883181256 | 1.12E-16 | 4.73E-15 |
| TMEM158 | -0.883618713 | 1.69E-06 | 1.69E-05 |
| FMNL3 | -0.884423582 | 4.57E-07 | 5.17E-06 |
| PAG1 | -0.884715515 | 0.00034016 | 0.00188794 |
| GALNT2 | -0.88553794 | 2.55E-10 | 4.83E-09 |

| | | | |
|---|---|---|---|
| VIM | -0.885674653 | 2.72E-12 | 6.69E-11 |
| PC | -0.887229033 | 7.79E-11 | 1.59E-09 |
| VWA5A | -0.887785953 | 0.00102968 | 0.00492791 |
| NPTXR | -0.892055639 | 0.00015401 | 0.00094491 |
| PHC2 | -0.89231973 | 1.69E-13 | 4.90E-12 |
| PRKAR1AP | -0.892613646 | 1.78E-08 | 2.58E-07 |
| C11orf35 | -0.894066985 | 0.0011271 | 0.00532914 |
| MYLIP | -0.896190425 | 0.00060214 | 0.00308717 |
| BMP6 | -0.896489434 | 0.00040846 | 0.00220812 |
| RP11-152P17 | -0.896859416 | 0.00033209 | 0.00184876 |
| LRRC29 | -0.897142099 | 0.00186611 | 0.00815828 |
| IFT80 | -0.899125671 | 4.39E-09 | 7.03E-08 |
| HAS3 | -0.899851763 | 2.82E-05 | 0.00021252 |
| TRIOBP | -0.90047839 | 6.40E-08 | 8.45E-07 |
| FAM184A | -0.900719287 | 3.56E-07 | 4.11E-06 |
| C6orf141 | -0.900967234 | 0.00127962 | 0.00592612 |
| SNRPB2 | -0.901844022 | 2.64E-13 | 7.40E-12 |
| H1FX-AS1 | -0.902465048 | 4.60E-05 | 0.00032939 |
| GPC6 | -0.902740555 | 5.35E-05 | 0.00037662 |
| AP001372.2 | -0.902963328 | 4.46E-06 | 4.12E-05 |
| TSHZ3 | -0.903198213 | 6.96E-06 | 6.17E-05 |
| RP11-396K3. | -0.903541217 | 0.00020205 | 0.00119767 |
| CTA-246H3.1 | -0.907811848 | 0.00035348 | 0.00194486 |
| LSR | -0.909384083 | 0.00024812 | 0.00143113 |
| NOVA1 | -0.909880024 | 1.73E-07 | 2.12E-06 |
| EVA1C | -0.910619175 | 1.08E-06 | 1.13E-05 |
| PRNP | -0.911306794 | 5.14E-15 | 1.79E-13 |
| ZNF117 | -0.911666752 | 1.15E-07 | 1.44E-06 |
| H6PD | -0.912278524 | 0.00011816 | 0.00075037 |
| ZNF75D | -0.912417665 | 0.00014544 | 0.00089798 |
| FGFRL1 | -0.912845528 | 5.30E-12 | 1.26E-10 |
| C11orf80 | -0.913555492 | 3.13E-05 | 0.00023398 |
| EFCAB13 | -0.914898679 | 9.32E-06 | 7.96E-05 |
| POU3F2 | -0.915421143 | 1.28E-09 | 2.22E-08 |
| NQO1 | -0.915591901 | 4.17E-16 | 1.66E-14 |
| BTG1 | -0.91663637 | 5.16E-10 | 9.40E-09 |
| BBS12 | -0.917484139 | 0.00020415 | 0.00120855 |
| TTC28 | -0.918308026 | 6.94E-05 | 0.00047415 |
| HMG20B | -0.921406343 | 9.05E-15 | 3.04E-13 |
| KYNU | -0.92152728 | 3.73E-07 | 4.29E-06 |
| ABR | -0.922064776 | 1.37E-17 | 6.24E-16 |
| GAREM | -0.922083704 | 2.89E-05 | 0.00021692 |
| QPCT | -0.922420049 | 1.11E-05 | 9.31E-05 |
| NAB2 | -0.922660591 | 4.69E-13 | 1.27E-11 |
| GFPT2 | -0.923287635 | 3.69E-11 | 7.91E-10 |
| LGR4 | -0.923897314 | 0.00233639 | 0.00984583 |
| MYO1E | -0.924739838 | 1.87E-08 | 2.69E-07 |
| FARP1 | -0.925188646 | 1.38E-10 | 2.71E-09 |
| C9orf89 | -0.925833437 | 1.25E-09 | 2.17E-08 |
| CCDC40 | -0.928271905 | 0.00034248 | 0.00189638 |
| LRRC46 | -0.92994543 | 0.00011276 | 0.0007222 |
| DUSP6 | -0.930683506 | 6.50E-06 | 5.82E-05 |
| IRF2BPL | -0.931330359 | 8.02E-06 | 7.01E-05 |
| FAM222A | -0.931510053 | 1.57E-05 | 0.00012617 |
| CPT1A | -0.931670184 | 5.55E-08 | 7.38E-07 |
| DUSP4 | -0.93290326 | 2.62E-18 | 1.31E-16 |

| | | | |
|---|---|---|---|
| CTD-2044J15 | -0.933150655 | 0.00014871 | 0.00091647 |
| STK17A | -0.936215877 | 1.74E-10 | 3.35E-09 |
| RP11-37B2.1 | -0.937456363 | 1.49E-07 | 1.84E-06 |
| PHF10 | -0.938387386 | 4.12E-09 | 6.62E-08 |
| IL11RA | -0.938947608 | 4.76E-08 | 6.42E-07 |
| VMAC | -0.943545963 | 1.31E-06 | 1.34E-05 |
| SOS2 | -0.944900428 | 1.72E-11 | 3.86E-10 |
| LTBP3 | -0.945689174 | 6.66E-16 | 2.59E-14 |
| MBNL3 | -0.946193633 | 3.20E-08 | 4.46E-07 |
| PPFIBP1 | -0.948135443 | 3.43E-17 | 1.49E-15 |
| LRRC48 | -0.948223023 | 1.48E-06 | 1.50E-05 |
| ITFG3 | -0.949770055 | 1.11E-11 | 2.53E-10 |
| LMCD1 | -0.951125012 | 1.24E-10 | 2.45E-09 |
| MFSD12 | -0.951181271 | 1.02E-17 | 4.72E-16 |
| USP46-AS1 | -0.953103357 | 8.54E-06 | 7.38E-05 |
| HBP1 | -0.953930911 | 2.28E-08 | 3.25E-07 |
| UST | -0.956677446 | 2.58E-07 | 3.07E-06 |
| DLG3 | -0.956855997 | 2.27E-11 | 5.02E-10 |
| RP5-882C2.2 | -0.956954249 | 0.00047243 | 0.00249835 |
| PLEKHH2 | -0.958220161 | 6.66E-07 | 7.25E-06 |
| MEGF8 | -0.958421788 | 3.99E-10 | 7.41E-09 |
| SNX10 | -0.958916265 | 8.22E-05 | 0.00055155 |
| CYP27A1 | -0.960175716 | 2.14E-05 | 0.00016648 |
| EMP2 | -0.965120839 | 3.95E-10 | 7.34E-09 |
| EFNB2 | -0.966087929 | 1.13E-05 | 9.45E-05 |
| CDC42EP3 | -0.966423139 | 3.16E-16 | 1.27E-14 |
| RGS17 | -0.966727776 | 5.12E-06 | 4.68E-05 |
| SEMA3A | -0.966810471 | 1.74E-09 | 2.95E-08 |
| RP11-277P12 | -0.96731693 | 0.00138508 | 0.00633439 |
| CRLF1 | -0.968790875 | 1.29E-06 | 1.33E-05 |
| S100A4 | -0.969801505 | 0.00019183 | 0.0011466 |
| ITGAV | -0.97047427 | 3.92E-12 | 9.48E-11 |
| B3GNT5 | -0.970841511 | 1.74E-07 | 2.13E-06 |
| ROM1 | -0.97140654 | 0.00011436 | 0.00073043 |
| NBEA | -0.972222221 | 4.18E-08 | 5.70E-07 |
| CDK19 | -0.972420397 | 1.36E-11 | 3.09E-10 |
| FMNL2 | -0.973034212 | 2.51E-13 | 7.07E-12 |
| THSD4 | -0.973484211 | 8.35E-07 | 8.95E-06 |
| ARHGAP5 | -0.974018518 | 8.41E-18 | 3.93E-16 |
| AC010904.1 | -0.974140941 | 2.37E-07 | 2.83E-06 |
| ARHGEF9 | -0.97420946 | 0.00175006 | 0.00772062 |
| CNIH3 | -0.974953154 | 0.00013852 | 0.00085947 |
| RP13-228J13 | -0.975052726 | 5.96E-05 | 0.00041436 |
| LINC00963 | -0.975264024 | 2.90E-08 | 4.05E-07 |
| CDKN2C | -0.975299082 | 0.00010381 | 0.00067296 |
| ETV5 | -0.976335832 | 1.62E-12 | 4.11E-11 |
| AQP3 | -0.97658643 | 0.00027795 | 0.00157882 |
| RCBTB2 | -0.98132057 | 7.69E-07 | 8.27E-06 |
| ARHGEF6 | -0.982319136 | 1.00E-05 | 8.49E-05 |
| ACSL3 | -0.984091614 | 3.58E-18 | 1.77E-16 |
| ATP7A | -0.985728347 | 3.35E-11 | 7.22E-10 |
| CTA-217C2.1 | -0.986151137 | 5.84E-05 | 0.0004067 |
| GNAS | -0.987196104 | 6.47E-12 | 1.51E-10 |
| ST6GALNAC3 | -0.988119399 | 1.11E-07 | 1.40E-06 |
| IL22RA1 | -0.989220316 | 6.29E-05 | 0.00043377 |
| CDH12 | -0.989225074 | 8.93E-10 | 1.58E-08 |

| | | | |
|---|---|---|---|
| KALRN | -0.990289232 | 0.00081596 | 0.00403277 |
| TMEM164 | -0.990765771 | 6.02E-12 | 1.41E-10 |
| MRC2 | -0.991057908 | 3.74E-11 | 8.00E-10 |
| TTLL7 | -0.991071892 | 1.30E-05 | 0.00010693 |
| FHDC1 | -0.991342678 | 9.26E-05 | 0.00061003 |
| FRMD3 | -0.99392781 | 0.00137014 | 0.00627826 |
| RP11-108P20 | -0.994744221 | 1.23E-05 | 0.00010197 |
| S100A6 | -0.99566465 | 4.14E-16 | 1.65E-14 |
| SALL1 | -0.995882007 | 8.75E-06 | 7.52E-05 |
| RP11-496I9.1 | -0.995962771 | 0.00200116 | 0.00865906 |
| FAXDC2 | -0.996123902 | 4.81E-07 | 5.42E-06 |
| COL16A1 | -0.996666261 | 1.75E-09 | 2.96E-08 |
| CXXC5 | -0.997439708 | 3.41E-10 | 6.39E-09 |
| C2orf81 | -0.999222663 | 2.18E-08 | 3.11E-07 |
| SYNJ2 | -1.000147359 | 0.00026757 | 0.00152726 |
| TBC1D8 | -1.002963481 | 1.52E-11 | 3.44E-10 |
| SFT2D2 | -1.004744654 | 6.20E-19 | 3.22E-17 |
| SCN1B | -1.005510618 | 1.60E-05 | 0.00012817 |
| LNX1 | -1.009834688 | 4.41E-07 | 5.01E-06 |
| KIAA1549L | -1.011291172 | 7.08E-05 | 0.00048284 |
| MUC15 | -1.014504708 | 0.00020403 | 0.00120854 |
| SHISA4 | -1.014929036 | 6.42E-08 | 8.48E-07 |
| MXD3 | -1.015038895 | 4.33E-08 | 5.89E-07 |
| PRKCDBP | -1.016151027 | 4.17E-10 | 7.70E-09 |
| BMP8B | -1.016834503 | 6.01E-12 | 1.41E-10 |
| CRYL1 | -1.017059451 | 0.00234561 | 0.00987206 |
| ETFB | -1.019298232 | 4.13E-19 | 2.18E-17 |
| GPT | -1.021383005 | 5.41E-05 | 0.00038014 |
| SEMA3C | -1.02167646 | 3.90E-10 | 7.26E-09 |
| SAMD14 | -1.021810193 | 6.67E-06 | 5.95E-05 |
| TPT1-AS1 | -1.023169991 | 0.00149275 | 0.00674256 |
| ST6GAL1 | -1.023978378 | 9.48E-12 | 2.18E-10 |
| RP11-549B18 | -1.027310196 | 5.63E-05 | 0.00039358 |
| DLX2 | -1.027616338 | 0.00077634 | 0.00386131 |
| RP11-477D19 | -1.027702987 | 2.72E-08 | 3.83E-07 |
| AGA | -1.028288554 | 2.04E-10 | 3.90E-09 |
| TLE2 | -1.029657985 | 0.0008932 | 0.00436094 |
| ICK | -1.030214986 | 8.74E-06 | 7.52E-05 |
| DIXDC1 | -1.030608934 | 3.71E-07 | 4.27E-06 |
| SNTB2 | -1.030839647 | 4.82E-18 | 2.34E-16 |
| SLFN5 | -1.031095316 | 6.64E-09 | 1.04E-07 |
| AHRR | -1.031250657 | 1.53E-12 | 3.88E-11 |
| RAP1GAP | -1.031306735 | 3.02E-05 | 0.00022599 |
| ZNF385A | -1.032338956 | 8.81E-16 | 3.34E-14 |
| CNTRL | -1.032984532 | 2.26E-14 | 7.32E-13 |
| ARSE | -1.034145438 | 2.77E-07 | 3.27E-06 |
| IFNAR2 | -1.035532068 | 2.66E-10 | 5.02E-09 |
| TBX19 | -1.035565436 | 3.55E-07 | 4.10E-06 |
| CADM1 | -1.037075063 | 8.19E-10 | 1.46E-08 |
| LAMA2 | -1.039748921 | 0.00058331 | 0.00300841 |
| SDHAP3 | -1.041766419 | 0.00103263 | 0.0049363 |
| JADE2 | -1.042233715 | 1.35E-13 | 3.98E-12 |
| CNP | -1.042239395 | 6.07E-22 | 4.42E-20 |
| RBM47 | -1.042459152 | 0.00011899 | 0.00075444 |
| RP11-551L14 | -1.043062298 | 0.00079833 | 0.00395874 |
| PPP4R4 | -1.043241807 | 0.000339 | 0.00188217 |

| | | | |
|---|---|---|---|
| ACSS2 | -1.044600803 | 5.31E-05 | 0.00037411 |
| STK32B | -1.049595554 | 5.30E-12 | 1.26E-10 |
| SH3PXD2A | -1.050153621 | 3.01E-15 | 1.07E-13 |
| KAT2B | -1.050406117 | 0.00018658 | 0.00112092 |
| ZNF235 | -1.051882704 | 6.73E-07 | 7.32E-06 |
| KCNIP3 | -1.05407733 | 9.53E-07 | 1.01E-05 |
| TGFB3 | -1.054102524 | 3.63E-06 | 3.40E-05 |
| RPGR | -1.056237212 | 3.97E-09 | 6.40E-08 |
| RHPN1-AS1 | -1.056655946 | 1.00E-07 | 1.28E-06 |
| SPATA13 | -1.057056044 | 1.65E-13 | 4.81E-12 |
| PRKG1 | -1.057738651 | 2.21E-05 | 0.00017194 |
| PDE9A | -1.060120137 | 3.44E-06 | 3.24E-05 |
| SFMBT2 | -1.061964444 | 6.37E-08 | 8.43E-07 |
| CYP1B1 | -1.06210693 | 2.40E-05 | 0.00018496 |
| RP11-50E11.: | -1.062505474 | 0.00051296 | 0.0026834 |
| PLAT | -1.062528009 | 7.79E-18 | 3.66E-16 |
| RP11-244O1( | -1.063530157 | 4.27E-07 | 4.88E-06 |
| ARHGAP26 | -1.064129402 | 2.89E-13 | 8.04E-12 |
| CPE | -1.066531449 | 6.57E-07 | 7.18E-06 |
| CAT | -1.067673292 | 4.66E-25 | 4.39E-23 |
| CAMK4 | -1.068400299 | 1.25E-07 | 1.56E-06 |
| RP11-359B12 | -1.068533544 | 0.00145508 | 0.00660317 |
| TP73 | -1.068915235 | 6.16E-11 | 1.28E-09 |
| ST3GAL4 | -1.069851645 | 3.16E-17 | 1.38E-15 |
| FAM155A | -1.072892733 | 1.70E-08 | 2.47E-07 |
| MYH15 | -1.07303246 | 0.00137073 | 0.00627922 |
| NIPAL3 | -1.073644921 | 2.19E-16 | 8.98E-15 |
| NCOA7 | -1.073753657 | 0.00034501 | 0.00190666 |
| RP11-282O18 | -1.073945432 | 7.42E-08 | 9.69E-07 |
| WNT5A | -1.074533855 | 3.83E-06 | 3.57E-05 |
| TRIB1 | -1.075015518 | 0.00016318 | 0.00099592 |
| PIP5KL1 | -1.07606845 | 0.00041994 | 0.00225992 |
| ICOSLG | -1.076379602 | 3.97E-08 | 5.44E-07 |
| PCDH9 | -1.078355576 | 1.56E-12 | 3.97E-11 |
| PIK3R3 | -1.078783197 | 2.49E-09 | 4.11E-08 |
| TLR6 | -1.080018936 | 1.58E-10 | 3.07E-09 |
| SEMA3B | -1.080054182 | 1.85E-11 | 4.13E-10 |
| GPR110 | -1.084408469 | 0.00113896 | 0.00537906 |
| OSBPL10 | -1.08446563 | 4.16E-15 | 1.46E-13 |
| MFI2-AS1 | -1.087143859 | 5.42E-07 | 6.00E-06 |
| PHYHIP | -1.08728855 | 5.51E-06 | 4.99E-05 |
| RCAN3 | -1.089669156 | 4.62E-10 | 8.48E-09 |
| NRP2 | -1.090960121 | 1.22E-13 | 3.64E-12 |
| CDK2AP1 | -1.090990606 | 1.86E-24 | 1.69E-22 |
| LHX1 | -1.091194981 | 0.0001764 | 0.0010661 |
| PLD1 | -1.091718744 | 1.68E-08 | 2.45E-07 |
| ZNF827 | -1.093666302 | 6.85E-26 | 7.06E-24 |
| GULP1 | -1.093743515 | 2.65E-21 | 1.81E-19 |
| GPR37 | -1.094356213 | 0.00198695 | 0.00861341 |
| PGAP3 | -1.094772468 | 6.38E-08 | 8.44E-07 |
| SULF2 | -1.094805723 | 1.49E-09 | 2.56E-08 |
| DOCK4 | -1.097654154 | 2.06E-13 | 5.92E-12 |
| FAM46B | -1.097888817 | 0.00076911 | 0.00383344 |
| CMTM7 | -1.098829023 | 1.13E-15 | 4.26E-14 |
| SLC5A3 | -1.09947016 | 1.95E-05 | 0.00015287 |
| WBP1 | -1.102430305 | 2.38E-09 | 3.94E-08 |

| | | | |
|---|---|---|---|
| ABCA1 | -1.106156544 | 2.66E-08 | 3.75E-07 |
| SPARC | -1.108258024 | 2.68E-12 | 6.63E-11 |
| S100A13 | -1.10969994 | 2.10E-19 | 1.16E-17 |
| SYNE2 | -1.113708388 | 6.47E-08 | 8.52E-07 |
| STON1 | -1.116378975 | 9.35E-05 | 0.0006152 |
| LINC00886 | -1.117490764 | 4.54E-05 | 0.00032608 |
| DISP1 | -1.120062322 | 2.04E-13 | 5.86E-12 |
| UBE2F | -1.122572017 | 3.76E-13 | 1.03E-11 |
| DUSP10 | -1.124956853 | 5.77E-08 | 7.66E-07 |
| SLCO3A1 | -1.126376615 | 1.14E-12 | 2.95E-11 |
| RP11-284F21 | -1.126723632 | 3.17E-12 | 7.76E-11 |
| LINC00622 | -1.127116752 | 0.00013335 | 0.00083255 |
| PLXNB3 | -1.129206994 | 1.40E-10 | 2.75E-09 |
| C9orf3 | -1.131074487 | 6.07E-07 | 6.67E-06 |
| HIPK2 | -1.134280418 | 1.33E-13 | 3.93E-12 |
| LYPD3 | -1.134796553 | 7.45E-05 | 0.00050612 |
| GOLGA7B | -1.136225609 | 0.00211654 | 0.00907253 |
| PPARG | -1.136541711 | 2.10E-14 | 6.82E-13 |
| DMRTA1 | -1.136593074 | 4.32E-06 | 4.00E-05 |
| FUT8 | -1.137508516 | 2.28E-07 | 2.74E-06 |
| WDR72 | -1.137651371 | 4.11E-16 | 1.64E-14 |
| PHLDA1 | -1.138092975 | 2.63E-13 | 7.37E-12 |
| SH3KBP1 | -1.139377696 | 2.58E-19 | 1.41E-17 |
| IFNGR2 | -1.139654634 | 4.30E-22 | 3.16E-20 |
| SIPA1L2 | -1.139688963 | 0.00137447 | 0.00629284 |
| MOXD1 | -1.144482446 | 0.00095393 | 0.00461227 |
| FHOD3 | -1.145892283 | 1.27E-05 | 0.00010498 |
| PBX1 | -1.148259318 | 8.23E-08 | 1.07E-06 |
| RP11-558F24 | -1.149874309 | 7.88E-05 | 0.00053239 |
| VIM-AS1 | -1.150399126 | 4.39E-08 | 5.97E-07 |
| CCDC92 | -1.152073885 | 9.56E-10 | 1.69E-08 |
| DYNLT3P1 | -1.153405294 | 4.02E-07 | 4.60E-06 |
| KHDRBS3 | -1.154832387 | 2.87E-08 | 4.02E-07 |
| CTSF | -1.156549811 | 4.11E-06 | 3.82E-05 |
| C1orf106 | -1.156597958 | 3.27E-12 | 8.00E-11 |
| MAP2K6 | -1.158867264 | 3.19E-05 | 0.00023729 |
| PCDHGA7 | -1.160428881 | 3.56E-06 | 3.34E-05 |
| B4GALNT1 | -1.160939458 | 2.56E-15 | 9.20E-14 |
| ACOX2 | -1.162153956 | 7.66E-06 | 6.72E-05 |
| ALG1L6P | -1.162909831 | 0.00032739 | 0.00182512 |
| RHBDF1P1 | -1.163028964 | 1.31E-06 | 1.34E-05 |
| PAX8-AS1 | -1.164827966 | 0.00013768 | 0.00085551 |
| SLC7A11 | -1.168391688 | 0.00052159 | 0.0027244 |
| CARF | -1.168870298 | 1.22E-08 | 1.83E-07 |
| ZNF608 | -1.168878697 | 8.06E-05 | 0.00054264 |
| PAPSS2 | -1.171561044 | 1.54E-21 | 1.08E-19 |
| KCNQ1OT1 | -1.175186048 | 7.06E-06 | 6.25E-05 |
| RHBDF1 | -1.178231897 | 2.79E-17 | 1.23E-15 |
| ENC1 | -1.178260735 | 0.00010347 | 0.00067134 |
| GSAP | -1.179389282 | 4.56E-10 | 8.39E-09 |
| RNF157 | -1.182122424 | 3.68E-13 | 1.01E-11 |
| PPP1R3F | -1.183402473 | 6.82E-08 | 8.94E-07 |
| LCTL | -1.184157269 | 2.37E-05 | 0.000183 |
| WIPF1 | -1.184699622 | 5.19E-21 | 3.38E-19 |
| AHR | -1.189954801 | 7.50E-11 | 1.53E-09 |
| TMEM117 | -1.191205932 | 1.10E-05 | 9.26E-05 |

| | | | |
|---|---|---|---|
| ZNF365 | -1.197445326 | 7.04E-11 | 1.45E-09 |
| PROX1 | -1.19749615 | 3.04E-07 | 3.57E-06 |
| TBXAS1 | -1.203394031 | 1.80E-08 | 2.60E-07 |
| MEOX2 | -1.203445961 | 0.00130033 | 0.00600685 |
| TSC22D1 | -1.203501746 | 2.77E-12 | 6.81E-11 |
| MMP2 | -1.205502298 | 4.15E-12 | 1.00E-10 |
| SPATA6 | -1.206649766 | 2.13E-20 | 1.30E-18 |
| MXRA8 | -1.207214617 | 8.16E-09 | 1.25E-07 |
| S1PR2 | -1.207544507 | 1.24E-07 | 1.55E-06 |
| PHLDA2 | -1.208619075 | 2.58E-16 | 1.05E-14 |
| LTBP1 | -1.210159307 | 3.39E-08 | 4.71E-07 |
| SLC12A2 | -1.210628787 | 1.36E-17 | 6.23E-16 |
| DAAM2 | -1.211492845 | 6.01E-05 | 0.00041726 |
| RP4-792G4.2 | -1.211509704 | 8.00E-05 | 0.00053932 |
| PTHLH | -1.214558374 | 5.01E-05 | 0.00035551 |
| SMIM14 | -1.215700317 | 4.62E-06 | 4.25E-05 |
| C1QTNF1 | -1.21579415 | 7.59E-06 | 6.66E-05 |
| TMCC3 | -1.217838419 | 2.63E-09 | 4.33E-08 |
| HTR7 | -1.218848483 | 1.13E-16 | 4.75E-15 |
| RP11-460N16 | -1.220186562 | 6.12E-05 | 0.00042329 |
| MAML2 | -1.222443467 | 3.91E-28 | 4.99E-26 |
| HS3ST1 | -1.223031638 | 8.44E-06 | 7.31E-05 |
| ESPN | -1.22438873 | 1.36E-06 | 1.39E-05 |
| LPP-AS2 | -1.224538276 | 1.82E-07 | 2.22E-06 |
| AP3B2 | -1.226061218 | 0.00012389 | 0.00078074 |
| FAM78B | -1.226763492 | 6.35E-10 | 1.14E-08 |
| PCDHB11 | -1.22784978 | 0.00027817 | 0.00157957 |
| AC138123.2 | -1.231386167 | 4.36E-06 | 4.03E-05 |
| C16orf52 | -1.232188446 | 1.46E-25 | 1.44E-23 |
| C19orf57 | -1.236498474 | 3.35E-07 | 3.91E-06 |
| RAB17 | -1.236514355 | 6.54E-06 | 5.85E-05 |
| RNF125 | -1.237270532 | 5.91E-07 | 6.49E-06 |
| ELTD1 | -1.238641488 | 0.00034151 | 0.00189419 |
| RASSF8 | -1.241269511 | 1.01E-11 | 2.32E-10 |
| DYNLT3P2 | -1.241548202 | 5.77E-07 | 6.36E-06 |
| ABCC3 | -1.243693185 | 3.38E-07 | 3.93E-06 |
| TCF7L2 | -1.244351568 | 9.42E-13 | 2.45E-11 |
| RNF144A-AS1 | -1.245506228 | 0.00034503 | 0.00190666 |
| MEF2C | -1.247292084 | 1.03E-05 | 8.70E-05 |
| SLC22A17 | -1.249400884 | 1.15E-05 | 9.58E-05 |
| PCDHGC3 | -1.250030355 | 1.97E-06 | 1.95E-05 |
| DCHS1 | -1.25004154 | 1.83E-05 | 0.00014489 |
| XYLT1 | -1.250974894 | 0.00011516 | 0.00073444 |
| RP11-6N17.4 | -1.253259386 | 5.29E-07 | 5.88E-06 |
| ANKRD50 | -1.253383647 | 2.15E-17 | 9.57E-16 |
| RP11-1055B8 | -1.254289746 | 1.50E-06 | 1.52E-05 |
| LEMD1 | -1.255831014 | 0.00024035 | 0.00139508 |
| DYNLT3 | -1.259861264 | 1.37E-19 | 7.66E-18 |
| NTM | -1.260001342 | 1.45E-08 | 2.14E-07 |
| CLMP | -1.26296947 | 1.21E-12 | 3.11E-11 |
| CDON | -1.264121449 | 8.69E-23 | 6.72E-21 |
| ZNF362 | -1.264313037 | 8.86E-18 | 4.12E-16 |
| SH3TC2 | -1.264549366 | 5.40E-21 | 3.50E-19 |
| ANPEP | -1.265995934 | 3.16E-06 | 3.01E-05 |
| C19orf71 | -1.267099635 | 2.48E-09 | 4.10E-08 |
| TLR4 | -1.268741217 | 2.65E-11 | 5.81E-10 |

| | | | |
|---|---|---|---|
| GRK5 | -1.270626804 | 1.22E-13 | 3.62E-12 |
| RTKN | -1.273599461 | 4.16E-23 | 3.28E-21 |
| CYP4X1 | -1.275840234 | 6.85E-07 | 7.45E-06 |
| LPAR6 | -1.276569642 | 6.56E-14 | 2.03E-12 |
| ADAMTS9 | -1.279956265 | 5.80E-05 | 0.00040401 |
| SSBP2 | -1.280873351 | 2.64E-05 | 0.00020054 |
| RASSF8-AS1 | -1.282044222 | 2.33E-08 | 3.31E-07 |
| RNF144A | -1.282726354 | 1.60E-10 | 3.10E-09 |
| DHRS3 | -1.283871107 | 5.82E-07 | 6.41E-06 |
| MFI2 | -1.287395247 | 1.49E-16 | 6.18E-15 |
| ATXN1 | -1.289108294 | 6.22E-12 | 1.46E-10 |
| KLF12 | -1.290832771 | 7.55E-16 | 2.90E-14 |
| PDZD2 | -1.290840501 | 3.77E-07 | 4.33E-06 |
| CLMN | -1.292820669 | 6.30E-06 | 5.66E-05 |
| EYA4 | -1.294025614 | 8.55E-15 | 2.88E-13 |
| SRPX | -1.294530153 | 1.07E-30 | 1.64E-28 |
| BIN3 | -1.29565421 | 9.56E-26 | 9.66E-24 |
| ARHGAP12 | -1.298344515 | 2.24E-22 | 1.68E-20 |
| RP11-66B24. | -1.299709105 | 5.15E-12 | 1.23E-10 |
| LIMCH1 | -1.300063454 | 8.53E-14 | 2.61E-12 |
| SECTM1 | -1.304681698 | 5.49E-05 | 0.00038479 |
| MFAP2 | -1.306608654 | 1.83E-17 | 8.21E-16 |
| SLC25A45 | -1.307376059 | 4.90E-09 | 7.81E-08 |
| SPRY1 | -1.308626675 | 8.46E-06 | 7.32E-05 |
| PITPNC1 | -1.30996918 | 7.96E-08 | 1.04E-06 |
| KCNN4 | -1.310672197 | 2.50E-27 | 3.00E-25 |
| C16orf45 | -1.31403995 | 1.47E-15 | 5.41E-14 |
| AC007362.1 | -1.32037854 | 2.54E-12 | 6.32E-11 |
| CEND1 | -1.322191699 | 7.10E-07 | 7.69E-06 |
| ARRDC3 | -1.323880467 | 4.33E-06 | 4.00E-05 |
| KANK1 | -1.327204092 | 1.22E-34 | 2.50E-32 |
| FAM84B | -1.328228296 | 8.75E-34 | 1.72E-31 |
| ZNF469 | -1.336072161 | 1.91E-12 | 4.78E-11 |
| CTD-2369P2. | -1.346225879 | 1.31E-09 | 2.27E-08 |
| CYB5R2 | -1.350650399 | 0.00044835 | 0.00239098 |
| GAS1 | -1.3540319 | 9.46E-07 | 1.00E-05 |
| UGT8 | -1.355398763 | 4.23E-19 | 2.23E-17 |
| ATP2B4 | -1.355629478 | 2.67E-18 | 1.33E-16 |
| SLC22A18 | -1.358950442 | 6.59E-11 | 1.36E-09 |
| CD58 | -1.359009729 | 2.43E-25 | 2.36E-23 |
| TTLL1 | -1.362614642 | 8.35E-08 | 1.08E-06 |
| RP11-582J16 | -1.362750241 | 1.53E-14 | 5.02E-13 |
| TRPV4 | -1.365625069 | 3.32E-22 | 2.45E-20 |
| ARHGEF40 | -1.366295001 | 4.18E-31 | 6.68E-29 |
| TNFRSF9 | -1.366757787 | 2.73E-05 | 0.00020676 |
| TIGD4 | -1.367378226 | 1.40E-07 | 1.74E-06 |
| TNFRSF21 | -1.367447536 | 1.97E-11 | 4.39E-10 |
| PIANP | -1.370304385 | 1.14E-08 | 1.71E-07 |
| COL6A1 | -1.370527471 | 1.42E-10 | 2.78E-09 |
| EDNRA | -1.372434429 | 0.00010703 | 0.00069086 |
| TMEM47 | -1.379388712 | 0.00169376 | 0.00752058 |
| ISG20 | -1.383853638 | 1.17E-18 | 5.96E-17 |
| ATP6V0A4 | -1.39152146 | 0.00074423 | 0.0037309 |
| AMOTL1 | -1.392614978 | 5.09E-27 | 6.03E-25 |
| PXDN | -1.392958655 | 1.19E-18 | 6.03E-17 |
| FAM86DP | -1.404389082 | 8.95E-20 | 5.15E-18 |

| | | | |
|---|---|---|---|
| IL8 | -1.405498611 | 0.00038584 | 0.00210186 |
| PRSS12 | -1.406933165 | 4.49E-26 | 4.77E-24 |
| ABTB2 | -1.407057444 | 7.41E-19 | 3.80E-17 |
| TMTC2 | -1.409653305 | 6.07E-11 | 1.27E-09 |
| ST8SIA4 | -1.410019549 | 1.89E-05 | 0.0001492 |
| CCDC149 | -1.41214378 | 7.20E-06 | 6.37E-05 |
| PRRT2 | -1.412676902 | 1.18E-08 | 1.76E-07 |
| ERRFI1 | -1.415945707 | 1.11E-13 | 3.32E-12 |
| KLF9 | -1.416746297 | 0.00182645 | 0.00800828 |
| GRAMD1B | -1.418244117 | 8.82E-05 | 0.00058688 |
| COL18A1 | -1.425611548 | 2.51E-10 | 4.76E-09 |
| CTNNBIP1 | -1.427604103 | 3.29E-18 | 1.63E-16 |
| RAB4B | -1.428023281 | 6.12E-16 | 2.39E-14 |
| SEC14L2 | -1.430847932 | 4.53E-18 | 2.21E-16 |
| RCAN1 | -1.43161897 | 2.68E-05 | 0.00020344 |
| DNAH10OS | -1.431657998 | 3.36E-07 | 3.92E-06 |
| FAM134B | -1.432673861 | 1.84E-05 | 0.00014568 |
| PYROXD2 | -1.441214464 | 1.61E-11 | 3.63E-10 |
| KCNS3 | -1.441471678 | 9.17E-07 | 9.75E-06 |
| VAT1L | -1.448361342 | 0.00018515 | 0.00111392 |
| SH2B3 | -1.453563764 | 1.57E-21 | 1.09E-19 |
| LINC00460 | -1.455855124 | 7.83E-10 | 1.40E-08 |
| IGSF3 | -1.45803276 | 4.57E-26 | 4.83E-24 |
| FAM20C | -1.460310572 | 5.15E-13 | 1.38E-11 |
| RP4-758J24.5 | -1.463683148 | 1.63E-08 | 2.38E-07 |
| IL1RAP | -1.464916059 | 3.73E-07 | 4.29E-06 |
| CTD-2207A17 | -1.466863956 | 1.59E-06 | 1.60E-05 |
| ST8SIA1 | -1.471687985 | 0.00086016 | 0.00421457 |
| SLC20A1 | -1.472039336 | 2.91E-25 | 2.79E-23 |
| AC079922.3 | -1.477284505 | 9.14E-13 | 2.38E-11 |
| MDGA1 | -1.47807867 | 8.73E-07 | 9.31E-06 |
| TANC2 | -1.482484184 | 2.08E-20 | 1.28E-18 |
| U73166.2 | -1.482774659 | 2.64E-05 | 0.00020059 |
| NPAS2 | -1.484239907 | 2.04E-48 | 1.16E-45 |
| SPOCK1 | -1.487757688 | 1.53E-10 | 2.98E-09 |
| EDNRB | -1.491076265 | 4.86E-13 | 1.31E-11 |
| GPR56 | -1.504154884 | 2.52E-23 | 2.04E-21 |
| PCDH18 | -1.504242958 | 4.17E-10 | 7.70E-09 |
| INHBA | -1.504400312 | 9.78E-07 | 1.03E-05 |
| GRIK2 | -1.504696751 | 1.76E-14 | 5.73E-13 |
| CNTN3 | -1.507147638 | 2.14E-09 | 3.59E-08 |
| GLT8D2 | -1.508572771 | 0.00023693 | 0.00137817 |
| GNG7 | -1.509076147 | 5.51E-08 | 7.34E-07 |
| MAB21L1 | -1.510939678 | 0.00021278 | 0.00125453 |
| IL1A | -1.511305273 | 0.00080201 | 0.00397577 |
| EGR2 | -1.511880965 | 0.00090754 | 0.00441654 |
| ANKH | -1.512927979 | 1.70E-33 | 3.18E-31 |
| ADAM19 | -1.515341 | 0.00010443 | 0.00067647 |
| ARHGAP31 | -1.516421244 | 6.60E-11 | 1.36E-09 |
| C3orf70 | -1.519109889 | 1.17E-07 | 1.46E-06 |
| LINC00461 | -1.525724073 | 2.16E-08 | 3.09E-07 |
| EPHA7 | -1.527898833 | 1.09E-16 | 4.60E-15 |
| SPRY2 | -1.530385187 | 1.25E-17 | 5.71E-16 |
| ANO1-AS2 | -1.530876789 | 8.09E-06 | 7.05E-05 |
| SLC5A12 | -1.535616583 | 8.96E-05 | 0.00059485 |
| LINC00327 | -1.53699587 | 1.85E-09 | 3.12E-08 |

| | | | |
|---|---|---|---|
| DEPTOR | -1.542894602 | 4.44E-07 | 5.04E-06 |
| CSPG4 | -1.549922571 | 5.40E-18 | 2.60E-16 |
| CNTNAP3B | -1.557828713 | 3.74E-09 | 6.04E-08 |
| DLGAP1-AS2 | -1.558385968 | 1.66E-08 | 2.42E-07 |
| ZCCHC24 | -1.558734664 | 2.09E-15 | 7.58E-14 |
| SERPINA1 | -1.558935301 | 4.48E-05 | 0.00032234 |
| TXK | -1.561995738 | 1.72E-11 | 3.87E-10 |
| MAF | -1.562279829 | 7.61E-11 | 1.55E-09 |
| CBR3 | -1.564701903 | 4.59E-13 | 1.24E-11 |
| RAB9B | -1.569971865 | 1.12E-10 | 2.23E-09 |
| BEND6 | -1.570207486 | 6.13E-11 | 1.28E-09 |
| RNF182 | -1.570880412 | 9.50E-14 | 2.88E-12 |
| CNIH2 | -1.571863043 | 5.98E-19 | 3.11E-17 |
| TRIM62 | -1.575286946 | 6.27E-33 | 1.09E-30 |
| ASAP3 | -1.577830964 | 1.19E-06 | 1.23E-05 |
| STX1A | -1.586025817 | 6.00E-10 | 1.09E-08 |
| RLBP1 | -1.597067314 | 9.04E-07 | 9.63E-06 |
| DNM3 | -1.598641786 | 8.70E-08 | 1.12E-06 |
| IKZF2 | -1.600636139 | 0.00127732 | 0.00592086 |
| SYNM | -1.601740624 | 2.29E-08 | 3.26E-07 |
| FGF12 | -1.601898071 | 4.50E-13 | 1.22E-11 |
| FZD7 | -1.606141804 | 6.19E-14 | 1.93E-12 |
| NRP1 | -1.606566469 | 5.81E-14 | 1.82E-12 |
| GBP2 | -1.617573329 | 0.00039797 | 0.00215932 |
| PAQR8 | -1.623449901 | 1.57E-14 | 5.16E-13 |
| ADAMTS3 | -1.624375532 | 2.75E-16 | 1.11E-14 |
| TMX4 | -1.629573028 | 2.48E-31 | 4.04E-29 |
| HEY1 | -1.629787047 | 6.16E-07 | 6.75E-06 |
| PRR7-AS1 | -1.629877587 | 1.71E-15 | 6.26E-14 |
| RELL1 | -1.644428138 | 2.36E-15 | 8.51E-14 |
| ETV1 | -1.648084719 | 2.77E-25 | 2.67E-23 |
| TRIB2 | -1.648753904 | 1.81E-08 | 2.62E-07 |
| CXCL1 | -1.649449804 | 1.81E-08 | 2.61E-07 |
| P2RX6 | -1.649794725 | 5.03E-07 | 5.63E-06 |
| LRAT | -1.653454727 | 2.33E-23 | 1.89E-21 |
| NOX4 | -1.653552805 | 6.24E-16 | 2.43E-14 |
| NHSL1 | -1.654556233 | 2.12E-44 | 9.18E-42 |
| IL13RA2 | -1.6549522 | 5.88E-58 | 5.38E-55 |
| PDE4B | -1.655197273 | 1.11E-07 | 1.40E-06 |
| LPCAT2 | -1.657585016 | 3.62E-33 | 6.56E-31 |
| TFAP2C | -1.659249367 | 2.88E-55 | 2.15E-52 |
| GJB1 | -1.659982988 | 1.98E-09 | 3.33E-08 |
| COLEC12 | -1.663603712 | 1.68E-06 | 1.68E-05 |
| KIAA0040 | -1.667589807 | 1.00E-08 | 1.52E-07 |
| PPAPDC1A | -1.669057659 | 0.00050007 | 0.00261914 |
| KIAA1217 | -1.669183523 | 3.88E-33 | 6.96E-31 |
| ARHGAP5-AS | -1.669520296 | 1.16E-11 | 2.64E-10 |
| SLC26A4 | -1.670784813 | 7.46E-05 | 0.00050633 |
| AIM1 | -1.679315636 | 5.52E-19 | 2.89E-17 |
| PDK4 | -1.67952479 | 3.79E-06 | 3.54E-05 |
| LOXL4 | -1.683867085 | 6.12E-06 | 5.52E-05 |
| MMP17 | -1.68751469 | 4.96E-21 | 3.27E-19 |
| PCDH1 | -1.689461354 | 4.14E-20 | 2.46E-18 |
| PLEKHA4 | -1.690779893 | 4.94E-13 | 1.33E-11 |
| DACT1 | -1.691739935 | 0.00083599 | 0.00410837 |
| TSPAN13 | -1.693651642 | 1.00E-20 | 6.37E-19 |

| | | | |
|---|---|---|---|
| UNC5D | -1.695206593 | 5.24E-06 | 4.77E-05 |
| BFSP1 | -1.698768314 | 9.14E-05 | 0.00060365 |
| TRIM2 | -1.700646246 | 1.09E-20 | 6.91E-19 |
| TRAF1 | -1.700700506 | 1.49E-08 | 2.19E-07 |
| TRIM9 | -1.70278266 | 9.43E-05 | 0.00061972 |
| KRCC1 | -1.709180989 | 2.96E-19 | 1.61E-17 |
| TMEM2 | -1.720111396 | 4.50E-11 | 9.55E-10 |
| LRP1B | -1.722522001 | 0.00087622 | 0.00428562 |
| SETBP1 | -1.72420276 | 8.05E-09 | 1.24E-07 |
| SORCS1 | -1.725363969 | 3.99E-07 | 4.58E-06 |
| LPHN3 | -1.726769307 | 8.27E-08 | 1.07E-06 |
| VGF | -1.729748972 | 1.56E-17 | 7.06E-16 |
| SCG2 | -1.731308768 | 3.66E-25 | 3.49E-23 |
| AIM2 | -1.73154144 | 1.17E-15 | 4.41E-14 |
| GLDCP1 | -1.734920051 | 1.84E-05 | 0.0001453 |
| CD109 | -1.737030199 | 2.69E-30 | 3.99E-28 |
| MIR146A | -1.739406894 | 2.53E-05 | 0.00019382 |
| FXYD3 | -1.743511496 | 0.00197636 | 0.00857429 |
| ST3GAL5 | -1.748344901 | 1.17E-16 | 4.91E-15 |
| PHEX | -1.749351412 | 0.00016789 | 0.00102015 |
| RP13-735L24 | -1.7496417 | 7.82E-15 | 2.65E-13 |
| SHROOM4 | -1.752372177 | 1.06E-19 | 6.03E-18 |
| PDGFRA | -1.755896213 | 1.09E-07 | 1.38E-06 |
| LOXL3 | -1.758303441 | 3.80E-23 | 3.02E-21 |
| LINC01059 | -1.758525593 | 0.00145861 | 0.00661556 |
| ITGA6 | -1.758557903 | 9.45E-61 | 1.04E-57 |
| RP11-59D5__ | -1.763246993 | 2.36E-20 | 1.44E-18 |
| PCSK1 | -1.763733348 | 0.00082673 | 0.00407135 |
| S100A1 | -1.765020768 | 7.09E-11 | 1.45E-09 |
| RP11-93B14. | -1.770448064 | 4.87E-11 | 1.03E-09 |
| FBXO32 | -1.772196209 | 6.09E-29 | 8.22E-27 |
| RFTN2 | -1.77467352 | 5.10E-05 | 0.00036057 |
| MAML3 | -1.776309184 | 3.25E-44 | 1.34E-41 |
| FRMPD4 | -1.776902309 | 2.81E-06 | 2.70E-05 |
| LINC00473 | -1.780541742 | 1.04E-15 | 3.92E-14 |
| RP11-726G1. | -1.781291927 | 6.27E-12 | 1.46E-10 |
| AMOT | -1.78152471 | 7.10E-25 | 6.57E-23 |
| AATK | -1.783674949 | 9.08E-05 | 0.00060047 |
| RIPK4 | -1.785468758 | 1.28E-21 | 9.11E-20 |
| GPR153 | -1.786141963 | 2.39E-18 | 1.21E-16 |
| SNTB1 | -1.788910354 | 2.42E-07 | 2.89E-06 |
| NR4A2 | -1.790984214 | 3.16E-09 | 5.15E-08 |
| B3GAT1 | -1.792317574 | 3.09E-10 | 5.83E-09 |
| FAM19A2 | -1.7936857 | 7.22E-06 | 6.37E-05 |
| SLC1A1 | -1.796424125 | 5.71E-24 | 4.92E-22 |
| ADAMTS15 | -1.798060291 | 9.56E-07 | 1.01E-05 |
| OLFML2A | -1.799730587 | 0.00011262 | 0.00072158 |
| ERO1LB | -1.802121986 | 3.74E-20 | 2.24E-18 |
| MSC | -1.802859692 | 2.61E-07 | 3.09E-06 |
| C10orf90 | -1.802865458 | 1.22E-09 | 2.12E-08 |
| LRIG3 | -1.804312538 | 8.17E-15 | 2.76E-13 |
| ISLR | -1.80479786 | 3.71E-05 | 0.00027182 |
| CIITA | -1.81004833 | 2.48E-09 | 4.10E-08 |
| ALDH1A3 | -1.810533749 | 3.31E-62 | 3.93E-59 |
| LONRF2 | -1.810979451 | 1.27E-12 | 3.26E-11 |
| EYS | -1.813795906 | 6.58E-18 | 3.13E-16 |

| | | | |
|---|---|---|---|
| THBS2 | -1.815955304 | 5.32E-13 | 1.42E-11 |
| NTSR1 | -1.818305363 | 3.00E-16 | 1.21E-14 |
| PYGB | -1.820261896 | 1.01E-39 | 3.08E-37 |
| STRA6 | -1.821303468 | 5.99E-21 | 3.85E-19 |
| C15orf48 | -1.825088367 | 0.00210001 | 0.00901617 |
| LRRK2 | -1.828085053 | 1.08E-13 | 3.23E-12 |
| PFKFB4 | -1.832656304 | 1.23E-10 | 2.45E-09 |
| BRINP2 | -1.836356018 | 0.00025517 | 0.00146514 |
| SOX6 | -1.838784338 | 5.91E-13 | 1.56E-11 |
| AGPAT9 | -1.843906135 | 2.59E-15 | 9.28E-14 |
| FOLH1 | -1.847407827 | 1.44E-08 | 2.13E-07 |
| MANSC1 | -1.849122808 | 1.51E-46 | 7.55E-44 |
| EPHA4 | -1.856516337 | 3.44E-11 | 7.40E-10 |
| NES | -1.857837405 | 2.41E-16 | 9.79E-15 |
| DMRT2 | -1.861687168 | 1.32E-13 | 3.89E-12 |
| SHC4 | -1.865029497 | 1.16E-10 | 2.31E-09 |
| SVIL | -1.867812129 | 5.61E-42 | 2.01E-39 |
| TMEM229B | -1.868898987 | 1.35E-16 | 5.65E-15 |
| NLGN1 | -1.877715948 | 7.11E-18 | 3.37E-16 |
| AC020907.1 | -1.878629959 | 2.14E-06 | 2.10E-05 |
| MRGPRX4 | -1.882935376 | 1.83E-06 | 1.82E-05 |
| JAG1 | -1.88563035 | 0.00018106 | 0.00109054 |
| CD74 | -1.888982617 | 7.25E-18 | 3.42E-16 |
| SIRPB1 | -1.889339089 | 1.83E-16 | 7.54E-15 |
| PLP1 | -1.89993675 | 7.54E-05 | 0.00051116 |
| BAMBI | -1.900955288 | 2.78E-21 | 1.88E-19 |
| SLITRK6 | -1.906328815 | 1.75E-09 | 2.97E-08 |
| ITGA10 | -1.906348982 | 3.16E-05 | 0.00023564 |
| ARNT2 | -1.907824182 | 8.60E-24 | 7.26E-22 |
| HLA-DRB5 | -1.921099339 | 3.57E-09 | 5.78E-08 |
| PTGES | -1.921574373 | 1.34E-06 | 1.37E-05 |
| FAM19A3 | -1.921812626 | 8.97E-06 | 7.70E-05 |
| CHST6 | -1.929756126 | 1.08E-12 | 2.79E-11 |
| SERPINE2 | -1.933037478 | 2.71E-11 | 5.91E-10 |
| ITGB8 | -1.935598781 | 3.21E-17 | 1.40E-15 |
| PLA2G7 | -1.938930527 | 6.16E-11 | 1.28E-09 |
| FREM2 | -1.94655965 | 3.31E-21 | 2.23E-19 |
| COL9A2 | -1.966755011 | 1.20E-16 | 5.01E-15 |
| RNF150 | -1.97168217 | 5.34E-11 | 1.12E-09 |
| CXCL3 | -1.974904317 | 5.05E-09 | 8.03E-08 |
| TRPM8 | -1.975645769 | 0.00031771 | 0.00178077 |
| ABHD17C | -1.98001446 | 1.22E-28 | 1.64E-26 |
| CTHRC1 | -1.980111787 | 1.30E-19 | 7.30E-18 |
| NREP | -1.985592846 | 6.94E-26 | 7.10E-24 |
| INPP5F | -1.987561808 | 1.20E-47 | 6.36E-45 |
| TIMP3 | -1.991218279 | 1.82E-46 | 8.81E-44 |
| CYP27C1 | -1.991473077 | 1.54E-13 | 4.50E-12 |
| CD200 | -1.997431055 | 1.22E-05 | 0.00010156 |
| FGF13 | -2.001336811 | 4.64E-16 | 1.84E-14 |
| FZD1 | -2.004955037 | 1.50E-40 | 4.83E-38 |
| LINC01060 | -2.006515644 | 7.00E-16 | 2.70E-14 |
| LINC00511 | -2.00751653 | 1.85E-19 | 1.03E-17 |
| KIAA1199 | -2.010230015 | 2.79E-05 | 0.0002104 |
| ITGA1 | -2.017864685 | 6.24E-12 | 1.46E-10 |
| MUC5B | -2.021703634 | 8.68E-16 | 3.30E-14 |
| RP11-599J14 | -2.021775885 | 2.86E-18 | 1.43E-16 |

| | | | |
|---|---|---|---|
| RHOJ | -2.035250017 | 1.64E-08 | 2.39E-07 |
| GSN | -2.037670486 | 1.36E-07 | 1.69E-06 |
| RP11-472N13 | -2.041212892 | 2.89E-08 | 4.05E-07 |
| PTN | -2.042260294 | 1.25E-22 | 9.46E-21 |
| TMPRSS5 | -2.052192358 | 1.07E-20 | 6.77E-19 |
| SHROOM2 | -2.061508802 | 4.21E-34 | 8.45E-32 |
| RP11-390P2.4 | -2.062213608 | 6.01E-14 | 1.87E-12 |
| SDC3 | -2.064081957 | 1.11E-09 | 1.95E-08 |
| PRSS33 | -2.072616831 | 1.43E-05 | 0.00011559 |
| AC145436.1 | -2.087050461 | 5.15E-10 | 9.40E-09 |
| SERPINF1 | -2.09023766 | 0.00230687 | 0.00974137 |
| IL18R1 | -2.097165393 | 1.73E-05 | 0.0001373 |
| GLDC | -2.099955895 | 1.58E-10 | 3.07E-09 |
| GNG2 | -2.103581173 | 1.43E-44 | 6.39E-42 |
| OPRD1 | -2.104044152 | 5.26E-12 | 1.25E-10 |
| STARD4-AS1 | -2.108352018 | 3.16E-15 | 1.12E-13 |
| SOX10 | -2.112284703 | 1.49E-39 | 4.40E-37 |
| RP11-34A14. | -2.1209423 | 1.24E-05 | 0.00010281 |
| RP4-718J7.4 | -2.121467927 | 2.37E-09 | 3.93E-08 |
| LINGO2 | -2.121986282 | 2.61E-18 | 1.31E-16 |
| SOBP | -2.131320383 | 2.94E-26 | 3.19E-24 |
| CPN1 | -2.137310035 | 1.33E-10 | 2.61E-09 |
| FLT1 | -2.147749463 | 6.68E-09 | 1.04E-07 |
| ANO1 | -2.164135129 | 1.52E-30 | 2.28E-28 |
| CDH19 | -2.16641223 | 8.80E-11 | 1.78E-09 |
| CAPS | -2.167627209 | 4.76E-20 | 2.81E-18 |
| HEY2 | -2.169428413 | 3.25E-05 | 0.00024123 |
| LINC00326 | -2.170546099 | 2.67E-20 | 1.62E-18 |
| BCL2A1 | -2.173280349 | 0.00013763 | 0.00085551 |
| COL6A3 | -2.180489652 | 0.0007415 | 0.00371945 |
| FCGR2A | -2.181479651 | 6.67E-09 | 1.04E-07 |
| SLC26A2 | -2.187670868 | 1.69E-35 | 3.77E-33 |
| WSCD1 | -2.187705089 | 0.0001308 | 0.00081841 |
| NRCAM | -2.192105387 | 2.40E-16 | 9.79E-15 |
| TGFA | -2.202426875 | 1.01E-16 | 4.30E-15 |
| SFRP1 | -2.203077995 | 2.82E-43 | 1.08E-40 |
| PMEPA1 | -2.212418681 | 2.33E-05 | 0.00018014 |
| CTC-529P8.1 | -2.215247042 | 2.10E-16 | 8.66E-15 |
| GAS7 | -2.220709774 | 5.68E-12 | 1.34E-10 |
| COL22A1 | -2.224473487 | 0.00032322 | 0.00180623 |
| AFAP1L2 | -2.224617588 | 4.17E-10 | 7.70E-09 |
| HIVEP3 | -2.227234489 | 2.41E-73 | 6.61E-70 |
| SLC24A3 | -2.228492177 | 2.16E-09 | 3.61E-08 |
| CHRM3 | -2.24571852 | 1.04E-10 | 2.07E-09 |
| CORO2B | -2.254623371 | 1.36E-21 | 9.59E-20 |
| EXTL1 | -2.26779066 | 1.16E-32 | 1.97E-30 |
| ABCA10 | -2.269698509 | 0.00114172 | 0.00538744 |
| LYPD1 | -2.27889099 | 2.26E-13 | 6.44E-12 |
| SERPIND1 | -2.280937381 | 9.16E-06 | 7.84E-05 |
| ID4 | -2.280987543 | 1.68E-05 | 0.00013392 |
| S100B | -2.288348333 | 2.08E-13 | 5.95E-12 |
| AGMO | -2.290850792 | 3.39E-06 | 3.20E-05 |
| RAMP1 | -2.291580167 | 6.57E-36 | 1.52E-33 |
| AMACR | -2.298884455 | 1.51E-14 | 4.98E-13 |
| ACAN | -2.301085433 | 2.55E-13 | 7.19E-12 |
| EPHB3 | -2.30376181 | 1.18E-20 | 7.40E-19 |

| | | | |
|---|---|---|---|
| MIA | -2.305010276 | 7.19E-28 | 9.04E-26 |
| RP11-399D6. | -2.314543627 | 1.85E-27 | 2.25E-25 |
| PRRX1 | -2.325751585 | 1.99E-11 | 4.41E-10 |
| LSAMP | -2.337180761 | 6.31E-10 | 1.14E-08 |
| ACPL2 | -2.338261478 | 2.97E-22 | 2.22E-20 |
| FZD8 | -2.351071619 | 1.20E-13 | 3.59E-12 |
| MGC4294 | -2.352642562 | 9.90E-09 | 1.50E-07 |
| PLEKHB1 | -2.354834648 | 5.05E-16 | 1.99E-14 |
| HTRA1 | -2.367817353 | 4.94E-18 | 2.38E-16 |
| SHISA2 | -2.368339763 | 2.78E-07 | 3.28E-06 |
| FAM65B | -2.371237698 | 1.24E-10 | 2.45E-09 |
| CD96 | -2.380899073 | 3.31E-24 | 2.93E-22 |
| PART1 | -2.390967245 | 5.42E-17 | 2.33E-15 |
| MMP16 | -2.406196164 | 3.34E-62 | 3.93E-59 |
| CNTNAP3 | -2.414984995 | 5.51E-26 | 5.75E-24 |
| TNFRSF19 | -2.419251209 | 9.15E-40 | 2.84E-37 |
| CTD-2023N9. | -2.42086427 | 1.59E-08 | 2.32E-07 |
| CHST1 | -2.426748146 | 2.13E-23 | 1.75E-21 |
| DNAH9 | -2.443797386 | 2.09E-07 | 2.53E-06 |
| COL9A3 | -2.448185513 | 2.09E-53 | 1.38E-50 |
| FLRT3 | -2.457162521 | 1.36E-15 | 5.04E-14 |
| VIT | -2.464075835 | 3.17E-05 | 0.00023631 |
| SERPINA5 | -2.473504114 | 1.12E-41 | 3.91E-39 |
| FCRLA | -2.475860738 | 1.55E-29 | 2.20E-27 |
| PTPRZ1 | -2.480812826 | 9.92E-08 | 1.27E-06 |
| B3GNT7 | -2.492995824 | 7.18E-26 | 7.30E-24 |
| SNED1 | -2.495946866 | 1.29E-19 | 7.26E-18 |
| SYTL5 | -2.496238463 | 2.26E-21 | 1.55E-19 |
| AC017104.6 | -2.499187565 | 1.98E-24 | 1.79E-22 |
| UCN2 | -2.541785567 | 1.42E-13 | 4.17E-12 |
| HR | -2.551818341 | 4.14E-64 | 6.20E-61 |
| AL121578.2 | -2.567063012 | 6.57E-14 | 2.03E-12 |
| IGFBP5 | -2.573225123 | 1.64E-05 | 0.00013116 |
| GRM8 | -2.576404935 | 2.03E-17 | 9.05E-16 |
| SOX5 | -2.5765761 | 1.95E-33 | 3.60E-31 |
| EPHA3 | -2.590877579 | 1.65E-33 | 3.13E-31 |
| EGR3 | -2.596800336 | 1.24E-17 | 5.71E-16 |
| RP11-370B11 | -2.63434927 | 2.38E-20 | 1.45E-18 |
| LAMA4 | -2.643069602 | 2.13E-59 | 2.20E-56 |
| HES1 | -2.65067795 | 4.75E-12 | 1.14E-10 |
| FABP7 | -2.653394261 | 3.16E-08 | 4.42E-07 |
| CSGALNACT1 | -2.67272024 | 5.54E-37 | 1.32E-34 |
| SERPINA3 | -2.673875227 | 2.26E-09 | 3.76E-08 |
| SPRY4 | -2.677006054 | 3.04E-22 | 2.26E-20 |
| ITIH6 | -2.678615155 | 1.33E-05 | 0.00010938 |
| CDKN1C | -2.704260791 | 5.87E-27 | 6.86E-25 |
| RP11-277B15 | -2.725820084 | 5.16E-07 | 5.75E-06 |
| SOX5P | -2.733101222 | 1.17E-22 | 8.89E-21 |
| ATOH8 | -2.739329287 | 1.44E-13 | 4.23E-12 |
| RP1-212P9.2 | -2.74457678 | 3.67E-19 | 1.96E-17 |
| NFATC2 | -2.759091175 | 1.95E-07 | 2.37E-06 |
| TESC | -2.763201687 | 4.26E-19 | 2.23E-17 |
| ERBB3 | -2.778704945 | 1.14E-39 | 3.40E-37 |
| ENPP2 | -2.795471997 | 6.91E-05 | 0.0004724 |
| HILS1 | -2.804607481 | 1.36E-15 | 5.04E-14 |
| CTD-2023N9. | -2.829626425 | 1.23E-45 | 5.79E-43 |

| | | | |
|---|---|---|---|
| COL15A1 | -2.848038779 | 9.49E-05 | 0.0006229 |
| ZP4 | -2.849177585 | 1.53E-10 | 2.99E-09 |
| CHRDL1 | -2.856743257 | 8.21E-13 | 2.15E-11 |
| PDE3A | -2.882450375 | 3.45E-15 | 1.22E-13 |
| TFAP2B | -2.91486089 | 5.86E-08 | 7.77E-07 |
| RXRG | -2.960655416 | 3.61E-05 | 0.00026522 |
| COL19A1 | -2.974268578 | 5.03E-21 | 3.30E-19 |
| ARPP21 | -3.010876131 | 9.78E-30 | 1.41E-27 |
| ITGA9 | -3.014858709 | 1.40E-06 | 1.42E-05 |
| PKNOX2 | -3.031950179 | 1.58E-15 | 5.82E-14 |
| ITGB3 | -3.173822451 | 1.11E-100 | 1.83E-96 |
| APOD | -3.1899115 | 7.77E-08 | 1.01E-06 |
| RP11-408N14 | -3.200162298 | 4.47E-24 | 3.90E-22 |
| ART3 | -3.23083861 | 3.02E-40 | 9.57E-38 |
| ITIH5 | -3.252141131 | 9.83E-05 | 0.00064232 |
| TNC | -3.376372547 | 1.22E-76 | 4.01E-73 |
| EYA1 | -3.42608129 | 1.08E-30 | 1.65E-28 |
| HMCN1 | -3.428972573 | 3.51E-11 | 7.54E-10 |
| SOD3 | -3.449552178 | 1.02E-08 | 1.53E-07 |
| NPPC | -3.50025496 | 5.11E-08 | 6.86E-07 |
| HPN-AS1 | -3.514370088 | 3.91E-15 | 1.37E-13 |
| SLC24A2 | -3.600536498 | 5.35E-07 | 5.95E-06 |
| ABCA6 | -3.618578981 | 1.55E-05 | 0.00012445 |
| MGP | -4.684127849 | 1.48E-05 | 0.00011993 |

**Table S8. NEDD4L, WWTR1 and EGFR expression across cell lines.**

Expression values of NEDD4L, WWTR1 and EGFR in a panel of cancer cell lines. Gene expression values were measured by U219 microarray.

| Cell line | NEDD4L | WWTR1 | EGFR |
|---|---|---|---|
| PL-21 | 2.29175 | 3.14747 | 3.08188 |
| A204 | 2.46693 | 9.76958 | 2.96693 |
| MC116 | 2.54269 | 2.55284 | 3.18262 |
| Ramos-2G6-4C10 | 2.5779 | 2.53478 | 6.63972 |
| G-402 | 2.63731 | 8.01423 | 3.04396 |
| KARPAS-231 | 2.64618 | 2.80359 | 3.21789 |
| H-EMC-SS | 2.71171 | 5.84024 | 2.94718 |
| HL-60 | 2.72787 | 2.70074 | 3.14476 |
| MONO-MAC-6 | 2.7746 | 2.7232 | 2.84806 |
| OCI-LY-19 | 2.79175 | 2.67394 | 3.40683 |
| G-401 | 2.82343 | 5.83319 | 3.16732 |
| EoL-1-cell | 2.85012 | 3.06937 | 2.94594 |
| L-363 | 2.86067 | 2.65865 | 3.25626 |
| MV-4-11 | 2.86962 | 2.72333 | 3.0987 |
| KCL-22 | 2.90695 | 4.18698 | 3.14445 |
| OACM5-1 | 2.91929 | 6.46573 | 3.38265 |
| MEC-1 | 2.93905 | 3.09251 | 3.3709 |
| BV-173 | 2.98501 | 2.61102 | 3.16617 |
| K052 | 3.05215 | 3.14375 | 2.8366 |
| KYM-1 | 3.08404 | 8.43412 | 3.19788 |
| MONO-MAC-1 | 3.12507 | 2.92721 | 2.94759 |
| P12-ICHIKAWA | 3.1771 | 3.19293 | 2.88247 |
| MOLM-13 | 3.19557 | 2.68904 | 3.43888 |
| OCI-AML5 | 3.2346 | 2.83405 | 3.14259 |
| MOLT-13 | 3.24741 | 3.06261 | 3.23675 |
| EM-2 | 3.29223 | 2.97651 | 2.86686 |
| SW1573 | 3.29984 | 6.68208 | 7.41624 |
| SU-DHL-8 | 3.31909 | 2.66084 | 3.04439 |
| SU-DHL-10 | 3.36307 | 2.8242 | 3.2845 |
| KOPN-8 | 3.37767 | 3.08347 | 3.3129 |
| KY821 | 3.3792 | 2.65768 | 2.93901 |
| NB12 | 3.38383 | 2.88889 | 3.18327 |
| EW-7 | 3.38847 | 6.15968 | 4.29662 |
| NCI-SNU-1 | 3.41904 | 3.67536 | 8.31838 |
| CTV-1 | 3.43954 | 3.46741 | 3.15467 |
| NCI-H661 | 3.4717 | 6.65515 | 5.65966 |
| GI-1 | 3.52318 | 7.57045 | 5.1899 |
| SK-N-FI | 3.58173 | 2.90908 | 4.95306 |
| TE-8 | 3.61225 | 7.64022 | 10.3142 |
| MOLT-4 | 3.61819 | 2.84584 | 3.31428 |
| THP-1 | 3.66676 | 3.36618 | 3.30791 |
| YKG-1 | 3.67156 | 8.82111 | 6.07505 |
| RS4-11 | 3.67658 | 2.5773 | 2.78862 |
| PFSK-1 | 3.72409 | 7.38534 | 3.79638 |
| MOLT-16 | 3.75874 | 3.09166 | 3.04515 |
| SK-N-SH | 3.79025 | 5.89253 | 5.55912 |
| NBsusSR | 3.81285 | 6.81668 | 3.2286 |
| ML-2 | 3.85776 | 2.71967 | 3.15017 |
| JEKO-1 | 3.87626 | 2.83461 | 3.11932 |
| NB10 | 3.88277 | 2.71143 | 3.49471 |
| MDA-MB-157 | 3.88394 | 8.39938 | 5.50282 |

| | | | |
|---|---|---|---|
| CCRF-CEM | 3.90922 | 2.44938 | 2.82998 |
| DND-41 | 3.98335 | 2.62751 | 3.30207 |
| SBC-3 | 3.98431 | 6.63784 | 3.50979 |
| NCI-H28 | 3.99419 | 9.09851 | 8.68852 |
| HARA | 4.00568 | 7.33234 | 8.0758 |
| LU-165 | 4.02867 | 3.06078 | 3.64416 |
| ST486 | 4.03976 | 2.7282 | 2.78877 |
| A172 | 4.0425 | 9.213 | 7.31012 |
| VAL | 4.05575 | 2.97463 | 2.95198 |
| D-263MG | 4.05821 | 6.78869 | 6.49302 |
| OCI-AML2 | 4.12048 | 4.77586 | 4.40405 |
| ES4 | 4.12296 | 6.23549 | 5.49444 |
| U251 | 4.13378 | 8.91668 | 6.49193 |
| RPMI-2650 | 4.15091 | 6.35833 | 2.8809 |
| OPM-2 | 4.18273 | 2.87842 | 3.43432 |
| NB69 | 4.22115 | 3.15014 | 7.30148 |
| BT-549 | 4.24169 | 8.98339 | 7.63612 |
| LAN-6 | 4.24752 | 2.85826 | 3.32244 |
| D-502MG | 4.2476 | 9.78209 | 5.65669 |
| CHP-212 | 4.26475 | 6.53659 | 4.47928 |
| SW962 | 4.27282 | 7.74197 | 7.549 |
| KE-37 | 4.28375 | 2.75053 | 3.11442 |
| AMO-1 | 4.30056 | 2.8729 | 3.35934 |
| NU-DUL-1 | 4.30063 | 2.62976 | 2.96971 |
| HOP-62 | 4.31282 | 8.01497 | 5.50918 |
| NCI-H929 | 4.35669 | 3.64348 | 2.97655 |
| HAL-01 | 4.36055 | 2.60411 | 2.61569 |
| KMRC-20 | 4.36218 | 8.32604 | 8.8028 |
| PF-382 | 4.36364 | 3.05208 | 3.23562 |
| NCI-H1155 | 4.37014 | 2.57208 | 4.77053 |
| JJN-3 | 4.38113 | 2.87475 | 3.44359 |
| D-542MG | 4.38948 | 7.38474 | 7.13914 |
| NH-12 | 4.40022 | 5.6352 | 7.2398 |
| MFE-280 | 4.4018 | 8.3614 | 5.03862 |
| KP-4 | 4.43518 | 9.55462 | 5.21188 |
| IST-MES1 | 4.44324 | 7.10899 | 9.01044 |
| TUR | 4.44622 | 2.74552 | 3.02749 |
| BE2-M17 | 4.45599 | 3.08325 | 5.49529 |
| H2804 | 4.46916 | 7.92369 | 8.5183 |
| FADU | 4.4786 | 6.43074 | 9.46033 |
| COLO-678 | 4.48605 | 4.82674 | 8.50743 |
| DK-MG | 4.5243 | 8.96146 | 6.18631 |
| NCI-H1563 | 4.52758 | 8.46215 | 8.49442 |
| LC4-1 | 4.5293 | 3.07578 | 3.1732 |
| ALL-PO | 4.54368 | 3.07563 | 3.25777 |
| SK-PN-DW | 4.56308 | 5.74135 | 3.08068 |
| HCC1569 | 4.59069 | 7.42777 | 3.46292 |
| EFM-19 | 4.59083 | 6.85815 | 3.08339 |
| CA46 | 4.59114 | 3.08209 | 3.08189 |
| P30-OHK | 4.59682 | 2.81926 | 3.78332 |
| RH-1 | 4.60948 | 6.99193 | 3.75947 |
| ES1 | 4.63581 | 6.39388 | 5.09958 |
| NCI-H810 | 4.63804 | 2.64863 | 2.85083 |
| HCC1937 | 4.65254 | 8.25699 | 7.19141 |
| MHH-PREB-1 | 4.68175 | 2.91734 | 3.2699 |
| P32-ISH | 4.68332 | 2.88229 | 3.09492 |

| | | | |
|---|---|---|---|
| KURAMOCHI | 4.69273 | 8.39407 | 4.14552 |
| KINGS-1 | 4.69955 | 9.86846 | 5.68036 |
| CGTH-W-1 | 4.73972 | 8.32427 | 7.1593 |
| A673 | 4.74203 | 7.71803 | 3.13048 |
| SNB75 | 4.74622 | 8.49853 | 5.84854 |
| A498 | 4.74833 | 7.13026 | 9.40476 |
| EB-3 | 4.78485 | 2.95038 | 2.70046 |
| EW-16 | 4.78671 | 6.06726 | 4.19937 |
| A4-Fuk | 4.79244 | 4.57474 | 3.01248 |
| KELLY | 4.83067 | 2.62696 | 6.37542 |
| COLO-320 | 4.85323 | 7.77404 | 3.14818 |
| H513 | 4.87835 | 6.8554 | 6.40164 |
| J-RT3-T3-5 | 4.88431 | 2.55306 | 3.11966 |
| REH | 4.9001 | 2.42217 | 3.10732 |
| SUP-T1 | 4.90423 | 3.01531 | 3.14032 |
| BEN | 4.90966 | 4.46975 | 3.54948 |
| NCI-H196 | 4.9232 | 7.85755 | 7.01138 |
| ES3 | 4.92354 | 4.7664 | 3.03392 |
| D-336MG | 4.92452 | 6.50463 | 5.99601 |
| MMAC-SF | 4.925 | 8.63661 | 3.58639 |
| 697 | 4.9361 | 3.75562 | 3.24559 |
| KMRC-1 | 4.94316 | 9.1018 | 9.4841 |
| HCC2157 | 4.94593 | 4.72114 | 4.16288 |
| NB5 | 4.94605 | 6.32454 | 5.37258 |
| SCC-3 | 4.95723 | 4.28656 | 2.95485 |
| SW872 | 4.96772 | 8.98897 | 6.86514 |
| KLE | 4.97009 | 8.14805 | 5.99241 |
| U-698-M | 4.97389 | 3.04043 | 3.52197 |
| LU-135 | 4.97659 | 2.55893 | 5.7856 |
| BE-13 | 4.97897 | 2.80591 | 3.26343 |
| SF539 | 4.98471 | 7.60296 | 6.04184 |
| EC-GI-10 | 5.0105 | 6.57508 | 9.10897 |
| HT-1197 | 5.0105 | 6.16083 | 8.88638 |
| HeLa | 5.01418 | 7.79611 | 6.08517 |
| MG-63 | 5.01776 | 9.53436 | 6.39713 |
| SNU-C2B | 5.02725 | 7.1244 | 4.26119 |
| ECC10 | 5.03612 | 2.40184 | 3.01966 |
| NB14 | 5.03907 | 2.62423 | 4.13021 |
| OSC-20 | 5.04084 | 6.51039 | 9.22686 |
| COLO-320-HSR | 5.05601 | 7.70817 | 2.82907 |
| KNS-81-FD | 5.07266 | 9.84247 | 6.18764 |
| HCE-T | 5.08053 | 6.36388 | 8.3841 |
| SR | 5.08602 | 3.02538 | 3.11115 |
| MS-1 | 5.10893 | 6.70379 | 5.53786 |
| KARPAS-620 | 5.12691 | 4.39789 | 3.19795 |
| NOMO-1 | 5.13536 | 2.6546 | 2.79275 |
| U-87-MG | 5.15378 | 6.001 | 6.9932 |
| T98G | 5.16365 | 7.62437 | 7.60866 |
| QIMR-WIL | 5.16438 | 4.56544 | 2.99087 |
| HeLaSF | 5.16529 | 6.72412 | 6.37834 |
| SNU-182 | 5.16771 | 6.59486 | 5.37347 |
| NCI-H1651 | 5.17527 | 7.64164 | 6.74322 |
| OVCAR-8 | 5.20582 | 6.20367 | 6.61121 |
| NAMALWA | 5.2167 | 2.71512 | 3.49143 |
| OVCAR-4 | 5.21976 | 6.59668 | 8.57621 |
| 23132-87 | 5.22015 | 3.09578 | 4.35822 |

| | | | |
|---|---|---|---|
| A253 | 5.2256 | 6.68796 | 9.52141 |
| NCI-H1666 | 5.23906 | 6.9699 | 7.19487 |
| NCI-H378 | 5.24534 | 2.76289 | 4.46161 |
| BB49-HNC | 5.24746 | 7.91625 | 6.1319 |
| OVCAR-3 | 5.24991 | 7.51934 | 7.7836 |
| NCI-H1623 | 5.25099 | 4.17032 | 6.65772 |
| BC-1 | 5.25673 | 5.22867 | 3.0135 |
| LNZTA3WT4 | 5.25827 | 9.23904 | 7.14863 |
| HEL | 5.2627 | 2.47175 | 2.89179 |
| H2731 | 5.26884 | 7.50747 | 9.05592 |
| EW-11 | 5.2747 | 2.7937 | 3.01558 |
| EW-13 | 5.28209 | 4.32472 | 3.03632 |
| LOUCY | 5.2976 | 2.6898 | 3.34979 |
| MRK-nu-1 | 5.29844 | 5.29774 | 3.21191 |
| KNS-62 | 5.31131 | 7.43849 | 9.36684 |
| PCI-6A | 5.31248 | 6.22844 | 9.79409 |
| RERF-GC-1B | 5.31366 | 6.04707 | 9.34654 |
| OCUB-M | 5.32039 | 5.292 | 3.90137 |
| ES8 | 5.3296 | 5.81778 | 5.53451 |
| HOS | 5.3392 | 7.49372 | 7.07811 |
| WSU-NHL | 5.34652 | 2.92175 | 2.82436 |
| TCCSUP | 5.37698 | 6.94573 | 7.01006 |
| SiHa | 5.38149 | 5.55585 | 6.85575 |
| RERF-LC-MS | 5.38155 | 8.2791 | 6.78074 |
| PSN1 | 5.38275 | 9.04026 | 7.00509 |
| D-392MG | 5.39426 | 7.26899 | 6.0756 |
| Ca9-22 | 5.39624 | 8.28727 | 10.7995 |
| SUIT-2 | 5.40086 | 8.20236 | 8.40323 |
| Becker | 5.41718 | 8.23924 | 7.03194 |
| WIL2-NS | 5.41987 | 4.55662 | 3.16275 |
| NCI-H1581 | 5.42393 | 4.92422 | 3.44919 |
| JURL-MK1 | 5.42675 | 4.9151 | 2.6808 |
| SNU-398 | 5.43158 | 6.05349 | 3.90346 |
| PC-14 | 5.43982 | 7.41507 | 9.13002 |
| DOHH-2 | 5.45129 | 2.68207 | 3.35024 |
| OS-RC-2 | 5.46525 | 9.33599 | 9.76297 |
| SUP-B15 | 5.46564 | 4.13493 | 3.36003 |
| SNU-387 | 5.46807 | 8.91374 | 6.20876 |
| ES5 | 5.46851 | 6.80381 | 3.24684 |
| BT-20 | 5.50868 | 6.99045 | 10.1916 |
| T-T | 5.51056 | 7.13014 | 9.13496 |
| SW837 | 5.51664 | 2.88468 | 6.53656 |
| OVK-18 | 5.51981 | 8.51903 | 3.36716 |
| EFM-192A | 5.5293 | 5.8701 | 3.16134 |
| HUTU-80 | 5.53253 | 5.74435 | 3.24836 |
| EW-1 | 5.53428 | 6.47233 | 3.49532 |
| KARPAS-422 | 5.54456 | 2.72735 | 2.99503 |
| BFTC-905 | 5.54781 | 8.40233 | 8.90341 |
| RH-41 | 5.54868 | 7.35466 | 4.00121 |
| G-292-Clone-A141B1 | 5.55538 | 8.04635 | 8.01802 |
| UMC-11 | 5.56006 | 2.85002 | 3.0813 |
| KP-1N | 5.57664 | 7.84552 | 7.74798 |
| SJRH30 | 5.57989 | 7.49481 | 4.30875 |
| HD-MY-Z | 5.58042 | 9.35813 | 6.30322 |
| SK-MM-2 | 5.58715 | 2.72137 | 3.16557 |
| NB7 | 5.58921 | 6.39011 | 3.56709 |

| | | | |
|---|---|---|---|
| SBC-1 | 5.58957 | 2.95922 | 3.42169 |
| PE-CA-PJ15 | 5.59579 | 8.41695 | 7.91411 |
| BHY | 5.60181 | 7.19584 | 8.44688 |
| GI-ME-N | 5.60402 | 8.00317 | 7.86231 |
| CAL-27 | 5.6124 | 6.62308 | 10.1814 |
| SKM-1 | 5.61673 | 2.61425 | 2.75886 |
| NCI-H3122 | 5.61756 | 6.57262 | 6.81077 |
| A427 | 5.62146 | 6.3588 | 5.08734 |
| AU565 | 5.62601 | 6.3237 | 7.9903 |
| T84 | 5.63883 | 3.51897 | 7.30176 |
| ECC12 | 5.65016 | 4.19944 | 2.81146 |
| D-247MG | 5.65467 | 8.37341 | 7.36559 |
| HA7-RCC | 5.65905 | 9.61235 | 8.67852 |
| no-11 | 5.66044 | 8.38706 | 8.18232 |
| ML-1 | 5.66897 | 7.05994 | 8.32865 |
| KM-H2 | 5.6779 | 4.71383 | 3.45748 |
| OAW-42 | 5.67863 | 8.01162 | 7.09008 |
| KYSE-450 | 5.67931 | 7.09816 | 11.061 |
| SBC-5 | 5.68117 | 2.53093 | 2.88345 |
| SW13 | 5.69341 | 3.25798 | 3.20295 |
| TE-1 | 5.6973 | 6.72062 | 8.34397 |
| HGC-27 | 5.70376 | 6.10208 | 2.96515 |
| NCI-H1092 | 5.71392 | 2.81803 | 3.05986 |
| NCI-H841 | 5.71533 | 4.59537 | 7.30871 |
| SW48 | 5.71669 | 4.20891 | 7.44937 |
| TT2609-C02 | 5.71931 | 8.97808 | 7.28577 |
| SK-NEP-1 | 5.7261 | 4.42518 | 2.96786 |
| HCE-4 | 5.72713 | 7.2461 | 6.85643 |
| NCI-H838 | 5.73288 | 7.98298 | 4.75851 |
| LU-65 | 5.73851 | 9.74349 | 9.84195 |
| I-9-2 | 5.7402 | 2.85495 | 3.62295 |
| NALM-6 | 5.74752 | 2.73307 | 3.04507 |
| PA-TU-8988T | 5.7548 | 6.29048 | 8.86472 |
| EW-22 | 5.76319 | 5.58403 | 3.1178 |
| KYSE-70 | 5.77292 | 7.16682 | 9.56195 |
| NCI-H2405 | 5.77486 | 8.34393 | 8.51049 |
| EW-3 | 5.78173 | 4.73554 | 2.90329 |
| Saos-2 | 5.78807 | 7.82526 | 5.73986 |
| RKN | 5.7886 | 7.85253 | 9.09782 |
| COR-L88 | 5.80204 | 2.94448 | 3.33703 |
| SW1990 | 5.80957 | 7.64419 | 7.99624 |
| NTERA-S-cl-D1 | 5.81932 | 5.84412 | 4.19988 |
| SK-OV-3 | 5.81977 | 6.75602 | 6.42783 |
| IMR-5 | 5.82053 | 2.5251 | 4.93895 |
| CAMA-1 | 5.82557 | 4.68743 | 3.23032 |
| OCI-M1 | 5.82606 | 2.80531 | 3.08935 |
| LAMA-84 | 5.82913 | 4.25023 | 3.1344 |
| LS-123 | 5.83687 | 7.12854 | 7.81526 |
| SW900 | 5.83692 | 8.23998 | 7.08875 |
| ABC-1 | 5.83891 | 4.57672 | 9.01771 |
| OE21 | 5.84455 | 6.32022 | 11.915 |
| SNU-475 | 5.84462 | 7.69618 | 5.6343 |
| TOV-112D | 5.84662 | 7.11627 | 3.31006 |
| GOTO | 5.85333 | 2.58087 | 3.18937 |
| NCI-H64 | 5.85605 | 3.0546 | 3.99295 |
| KS-1 | 5.86523 | 8.26189 | 6.44079 |

| | | | |
|---|---|---|---|
| TE-12 | 5.86776 | 6.44161 | 9.3965 |
| HCC-15 | 5.87101 | 6.41733 | 7.00614 |
| OACp4C | 5.87309 | 6.04569 | 6.45099 |
| KYSE-510 | 5.87489 | 5.95044 | 7.90173 |
| A549 | 5.87886 | 8.61249 | 7.10468 |
| CCK-81 | 5.88063 | 2.69999 | 4.87149 |
| EN | 5.88302 | 7.97789 | 3.16365 |
| NCI-H1648 | 5.88599 | 7.36447 | 7.80803 |
| NCC021 | 5.88751 | 8.92224 | 8.56289 |
| SW684 | 5.89294 | 7.41317 | 5.85284 |
| NCI-H2126 | 5.90892 | 4.85497 | 3.09406 |
| PCI-15A | 5.91545 | 7.18663 | 12.2089 |
| NCI-H596 | 5.91623 | 7.05981 | 8.67262 |
| H3255 | 5.9214 | 7.83414 | 11.4202 |
| RD | 5.92522 | 9.58012 | 6.39012 |
| A2780 | 5.926 | 7.1155 | 4.34682 |
| GAMG | 5.93064 | 8.15308 | 8.98281 |
| WM-115 | 5.93616 | 8.15921 | 8.26874 |
| MFH-ino | 5.9433 | 7.32546 | 7.30088 |
| RT-112 | 5.94809 | 6.67088 | 8.16805 |
| NCI-H2452 | 5.9613 | 7.57147 | 9.0994 |
| SW1463 | 5.96276 | 2.79399 | 5.19226 |
| SK-MEL-24 | 5.96353 | 9.28432 | 4.57151 |
| ES7 | 5.96406 | 7.02696 | 3.48609 |
| HT-1376 | 5.96822 | 7.24591 | 8.91936 |
| HCT-116 | 5.9689 | 7.74398 | 6.29801 |
| JEG-3 | 5.97213 | 6.02361 | 6.92064 |
| NCI-H747 | 5.9753 | 6.13576 | 8.29484 |
| MDST8 | 5.98046 | 7.85055 | 7.59859 |
| EPLC-272H | 5.98134 | 7.68971 | 8.24645 |
| OVKATE | 5.98456 | 7.97 | 8.88737 |
| CAKI-1 | 5.99333 | 9.03007 | 8.34201 |
| HCC1954 | 5.99697 | 6.27978 | 7.63363 |
| RPMI-8226 | 6.0014 | 2.90755 | 3.72054 |
| GA-10 | 6.00587 | 2.77701 | 2.83303 |
| CAL-85-1 | 6.01376 | 7.77573 | 8.53613 |
| CAPAN-1 | 6.01444 | 6.83701 | 6.67662 |
| MCF7 | 6.01512 | 4.47626 | 3.00531 |
| U-266 | 6.01594 | 2.5482 | 3.21925 |
| KYSE-50 | 6.01709 | 7.05965 | 10.0711 |
| CAL-39 | 6.01864 | 8.10311 | 7.72383 |
| SU-DHL-1 | 6.03089 | 3.03037 | 3.10627 |
| WM278 | 6.04124 | 10.6664 | 5.22566 |
| OC-314 | 6.04153 | 8.1222 | 6.9802 |
| ONS-76 | 6.04215 | 8.14964 | 7.58398 |
| H2795 | 6.04695 | 7.17294 | 7.18745 |
| WSU-DLCL2 | 6.05051 | 2.93654 | 3.05869 |
| HCC1428 | 6.05198 | 6.86238 | 4.78574 |
| DoTc2-4510 | 6.05676 | 7.66644 | 8.82908 |
| COLO-680N | 6.05921 | 6.64453 | 8.96541 |
| MES-SA | 6.06154 | 8.39511 | 3.16412 |
| EW-24 | 6.07343 | 5.73538 | 3.22884 |
| NCI-H520 | 6.0767 | 6.04229 | 3.16403 |
| RH-18 | 6.08376 | 7.33177 | 6.50779 |
| NCI-H1688 | 6.08515 | 5.43618 | 5.60108 |
| NCI-H1944 | 6.08723 | 7.00062 | 5.53735 |

| | | | |
|---|---|---|---|
| ChaGo-K-1 | 6.08736 | 6.31863 | 9.10108 |
| LXF-289 | 6.08798 | 8.29143 | 8.5695 |
| NCI-H1573 | 6.09658 | 6.22229 | 9.9264 |
| SKN | 6.09699 | 7.25769 | 7.19246 |
| NCI-H740 | 6.10431 | 2.92152 | 3.40226 |
| H3118 | 6.10779 | 8.45293 | 9.82657 |
| KNS-42 | 6.11104 | 7.99448 | 3.94277 |
| BHT-101 | 6.11735 | 8.10168 | 7.52453 |
| NCI-H1299 | 6.11774 | 7.76498 | 6.32932 |
| NCI-H209 | 6.12659 | 3.23397 | 4.24549 |
| H2803 | 6.13719 | 8.04877 | 8.36295 |
| EW-18 | 6.14287 | 5.19991 | 3.17045 |
| TE-6 | 6.14395 | 8.6487 | 9.77706 |
| ME-180 | 6.1443 | 7.85416 | 9.43498 |
| HSC-3 | 6.14479 | 7.5271 | 10.2997 |
| 769-P | 6.15124 | 9.73249 | 7.86166 |
| SUP-B8 | 6.15138 | 2.99659 | 3.20607 |
| JHH-6 | 6.15212 | 5.35336 | 8.18678 |
| MEG-01 | 6.15555 | 4.44703 | 3.14892 |
| HCC2218 | 6.15898 | 4.00502 | 6.17912 |
| A388 | 6.16997 | 7.90866 | 11.1064 |
| OVISE | 6.17056 | 10.1091 | 8.75893 |
| JHU-011 | 6.17178 | 6.43624 | 7.90499 |
| CTB-1 | 6.17549 | 2.47833 | 3.00299 |
| NCI-H2085 | 6.17738 | 7.34566 | 8.79045 |
| MEC-2 | 6.17827 | 3.11051 | 3.22395 |
| SW780 | 6.18029 | 6.15961 | 7.86847 |
| SCC-15 | 6.18395 | 6.96235 | 9.64705 |
| NCI-H522 | 6.19178 | 5.65124 | 2.98464 |
| GCT | 6.19311 | 7.3906 | 7.64721 |
| U-118-MG | 6.20143 | 8.15552 | 7.20175 |
| LS-1034 | 6.20497 | 3.15591 | 4.51008 |
| Calu-6 | 6.2072 | 9.58922 | 6.27162 |
| HCC1500 | 6.20797 | 2.76959 | 3.52942 |
| PC-3_[JPC-3] | 6.21062 | 6.15865 | 9.05356 |
| PCI-38 | 6.22054 | 7.61243 | 10.4373 |
| IGROV-1 | 6.22424 | 8.58241 | 5.46431 |
| HCC-56 | 6.22616 | 6.66912 | 4.83864 |
| NCI-H2029 | 6.23539 | 3.23207 | 4.20703 |
| H2722 | 6.243 | 8.14627 | 8.45025 |
| T47D | 6.24402 | 6.29811 | 5.00086 |
| OV-90 | 6.25283 | 7.45794 | 5.11507 |
| NB13 | 6.25364 | 2.75494 | 4.6602 |
| TGBC11TKB | 6.26141 | 5.54354 | 4.02511 |
| RPMI-7951 | 6.26668 | 7.23284 | 7.02202 |
| MKN1 | 6.26684 | 6.82154 | 7.03405 |
| CL-11 | 6.28007 | 2.70852 | 7.47165 |
| NCI-H835 | 6.28582 | 3.07439 | 3.00539 |
| MFE-319 | 6.28582 | 6.11942 | 5.27536 |
| SW620 | 6.29024 | 3.87248 | 3.15177 |
| PCI-30 | 6.29846 | 6.43146 | 9.02884 |
| Hs-766T | 6.30006 | 6.37546 | 6.90691 |
| HCC1187 | 6.30151 | 5.12829 | 6.63342 |
| NCI-H1694 | 6.3035 | 2.50817 | 4.78869 |
| WM-266-4 | 6.30536 | 8.48319 | 3.01344 |
| SCC-4 | 6.30674 | 7.40238 | 10.4508 |

| | | | |
|---|---|---|---|
| CHP-134 | 6.31048 | 2.55296 | 3.3412 |
| OE33 | 6.31227 | 7.6766 | 8.17612 |
| NCI-H2342 | 6.31676 | 7.00106 | 9.75984 |
| LU-99A | 6.32509 | 6.68 | 9.50909 |
| RCC-MF | 6.33546 | 8.64418 | 8.67728 |
| NCI-H1755 | 6.33584 | 7.29904 | 6.69962 |
| NCI-H2227 | 6.34162 | 2.86822 | 3.03444 |
| LS-513 | 6.34342 | 2.57413 | 7.00787 |
| OAW-28 | 6.34873 | 7.86434 | 7.20271 |
| KALS-1 | 6.35826 | 7.71951 | 5.01585 |
| DEL | 6.36457 | 3.22404 | 3.11612 |
| NCI-H1568 | 6.38701 | 6.22239 | 11.4626 |
| RCM-1 | 6.38949 | 2.90263 | 6.37983 |
| LU-134-A | 6.39084 | 2.39606 | 3.17615 |
| BB30-HNC | 6.39335 | 6.63852 | 9.36668 |
| T-24 | 6.39555 | 8.42309 | 7.68006 |
| EMC-BAC-2 | 6.40758 | 8.68015 | 7.36242 |
| ES-2 | 6.40814 | 6.04338 | 8.38473 |
| ARH-77 | 6.4312 | 2.88253 | 3.47117 |
| DMS-114 | 6.43492 | 5.16045 | 3.41544 |
| DOK | 6.43742 | 6.40429 | 11.7614 |
| SW954 | 6.43783 | 7.96871 | 9.02009 |
| BCPAP | 6.44187 | 6.86331 | 7.67462 |
| NMC-G1 | 6.44491 | 8.62825 | 8.4013 |
| A101D | 6.44575 | 9.85043 | 5.60378 |
| OE19 | 6.45166 | 2.51379 | 6.80961 |
| KYSE-410 | 6.46078 | 7.68888 | 8.27597 |
| UACC-62 | 6.46571 | 10.5339 | 4.92297 |
| NCI-H1355 | 6.46705 | 7.68804 | 6.41626 |
| SK-ES-1 | 6.48238 | 5.65396 | 2.82005 |
| TE-15 | 6.49583 | 7.62856 | 8.13079 |
| CAL-120 | 6.50345 | 6.85677 | 8.00876 |
| BOKU | 6.51063 | 11.6518 | 7.61939 |
| HDQ-P1 | 6.51689 | 7.3317 | 7.94311 |
| C2BBe1 | 6.51936 | 7.43927 | 6.83505 |
| DB | 6.52037 | 3.00938 | 3.24372 |
| NCI-H1963 | 6.53342 | 2.39816 | 3.10083 |
| COLO-205 | 6.53632 | 4.702 | 5.58331 |
| SKN-3 | 6.54839 | 6.35483 | 9.82087 |
| VA-ES-BJ | 6.54841 | 8.90604 | 7.08147 |
| NCI-H23 | 6.54933 | 7.31702 | 5.84449 |
| HH | 6.55651 | 5.46887 | 3.13218 |
| BPH-1 | 6.55924 | 7.0015 | 8.95723 |
| SISO | 6.56568 | 7.02231 | 8.50434 |
| Daoy | 6.56581 | 8.01003 | 7.25393 |
| CAL-72 | 6.5663 | 6.50288 | 6.0464 |
| A431 | 6.56883 | 7.62881 | 11.1723 |
| NCI-H2228 | 6.56967 | 8.03896 | 8.52948 |
| Hs-683 | 6.57368 | 7.48488 | 7.35251 |
| KATOIII | 6.57991 | 2.6564 | 6.23702 |
| 639-V | 6.58169 | 8.43867 | 7.04036 |
| OVMIU | 6.58588 | 8.35354 | 7.87365 |
| NCI-H1304 | 6.58937 | 2.89933 | 3.13748 |
| LN-405 | 6.59282 | 7.65574 | 6.00121 |
| IM-9 | 6.59616 | 3.68249 | 3.40642 |
| MKN28 | 6.59816 | 4.88102 | 9.26521 |

| | | | |
|---|---|---|---|
| YAPC | 6.61703 | 8.80872 | 8.175 |
| NCI-H358 | 6.61995 | 8.14634 | 6.12374 |
| TE-5 | 6.62757 | 7.61224 | 9.51009 |
| HCC1419 | 6.62873 | 4.96666 | 3.44802 |
| TE-9 | 6.63029 | 7.25119 | 8.74673 |
| OVCAR-5 | 6.63134 | 8.80474 | 8.36464 |
| H2595 | 6.6368 | 7.9014 | 7.22068 |
| YH-13 | 6.65237 | 8.82107 | 8.01683 |
| LOU-NH91 | 6.65854 | 4.74913 | 9.1052 |
| FU-OV-1 | 6.67133 | 7.58307 | 7.87348 |
| BT-483 | 6.6795 | 3.91261 | 2.98145 |
| HCC1806 | 6.68061 | 7.23256 | 9.03041 |
| NCI-H1734 | 6.68105 | 6.9475 | 6.10234 |
| PANC-10-05 | 6.68256 | 6.24651 | 8.51209 |
| JHH-7 | 6.68759 | 5.47621 | 5.9312 |
| NCI-H1993 | 6.68789 | 7.83168 | 7.18913 |
| SN12C | 6.68871 | 5.91429 | 8.27968 |
| NCI-H647 | 6.68973 | 7.87521 | 5.28961 |
| JHH-4 | 6.6908 | 9.02197 | 8.64762 |
| HPAC | 6.69742 | 7.09192 | 7.55941 |
| H2869 | 6.70388 | 7.08723 | 8.09477 |
| HN | 6.71513 | 6.49417 | 9.26557 |
| RC-K8 | 6.71749 | 3.20534 | 2.96765 |
| HCC202 | 6.71803 | 4.96102 | 4.8024 |
| NCI-H2444 | 6.71937 | 7.60735 | 8.62379 |
| C-33-A | 6.72159 | 4.7959 | 3.053 |
| IST-SL2 | 6.72295 | 6.10604 | 4.83471 |
| H2810 | 6.72538 | 6.25373 | 8.05211 |
| SNU-449 | 6.72621 | 8.45293 | 8.1491 |
| A375 | 6.72641 | 11.116 | 3.58785 |
| CAL-33 | 6.7295 | 8.16634 | 10.497 |
| L-1236 | 6.73079 | 6.43394 | 3.24532 |
| OSC-19 | 6.73139 | 7.18735 | 9.33391 |
| CAL-29 | 6.7365 | 8.37025 | 9.52144 |
| KYSE-150 | 6.74924 | 6.19528 | 8.92779 |
| NCI-H1395 | 6.74969 | 8.21479 | 6.23945 |
| NCI-H322M | 6.75034 | 6.49436 | 7.28031 |
| KP-3 | 6.75894 | 8.09 | 8.32956 |
| PA-1 | 6.7597 | 5.92814 | 3.8482 |
| no-10 | 6.76126 | 7.44921 | 6.59983 |
| 42-MG-BA | 6.76212 | 7.80571 | 7.96754 |
| HSC-4 | 6.76477 | 7.55372 | 10.1719 |
| SHP-77 | 6.76713 | 2.45328 | 6.79564 |
| ZR-75-30 | 6.76997 | 5.28788 | 3.89638 |
| CAL-78 | 6.77441 | 7.60568 | 4.85138 |
| HCC2998 | 6.7852 | 2.97471 | 6.45844 |
| JHH-2 | 6.78648 | 8.7858 | 8.34296 |
| NCI-H727 | 6.78886 | 6.07407 | 7.8562 |
| NCI-H2023 | 6.7964 | 7.63017 | 7.09997 |
| RCC-AB | 6.80067 | 9.27776 | 7.12022 |
| HPAF-II | 6.80826 | 6.41131 | 8.03045 |
| TE-10 | 6.81307 | 7.83892 | 8.34394 |
| M14 | 6.82792 | 8.56651 | 3.86145 |
| TGBC24TKB | 6.82995 | 3.51183 | 5.34895 |
| NCI-H1915 | 6.84951 | 7.97284 | 6.78147 |
| LB647-SCLC | 6.84971 | 2.65954 | 2.96952 |

| | | | |
|---|---|---|---|
| PL18 | 6.85045 | 7.90456 | 6.57121 |
| SW1710 | 6.85573 | 8.82773 | 7.62284 |
| SF126 | 6.85803 | 8.66301 | 7.30197 |
| NB17 | 6.86187 | 2.7327 | 5.77513 |
| H4 | 6.87734 | 7.37287 | 6.77383 |
| UACC-893 | 6.87824 | 6.16309 | 7.15514 |
| RCC-JW | 6.88632 | 8.71828 | 9.04942 |
| HT-115 | 6.89037 | 4.76546 | 5.85671 |
| DAN-G | 6.89885 | 9.24045 | 8.49277 |
| HT-29 | 6.89987 | 2.60853 | 5.97077 |
| ETK-1 | 6.90393 | 6.34668 | 8.11432 |
| SW982 | 6.91062 | 8.57538 | 6.07235 |
| SW1088 | 6.9109 | 7.63525 | 7.21327 |
| CAS-1 | 6.91319 | 8.14881 | 5.43786 |
| EMC-BAC-1 | 6.91484 | 7.11454 | 7.88769 |
| IST-SL1 | 6.93685 | 3.74108 | 3.05453 |
| HT-3 | 6.93937 | 5.84975 | 7.81318 |
| NCI-H1793 | 6.94064 | 8.57462 | 6.69037 |
| HCC-44 | 6.94461 | 7.09531 | 6.30246 |
| NCI-H630 | 6.94527 | 3.23899 | 4.86226 |
| NCI-H510A | 6.94563 | 2.83713 | 3.89048 |
| HCC-827 | 6.947 | 6.1163 | 10.0566 |
| NCI-H1650 | 6.94895 | 7.39554 | 8.16808 |
| KYSE-140 | 6.95006 | 6.23116 | 10.1254 |
| U031 | 6.95574 | 7.96737 | 8.03781 |
| LN-18 | 6.96289 | 5.63079 | 7.31959 |
| EHEB | 6.96326 | 4.22152 | 3.10797 |
| RF-48 | 6.96445 | 4.25103 | 3.36329 |
| HuO9 | 6.97409 | 7.51009 | 3.25646 |
| LS-411N | 6.97667 | 4.41599 | 6.08188 |
| SCC-25 | 6.98009 | 6.98626 | 9.14915 |
| MS751 | 6.98197 | 7.42418 | 9.98452 |
| HCT-15 | 6.98377 | 4.38509 | 5.79304 |
| H2461 | 6.98504 | 6.66096 | 9.1761 |
| S-117 | 6.9938 | 6.52406 | 6.34228 |
| TOV-21G | 6.99664 | 7.37932 | 5.30101 |
| AN3-CA | 6.9994 | 7.13967 | 7.57215 |
| IST-MEL1 | 7.00248 | 9.58076 | 5.93493 |
| EB2 | 7.00301 | 3.00703 | 3.99624 |
| NCI-SNU-16 | 7.00975 | 2.54275 | 6.8355 |
| MDA-MB-361 | 7.02109 | 4.73915 | 4.15373 |
| RKO | 7.0247 | 6.91223 | 4.10275 |
| ESO26 | 7.02702 | 5.439 | 6.24147 |
| J82 | 7.03186 | 6.55433 | 6.36117 |
| NCI-H187 | 7.03775 | 2.8129 | 3.36014 |
| SCC-9 | 7.03989 | 6.79303 | 8.40028 |
| SW948 | 7.04981 | 2.9643 | 6.35903 |
| 786-o | 7.06584 | 7.73891 | 9.20224 |
| LU-139 | 7.07255 | 2.91566 | 3.66463 |
| RXF393 | 7.07266 | 8.89928 | 7.98856 |
| NCI-H650 | 7.07425 | 7.97188 | 5.99996 |
| NCI-H1975 | 7.08366 | 7.46717 | 7.56072 |
| KOSC-2 | 7.08727 | 7.45041 | 9.55989 |
| RMG-I | 7.08832 | 8.10155 | 7.88603 |
| MDA-MB-453 | 7.11053 | 4.44602 | 3.46733 |
| RL | 7.11395 | 2.55814 | 3.27241 |

| | | | |
|---|---|---|---|
| SU-DHL-4 | 7.11914 | 2.95332 | 3.28524 |
| MZ2-MEL | 7.12857 | 10.7312 | 3.18317 |
| JAR | 7.12876 | 5.83638 | 6.78345 |
| KYSE-520 | 7.13432 | 6.89454 | 12.2212 |
| BICR78 | 7.13555 | 6.67256 | 9.34861 |
| EFO-27 | 7.14428 | 7.03272 | 6.27845 |
| 647-V | 7.15344 | 6.74686 | 8.54274 |
| NCI-H2135 | 7.15471 | 8.10766 | 6.94659 |
| SNU-423 | 7.15913 | 6.59627 | 6.63647 |
| NCI-H1838 | 7.1606 | 6.74757 | 9.26529 |
| DSH1 | 7.17281 | 3.46103 | 6.60434 |
| GP5d | 7.17456 | 4.32451 | 6.6775 |
| HCC70 | 7.17553 | 7.55572 | 7.77452 |
| NCI-H1869 | 7.18415 | 7.04378 | 7.17914 |
| NCI-H2291 | 7.18711 | 7.07593 | 7.69875 |
| SCH | 7.19351 | 6.89681 | 7.23663 |
| CPC-N | 7.20407 | 5.8492 | 3.10559 |
| Hs-633T | 7.20839 | 7.86799 | 7.28398 |
| GB-1 | 7.21014 | 7.29873 | 5.36547 |
| CAL-54 | 7.23044 | 8.81778 | 9.43504 |
| SKG-IIIa | 7.23156 | 7.85663 | 7.56888 |
| SK-UT-1 | 7.23829 | 7.1947 | 4.68555 |
| HuH-7 | 7.2392 | 6.11998 | 8.41523 |
| IM-95 | 7.24099 | 2.4541 | 6.40401 |
| SF268 | 7.24581 | 4.55173 | 6.90245 |
| NY | 7.24726 | 6.44081 | 7.60553 |
| LN-229 | 7.25234 | 9.54254 | 6.06343 |
| CFPAC-1 | 7.25592 | 9.65191 | 7.7158 |
| SW1116 | 7.2582 | 4.32313 | 7.25387 |
| JHU-022 | 7.26 | 6.12304 | 7.37642 |
| COLO-668 | 7.2621 | 4.3707 | 5.17182 |
| Detroit562 | 7.27738 | 8.39898 | 9.88616 |
| BICR22 | 7.29045 | 7.90959 | 8.9347 |
| MFM-223 | 7.29687 | 6.71019 | 2.98584 |
| NCI-H146 | 7.31763 | 2.75028 | 3.22856 |
| H9 | 7.34026 | 3.13408 | 3.31969 |
| HT55 | 7.34273 | 5.02868 | 5.63879 |
| RPMI-8866 | 7.34426 | 2.36006 | 3.18678 |
| HDLM-2 | 7.35397 | 4.78797 | 3.00348 |
| Capan-2 | 7.35958 | 7.47496 | 7.52188 |
| ESO51 | 7.37359 | 7.35636 | 6.00478 |
| PANC-08-13 | 7.37617 | 8.46415 | 7.72122 |
| ATN-1 | 7.3764 | 4.08203 | 2.97739 |
| IHH-4 | 7.38702 | 7.35697 | 7.52167 |
| NCI-H1792 | 7.39284 | 8.01114 | 7.55389 |
| HCC-78 | 7.39286 | 7.43598 | 6.15221 |
| COLO-824 | 7.40784 | 6.61505 | 5.76266 |
| DG-75 | 7.40988 | 2.97486 | 2.9557 |
| VCaP | 7.41596 | 3.9071 | 5.01519 |
| CAL-51 | 7.42142 | 8.45772 | 5.32267 |
| EVSA-T | 7.43 | 5.73966 | 4.18427 |
| MKN7 | 7.43018 | 6.01617 | 7.419 |
| NCI-H1048 | 7.44338 | 7.13187 | 7.2481 |
| Ca-Ski | 7.44997 | 5.17917 | 9.41067 |
| KGN | 7.45275 | 6.88941 | 8.30496 |
| EKVX | 7.45636 | 7.25247 | 7.56653 |

| | | | |
|---|---|---|---|
| WM35 | 7.46126 | 9.71119 | 3.43283 |
| HEC-1 | 7.46717 | 7.91315 | 6.54755 |
| MPP-89 | 7.46737 | 8.03936 | 5.54188 |
| PANC-03-27 | 7.47795 | 8.16984 | 8.17096 |
| WM1552C | 7.48394 | 8.32636 | 7.50685 |
| SK-LMS-1 | 7.48769 | 9.06293 | 8.32452 |
| NCI-H2009 | 7.4928 | 5.33257 | 5.08033 |
| NCI-H526 | 7.49449 | 2.95297 | 2.9554 |
| M059K | 7.49716 | 7.79407 | 6.59717 |
| KYSE-270 | 7.52352 | 8.54419 | 9.54786 |
| LCLC-97TM1 | 7.52888 | 9.45026 | 8.18275 |
| JVM-3 | 7.53213 | 2.75031 | 2.95262 |
| C8166 | 7.53384 | 4.00271 | 3.47105 |
| SK-LU-1 | 7.53837 | 6.46881 | 6.03435 |
| NCI-H2347 | 7.57743 | 7.55113 | 7.38096 |
| LS-180 | 7.57999 | 5.22845 | 7.16801 |
| SK-MES-1 | 7.58012 | 8.17993 | 8.498 |
| H290 | 7.59034 | 7.326 | 6.71407 |
| NCI-H1781 | 7.6072 | 5.91866 | 6.31139 |
| RERF-LC-Sq1 | 7.61221 | 7.29273 | 8.87593 |
| COLO-684 | 7.61767 | 3.16443 | 2.74805 |
| MZ1-PC | 7.6284 | 7.32382 | 8.43244 |
| SK-MG-1 | 7.63822 | 7.09673 | 7.04222 |
| JHH-1 | 7.67177 | 10.1397 | 8.06302 |
| COLO-792 | 7.67265 | 8.28091 | 3.28165 |
| SAT | 7.6794 | 6.04442 | 9.73127 |
| AM-38 | 7.69065 | 5.34152 | 8.22171 |
| H2369 | 7.69585 | 6.71648 | 8.83124 |
| JiyoyeP-2003 | 7.70158 | 2.88561 | 3.15277 |
| QGP-1 | 7.71006 | 3.22111 | 6.14681 |
| JSC-1 | 7.71344 | 2.84071 | 3.12835 |
| BALL-1 | 7.73452 | 2.60809 | 2.99984 |
| LB2241-RCC | 7.73815 | 8.82177 | 9.18531 |
| 201T | 7.74144 | 7.83406 | 7.2053 |
| GMS-10 | 7.74166 | 7.62097 | 7.24156 |
| TE-11 | 7.76035 | 6.95182 | 9.09108 |
| HuCCT1 | 7.76714 | 7.08992 | 9.10108 |
| SLVL | 7.78411 | 3.81656 | 3.20555 |
| DU-4475 | 7.78934 | 2.50192 | 3.19442 |
| HT-144 | 7.80437 | 10.2998 | 3.03779 |
| OCUM-1 | 7.81221 | 6.33343 | 9.54516 |
| FU97 | 7.82912 | 4.7289 | 6.67193 |
| KON | 7.83162 | 6.89001 | 8.8263 |
| COLO-800 | 7.85215 | 9.55209 | 2.93196 |
| HuP-T3 | 7.85244 | 7.67361 | 7.35548 |
| SW1783 | 7.87508 | 6.02284 | 7.57884 |
| H2591 | 7.8761 | 6.01161 | 8.31348 |
| SW1417 | 7.90578 | 6.56752 | 8.07915 |
| BB65-RCC | 7.90598 | 8.21929 | 7.70675 |
| HuP-T4 | 7.9236 | 6.42363 | 5.7123 |
| RPMI-6666 | 7.94519 | 3.70862 | 3.00535 |
| NCI-H441 | 7.96092 | 7.51324 | 7.25831 |
| SK-MEL-1 | 7.96871 | 9.83174 | 3.47823 |
| HO-1-N-1 | 7.99765 | 6.55597 | 8.88704 |
| SCaBER | 8.00648 | 7.54101 | 9.99952 |
| NCI-H2052 | 8.01395 | 5.20625 | 7.27062 |

| | | | |
|---|---|---|---|
| SAS | 8.01697 | 6.58405 | 9.99697 |
| COLO-679 | 8.03297 | 9.01601 | 2.94234 |
| Hs746T | 8.03794 | 7.06749 | 6.21223 |
| NCI-H69 | 8.06651 | 2.76481 | 3.04721 |
| NCI-H211 | 8.09329 | 2.78969 | 3.1231 |
| IGR-1 | 8.10748 | 7.05833 | 3.04271 |
| C3A | 8.11634 | 7.02957 | 3.37738 |
| EGI-1 | 8.13511 | 7.28743 | 7.36196 |
| CRO-AP2 | 8.16418 | 2.82015 | 3.02159 |
| DU-145 | 8.17604 | 7.07786 | 8.00041 |
| Mewo | 8.1882 | 7.44397 | 3.48765 |
| HOP-92 | 8.23771 | 7.10246 | 7.77807 |
| G-MEL | 8.24189 | 7.66913 | 3.86564 |
| MEL-JUSO | 8.2648 | 9.68625 | 3.38982 |
| SH-4 | 8.2836 | 9.88913 | 3.42613 |
| IA-LM | 8.29516 | 8.69623 | 8.6407 |
| H2818 | 8.30025 | 6.4524 | 8.88633 |
| NCI-H1703 | 8.30357 | 8.3519 | 6.90962 |
| BT-474 | 8.31185 | 5.28139 | 5.60326 |
| CP66-MEL | 8.32333 | 10.2813 | 3.10287 |
| 5637 | 8.33373 | 5.57463 | 9.12172 |
| GCIY | 8.34806 | 8.39923 | 8.87534 |
| GAK | 8.35305 | 9.24208 | 3.84626 |
| LCLC-103H | 8.3614 | 6.21909 | 7.38795 |
| EFO-21 | 8.36716 | 8.27209 | 6.09686 |
| NCI-H82 | 8.3767 | 2.69141 | 2.91497 |
| VM-CUB-1 | 8.37827 | 6.98025 | 8.30773 |
| HLE | 8.39955 | 7.67923 | 7.11844 |
| SK-MEL-3 | 8.41362 | 7.54634 | 3.7036 |
| C32 | 8.41979 | 8.87284 | 3.83382 |
| 8305C | 8.43423 | 7.56793 | 9.31241 |
| ASH-3 | 8.49397 | 6.56798 | 6.90605 |
| COR-L105 | 8.49695 | 7.97371 | 9.08088 |
| MDA-MB-231 | 8.553 | 6.27124 | 7.75613 |
| DMS-79 | 8.56821 | 2.617 | 6.2535 |
| MZ7-mel | 8.57412 | 7.68197 | 3.20973 |
| HTC-C3 | 8.588 | 6.98804 | 6.60085 |
| RVH-421 | 8.6292 | 8.91381 | 3.55494 |
| NCI-H2030 | 8.655 | 7.27525 | 7.35353 |
| RT4 | 8.70732 | 5.73217 | 7.71938 |
| 451Lu | 8.73591 | 8.74712 | 3.15106 |
| CHL-1 | 8.7364 | 6.13536 | 3.65379 |
| HT-1080 | 8.74479 | 9.04744 | 7.00548 |
| IPC-298 | 8.76217 | 6.71894 | 3.01614 |
| UM-UC-3 | 8.79107 | 8.15783 | 7.23648 |
| Hs-944-T | 8.79545 | 8.28207 | 3.2912 |
| SK-MEL-5 | 8.79856 | 8.57057 | 3.67394 |
| GT3TKB | 8.80424 | 6.69426 | 8.95896 |
| HMV-II | 8.86767 | 9.42482 | 4.2442 |
| SK-MEL-28 | 8.8855 | 9.47798 | 3.49545 |
| A2058 | 8.91199 | 8.88747 | 3.53598 |
| LOXIMVI | 8.91679 | 7.22021 | 7.26013 |
| Mo-T | 8.95362 | 2.76117 | 3.15439 |
| NCI-H1693 | 8.96045 | 4.20302 | 8.48689 |
| DJM-1 | 8.97009 | 7.63068 | 8.75106 |
| NUGC-3 | 9.0076 | 5.19675 | 8.62371 |

| | | | |
|---|---|---|---|
| D-283MED | 9.11192 | 7.08141 | 2.93053 |
| SK-HEP-1 | 9.1143 | 6.62532 | 8.60087 |
| H2373 | 9.11435 | 5.98409 | 7.89281 |
| WM793B | 9.12145 | 8.20376 | 7.19387 |
| MEL-HO | 9.14272 | 7.90761 | 3.03935 |
| EBC-1 | 9.15328 | 8.23697 | 7.76145 |
| RO82-W-1 | 9.19641 | 5.51826 | 5.15562 |
| UACC-257 | 9.24477 | 8.20484 | 2.82874 |
| G-361 | 9.27926 | 8.02832 | 3.72157 |
| SK-MEL-30 | 9.32927 | 8.11061 | 3.45604 |
| CaR-1 | 9.33483 | 8.47066 | 8.50633 |
| IGR-37 | 9.56842 | 7.44125 | 4.01854 |
| CESS | 9.95113 | 4.46382 | 2.94334 |
| COR-L23 | 10.0948 | 7.31989 | 8.04282 |

**SUPPLEMENTARY METHODS**

**Transposon mutagenesis library construction and screen.** To construct transposon mutagenesis libraries, cells were seeded in ten T175 culture flasks at cell density of $2.5 \times 10^6$ cells per flask. After 24-hour incubation, each flask was transfected with 12 µg pPB-SB-CMV-puro-SD, 12 µg transposase plasmid pCMV-hyPBase, 48 µl X-tremeGENE 9 DNA transfection reagent (Roche) and 1.5 ml OPTI-MEM (Thermo Fisher Scientific). After three days, cells were selected using 2 µg/ml puromycin with medium refreshment every 4 days and cryo-preserved after 8 days of treatment. To screen for survivors of PLX4720 treatment, $3 \times 10^6$ cells from each library was plated on a 15 cm tissue culture dish, and treated with 10 µM PLX4720 for three weeks, with fresh medium and drug replacement twice a week. A control dish seeded with the same number of untransfected parental cells was treated in parallel to monitor selection. After this screen, 30-50 colonies emerged from each library while no colonies were found on the control plate. Clearly isolated founder colonies were randomly picked from screening plates using cloning discs (Sigma-Aldrich) and remaining cells were then trypsinized and expanded to 2-10 × $10^6$ cells for each resistant pool. Additional colonies were collected by seeding a pool of resistant cells on 96-well culture plates at 0.5 cells per well.

**PCR and sequencing for insert site identification.** Genomic DNA was prepared using Qiagen DNeasy Blood & Tissue kit. 170 ng of genomic DNA was digested using 2 units of Csp6I in 15 µl reaction at 37°C for 2 hours, followed by 65°C heat inactivation for 20 minutes, then ligated to 10 picomole indexed adapters with 100 units of T4 DNA ligase in 20 µl reaction at 16°C overnight. Double-stranded adapters were generated by annealing two sets of oligonucleotides (GTAATACGACTCACTATAGGGCACGCGTGGTCGACTGCGCATDDDDDDC and TAGUUUUUUATGCGCAGTTTTTTTGCAAAAA) whereas DDDDDD and UUUUUU denote complementary nucleotides for sample indexing [1]. Genomic DNA flanking the insert was amplified by two rounds of PCRs, with the first round in a 50 µl reaction with primers GTAATACGACTCACTATAGGGCACG and CGCTATTTAGAAAGAGAGAGCAATATTTCA respectively matching the adapters and the PB 5'-LTR, and with 0.2 µl PlatinumTaq DNA polymerase. Thermo-cycling conditions were 94°C/3'; 10 cycles of 94°C/15", 72°C/30" with -1°C/cycle touchdown, 72°C/1'; 20 cycles of 94°C/15", 62°C/30", 72°C/1'; followed by 72°C/2' extension. Second PCR was performed in a

10 µl reaction with 0.2 µl of first-round PCR product input and nested primers AATGATACGGCGACCACCGACACTCTactatagggcacgcgtggt and CAAGCAGAAGACGGCATACGAGCTCTTCagaatgcatgcgtcaattttacgcagac whereas uppercases matched Illumina P5 and P7 adapters and lowercases matched the first round of amplicons. Indexed PCR products were pooled, purified using Qiagen PCR purification columns and diluted to 2.5 ng/µl for Illumina sequencing. Up to 10 samples from resistant pools or 96 samples from clones were combined to be sequenced in a single channel. Sequencing was performed for single-end 50-cycle reactions (SR50) with custom sequencing primer ACTATAGGGCACGCGTGGTCGACTGCGCAT, following standard protocol except that sample loading density was reduced by half to avoid over-clustering.

**Lentiviral cDNA plasmid construction.** ORF (open reading frame) entry clones of *TRPS1* (HsCD00365530), *ESRRG* (HsCD00296449), *BRAF* (HsCD00379096), *ARHGEF28* (HsCD00295151), *YES1* (HsCD00376153), *DCN* (HsCD00044608), *WWTR1* (HsCD00045395), *RAPGEF6* (HsCD00379344), *USP25* (HsCD00372810), *SOX5* (HsCD00000303), *EYA1* (HsCD00376948), *NEDD4L*-dWW2 (HsCD00045367), *POU2F1* (HsCD00365256), *ZFHX3* (HsCD00365522), and *AKAP13* (HsCD00399180) were obtained from DF/HCC DNA Resource Core (http://horfdb.dfci.harvard.edu/index.php?page=home), and subcloned to lentiviral vectors pLENTI6.2/V5-DEST (Thermo Fisher Scientific) and pLENTI-puromycin (Addgene #39481) using Gateway L/R cloning. Both ends of the ORFs in the destination plasmids were verified by Sanger sequencing. cDNA plasmids of *NEDD4L*-WT (#27000), DD (#27001), SASA (#27003), S382A (#27036), and S468A (#27037) were obtained from Addgene [2]. *NEDD4L* ORFs were PCR-amplified using primers CACCATGGATTATCCGTATGATGTTC and TTAATCCACCCCTTCAAATCCTTG, subcloned to the entry vector pENTR/SD/D-TOPO (Thermo Fisher Scientific), and then to lentiviral vectors using Gateway L/R cloning. Full sequences of *NEDD4L* ORFs in lentiviral vectors were verified by sequencing. *ABCB1* plasmid purchased from Origene was amplified with primers CACCATGGATCTTGAAGGGGACCG and TCACTGGCGCTTTGTTCCAG and similarly subcloned to pENTR/SD/D-TOPO and then to lentiviral vectors.

**Cellular assays.** For short-term viability assays, cells were seeded in 96-well plates at 1250 cells per well in 100 µl growth media one day before treatment with 11-point/2-fold dilution series of compounds and assayed by adding 50 µl/well CellTiter-Glo (Promega) 5 days post treatment.

For apoptosis assays, caspase 3/7 (Promega) was added 24 hours after drug treatment, and signal was normalized to untreated cells. For cell growth assays, cells were plated at 1250 cells per well in 96-well plates, treated by compound one day after seeding, and assayed by CellTiter-Glo every day. For clonogenic assays, cells were plated at low density in clear 6-well or 96-well plates and drug-treated for 14 days, then were either stained with 1% Methylene Blue in 70% ethanol for 30 minutes or fixed with 12% glutaraldehyde for ten minutes then stained with 0.1% Crystal Violet for 30 minutes.

**qPCR.** Total RNA was prepared using Qiagen RNeasy mini kit and reverse-transcribed using iScript Supermix (Biorad) in 20 µl reactions. Quantitative PCR was performed with iTaq Universal SYBR Green Supermix using the Roche LightCycler LC480 system. Primers for each gene are listed below:

*TRPS1*: AGCCCCAGTAAGGGAGGAAA; GGGTGCAGGCCATATCTTGAG.
*ESRRG*: GCCCTCACTACACTGTGTGAC; CCTGCTAATTTGGACTGGTCTT.
*BRAF*: AATACACCAGCAAGCTAGATGC; AATCAGTTCCGTTCCCCAGAG.
*ARHGEF28*: AGGTGATGAAGTCTACGCTAACT; AGTGGCAGTGATTCCCTCTAT.
*YES1*: GCCTGTCAGTACAAGTGTGAG; AAAGGCGTTACCCCTGAGGAT.
*DCN*: ATGAAGGCCACTATCATCCTCC; GTCGCGGTCATCAGGAACTT.
*WWTR1*: GATCCTGCCGGAGTCTTTCTT; CACGTCGTAGGACTGCTGG.
*RAPGEF6*: CAGGCGTTGAGGAAGAAGC; TCTGGCAATCGTTTCTGAACAA.
*USP25*: GCACCAGCAGACGTTTTTGAA; AGCATTCTTCGCAGTAAGGAAA.
*SOX5*: CAGCCAGAGTTAGCACAATAGG; CTGTTGTTCCCGTCGGAGTT.
*EYA1*: CACCACAGATTTACCCTTCCAAC; GTACGTGGCATAGGCTGTAGC.
*NEDD4L*: GACATGGAGCATGGATGGGAA; GTTCGGCCTAAATTGTCCACT.
*POU2F1*: ATGAACAATCCGTCAGAAACCAG; GATGGAGATGTCCAAGGAAAGC.
*ZFHX3*: CAAGTTCACGACGGACAACCT; GCTTGCACTGGTATGAGTCCC.
*AKAP13*: GTCAACGGGCACACTTTCAG; GGAGGCTAGACTTTCTCGGC.

**siRNA transfection.** For samples used in the immunoblotting assays, cells were suspended in growth media at $1\times10^5$ cells/ml, and 2 ml of cells were reverse-transfected with 100 nM siRNA, 20 µl Lipofectamine RNAiMax (Thermo Fisher), and 0.5 ml OPTI-MEM in 6-well culture plates. After 48 hours of transfection, cells were treated with inhibitors for an additional 48 hours and cell lysates were collected. For samples used for viability assays, 100 µl of cells were

transfected with 100 nM of siRNA, 0.2 µl Lipofectamine RNAiMax, and 25 µl OPTI-MEM in 96-well microplates. After 24 hours, cells were treated with inhibitors for 72 hours before subjecting to CellTiter-Glo analysis.

**Immunoblotting.** Cultured cells were lysed on plates with cell lysis buffer (20 mM Tris, pH7.4, 150 mM NaCl, 1% Nonidet-P40 (Sigma-Aldrich), 1 mM EDTA, 1 mM EGTA, 10% glycerol), and quantified by Pierce BCA protein assay. Samples were balanced in NuPAGE LDS sample buffer (Thermo Fisher Scientific) and reducing agent, fractionated in 4-12% Bis-Tris SDS-PAGE gel, and transferred to polyvinylidene fluoride membranes. Samples were blocked with 5% non-fat milk and blotted in primary antibodies at 4°C overnight. Membranes were washed in TBST (50 mM Tris, pH7.6, 150 mM NaCl, 0.1% Tween 20) three times. Secondary antibodies were used at 1:20000 dilution followed by washing in TBST. Signals were detected using SuperSignal West Femto substrate (Thermo Fisher) and imaged with a G-box imager (Syngene). To re-probe, membranes were treated with stripping buffer (GM Biosciences) and then processed as above. Phospho-MEK (Ser217/221) (#9154), phospho-ERK (Thr202/204) (#9101), total ERK (#9102), phospho-AKT (Thr308) (#4056), phospho-AKT (Ser473) (#4060), phospho-S6K (Thr389) (#9205), phospho-S6 (Ser235/236) (#2211), phospho-4EBP (Thr37/46) (#2855), GAPDH (#2118), phopho-Gab1 (Tyr627) (#3233), phospho-IGF1Rβ (Tyr1135/1136) (#3024), TAZ (#2149), phosphor-SMAD2/3 (#9510), total SMAD2/3 (#8685), TEAD1 (#8526), Phospho-YAP1 (Ser127) (#4911), BRAF (#9002), and YES1 (#3201) antibodies were purchased from Cell Signaling Technology. NEDD4L (ab46521), ESRRG (ab49129), POU2F1 (ab15112), and phospho-EGFR (Tyr1068) (ab5644) were from Abcam. Total GAB1 (sc-9049) and total EGFR (sc-03) were from Santa Cruz Biotechnology. Phospho-RSK (Thr359/Ser363) (04-419) was from Millipore. YAP antibody (#13584) was from Proteintech. Peroxidase-conjugated goat anti-rabbit IgG (#31460) and anti-mouse IgG (#31430) were from Thermo Scientific.

**Permutation tests for STRING analysis.** We performed significance tests (permutation tests) to test whether the null hypothesis ($H_0$: there is no relationship between the two genesets) could be rejected. Permutation tests were set so that an equal number of random genes to the number of query screen genes were drawn from the query library and projected to the object geneset with the same STRING criteria used to test connections between hit lists. The direction of STRING analysis from queries to objects are indicated as arrows in corresponding graphs. Random draw was performed using the initial libraries corresponding to: 169 genes out of 14,457 candidate

genes for the ORF screen[3], 31 genes out of 16,601 for the shRNA screen[4], 263 genes out of of 18,080 for the CRISPR screen[5], and 186 out of 23,430 candidates for the dCAS9 library[6]. The number of permutations varied between $10^5$ and $10^6$ to confer statistical power. STRING and permutation tests were also performed between screened genesets and a set of MAPK pathway components (GRB2, SOS1, KRAS, NRAS, BRAF, CRAF, MEK, ERK, S6K, RSK, CREB1, FOS, JUN), with the above library background for each screen geneset plus all RefSeq genes as the library background for the transposon geneset. These analyses were run using scripts written both in R and Matlab (for random permutations, the built-in 'randperm' Matlab function was used). p-values were calculated as the number of random instances reaching the connectivity level vs. total permutations.

**Patient samples.** Patients with metastatic melanoma containing $BRAF^{V600E}$ mutation enrolled on clinical trials for treatment with a BRAF inhibitor vemurafenib or combined BRAF + MEK inhibitor (dabrafenib + trametinib or LGX818 + MEK162) were reported previously [7]. Tumor biopsies were performed pre-treatment (day 0), at 10-14 days on treatment, and/or at time of progression if applicable. Formalin-fixed tissue was analyzed to confirm that viable tumor was present via hematoxylin and eosin (H&E) staining. Additional tissue was snap-frozen and stored in liquid nitrogen or was immediately processed for purification of RNA. To assess therapy response, patients underwent computed tomography every 3 months. Responses were determined according to the Response Evaluation Criteria in Solid Tumors (RECIST), version 1.1. Samples were homogenized and disrupted using a mortar and pestle followed by use of a QIAshredder (Qiagen). A QIAcube was used to harvest RNA using the RNeasy Mini Protocol (Qiagen). Starting with about 700 ng of total RNA, poly-A containing mRNA molecules were converted into libraries suitable for next generation sequencing approaches using reagents and the protocol provided by Illumina TruSeq RNA Sample Prep Kit v2, and sequenced paired-end 2x75 bp using HiSeq 2000.


1. Chen, L., et al., *Transposon activation mutagenesis as a screening tool for identifying resistance to cancer therapeutics.* BMC Cancer, 2013. **13**(1): p. 93.
2. Gao, S., et al., *Ubiquitin ligase Nedd4L targets activated Smad2/3 to limit TGF-beta signaling.* Mol Cell, 2009. **36**(3): p. 457-68.
3. Johannessen, C.M., et al., *A melanocyte lineage program confers resistance to MAP kinase pathway inhibition.* Nature, 2013. **504**(7478): p. 138-42.

4. Whittaker, S.R., et al., *A genome-scale RNA interference screen implicates NF1 loss in resistance to RAF inhibition.* Cancer Discov, 2013. **3**(3): p. 350-62.
5. Shalem, O., et al., *Genome-scale CRISPR-Cas9 knockout screening in human cells.* Science, 2014. **343**(6166): p. 84-7.
6. Konermann, S., et al., *Genome-scale transcriptional activation by an engineered CRISPR-Cas9 complex.* Nature, 2015. **517**(7536): p. 583-8.
7. Kwong, L.N., et al., *Co-clinical assessment identifies patterns of BRAF inhibitor resistance in melanoma.* J Clin Invest, 2015. **125**(4): p. 1459-70.

**SUPPLEMENTARY TEXT**

>pPB-SB-CMV-puro plasmid with degenerate 5 bp barcodes 7101 bp

ATTCGCCCTATAGTGAGTCGTATTACAATTCACTGGCCGTCGTTTTACAACGTCGTGACTGGGAAAACCC
TGGCGTTACCCAACTTAATCGCCTTGCAGCACATCCCCCTTTCGCCAGCTGGCGTAATAGCGAAGAGGCC
CGCACCGATCGCCCTTCCCAACAGTTGCGCAGCCTGAATGGCGAATGGGACGCGCCCTGTAGCGGCGCAT
TAAGCGCGGCGGGTGTGGTGGTTACGCGCAGCGTGACCGCTACACTTGCCAGCGCCCTAGCGCCCGCTCC
TTTCGCTTTCTTCCCTTCCTTTCTCGCCACGTTCGCCGGCTTTCCCCGTCAAGCTCTAAATCGGGGGCTC
CCTTTAGGGTTCCGATTTAGTGCTTTACGGCACCTCGACCCCAAAAAACTTGATTAGGGTGATGGTTCAC
GTAGTGGGCCATCGCCCTGATAGACGGTTTTTCGCCCTTTGACGTTGGAGTCCACGTTCTTTAATAGTGG
ACTCTTGTTCCAAACTGGAACAACACTCAACCCTATCTCGGTCTATTCTTTTGATTTATAAGGGATTTTG
CCGATTTCGGCCTATTGGTTAAAAAATGAGCTGATTTAACAAAAATTTAACGCGAATTTTAACAAAATAT
TAACGCTTACAATTTAGGTGGCACTTTTCGGGGAAATGTGCGCGGAACCCCTATTTGTTTATTTTTCTAA
ATACATTCAAATATGTATCCGCTCATGAGACAATAACCCTGATAAATGCTTCAATAATATTGAAAAAGGA
AGAGTATGAGTATTCAACATTTCCGTGTCGCCCTTATTCCCTTTTTTGCGGCATTTTGCCTTCCTGTTTT
TGCTCACCCAGAAACGCTGGTGAAAGTAAAAGATGCTGAAGATCAGTTGGGTGCACGAGTGGGTTACATC
GAACTGGATCTCAACAGCGGTAAGATCCTTGAGAGTTTTCGCCCCGAAGAACGTTTTCCAATGATGAGCA
CTTTTAAAGTTCTGCTATGTGGCGCGGTATTATCCCGTATTGACGCCGGGCAAGAGCAACTCGGTCGCCG
CATACACTATTCTCAGAATGACTTGGTTGAGTACTCACCAGTCACAGAAAAGCATCTTACGGATGGCATG
ACAGTAAGAGAATTATGCAGTGCTGCCATAACCATGAGTGATAACACTGCGGCCAACTTACTTCTGACAA
CGATCGGAGGACCGAAGGAGCTAACCGCTTTTTTGCACAACATGGGGGATCATGTAACTCGCCTTGATCG
TTGGGAACCGGAGCTGAATGAAGCCATACCAAACGACGAGCGTGACACCACGATGCCTGTAGCAATGGCA
ACAACGTTGCGCAAACTATTAACTGGCGAACTACTTACTCTAGCTTCCCGGCAACAATTAATAGACTGGA
TGGAGGCGGATAAAGTTGCAGGACCACTTCTGCGCTCGGCCCTTCCGGCTGGCTGGTTTATTGCTGATAA
ATCTGGAGCCGGTGAGCGTGGGTCTCGCGGTATCATTGCAGCACTGGGGCCAGATGGTAAGCCCTCCCGT
ATCGTAGTTATCTACACGACGGGGAGTCAGGCAACTATGGATGAACGAAATAGACAGATCGCTGAGATAG
GTGCCTCACTGATTAAGCATTGGTAACTGTCAGACCAAGTTTACTCATATATACTTTAGATTGATTTAAA
ACTTCATTTTTAATTTAAAAGGATCTAGGTGAAGATCCTTTTTGATAATCTCATGACCAAAATCCCTTAA
CGTGAGTTTTCGTTCCACTGAGCGTCAGACCCCGTAGAAAAGATCAAAGGATCTTCTTGAGATCCTTTTT
TTCTGCGCGTAATCTGCTGCTTGCAAACAAAAAAACCACCGCTACCAGCGGTGGTTTGTTTGCCGGATCA
AGAGCTACCAACTCTTTTTCCGAAGGTAACTGGCTTCAGCAGAGCGCAGATACCAAATACTGTTCTTCTA
GTGTAGCCGTAGTTAGGCCACCACTTCAAGAACTCTGTAGCACCGCCTACATACCTCGCTCTGCTAATCC
TGTTACCAGTGGCTGCTGCCAGTGGCGATAAGTCGTGTCTTACCGGGTTGGACTCAAGACGATAGTTACC
GGATAAGGCGCAGCGGTCGGGCTGAACGGGGGGTTCGTGCACACAGCCCAGCTTGGAGCGAACGACCTAC
ACCGAACTGAGATACCTACAGCGTGAGCTATGAGAAAGCGCCACGCTTCCCGAAGGGAGAAAGGCGGACA

GGTATCCGGTAAGCGGCAGGGTCGGAACAGGAGAGCGCACGAGGGAGCTTCCAGGGGGAAACGCCTGGTA
TCTTTATAGTCCTGTCGGGTTTCGCCACCTCTGACTTGAGCGTCGATTTTTGTGATGCTCGTCAGGGGGG
CGGAGCCTATGGAAAAACGCCAGCAACGCGGCCTTTTTACGGTTCCTGGCCTTTTGCTGGCCTTTTGCTC
ACATGTTCTTTCCTGCGTTATCCCCTGATTCTGTGGATAACCGTATTACCGCCTTTGAGTGAGCTGATAC
CGCTCGCCGCAGCCGAACGACCGAGCGCAGCGAGTCAGTGAGCGAGGAAGCGGAAGAGCGCCCAATACGC
AAACCGCCTCTCCCCGCGCGTTGGCCGATTCATTAATGCAGCTGGCACGACAGGTTTCCCGACTGGAAAG
CGGGCAGTGAGCGCAACGCAATTAATGTGAGTTAGCTCACTCATTAGGCACCCCAGGCTTTACACTTTAT
GCTTCCGGCTCGTATGTTGTGTGGAATTGTGAGCGGATAACAATTTCACACAGGAAACAGCTATGACCAT
GATTACACCAAGCTCGAAATTAACCCTCACTAAAGGGAACAAAAGCTGGAGCTAGGGATAACAGGGTAAT
GGCGCGCCTTAATTAACTATAACGGTCCTAAGGTAGCGAGCTCGGTATTCACGACAGCAGGCTGAATAAT
AAAAAAATTAGAAACTATTATTTAACCCTAGAAAGATAATCATATTGTGACGTACGTTAAAGATAATCAT
GCGTAAAATTGACGCATGTGTTTTATCGGTCTGTATATCGAGGTTTATTTATTAATTTGAATAGATATTA
AGTTTTATTATATTTACACTTACATACTAATAATAAATTCAACAAACAATTTATTTATGTTTATTTATTT
ATTAAAAAAAAACAAAAACTCAAAATTTCTTCTATAAAGTAACAAAACTTTTAAACATTCTCTCTTTTAC
AAAAATAAACTTATTTTGTACTTTAAAAACAGTCATGTTGTATTATAAAATAAGTAATTAGCTTAACCTA
TACATAATAGAAACAAATTATACTTATTAGTCAGTCAGAAACAACTTTGGCACATATCAATATTATGCTC
TCGTTAATCGCCGAGCTACAGTTGAAGTCGGAAGTTTACATACACTTAAGTTGGAGTCATTAAAACTCGT
TTTTCAACTACTCCACAAATTTCTTGTTAACAAACAATAGTTTTGGCAAGTCAGTTAGGACATCTACTTT
GTGCATGACACAAGTCATTTTTCCAACAATTGTTTACAGACAGATTATTTCACTTATAATTCACTGTATC
ACAATTCCAGTGGGTCAGAAGTTTACATACACTAAGTTGACTGTGCCTTTAAACAGCTTGGAAAATTCCA
GAAAATGATGTCATGGCTTTAGAAGCTTCACGCTGCCGCAAGCACTCAGGGCGCAAGGGCTGCTAAAGGA
AGCGGAACACGTAGAAAGCCAGTCCGCAGAAACGGTGCTGACCCCGGATGAATGTCAGCTACTGGGCTAT
CTGGACAAGGGAAAACGCAAGCGCAAAGAGAAAGCAGGTAGCTTGCAGTGGGCTTACATGGCGATAGCTA
GACTGGGCGGTTTTATGGACAGCAAGCGAACCGGAATTGCCAGCTGGGGCGCCCTCTGGTAAGGTTGGGA
AGCCCTGCAAAGTAAACTGGATGGCTTTCTTGCCGCCAAGGATCTGATGGCGCAGGGGATCAAGATCCTG
CTTCATCCCCGTGGCCCGTTGCTCGCGTTTGCTGGCGGTGTCCCCGGAAGAAATATATTTGCATGTCTTT
AGTTCTATGATGACACAAACCCCGCCCAGCGTCTTGTCATTGGCGAATTCGAACACGCAGATGCAGTCGG
GGCGGCGCGGTCCCAGGTCCACTTCGCATATTAAGGTGACGCGTGTGGCCTCGAACACCGAGCGACCCTG
CAGCGACCCGCTTAACAGCGTCAACAGCGTGCCGCAGATCTTACCATGACCGAGTACAAGCCCACGGTGC
GCCTCGCCACCCGCGACGACGTCCCCAGGGCCGTACGCACCCTCGCCGCCGCGTTCGCCGACTACCCCGC
CACGCGCCACACCGTCGATCCGGACCGCCACATCGAGCGGGTCACCGAGCTGCAAGAACTCTTCCTCACG
CGCGTCGGGCTCGACATCGGCAAGGTGTGGGTCGCGGACGACGGCGCCGCGGTGGCGGTCTGGACCACGC
CGGAGAGCGTCGAAGCGGGGGCGGTGTTCGCCGAGATCGGCCCGCGCATGGCCGAGTTGAGCGGTTCCCG
GCTGGCCGCGCAGCAACAGATGGAAGGCCTCCTGGCGCCGCACCGGCCCAAGGAGCCCGCGTGGTTCCTG
GCCACCGTCGGCGTCTCGCCCGACCACCAGGGCAAGGGTCTGGGCAGCGCCGTCGTGCTCCCCGGAGTGG

AGGCGGCCGAGCGCGCCGGGGTGCCCGCCTTCCTGGAGACCTCCGCGCCCCGCAACCTCCCCTTCTACGA
GCGGCTCGGCTTCACCGTCACCGCCGACGTCGAGGTGCCCGAAGGACCGCGCACCTGGTGCATGACCCGC
AAGCCCGGTGCCTGACGCCCGGGAGATGGGGGAGGCTAACTGAAACACGGAAGGAGACAATACCGGAAGG
AACCCGCGCTATGACGGCAATAAAAAGACAGAATAAAACGCACGGTGTTGGGTCGTTTGTTCATAAACGC
GGGGTTCGGTCCCAGGGCTGGCACTCTGTCGATACCCCACCGAGACCCCATTGGGGCCAATACGCCCGCG
TTTCTTCCTTTTCCCCACCCCACCCCCCAAGTTCGGGTGAAGGCCCAGGGCTCGCAGCCAACGTCGGGGC
GGCAAGCCCTGCCATAGCCACGGCCCCGTGGGTTAGGGACGGCGGATCGCGGCCCTCTAGAGAGCTTGG
CCCATTGCATACGTTGTATCCATATCATAATATGTACATTTATATTGGCTCATGTCCAACATTACCGCCA
TGTTGACATTGATTATTGACTAGTTATTAATAGTAATCAATTACGGGGTCATTAGTTCATAGCCCATATA
TGGAGTTCCGCGTTACATAACTTACGGTAAATGGCCCGCCTGGCTGACCGCCCAACGACCCCCGCCCATT
GACGTCAATAATGACGTATGTTCCCATAGTAACGCCAATAGGGACTTTCCATTGACGTCAATGGGTGGAG
TATTTACGGTAAACTGCCCACTTGGCAGTACATCAAGTGTATCATATGCCAAGTACGCCCCCTATTGACG
TCAATGACGGTAAATGGCCCGCCTGGCATTATGCCCAGTACATGACCTTATGGGACTTTCCTACTTGGCA
GTACATCTACGTATTAGTCATCGCTATTACCATGGTGATGCGGTTTTGGCAGTACATCAATGGGCGTGGA
TAGCGGTTTGACTCACGGGGATTTCCAAGTCTCCACCCCATTGACGTCAATGGGAGTTTGTTTTGGCACC
AAAATCAACGGGACTTTCCAAAATGTCGTAACAACTCCGCCCCATTGACGCAAATGGGCGGTAGGCGTGT
ACGGTGGGAGGTCTATATAAGCAGAGCTCGTTTAGTGAACCGTCAGATCGCCTGGAGACGCCATCCACGC
TGTTTTGACCTCCATAGAAGACACCGGGACCGATCCAGCCTCCGGTCGACCGATCCTGAGAACTTCAGGG
TGAGTTTGGGGACCCTTGATTGTTCTTTCTTTTTCGCTATTGTAAAATTCATGTTATATGGAGGGGGCAA
AGTTTTCAGGGTGTTGTTTAGAATGGGAAGATGTCCCTTGTATCACCATGGACCCTCATGATAATTTTGT
TTCTTTCACTTTCTACTCTGTTGACAACCATTGTCTCCTCTTATTTTCTTTTCATTTTCTGTAACTTTTT
CGTTAAACTTTAGCTTGCATTTGTAACGAATTTTTAAATTCACTTTTGTTTATTTGTCAGATTGTAAGCT
TGTGGAAGGCTACTCGAAATGTTTGACCCAAGTTAAACAATTTAAAGGCAATGCTACCAAATACTAATTG
AGTGTATGTTAACTTCTGACCCACTGGGAATGTGATGAAAGAAATAAAAGCTGAAATGAATCATTCTCTC
TACTATTATTCTGATATTTCACATTCTTAAAATAAAGTGGTGATCCTAACTGACCTTAAGACAGGGAATC
TTTACTCGGATTAAATGTCAGGAATTGTGAAAAAGTGAGTTTAAATGTATTTGGCTAAGGTGTATGTAAA
CTTCCGACTTCAACTGTATATCTAGCCAACAAGCTCGTCATCGCTTTGCAGAAGAGCAGAGAGGATATGC
TCATCGTCTAAAGAACTACCCATTTTATTATATATTAGTCACGATATCTATAACAAGAAAATATATATAT
AATAAGTTATCACGTAAGTAGAACATGAAATAACAATATAATTATCGTATGAGTTAAATCTTAAAAGTCA
CGTAAAAGATAATCATGCGTCATTTTGACTCACGCGGTCGTTATAGTTCAAAATCAGTGACACTTACCGC
ATTGACAAGCACGCCTCACGGGAGCTCCAAGCGGCGACTGAGATGTCCTAAATGCACAGCGACGGATTCG
CGCTATTTAGAAAGAGAGAGCAATATTTCAAGAATGCATGCGTCAATTTTACGCAGACTATNNNNNTAGG
GTTAAAAAAGATTTGCGCTTTACTCGACCTAAACTTTAAACACGTCATAGAATCTTCGTTTGACAAAAAC
CACATTGTGGCCAAGCTGTGTGACGCGACGCGCGCTAAAGAATGGCAAACCAAGTCGCGCGAGGTACCTC
TCTTAAGGTAGCGCGATCGCACGCGTACCCA

Figure 3D orginal

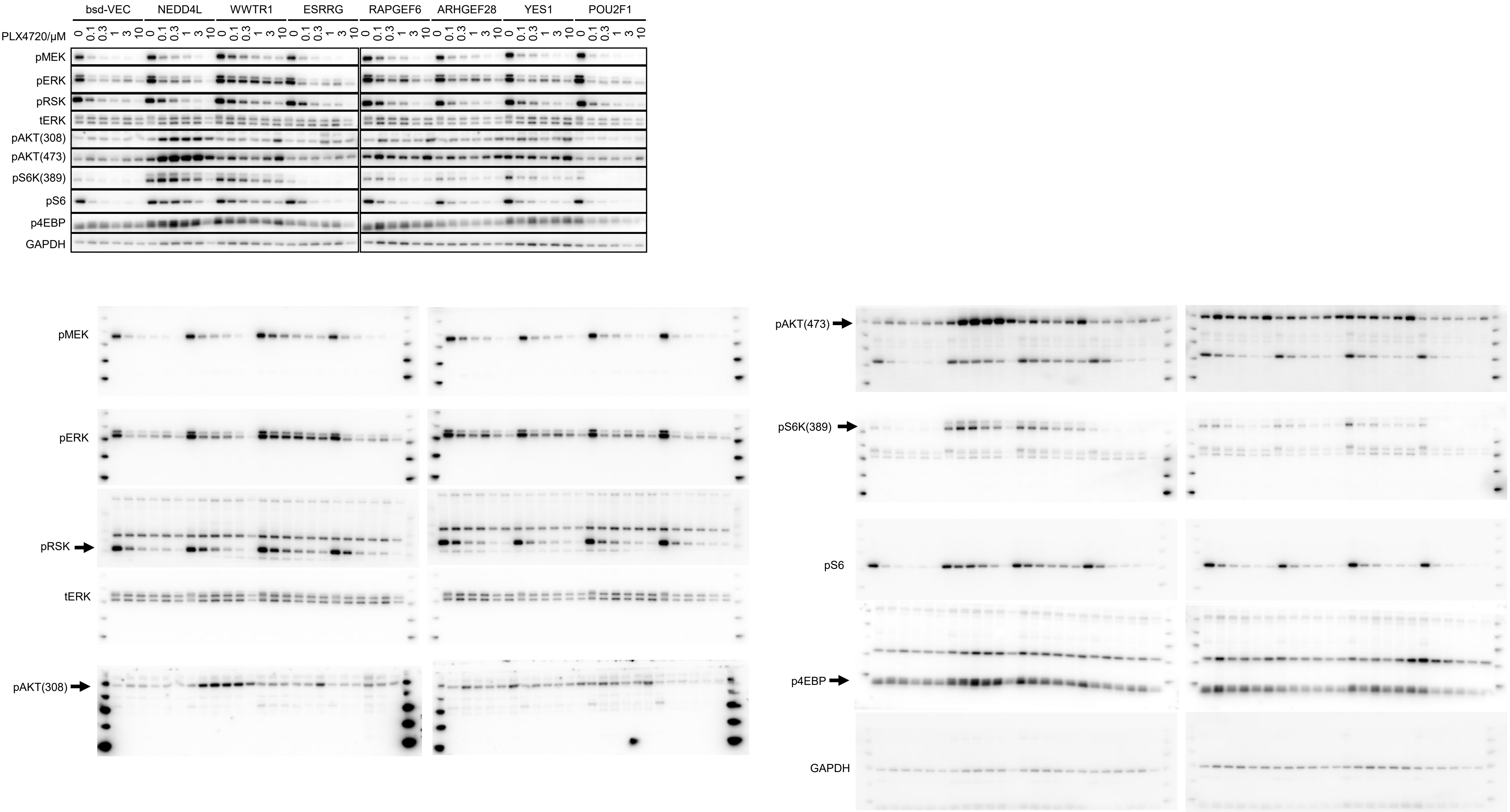

Figure 4B original

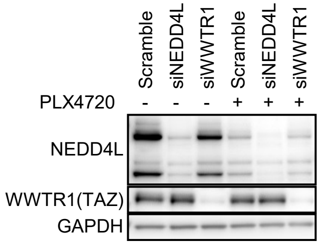


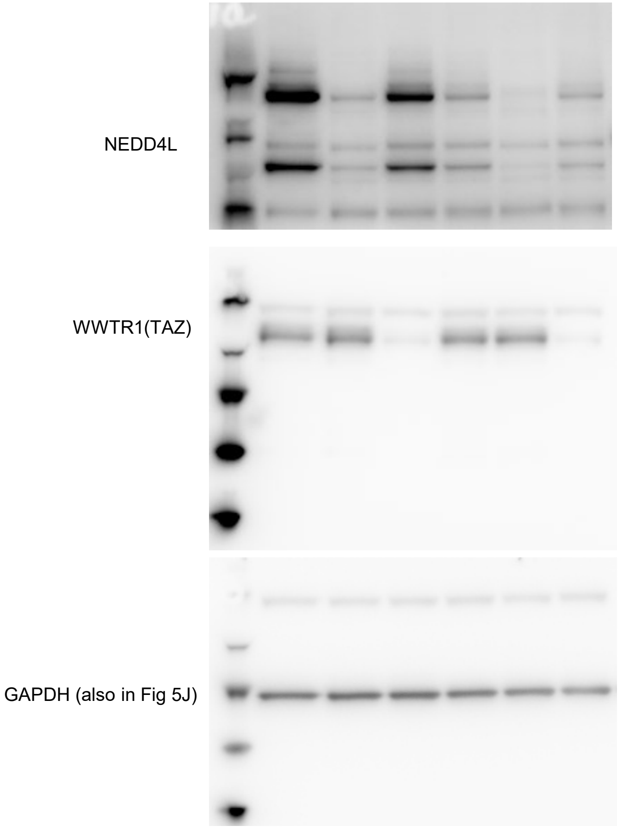

Figure 4D orginal

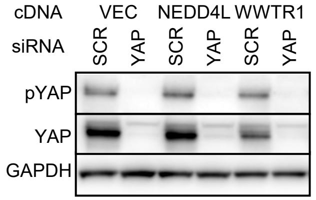


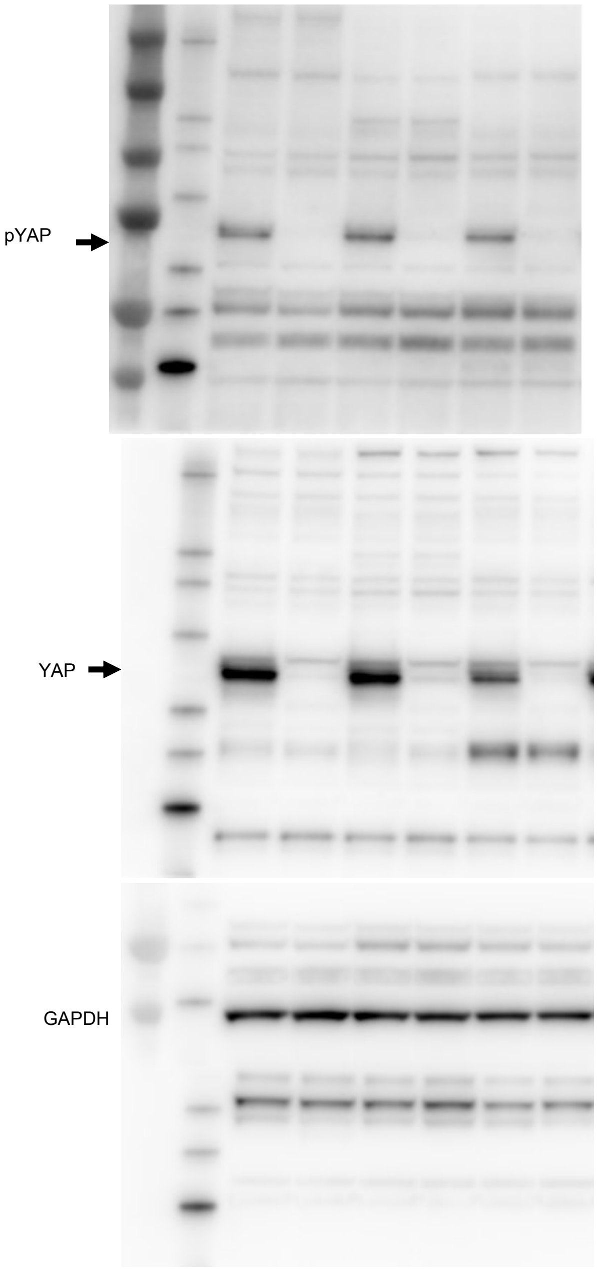

# Figure 4F original

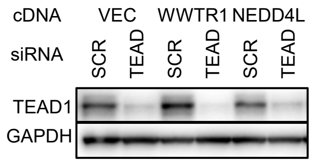


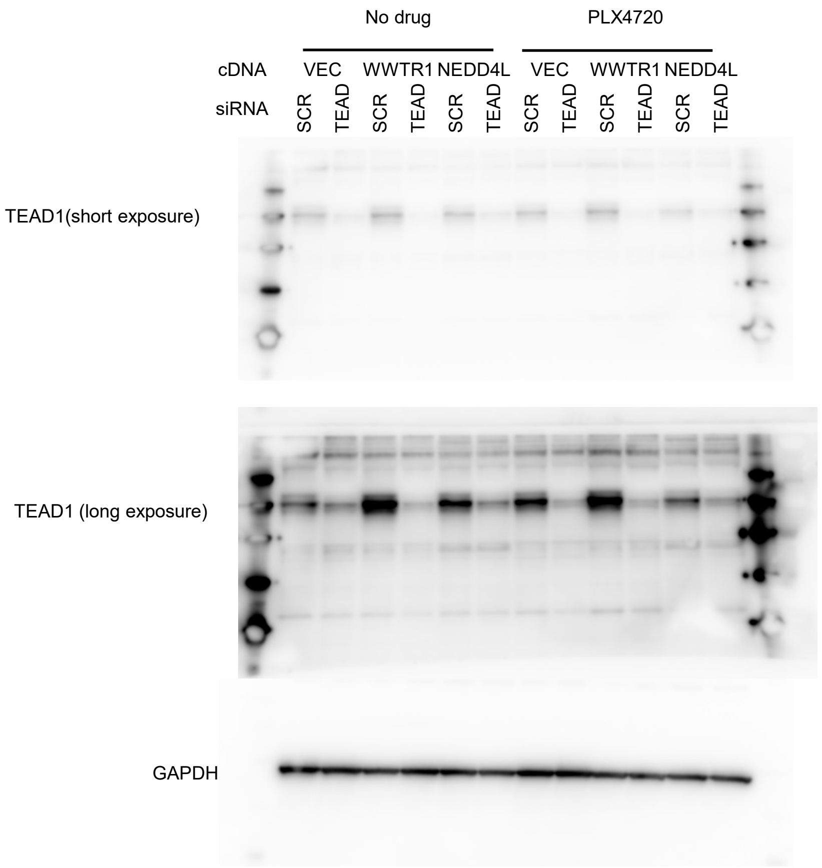

Figure 5C original

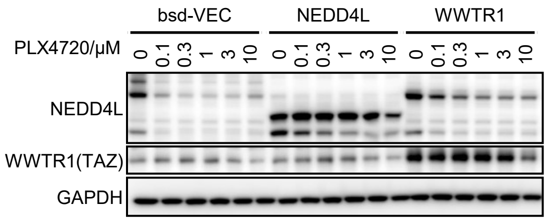


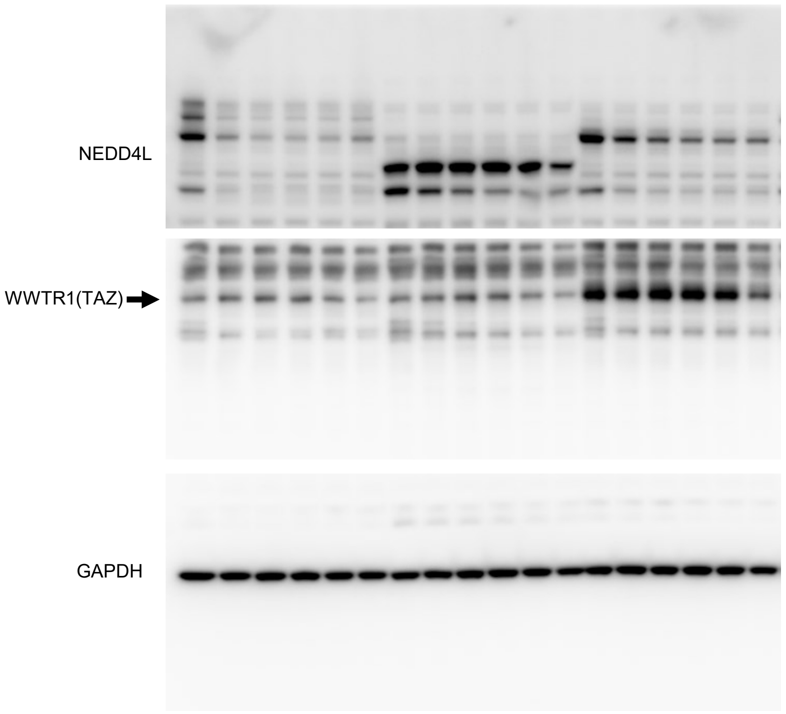

Figure 5F original

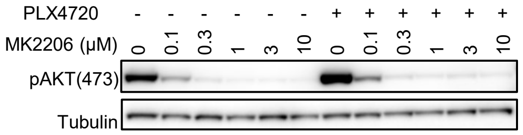


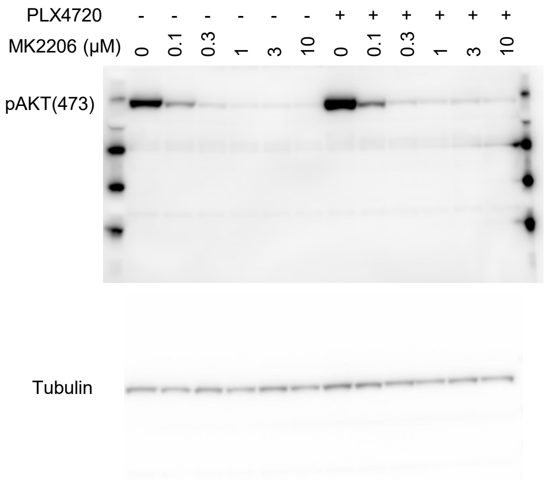

Figure 5H original

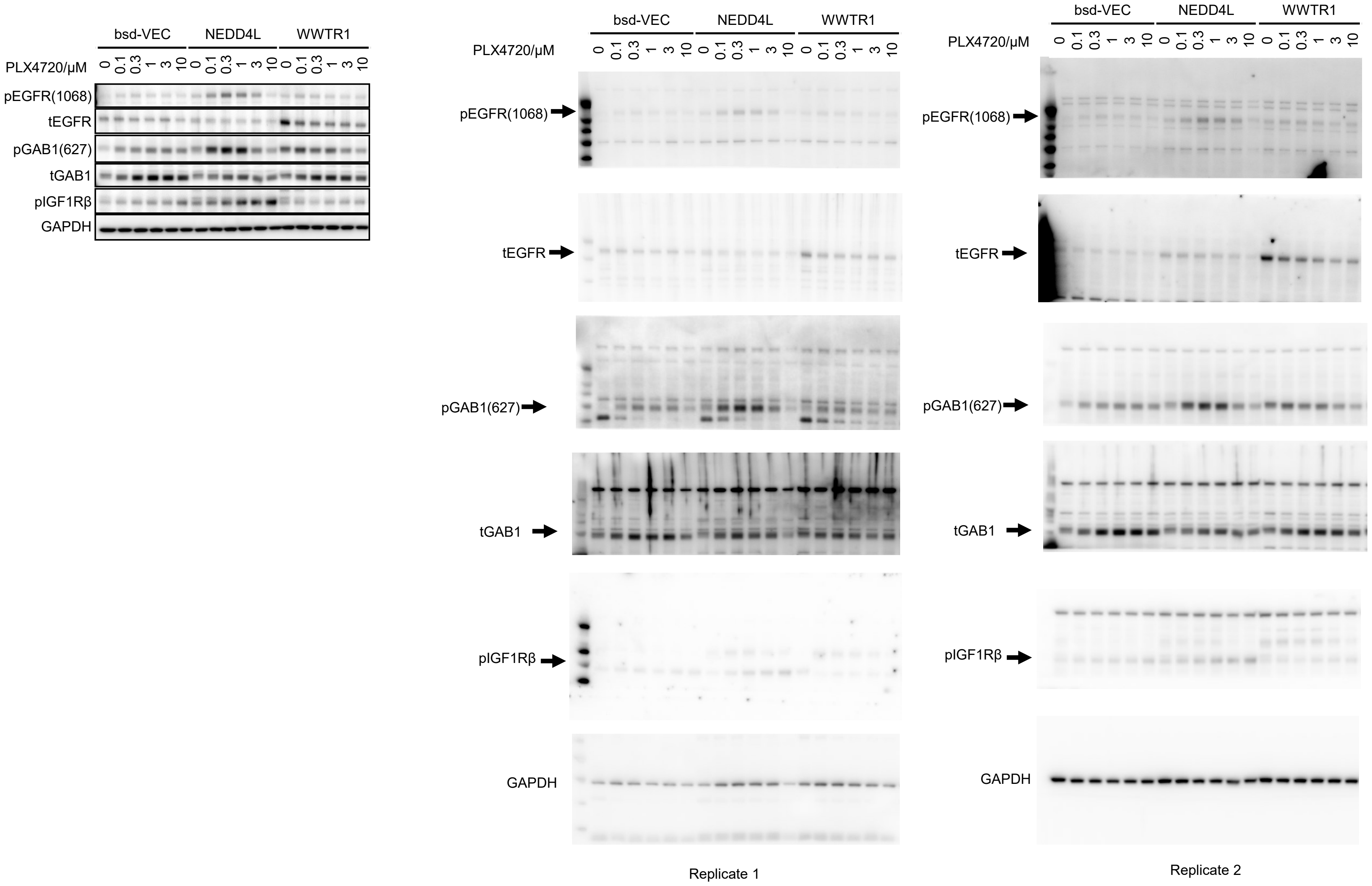

Figure 5J original

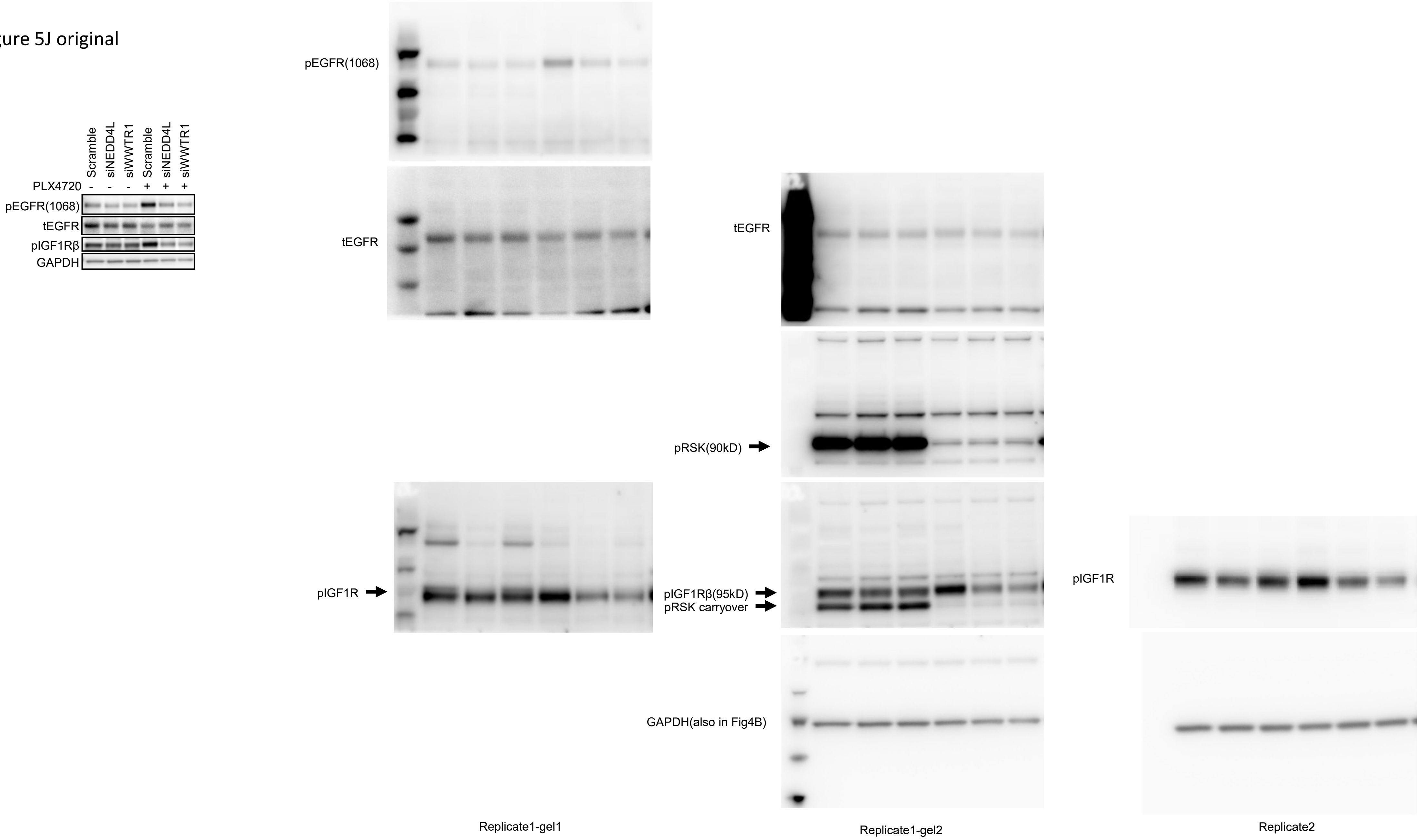

Figure S2D original

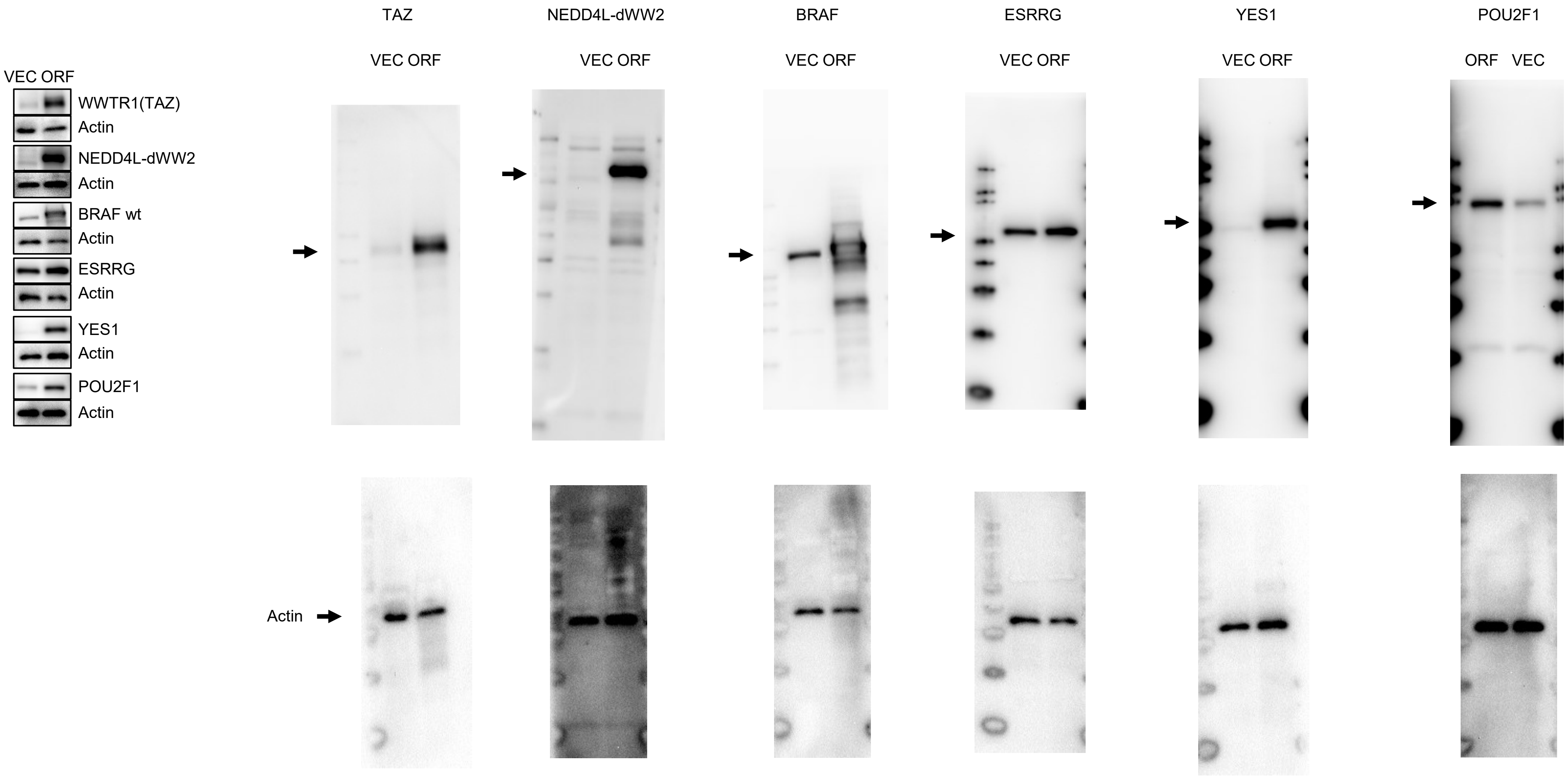

Figure S4A original

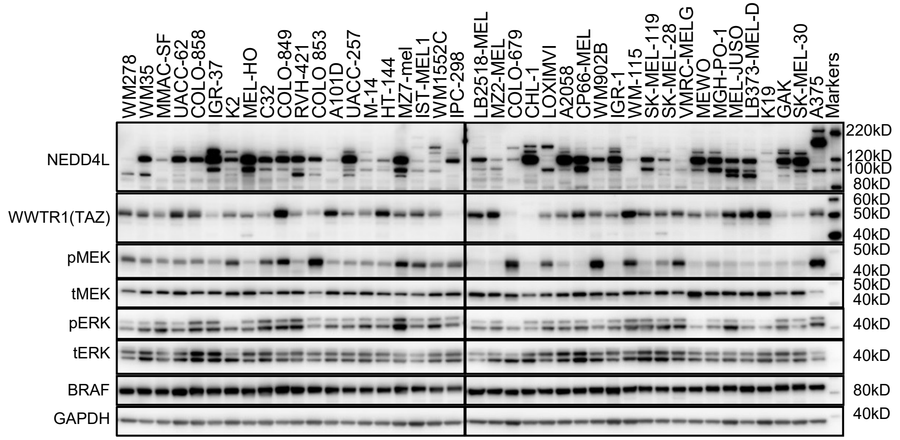


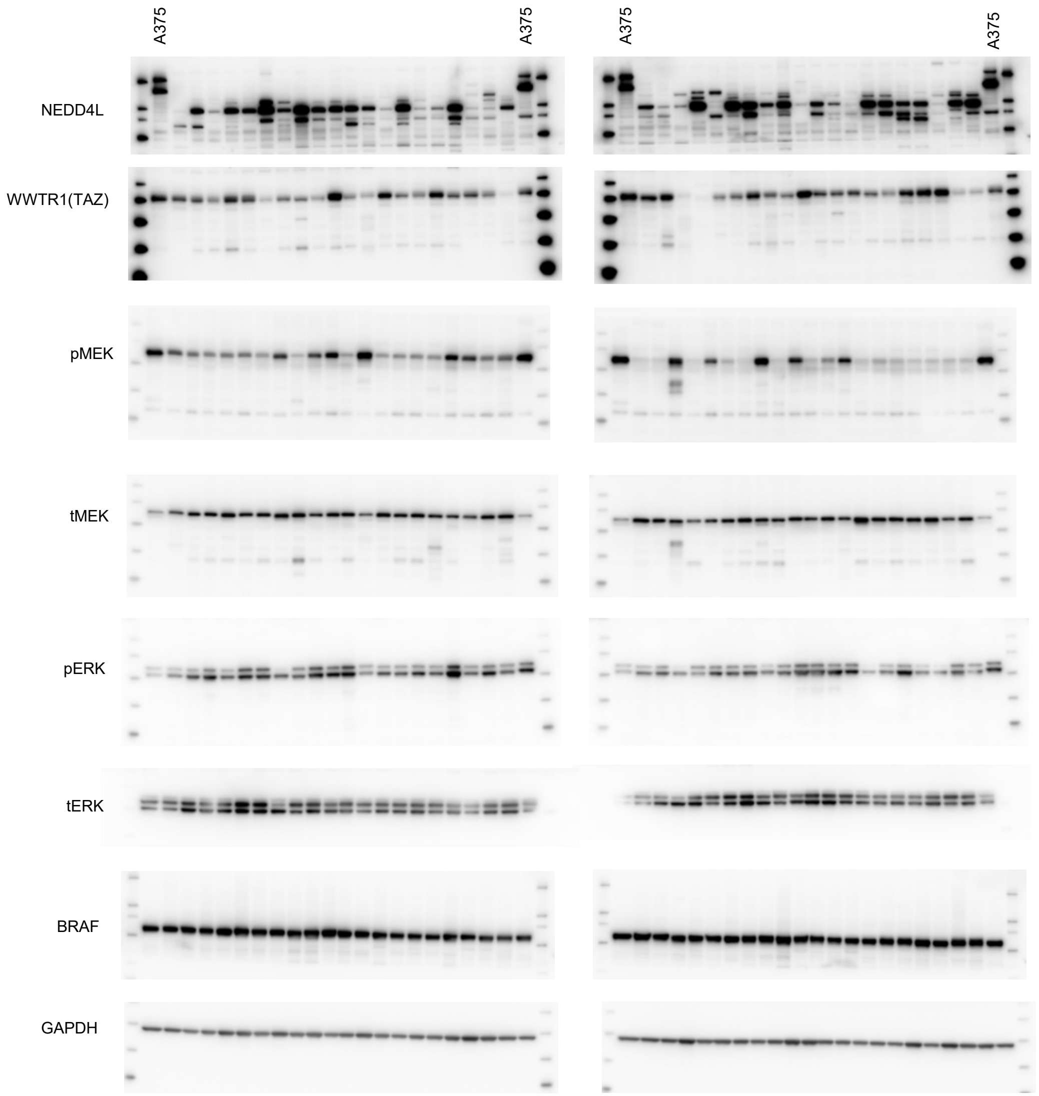

Figure S5A original

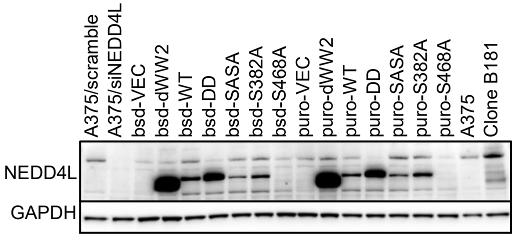


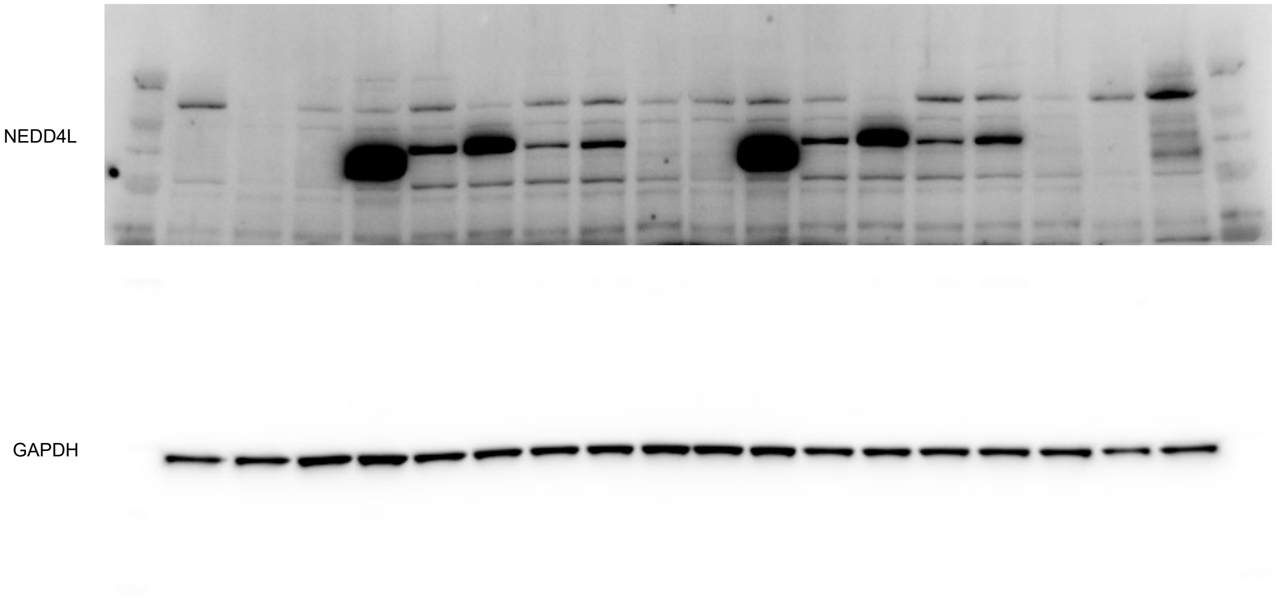

Figure S5E original

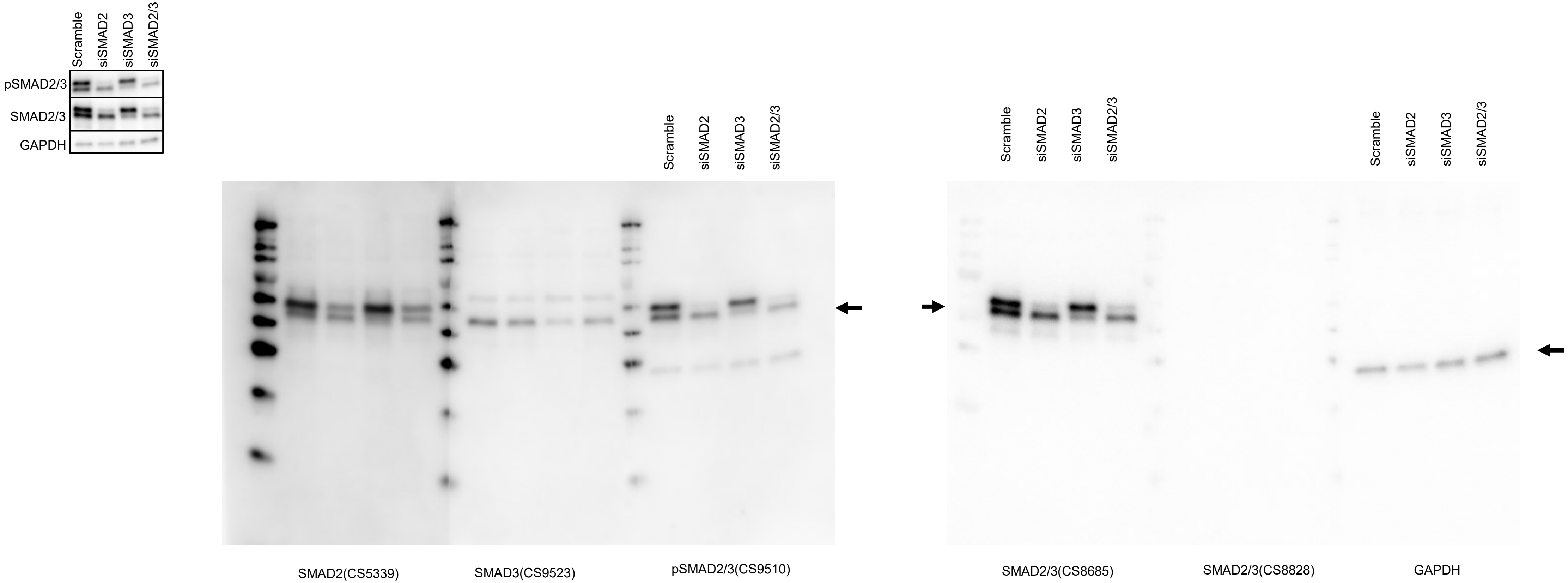

Figure S5F original

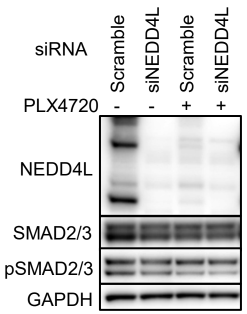


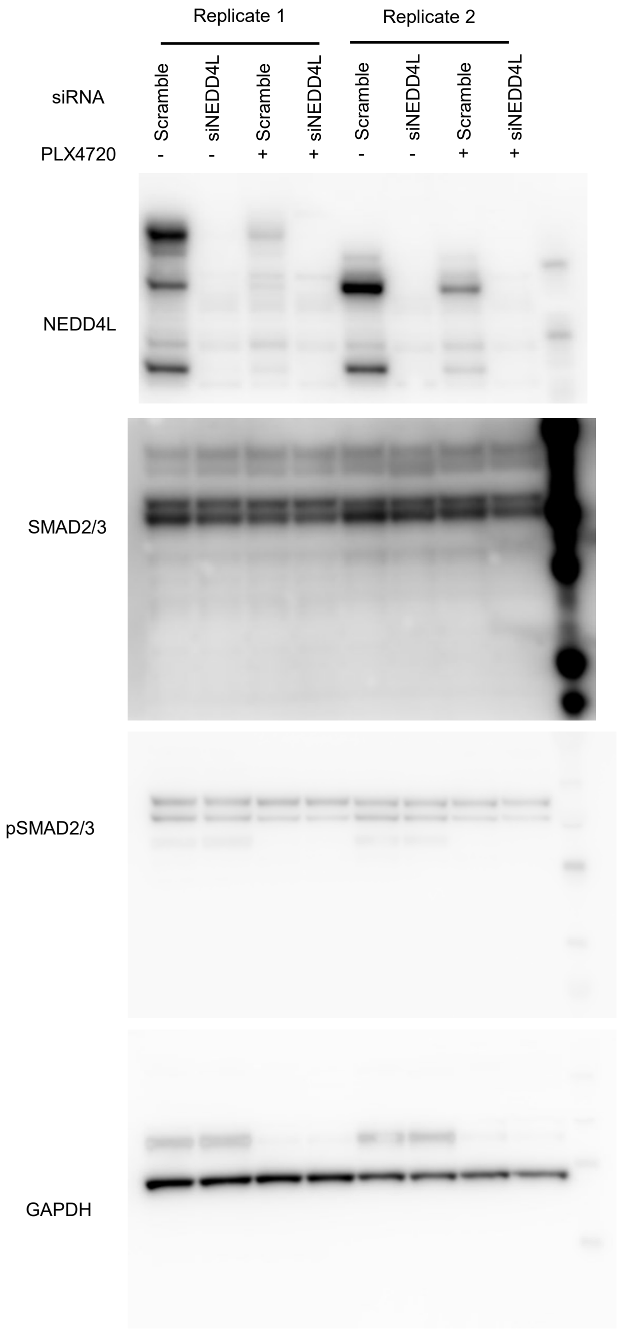

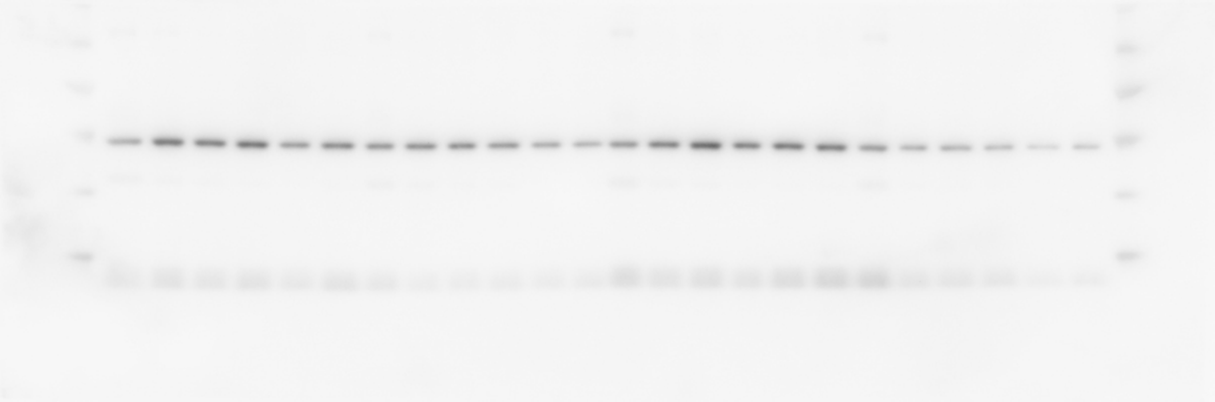

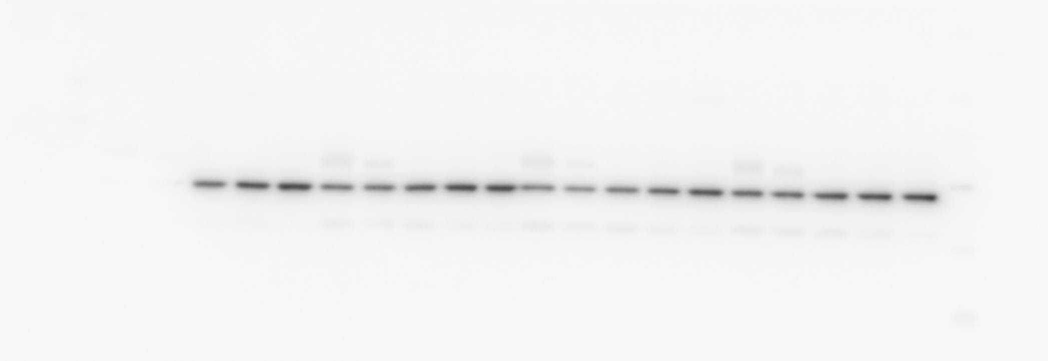

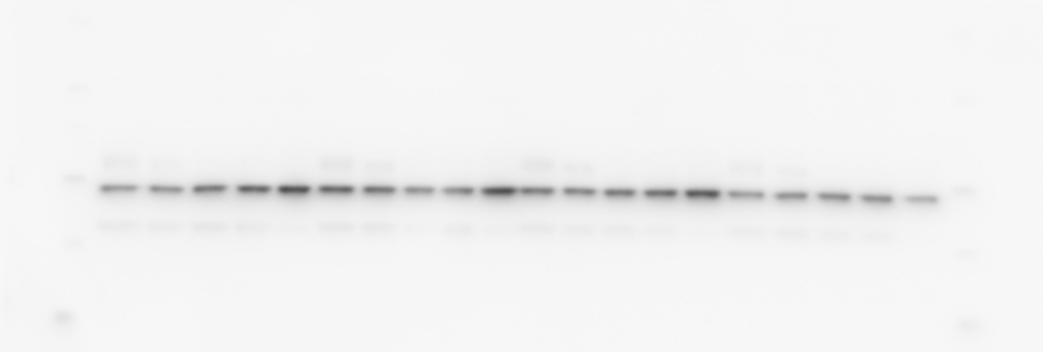

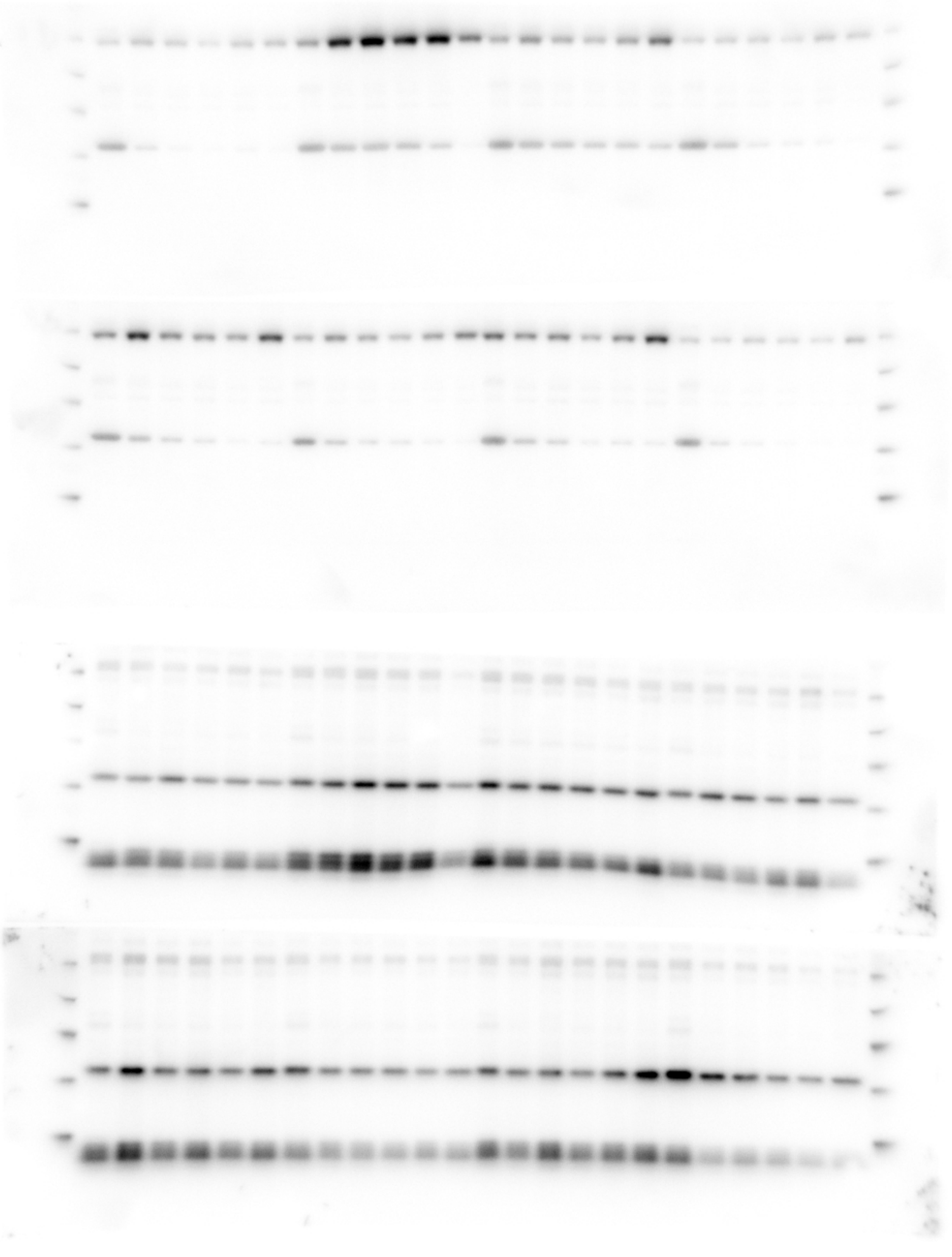

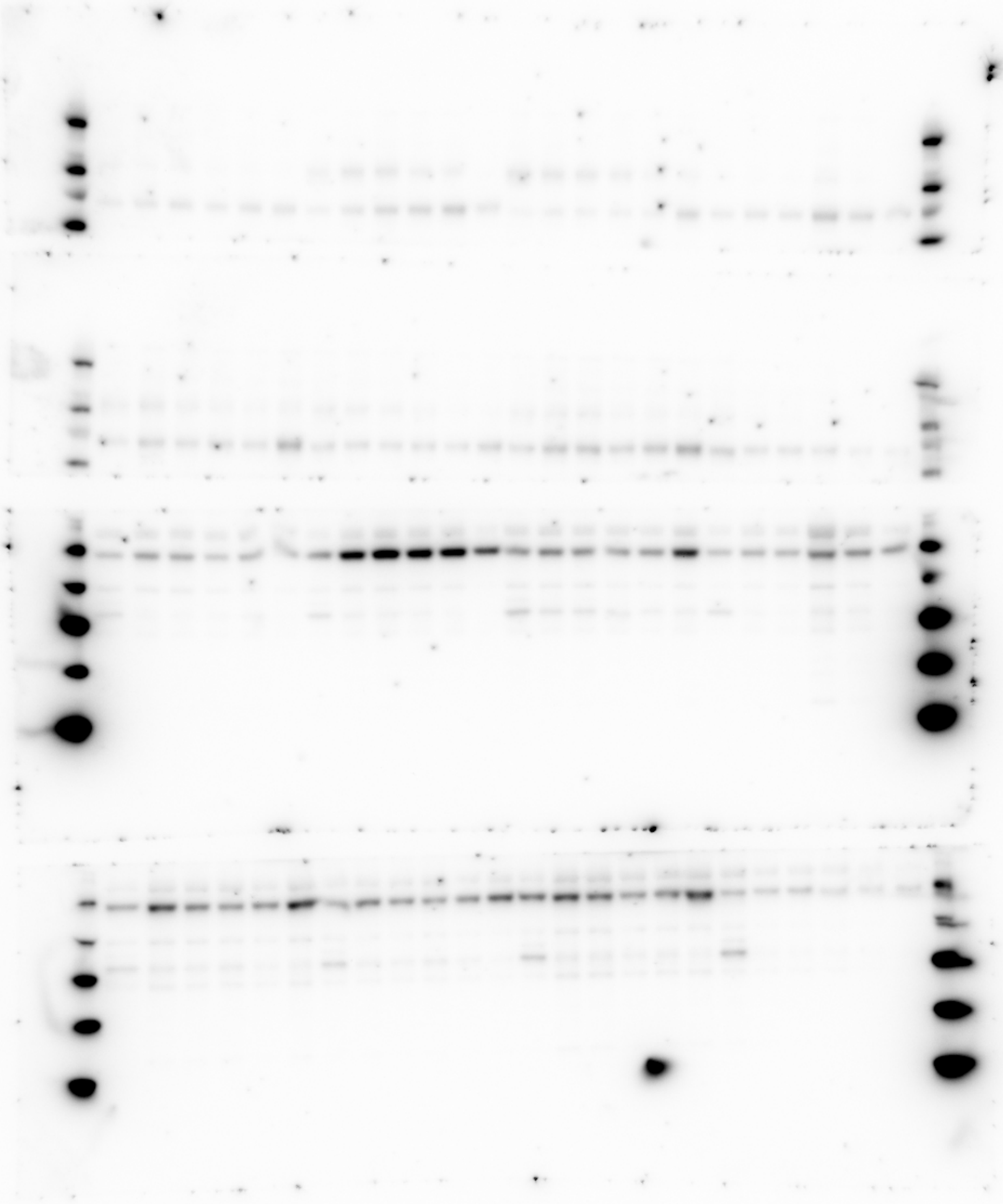

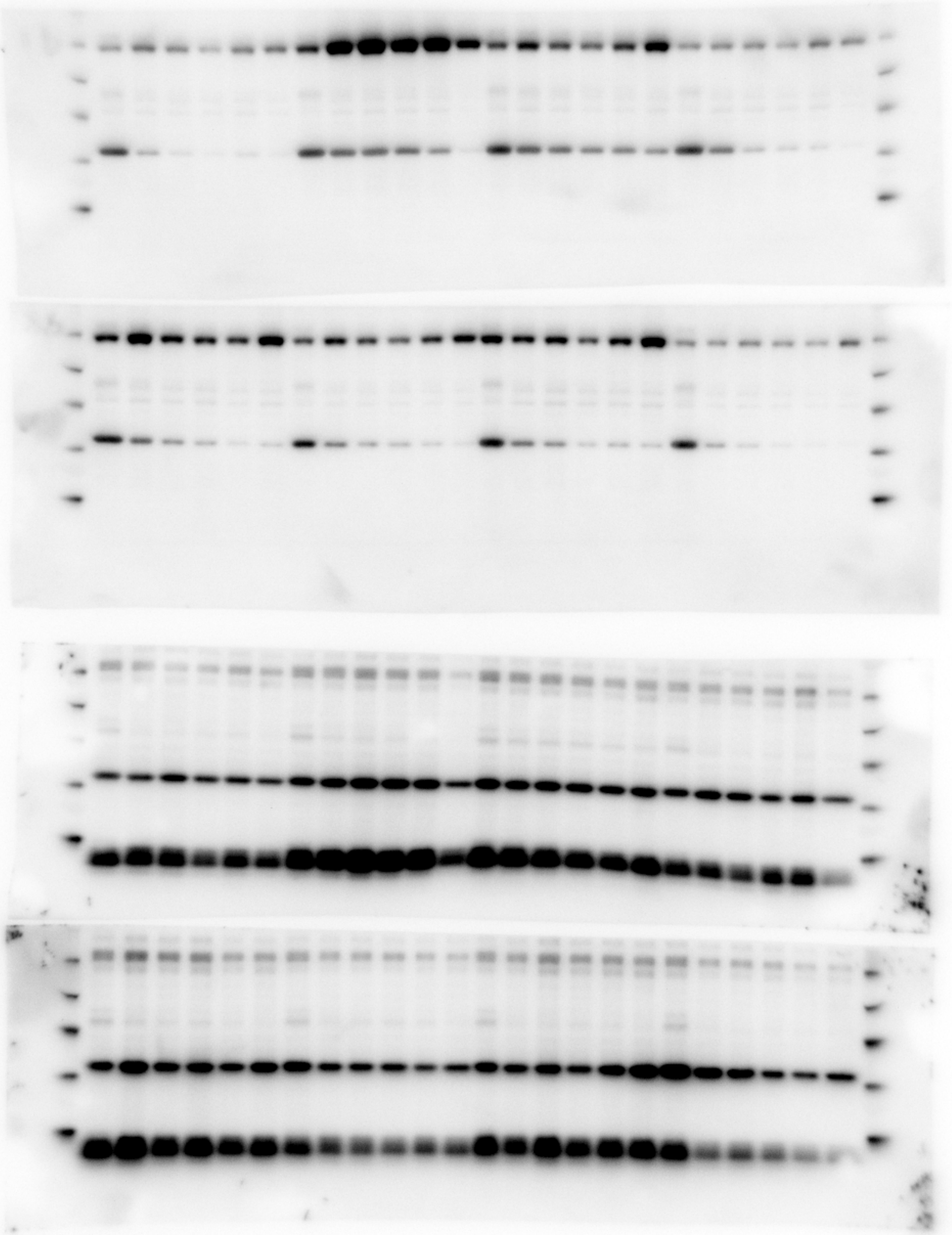

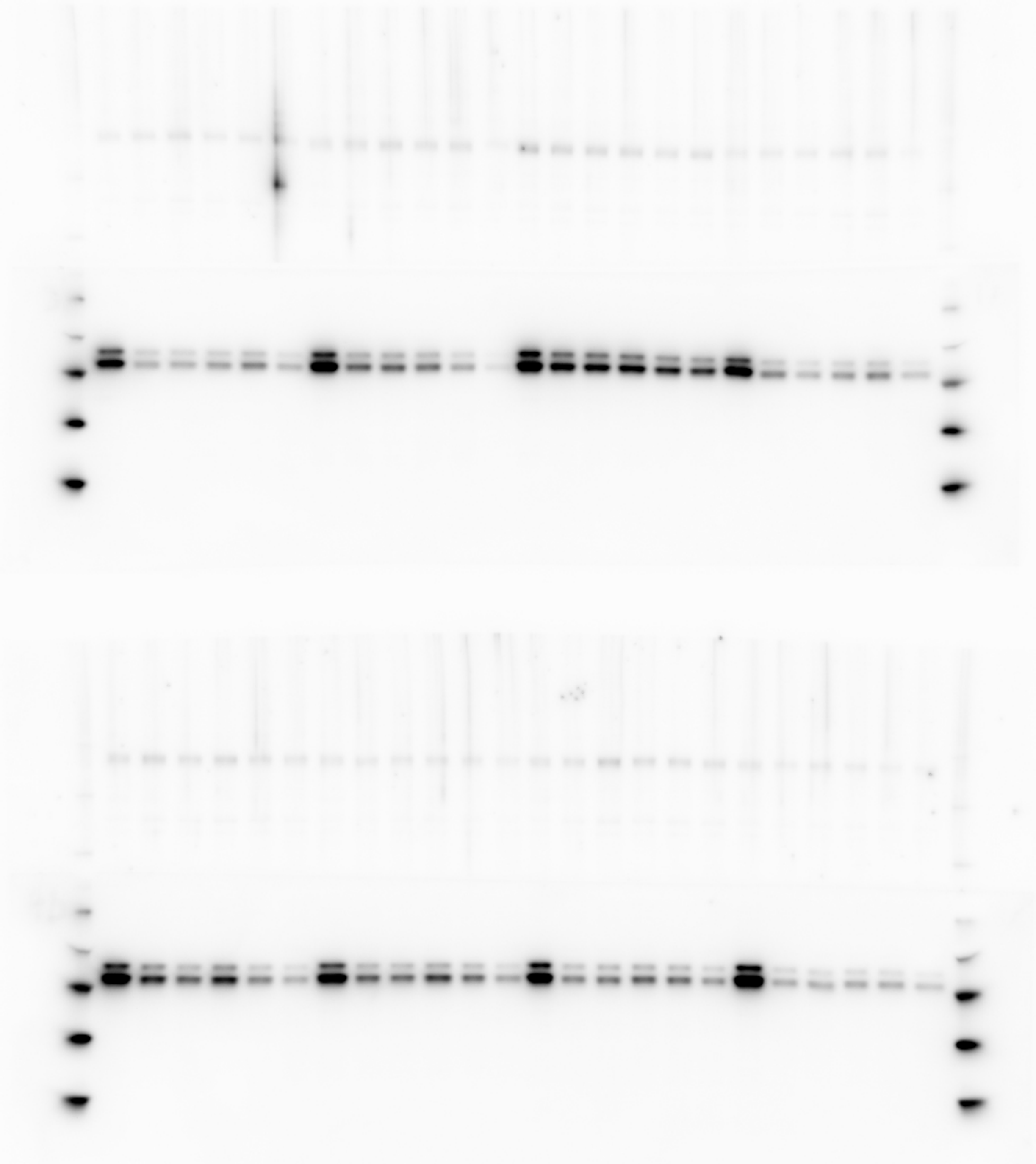

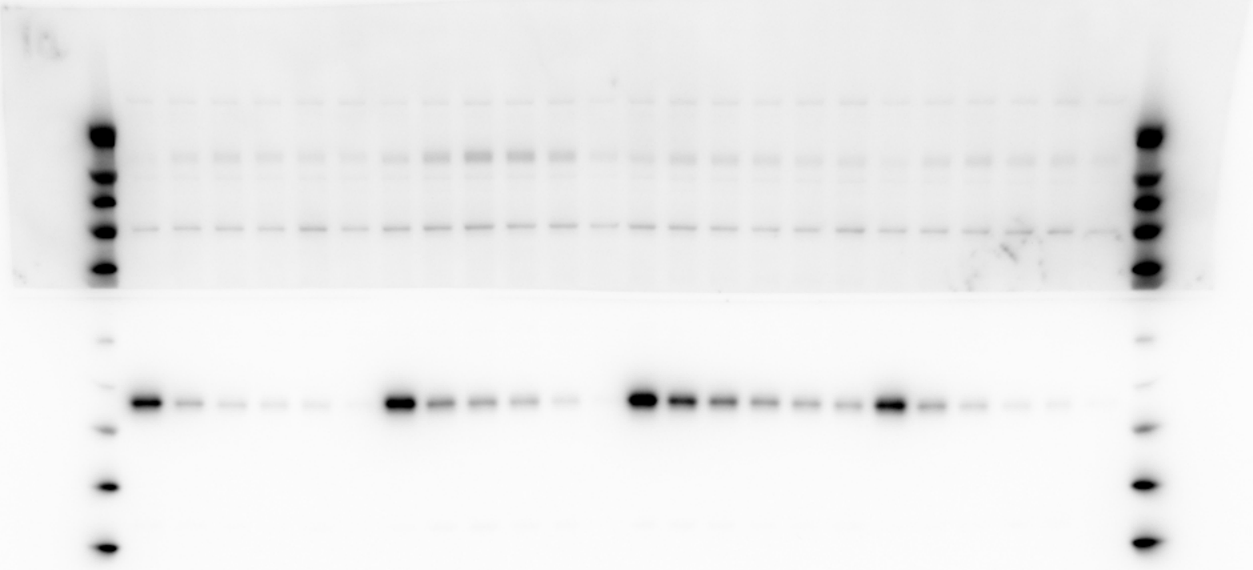

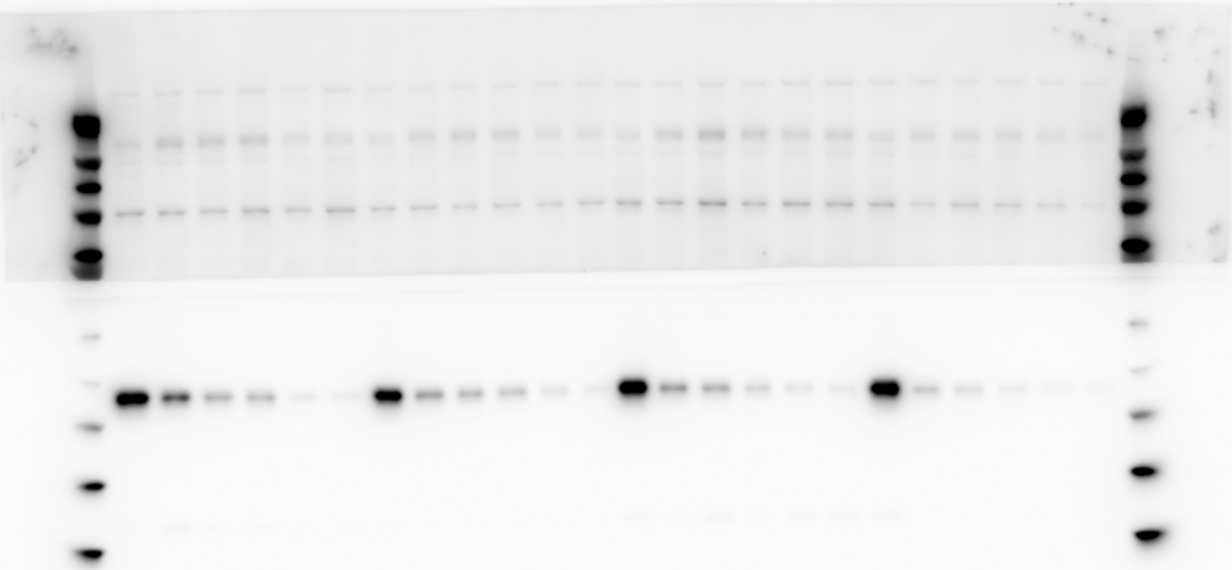

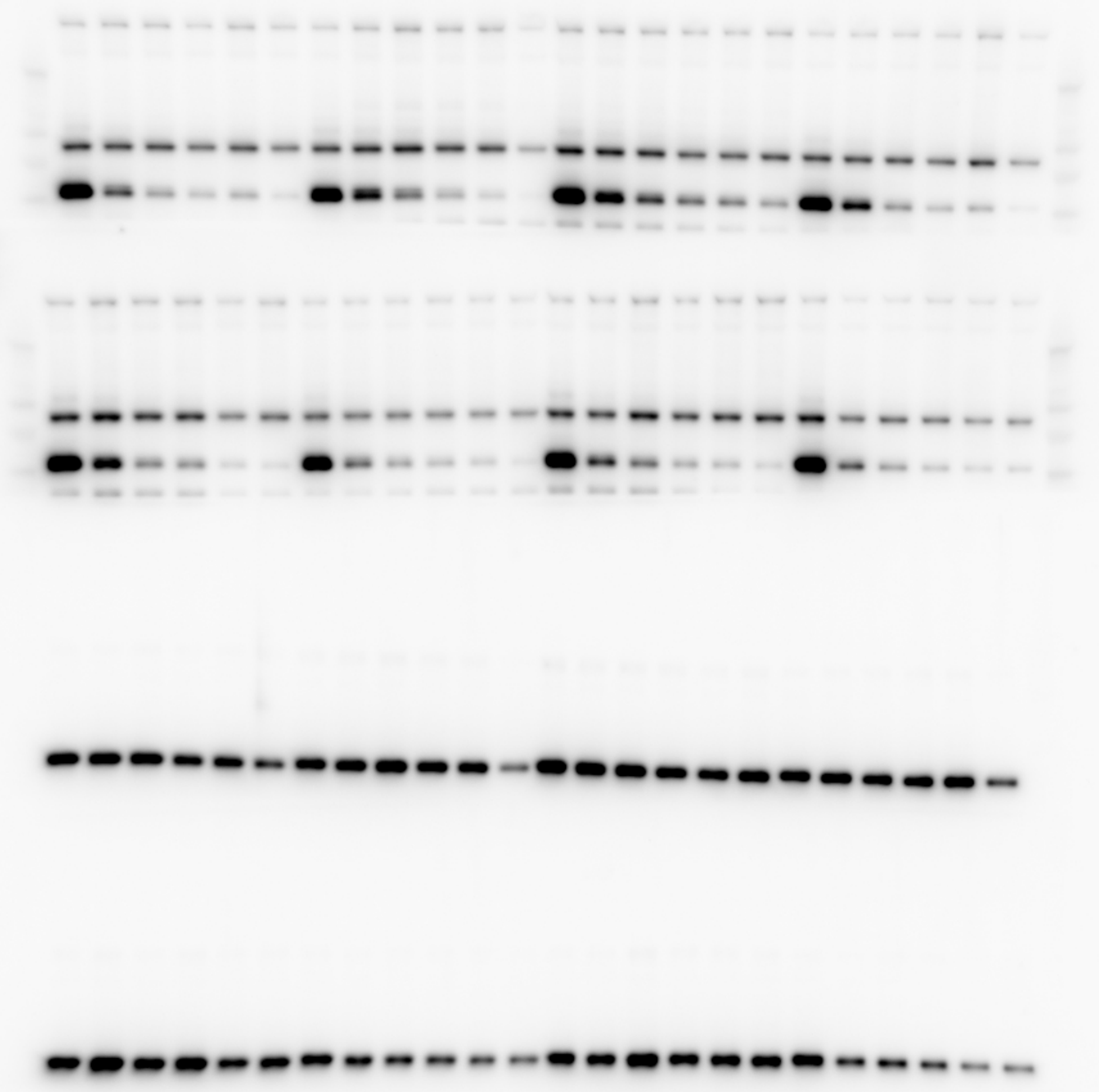

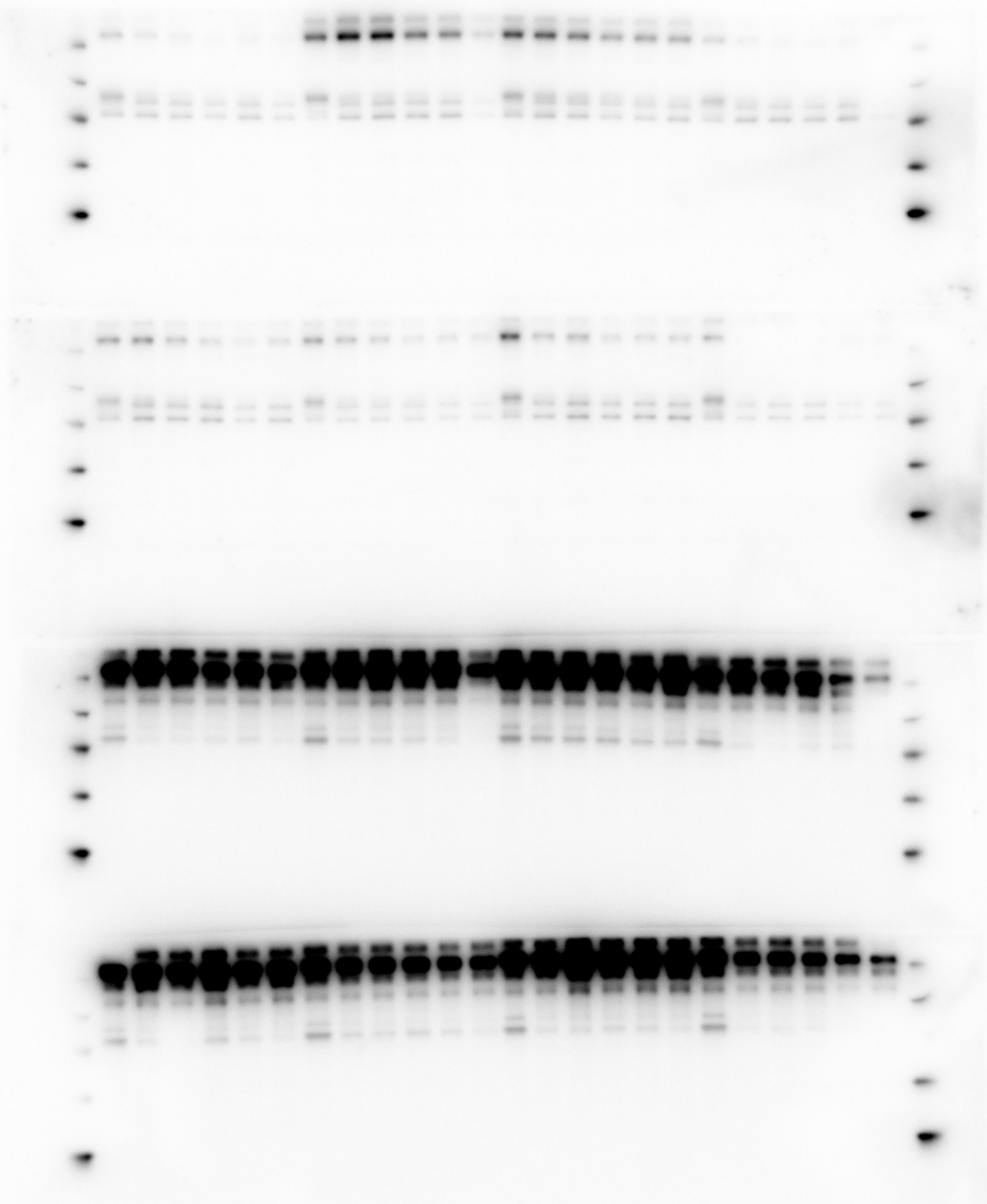

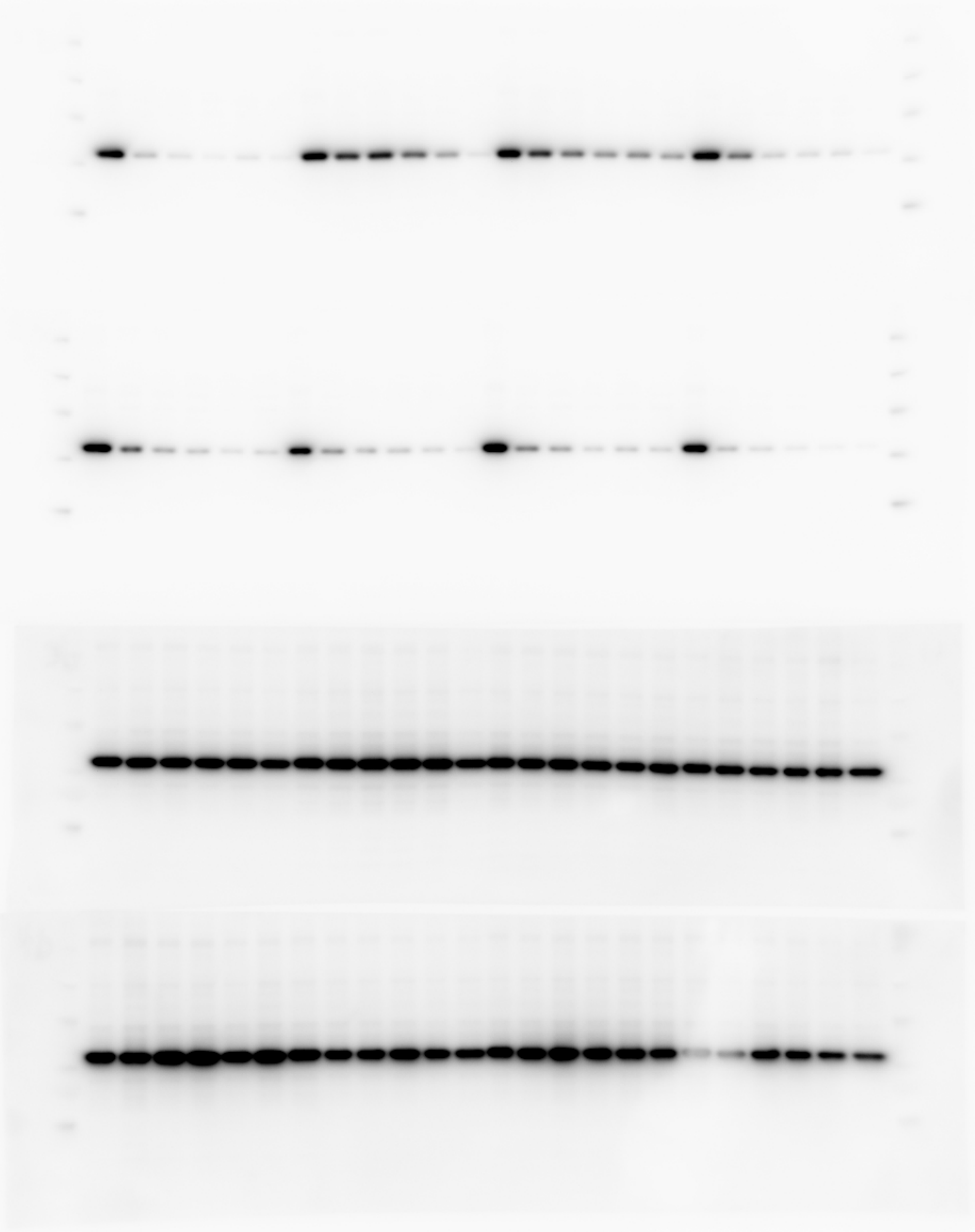

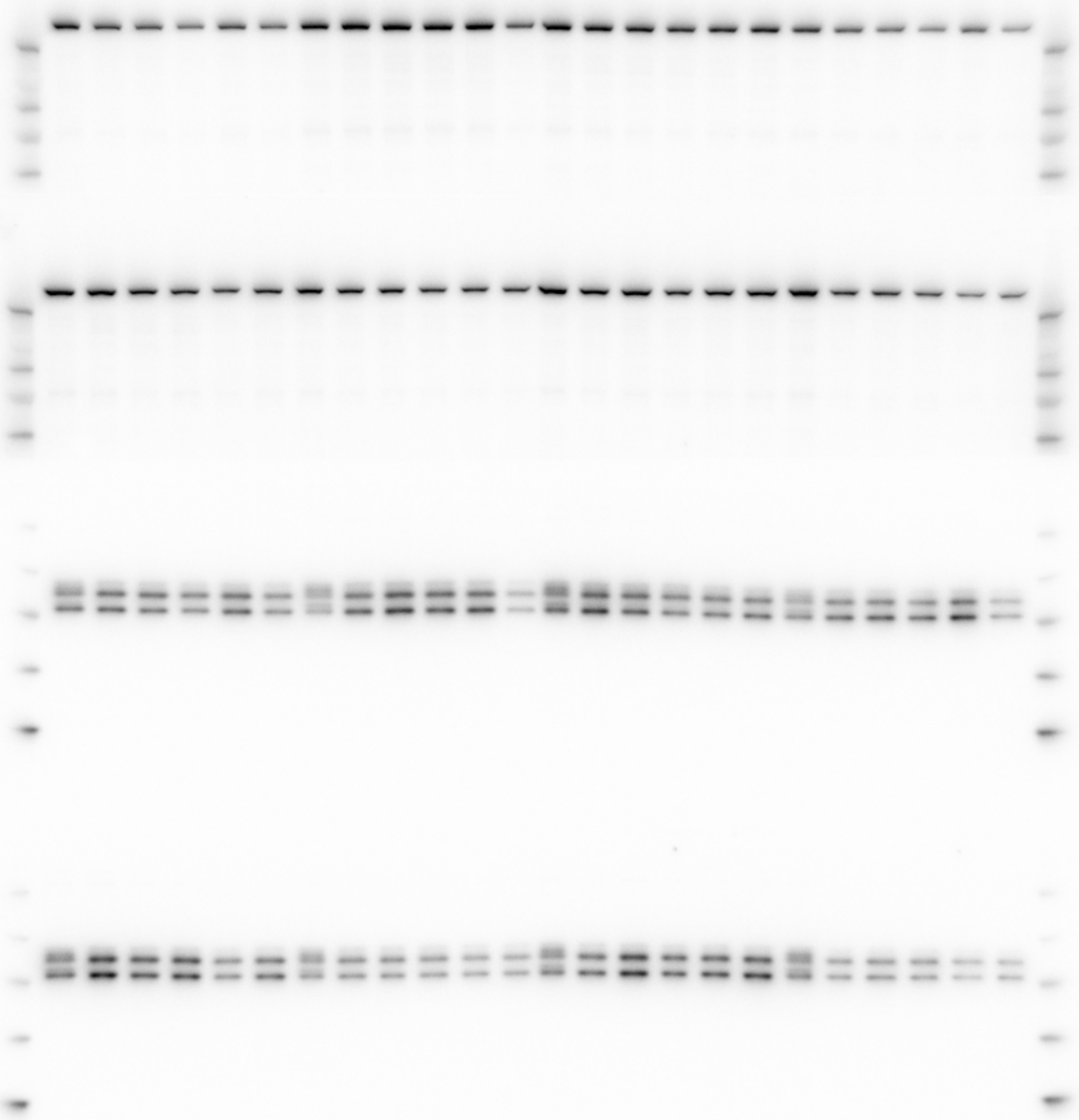

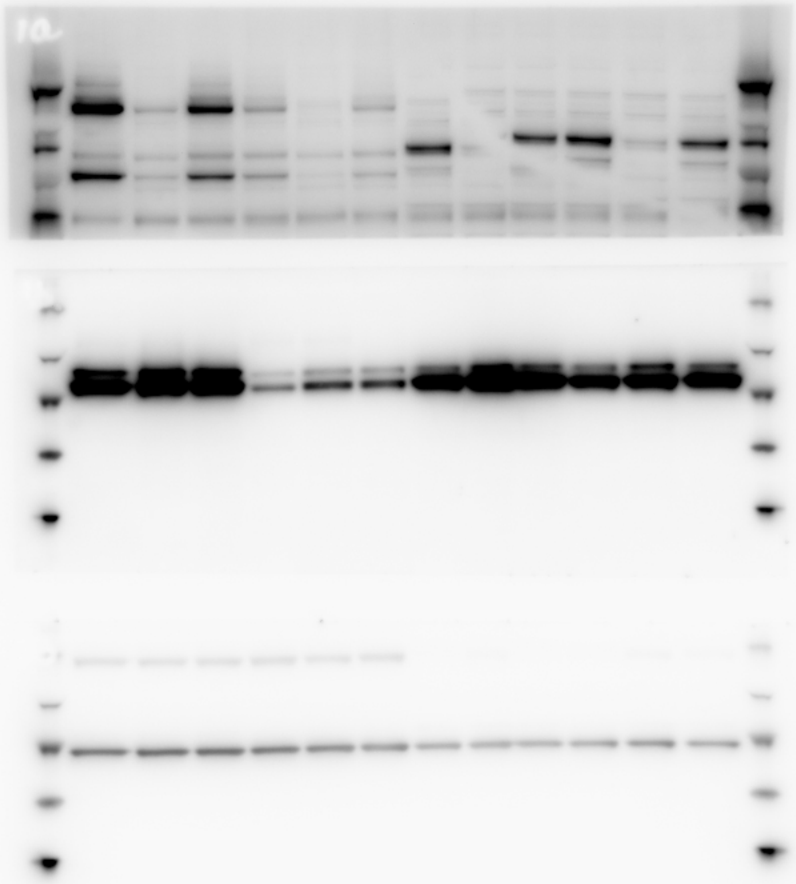
1a

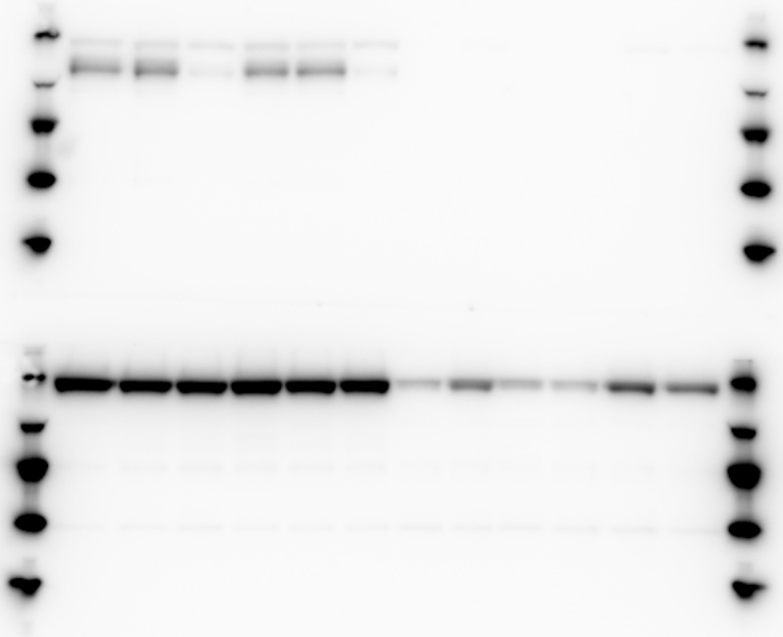

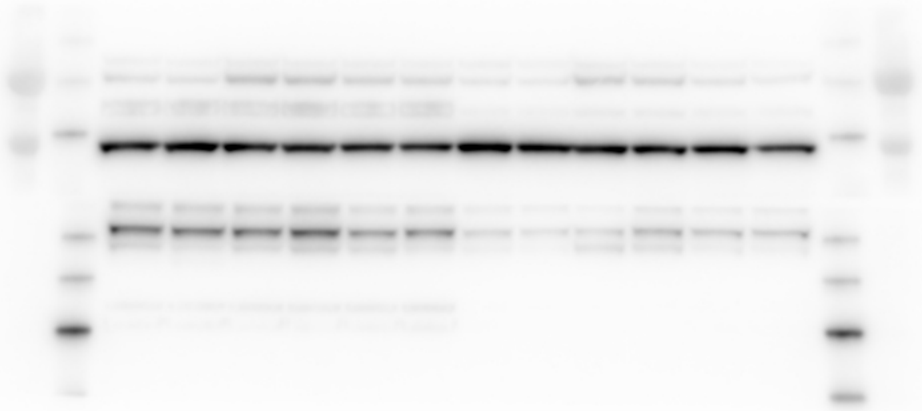

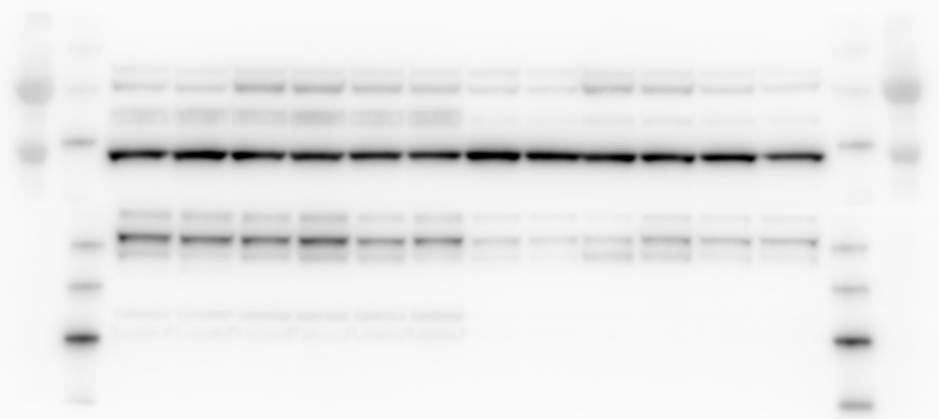

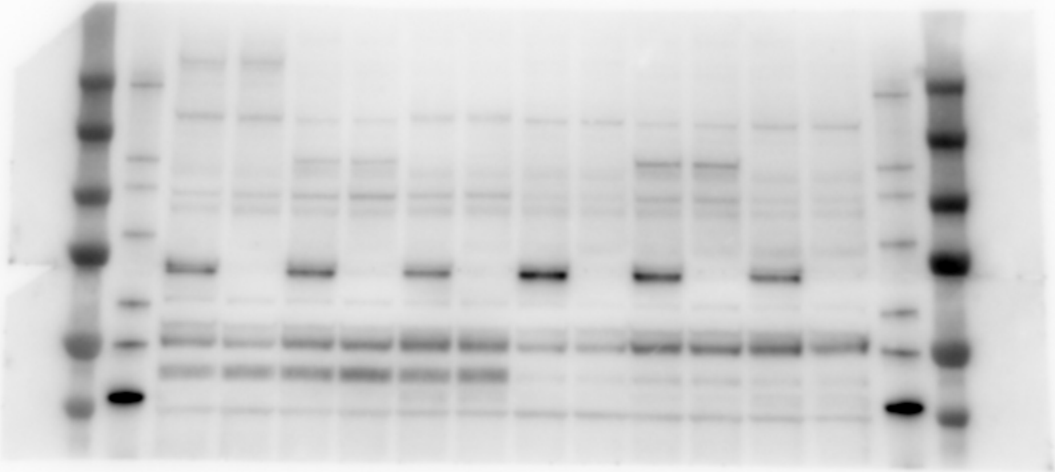

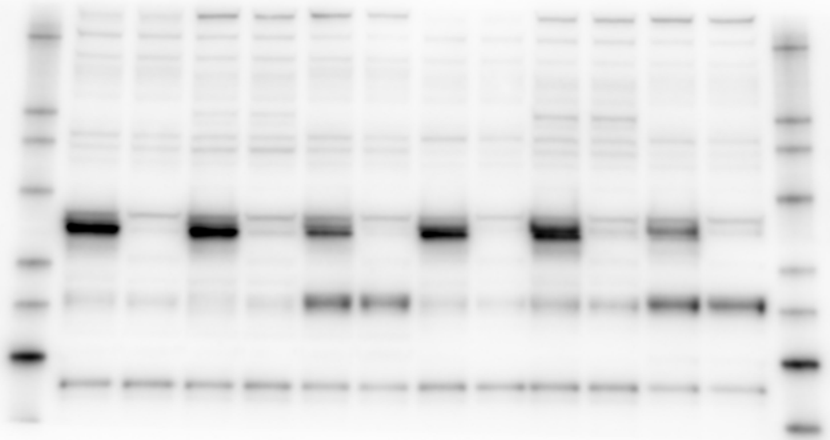

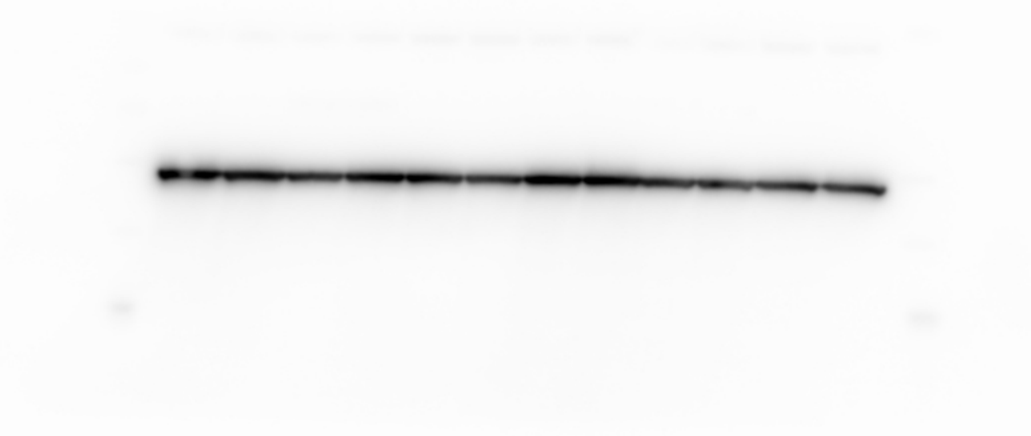

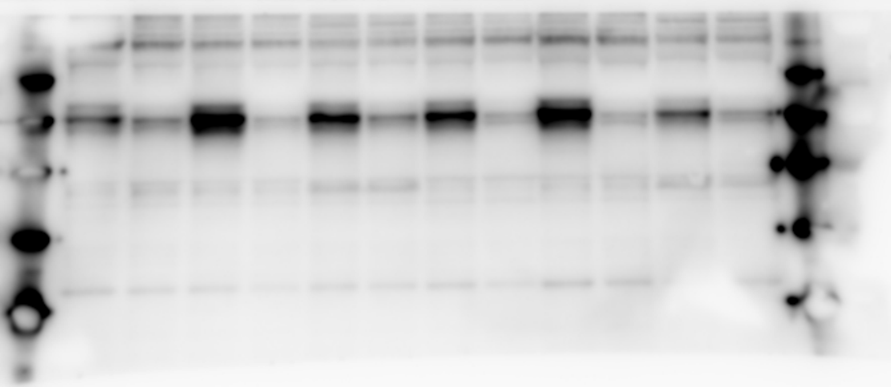

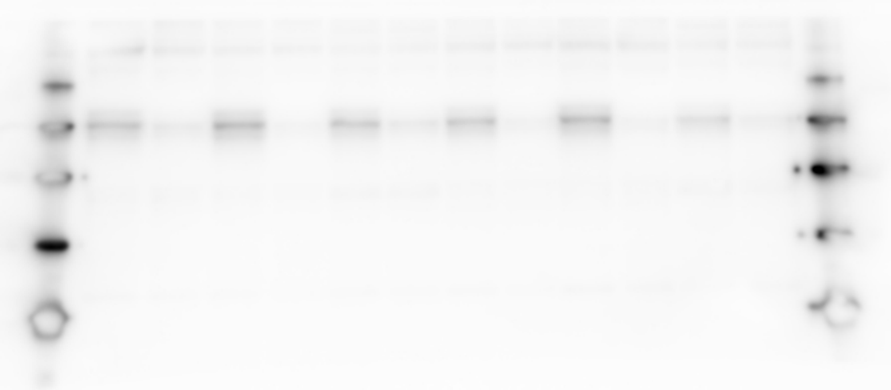

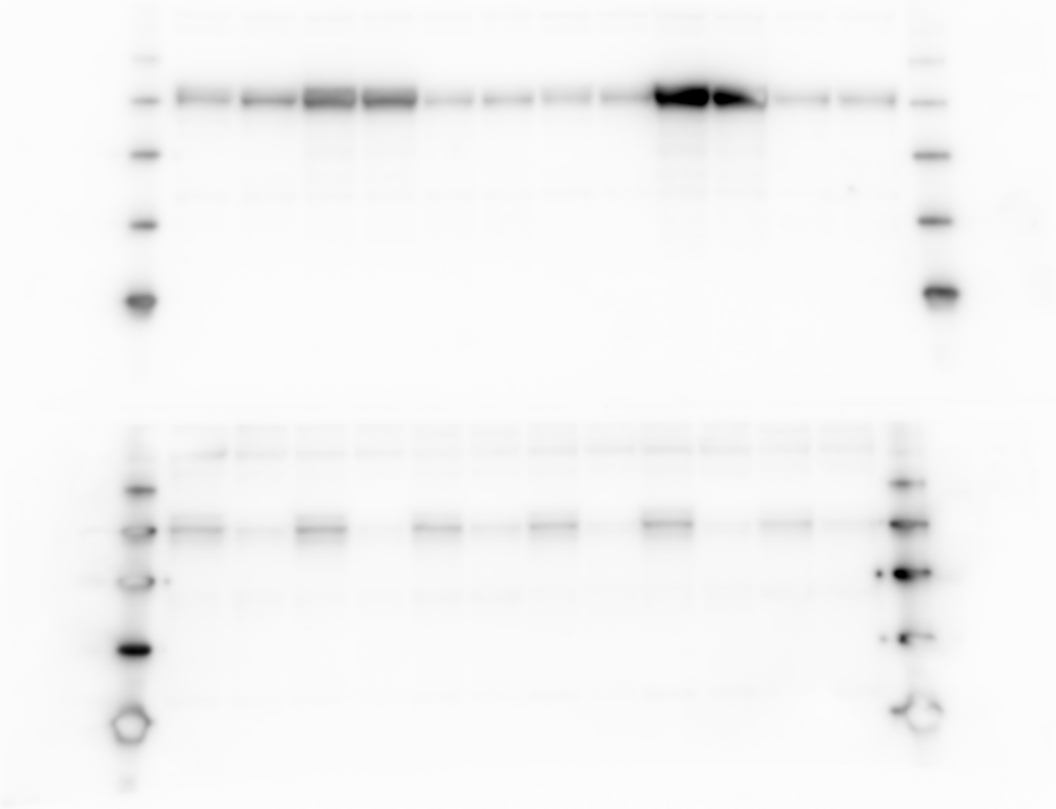

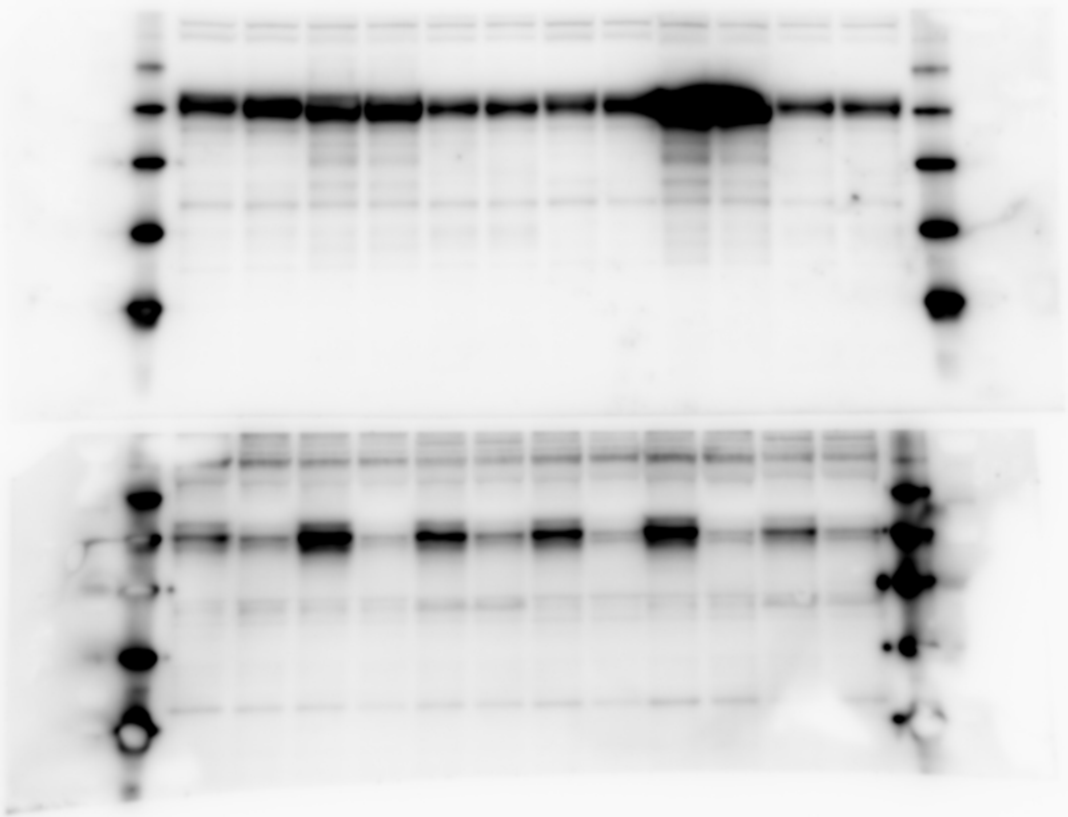

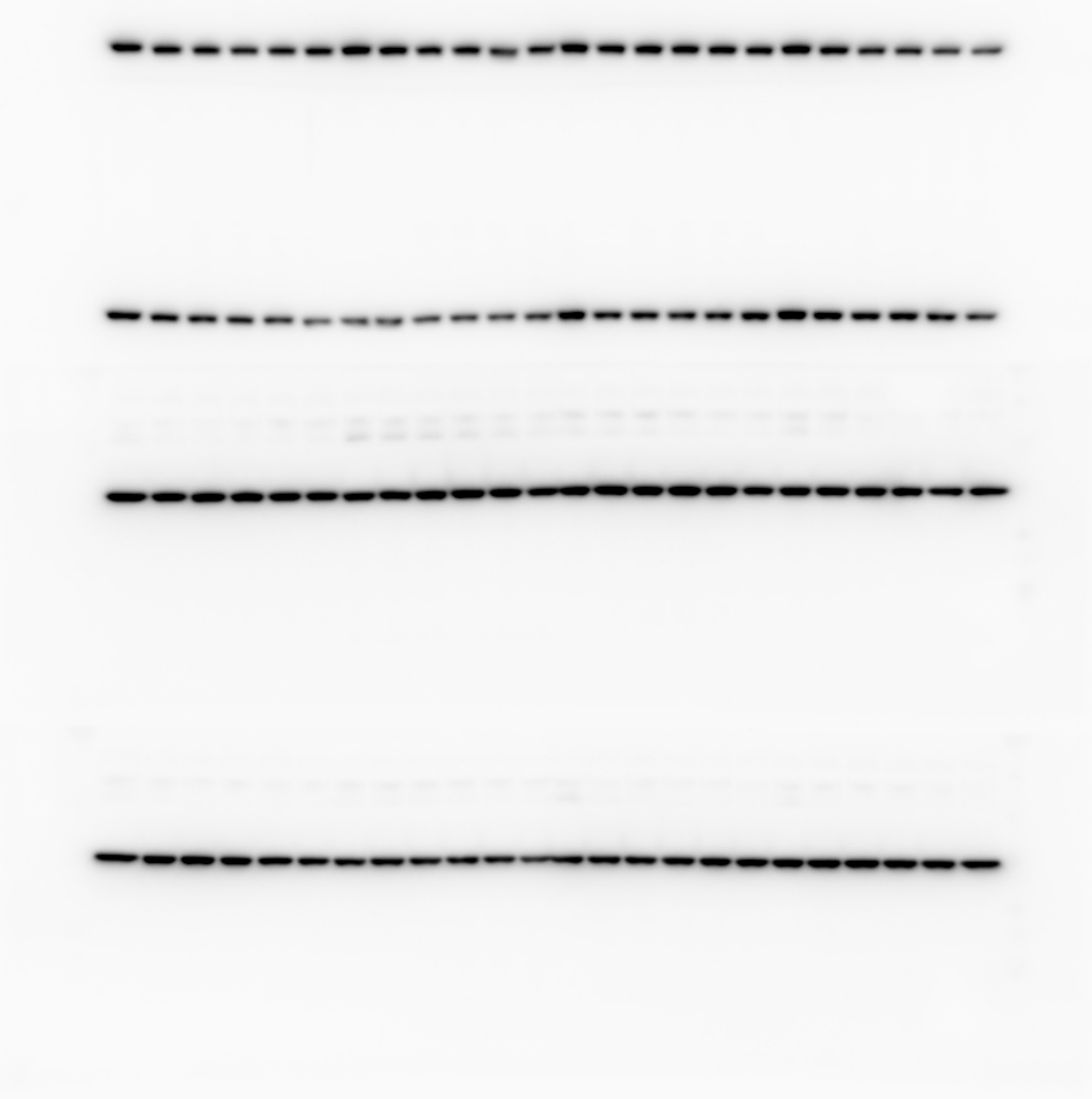

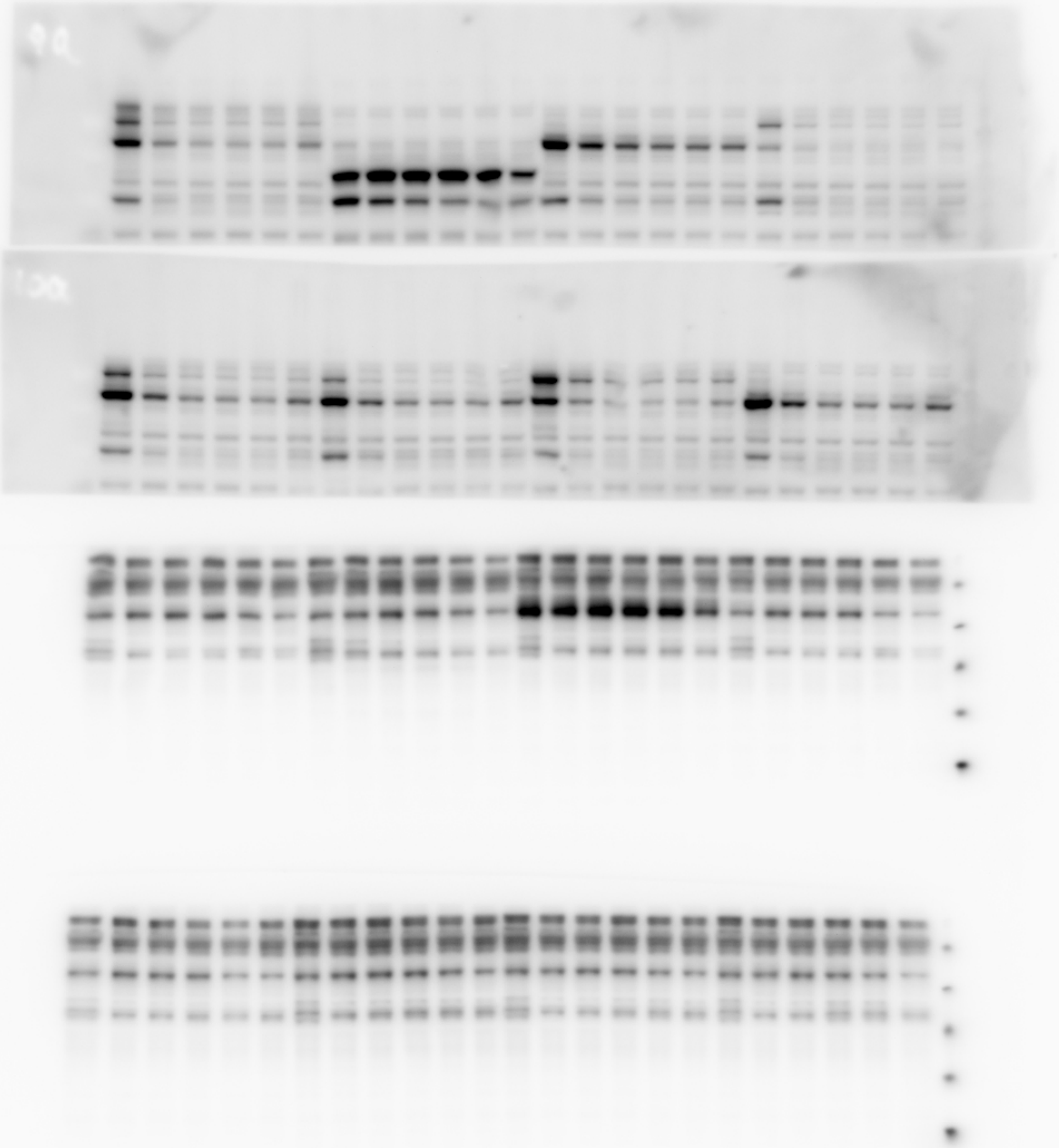

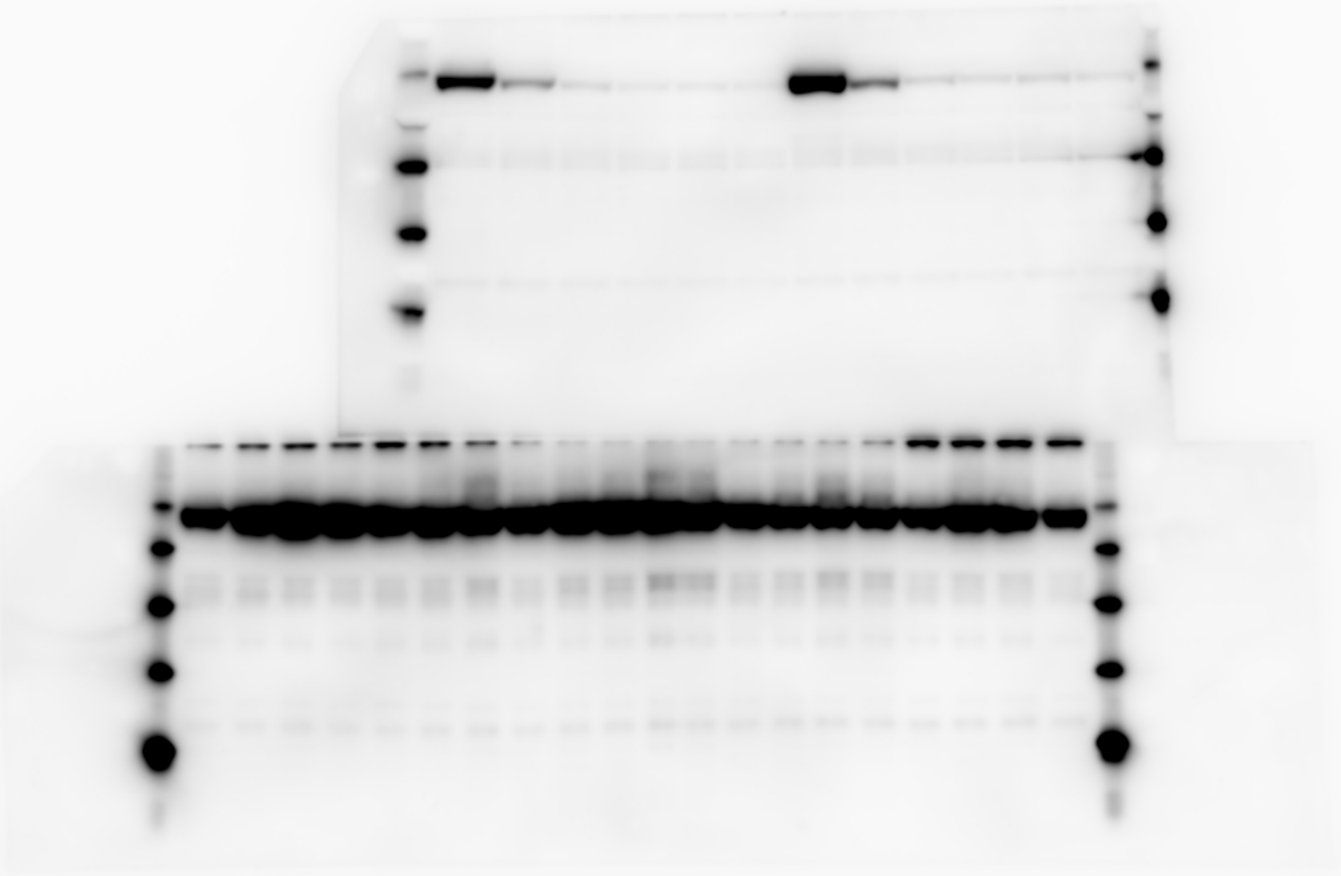

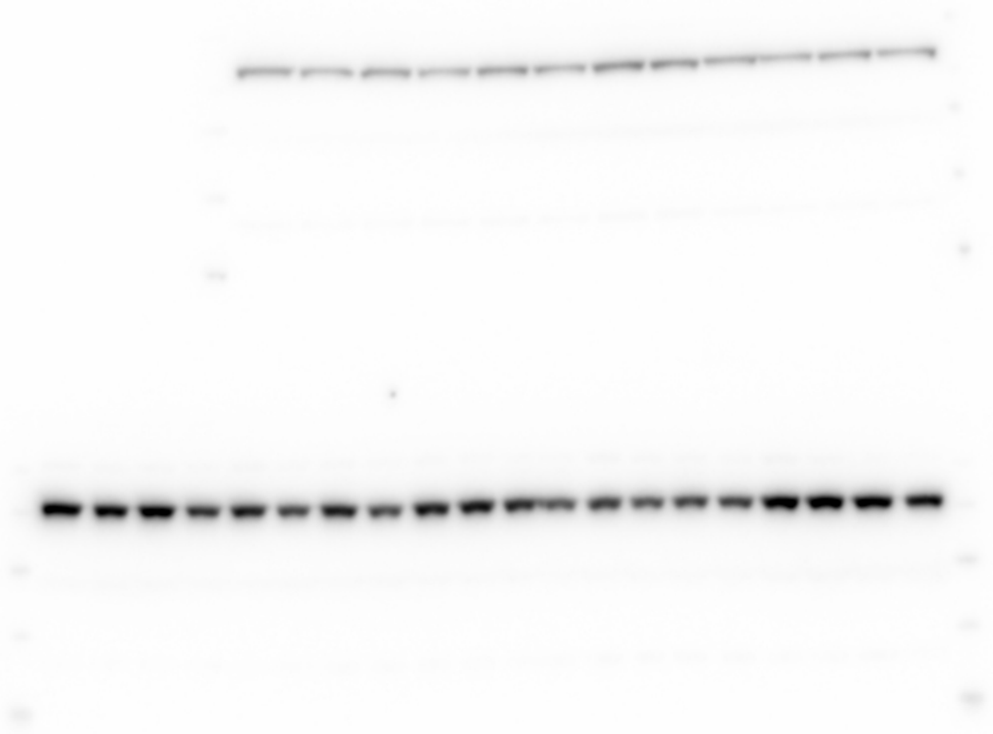

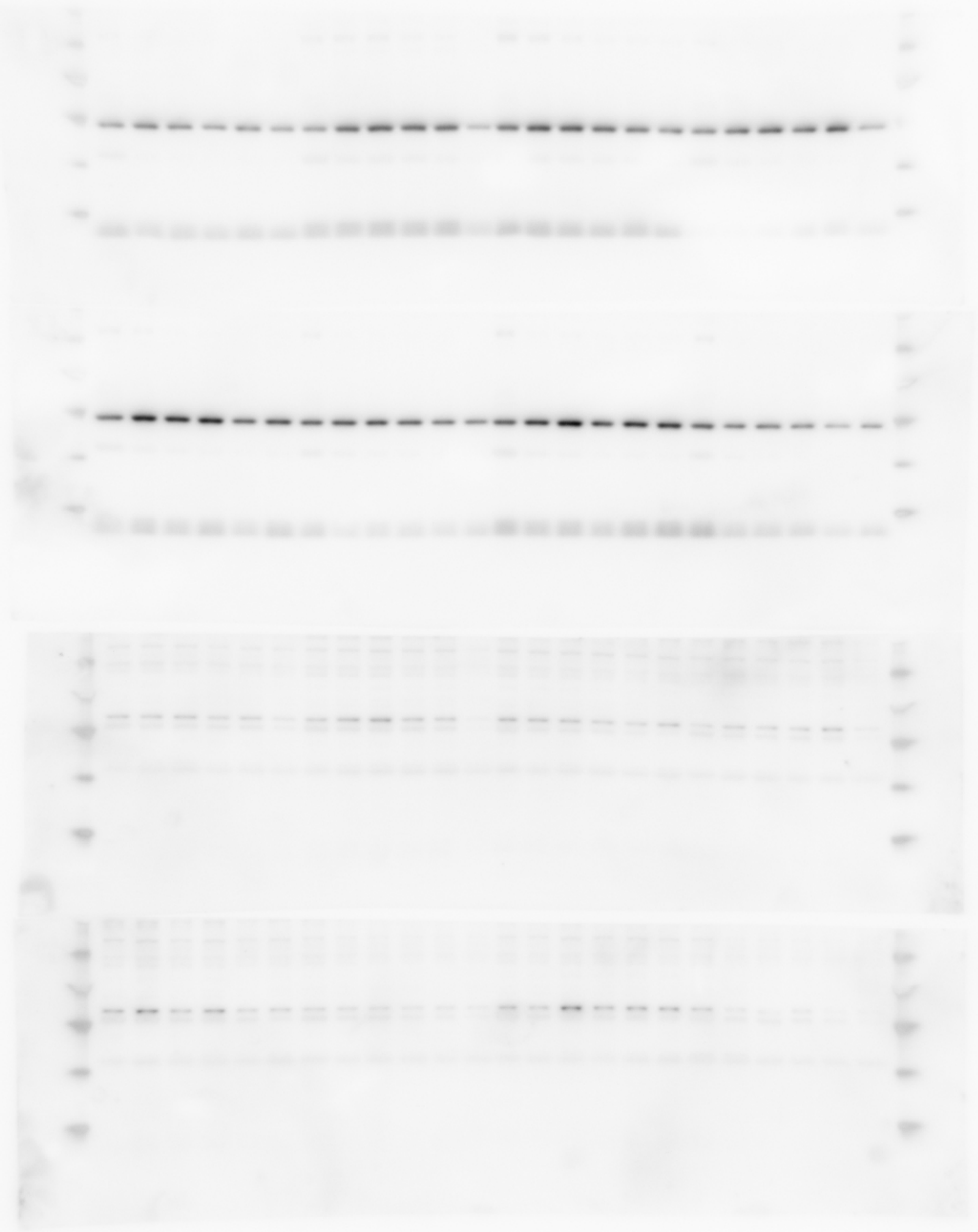

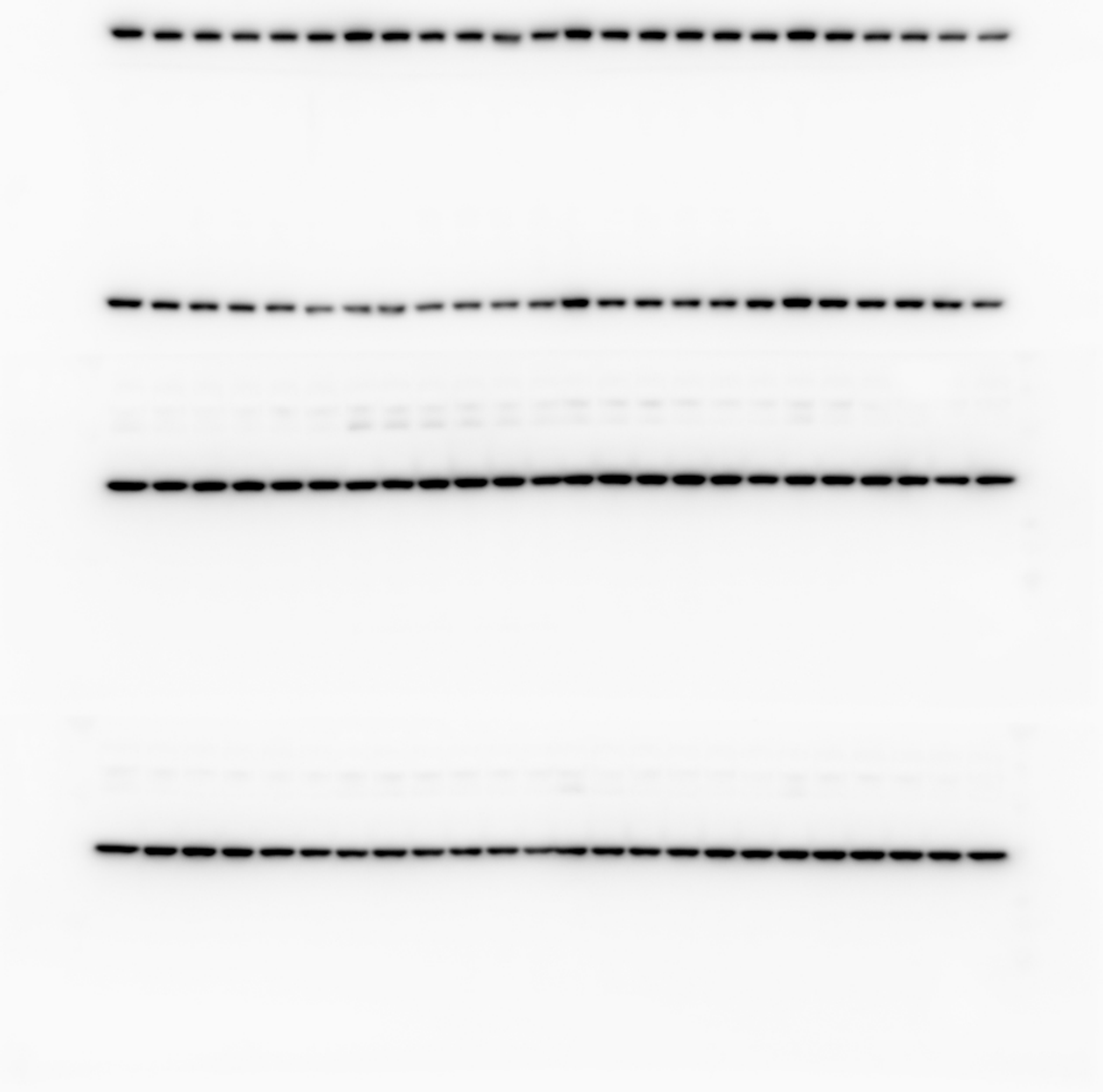

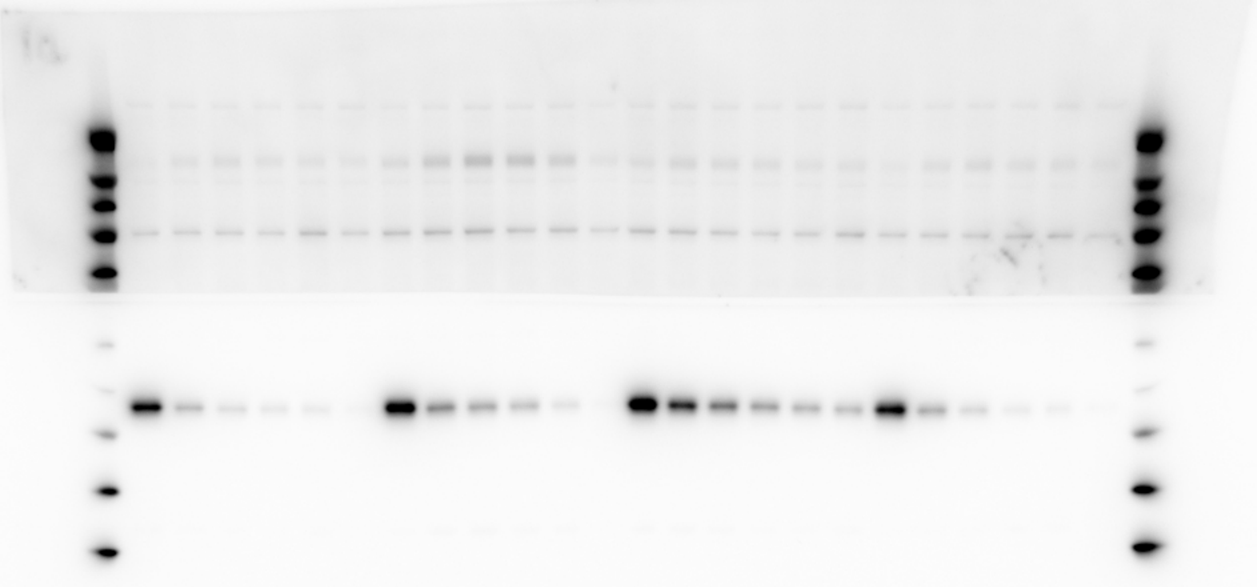
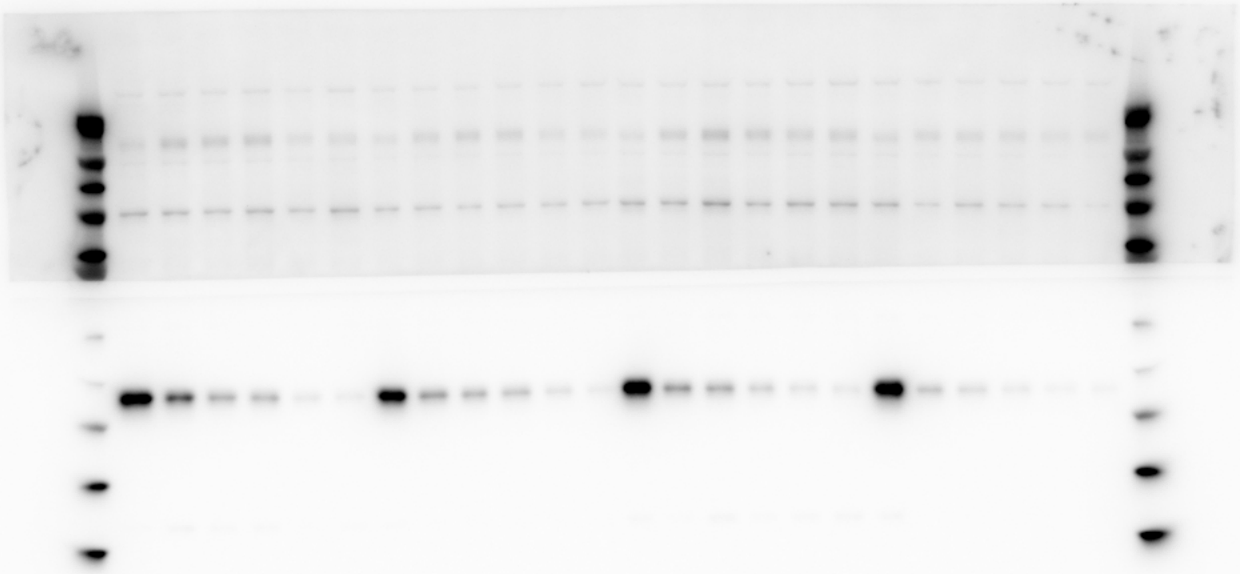

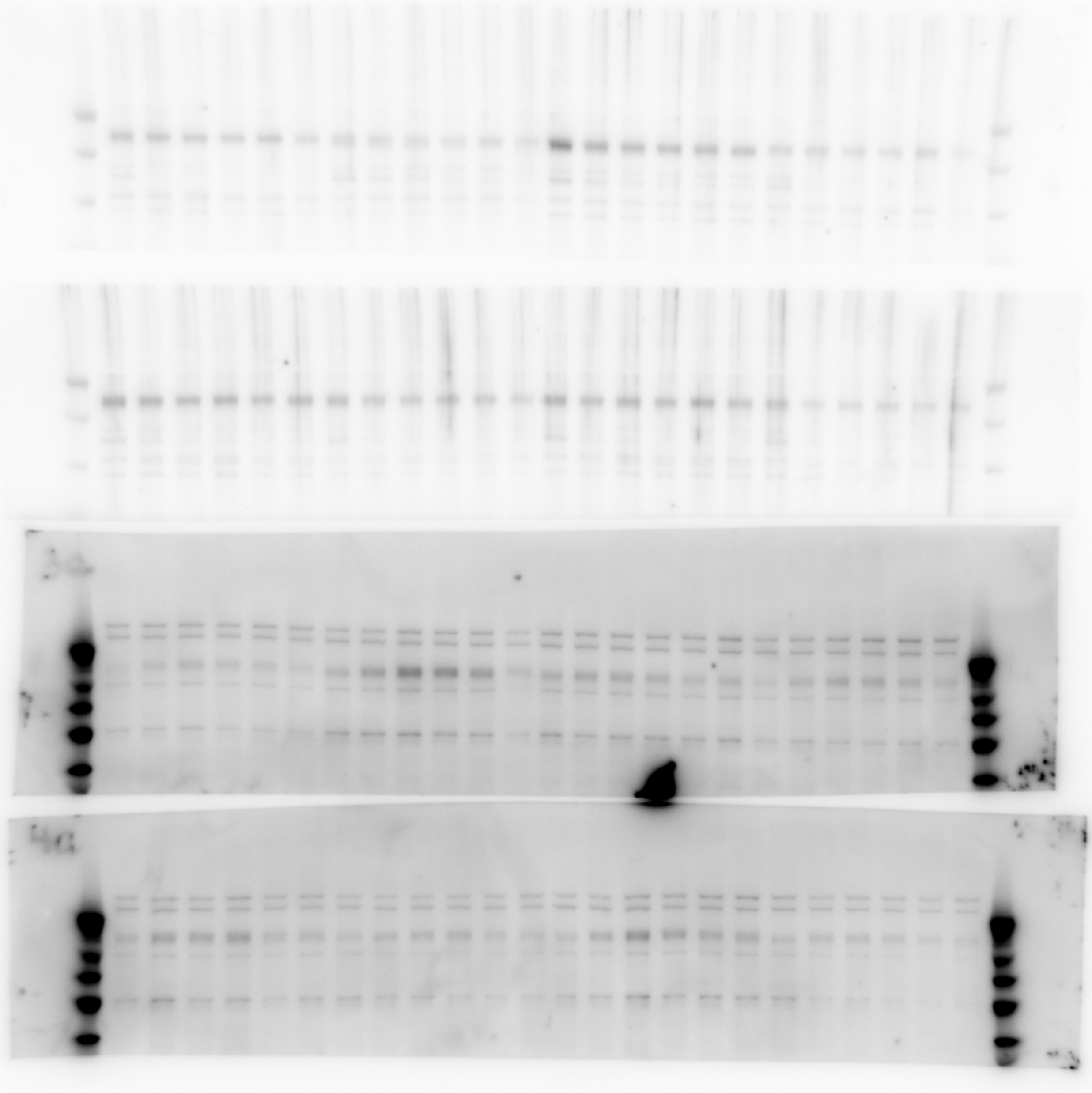

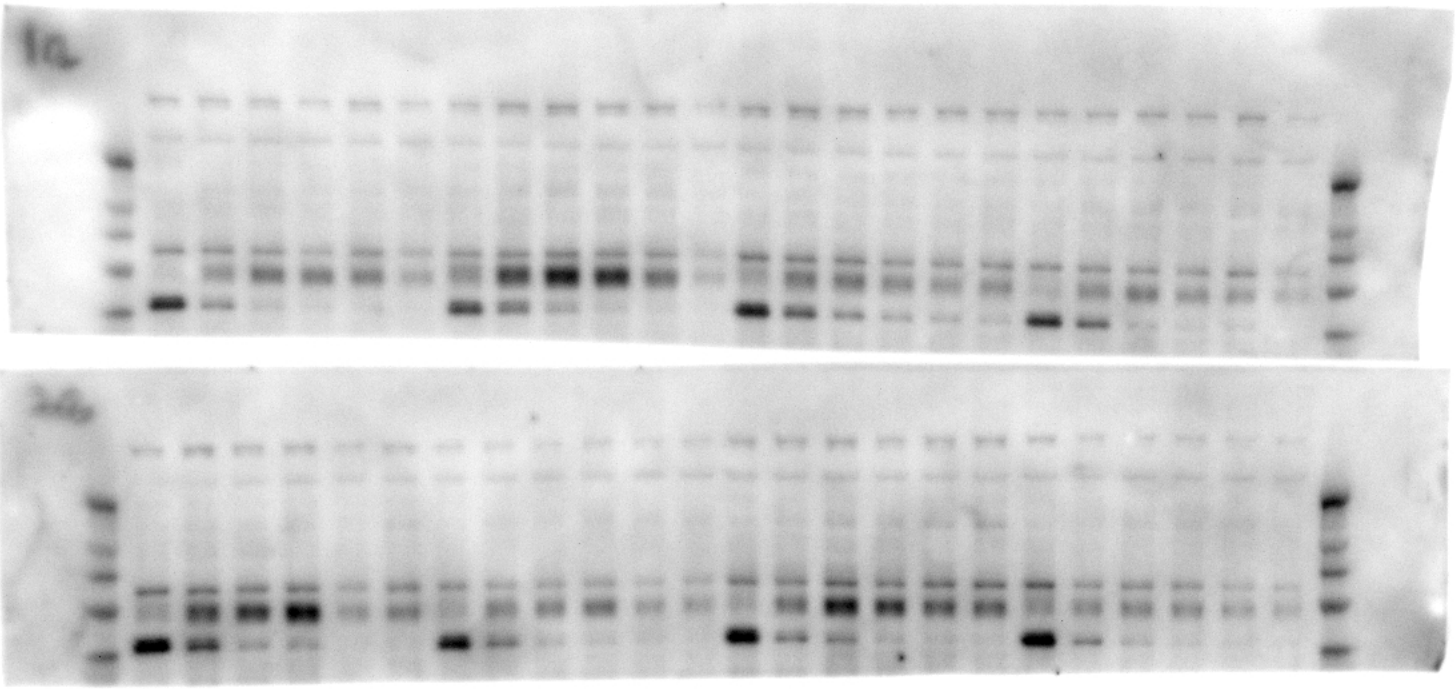

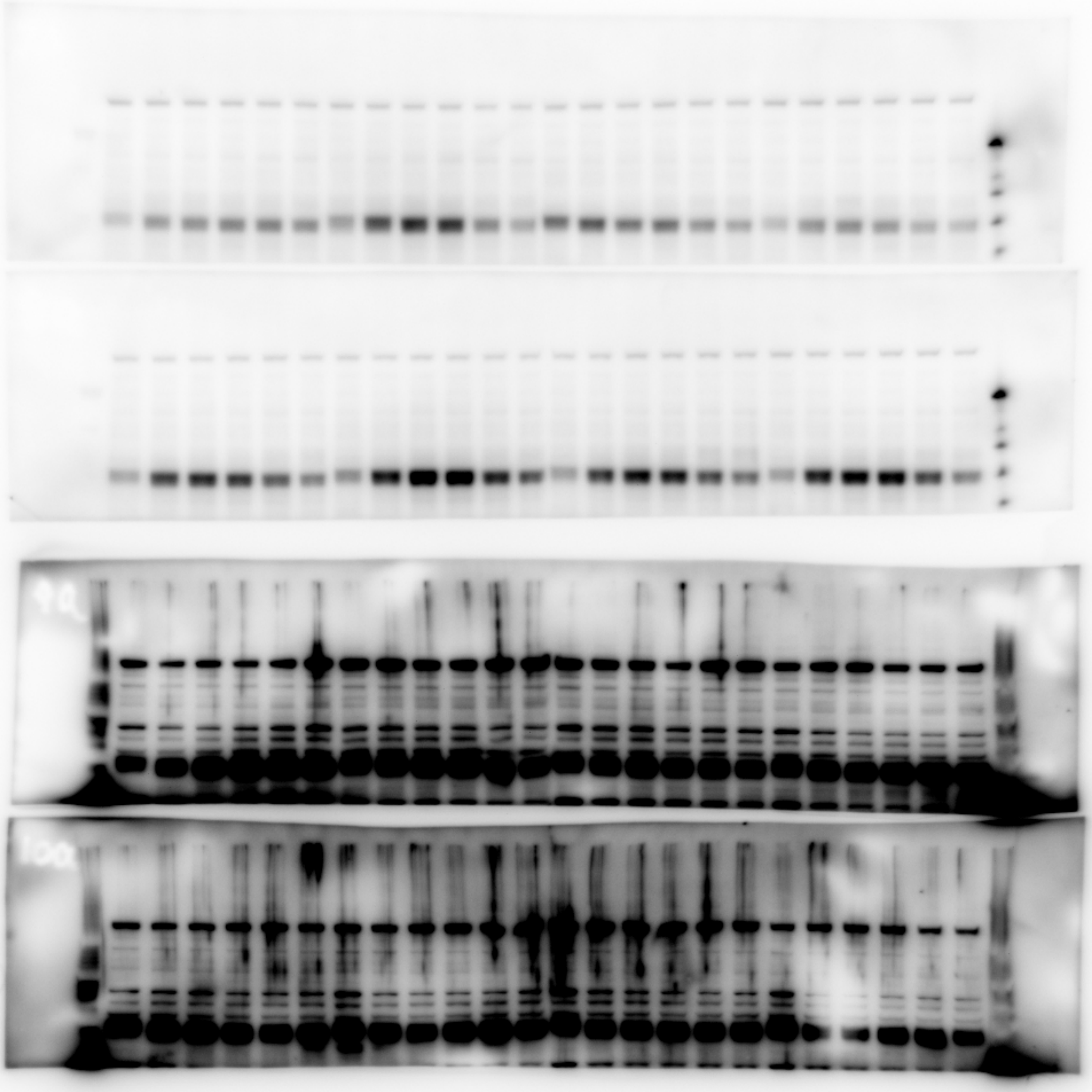
90
100

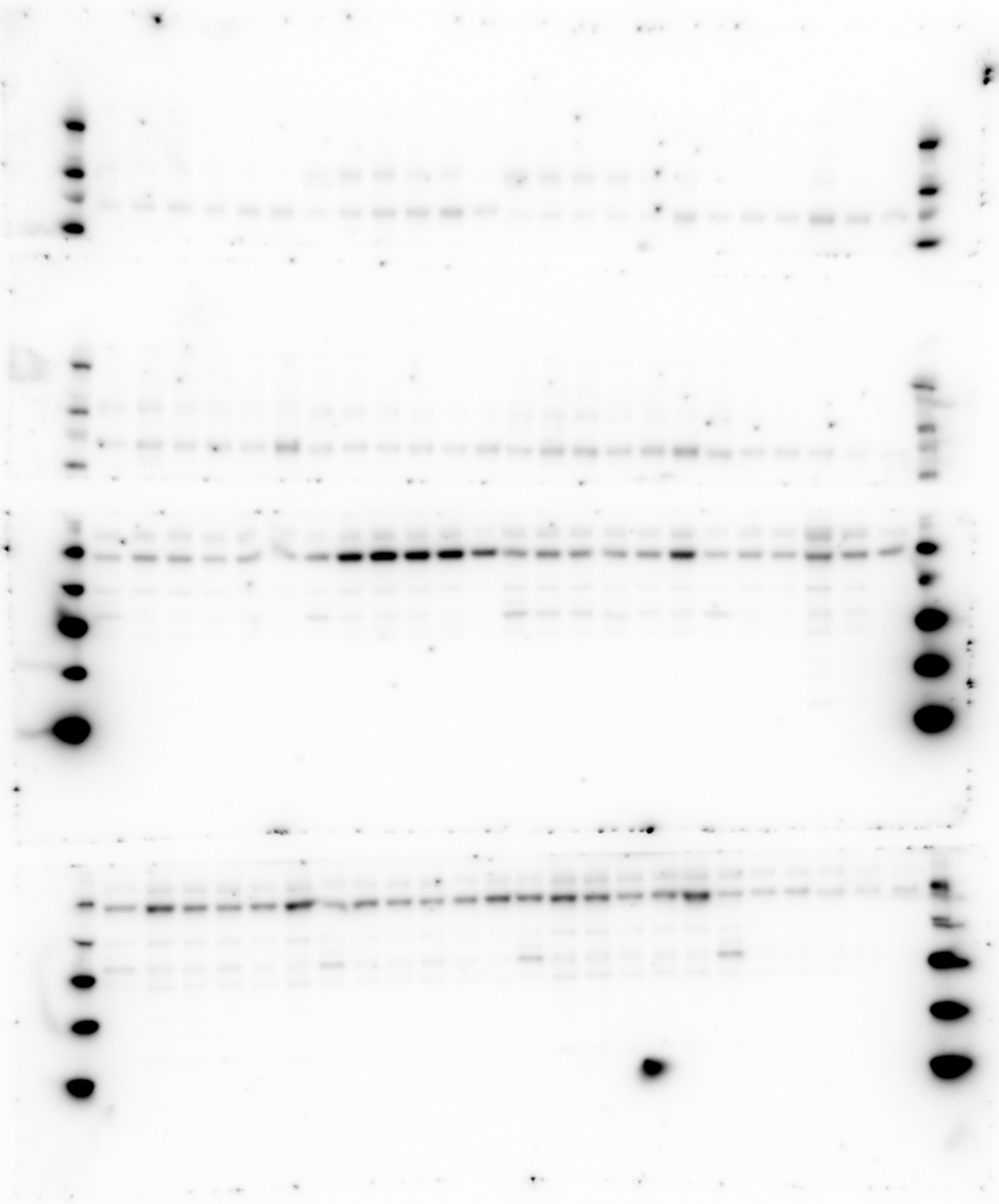

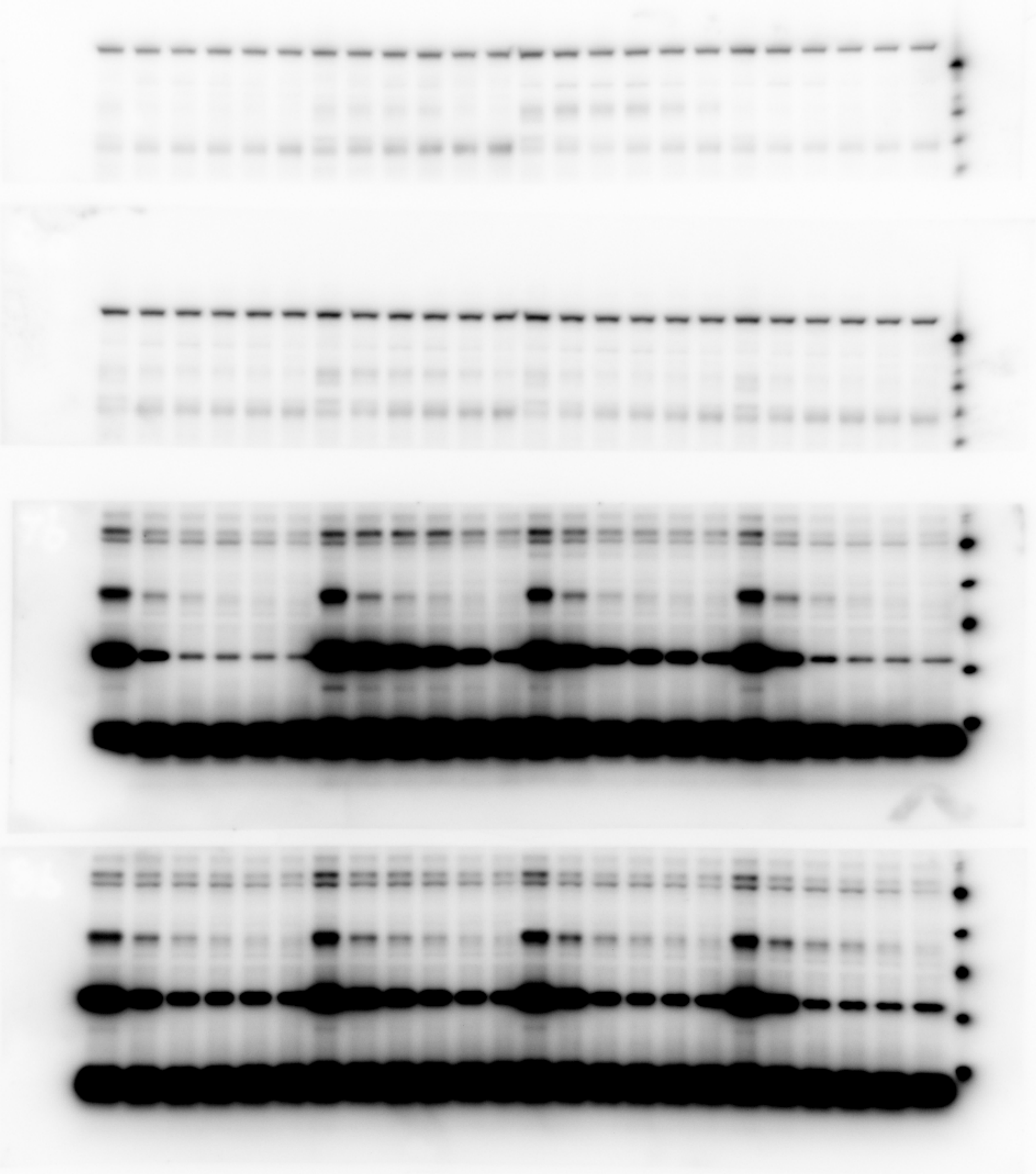

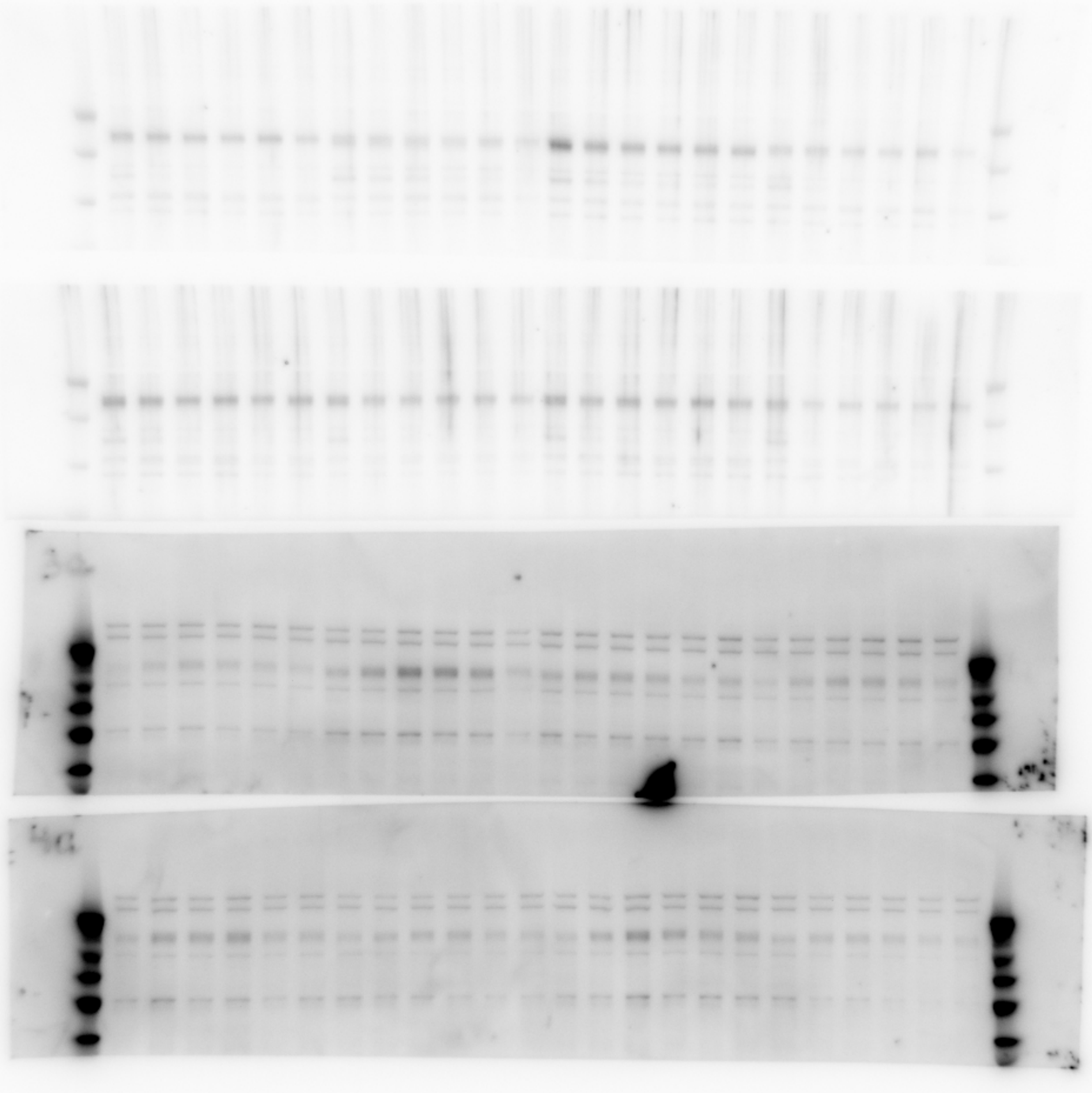

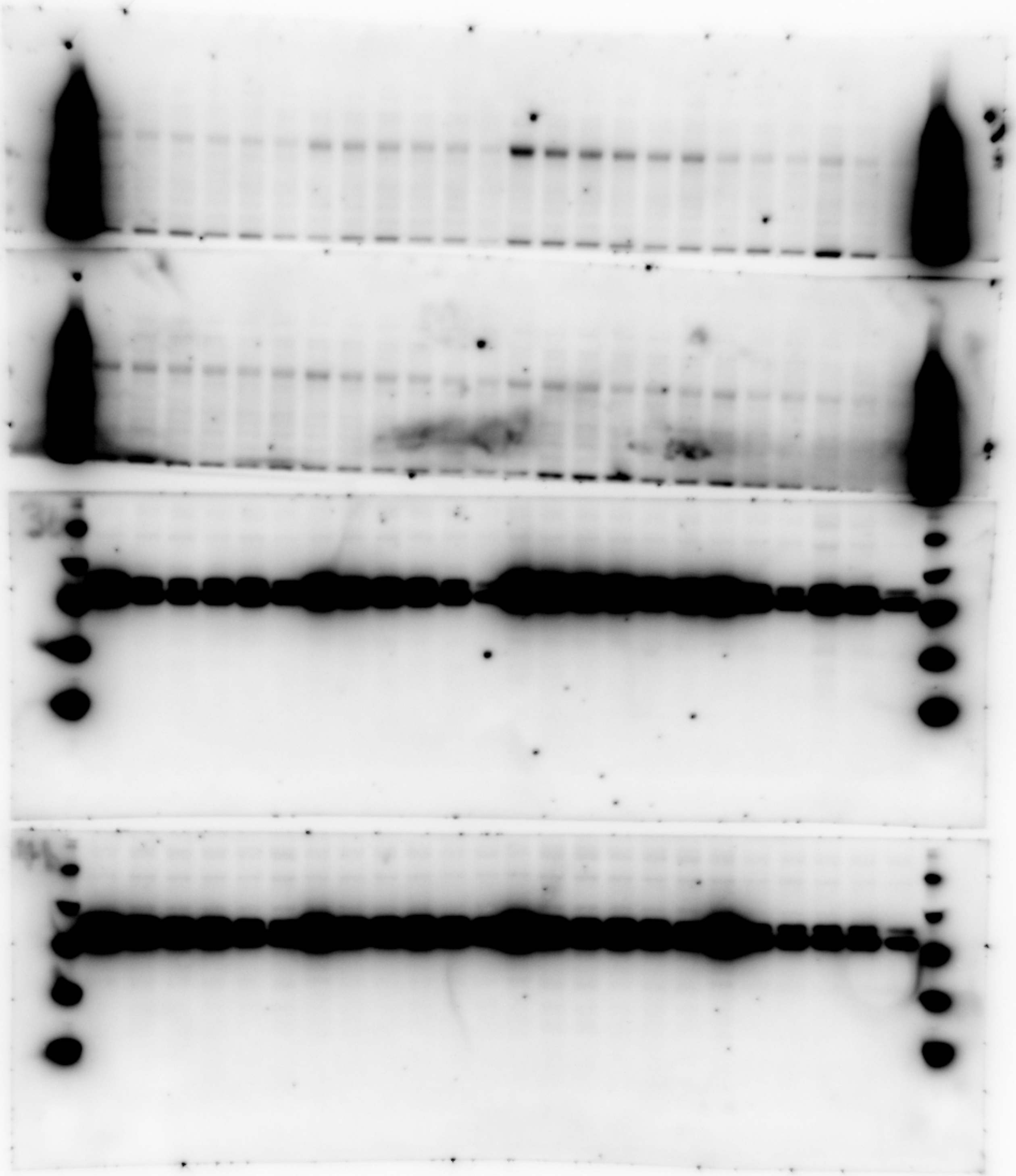

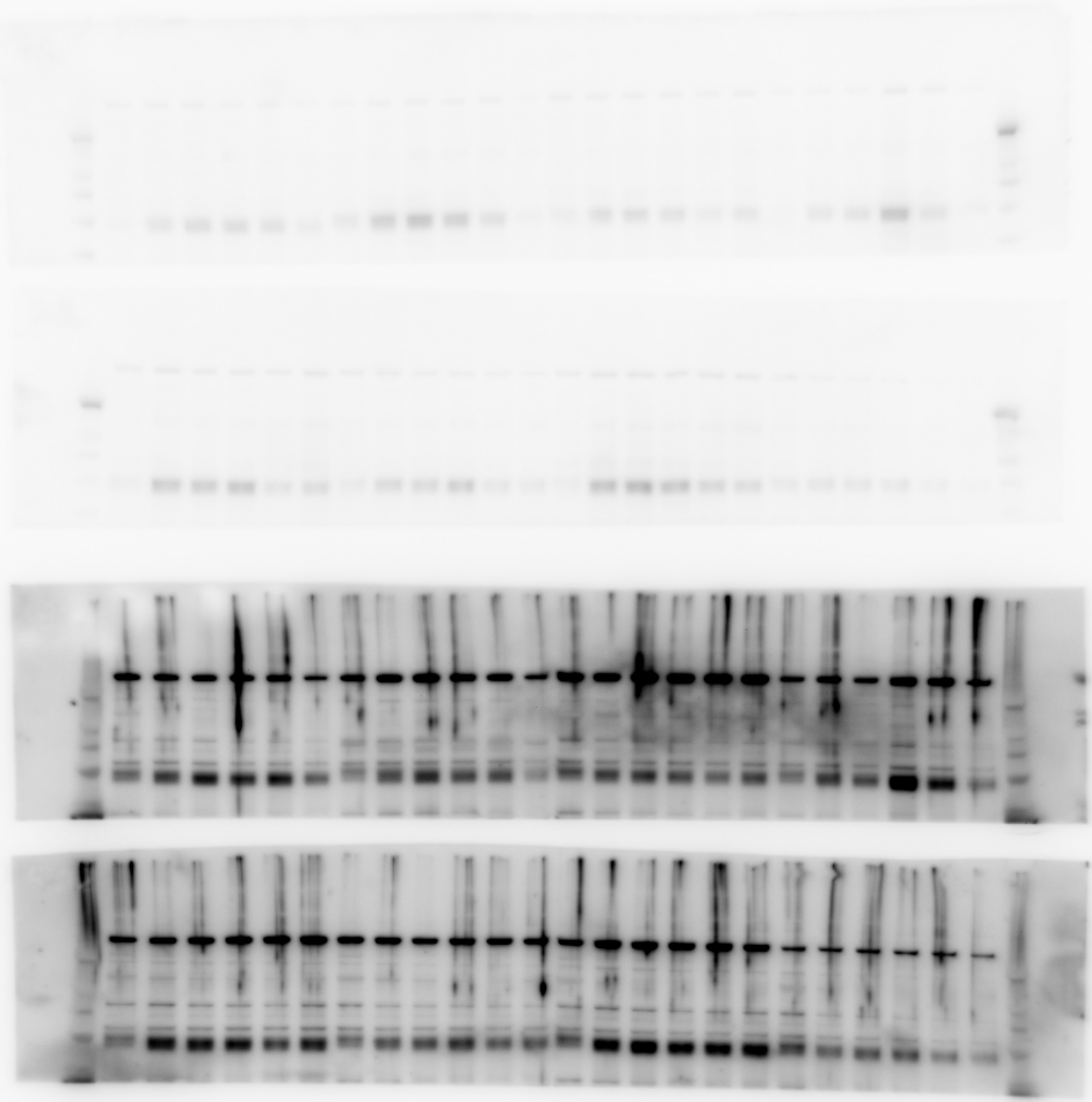

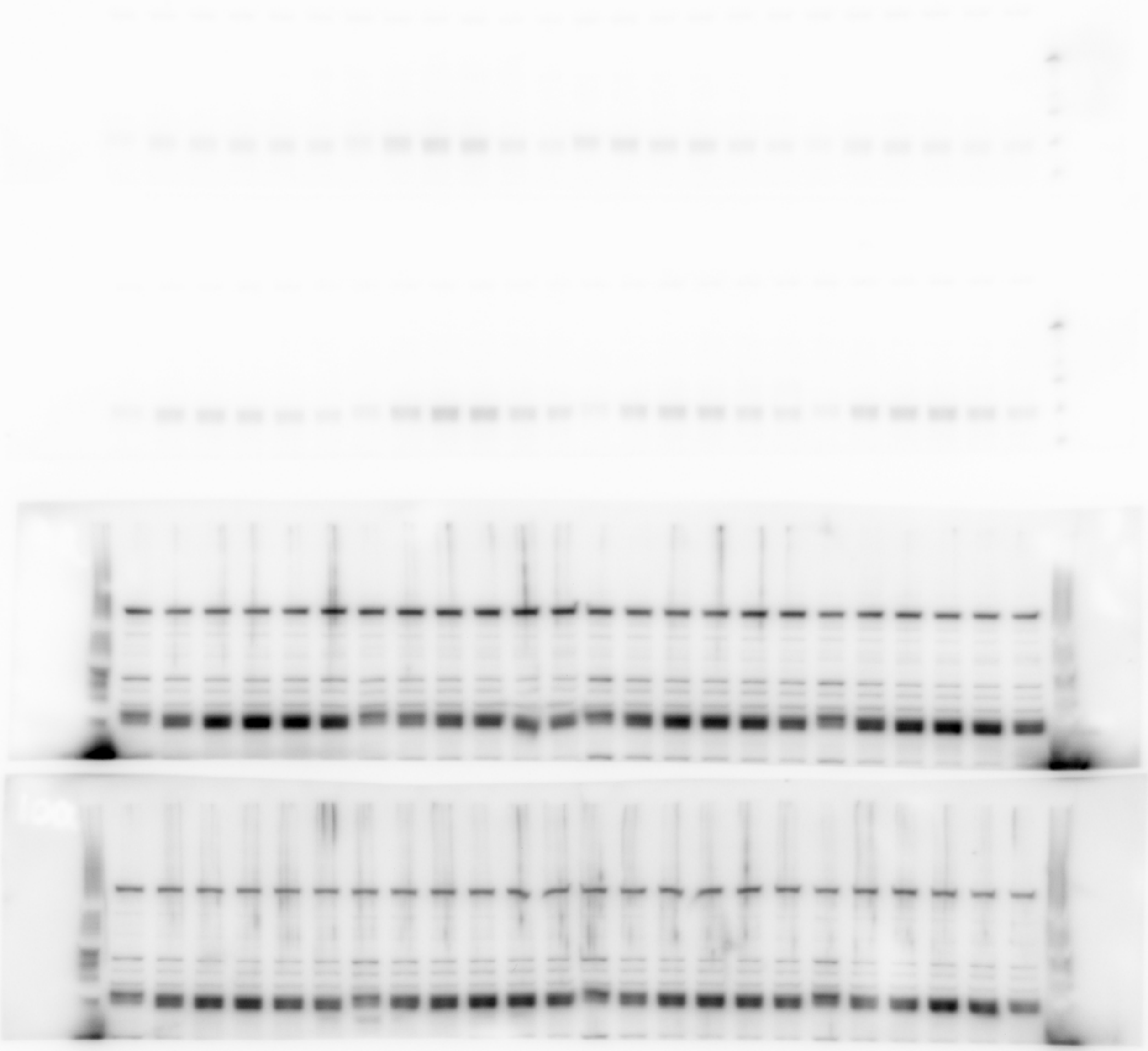

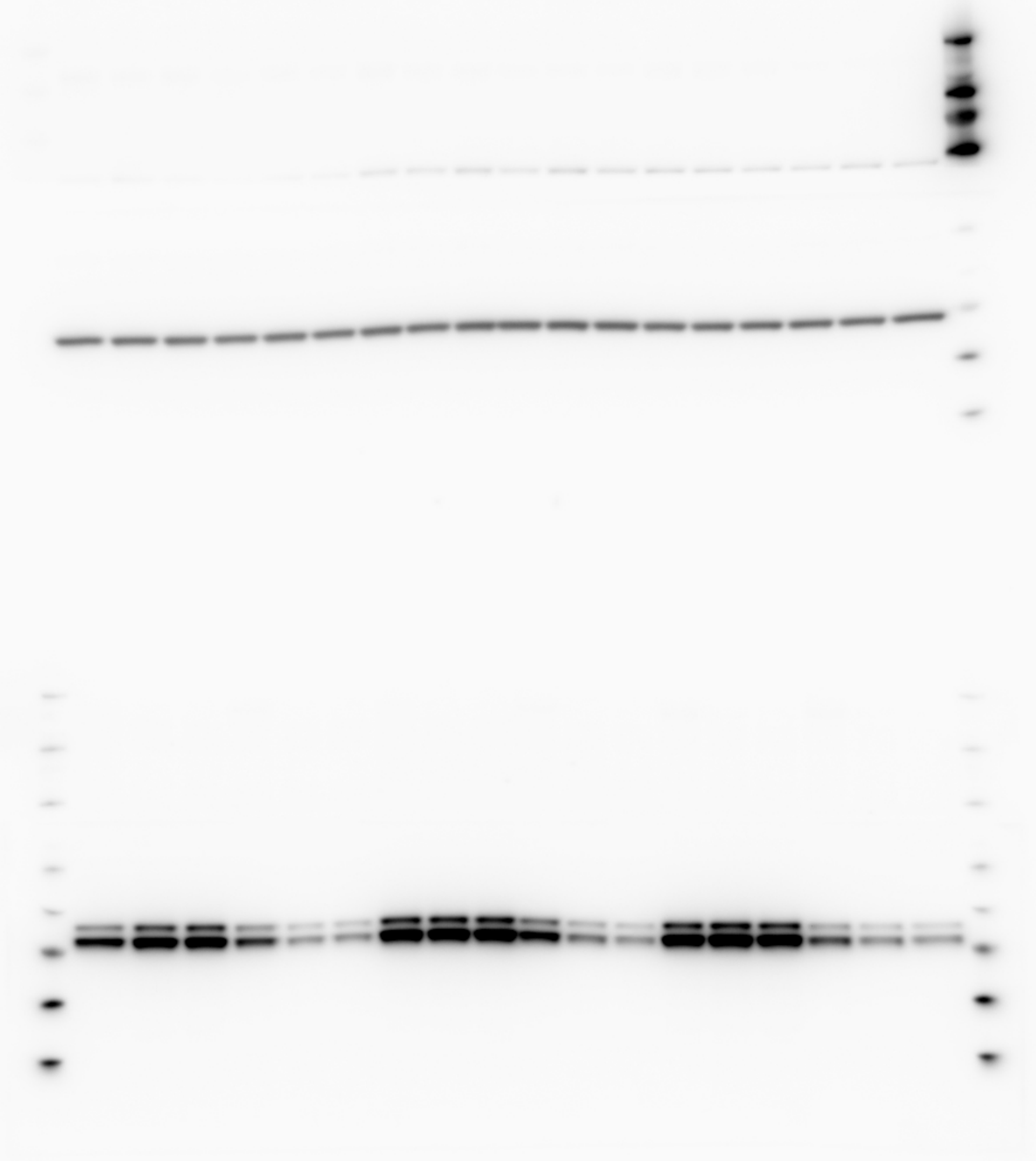

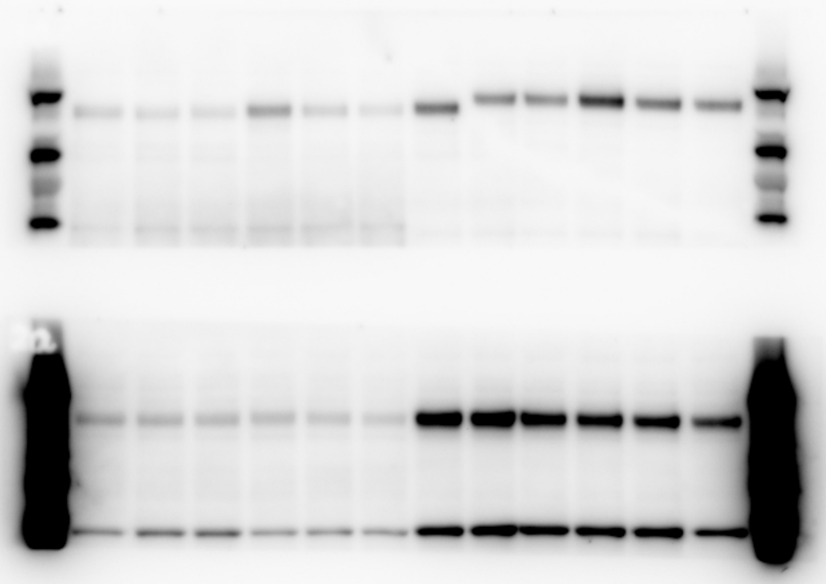

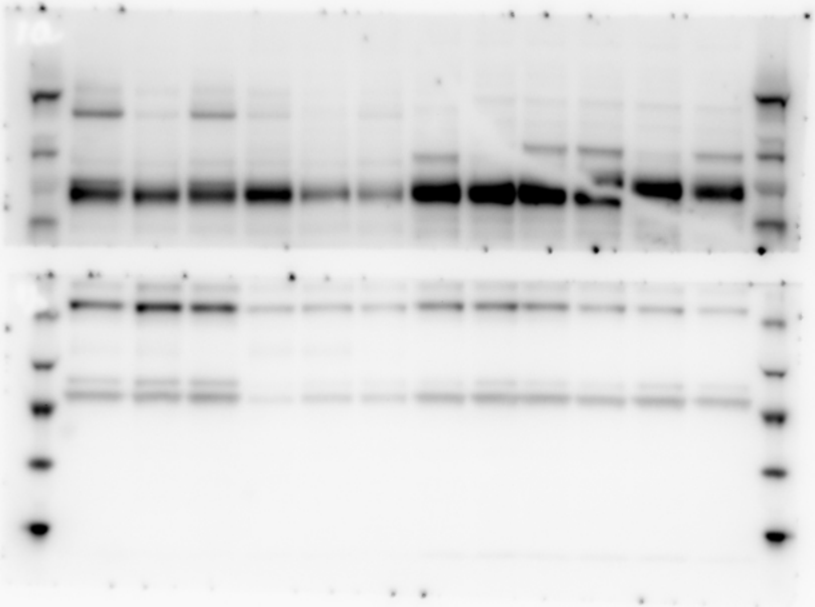

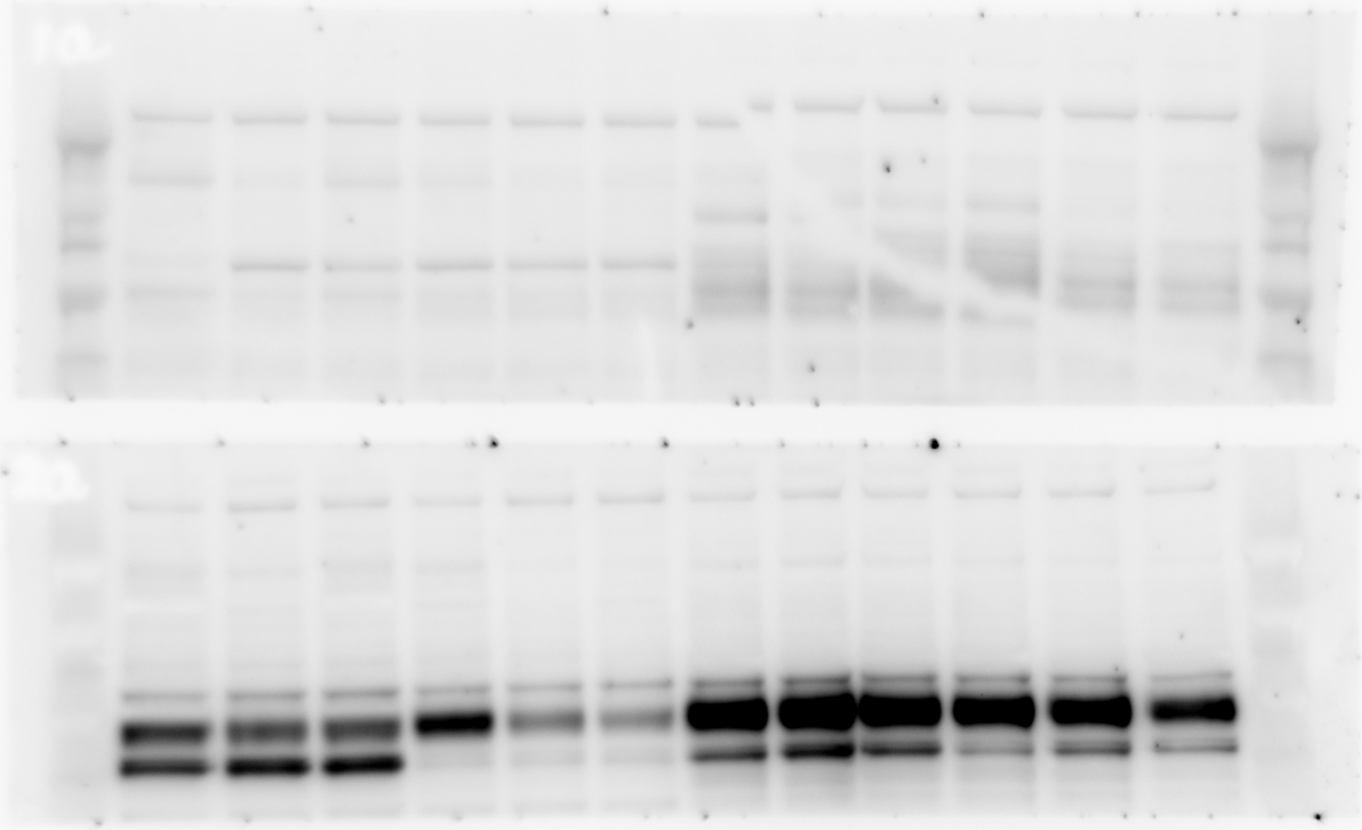
1a
2a

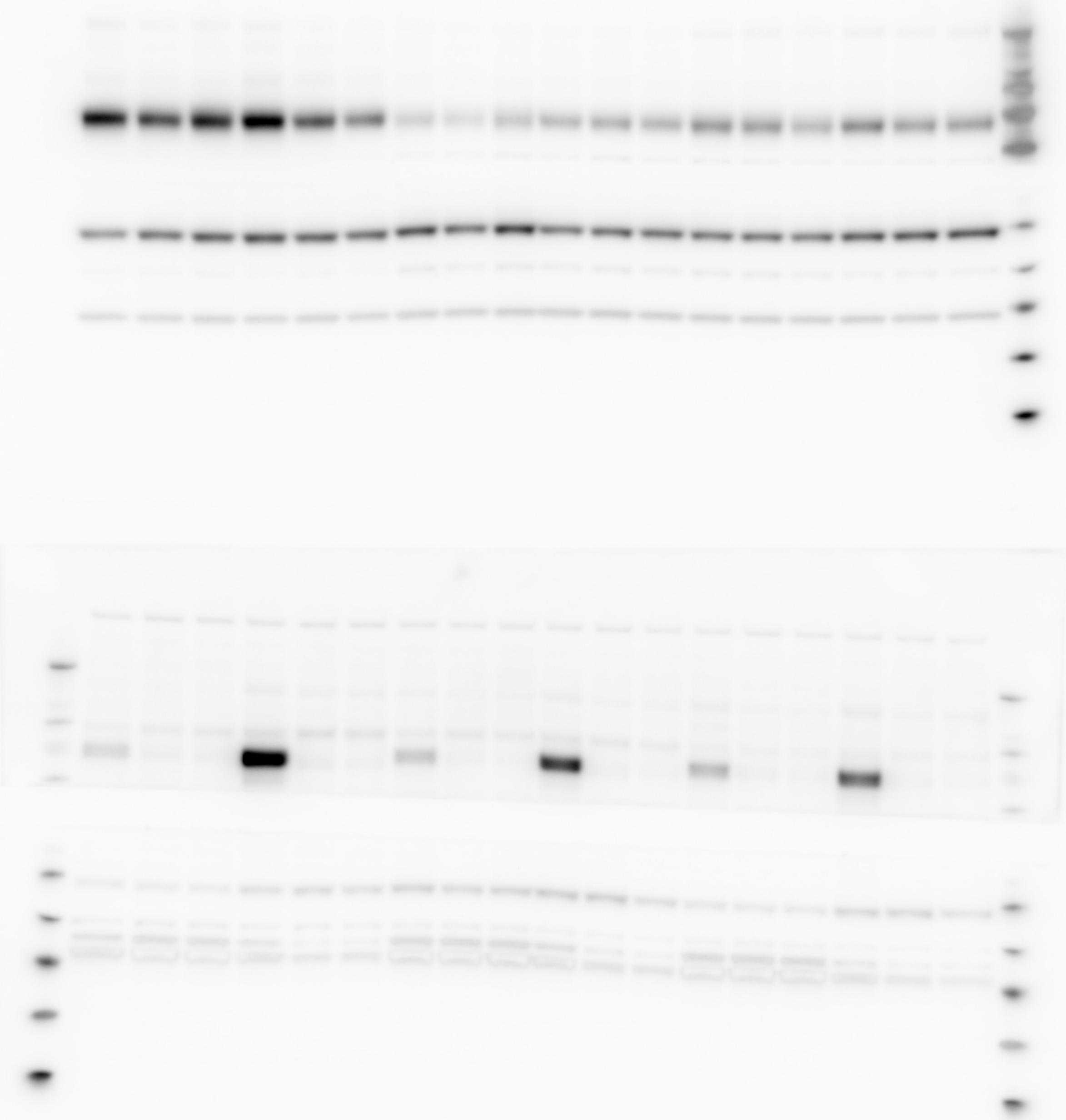

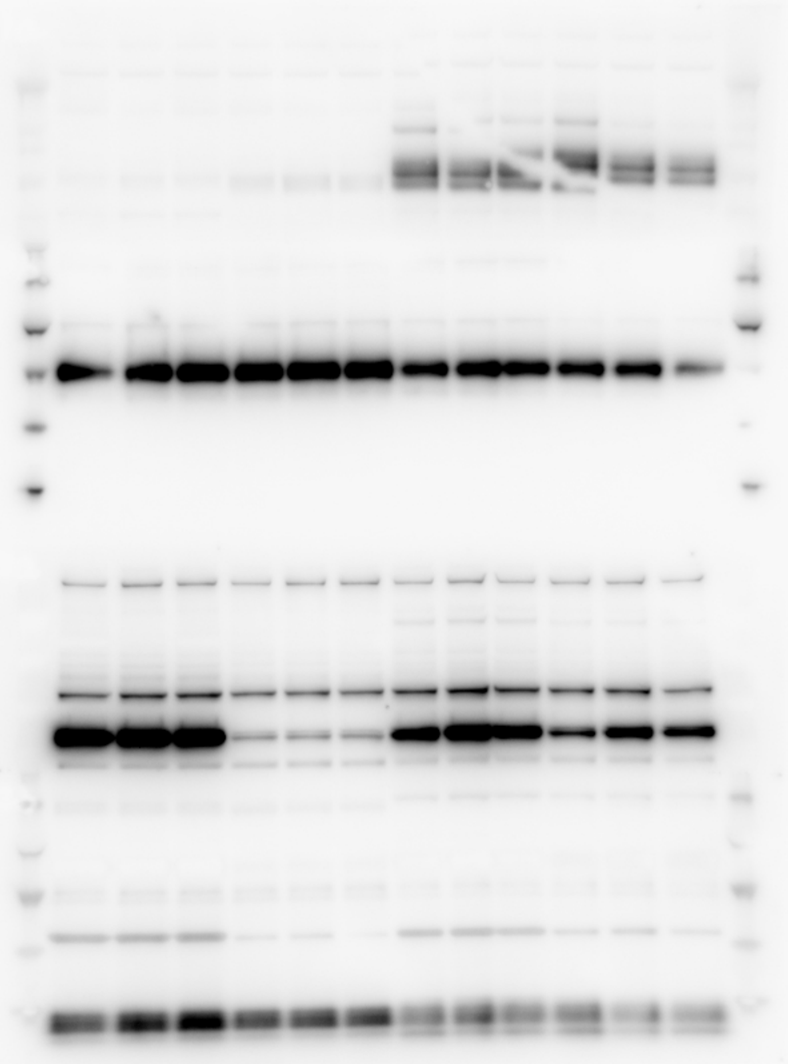

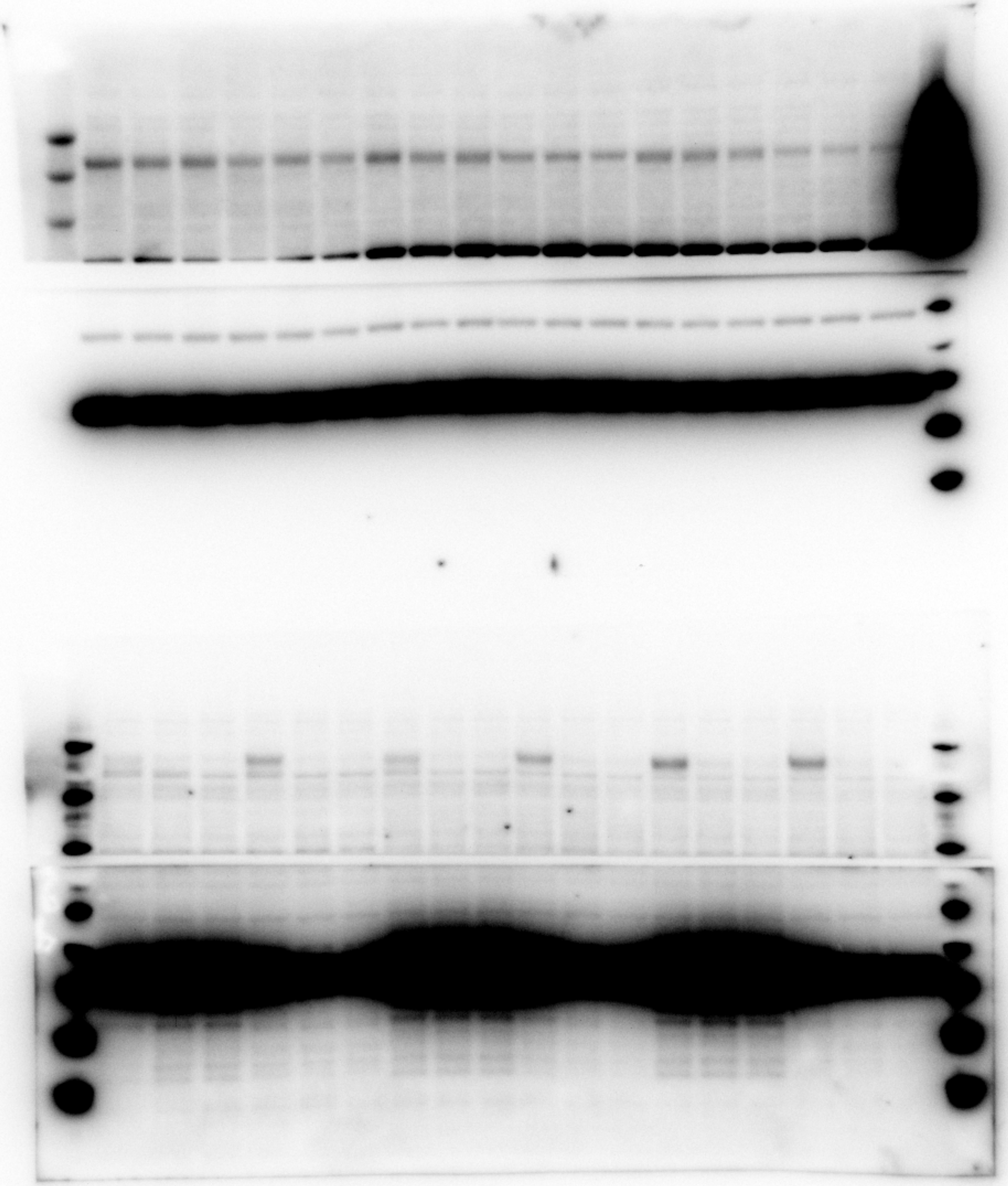

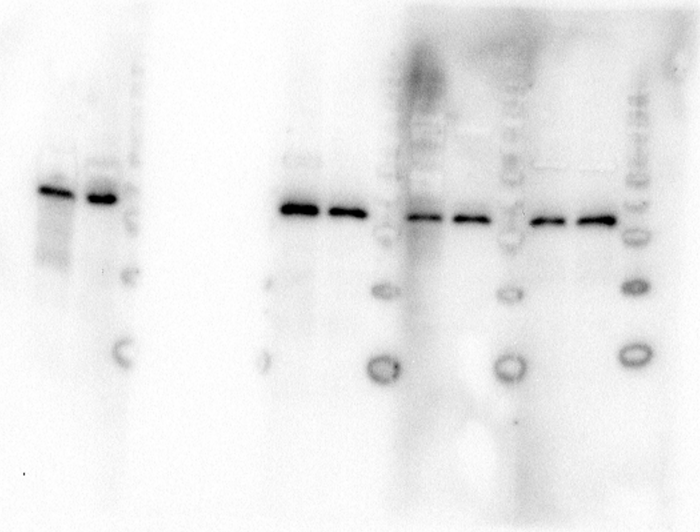

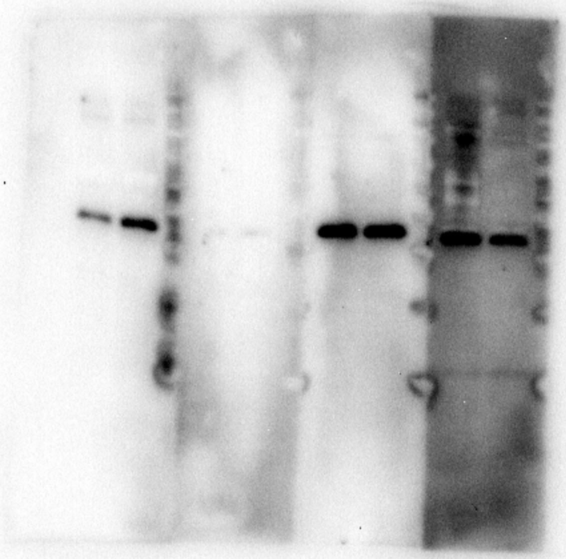

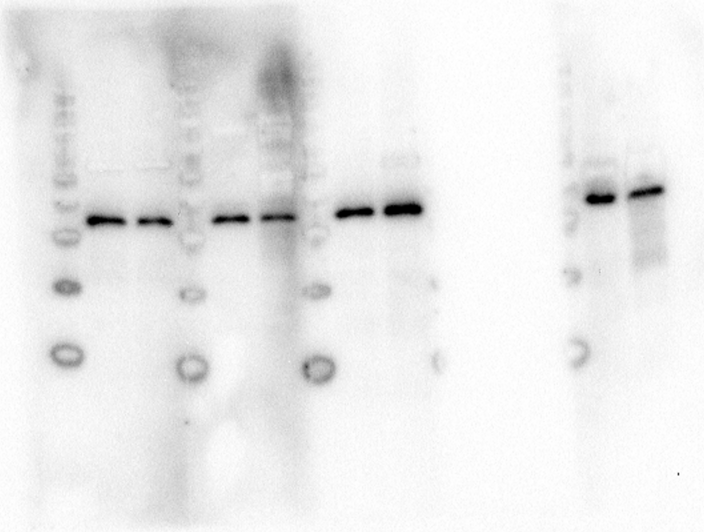

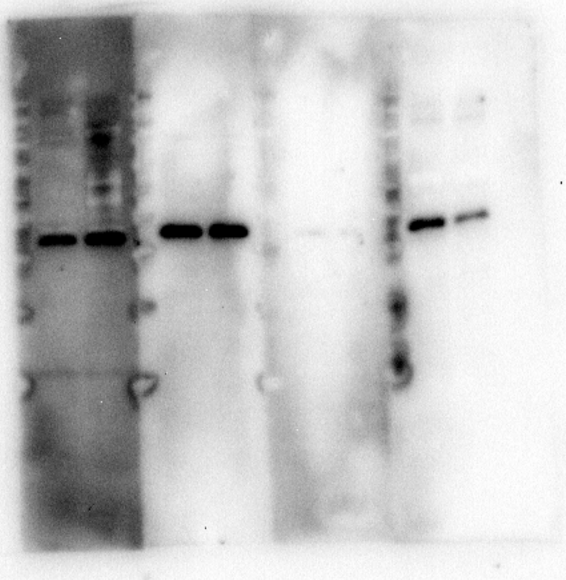

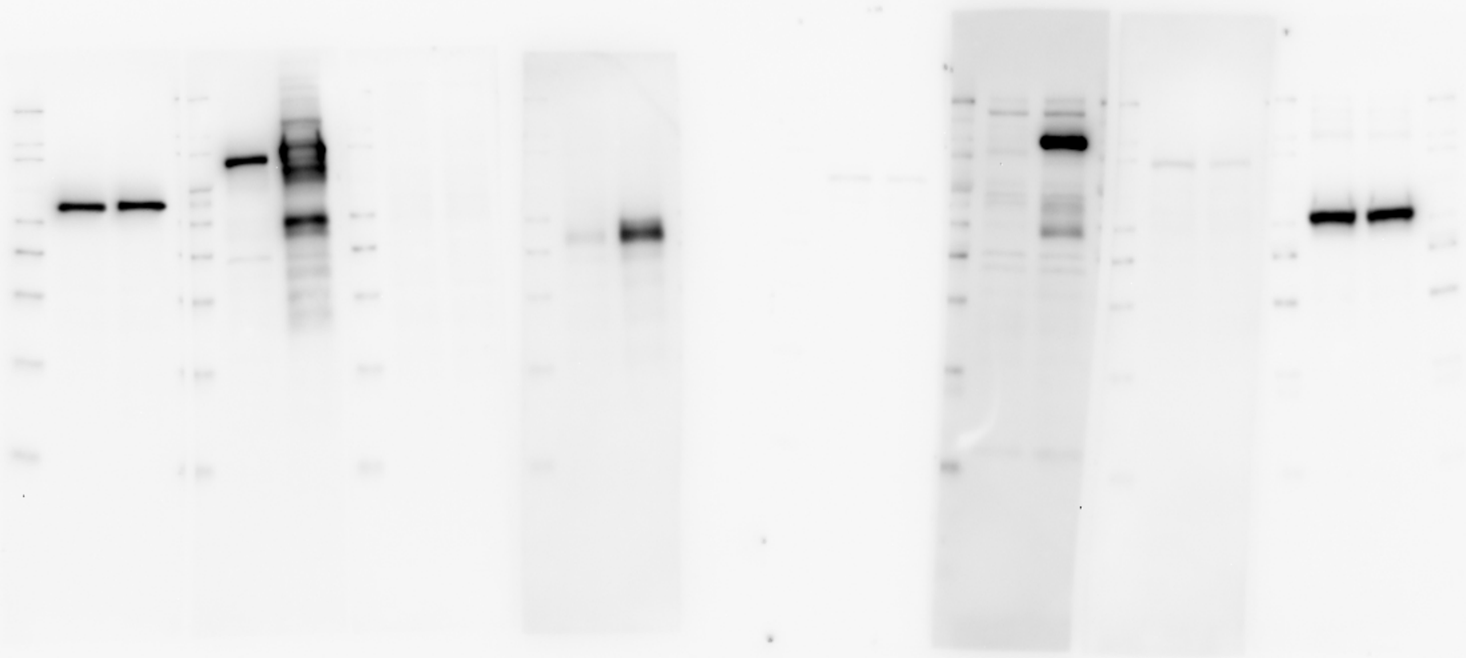

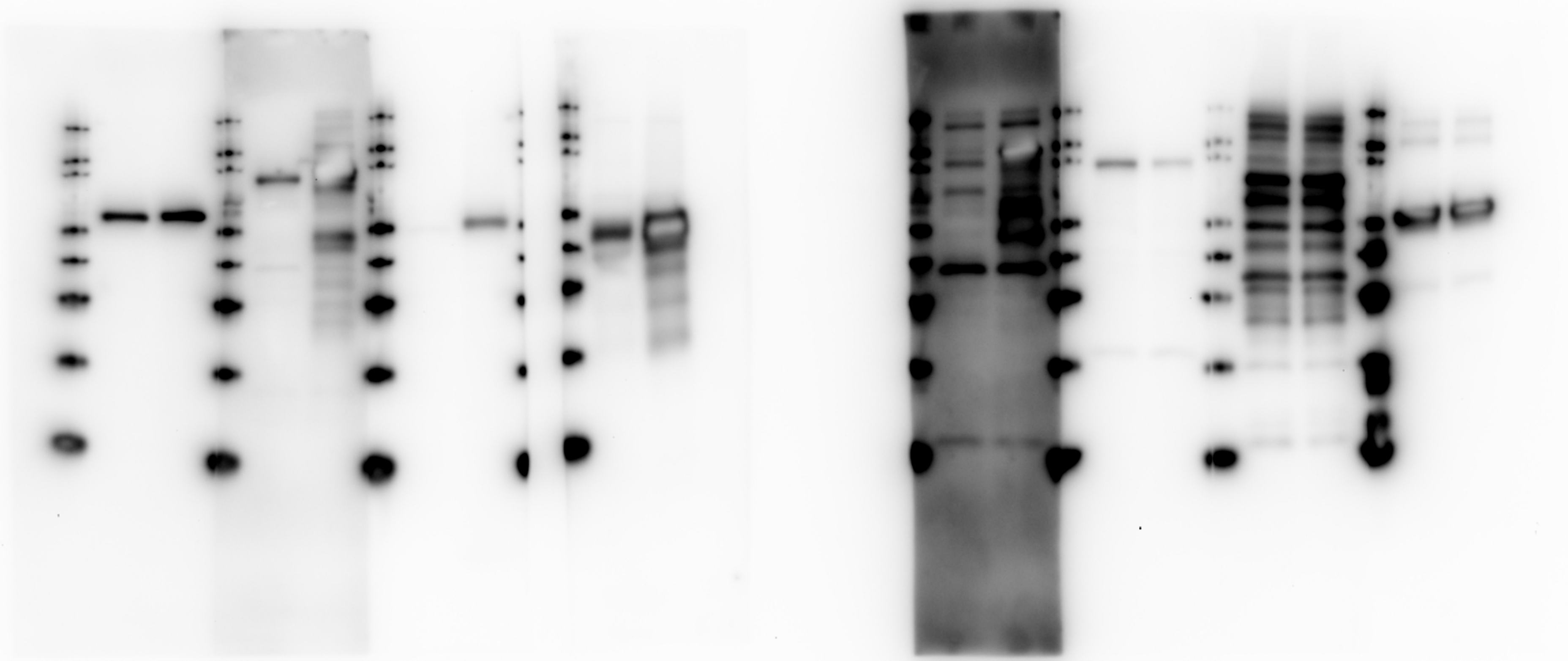

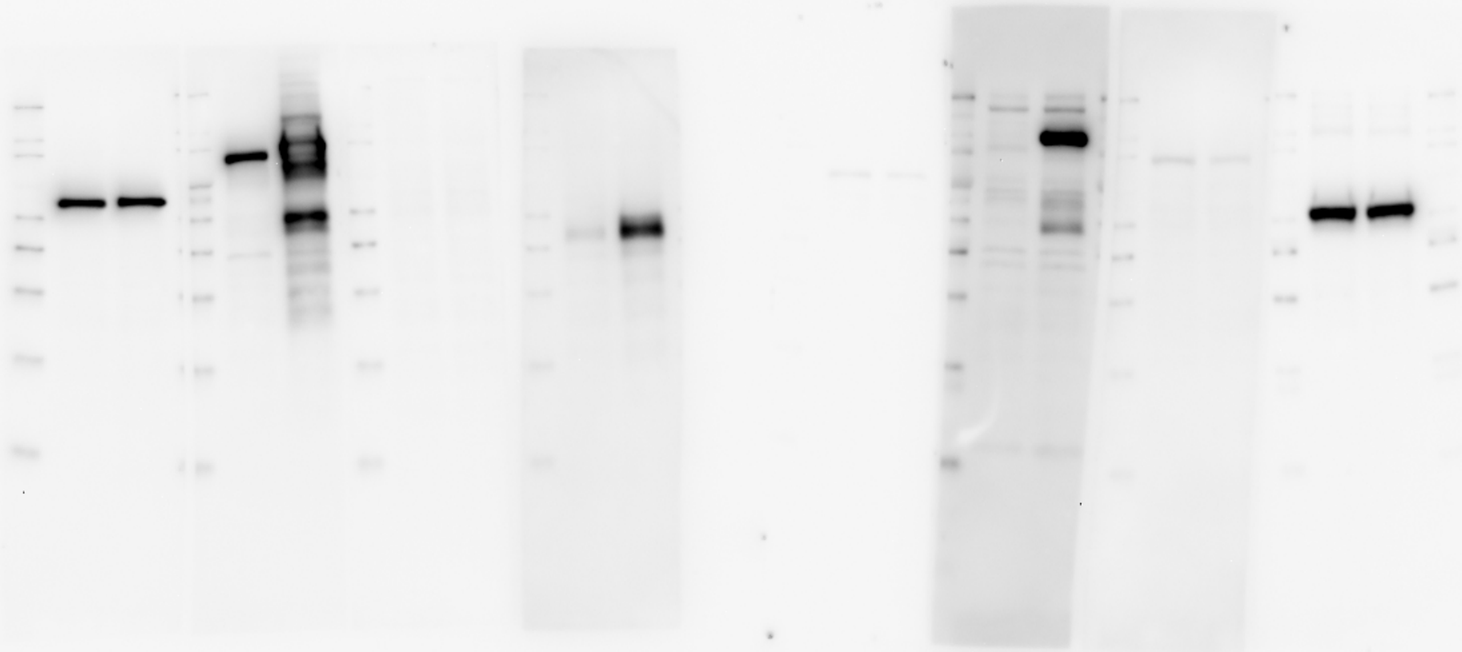

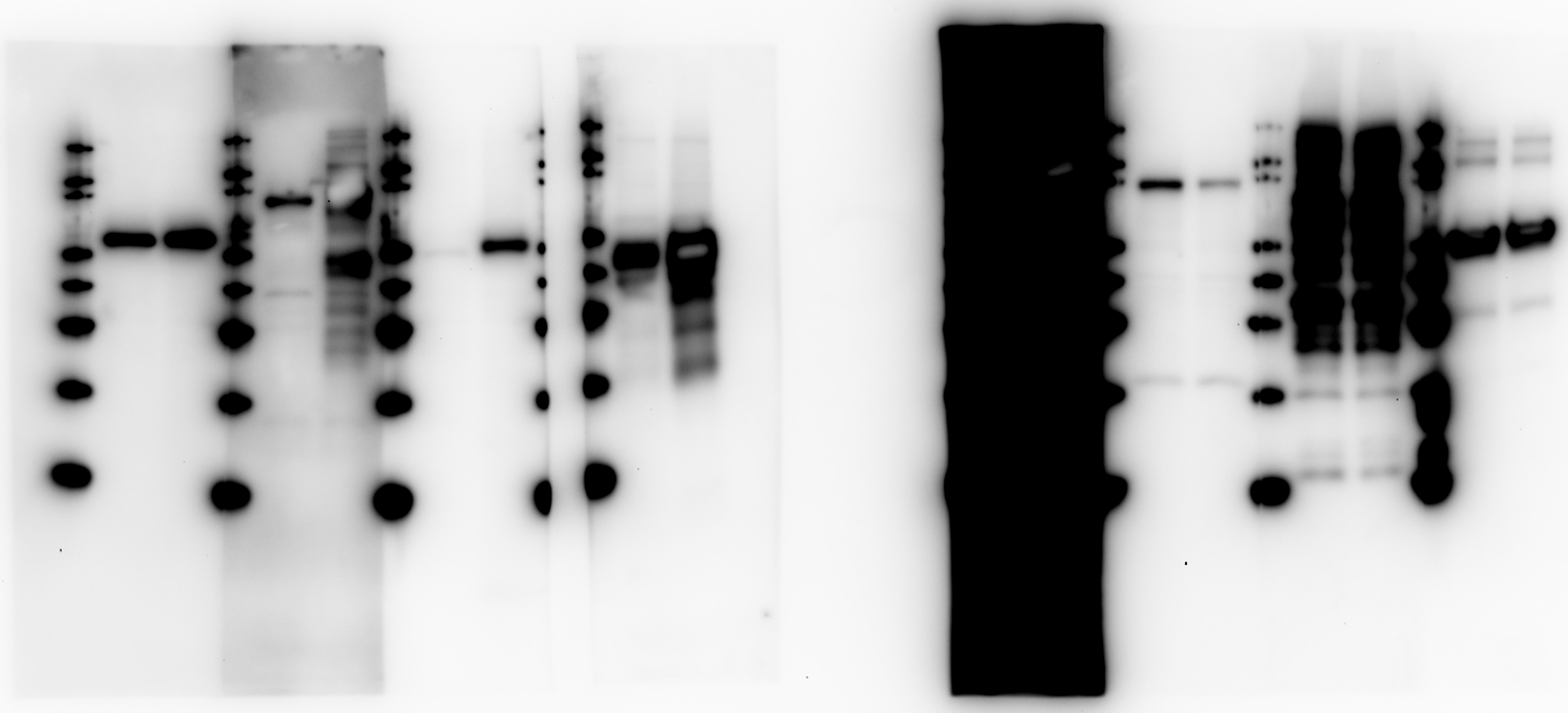

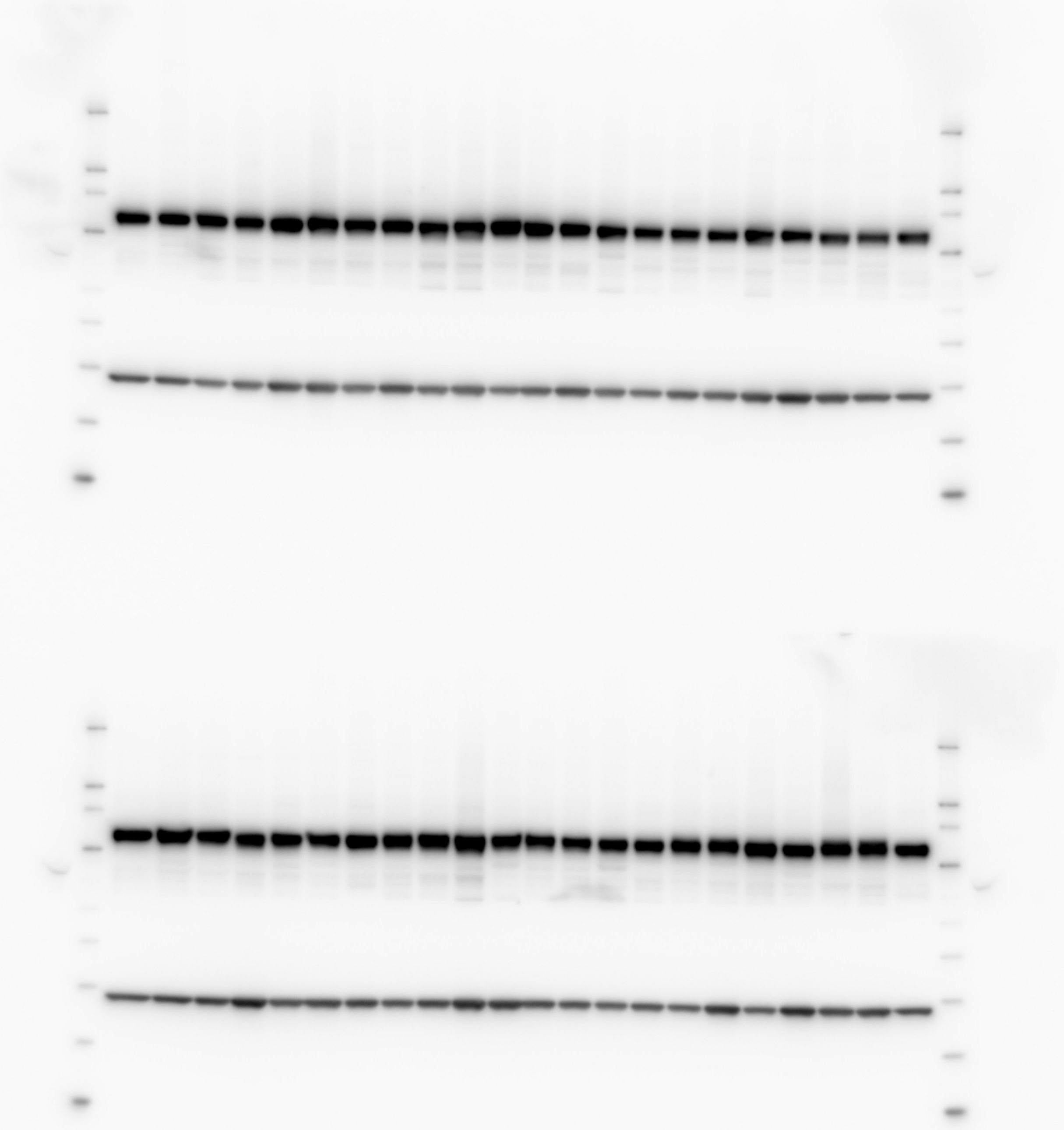

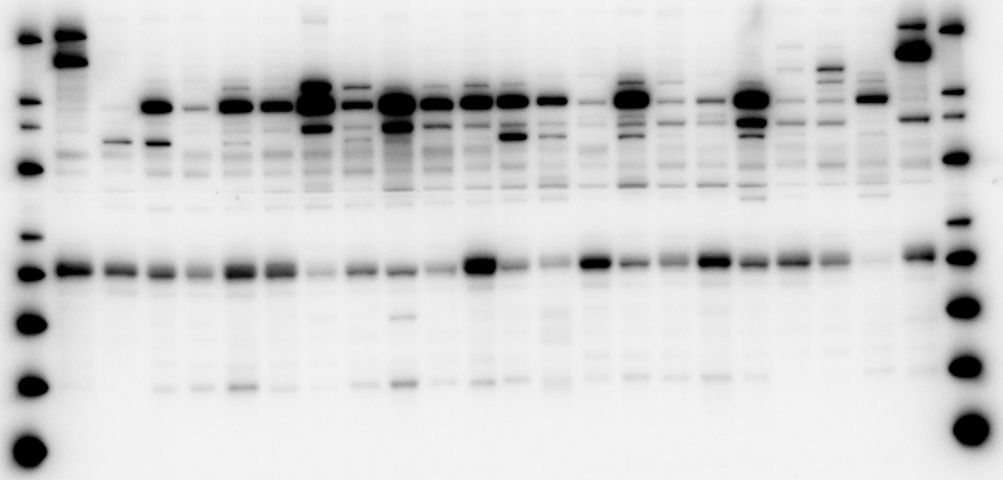

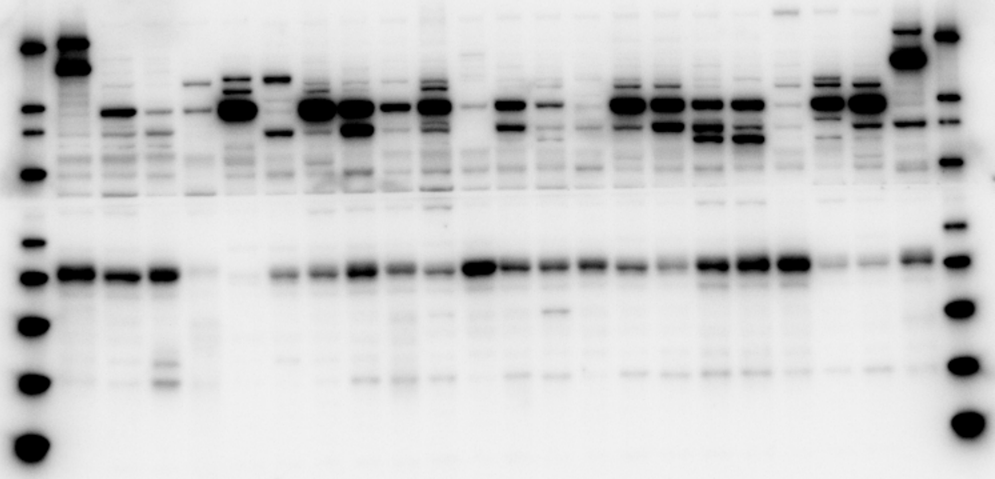

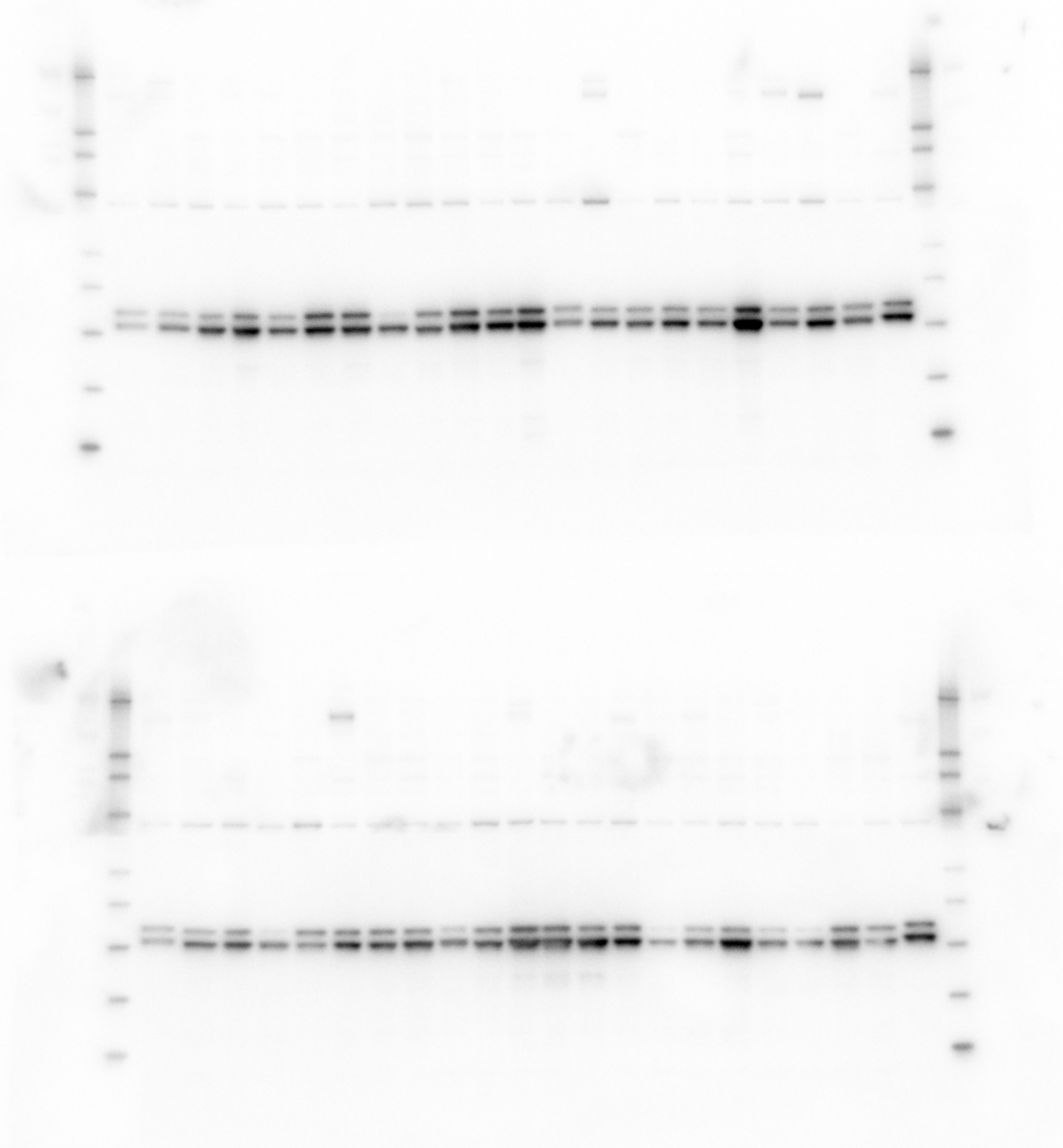

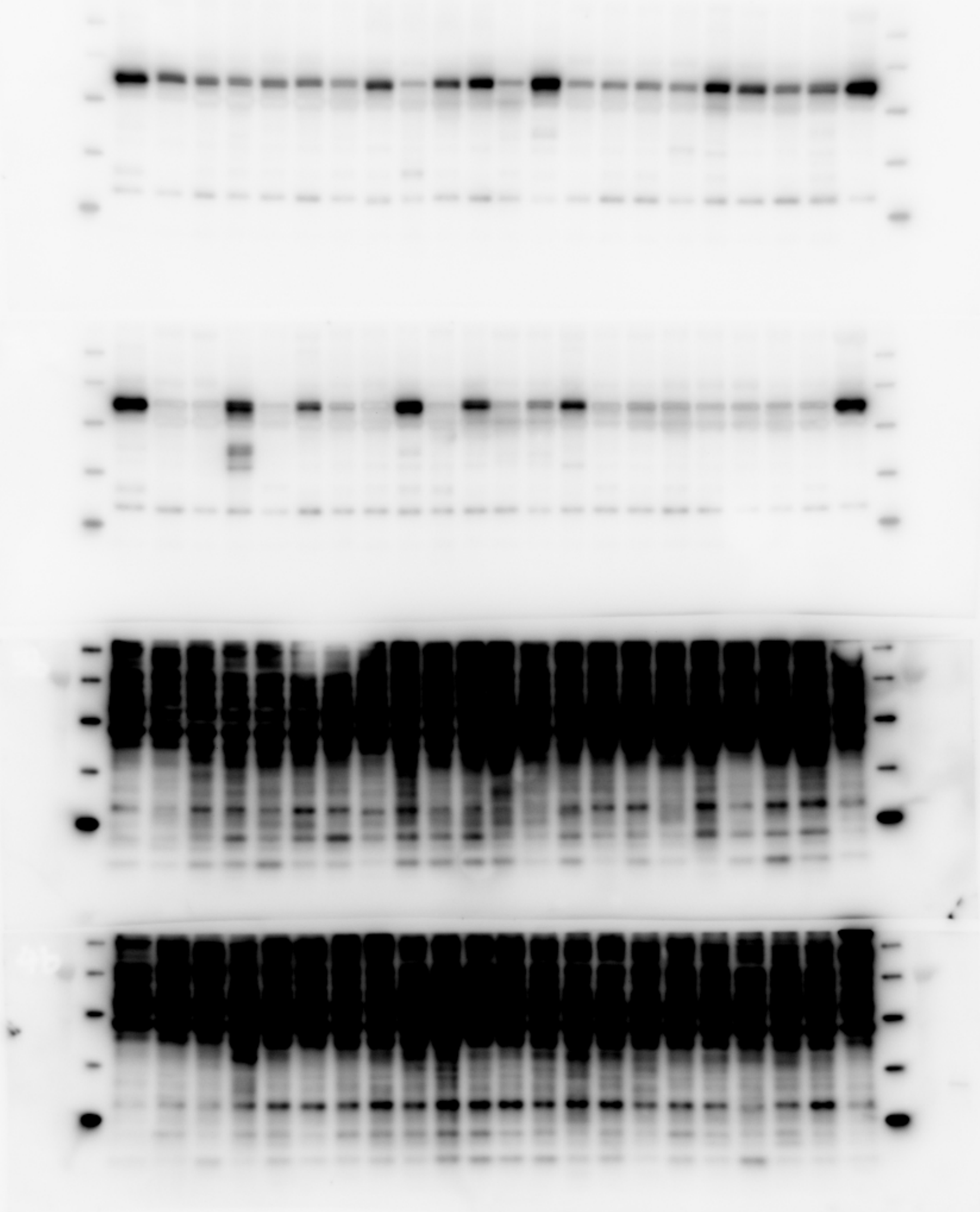

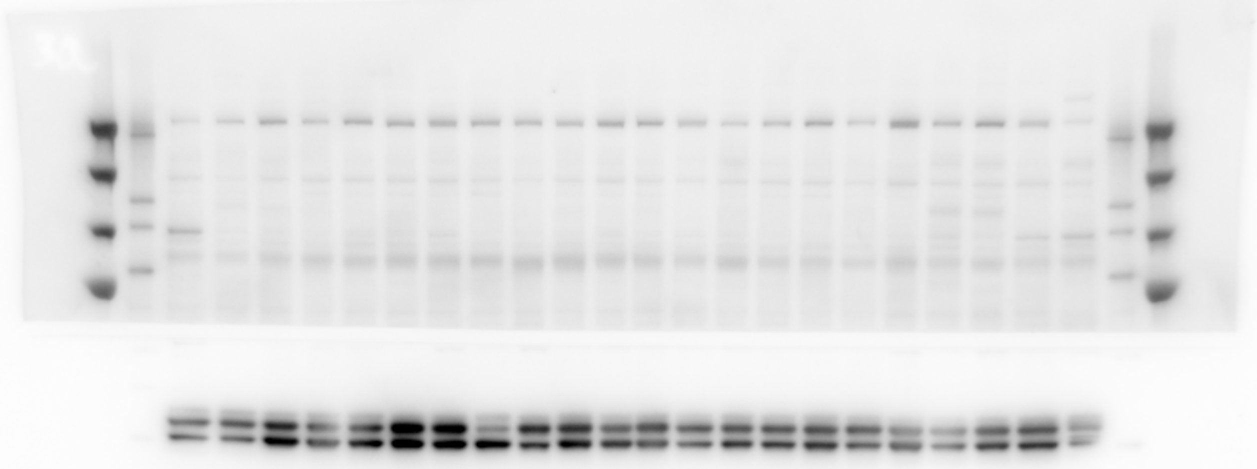

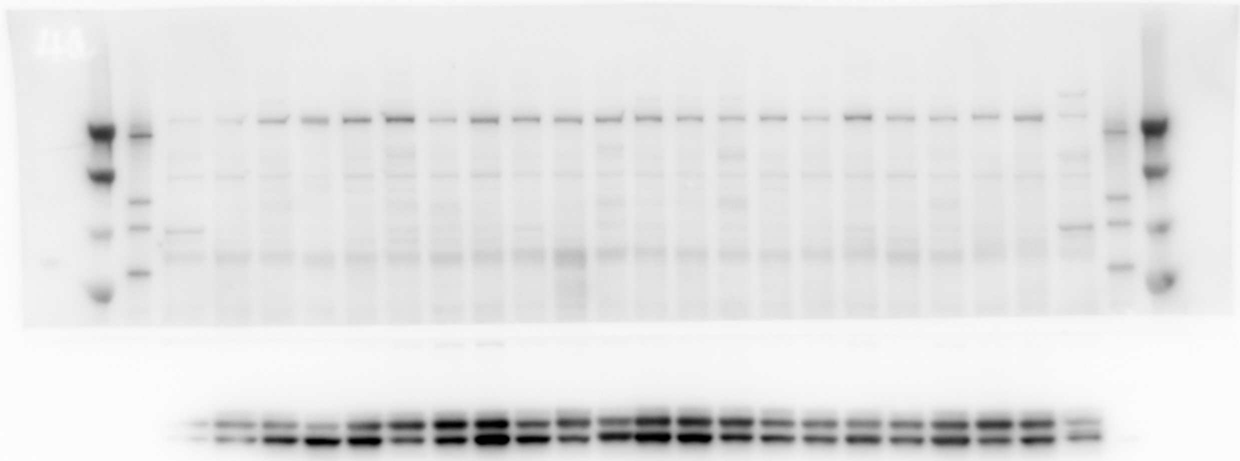

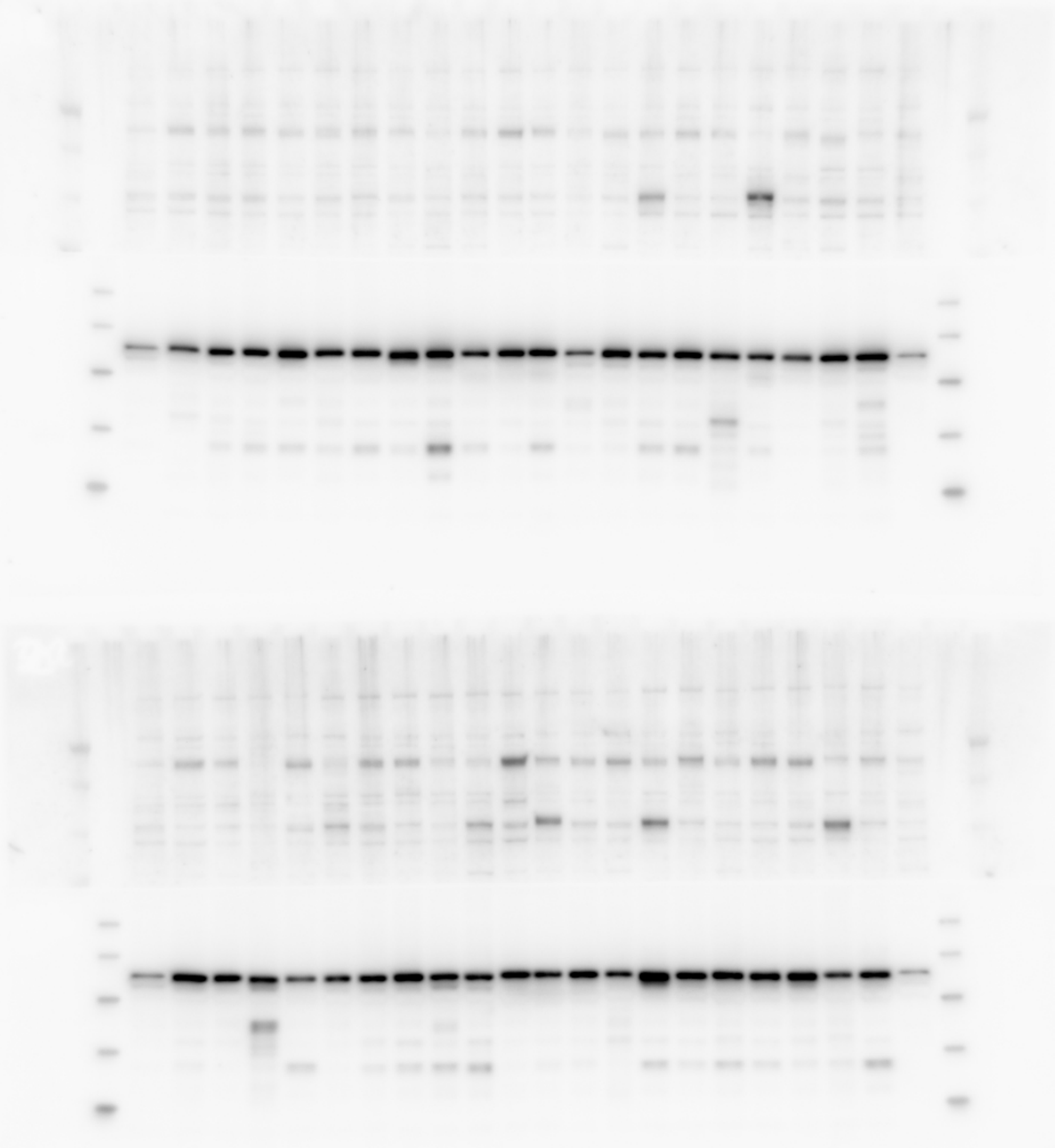

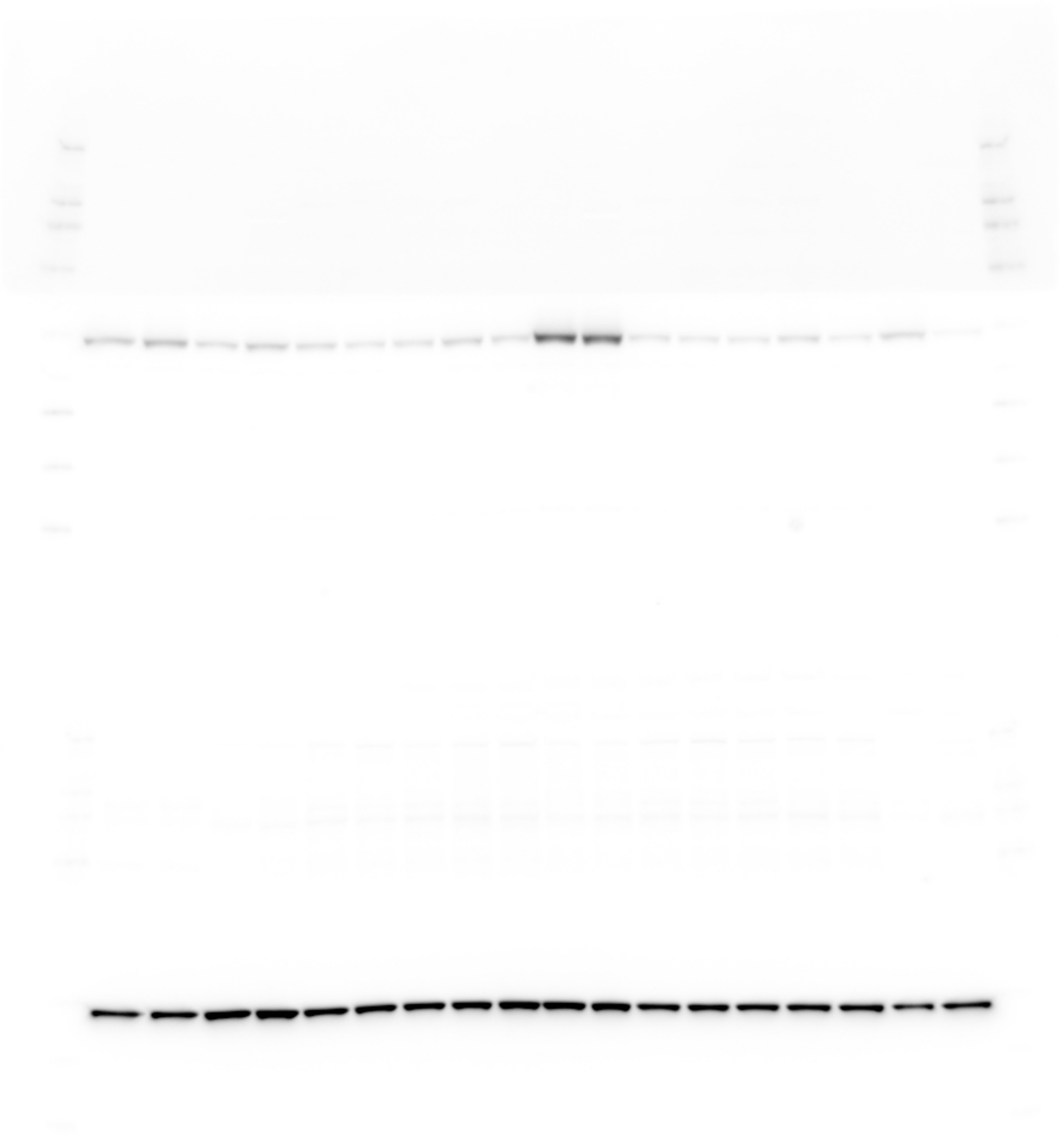

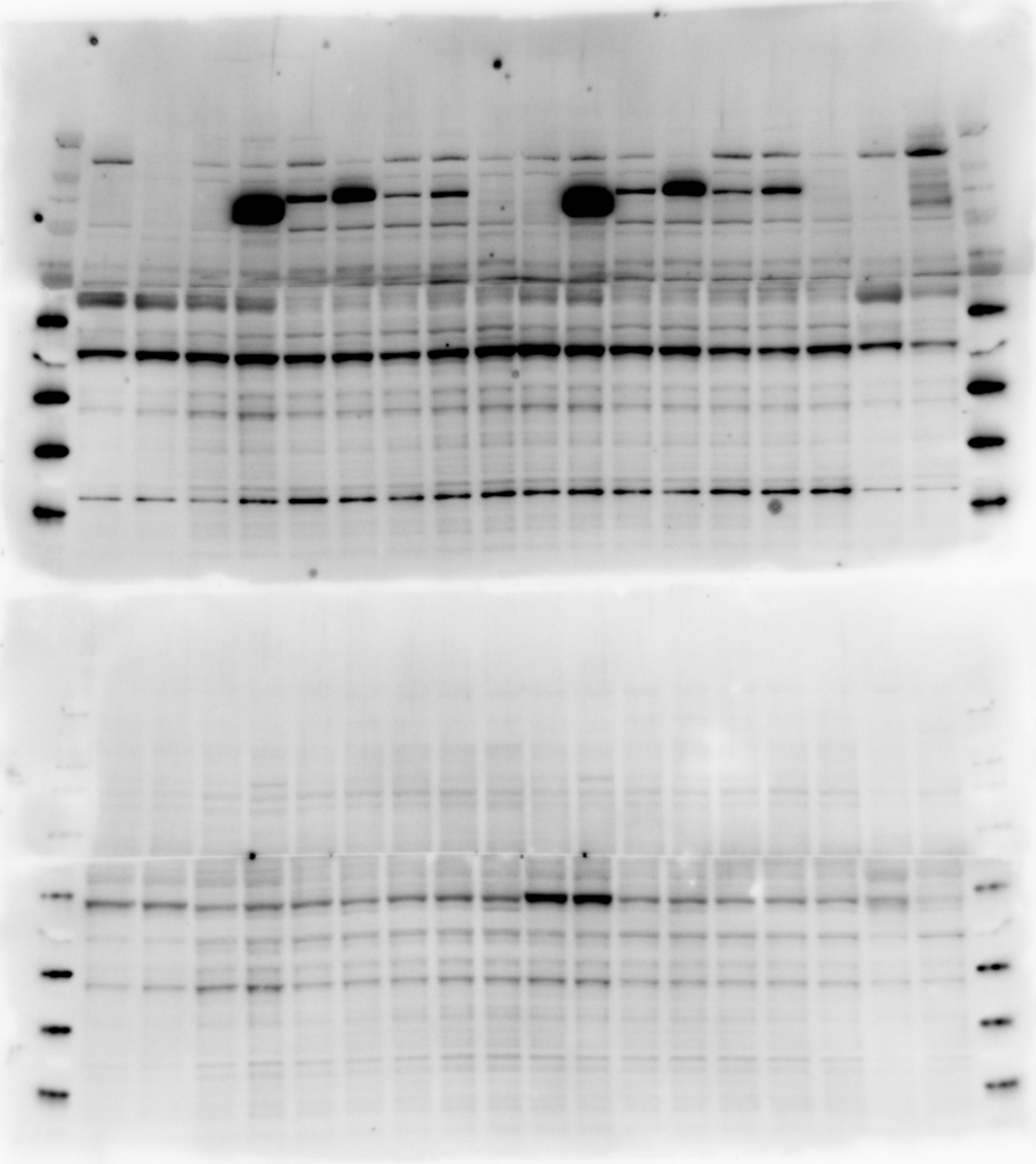

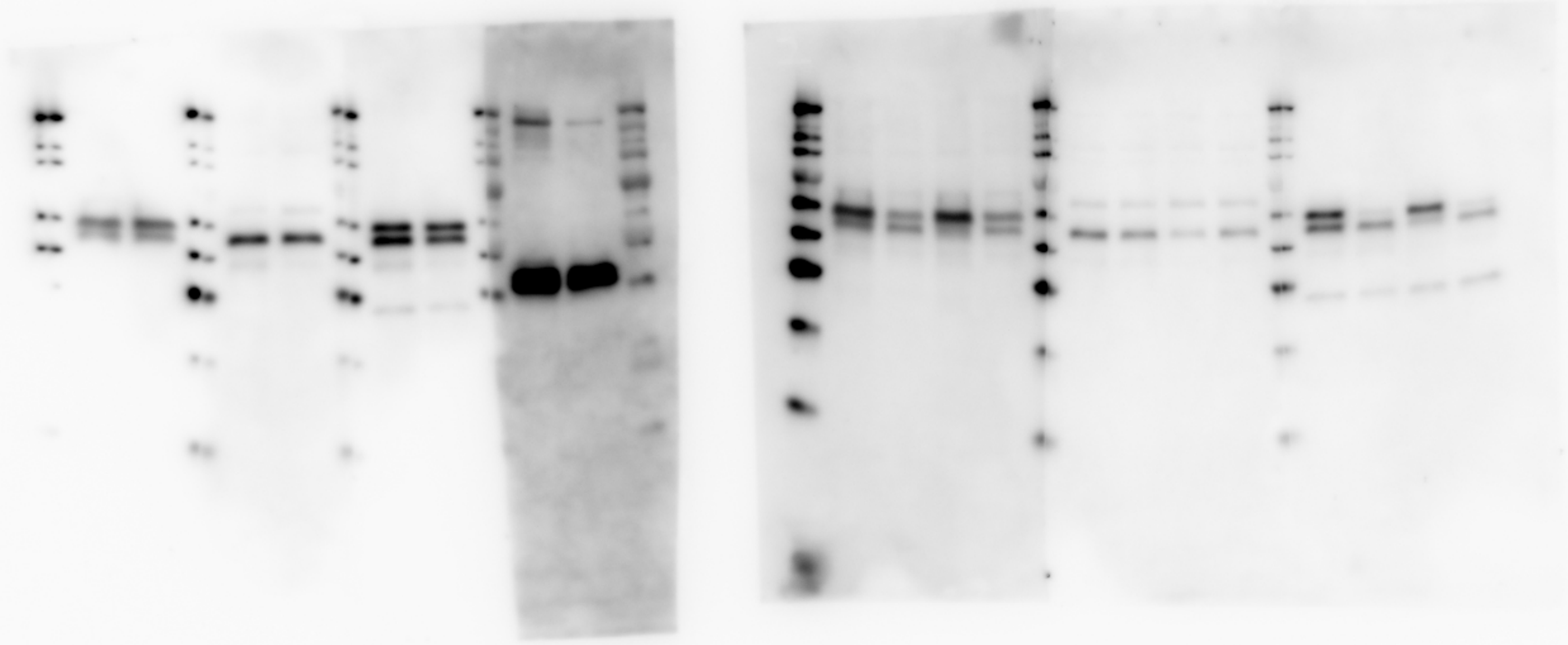

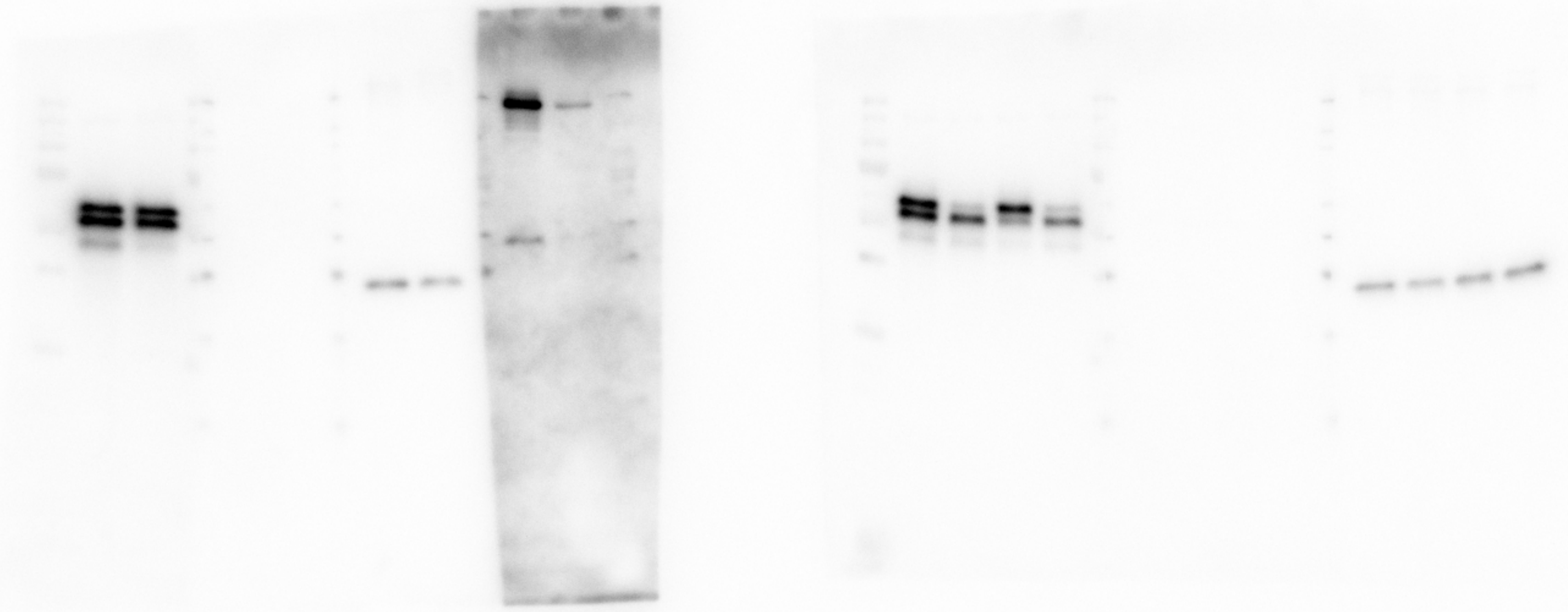

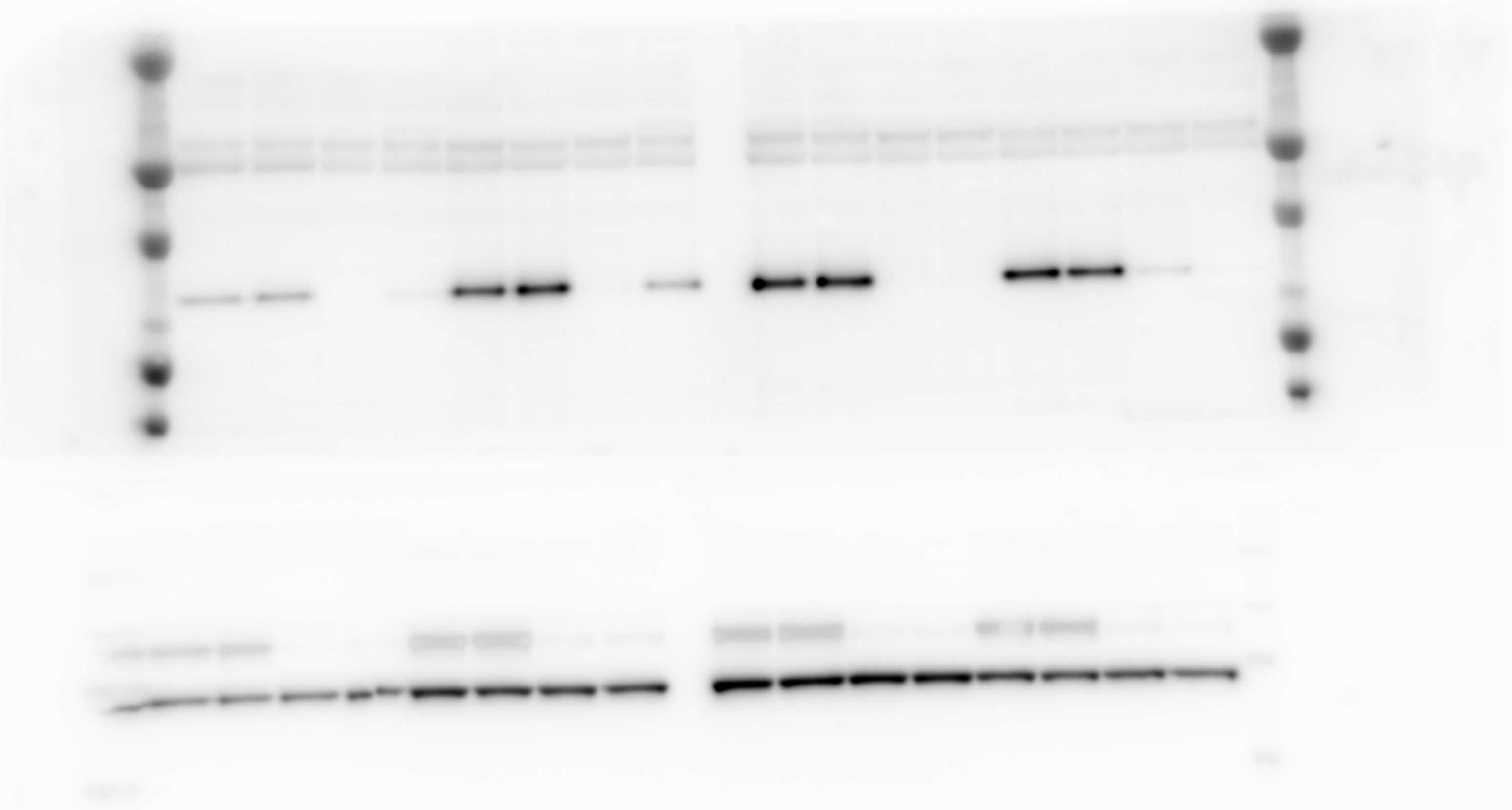

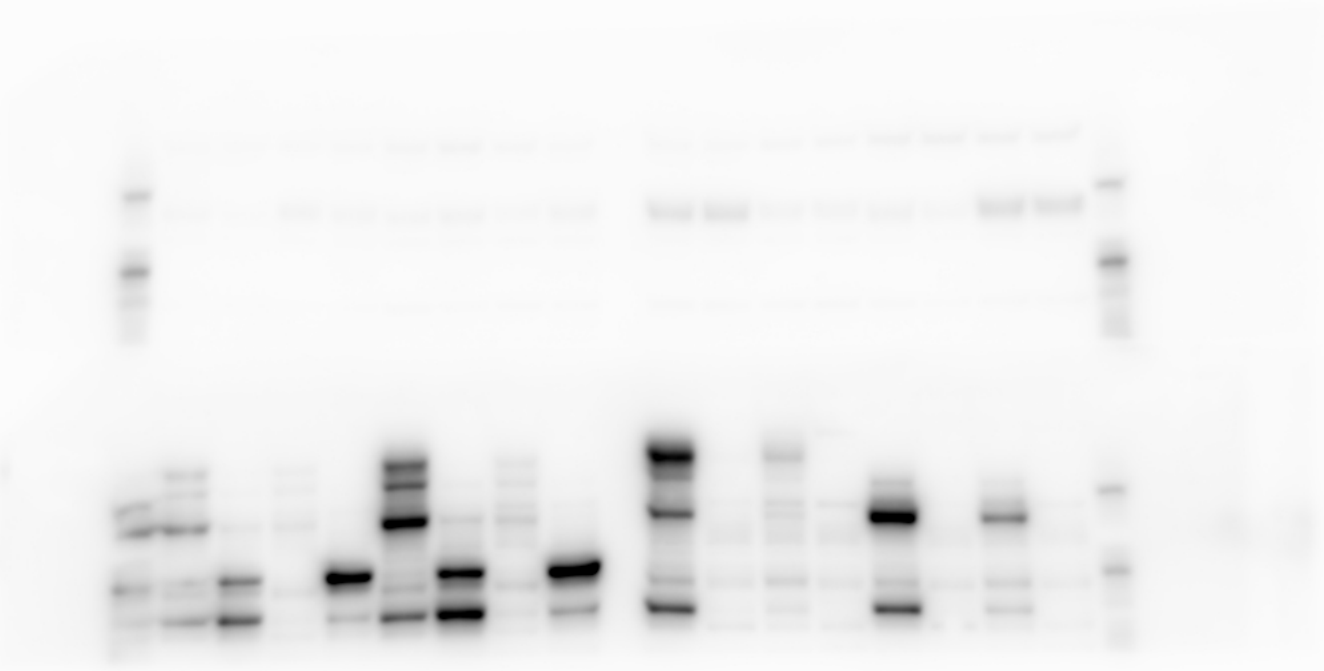

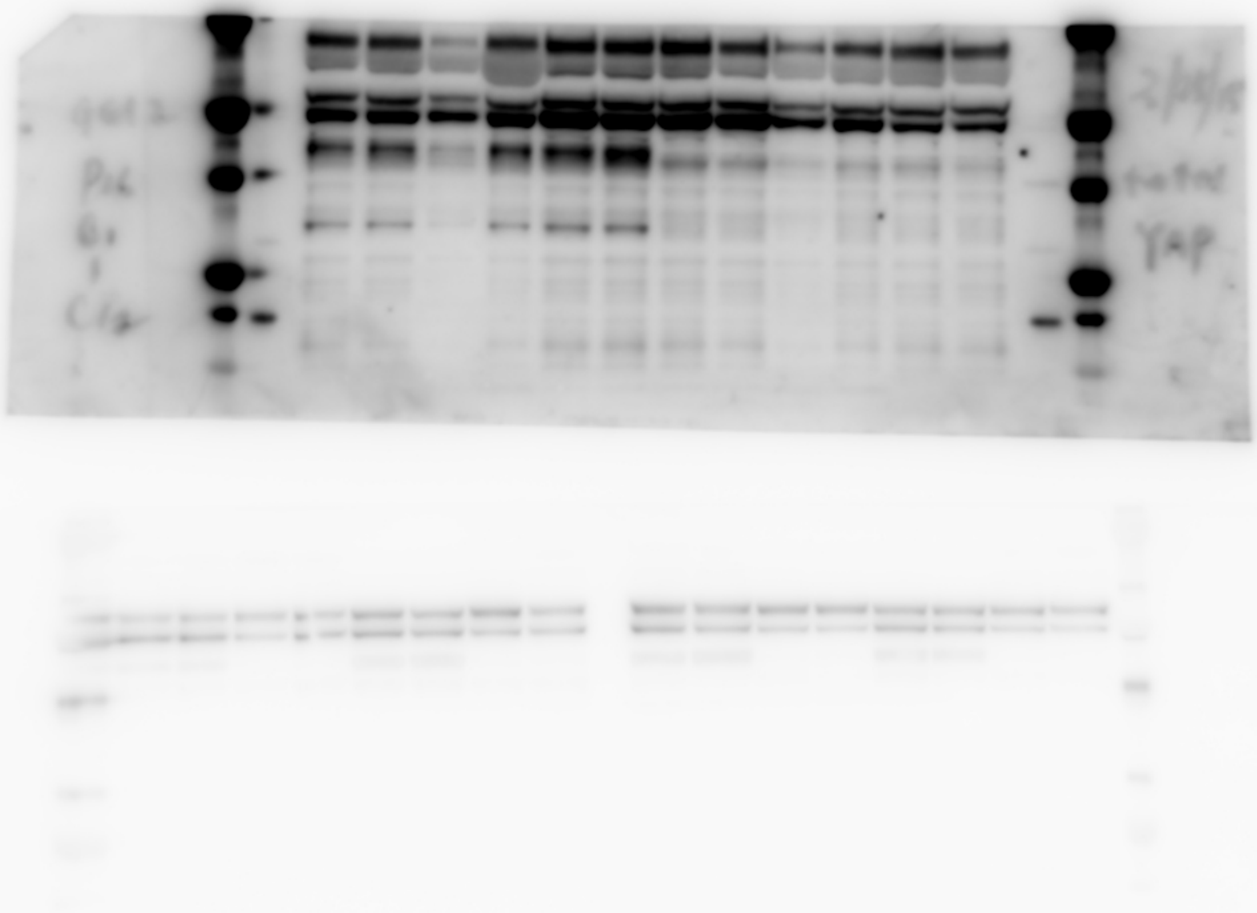
YAP

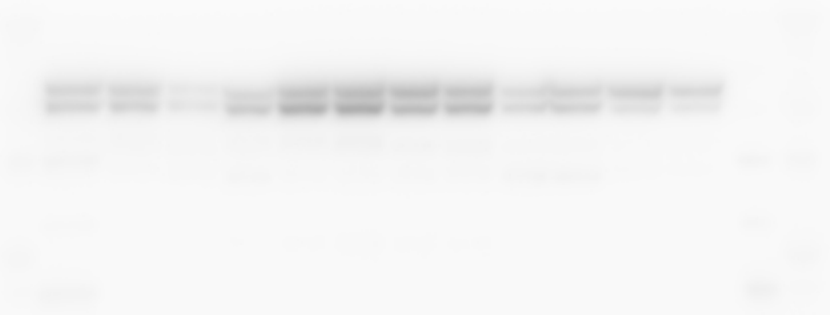

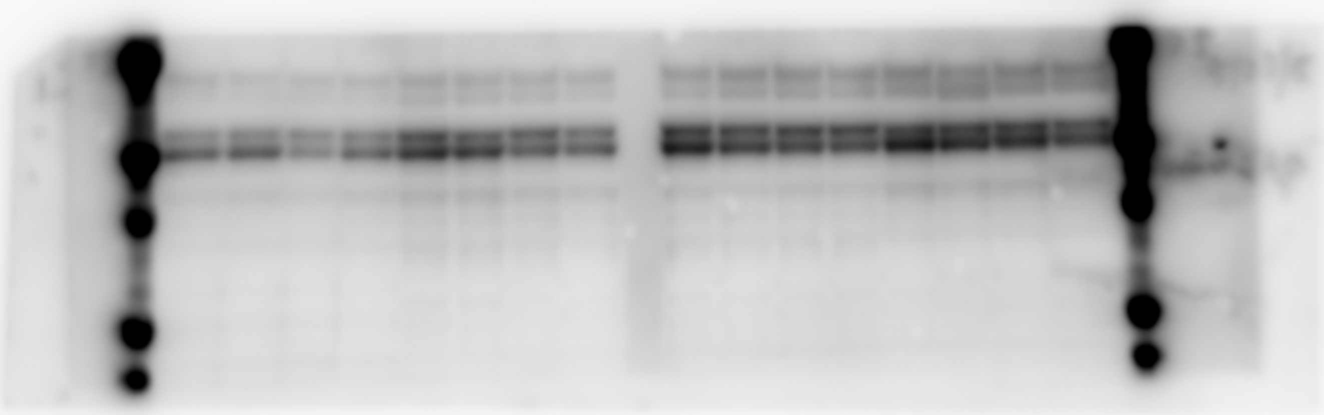

**list of orginal immunoblot image files**

| page | FigName_ProteinName_dirName_subDirName_tiffFileName |
|---|---|
| 164 | Fig3D_GAPDH_WBS131017_WB140125_GAPDH #5 |
| 165 | Fig3D_p4EBP_WBS131017_WB140114a_WB140114 pAKT p4EBP #4 |
| 166 | Fig3D_pAKT308_WBS140506_WB140508_pIGF1Rb pAKT308 exp2 #8 |
| 167 | Fig3D_pAKT473_WBS131017_WB140114a_WB140114 pAKT p4EBP #5 |
| 168 | Fig3D_pERK_WBS131017_WB140108b_tEGFR pERK #5 |
| 169 | Fig3D_pMEK_WBS131017_WB140108b_pEGFR pMEK #5 |
| 170 | Fig3D_pRSK_WBS131017_WB140117a_WB140117 pRSK tRSK #8 |
| 171 | Fig3D_pS6_WBS131017_WB140113a_WB140113 pS6 tS6 #3 |
| 172 | Fig3D_pS6k_WBS131017_WB140110a_pS6K tS6K #5 |
| 173 | Fig3D_tERK_WBS131017_WB140109b_pMTOR tERK |
| 174 | Fig4B_WWTR1_WBS140415_WB140424_WWTR1 pAKT473 #5 |
| 175 | Fig4B5J_NEDD4L_GAPDH_WBS140415_WB140425_NEDD4L pERK GAPDH #4 |
| 176 | Fig4D_GAPDH_WBS150323_WB04102015_top(GAPDH)_bot(pS6K)#4 |
| 177 | Fig4D_GAPDH_WBS150323_WB04102015_top(GAPDH)_bot(pS6K)#5 |
| 178 | Fig4D_pYAP_WBS150323_WB04132015_plate 16 E1-E12_p-YAP#5 |
| 179 | Fig4D_tYAP_WBS150323_WB04132015_plate 16 E1-E12_total-YAP#4 |
| 180 | Fig4F_GAPDH_WBS150521_WB05282015_17E1-E12_GAPDH#2 |
| 181 | Fig4F_TEAD1_WBS150521_WB05262015_17E1-E12_top(WWTR1)-bot(TEAD1)#3 |
| 182 | Fig4F_TEAD1_WBS150521_WB05262015_17E1-E12_top(WWTR1)-bot(TEAD1)#5 |
| 183 | Fig4F_TEAD1_WBS150521TEAD1_WB05252015_17E1-E12_top(WWTR1)-bot(TEAD1)#5 |
| 184 | Fig4F_TEAD1_WBS150521TEAD1_WB05262015_17E1-E12_top(WWTR1)-bot(TEAD1)#3 |
| 185 | Fig5C_GAPDH_WBS140519_WB140530_gel 9a10a-tRSK 9b10b-GAPDH#4 |
| 186 | Fig5C_NEDD4L_WWTR1_WBS140519_WB140523_gel 9a10a-NEDD4L 9b10b-WWTR1#4 |
| 187 | Fig5F_pAKT473_WBS150506MKsiPLEK_WB05082015_top(16H1-H12)-bot(17C1-D8)_pAKT473#5 |
| 188 | Fig5F_tubulin_WBS150521_WB05272015_top(16H1-H12)_bot(17C1-D8)_tubulin#4 |
| 189 | Fig5H_GAPDH_WBS131017_WB140124a_WB140124a GAPDH#8 |
| 190 | Fig5H_GAPDH_WBS140519_WB140530_gel 9a10a-tRSK 9b10b-GAPDH#4 |
| 191 | Fig5H_pEGFR1068_WBS131017_WB140108b_pEGFR pMEK #5 |
| 192 | Fig5H_pEGFR1068_WBS131017_WB140113a_WB140113 tEGFR pEGFR #5 |
| 193 | Fig5H_pGab1_WBS131017_WB140124_WB140124 pGAB1#8 |
| 194 | Fig5H_pGAB1_WBS140519_WB140521_gel 7a8a-pGAB1 9a10a-tGAB1#8 |
| 195 | Fig5H_pIGF1Rbeta_WBS140506_WB140508b_pIGF1Rb pAKT308 exp2 #8 |
| 196 | Fig5H_pIGF1Rbeta_WBS140519_WB140523_gel 7a8a-pIGF1Rb 7b8b-pS6+p4EBP#8 |
| 197 | Fig5H_tEGFR_WBS131017_WB140113a_WB140113 tEGFR pEGFR #5 |
| 198 | Fig5H_tEGFR_WBS140506_WB140508b_tEGFR pERK #8 |
| 199 | Fig5H_tGAB1_WBS140512_WB140516_gel 1a2a-pGAB1 3a4a-tGAB1 #6 |
| 200 | Fig5H_tGAB1_WBS140519_WB140521_gel 7a8a-pGAB1 9a10a-tGAB1#3 |
| 201 | Fig5J_GAPDH_WBS140512_WB140514b_gel 5a-tEGFR 5b-GAPDH 6a-pEGFR1068 6b-pERK #4 |
| 202 | Fig5J_pEGFR1068_tEGFR_WBS140415_WB140424_pEGFR tEGFR #7 |
| 203 | Fig5J_pIGF1R_WBS140512_WB140516_gel 5a-pIGF1Rb 5b-pAKT308 6a-pGAB1 6b-pAKT308 #6 |
| 204 | Fig5J_pIGF1RbetaGel1_WBS140415_WB140429_pIGF1Rb pS6K #5 |
| 205 | Fig5J_pIGF1RbetaGel2_WBS140415_WB140508_pPDGFRa pIGF1Rb #8 |
| 206 | Fig5J_pRSKgel2_WBS140415_WB140507_pGAB1 pPRAS40 pRSK p4EBP #5 |
| 207 | Fig5J_tEGFR_WBS140512_WB140514b_gel 5a-tEGFR 5b-GAPDH 6a-pEGFR1068 6b-pERK #8 |
| 208 | FigS2D_ACTB_of_POU2F1_WBS130422_WB130514actb_130513actb7flip |
| 209 | FigS2D_ACTB_of_TAZ_NEDD4L_BRAF_ESRRG_YES1_WBS130422_WB130514actb_130513actb7 |
| 210 | FigS2D_BRAF_WBS130422_WB130516orfs_130516orfs8 |
| 211 | FigS2D_ESRRG_WBS130422_WB130508_orfs_WB130508orfs2invert |
| 212 | FigS2D_TAZ_NEDD4L_WBS130422_WB130516orfs_130516orfs9invert |
| 213 | FigS2D_YES1_POU2F1_WBS130422_WB130508_orfs_WB130508orfs3invert |
| 214 | FigS4A_BRAF_GAPDH_WBS131202_WB131206_gel 3 4 BRAF GAPDH #4 |
| 215 | FigS4A_NEDD4L_TAZ_WBS131202_WB131206_gel 1 2 NEDD4L WWTR1 #6w |
| 216 | FigS4A_pERK_WBS131202_WB131209_WB131209 tEGFR pERK #1 |
| 217 | FigS4A_pMEK_WBS131202_WB140106_subImage5 |

| | |
|---|---|
| 218 | FigS4A_tERK_WBS131202_WB140102_gel 3 4 #3 |
| 219 | FigS4A_tMEK_WBS131202_WB140102_gel 1 2 #4 |
| 220 | FigS5A_GAPDH_WBS130904_WB130910_t-mTOR p-AKTS473 p-mTOR GAPDH_invert |
| 221 | FigS5A_NEDD4L_WBS130904_WB130906_NEDD4L_WBS_WB_5 |
| 222 | FigS5E_pSMAD2_3_WBS130729_WB130731_subImage8 |
| 223 | FigS5E_SMAD2_3_GAPDH_WBS130729_WB130730_subImage2 |
| 224 | FigS5F_GAPDH_WBS150323_WB04032015_RerunP10+P12 samples_top(pS6+p4EBP1)_bot(GAPDH) (5) |
| 225 | FigS5F_NEDD4L_WBS150323_WB03302015_RerunP10+P12 samples_top(p_EGFR)_bot(NEDD4L) #5 |
| 226 | FigS5F_pSMAD2_3_WBS150323_WB04082015_top(P16C1-C12)_total smad23_bot(return p10+P12)_p-smad23#5 |
| 227 | FigS5F_SMAD2_3_WBS150323_WB04022015_top(psmad_C1-C12)_bot(total smad_rerunP10+P12)#4 |
